# Parametrization of κ^2^-*N*,*O*-Oxazoline Preligands for Enantioselective Cobaltaelectro-Catalyzed C–H Activations

**DOI:** 10.1021/acscatal.5c00250

**Published:** 2025-02-28

**Authors:** Suman Dana, Neeraj Kumar Pandit, Philipp Boos, Tristan von Münchow, Sven Erik Peters, Sven Trienes, Laura Haberstock, Regine Herbst-Irmer, Dietmar Stalke, Lutz Ackermann

**Affiliations:** WISCh (Wöhler-Research Institute for Sustainable Chemistry), Georg-August-Universität Göttingen, 37077 Göttingen, Germany

**Keywords:** parametrization, ligand design, enantioselective cobalt-catalysis, C–H activation, electrochemistry

## Abstract

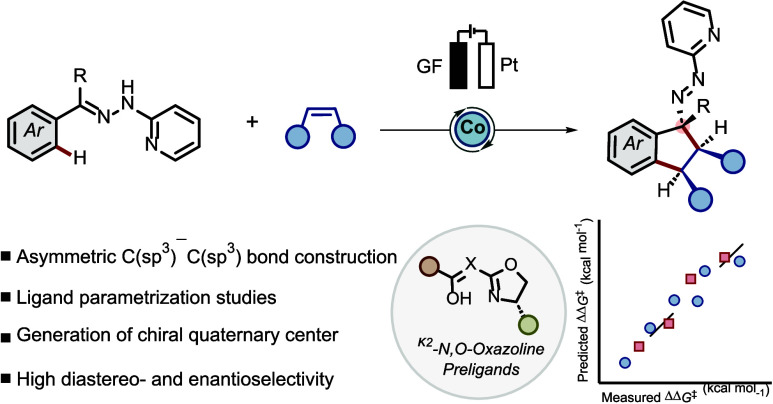

Enantioselective electrocatalyzed C–H activations have emerged as a transformative platform for the assembly of value-added chiral organic molecules. Despite the recent progress, the construction of multiple C(sp^3^)-stereogenic centers via a C(sp^3^)–C(sp^3^) bond formation has thus far proven to be elusive. In contrast, we herein report an annulative C–H activation strategy, generating chiral Fsp^3^-rich molecules with high levels of diastereo- and enantioselectivity. κ^2^-*N*,*O*-oxazoline preligands were effectively employed in enantioselective cobalt(III)-catalyzed C–H activation reactions. Using DFT-derived descriptors and regression statistical modeling, we performed a parametrization study on the modularity of chiral κ^2^-*N*,*O*-oxazoline preligands. The study resulted in a model describing ligands’ selectivity characterized by key steric, electronic, and interaction behaviors.

## Introduction

**Figure 1:**
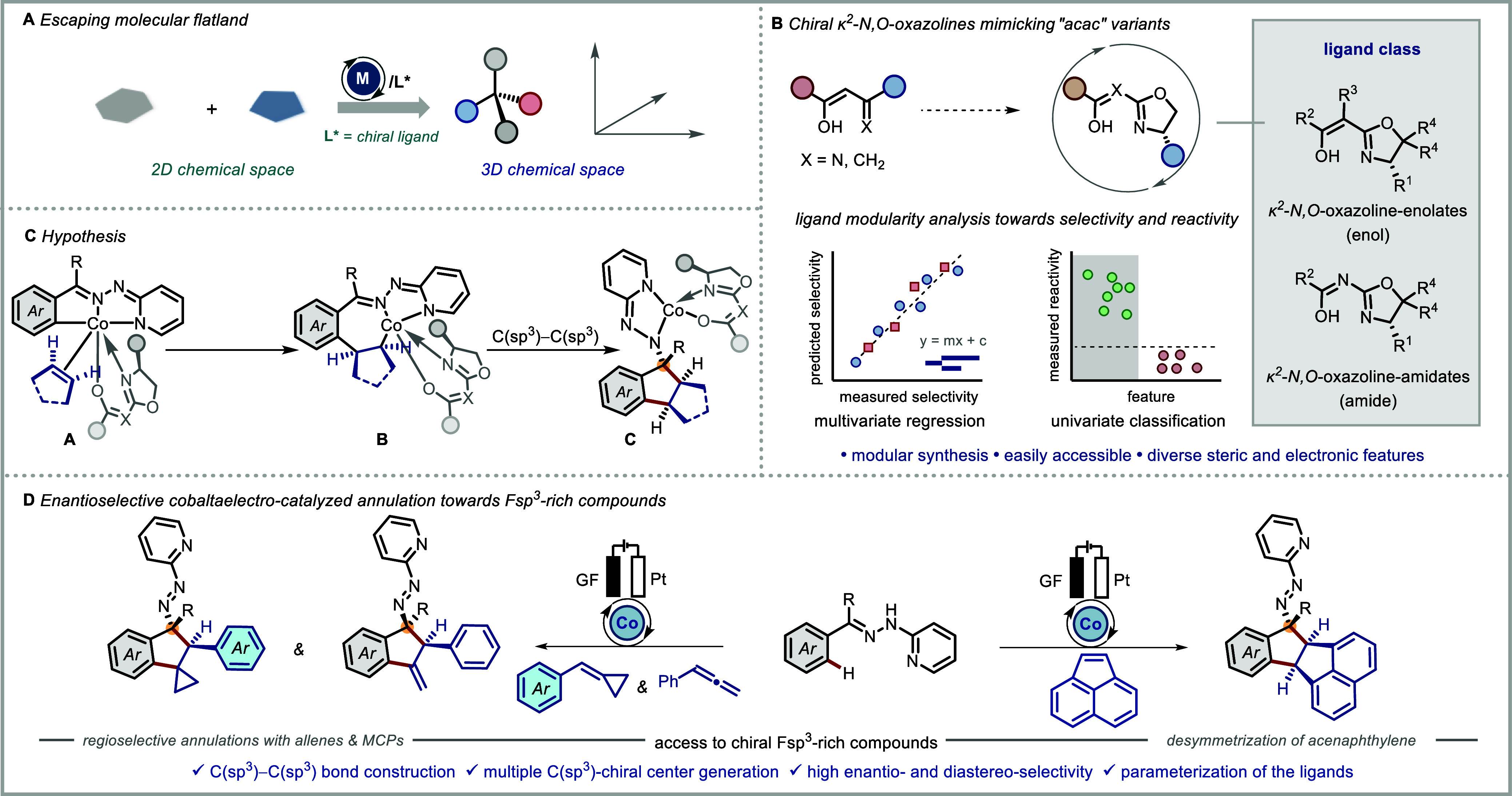
(A) Escaping molecular flatland through transition metal catalysis. (B) Adapted data-driven methods for investigating the κ^2^-*N*,*O*-oxazoline preligands’ modularity toward selectivity and reactivity for reported reaction. (C) Working hypothesis for C(sp^3^)–C(sp^3^) bond formation. (D) Enantioselective cobaltaelectro-catalyzed annulation of olefins by hydrazones.

Molecules embracing a high fraction of chiral sp^3^ carbon atoms (Fsp^3^) feature an increased rate of clinical success owing to their enhanced solubility, accessibility to more target space, and other pharmacological properties.^1−6^ As a consequence, enantioselective transition metal-catalyzed construction of Fsp^3^-enriched molecules remained a dominant area of research in mainstream organic synthesis (Figure 1A).^7−15^ Conceptually, the enantioselective construction of Fsp^3^-enriched molecules generating multiple stereodefined C(sp^3^)-centers and quaternary chiral centers are among the most perplexing and fundamentally challenging formations in synthetic organic chemistry.^7−10,16,17^ Thus, assembling these moieties catalytically has been a cardinal objective for many years. In this context, ligand-enabled enantioselective high-valent cobalt(III)-catalyzed C(sp^2^)–H activations have evolved as a reliable platform to render chiral Fsp^3^-enriched molecules from 2D chemical space.^18−21^ Chiral ligands play a critical role in these transformations, determining the reactivity and selectivity. Thus, de novo design and structural modification of chiral ligands are integral parts of the advancement of this regime.^22−26^ Modular synthesis and on-demand tuning of the steric and electronic features of the ligands are the most sought-after attributes to maneuver reactivity and selectivity. Thus, dedicated efforts to the quest for new ligand classes suitable for these reaction manifolds are necessary to identify new transformations. Thereafter, parametrization of the developed ligands with data-guided approaches can significantly reduce the number of required experiments for the ligand’s successful modulations and provide an in-depth understanding of its structure–property relationships.^27−34^ Additionally, exploring the key interactions in intermediate states with a rational evaluation of the changing structural features strongly impacts the enantio-induction. It affords a predictive model to facilitate further upgrades and evolution of the ligand classes.

Earlier our group contributed in this direction by developing a novel imidazolidine-derived chiral acid for the first enantioselective cobalt(III)-catalyzed C–H activation.^35,36^ Now, with our continuous interest in enantioselective cobaltaelectro-catalyzed C–H activations, we intended to corroborate κ^2^-*N*,*O*-oxazoline-enolates and κ^2^-*N*,*O*-oxazoline-amidates as viable ligand classes in this platform. Notably, these ligand classes structurally mimic the β-ketoenolate^37^ and β-ketoiminate^38,39^ ligands and selected transition metal complexes have been synthesized with κ^2^-*N*,*O*-oxazoline-enolates as ligands (Figure 1B).^40−43^ It is noteworthy that Shi and Niu showcased the applicability of salicyloxazoline (Salox) ligands for oxidative enantioselective cobalt(III)-catalyzed C–H activations.^44,45^ Numerous cobaltaelectro-catalyzed C–H activations with cobalt acetylacetonato (acac) complexes reported by our and other groups^18,20,21^ and mechanistic understanding with electrochemically synthesized isolated cobaltacycle intermediates^46^ encouraged us to assess the applicability of chiral κ^2^-*N*,*O*-oxazoline enolates, and amides in enantioselective C–H activations. These ligands are accessible with modifiable steric and electronic features from simple precursors. To our delight, despite considerable prospects in asymmetric synthesis, these classes of oxazolines are still in a rudimentary stage of evolution and the reactivity and modularity of these ligand classes have never been systematically studied.^47,48^ In an early study, Andersson attempted a copper-mediated enantioselective aziridination of styrene utilizing κ^2^-*N*,*O*-oxazoline-enolates,^48^ albeit with moderate enantioselectivity. Nevertheless, the application of these ligand classes is still elusive in asymmetric transition metal catalysis, especially for enantioselective organometallic C–H activation reactions. Hence, we capitalized on this synthetic space, evaluating the feasibility of these ligands for cobaltaelectro-catalyzed C–H activations along with understanding the key features of the ligand classes through data-guided analysis (Figure 1B).

Notably, enantioselective metallaelectro-catalyzed C–H activation has flourished as a resource-economic transformative tool for the sustainable synthesis of chiral organic molecules.^49−76^ Since our pioneering findings on enantioselective palladium,^59−62^ and 3d transition metal-catalyzed^66−68,77^ electro-oxidative C–H activation strategies, this realm has drawn significant attention from the synthetic community.^65,69−76^ Noteworthily, the advancement in 3d-metallaelectro-catalyzed C–H activations was mostly confined to 8-aminoquinoline directing group for C–H/N–H annulation reactions.^66−68,70,73−75^ The enantioselective construction of 5-membered rings through a dicarbofunctionalization of olefins is rare. We have recently elucidated an enantioselective cobaltaelectro-catalyzed carboacylation of olefins, realized by an intramolecular addition/elimination process involving the electrophilic carbonyl moiety of the benzamide.^77,78^ These transformations essentially relied upon the formation of C(sp^2^)–C(sp^3^) bonds, and the migratory insertion onto the olefin governs the formation of stereogenic centers. Alternatively, aerobic cobalt-catalyzed C–H activation, first described by our group,^79^ was recently applied for a relevant enantioselective domino synthesis of bridged bicycles involving bridged bicyclic olefins.^80^

We envisioned adopting this pursuit for an enantioselective cobaltaelectro-catalyzed C–H activation engaging innate electrophilic hydrazone functionality as the directing group with olefins.^80−82^ We questioned whether novel κ^2^-*N*,*O*-oxazoline ligands could allow an enantioselective annulation following an elusive diastereoselective C(sp^3^)–C(sp^3^) bond construction, accessing chiral Fsp^3^-rich molecules. Eventually, the addition of the C–Co bond of alleged intermediate **B** to the electrophilic C=N bond will govern the formation of a chiral azo compound (Figure 1C).

We herein demonstrate the first successful application of chiral κ^2^-*N*,*O*-oxazolines as ligands in organometallic C–H activations, accomplishing an enantioselective cobaltaelectro-catalyzed annulation toward vicinal chiral C(sp^3^)–C(sp^3^) bonds (Figure 1D). The significance of this ligand class is attributed to the ease of steric and electronic modification on the ligand’s backbone through a modular synthetic route starting from commercially available precursors. Ligand parametrization through computationally guided feature analysis provided a detailed understanding of the steric and electronic descriptors of the preligands. This illustrates their potential for broad usage in enantioselective transition metal catalysis. Salient features of the current strategy are (a) the formation of multiple C(sp^3^)-stereocenters, (b) high diastereo-control of the newly formed C(sp^3^)–C(sp^3^) bond, and (c) the use of simple precursors and inexpensive cobalt catalysts. Our strategy renders diverse peripherally decorated biologically relevant chiral indanes consisting of an aliphatic azo moiety with multiple aliphatic carbon stereocenters (Figure 1D).^83,84^ The azo-functionality can be easily manipulated to valuable biologically relevant chiral amines.^85−88^

## Results and Discussion

**Scheme 1:**
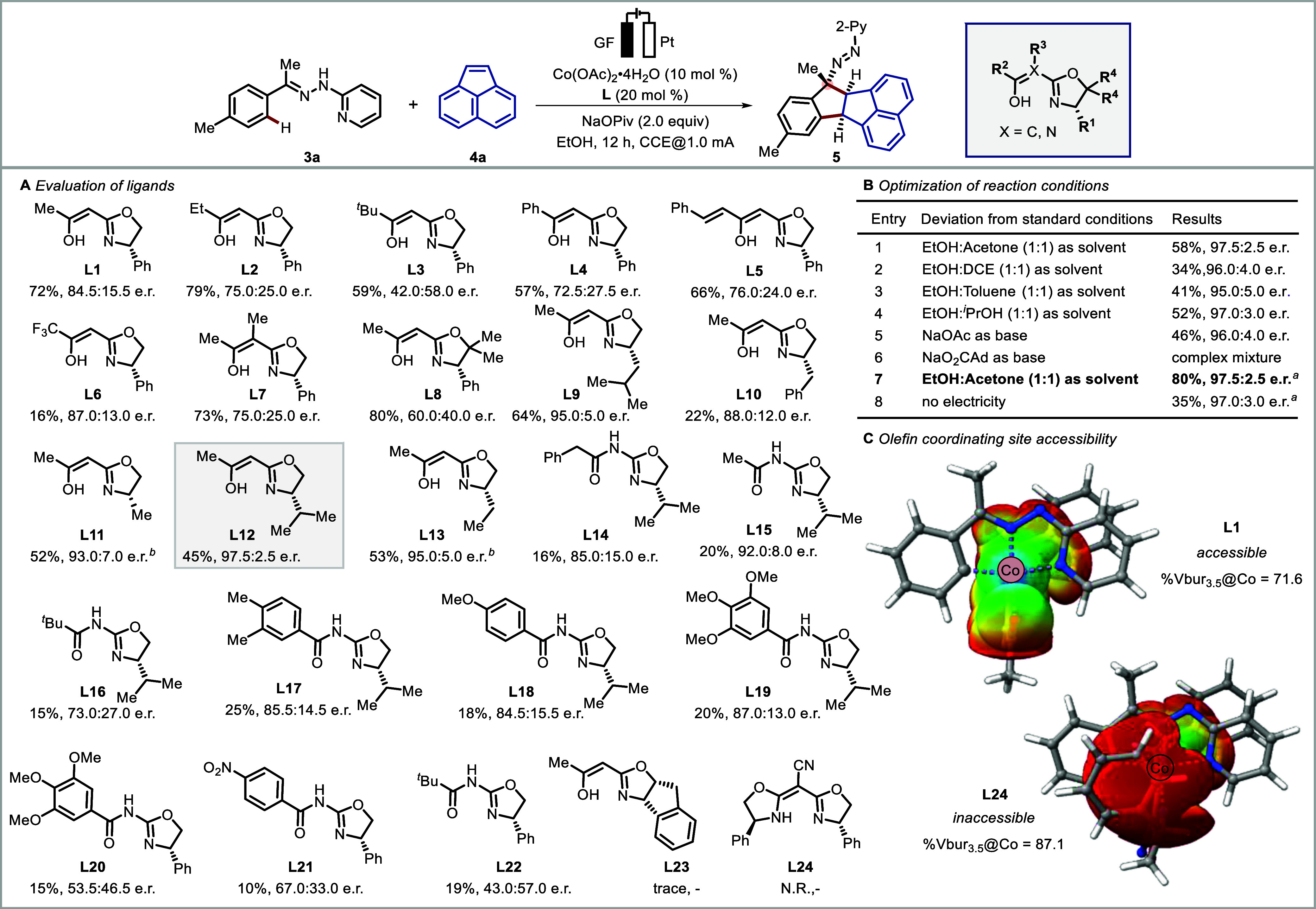
(A) Evaluation of Chiral Preligands; (B) Reaction Optimization; (C) Measured Percentage Buried Volume (% *V*_bur_) at the Cobalt Center for **L1** and **L24** Reaction conditions: undivided cell, **3a** (0.2 mmol), **4a** (0.4 mmol), Co(OAc)_2_·4H_2_O (10 mol %), **L** (20 mol %), NaOPiv (2.0 equiv), in EtOH (5.0 mL) at room temperature with constant current at 1.0 mA for 12 h. ^*a*^Undivided cell, **3a** (0.3 mmol), **4a** (0.6 mmol), Co(OAc)_2_·4H_2_O (15 mol %), **L12** (30 mol %), NaOPiv (2.0 equiv), in EtOH/Acetone = 1:1 (4.0 mL) at room temperature with constant current at 1.0 mA for 24 h. ^*b*^In EtOH/Acetone = 1:1 (5.0 mL). The yield was determined by ^1^H-NMR spectroscopy using 1,3,5-trimethoxybenzene as the internal standard. The enantiomeric ratio (e.r.) was determined by HPLC. d.r. > 20:1.

We initiated our studies by synthesizing a library of κ^2^-*N*,*O*-oxazoline-enolate (**L1**–**L13**) and κ^2^-*N*,*O*-oxazoline-amidate (**L14**–**L22**) preligands (see the Supporting Information). We considered hydrazone **3a** and olefin **4a** as the model substrates for our study. Gratifyingly, the application of electro-oxidative conditions with graphite felt (GF) anode and Pt-cathode in ethanol medium combining Co(OAc)_2_·4H_2_O catalyst, preligand **L1**, and NaOPiv base delivered the desired product **5** in 72% yield, 84.5:15.5 e.r., and >20:1 d.r (Scheme 1A). We continued our study by examining the library of synthesized preligands for the desired transformation (Scheme 1A). Systematic studies revealed that the enantioselectivity of the transformation is highly sensitive to the steric features of the ligand. Substitution in the enol backbone plays a crucial role in determining the enantioselectivity. The enantiomeric ratio (e.r.) drops significantly as the steric hindrance increases at the *R*^2^ position (**L2**–**L5**). The presence of an electron-withdrawing group in the preligand backbone (**L6**) provided inferior results. Increasing the steric hindrance at *R*^3^ (**L7**) further diminished the e.r. significantly. Next, the substitution pattern at the oxazoline moiety was examined (**L9**–**L13**), and the valinol-derived preligand (**L12**) performed particularly well, offering the highest er of 97.5:2.5. Sterically hindered oxazoline-derived preligand **L8** displayed a reduced enantioselectivity. A similar trend was also observed for the κ^2^-*N*,*O*-oxazoline-amidate preligands (**L14**–**L22**), although reasonably poor yields were obtained in these cases. Noticeably, electron-deficient groups in the enol or amide motifs showcased detrimental effects, resulting in poor yields of **5**. Moreover, the observed ligands’s tautomerism was shown to have no effect on the enantioinduction. Then, various common organic solvents were probed, and a mixture of ethanol and acetone served best among others (Scheme 1B, entries 1–4). Further experimentation with different carboxylate bases revealed NaOPiv as the optimal choice for this transformation (Scheme 1B, entries 5–7). It is worth noting that electrolysis of the reaction mixture was necessary to furnish high yields of the desired product (Scheme 1B, entry 8). No product was observed with **L24** due to the ligand’s steric hindrance, which obstructs the olefin coordination after hydrazone and ligand complexation.

We further quantified the coordinating site accessibility using the percentage buried volume (% *V*_bur_) at the cobalt. An exceedingly high % *V*_bur_ (87.1) was observed for **L24** compared to the rest of the ligand set, which present % *V*_burs_ ranging from 70.4 to 76.1 (Scheme 1C).

**Figure 2:**
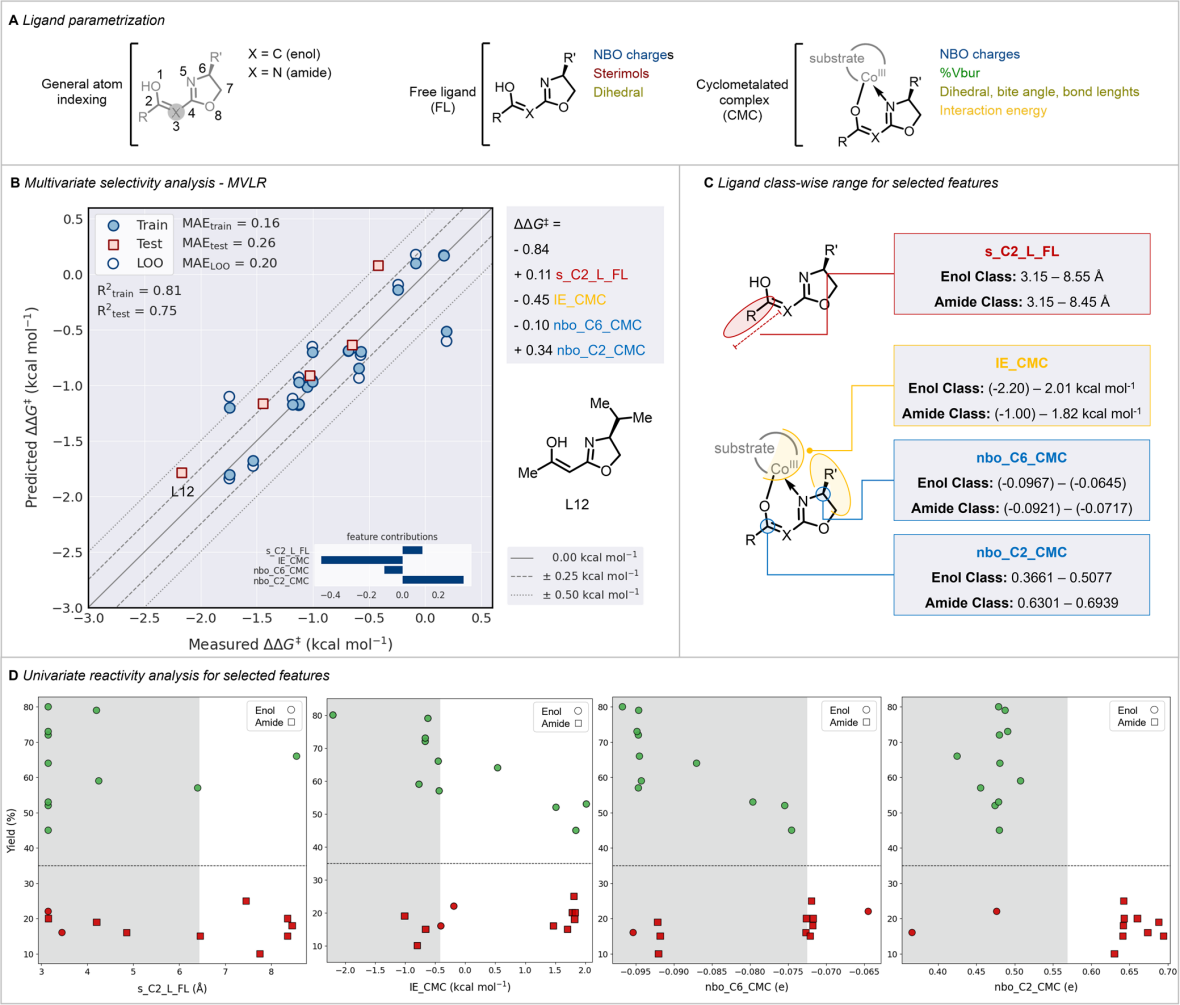
(A) Ligand parametrization. (B) Statistical modeling: MVLR model obtained for the ligand-induced selectivity. (C) Key features obtained from the M4 model for the selectivity. (D) Univariate classification for the key features against reaction yield.

Next, we sought to gain additional insight into the features of the novel oxazoline preligands. The fundamental understanding of the ligand’s structural and electronic modularity provides reasonable mechanistic insights,^89^ efficient ligand tuning^90^ with wide applicability, and reaction optimizations. Thus, a comprehensive theoretical parametrization study was performed to develop a quantitative structure–enantioselectivity relationship model for the ligands (see the Supporting Information for details).^91,92^ Several DFT-based descriptors were obtained from the optimized free preligand (FL) structures and cyclometalated cobalt(III)-complexes (CMC) (Figure 2A). Thus, the properties of the isolated preligand and the complex formed were taken into consideration. All possible combination-based multivariant linear regression (MVLR) models were constructed and tested up to six parameters. From the performance evaluation, the four-parameter-based model (M4) gave satisfactory selectivity predictions for both (Figure 2B) train (*R*^2^ score = 0.81, MAE = 0.16 kcal mol^–1^) and test (*R*^2^ score = 0.75, MAE = 0.26 kcal mol^–1^) samples. Additionally, the model also performed well (MAE = 0.20 kcal mol^–1^) during the leave-one-out (LOO) train sample cross-validation, which demonstrated a robust and good generalization ability toward both samples. Notably, the four-parameter-based M4 model comprised a sterimol component at the C2 substituent (s_C2_L), the natural bonding orbital (NBO) charges at C2 and C6 (nbo_C2, nbo_C6), and the probed interaction energy (IE)^93^ between the ligand as well as the substrate. The model suggested a significant role of nbo_C6 and IE parameters of C6 substituents in stabilizing the substrate plane, while the C2 site of the ligand demanded lower electron density to induce better selectivity for the given reaction (Figure 2C). For a given reaction, the trade-offs between a ligand’s selectivity and reactivity play a critical role.^95^ Thus, to further evaluate the contributing features obtained from the M4 model, we performed a featurewise univariate reactivity (yield) classification to identify the feature thresholds for higher ligand reactivity (Figure 2D) (see the Supporting Information for details). The classification identified 6.42 Å as the maximum threshold for s_C2_L, suggesting that substitutions above this threshold would result in lower yields. For the IE, a minimum threshold of −0.421 kcal mol^–1^ (stabilizing interaction) was obtained. Interestingly, a pattern was observed in the substituents at C6: all the alkyl substituents located in the region, where IE > −0.421 kcal mol^–1^, presented lower yields. In contrast, phenyl-substituted oxazoline ligands, which occurred in the opposite region, presented higher yields. The ligand **L8**, with the highest stabilizing IE, conferred the highest yield. The obtained maximum thresholds for nbo_C6 and nbo_C2 were −0.07 and 0.57, respectively. The nbo_C2 was classified most accurately, revealing that ligands with an NBO charge at the C2 carbon higher than 0.57 will provide low yields, where the entire tested amide ligand class was present. Thus, based on the developed model; the nbo_C2_CMC, nbo_C6_CMC, s_C2_L_FL, and IE_CMC are the most important features from the ligand determining the stereoselectivity. Moreover, nbo_C2_CMC and IE_CMC were also found to better predict the reactivity thresholds of the ligand. With the identified selectivity model and the obtained threshold values, virtual screening of the ligand classes can be efficient in determining ligands with higher selectivity as well as reactivity.

**Figure 3:**
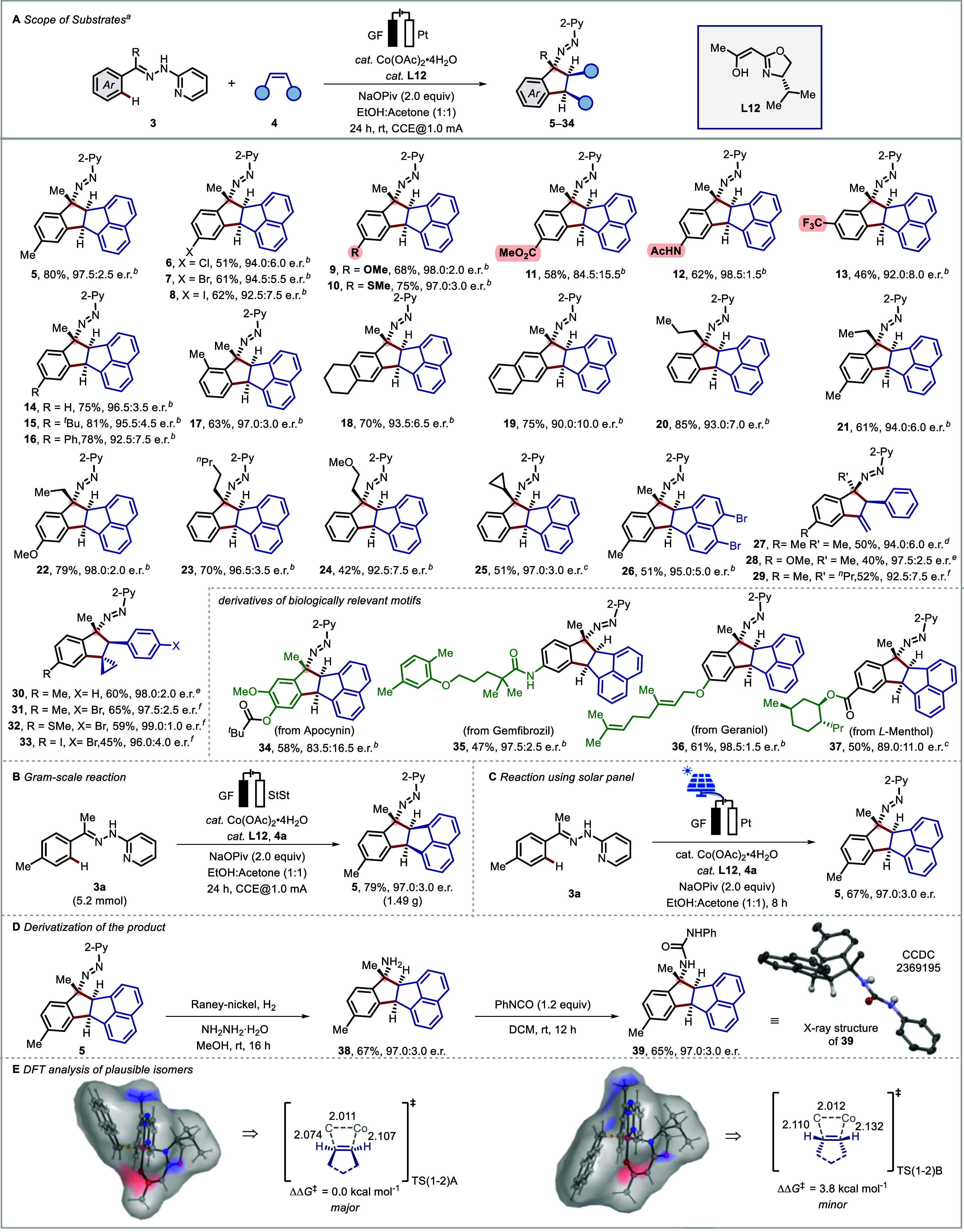
Scope of the methodology. (A) Scope of substrates: ^a^Reaction conditions: undivided cell, **3** (0.3 mmol), **4** (0.6 mmol), Co(OAc)_2_·4H_2_O(15 mol %), **L12** (30 mol %), NaOPiv (2.0 equiv), in ethanol/acetone = 1:1 (4.0 mL) at room temperature with constant current at 1.0 mA for 24 h. ^b^d.r.>20:1. ^c^d.r. = 10:1. ^d^d.r. = 5:1. ^e^d.r. = 4:1. ^f^d.r. = 3:1. (B) Gram-scale reaction. (C) Reaction using a solar panel. (D) Derivatization of the product. (E) Performed DFT analysis of the olefin migratory insertion step for the formation of stereoisomers at the PW6B95-D4/def2-TZVPP + SMD (ethanol and acetone)//TPSS-D3(BJ)/def2-SVP level of theory with key distances given in Å. The enantiomeric ratio (e.r.) was determined by chiral HPLC. The absolute configuration of molecular structures shown was derived from the obtained X-ray structure of **39**.^94^

Subsequently, we focused on evaluating the viable substrate scope of the desired transformation (Figure 3A). First, different substitutions on the aromatic ring of the hydrazone were examined (**5**–**19**). Sensitive halogen functionalities (**6**–**8**), ether (**9**), thioether (**10**), carboxylate ester (**11**), and acetamido (**12**) moieties were tolerated under the reaction conditions, furnishing the desired products in synthetically useful yields and high e.r. Alkyl substituents at different positions of the aromatic ring delivered the desired annulated products (**13**–**17**) in good yield with high enantioselectivity (up to 97.0:3.0 e.r.). Sterically hindered hydrazone analogues furnished annulated products (**18**–**19**) at the sterically less hindered site. Second, alkyl substituents on the hydrazone were examined. Alkyl groups of various lengths (**20**–**24**) and a cyclopropyl moiety (**25**) were accommodated, gaining access to corresponding products with high selectivity. The presence of an ether moiety in the aliphatic chain (**24**) was also suitable for this transformation. In all the cases, very high diastereoselectivities could be achieved (up to >20:1).

Third, we attempted to explore the scope of olefins in the cobaltaelectro-catalysis. Bromo substitution on the acenaphthylene was compatible allowing further modification of the product (**26**). Further, allenes also showcased a similar reactivity to generate the structurally interesting chiral indanes in moderate yields and good enantioselectivity (**27**–**29**). Moreover, methylene cyclopropanes (MCPs) were considered as potential reaction partners. To our delight, MCPs efficiently participated in the transformation, delivering structurally diverse chiral indanes in high enantioselectivities (**30**–**33**). Derivatives of biologically relevant apocynin (**34**), gemfibrozil (**35**), geraniol (**36**), and *L*-menthol (**37**) were also amenable to this protocol, generating the functionalized products in good yields and selectivities.

To showcase the synthetic utility, we next assessed the scalability of the process. Delightfully, we observed a similar level of efficacy on a gram scale, where the desired product **5** was obtained in comparable yield and enantioselectivity (Figure 3B). Noticeably, in this case, the reaction was performed with inexpensive stainless steel (StSt) as the cathode instead of a Pt plate (Figure 3B). Furthermore, a solar panel could also be employed as a sustainable source of electricity to effectuate renewable solar energy to promote chemical transformation, highlighting the tolerance of the catalytic conditions to the current fluctuations. Gratifyingly, the reaction provided a 67% yield of **5** with a similar level of enantioselectivity (Figure 3C).

As ambitioned, the chiral azo compounds were transformed to chiral amines through a simple Raney-Ni reduction at room temperature, where the respective chiral amine **38** was obtained in 67% yield and 97.0:3.0 e.r (Figure 3D). The amine **38** was further modified to the chiral urea analogue **39**, which allowed the determination of the absolute configuration by single crystal X-ray crystallographic analysis (Figure 3D).^94^

In order to gain insights into the observed enantioselectivity, DFT calculations were performed for the migratory insertion elementary step in the presence of preligand **L12** (Figure 3E). Calculations revealed that the major enantiomer was energetically favored over the minor enantiomers by 3.8 kcal mol^–1^, which is in agreement with the experimental findings (see the Supporting Information for details).

**Figure 4:**
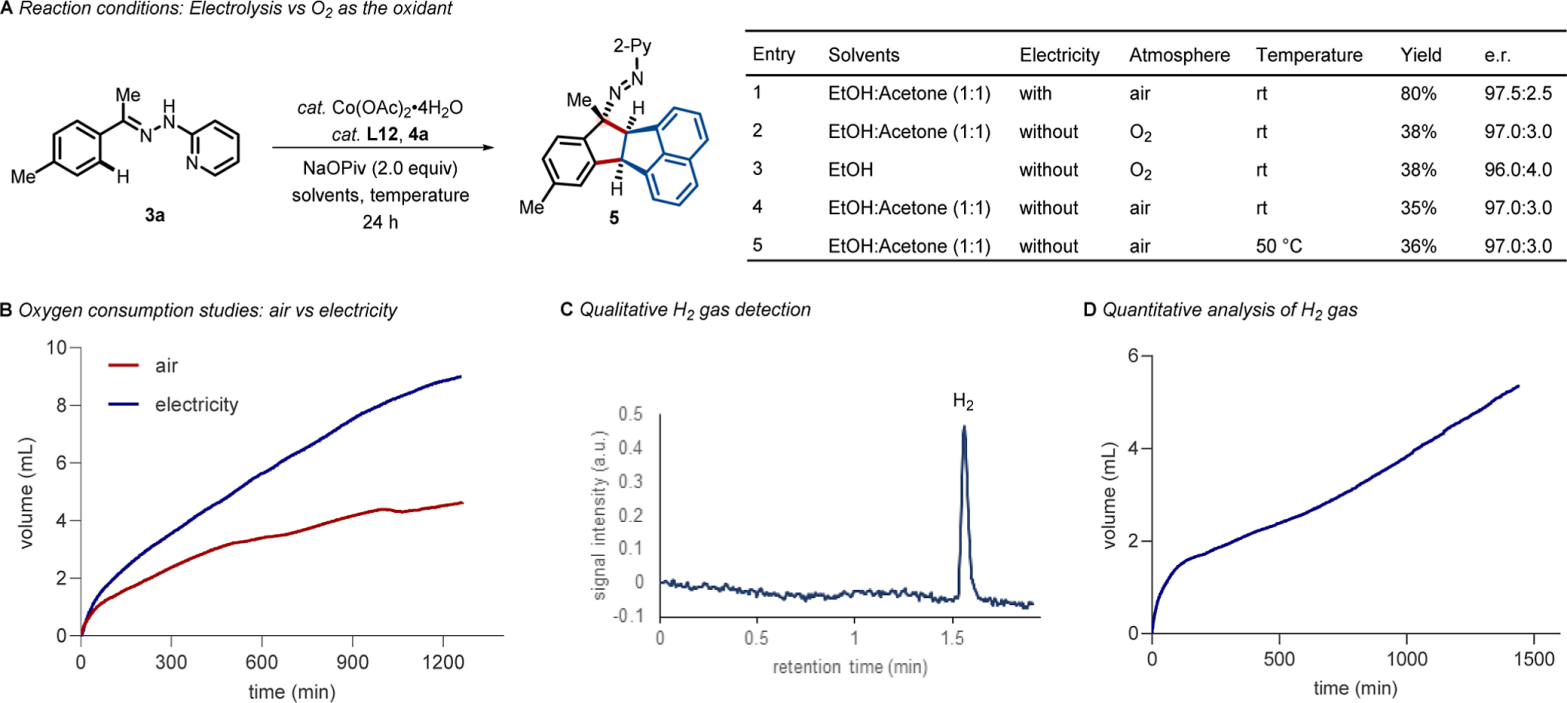
Mechanistic insights. (A) Reaction under O_2_, air atmosphere, and electrocatalysis. (B) Reaction under an inert atmosphere. (C) Qualitative detection of molecular hydrogen. (D) Quantification of molecular hydrogen.

To illustrate the indispensable role of electrolysis for the reaction outcome, we conducted experiments under pure O_2_ as a terminal oxidant (Figure 4A). The yields were significantly reduced to less than 40%. To identify the cathodic half-reaction, we systematically analyzed the headspace of the reaction vessel using headspace gas chromatography. The oxygen reduction reaction was comprehended as a possible cathodic reaction in the transformation (see Figure S4 in the Supporting Information), and the oxygen consumption was faster in the presence of electricity (See the Supporting Information for details). Literature precedents on the electrochemical synthesis of azo compounds further supported our analysis.^96−99^ The oxygen consumption was further quantitatively measured, where significantly accelerated oxygen consumption and product formation were observed under mild electrochemical conditions (Faradaic efficiency = 22%) (Figure 4B). In this context, a meticulously planned combination of anodic oxidation with a hydrogen evolution reaction (HER) bears a distinguished potential toward a green hydrogen economy. Thus, the reaction was performed under an inert atmosphere (see the Supporting Information), where the cathodic reaction was switched to HER as the main pathway. Unlike the aerobic conditions, hydrogen gas formation was detected from the headspace of the reaction vessel during the transformation (Figure 4C). We also quantitatively determined the evolution of hydrogen gas (*n*_H2_/*n*_**5**_ = 0.43), demonstrating cobaltaelectro-catalysis as a sustainable technique for the merger with HER (Figure 4D). Additionally, the catalytic reaction was monitored via high-resolution mass-spectrometry, where the formations of heteroleptic cobalt(II)-complex and cobalt(III)-complex were detected as crucial intermediates (see the Supporting Information for details).

## Conclusions

In summary, we have devised an enantioselective cobaltaelectro-catalyzed annulation between aryl hydrazones and olefins, constructing chiral Fsp^3^-rich indanes. The current method is mild, operationally simple, and scalable. A notable feature of the present strategy is the highly enantio- and diastereoselective C(sp^3^)–C(sp^3^) bond formation. Simultaneously, the untapped potential of chiral κ^2^-*N*,*O*-oxazoline preligands was exploited for an enantioselective C–H activation strategy. This preligand class allows for modular stereoelectronic modifications, offering beneficial features for asymmetric transition metal catalysis in general. DFT feature-based multivariate regression modeling disclosed a suitable MVLR model describing the ligand’s modularity toward diverse electronic, steric, and interaction behavior influencing the enantioselectivity. Furthermore, the univariate classification unveiled the role of IE and NBO charge influencing the reaction yield. The in-depth analysis and the generated model can facilitate the rational design and efficient screening of novel κ^2^-*N*,*O*-oxazoline preligands, extending its applicability toward other asymmetric transformations. We anticipate that our findings provide a valuable platform for chiral ligands and expect their use in asymmetric transition metal catalysis in the future.

## Material and Methods

### General Procedure for the Catalytic (3 + 2)-Annulation Reaction

The electrolysis was carried out in an undivided cell setup. A GF anode (10 mm × 15 mm × 6 mm) and a platinum cathode (25 mm × 10 mm × 0.125 mm) with an electrode holder made of stainless steel were used. The cell was charged with the hydrazone **3** (0.30 mmol, 1.0 equiv), olefin **4** (0.60 mmol, 2.0 equiv), and PivONa (0.6 mmol, 2.0 equiv) and a Teflon-coated magnetic stirring bar (15× 6 mm). Then Co(OAc)_2_·4H_2_O (15 mol %) and the ligand **L12** (30 mol %) were added into it along with the addition of 2 mL of EtOH and Acetone each. The resulting mixture was stirred for 10 min to make a homogeneous solution. Afterward, the electrolysis was performed at room temperature with a constant current of 1.0 mA maintained for 24 h with a stirring rate of 500 rpm. After completion of the reaction, the reaction mixture was diluted with 2 mL of ethyl acetate and transferred to a round-bottom flask. The electrodes (platinum and GF) were washed in the reaction flask with ethyl acetate (3 × 5 mL) in an ultrasonic cleaner (3 × 3 min), and the washes were combined in the round-bottom flask. The solvent was then removed under vacuum and the residue was purified by column chromatography (ethyl acetate/*n*-hexane) to afford the title compound (refer to the Supporting Information for additional details).

## Supporting Information Available

All experimental procedures, optimization studies, racemization experiments, mechanistic experiments, characterization of the new compounds, HPLC chromatograms, NMR spectra, and crystal data; computational details from the ligand parametrization, statistical modeling and code repository link, univariate classification and cartesian coordinates of optimized structures from the DFT studies are provided (PDF)
X-ray structure of compound 39 (CIF)

## Supplementary Material

cs5c00250_si_001.pdf

cs5c00250_si_002.cif
