# Supplementary material for: Parametrization of κ2-N,O-Oxazoline Preligands for Enantioselective Cobaltaelectro-Catalyzed C–H Activations
Source: ACS Catal. 2025 Feb 28;15(6):4450–9. doi: 10.1021/acscatal.5c00250 (PMC11934137; doi:10.1021/acscatal.5c00250)
Supplement: Supplementary file 1 — cs5c00250_si_001.pdf [file cs5c00250_si_001.pdf]

Supporting Information

# Parametrization of $\kappa^2$-*N*,*O*-Oxazoline Preligands for Enantioselective Cobaltaelectro-catalyzed C–H Activations

Suman Dana,[a‡] Neeraj Kumar Pandit,[a‡] Philipp Boos,[a‡] Tristan von Münchow,[a] Sven Erik Peters,[a] Sven Trienes,[a] Laura Haberstock,[a] Regine Herbst-Irmer,[a] Dietmar Stalke,[a] Lutz Ackermann[a]*

[a]WISCh (Wöhler-Research Institute for Sustainable Chemistry), Georg-August-Universität Göttingen, 37077 Göttingen, Germany.

‡These authors contributed equally to this work.

*Corresponding author. E-mail: Lutz.Ackermann@chemie.uni-goettingen.de

# Table of Contents

**Materials and Methods** ........ 4
**General Remarks** ........ 4
**Synthesis of Substrates** ........ 5
**Characterization Data for the Hydrazones** ........ 5
**Synthesis of Ligands** ........ 9
General Procedure 1 (GP1): Synthesis of Enol-Oxazoline Ligands ........ 9
General Procedure 2 (GP2): Synthesis of Enol-Oxazoline Ligands ........ 9
General Procedure 3 (GP3): Synthesis of Enol-Oxazoline Ligands ........ 10
General Procedure 4 (GP4): Synthesis of Enol-Oxazoline Ligand ........ 10
General Procedure 5 (GP5): Synthesis of Amido-Oxazoline Ligands ........ 11
General Procedure 6 (GP6): Synthesis of Amido-Oxazoline Ligands ........ 11
**Characterization data of the synthesized ligands** ........ 12
**Optimization of Reaction Conditions** ........ 24
Screening of Ligands ........ 24
Optimization of Reaction Conditions ........ 25
**General Procedure for the Catalytic (3+2)-Annulation Reaction (GP7)** ........ 26
**General Procedure for the Catalytic (3+2)-Annulation Reaction in gram-scale (GP8)** ........ 26
**General Procedure for the Catalytic (3+2)-Annulation Reaction using a Solar Panel (GP9)** ........ 28
**NMR Data of Synthesized Compounds:** ........ 29
**Derivatization of the Products** ........ 66
General Procedure for the Reduction of the Azo Compound 5 to Amine 38 (GP10) ........ 66
General Procedure for the Synthesis of Urea 39 (GP11) ........ 67
Qualitative Analysis of Electrochemical Oxygen Reduction during the Catalysis ........ 68
Qualitative Analysis of Electrochemical Oxygen Reduction during the Catalysis ........ 71
**Reaction under air- Oxygen Gas Consumption Studies** ........ 71
Qualitative Detection of Molecular Dihydrogen as the Byproduct of Catalysis ........ 72
Quantitative Detection of Molecular Dihydrogen as the Byproduct of Catalysis ........ 73
Monitoring by Mass Spectrometry ........ 75
**Crystallographic data of 38** ........ 78
General Data Acquisition and Processing ........ 78
**Ligand Parameterization** ........ 83
Ligand Screening Data Used for Feature Analysis ........ 83
Features (descriptor) Calculations and Analysis ........ 83
Percentage Buried Volume (%Vbur) ........ 84
Interaction Energy (IE) Calculation ........ 85
Statistical Modeling ........ 87

Ligand Reactivity Analysis ........ 90
**Computational Studies** ........ 91
Method Details ........ 91
Cartesian Coordinates of the Optimized Structures from Computational Studies ........ 94
**References** ........ 140
**NMR Characterization Data of the Synthesized Hydrazone Substrates** ........ 142
**NMR Characterization Data of the Synthesized Ligands** ........ 152
**NMR Characterization Data of the Synthesized Compounds** ........ 176
**Representative 2D NMR Spectra of the Synthesized Compounds** ........ 212

# Materials and Methods

## General Remarks

Solvents for column chromatography and extraction (EtOAc, *n*-hexane, *n*-pentane, $Et_2O$, DCM) were distilled before their use. Routine TLC analysis was carried out on aluminum sheets coated with silica gel 60 F254, 0.2 mm thickness. Plates were analyzed using a 254 nm UV lamp. Chromatography was carried out on Merck silica gel 60 (40–63 µm).

Platinum electrodes (10 mm × 15 mm × 0.25 mm, 99.9%; obtained from ChemPur® Karlsruhe, Germany) and graphite felt (GF) electrodes (10 mm × 15 mm × 6 mm, SIGRACELL®GFA 6 EA, obtained from SGL Carbon, Wiesbaden, Germany) were connected using stainless steel adapters. Electrocatalysis was conducted using an AUTOLAB multichannel Line from METROHM or Rohde & Schwarz Galvanostat (HMP4040). Electrocatalysis powered by sunlight was executed using a commercially available photovoltaic cell

(Conrad Electronic SE, TPS-103 6 W, 17.5 V max. voltage, 428 mA max. current, 467 mm x 161 mm x 19 mm), where the output current was controlled with a customized and normalized constant current regulator and double-checked with an ammeter.

NMR spectra were collected on the Bruker Avance 300, Bruker Avance Neo 400 at 300 MHz, 400 MHz, ($^1$H-NMR), 75 MHz, 101 MHz ($^{13}$C-NMR), 162 MHz ($^{31}$P NMR) and 282 MHz, 377 MHz ($^{19}$F-NMR). Chemical shifts are stated as $\delta$-values in parts per million (ppm) referenced to the residual proton peak of the deuterated solvent ($^1$H; $CDCl_3$: 7.26 ppm, $CD_3OD$: 4.87 ppm) or the carbon peak of the solvent ($^{13}$C: $CDCl_3$: 77.16 ppm, $CD_3OD$: 49.00 ppm). Coupling constants $J$ are reported in Hertz (Hz). The evaluation of the NMR spectra was carried out with the software MNova NMR v 10.0.2 from Mestrelab Research. Yields refer to isolated compounds, estimated to be >95% pure as determined by $^1$H-NMR spectroscopy. The chiral azo compounds are unstable and undergo slow decomposition in solution. IR spectra were recorded on a Bruker FT-IR Alpha-P instrument and were recorded in the range from 4000 to 400 cm$^{-1}$. Electrospray-ionization mass spectra (ESI-MS) were obtained on a Thermo Scientific Exactive Plus equipped with an orbitrap mass analyzer and Bruker Daltonics maXis instrument in positive ion mode. Headspace analysis of the reaction mixture was performed on 7890B gas chromatograph (GC) system by Agilent Technologies. Melting points were determined on a Stuart melting point apparatus SMP3 from Barloworld Scientific. The values are uncorrected.

HPLC chromatograms were recorded on an Agilent 1290 Infinity using CHIRALPAK® IA-3, IB-3, IC-3, ID-3, IE-3, IF-3, columns (3.0 µm particle size; Ø: 4.6 mm and 250 mm length). Optical rotations were measured with Anton Paar MCP 150 at 20 °C under a Na/Hg lamp, $\lambda$ = 589 nm (c in g/100 mL). Values were denoted as specific rotations: $[\alpha]_D^{20}$.

X-ray diffraction experiments for the compounds analyzed were carried out at 100(2) K on a Bruker D8 Venture four-circle diffractometer from Bruker AXS GmbH. A Photon II detector purchased from Bruker AXS GmbH and microfocus IµS 2.0 Cu/Mo and microfocus IµS 3.0 Ag/Mo from Incoatec GmbH with mirror optics HELIOS and single-hole collimator from Bruker AXS GmbH were employed. The detection was carried out via the Photon III CE14 (Cu/Mo) and Photon III HE (Ag/Mo) from Bruker AXS GmbH. The programs used were APEX3 Suite, SAINT V8.38A, and SADABS 2016/2 from Bruker AXS GmbH. Furthermore, SHELXT and SHELXL-2018/3 were used. The data was visualized using Mercury. Non-hydrogen atoms were refined anisotropically, while the carbon-bound hydrogen atoms were located geometrically and refined with a riding model.

## Synthesis of Substrates

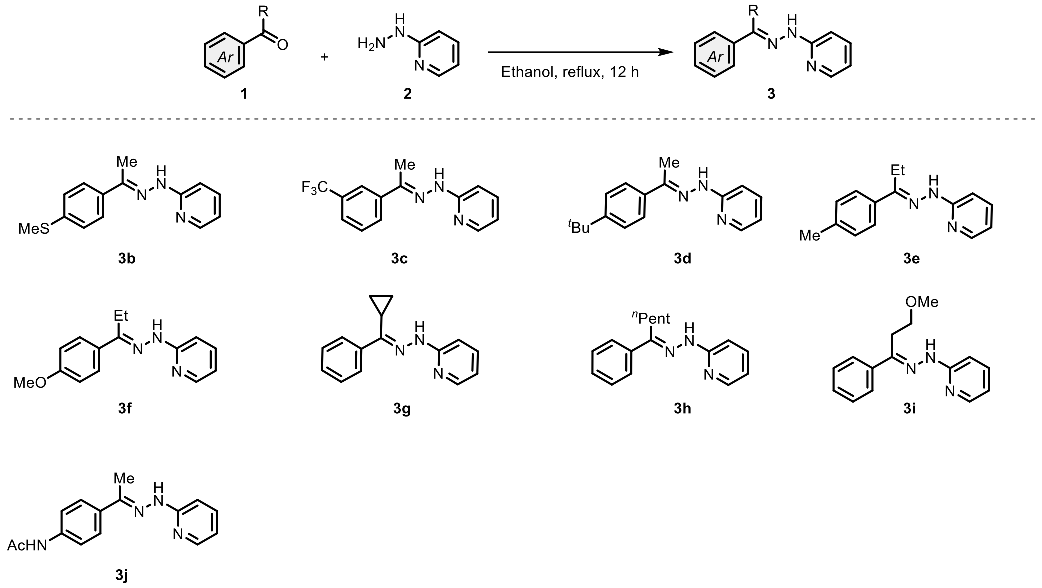

**Figure S1.** Synthesis of hydrazones.

*Synthesis of Hydrazones:* Hydrazones were synthesized following the available literature procedure.[1] In an oven-dried round-bottom flask charged with a magnetic stirring bar, corresponding ketone (**1**) (10.0 mmol, 1.0 equiv.) and 2-Hydrazinopyridine (**2**) (12.0 mmol, 1.1 equiv.) were added. Next, 20 mL of Ethanol was added into the mixture and the solution was refluxed overnight. Then, the solvent was evaporated using a rotary evaporator and the crude mixture was purified through column chromatography.

## Characterization Data for the Hydrazones

### (*E*)-2-(2-(1-(4-(Methylthio)phenyl)ethylidene)hydrazineyl)pyridine (3b):

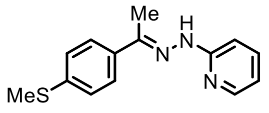

Prepared according to the general procedure on a 10.0 mmol scale, column chromatography (*n*-hexane/ethyl acetate = 2:1) afforded the title compound as a yellow solid; Yield: 93 % (2.39 g, 9.3 mmol).

**$^1$H-NMR** (400 MHz, $CDCl_3$) δ 8.17 – 8.11 (m, 1H), 8.05 (s, 1H), 7.74 – 7.70 (m, 2H), 7.64 – 7.59 (m, 1H), 7.42 – 7.37 (m, 1H), 7.27 – 7.24 (m, 2H), 6.81 – 6.76 (m, 1H), 2.51 (s, 3H), 2.23 (s, 3H) ppm;

**$^{13}$C-NMR** (101 MHz, $CDCl_3$) δ 157.2 ($C_q$), 147.8 (CH), 142.5 ($C_q$), 139.0 ($C_q$), 138.3 (CH), 135.7 ($C_q$), 127.1 (CH), 126.3 (CH), 126.2 (CH), 125.9 (CH), 116.0 (CH), 107.8 (CH), 15.8 ($CH_3$), 12.2 ($CH_3$) ppm;

**IR** (ATR): $\tilde{\nu}$ = 2920, 1596, 1574, 1489, 1441, 1292, 1140, 770 cm$^{-1}$;

**HRMS (ESI):** *m/z* $[M+H]^+$ calcd for $C_{14}H_{16}N_3S$: 258.1065; found: 258.1060.

**(*E*)-2-(2-(1-(3-(Trifluoromethyl)phenyl)ethylidene)hydrazineyl)pyridine (3c):**

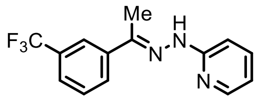

Prepared according to the general procedure on a 10.0 mmol scale, column chromatography (*n*-hexane/ethyl acetate = 2:1) afforded the title compound as a yellow viscous oil; Yield: 67 % (1.87 g, 6.7 mmol).

**$^1$H-NMR** (300 MHz, $CDCl_3$) δ 8.23 (s, 1H), 8.18 – 8.14 (m, 1H), 8.03 (s, 1H), 7.95 (d, $J$ = 7.8 Hz, 1H), 7.68 – 7.61 (m, 1H), 7.59 – 7.54 (m, 1H), 7.52 – 7.46 (m, 1H), 7.44 – 7.39 (m, 1H), 6.84 – 6.79 (m, 1H), 2.27 (s, 3H) ppm;

**$^{13}$C-NMR** (75 MHz, $CDCl_3$) δ 156.9 ($C_q$), 147.8 (CH), 141.3 ($C_q$), 139.6 ($C_q$), 138.4 (CH), 130.9 (q, $J_{CF}$ = 32.3 Hz, $C_q$), 129.0 (CH), 128.9 (CH), 128.9 (CH), 126.1 ($C_q$), 124.8 (q, $J_{CF}$ = 3.7 Hz, CH), 122.5 (q, $J_{CF}$ = 3.9 Hz, CH), 116.5 (CH), 107.9 (CH), 12.3 ($CH_3$) ppm;

**$^{19}$F-NMR** (282 MHz, $CDCl_3$) δ -62.66 ppm;
**IR** (ATR): $\tilde{\nu}$ = 3191, 1596, 1574, 1437, 1320, 1267, 1165, 1119 $cm^{-1}$;

**HRMS (ESI):** *m/z* $[M+H]^+$ calcd for $C_{14}H_{13}F_3N_3$: 280.1062; found: 280.1063.

**(*E*)-2-(2-(1-(4-(*tert*-Butyl)phenyl)ethylidene)hydrazineyl)pyridine (3d):**

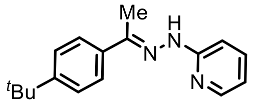

Prepared according to the general procedure on a 10.0 mmol scale, column chromatography (*n*-hexane/ethyl acetate = 2:1) afforded the title compound as a yellow solid; Yield: 93% (2.49 g, 9.3 mmol).

**$^1$H-NMR** $^1$H-NMR (300 MHz, $CDCl_3$) δ 8.17 – 8.10 (m, 1H), 8.03 (s, 1H), 7.76 – 7.69 (m, 2H), 7.64 – 7.58 (m, 1H), 7.44 – 7.38 (m, 3H), 6.81 – 6.75 (m, 1H), 2.26 (s, 3H), 1.34 (s, 9H) ppm;

**$^{13}$C-NMR** (75 MHz, $CDCl_3$) δ 157.3 ($C_q$), 151.7 ($C_q$), 147.8 (CH), 143.2 ($C_q$), 138.2 (CH), 136.2 ($C_q$), 125.6 (CH), 125.5 (CH), 115.9 (CH), 107.8 (CH), 34.8 ($C_q$), 31.4 ($CH_3$), 12.4 ($CH_3$) ppm;

**IR** (ATR): $\tilde{\nu}$ = 3174, 2959, 1598, 1560, 1440, 1292, 1138, 769 $cm^{-1}$;

**HRMS (ESI):** *m/z* $[M+H]^+$ calcd for $C_{17}H_{22}N_3$: 268.1814; found: 268.1815.

**(*E*)-2-(2-(1-(*p*-Tolyl)propylidene)hydrazineyl)pyridine (3e):**

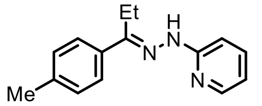

Prepared according to the general procedure on a 10.0 mmol scale, column chromatography (*n*-hexane/ethyl acetate = 2:1) afforded the title compound as a yellow viscous oil; Yield: 90 % (2.15 g, 9.0 mmol).

**$^1$H-NMR** (300 MHz, $CDCl_3$) δ 8.19 (s, 1H), 8.16 – 8.11 (m, 1H), 7.74 – 7.66 (m, 2H), 7.65 – 7.58 (m, 1H), 7.46 – 7.39 (m, 1H), 7.20 (d, $J$ = 8.0 Hz, 2H), 6.81 – 6.74 (m, 1H), 2.73 (q, $J$ = 7.8 Hz, 2H), 2.38 (s, 3H), 1.23 (t, $J$ = 7.7 Hz, 3H) ppm;

**$^{13}$C-NMR** (75 MHz, $CDCl_3$) δ 157.3 ($C_q$), 147.9 ($C_q$), 147.7 (CH), 138.4 ($C_q$), 138.2 (CH), 135.1 ($C_q$), 130.2 (CH), 129.3 (CH), 127.3 (CH), 125.7 (CH), 115.8 (CH), 107.8 (CH), 21.4 ($CH_3$), 19.2 ($CH_2$), 10.4 ($CH_3$) ppm;

**IR** (ATR): $\tilde{\nu}$ = 2972, 1594, 1575, 1503, 1141, 1313, 1138, 770 $cm^{-1}$;

**HRMS (ESI):** *m/z* $[M+H]^+$ calcd for $C_{15}H_{18}N_3$: 240.1501; found: 240.1497.

**(*E*)-2-(2-(1-(4-Methoxyphenyl)propylidene)hydrazineyl)pyridine (3f):**

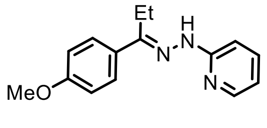

Prepared according to the general procedure on a 10.0 mmol scale, column chromatography (*n*-hexane/ethyl acetate = 2:1) afforded the title compound as a yellow solid; Yield: 95% (2.43 g, 9.5 mmol).

*Note*: The product was isolated as a mixture of *E* and *Z* Stereoisomers. The ratio of *E*/*Z* is 85:15

**$^1$H-NMR** (400 MHz, $CDCl_3$) δ 8.33 (s, 1H), 8.14 – 8.09 (m, 1H), 7.78 – 7.73 (m, 2H), 7.64 – 7.59 (m, 1H), 7.43 – 7.39 (m, 1H), 6.94 – 6.90 (m, 2H), 6.78 – 6.74 (m, 1H), 3.84 (s, 3H), 2.73 (q, *J* = 7.7 Hz, 2H), 1.23 (t, *J* = 7.7 Hz, 3H) ppm;

**$^{13}$C-NMR** (101 MHz, $CDCl_3$) δ 160.1 ($C_q$), 157.2 ($C_q$), 148.2 ($C_q$), 147.1 (CH), 138.5 (CH), 130.5 ($C_q$), 128.9 (CH), 127.2 (CH), 115.6 (CH), 115.0 (CH), 114.0 (CH), 107.9 (CH), 55.5 ($CH_3$), 19.3 ($CH_2$), 10.5 ($CH_3$) ppm;

**IR** (ATR): $\tilde{\nu}$ = 2987, 1670, 1635, 1524, 1437, 1370, 1203, 840 $cm^{-1}$;

**HRMS (ESI):** *m/z* $[M+H]^+$ calcd for $C_{15}H_{18}N_3O$: 256.1450; found: 256.1455.

**(*E*)-2-(2-(Cyclopropyl(phenyl)methylene)hydrazineyl)pyridine (3g):**

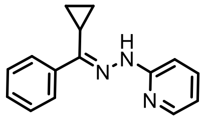

Prepared according to the general procedure on a 10.0 mmol scale, column chromatography (*n*-hexane/ethyl acetate = 2:1) afforded the title compound as a yellow oil; Yield: 76% (1.80 g, 7.6 mmol).

*Note*: The product was isolated as a mixture of *E* and *Z* Stereoisomers. The ratio of *E*/*Z* is 3:1.

**$^1$H-NMR** (400 MHz, $CDCl_3$) δ 8.99 (s, 1H), 8.19 – 8.12 (m, 1H), 7.86 – 7.82 (m, 2H), 7.60 – 7.55 (m, 1H), 7.43 – 7.40 (m, 1H), 7.39 – 7.36 (m, 1H), 7.35 – 7.33 (m, 1H), 7.31 – 7.26 (m, 2H), 6.77 – 6.72 (m, 1H), 1.61 – 1.54 (m, 1H), 1.16 – 1.10 (m, 2H), 0.66 – 0.61 (m, 2H) ppm;

**$^{13}$C-NMR** (101 MHz, $CDCl_3$) δ 156.6 ($C_q$), 147.6 (CH), 145.5 ($C_q$), 138.0 (CH), 137.6 ($C_q$), 129.3 (CH), 127.9 (CH), 127.8 (CH), 127.6 (CH), 126.6 (CH), 115.7 (CH), 107.5 (CH), 7.0 (CH), 6.4 ($CH_2$) ppm;

**IR** (ATR): $\tilde{\nu}$ = 3350, 3004, 1592, 1573, 1488, 1440, 1141, 766 $cm^{-1}$;

**HRMS (ESI):** *m/z* $[M+H]^+$ calcd for $C_{15}H_{16}N_3$: 238.1344; found: 238.1344.

**(*E*)-2-(2-(1-Phenylhexylidene)hydrazineyl)pyridine (3h):**

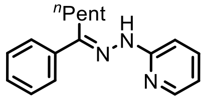

Prepared according to the general procedure on a 10 mmol scale, column chromatography (*n*-hexane/ethyl acetate = 2:1) afforded the title compound as a yellow viscous oil; Yield: 63% (1.79 g, 6.3 mmol).

*Note*: The product was isolated as a mixture of *E* and *Z* Stereoisomers. The ratio of *E*/*Z* is 3:1.

**$^1$H-NMR** (400 MHz, $CDCl_3$) δ 8.43 (s, 1H), 8.16 (dd, *J* = 5.0, 0.9 Hz, 1H), 7.83 – 7.79 (m, 2H), 7.62 – 7.57 (m, 1H), 7.47 – 7.44 (m, 1H), 7.41 – 7.36 (m, 2H), 7.34 – 7.30 (m, 1H), 6.78 – 6.73 (m, 1H), 2.71 – 2.65 (m, 2H), 1.63 – 1.56 (m, 2H), 1.40 – 1.33 (m, 4H), 0.92 – 0.88 (m, 3H) ppm;

**$^{13}$C-NMR** (101 MHz, $CDCl_3$) δ 157.20 ($C_q$), 147.50 (CH), 146.61 ($C_q$), 138.03 ($C_q$), 137.99 (CH), 129.34 (CH), 128.30 (CH), 128.16 (CH), 127.17 (CH), 125.63 (CH), 115.66 (CH), 107.66 (CH), 32.03 ($CH_2$), 25.85 ($CH_2$), 25.43 ($CH_2$), 22.42 ($CH_2$), 13.90 ($CH_3$) ppm;

**IR** (ATR): $\tilde{\nu}$ = 2957, 1595, 1574, 1492, 1440, 1138, 769, 692 $cm^{-1}$;

**HRMS (ESI):** *m/z* $[M+H]^+$ calcd for $C_{17}H_{22}N_3$: 268.1814; found: 268.1815.

**(*E*)-2-(2-(3-methoxy-1-phenylpropylidene)hydrazineyl)pyridine (3i):**

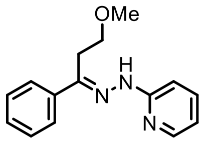


Prepared according to the general procedure on a 5.0 mmol scale, column chromatography (*n*-hexane/ethyl acetate = 2:1) afforded the title compound as a sticky liquid (108 mg, 0.432 mmol, 8%).

**$^1$H-NMR** (300 MHz, $CDCl_3$) δ 8.05 – 7.94 (m, 2H), 7.60 – 7.37 (m, 4H), 7.30 (dd, *J* = 8.2, 1.6 Hz, 3H), 6.69 (ddd, *J* = 7.2, 5.0, 1.1 Hz, 1H), 3.61 (t, *J* = 6.8 Hz, 2H), 3.35 (s, 3H), 2.87 (t, *J* = 6.7 Hz, 2H) ppm.

**$^{13}$C-NMR** (75 MHz, CDCl3) δ 157.0 ($C_q$), 147.7 (CH), 146.8 ($C_q$), 138.1 (CH), 134.1 ($C_q$), 129.7 (CH), 129.3 (CH), 127.5 (CH), 115.4 (CH), 107.4 (CH), 69.6 ($CH_2$), 58.8 ($CH_3$), 38.3 ($CH_2$) ppm.

**IR** (ATR): $\tilde{\nu}$ = 3341, 2923, 2874, 1594, 1527, 1497, 1442, 1115, 1086, 771, 703 $cm^{-1}$;

**HRMS** (ESI): *m/z* $[M+H]^+$ calcd for $C_{15}H_{18}N_3O$: 287.1264; found: 287.1273.

**(*E*)-*N*-(4-(1-(2-(pyridin-2-yl)hydrazineylidene)ethyl)phenyl)acetamide (3j):**

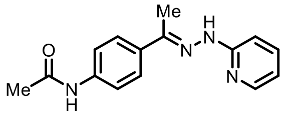


Prepared according to the general procedure on a 10.0 mmol scale, column chromatography (*n*-hexane/ethyl acetate = 1:2) afforded the title compound as a yellow solid; Yield: 53 % (1.42 g, 5.3 mmol).

*Note*: The product was isolated as a mixture of *E* and *Z* Stereoisomers. The ratio of *E*/*Z* is 10:1.

**$^1$H-NMR** (400 MHz, Acetone-$d_6$) δ 9.25 (bs, 1H), 8.70 (bs, 1H), 8.12 – 8.04 (m, 1H), 7.84 – 7.76 (m, 3H), 7.71 – 7.61 (m, 4H), 7.40 – 7.32 (m, 2H), 6.79 – 6.71 (m, 1H), 2.37 (s, 3H), 2.09 (s, 3H) ppm.

**$^{13}$C-NMR** (101 MHz, Acetone-$d_6$) δ 168.9 ($C_q$), 158.9 ($C_q$), 148.7 (CH), 143.8 ($C_q$), 140.6 ($C_q$), 138.6 (CH), 134.9 ($C_q$), 127.0 (CH), 119.6 (CH), 116.2 (CH), 108.0 (CH), 24.4 ($CH_3$), 12.5 ($CH_3$) ppm.

**IR** (ATR): $\tilde{\nu}$ = 3301, 3262, 1669, 1597, 1530, 1443, 1320, 1266, 1141, 839, 772 $cm^{-1}$;

**HRMS (ESI):** *m/z* $[M+H]^+$ calcd for $C_{15}H_{17}N_4O$: 269.1397; found: 269.1401.

## Synthesis of Ligands

### General Procedure 1 (GP1): Synthesis of Enol-Oxazoline Ligands

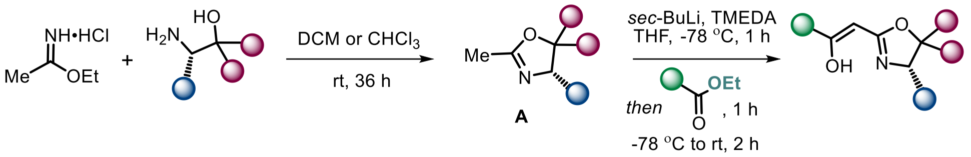


The ligands were synthesized following a modified literature procedure.[2] In an oven-dried 250 mL round-bottom flask charged with a magnetic stirring bar, corresponding (*S*)-amino alcohol (20.0 mmol) was added. Next 70 mL of dichloromethane or chloroform was added to the flask and it was cooled in ice-bath. Next, ethyl acetimidate hydrochloride (22.0 mmol, 1.1 equiv) was added in portions to the solution. Once the addition was completed, the mixture was allowed to warm to room temperature and was continued to stir for 36 hours at room temperature. Then the reaction mixture was quenched with water and the aqueous layer was extracted with dichloromethane (3x30 mL) using a separatory funnel. The extracted organic layer was dried over $Na_2SO_4$, and then evaporated to obtain the intermediate oxazoline (**A**).

In a two-neck flask equipped with a magnetic stirrer bar, crude oxazoline **A** (10.0 mmol), TMEDA (1.2 equiv), and dry THF (30 mL) were added under $N_2$ atmosphere. The mixture was cooled to –78 °C and *sec*-butyllithium (12.0 mmol, 1.2 equiv) was added dropwise for 15 min. The solution was then stirred for 1 hour at –78 °C. Then corresponding ester was added into the reaction mixture and was stirred for another 1 h, followed by slowly warming to the room temperature. After stirring the mixture at room temperature for 2 h, the reaction was quenched by the addition of saturated ammonium chloride solution (10 mL). The reaction mixture was then concentrated under a vacuum to remove the THF and then transferred to a separatory funnel. The aqueous layer was extracted with DCM (3x 20 mL). The combined organic layers were washed with brine (10 mL) and dried over $Na_2SO_4$. The concentrated organic layer was purified through flash column chromatography to obtain the corresponding ligand.

### General Procedure 2 (GP2): Synthesis of Enol-Oxazoline Ligands

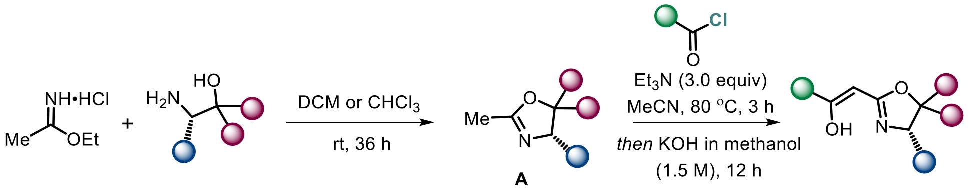


The ligands were synthesized following a modified literature procedure.[3,4] In an oven-dried 250 mL round-bottom flask charged with a magnetic stirring bar, corresponding (*S*)-amino alcohol (20.0 mmol) was added. Next 70 mL of dichloromethane or chloroform was added to the flask and it was cooled in ice-bath. Next, ethyl acetimidate hydrochloride (22.0 mmol, 1.1 equiv) was added in portions to the solution. Once the addition was completed, the mixture was allowed to warm to room temperature and was continued to stir for 36 hours at room temperature. Then the reaction mixture was quenched with water and aqueous layer was extracted with dichloromethane (3x30 mL) using a separatory funnel. The extracted organic layer was dried over $Na_2SO_4$, and then evaporated to obtain the intermediate oxazoline (**A**).

In a two-neck flask equipped with a magnetic stirrer bar, crude oxazoline **A** (10.0 mmol) and dry MeCN (30 mL) were added under $N_2$ atmosphere. The mixture was cooled to 0 °C and triethyl amine

(30.0 mmol, 3.0 equiv) was added dropwise. Next, to the ice-cold solution, corresponding acid chloride (3.0 equiv) was added and the solution was slowly warmed to reflux. After refluxing the mixture at for 3 h, the reaction was quenched by the addition of water. The reaction mixture was then concentrated under a vacuum to remove the MeCN and then transferred to a separatory funnel. The aqueous layer was extracted with DCM (3x 20 mL). The combined organic layers were washed with saturated $NaHCO_3$ solution and then brine (10 mL) and dried over $Na_2SO_4$. The concentrated organic layer was then treated with 1.5 M methanolic KOH and the mixture was stirred at room temperature for 12 hours. Next, the volatile components were removed in a vacuum and the residue was suspended in water (50 mL) and then extracted with DCM (3x20 mL). Finally, the organic layer was dried over $Na_2SO_4$, concentrated in vacuo, and purified through flash column chromatography to obtain the corresponding ligand.

**General Procedure 3 (GP3): Synthesis of Enol-Oxazoline Ligands**

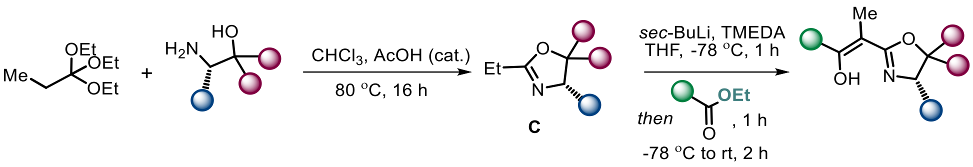


In an oven-dried 100 mL two-neck round-bottom flask charged with a magnetic stirring bar and connected to reflux condenser, corresponding (*S*)-amino alcohol (20.0 mmol) was added. Next 40 mL of chloroform and 100 µL of glacial acetic acid were added to the flask. Next, triethyl orthopropionate (22.0 mmol, 1.1 equiv) was added into the solution along with warming the mixture to 80 °C. The mixture was continued to stir for 16 hours. Then the reaction mixture was concentrated, and quenched with water. Then, the aqueous layer was extracted with dichloromethane (3x30 mL) using a separatory funnel. The extracted organic layer was dried over $Na_2SO_4$, and then evaporated to obtain the intermediate oxazoline (**C**).

In a two-neck flask equipped with a magnetic stirrer bar, crude oxazoline **C** (10.0 mmol), TMEDA (1.2 equiv), and dry THF (30 mL) were added under $N_2$ atmosphere. The mixture was cooled to −78 °C and *sec*-butyllithium (12.0 mmol, 1.2 equiv) was added dropwise for 15 min. The solution was then stirred for 1 hour at −78 °C. Then corresponding ester was added into the reaction mixture and was stirred for another 1 h, followed by slowly warming to room temperature. After stirring the mixture at room temperature for 2 h, the reaction was quenched by the addition of saturated ammonium chloride solution (10 mL). The reaction mixture was then concentrated under a vacuum to remove the THF and then transferred to a separatory funnel. The aqueous layer was extracted with DCM (3x 20 mL). The combined organic layers were washed with brine (10 mL) and dried over $Na_2SO_4$. The concentrated organic layer was purified through flash column chromatography to obtain the corresponding ligand.

**General Procedure 4 (GP4): Synthesis of Enol-Oxazoline Ligand**

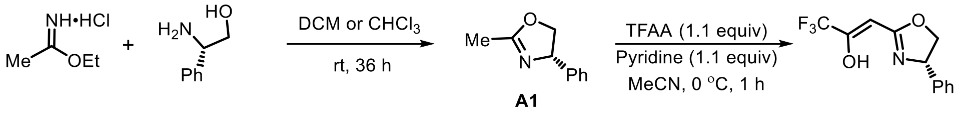


In an oven-dried 250 mL round-bottom flask charged with a magnetic stirring bar, corresponding (*S*)-amino alcohol (20.0 mmol) was added. Next 70 mL of dichloromethane was added to the flask and it was cooled in ice-bath. Next, ethyl acetimidate hydrochloride (22.0 mmol, 1.1 equiv) was added in portions to the solution. Once the addition was completed, the mixture was allowed to warm to room temperature and was continued to stir for 36 hours at room temperature. Then the reaction mixture was quenched with water and aqueous layer was extracted with dichloromethane (3x30 mL) using a

separatory funnel. The extracted organic layer was dried over $Na_2SO_4$, and then evaporated to obtain the intermediate oxazoline (**A1**).

In a two-neck flask equipped with a magnetic stirrer bar, crude oxazoline **A1** (10.0 mmol), Pyridine (1.1 equiv), and dry MeCN (30 mL) were added under $N_2$ atmosphere. The mixture was cooled to 0 °C and trifluoroacetic anhydride (TFAA) (12.0 mmol, 1.1 equiv) was added dropwise for 15 min. The solution was then stirred for 1 hour at 0 °C. Then, the reaction was quenched by the addition of saturated ice-cold $NaHCO_3$ solution (10 mL), concentrated under a vacuum to remove the MeCN and transferred to a separatory funnel. The aqueous layer was extracted with DCM (3x 20 mL). The combined organic layers were washed with brine (10 mL) and dried over $Na_2SO_4$. The concentrated organic layer was purified through flash column chromatography to obtain the corresponding ligand.

**General Procedure 5 (GP5): Synthesis of Amido-Oxazoline Ligands**

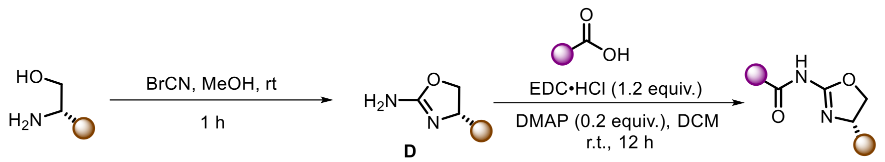


The amine was synthesized following the literature procedure.[5] To a solution of BrCN (11 mmol, 1.1 equiv) in anhydrous methanol (10 mL), corresponding (*S*)-amino alcohol (10.0 mmol, 1.0 equiv) in anhydrous methanol (20 mL) was added slowly at 0 ºC. The solution was allowed to warm slowly to room temperature by stirring for 1 h under $N_2$. Next, a 25% ammonia solution (5 mL) was added and was stirred for 10 min. After that, most of the solvent was evaporated under vacuum. Then, 10 mL of NaOH (20%) was added and the mixture was subsequently stirred vigorously. The mixture was then extracted with ethyl acetate (3x30 mL), the organic layer washed with saturated brine (20 mL), dried over $Na_2SO_4$, and concentrated to obtain oxazoline intermediate **D**. Crude oxazoline **D** (2.0 mmol) was then transferred to a 50 mL round-bottom flask charged with a magnetic stirring bar, then corresponding carboxylic acid (1.2 equiv), DMAP (0.2 equiv) were added under $N_2$ atmosphere followed by the addition of 25 mL of dry DCM. Next, the reaction mixture was cooled to 0 ºC and EDC.HCl (1.2 equiv) was added to the reaction mixture. The solution was slowly warmed to room temperature and was continued to stir overnight. The reaction mixture was quenched with saturated $NaHCO_3$ solution (20 mL) and extracted with DCM (3x20 mL) using a separatory funnel. The combined organic layer was dried over $Na_2SO_4$, concentrated, and purified through column chromatography to obtain the pure oxazoline ligands.

**General Procedure 6 (GP6): Synthesis of Amido-Oxazoline Ligands**

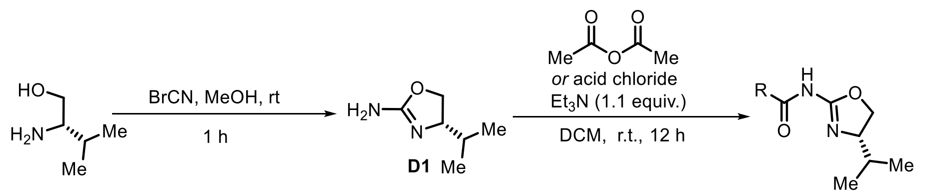


To a solution of BrCN (11 mmol, 1.1 equiv) in anhydrous methanol (10 mL), corresponding (*S*)-amino alcohol (10.0 mmol, 1.0 equiv) in anhydrous methanol (20 mL) was added slowly at 0 ºC. The solution was allowed to warm slowly to room temperature by stirring for 1 h under $N_2$. Next, a 25% ammonia solution (5 mL) was added and was stirred for 10 min. After that, most of the solvent was evaporated under vacuum. Then, 10 mL of NaOH (20%) was added and the mixture was subsequently stirred vigorously. The mixture was then extracted with ethyl acetate (3x30 mL), the organic layer washed with saturated brine (20 mL), dried over $Na_2SO_4$, and concentrated to obtain oxazoline intermediate **D1**.

Crude oxazoline **D1** (2.0 mmol) was then transferred to a 50 mL round-bottom flask charged with a magnetic stirring bar, then 25 mL of dry DCM was added under $N_2$ atmosphere followed by the addition of acetic anhydride ($Ac_2O$) (1.0 equiv) or corresponding acid chloride (1.0 equiv), and $Et_3N$ (1.1 equiv). The solution was continued to stir overnight. The reaction mixture was quenched with saturated $NaHCO_3$ solution (20 mL) and extracted with DCM (3x20 mL) using a separatory funnel. The combined organic layer was dried over $Na_2SO_4$, concentrated, and purified through column chromatography to obtain the pure oxazoline ligands.

## Characterization data of the synthesized ligands

*Tautomers of ligands were observed in the NMR spectra. Data of the major tautomer is mentioned in all the cases.*

**(*S*,*Z*)-1-(4-phenyl-4,5-dihydrooxazol-2-yl)prop-1-en-2-ol (L1):**

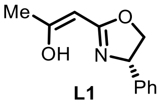


**L1**

Prepared according to procedure **GP1**, isolation by column chromatography (*n*-hexane/ethyl acetate) yielded **L1** (41%, 836 mg) as a grey foam. The product was isolated as a mixture of enol and keto forms in ~ 11:1 ratio.

**$^1$H-NMR** (300 MHz, $CDCl_3$) δ 7.28 – 7.16 (m, 5H), 5.02 – 4.88 (m, 2H), 4.65 – 4.56 (m, 1H), 4.08 (dd, $J$ = 8.6, 6.7 Hz, 1H), 1.97 (s, 3H) ppm.

**$^{13}$C-NMR** (75 MHz, $CDCl_3$) δ 194.5 ($C_q$), 169.1 ($C_q$), 139.2 ($C_q$), 129.1 (CH), 128.7 (CH), 126.1 (CH), 77.0 (CH), 74.6 ($CH_2$), 59.0 (CH), 28.8 ($CH_3$) ppm.

**HRMS (ESI):** *m/z* $[M+H]^+$ calcd for $C_{12}H_{14}NO_2$: 204.1020; found: 204.1019.

**IR** (ATR): $\tilde{\nu}$ = 2958, 1629, 1529, 1248, 1158, 1026, 951, 741, 698 $cm^{-1}$.

**(*S*,*Z*)-1-(4-phenyl-4,5-dihydrooxazol-2-yl)but-1-en-2-ol (L2):**

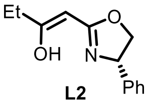


**L2**

Prepared according to procedure **GP1**, isolation by column chromatography (*n*-hexane/ethyl acetate) yielded **L2** (29%, 629 mg) as a red liquid. The product was isolated as a mixture of enol and keto forms in ~ 7:1 ratio.

**$^1$H-NMR** (400 MHz, $CDCl_3$) δ 7.33 – 7.22 (m, 5H), 5.01 – 4.95 (m, 2H), 4.65 – 4.59 (m, 1H), 4.10 (dd, $J$ = 8.6, 6.7 Hz, 1H), 2.28 (q, $J$ = 7.6 Hz, 2H), 1.09 (t, $J$ = 7.6 Hz, 3H) ppm.

**$^{13}$C-NMR** (101 MHz, $CDCl_3$) δ 197.8 ($C_q$), 169.0 ($C_q$), 139.1 ($C_q$), 128.8 (CH), 128.3 (CH), 125.8 (CH), 75.5 (CH), 74.2 ($CH_2$), 58.8 (CH), 34.5 ($CH_2$), 9.9 ($CH_3$) ppm.

**HRMS (ESI):** *m/z* $[M+H]^+$ calcd for $C_{13}H_{16}NO_2$: 218.1184; found: 218.1176.

**IR** (ATR): $\tilde{\nu}$ = 3268, 2971, 1625, 1526, 1492, 1132, 1015, 960, 698 $cm^{-1}$.

**(*S*,*Z*)-3,3-dimethyl-1-(4-phenyl-4,5-dihydrooxazol-2-yl)but-1-en-2-ol (L3):**

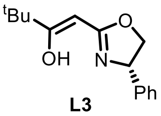


**L3**

Prepared according to procedure **GP2**, isolation by column chromatography (*n*-hexane/ethyl acetate) yielded **L3** (71%, 1.75 g) as a grey solid. The product was isolated as a mixture of enol and keto forms in ~ 7:1 ratio.

**$^1$H-NMR** (400 MHz, $CDCl_3$) δ 7.35 – 7.24 (m, 6H), 5.19 (s, 1H), 5.05 – 4.97 (m, 1H), 4.68 – 4.60 (m, 1H), 4.16 – 4.07 (m, 1H), 1.16 (s, 9H) ppm;

**$^{13}$C-NMR** (101 MHz, $CDCl_3$) δ 202.9 ($C_q$), 169.9 ($C_q$), 139.0 ($C_q$), 128.9 (CH), 128.4 (CH), 126.0 (CH), 74.2 ($CH_2$), 72.5 ($CH_3$), 59.1 (CH), 41.0 ($C_q$), 27.7 ($CH_3$) ppm;

**HRMS** (ESI): *m/z* $[M+H]^+$ calcd for $C_{15}H_{20}NO_2$: 246.1489; found: 246.1489.

**IR** (ATR): $\tilde{\nu}$ = 2962, 1630, 1522, 1491, 1360, 1200, 993, 892, 754 $cm^{-1}$;

**(*S*,*Z*)-1-phenyl-2-(4-phenyl-4,5-dihydrooxazol-2-yl)ethen-1-ol (L4):**

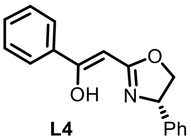


**L4**

Prepared according to procedure **GP2**, isolation by column chromatography (*n*-hexane/ethyl acetate) yielded **L4** (61%, 1.62 g) as a grey foam. The product was isolated as a mixture of enol and keto forms in ~ 11:1 ratio.

**$^1$H-NMR** (400 MHz, $CDCl_3$) δ 7.90 (dd, *J* = 7.9, 1.8 Hz, 2H), 7.44 – 7.32 (m, 9H), 5.73 (s, 1H), 5.16 (dd, *J* = 8.7, 6.8 Hz, 1H), 4.85 – 4.76 (m, 1H), 4.28 (dd, *J* = 8.6, 6.9 Hz, 1H) ppm.

**$^{13}$C-NMR** (101 MHz, $CDCl_3$) δ 187.9 ($C_q$), 170.6 ($C_q$), 139.9 ($C_q$), 139.1 ($C_q$), 130.9 (CH), 129.3 (CH), 129.0 (CH), 128.3 (CH), 127.1 (CH), 126.3 (CH), 75.0 ($CH_2$), 74.3 (CH), 59.4 (CH) ppm.

**HRMS** (ESI): *m/z* $[M+H]^+$ calcd for $C_{17}H_{16}NO_2$: 266.1179; found: 266.1176.

**IR** (ATR): $\tilde{\nu}$ = 3060, 1720, 1628, 1580, 1523, 1267, 1189, 995, 697 $cm^{-1}$.

**(*1Z,3E*)-4-phenyl-1-((*S*)-4-phenyl-4,5-dihydrooxazol-2-yl)buta-1,3-dien-2-ol (L5):**

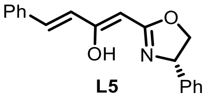

**L5**

Prepared according to procedure **GP1**, isolation by column chromatography (*n*-hexane/ethyl acetate) yielded **L5** (27%, 786 mg) as a grey foam.

**$^{1}$H-NMR** (400 MHz, $CDCl_3$) δ 7.65 – 7.52 (m, 3H), 7.47 – 7.19 (m, 8H), 6.78 (d, *J* = 15.8 Hz, 1H), 5.30 (s, 1H), 5.21 – 5.09 (m, 1H), 4.84 – 4.68 (m, 1H), 4.31 – 4.17 (m, 1H) ppm;

**$^{13}$C-NMR** (101 MHz, $CDCl_3$) δ 184.4 ($C_q$), 170.0 ($C_q$), 139.2 ($C_q$), 137.1 (CH), 135.8 ($C_q$), 129.1 (CH), 129.0 (CH), 128.7 (CH), 128.6 (CH), 128.0 (CH), 127.7 (CH), 126.1 (CH), 78.9 (CH), 74.7 ($CH_2$), 59.6 (CH) ppm;

**HRMS** (ESI): *m/z* $[M+H]^+$ calcd for $C_{19}H_{18}NO_2$: 292.1339; found: 292.1332.

**IR** (ATR): $\tilde{\nu}$ = 3026, 1647, 1517, 1492, 1268, 1011, 998, 693 $cm^{-1}$;

**(*S,Z*)-3,3,3-Trifluoro-1-(4-phenyl-4,5-dihydrooxazol-2-yl)prop-1-en-2-ol (L6):**

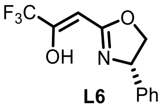

**L6**

Prepared according to procedure **GP4**, isolation by column chromatography (*n*-hexane/ethyl acetate) yielded **L6** (51%, 1.31 g) as a grey foam. The product was isolated as a mixture of enol and keto forms in ~ 7:1 ratio.

**$^{1}$H-NMR** (300 MHz, $CDCl_3$) δ 9.93 (s, 1H), 7.46 – 7.38 (m, 3H), 7.31 – 7.25 (m, 2H), 5.37 (s, 1H), 5.21 (dd, *J* = 9.1, 7.0 Hz, 1H), 4.90 (t, *J* = 9.0 Hz, 1H), 4.38 (dd, *J* = 9.0, 7.0 Hz, 1H) ppm;

**$^{13}$C-NMR** (101 MHz, $CDCl_3$) δ 176.14 (q, J = 33.2 Hz), 171.6 ($C_q$), 138.1 ($C_q$), 129.2 (CH), 129.1 (CH), 126.1 (CH), 117.85 (q, J = 288.4 Hz), 75.7 ($CH_2$), 72.5 (CH), 59.2 (CH) ppm;

**$^{19}$F-NMR** (282 MHz, $CDCl_3$) δ –76.84 ppm;

**HRMS (ESI):** *m/z* $[M+H]^+$ calcd for $C_{12}H_{11}F_3NO_2$: 258.0742; found: 258.0746.

**IR** (ATR): $\tilde{\nu}$ = 3256, 2975, 1628, 1523, 1521, 1381, 1020, 976 $cm^{-1}$.

**(*S*,*Z*)-3-(4-phenyl-4,5-dihydrooxazol-2-yl)but-2-en-2-ol (L7):**

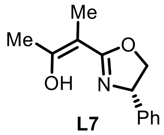

**L7**

Prepared according to procedure **GP3**, isolation by column chromatography (*n*-hexane/ethyl acetate) yielded **L7** (35%, 760 mg) as a red liquid. The product was isolated as a mixture of enol and keto forms in ~ 3:1 ratio.

**$^1$H-NMR** (300 MHz, $CDCl_3$) δ 10.40 (s, 1H), 7.29 – 7.18 (m, 5H), 5.01 (dd, *J* = 8.6, 6.9 Hz, 1H), 4.68 – 4.60 (m, 1H), 4.11 (dd, *J* = 8.6, 6.9 Hz, 1H), 2.07 (s, 3H), 1.81 (s, 3H) ppm.

**$^{13}$C-NMR** (75 MHz, $CDCl_3$) δ 193.9 ($C_q$), 167.7 ($C_q$), 139.8 ($C_q$), 129.2 (CH), 128.6 (CH), 126.3 (CH), 83.6 ($C_q$), 74.6 ($CH_2$), 60.1 (CH), 26.5 ($CH_3$), 11.3 ($CH_3$) ppm.

**HRMS (ESI):** *m/z* $[M+H]^+$ calcd for $C_{13}H_{16}NO_2$: 218.1178; found: 218.1176.

**IR** (ATR): $\tilde{\nu}$ = 2932, 1722, 1631, 1515, 1358, 1233, 1088, 943, 756, 699 $cm^{-1}$.

**(*S*,*Z*)-1-(5,5-Dimethyl-4-phenyl-4,5-dihydrooxazol-2-yl)prop-1-en-2-ol (L8):**

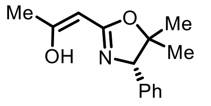

**L8**

Prepared according to procedure **GP1**, isolation by column chromatography (*n*-hexane/ethyl acetate) yielded **L8** (16%, 370 mg) as a grey foam.

**$^1$H-NMR** (400 MHz, $CDCl_3$) δ 7.44 – 7.33 (m, 3H), 7.28 – 7.21 (m, 2H), 5.37 – 4.88 (m, 1H), 4.76 (s, 1H), 2.07 (s, 3H), 1.62 (s, 3H), 0.96 (s, 3H) ppm;

**$^{13}$C-NMR** (101 MHz, $CDCl_3$) δ 168.0 ($C_q$), 136.6 ($C_q$), 128.6 (CH), 128.5 (CH), 128.4 (CH), 126.7 (CH), 126.5 (CH), 87.2 ($C_q$), 67.6 (CH), 28.8 ($CH_3$), 27.5 ($CH_3$), 23.0 ($CH_3$) ppm;

**HRMS (ESI):** *m/z* $[M+H]^+$ calcd for $C_{14}H_{18}NO_2$: 232.1338; found: 232.1339.

**IR** (ATR): $\tilde{\nu}$ = 3249, 2979, 2927, 1634, 1539, 1238, 968, 740 $cm^{-1}$.

**(*S*,*Z*)-1-(4-Isobutyl-4,5-dihydrooxazol-2-yl)prop-1-en-2-ol (L9):**

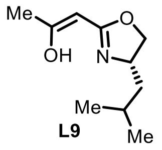

Prepared according to procedure **GP1**, isolation by column chromatography (*n*-hexane/ethyl acetate) yielded **L9** (39%, 714 mg) as a grey foam. The product was isolated as a mixture of enol and keto forms in ~ 15:1 ratio.

**$^1$H-NMR** (400 MHz, $CDCl_3$) δ 9.75 (s, 1H), 4.90 (s, 1H), 4.48 (t, $J$ = 8.1 Hz, 1H), 4.10 – 4.02 (m, 1H), 3.97 (dd, $J$ = 8.4, 7.0 Hz, 1H), 2.03 (s, 3H), 1.73 – 1.64 (m, 1H), 1.62 – 1.55 (m, 1H), 1.44 – 1.36 (m, 1H), 0.96 (d, $J$ = 4.5 Hz, 3H), 0.95 (d, $J$ = 4.5 Hz, 3H) ppm;

**$^{13}$C-NMR** (101 MHz, $CDCl_3$) δ 194.4 ($C_q$), 169.1 ($C_q$), 77.0 (CH), 72.9 ($CH_2$), 54.0 (CH), 43.9 ($CH_2$), 28.9 (CH), 25.7 ($CH_3$), 23.0 ($CH_3$), 22.3 ($CH_3$) ppm;

**HRMS (ESI):** *m/z* $[M+H]^+$ calcd for $C_{10}H_{18}NO_2$: 184.1338; found: 184.1335.

**IR** (ATR): $\tilde{\nu}$ = 3269, 2956, 1645, 1551, 1498, 1461, 1020, 979 $cm^{-1}$.

**(*S*,*Z*)-1-(4-benzyl-4,5-dihydrooxazol-2-yl)prop-1-en-2-ol (L10):**

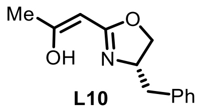

Prepared according to procedure **GP1**, isolation by column chromatography (*n*-hexane/ethyl acetate) yielded **L10** (32%, 694 mg) as a grey foam. The product was isolated as a mixture of enol and keto forms in ~ 5:1 ratio.

**$^1$H-NMR** (300 MHz, $CDCl_3$) δ 7.25 – 7.01 (m, 5H), 4.78 (s, 1H), 4.24 – 4.14 (m, 1H), 4.11 – 3.91 (m, 2H), 2.81 – 2.60 (m, 2H), 1.90 (s, 3H) ppm.

**$^{13}$C-NMR** (75 MHz, $CDCl_3$) δ 193.5 ($C_q$), 168.3 ($C_q$), 135.9 ($C_q$), 128.6 (2xCH), 126.8 (CH), 76.5 (CH), 71.1 ($CH_2$), 56.4 (CH), 40.3 ($CH_2$), 28.4 ($CH_3$) ppm.

**HRMS (ESI):** *m/z* $[M+H]^+$ calcd for $C_{13}H_{16}NO_2$: 218.1182; found: 218.1176.

**IR** (ATR): $\tilde{\nu}$ = 3266, 1632, 1538, 1493, 1454, 1236, 1156, 975, 695 $cm^{-1}$.

**(*S*,*Z*)-1-(4-Methyl-4,5-dihydrooxazol-2-yl)prop-1-en-2-ol (L11):**

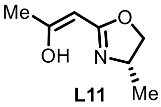

**L11**

Prepared according to procedure **GP1**, isolation by column chromatography (*n*-hexane/ethyl acetate) yielded **L11** (34%, 479 mg) as a grey foam. The product was isolated as a mixture of enol and keto forms in ~ 15:1 ratio.

**$^1$H-NMR** (400 MHz, $CDCl_3$) δ 9.60 (s, 1H), 4.88 (s, 1H), 4.46 (t, *J* = 8.3 Hz, 1H), 4.17 – 4.08 (m, 1H), 3.94 – 3.89 (m, 1H), 2.01 (s, 3H), 1.31 (d, *J* = 6.2 Hz, 3H) ppm;

**$^{13}$C-NMR** (101 MHz, $CDCl_3$) δ 194.6 ($C_q$), 169.0 ($C_q$), 76.9 (CH), 73.7 ($CH_2$), 51.0 (CH), 28.9 ($CH_3$), 20.3 ($CH_3$) ppm;

**HRMS (ESI):** *m/z* $[M+H]^+$ calcd for $C_7H_{12}NO_2$: 142.0868; found: 142.0864.

**IR** (ATR): $\tilde{\nu}$ = 3271, 2972, 1626, 1523, 1467, 1244, 1159, 1021, 976, 946 $cm^{-1}$.

**(*S*,*Z*)-1-(4-isopropyl-4,5-dihydrooxazol-2-yl)prop-1-en-2-ol (L12):**

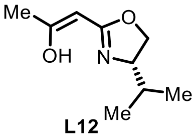

**L12**

Prepared according to procedure **GP1**, isolation by column chromatography (*n*-hexane/ethyl acetate) yielded **L12** (39%, 659 mg) as a red liquid. The product was isolated as a mixture of enol and keto forms in ~ 20:1 ratio.

**$^1$H-NMR** (300 MHz, $CDCl_3$) δ 9.80 (s, 1H), 4.87 (s, 1H), 4.41 (t, *J* = 8.7 Hz, 1H), 4.14 – 4.08 (m, 1H), 3.80 – 3.72 (m, 1H), 2.02 (s, 3H), 1.81 – 1.70 (m, 1H), 0.96 (d, *J* = 6.7 Hz, 3H), 0.90 (d, *J* = 6.8 Hz, 3H) ppm;

**$^{13}$C-NMR** (75 MHz, $CDCl_3$) 194.4 ($C_q$), 169.3 ($C_q$), 76.6 (CH), 70.6 ($CH_2$), 61.4 (CH), 32.5 ($CH_3$), 28.9 (CH), 18.4 ($CH_3$), 18.1 ($CH_3$) ppm;

**HRMS (ESI):** *m/z* $[M+H]^+$ calcd for $C_9H_{16}NO_2$: 170.1179; found: 170.1176.

**IR** (ATR): $\tilde{\nu}$ = 2961, 1630, 1628, 1625, 1253, 1149, 971, 751 $cm^{-1}$.

**(*S*,*Z*)-1-(4-ethyl-4,5-dihydrooxazol-2-yl)prop-1-en-2-ol (L13):**

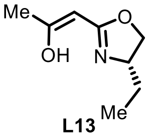


**L13**

Prepared according to procedure **GP1**, isolation by column chromatography (*n*-hexane/ethyl acetate) yielded **L13** (33%, 511 mg) as a red liquid. The product was isolated as a mixture of enol and keto forms in ~ 15:1 ratio.

**$^1$H-NMR** (400 MHz, $CDCl_3$) δ 4.82 (s, 1H), 4.42 – 4.35 (m, 1H), 4.00 – 3.95 (m, 1H), 3.93 – 3.85 (m, 1H), 1.95 (s, 3H), 1.62 – 1.50 (m, 2H), 0.89 (t, *J* = 7.5 Hz, 3H) ppm.

**$^{13}$C-NMR** (101 MHz, $CDCl_3$) δ 194.1 ($C_q$), 169.0 ($C_q$), 76.6 (CH), 71.9 ($CH_2$), 56.6 (CH), 28.7 ($CH_3$), 27.6 ($CH_2$), 9.6 ($CH_3$) ppm.

**HRMS** (ESI): *m/z* $[M+H]^+$ calcd for $C_8H_{14}NO_2$: 156.1018; found: 156.1019.

**IR** (ATR): $\tilde{\nu}$ = 3293, 2966, 1630, 1535, 1495, 1458, 1248, 1159, 954 $cm^{-1}$.

**(*S*)-*N*-(4-isopropyl-4,5-dihydrooxazol-2-yl)-2-phenylacetamide (L14):**

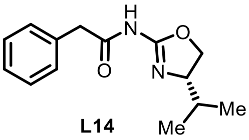


**L14**

Prepared according to procedure **GP5**, isolation by column chromatography (*n*-hexane/ethyl acetate) yielded **L14** (75%, 369 mg) as an off-white foam. The product was isolated as a mixture of tautomer in ~ 9:1 ratio.

**$^1$H-NMR** (300 MHz, $CDCl_3$) δ 9.32 (d, *J* = 7.1 Hz, 1H), 7.27 – 7.19 (m, 4H), 7.18 – 7.09 (m, 1H), 4.34 (dd, 1H), 4.04 (dd, *J* = 9.0, 6.6 Hz, 1H), 3.75 – 3.68 (m, 1H), 3.66 (s, 2H), 1.68 (h, *J* = 6.8 Hz, 1H), 0.85 (d, *J* = 6.7 Hz, 3H), 0.81 (d, *J* = 6.8 Hz, 3H) ppm.

**$^{13}$C-NMR** (75 MHz, $CDCl_3$) δ 185.8 ($C_q$), 166.6 ($C_q$), 136.2 ($C_q$), 129.5 (CH), 128.3 (CH), 126.5 (CH), 68.3 ($CH_2$), 60.8 (CH), 47.3 ($CH_2$), 32.2 (CH), 18.0 ($CH_3$), 17.7 ($CH_3$) ppm.

**HRMS** (ESI): *m/z* $[M+H]^+$ calcd for $C_{14}H_{19}N_2O_2$: 247.1442; found: 247.1441.

**IR** (ATR): $\tilde{\nu}$ = 3304, 2962, 1702, 1632, 1566, 1435, 1348, 1030, 698 $cm^{-1}$.

**(*S*)-*N*-(4-isopropyl-4,5-dihydrooxazol-2-yl)acetamide (L15):**

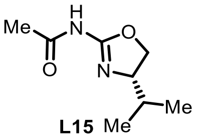

**L15**

Prepared according to procedure **GP6**, isolation by column chromatography (*n*-hexane/ethyl acetate) yielded **L15** (46%, 391 mg) as a colorless liquid.

**$^{1}$H-NMR** (400 MHz, $CDCl_3$) δ 4.45 (dd, *J* = 9.0, 9.0 Hz, 1H), 4.15 (dd, *J* = 9.0, 6.4 Hz, 1H), 3.89 – 3.81 (m, 1H), 2.16 (s, 3H), 1.79 (h, *J* = 6.7 Hz, 1H), 0.97 (d, *J* = 6.7 Hz, 3H), 0.92 (d, *J* = 6.8 Hz, 3H) ppm;

**$^{13}$C-NMR** (101 MHz, $CDCl_3$) δ 175.3 ($C_q$), 166.0 ($C_q$), 68.5 ($CH_2$), 61.2 (CH), 32.4 ($CH_3$), 27.7 (CH), 18.1 ($CH_3$), 17.8 ($CH_3$) ppm.

**HRMS** (ESI): *m/z* $[M+H]^+$ calcd for $C_8H_{15}N_2O_2$: 171.1131; found: 171.1128.

**IR** (ATR): $\tilde{\nu}$ = 3289, 2962, 1690, 1638, 1544, 1368, 1226, 1070, 980, 600 $cm^{-1}$;

**(*S*)-*N*-(4-isopropyl-4,5-dihydrooxazol-2-yl)pivalamide (L16):**

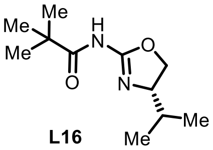

**L16**

Prepared according to procedure **GP5**, isolation by column chromatography (*n*-hexane/ethyl acetate) yielded **L16** (65%, 276 mg) as an off-white foam.

**$^{1}$H-NMR** (400 MHz, $CDCl_3$) δ 9.42 (s, 1H), 4.47 – 4.40 (m, 1H), 4.11 (dd, *J* = 8.9, 6.9 Hz, 1H), 3.81 (d, *J* = 8.9 Hz, 1H), 1.84 – 1.72 (m, 1H), 1.19 (s, 9H), 0.98 (d, *J* = 6.7 Hz, 3H), 0.92 (d, *J* = 6.7 Hz, 3H) ppm.

**$^{13}$C-NMR** (101 MHz, $CDCl_3$) δ 194.2 ($C_q$), 167.2 ($C_q$), 68.2 ($CH_2$), 61.0 (CH), 41.6 ($C_q$), 32.6 (CH), 27.8 ($CH_3$), 18.3 ($CH_3$), 18.0 ($CH_3$).

**HRMS** (ESI): *m/z* $[M+H]^+$ calcd for $C_{11}H_{21}N_2O_2$: 213.1604; found: 213.1598.

**IR** (ATR): $\tilde{\nu}$ = 3317, 2963, 1631, 1563, 1431, 1327, 1197, 996, 751 $cm^{-1}$.

**(*S*)-*N*-(4-isopropyl-4,5-dihydrooxazol-2-yl)-3,4-dimethylbenzamide (L17):**

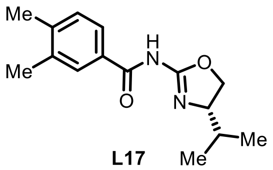


**L17**

Prepared according to procedure **GP5**, isolation by column chromatography (*n*-hexane/ethyl acetate) yielded **L17** (74%, 385 mg) as an off-white foam.

**$^{1}$H-NMR** (400 MHz, $CDCl_3$) δ 9.60 (s, 1H), 7.94 (d, *J* = 1.9 Hz, 1H), 7.90 – 7.84 (m, 1H), 7.07 (d, *J* = 7.9 Hz, 1H), 4.40 – 4.27 (m, 1H), 4.05 (dd, *J* = 9.0, 6.3 Hz, 1H), 3.82 – 3.72 (m, 1H), 2.25 – 2.15 (m, 6H), 1.69 (h, *J* = 6.7 Hz, 1H), 0.85 (d, *J* = 6.7 Hz, 3H), 0.79 (d, *J* = 6.8 Hz, 3H) ppm.

**$^{13}$C-NMR** (101 MHz, $CDCl_3$) δ 178.6 ($C_q$), 166.8 ($C_q$), 140.8 ($C_q$), 135.9 ($C_q$), 134.2 ($C_q$), 130.3 (CH), 129.1 (CH), 126.7 (CH), 67.8 ($CH_2$), 60.4 (CH), 31.9 (CH), 19.6 ($CH_3$), 19.4 ($CH_3$), 17.5 ($CH_3$), 17.3 ($CH_3$) ppm.

**HRMS** (ESI): *m/z* $[M+H]^+$ calcd for $C_{15}H_{21}N_2O_2$: 261.1600; found: 261.1598.

**IR** (ATR): $\tilde{\nu}$ = 2965, 1624, 1549, 1433, 1332, 1260, 1104, 983, 748 $cm^{-1}$.

**(*S*)-*N*-(4-isopropyl-4,5-dihydrooxazol-2-yl)-4-methoxybenzamide (L18):**

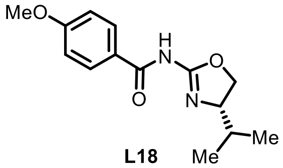


**L18**

Prepared according to procedure **GP5**, isolation by column chromatography (*n*-hexane/ethyl acetate) yielded **L18** (72%, 377 mg) as an off-white foam.

**$^{1}$H-NMR** (300 MHz, $CDCl_3$) δ 9.64 (s, 1H), 8.19 (d, *J* = 8.9 Hz, 2H), 6.90 (d, *J* = 8.9 Hz, 2H), 4.55 – 4.45 (m, 1H), 4.20 (dd, *J* = 9.0, 6.4 Hz, 1H), 3.94 – 3.83 (m, 4H), 1.84 (h, *J* = 6.7 Hz, 1H), 1.04 – 0.91 (m, 6H) ppm.

**$^{13}$C-NMR** (75 MHz, $CDCl_3$) δ 178.6 ($C_q$), 167.2 ($C_q$), 163.0 ($C_q$), 131.6 (CH), 129.6 ($C_q$), 113.4 (CH), 68.3 ($CH_2$), 61.0 (CH), 55.5 ($CH_3$), 32.5 (CH), 18.2 ($CH_3$), 17.9 ($CH_3$) ppm.

**HRMS** (ESI): *m/z* $[M+H]^+$ calcd for $C_{14}H_{19}N_2O_3$: 263.1396; found: 263.1390.

**IR** (ATR): $\tilde{\nu}$ = 3308, 2962, 1630, 1563, 1433, 1348, 1259, 1029, 959, 695 $cm^{-1}$.

**(*S*)-*N*-(4-isopropyl-4,5-dihydrooxazol-2-yl)-3,4,5-trimethoxybenzamide (L19):**

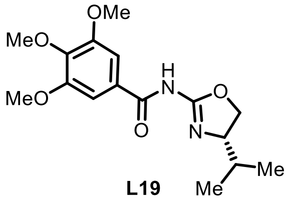


**L19**

Prepared according to procedure **GP5**, isolation by column chromatography (*n*-hexane/ethyl acetate) yielded **L19** (71%, 457 mg) as an off-white foam.

**$^1$H-NMR** (400 MHz, $CDCl_3$) δ 9.65 (s, 1H), 7.52 (s, 2H), 4.56 – 4.49 (m, 1H), 4.22 (dd, *J* = 9.0, 6.4 Hz, 1H), 3.91 (s, 7H), 3.88 (s, 3H), 1.84 (h, *J* = 6.7 Hz, 1H), 1.01 (d, *J* = 6.7 Hz, 3H), 0.96 (d, *J* = 6.7 Hz, 3H) ppm.

**$^{13}$C-NMR** (101 MHz, $CDCl_3$) δ 178.5 ($C_q$), 167.3 ($C_q$), 152.8 ($C_q$), 141.6 ($C_q$), 132.1 ($C_q$), 106.6 (CH), 68.5 ($CH_2$), 61.0 (CH), 56.3 (2x$CH_3$), 32.5 (CH), 18.0 ($CH_3$), 17.9 ($CH_3$) ppm.

**HRMS** (ESI): *m/z* $[M+H]^+$ calcd for $C_{16}H_{23}N_2O_5$: 323.1607; found: 323.1601.

**IR** (ATR): $\tilde{\nu}$ = 2965, 1624, 1559, 1411, 1336, 1218, 1126, 999, 744 $cm^{-1}$.

**(*S*)-3,4,5-trimethoxy-N-(4-phenyl-4,5-dihydrooxazol-2-yl)benzamide (L20):**

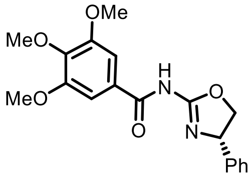


**L20**

Prepared according to procedure **GP5**, isolation by column chromatography (*n*-hexane/ethyl acetate) yielded **L20** (76%, 541 mg) as a colorless liquid.

**$^1$H-NMR** (400 MHz, $CDCl_3$) δ 9.80 (s, 1H), 7.55 (s, 2H), 7.43 – 7.36 (m, 3H), 7.33 – 7.30 (m, 2H), 5.22 (dd, *J* = 9.2, 7.0 Hz, 1H), 4.87 – 4.78 (m, 1H), 4.31 (dd, *J* = 8.8, 7.0 Hz, 1H), 3.93 – 3.88 (m, 9H) ppm.

**$^{13}$C-NMR** (101 MHz, $CDCl_3$) δ 178.5 ($C_q$), 167.3 ($C_q$), 152.8 ($C_q$), 141.7 ($C_q$), 138.5 ($C_q$), 132.0 ($C_q$), 129.5 (CH), 129.3 (CH), 126.2 (CH), 106.7 (CH), 72.5 ($CH_2$), 61.0 (CH), 58.8 ($CH_3$), 56.3 ($CH_3$) ppm.

**HRMS** (ESI): *m/z* $[M+H]^+$ calcd for $C_{19}H_{21}N_2O_5$: 357.1442; found: 357.1445.

**IR** (ATR): $\tilde{\nu}$ = 3011, 1624, 1559, 1456, 1410, 1332, 1219, 1126, 998, 743 $cm^{-1}$.

**(*S*)-4-nitro-N-(4-phenyl-4,5-dihydrooxazol-2-yl)benzamide (L21):**

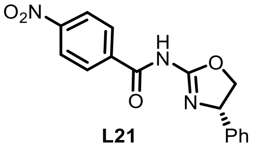


L21

Prepared according to procedure **GP6**, isolation by column chromatography (*n*-hexane/ethyl acetate) yielded **L21** (53%, 330 mg) as a yellow solid.

**$^1$H-NMR** (400 MHz, $CDCl_3$) δ 9.85 (s, 1H), 8.31 (d, *J* = 8.9 Hz, 2H), 8.19 (d, *J* = 8.9 Hz, 2H), 7.44 – 7.37 (m, 3H), 7.34 – 7.29 (m, 2H), 5.26 (dd, *J* = 9.3, 7.1 Hz, 1H), 4.92 – 4.83 (m, 1H), 4.35 (dd, *J* = 9.0, 7.1 Hz, 1H) ppm.

**$^{13}$C-NMR** (101 MHz, $CDCl_3$) δ 176.8 ($C_q$), 167.6 ($C_q$), 150.0 ($C_q$), 142.1 ($C_q$), 138.1 ($C_q$), 130.5 (CH), 129.5 (CH), 126.2 (CH), 123.2 (CH), 72.8 ($CH_2$), 58.9 (CH) ppm. (One carbon peak merged)

**HRMS** (ESI): *m/z* $[M+H]^+$ calcd for $C_{16}H_{14}N_3O_4$: 312.0978; found: 312.0979.

**IR** (ATR): $\tilde{\nu}$ = 3304, 1630, 1602, 1519, 1342, 999, 969, 847, 721 $cm^{-1}$.

**(*S*,*Z*)-*N*-(4-Phenyl-4,5-dihydrooxazol-2-yl)pivalimidic acid (L22):**

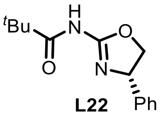


L22

Prepared according to procedure **GP5**, isolation by column chromatography (*n*-hexane/ethyl acetate) yielded **L22** (75%, 369 mg) as an off-white foam.

**$^1$H-NMR** (400 MHz, $CDCl_3$) δ 7.43 – 7.39 (m, 1H), 7.39 – 7.35 (m, 2H), 7.32 – 7.28 (m, 2H), 5.15 (dd, *J* = 9.2, 7.3 Hz, 1H), 4.75 (t, *J* = 8.9 Hz, 1H), 4.22 (dd, *J* = 8.8, 7.5 Hz, 1H), 1.23 (s, 9H) ppm;

**$^{13}$C-NMR** 101 MHz, $CDCl_3$) 194.0 ($C_q$), 167.1 ($C_q$), 138.5 ($C_q$), 129.4 (CH), 129.2 (CH), 126.3 (CH), 72.1 ($CH_2$), 58.9 (CH), 41.7 ($C_q$), 27.8 ($CH_3$) ppm;

**HRMS (ESI):** *m/z* $[M+H]^+$ calcd for $C_{14}H_{19}N_2O_2$: 247.1447; found: 247.1446.

**IR** (ATR): $\tilde{\nu}$ = 3299, 2966, 1706, 1634, 1562, 1424, 1342, 999, 697 $cm^{-1}$.

**(*Z*)-1-((3a*S*,8a*R*)-3a,8a-Dihydro-8*H*-indeno[1,2-*d*]oxazol-2-yl)prop-1-en-2-ol (L23):**

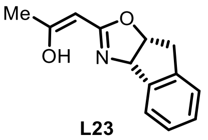

**L23**

Prepared according to procedure **GP1**, isolation by column chromatography (*n*-hexane/ethyl acetate) yielded **L23** (15%, 323 mg) as a grey foam.

**$^{1}$H-NMR** (400 MHz, $CDCl_3$) δ 7.53 (s, 1H), 7.48 (d, *J* = 7.2 Hz, 1H), 7.34 – 7.28 (m, 2H), 7.28 – 7.23 (m, 2H), 6.90 – 6.85 (m, 1H), 3.43 (d, *J* = 2.5 Hz, 2H), 2.24 (s, 3H) ppm;

**$^{13}$C-NMR** (101 MHz, $CDCl_3$) δ 168.8 ($C_q$), 143.0 ($C_q$), 139.8 ($C_q$), 135.5 ($C_q$), 126.1 (CH), 125.6 (2xCH), 124.4 (CH), 116.2 (CH), 115.9 (CH), 36.7 ($CH_2$), 24.3 ($CH_3$) ppm; (one carbon missing)

**IR** (ATR): $\tilde{\nu}$ = 3312, 1653, 1530, 1468, 1370, 1268, 752, 718 $cm^{-1}$;

**HRMS (ESI):** *m/z* $[M+H]^+$ calcd for $C_{14}H_{14}NO_2$: 216.1025; found: 216.1021.

## Optimization of Reaction Conditions

## Screening of Ligands

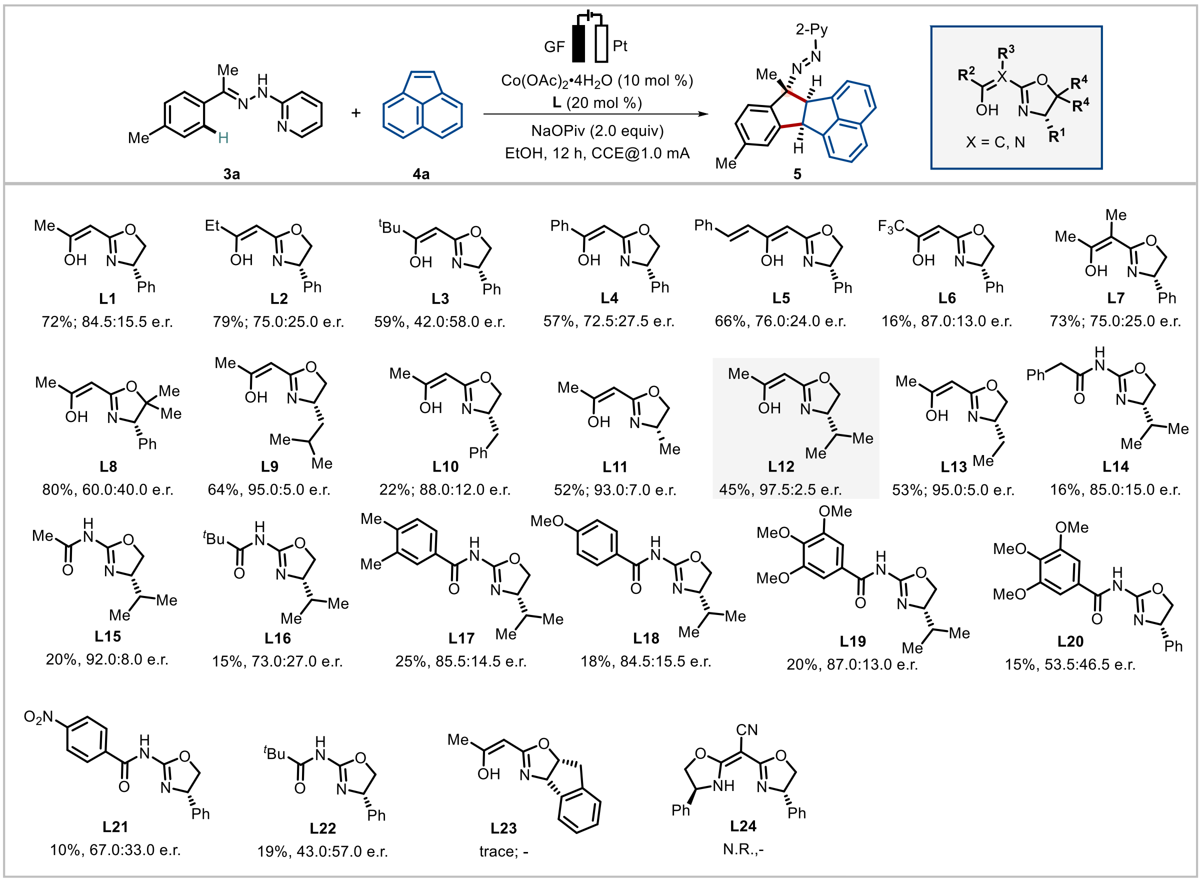


**Figure S2.** Screening of ligands.

The electrolysis was carried out in an undivided cell set up. A GF anode (10 mm × 15 mm × 6 mm) and a platinum cathode (25 mm × 10 mm × 0.125 mm) with electrode holder made of stainless steel were used. The cell was charged with the hydrazone **3a** (0.20 mmol, 1.0 equiv), olefin **4a** (0.40 mmol, 2.0 equiv), and PivONa (0.4 mmol, 2.0 equiv) and a teflon-coated magnetic stirring bar (15 × 6 mm). Then $Co(OAc)_2{\cdot}4H_2O$ (10 mol %) and the ligand **L** (20 mol %) were added into it along with the addition of 5.0 mL of EtOH. The resulting mixture was stirred for 10 minutes to make a homogeneous solution. Afterwards, the electrolysis was performed at room temperature with a constant current of 1.0 mA maintained for 12 h with a stirring rate of 500 rpm. After completion of the reaction, the reaction mixture was diluted with 2 mL ethyl acetate and transferred to a round bottom flask. The electrodes (platinum and graphite felt) were washed in the reaction flask with ethyl acetate (3 × 5 mL) in an ultrasonic cleaner (3 × 3 min) and the washes were combined in the round bottom flask. The yields of the title compound **5** were determined through $^1$H-NMR spectroscopy using 1,3,5-trimethoxybenzene as the internal standard unless otherwise noted.

## Optimization of Reaction Conditions

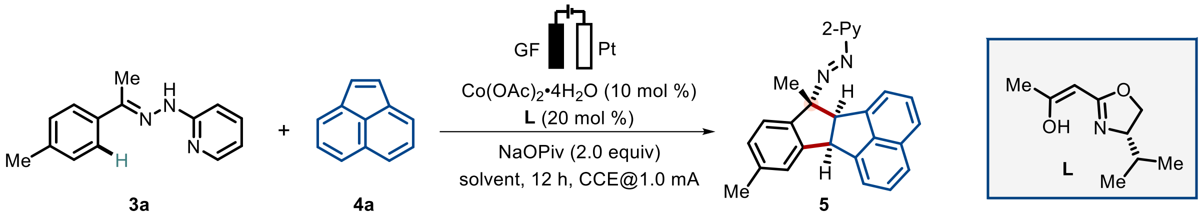


**Table S1.** Screening of solvents.

| Entry | Solvents | Results |
|---|---|---|
| **1** | EtOH:Acetone (1:1) | 58%, 97.5:2.5 e.r. |
| **2** | EtOH:DCE (1:1) | 34%, 96.0:4.0 e.r. |
| **3** | EtOH:Toluene (1:1) | 41%, 95.0:5.0 e.r. |
| **4** | EtOH:$^{i}$PrOH (1:1) | 52%, 97.0:3.0 e.r. |
| **5** | Acetone:$^{i}$PrOH (1:1) | 47%, 97.0:3.0 e.r. |
| **6** | Acetone | 50%, 97.0:3.0 e.r. |

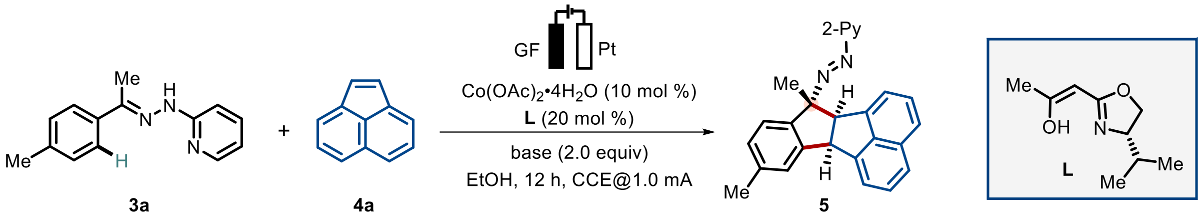


**Table S2.** Further optimization of reaction parameters.

| Entry | Deviation of Reaction Conditions | Results$^{a}$ |
|---|---|---|
| 1 | NaOAc as base | 46%, 96.0:4.0 e.r. |
| 2 | $NaO_2CAd$ as base | complex mixture |
| 3 | no electricity, $O_2$ atmosphere | 38%, 96.0:4.0 e.r.$^{b}$ |
| 4 | no electricity, EtOH:Acetone (1:1) as the solvent, under air | 35%, 97.0:3.0 e.r.$^{b}$ |
| 5 | no electricity, EtOH:Acetone (1:1) as the solvent, $O_2$ balloon | 38%, 97.0:3.0 e.r.$^{b}$ |
| **6** | **EtOH:Acetone (1:1), 24 h** | **80%, 97.5:2.5 e.r.**$^{b,c}$ |
| 7 | no ligand | traces |
| 8 | no $Co(OAc)_2·4H_2O$ | no reaction |

$^{a}$yields were determined by $^{1}$H-NMR with 1,3,5-trimethoxybenzene as the standard; $^{b}$0.3 mmol scale & 4 mL solvent was used; $^{c}$isolated yield

## General Procedure for the Catalytic (3+2)-Annulation Reaction (GP7)

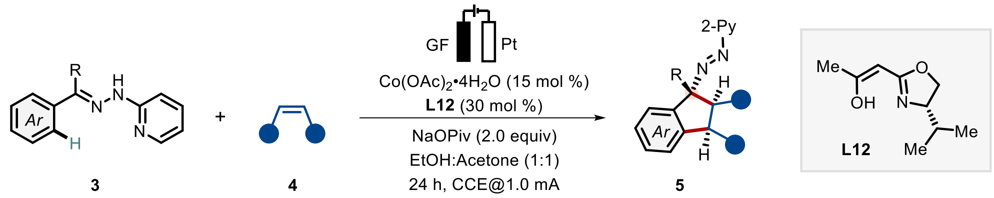


The electrolysis was carried out in an undivided cell set up. A GF anode (10 mm × 15 mm × 6 mm) and a platinum cathode (25 mm × 10 mm × 0.125 mm) with electrode holder made of stainless steel were used. The cell was charged with the hydrazone **3** (0.30 mmol, 1.0 equiv), olefin **4** (0.60 mmol, 2.0 equiv), and PivONa (0.6 mmol, 2.0 equiv) and a teflon-coated magnetic stirring bar (15 × 6 mm). Then $Co(OAc)_2{\cdot}4H_2O$ (15 mol %) and the ligand **L12** (30 mol %) were added into it along with the addition of 2 mL of EtOH and Acetone each. The resulting mixture was stirred for 10 minutes to make a homogeneous solution. Afterwards, the electrolysis was performed at room temperature with a constant current of 1.0 mA maintained for 24 h with a stirring rate of 500 rpm. After completion of the reaction, the reaction mixture was diluted with 2 mL ethyl acetate and transferred to a round bottom flask. The electrodes (platinum and graphite felt) were washed in the reaction flask with ethyl acetate (3 × 5 mL) in an ultrasonic cleaner (3 × 3 min) and the washes were combined in the round bottom flask. The solvent was then removed under vacuum and the residue was purified by column chromatography (ethyl acetate/*n*-hexane) to afford the title compound.

## General Procedure for the Catalytic (3+2)-Annulation Reaction in gram-scale (GP8)

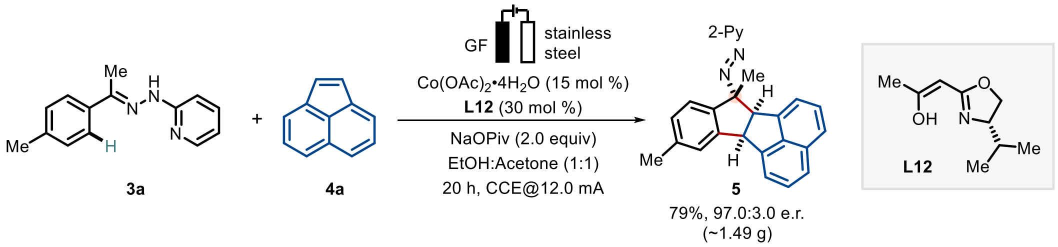


The electrolysis was carried out in an undivided cell set up of 100 mL. A GF anode (2 cm × 6 cm × 6 mm) and a stainless steel cathode (2 cm × 6 cm × 0.125 mm) with electrode holder made of stainless steel were used. The cell was charged with the hydrazone **3a** (5.0 mmol, 1.0 equiv), olefin **4a** (10.0 mmol, 2.0 equiv), and PivONa (10.0 mmol, 2.0 equiv) and a teflon-coated magnetic stirring bar (15 × 6 mm). Then $Co(OAc)_2{\cdot}4H_2O$ (15 mol %) and the ligand **L12** (30 mol %) were added into it along with the addition of 30 mL of EtOH and Acetone each. The resulting mixture was stirred for 10 minutes to make a homogeneous solution. Afterwards, the electrolysis was performed at room temperature with a constant current of 12.0 mA maintained for 20 h with a stirring rate of 500 rpm. After completion of the reaction, the reaction mixture was diluted with 20 mL ethyl acetate and transferred to a round bottom flask. The electrodes (platinum and graphite felt) were washed in the reaction flask with ethyl acetate (3 × 25 mL) in an ultrasonic cleaner (3 × 3 min) and the washes were combined in the round bottom flask. The solvent was then removed under vacuum and the residue was purified by column chromatography (ethylacetate/*n*-hexane) to afford the title compound **5** (yield = 79%, e.r. = 97.0:3.0).

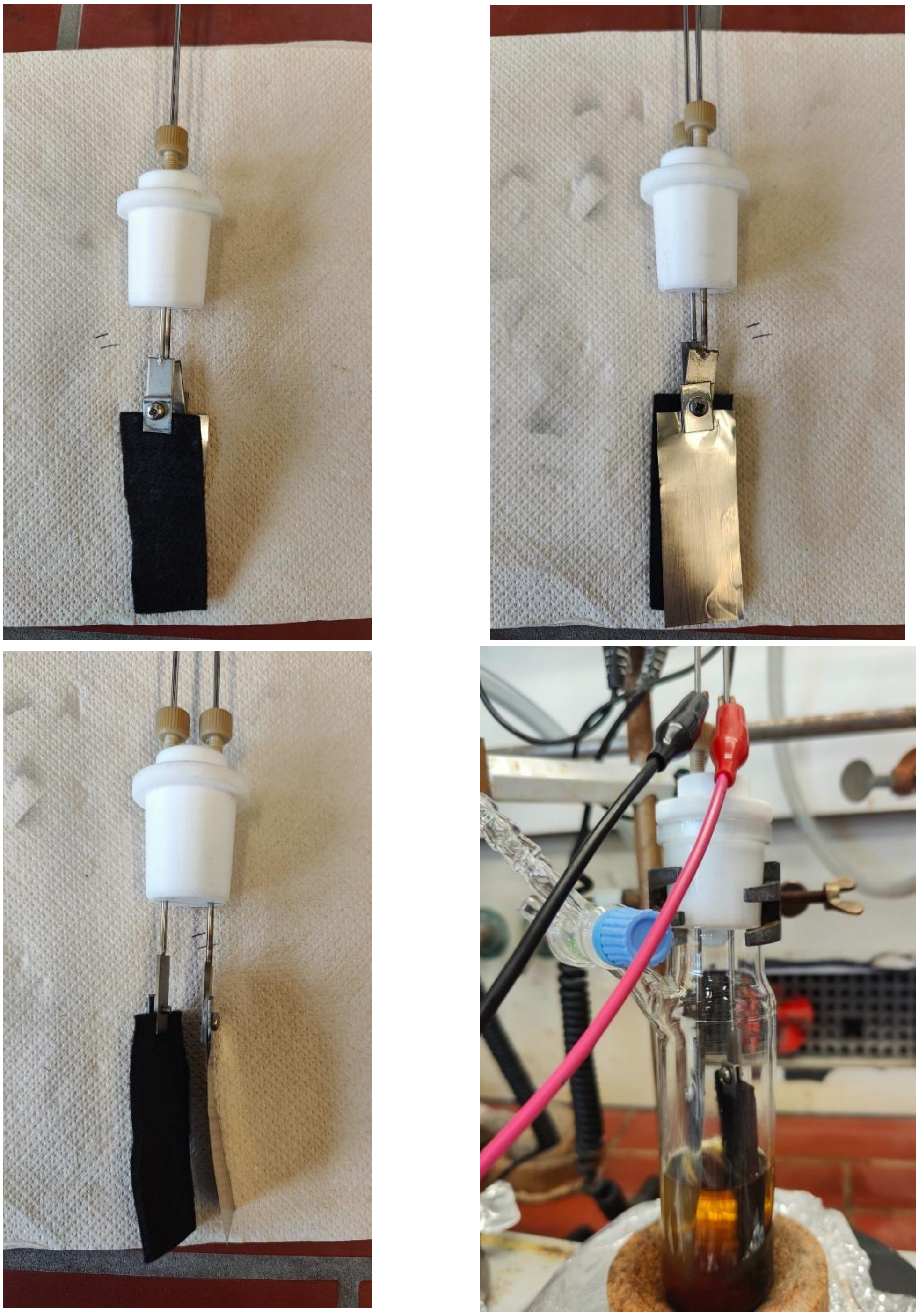

**Figure S3.** Set up for the gram-scale reaction.

## General Procedure for the Catalytic (3+2)-Annulation Reaction using a Solar Panel (GP9)

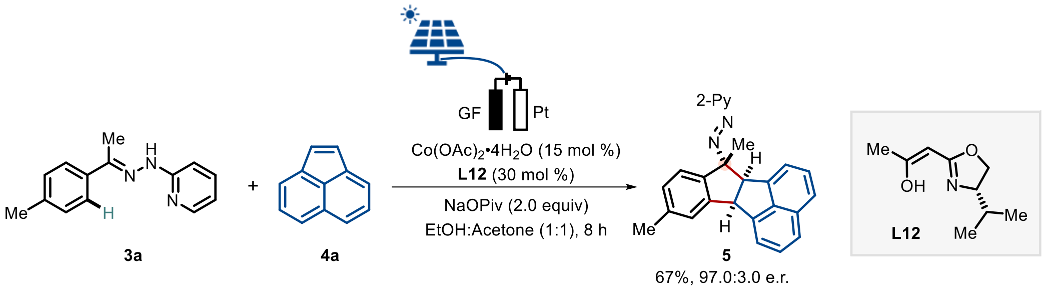


The electrolysis was carried out in an undivided cell set up of 100 mL. A GF anode (10 mm × 15 mm × 6 mm) and a platinum cathode (25 mm × 10 mm × 0.125 mm) with electrode holder made of stainless steel were used. The cell was charged with the hydrazone **3a** (0.4 mmol, 1.0 equiv), olefin **4a** (0.8 mmol, 2.0 equiv), and PivONa (0.8 mmol, 2.0 equiv) and a teflon-coated magnetic stirring bar (15 × 6 mm). Then $Co(OAc)_2{\cdot}4H_2O$ (15 mol %) and the ligand **L12** (30 mol %) were added into it along with the addition of 3.0 mL of EtOH and Acetone each. The resulting mixture was stirred for 10 minutes to make a homogeneous solution. Afterwards, The photovoltaic cell was exposed to natural sunlight and the electrolysis was performed at room temperature for 8 h with a stirring rate of 500 rpm. After completion of the reaction, the reaction mixture was diluted with 3 mL ethyl acetate and transferred to a round bottom flask. The electrodes (platinum and graphite felt) were washed in the reaction flask with ethyl acetate (3 × 5 mL) in an ultrasonic cleaner (3 × 3 min) and the washes were combined in the round bottom flask. The solvent was then removed under vacuum and the residue was purified by column chromatography (ethylacetate/*n*-hexane) to afford the title compound (67% yield, 97.0:3.0 e.r.).

## NMR Data of Synthesized Compounds:

**2-((*E*)-((6*bS*,11*R*,11*aR*)-8,11-dimethyl-6b,11a-dihydro-11H-indeno[1,2-*a*]acenaphthylen-11-yl)diazenyl)pyridine (5):**

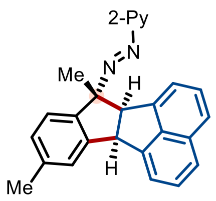


Prepared according to general procedure **GP7** on a 0.3 mmol scale, column chromatography (*n*-hexane/ethyl acetate = 4:1) afforded the title compound as a sticky solid (90 mg, 0.24 mmol, 80%), with an enantiomeric ratio of 97.5:2.5.

**$^1$H-NMR** (400 MHz, $CDCl_3$) δ 8.75 – 8.67 (m, 1H), 7.89 – 7.78 (m, 1H), 7.70 – 7.60 (m, 3H), 7.59 – 7.43 (m, 5H), 7.37 (dd, $J$ = 7.3, 4.9 Hz, 1H), 7.23 (d, $J$ = 7.8 Hz, 1H), 7.07 (d, $J$ = 7.8 Hz, 1H), 5.40 (d, $J$ = 7.3 Hz, 1H), 5.30 (d, $J$ = 7.3 Hz, 1H), 2.37 (s, 3H), 1.70 (s, 3H) ppm.

**$^{13}$C-NMR** (101 MHz, $CDCl_3$) δ 162.8 ($C_q$), 149.4 (CH), 146.4 ($C_q$), 145.0 ($C_q$), 143.8 ($C_q$), 141.7 ($C_q$), 138.9 ($C_q$), 138.5 ($C_q$), 138.5 (CH), 131.8 ($C_q$), 128.8 (CH), 128.0 (CH), 127.9 (CH), 125.1 (3xCH), 123.5 (CH), 123.4 (CH), 122.7 (CH), 119.1 (CH), 114.3 (CH), 85.1 ($C_q$), 56.8 (CH), 54.7 (CH), 23.6 ($CH_3$), 21.6 ($CH_3$) ppm.

**IR** (ATR): $\tilde{\nu}$ = 3048, 2928, 2855, 1684, 1584, 1463, 1427, 1368, 1108, 785 $cm^{-1}$.

**HRMS** (ESI): *m/z* $[M+Na]^+$ calcd for $C_{26}H_{21}N_3Na$: 398.1628; found: 398.1622.

**$[\alpha]^D_{20}$** = +10.0 (c = 1.0, $CHCl_3$).

**$R_t$** (IA column, *n*-hexane/*i*-PrOH 90/10, 1.0 mL/min, 250.4 nm): tr(major) = 9.2 min, tr(minor) = 7.1 min, 97.5:2.5 e.r.

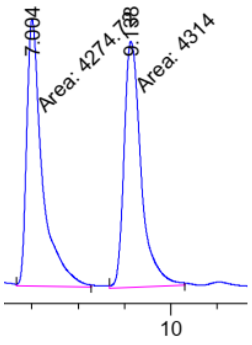


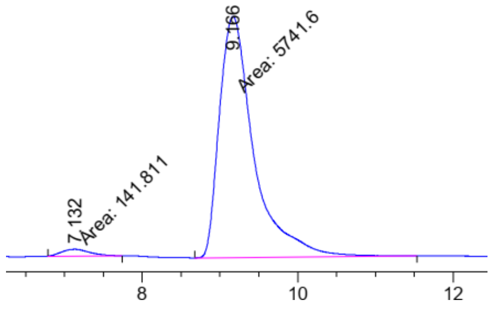


| Peak # | RetTime [min] | Type | Width [min] | Area [mAU*s] | Height [mAU] | Area % |
|---|---|---|---|---|---|---|
| 1 | 7.004 | MM | 0.3940 | 4274.72119 | 180.82039 | 49.7713 |
| 2 | 9.138 | MM | 0.4305 | 4314.00098 | 167.02019 | 50.2287 |

| Peak # | RetTime [min] | Type | Width [min] | Area [mAU*s] | Height [mAU] | Area % |
|---|---|---|---|---|---|---|
| 1 | 7.132 | MM | 0.4361 | 141.81071 | 5.42014 | 2.4103 |
| 2 | 9.166 | MM | 0.5161 | 5741.59814 | 185.40543 | 97.5897 |

**2-((*E*)-((6*bS*,11*R*,11*aR*)-8-chloro-11-methyl-6b,11a-dihydro-11H-indeno[1,2-*a*]acenaphthylen-11-yl)diazenyl)pyridine (6):**

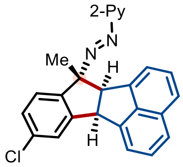


Prepared according to general procedure **GP7** on a 0.3 mmol scale, column chromatography (*n*-hexane/ethyl acetate = 4:1) afforded the title compound as a yellow sticky solid (59 mg, 0.15 mmol, 51%), with an enantiomeric ratio of 94.0:6.0.

**$^{1}$H-NMR** (400 MHz, $CDCl_3$) δ 8.73 – 8.68 (m, 1H), 7.89 – 7.83 (m, 1H), 7.70 – 7.66 (m, 2H), 7.62 – 7.57 (m, 2H), 7.57 – 7.47 (m, 4H), 7.41 – 7.37 (m, 1H), 7.28 – 7.25 (m, 1H), 7.23 – 7.19 (m, 1H), 5.36 (d, $J$ = 8.4 Hz, 1H), 5.29 (d, $J$ = 8.4 Hz, 1H), 1.70 (s, 3H) ppm.

**$^{13}$C-NMR** (101 MHz, $CDCl_3$) δ 162.5 ($C_q$), 149.5 (CH), 146.7 ($C_q$), 145.5 ($C_q$), 143.2 ($C_q$), 143.0 ($C_q$), 138.6 (CH), 138.4 ($C_q$), 134.8 ($C_q$), 131.9 ($C_q$), 128.1 (CH), 128.1 (CH), 128.0 (CH), 126.7 (CH), 125.3 (CH), 124.8 (CH), 123.8 (CH), 123.7 (CH), 122.8 (CH), 119.2 (CH), 114.8 (CH), 84.9 ($C_q$), 56.8 (CH), 54.4 (CH), 23.5 ($CH_3$) ppm.

**IR** (ATR): $\tilde{\nu}$ = 3053, 2929, 1713, 1675, 1592, 1475, 1429, 1216, 1080, 754 $cm^{-1}$.

**HRMS** (ESI): *m/z* $[M+Na]^+$ calcd for $C_{25}H_{18}N_3ClNa$: 418.1078; found: 418.1081.

**$[\alpha]^D_{20}$** = - 32.3 (c = 1.0, $CHCl_3$).

**$R_t$** (IA column, *n*-hexane/*i*-PrOH 90/10, 1.0 mL/min, 250.4 nm): tr(major) = 11.6 min, tr(minor) = 8.3 min, 94.0:6.0 e.r.

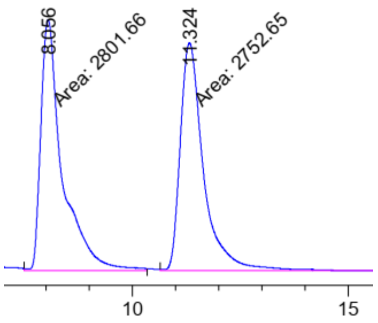


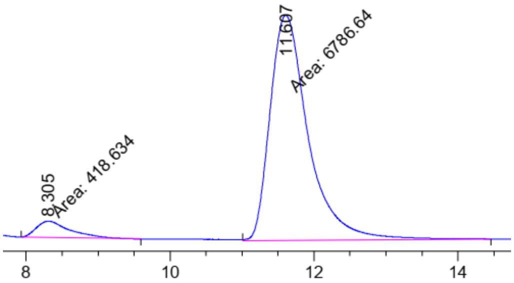


| Peak # | RetTime [min] | Type | Width [min] | Area [mAU*s] | Height [mAU] | Area % |
|---|---|---|---|---|---|---|
| 1 | 8.056 | MM | 0.5738 | 2801.65894 | 81.37717 | 50.4412 |
| 2 | 11.324 | MM | 0.6215 | 2752.64795 | 73.81985 | 49.5588 |

| Peak # | RetTime [min] | Type | Width [min] | Area [mAU*s] | Height [mAU] | Area % |
|---|---|---|---|---|---|---|
| 1 | 8.305 | MM | 0.5277 | 418.63394 | 13.22135 | 5.8101 |
| 2 | 11.607 | MM | 0.6162 | 6786.64014 | 183.57066 | 94.1899 |

**2-((*E*)-((6b*S*,11*R*,11a*R*)-8-bromo-11-methyl-6b,11a-dihydro-11H-indeno[1,2-*a*]acenaphthylen-11-yl)diazenyl)pyridine (7):**

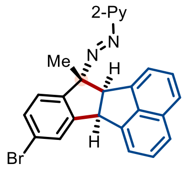


Prepared according to general procedure **GP7** on a 0.3 mmol scale, column chromatography (*n*-hexane/ethyl acetate = 4:1) afforded the title compound as a yellow sticky solid (84 mg, 0.19 mmol, 61%), with an enantiomeric ratio of 94.5:5.5.

**$^1$H-NMR** (400 MHz, $CDCl_3$) δ 8.73 – 8.68 (m, 1H), 7.89 – 7.83 (m, 1H), 7.79 – 7.76 (m, 1H), 7.70 – 7.67 (m, 2H), 7.62 – 7.57 (m, 1H), 7.57 – 7.46 (m, 4H), 7.42 – 7.34 (m, 2H), 7.21 (d, $J$ = 8.2 Hz, 1H), 5.37 (d, $J$ = 7.3 Hz, 1H), 5.28 (d, $J$ = 7.3 Hz, 1H), 1.69 (s, 3H) ppm.

**$^{13}$C-NMR** (101 MHz, $CDCl_3$) δ 162.5 ($C_q$), 149.5 (CH), 147.1 ($C_q$), 145.5 ($C_q$), 143.6 ($C_q$), 143.1 ($C_q$), 138.6 (CH), 138.4 ($C_q$), 131.9 ($C_q$), 130.9 (CH), 128.1 (CH), 128.0 (CH), 127.8 (CH), 127.1 (CH), 125.3 (CH), 123.8 (CH), 123.7 (CH), 123.0 (CH), 122.8 (CH), 119.2 (CH), 114.8 (CH), 84.9 ($C_q$), 56.7 (CH), 54.4 (CH), 23.5 ($CH_3$) ppm.

**IR** (ATR): $\tilde{\nu}$ = 2929, 1713, 1651, 1588, 1467, 1429,1215, 1072, 784, 751 cm$^{-1}$.

**HRMS** (ESI): *m/z* $[M+Na]^+$ calcd for $C_{25}H_{18}N_3BrNa$: 462.0572; found: 462.0576.

**$[\alpha]^D_{20}$** = - 54.3 (c = 1.0, $CHCl_3$).

**$R_t$** (IA column, *n*-hexane/*i*-PrOH 90/10, 1.0 mL/min, 250.4 nm): tr(major) = 11.8 min, tr(minor) = 8.3 min, 94.5:5.5 e.r.

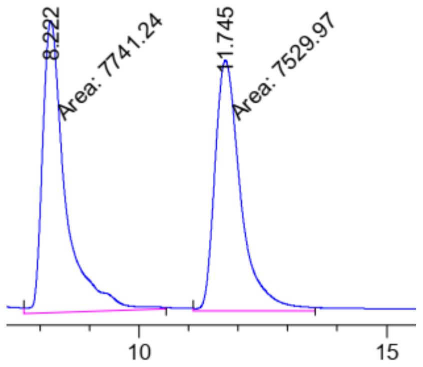


| Peak # | RetTime [min] | Type | Width [min] | Area [mAU*s] | Height [mAU] | Area % |
|---|---|---|---|---|---|---|
| 1 | 8.222 | MM | 0.5388 | 7741.23779 | 239.47740 | 50.6917 |
| 2 | 11.745 | MM | 0.6098 | 7529.96631 | 205.79837 | 49.3083 |

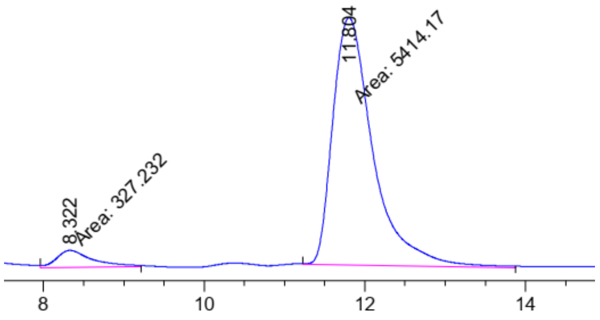


| Peak # | RetTime [min] | Type | Width [min] | Area [mAU*s] | Height [mAU] | Area % |
|---|---|---|---|---|---|---|
| 1 | 8.322 | MM | 0.5156 | 327.23215 | 10.57683 | 5.6995 |
| 2 | 11.804 | MM | 0.5796 | 5414.16748 | 155.67888 | 94.3005 |

**2-((*E*)-((6*bS*,11*R*,11*aR*)-8-iodo-11-methyl-6b,11a-dihydro-11H-indeno[1,2-*a*]acenaphthylen-11-yl)diazenyl)pyridine (8):**

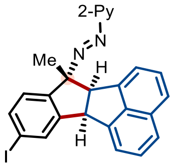


Prepared according to general procedure **GP7** on a 0.3 mmol scale, column chromatography (*n*-hexane/ethyl acetate = 3:1) afforded the title compound as a yellow sticky solid (91 mg, 0.19 mmol, 62%), with an enantiomeric ratio of 92.5:7.5.

**$^1$H-NMR** (400 MHz, $CDCl_3$) δ 8.74 – 8.67 (m, 1H), 8.01 – 7.97 (m, 1H), 7.89 – 7.80 (m, 1H), 7.72 – 7.65 (m, 2H), 7.61 – 7.47 (m, 6H), 7.42 – 7.35 (m, 1H), 7.10 (d, *J* = 8.1 Hz, 1H), 5.36 (d, *J* = 7.3 Hz, 1H), 5.26 (d, *J* = 7.3 Hz, 1H), 1.69 (s, 3H) ppm.

**$^{13}$C-NMR** (101 MHz, $CDCl_3$) δ 162.5 ($C_q$), 149.4 (CH), 147.2 ($C_q$), 145.4 ($C_q$), 144.3 ($C_q$), 143.1 ($C_q$), 138.6 (CH), 138.4 ($C_q$), 136.8 (CH), 133.8 (CH), 131.8 ($C_q$), 128.1 (CH), 128.0 (CH), 127.3 (CH), 125.3 (CH), 123.7 (CH), 123.7 (CH), 122.8 (CH), 119.2 (CH), 114.7 (CH), 94.8 ($C_q$), 85.0 ($C_q$), 56.5 (CH), 54.3 (CH), 23.4 ($CH_3$) ppm.

**IR** (ATR): $\tilde{\nu}$ = 3048, 2974, 1651, 1585, 1465, 1427, 1368, 1215, 1067, 783 $cm^{-1}$.

**HRMS** (ESI): *m/z* $[M+Na]^+$ calcd for $C_{25}H_{18}N_3INa$: 510.0435; found: 510.0438.

**$[\alpha]^D_{20}$** = - 59.5 (c = 1.0, $CHCl_3$).

**$R_t$** (IA column, *n*-hexane/*i*-PrOH 90/10, 1.0 mL/min, 250.4 nm): tr(major) = 11.1 min, tr(minor) = 8.0 min, 92.5:7.5 e.r.

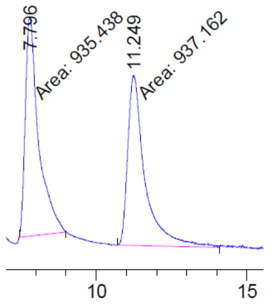


| Peak # | RetTime [min] | Type | Width [min] | Area [mAU*s] | Height [mAU] | Area % |
|---|---|---|---|---|---|---|
| 1 | 7.796 | MM | 0.5106 | 935.43774 | 30.53678 | 49.9540 |
| 2 | 11.249 | MM | 0.6599 | 937.16162 | 23.67062 | 50.0460 |

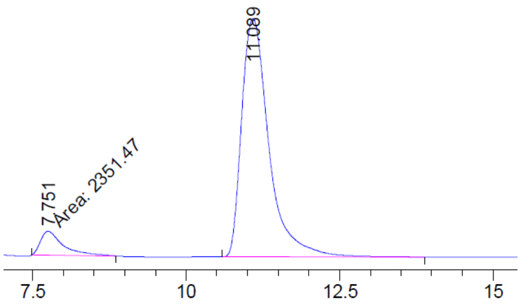


| Peak # | RetTime [min] | Type | Width [min] | Area [mAU*s] | Height [mAU] | Area % |
|---|---|---|---|---|---|---|
| 1 | 7.751 | MM | 0.4331 | 2351.47046 | 90.49634 | 7.7367 |
| 2 | 11.089 | BB | 0.4757 | 2.80422e4 | 906.89264 | 92.2633 |

**2-((*E*)-((6*bS*,11*R*,11*aR*)-8-methoxy-11-methyl-6b,11a-dihydro-11H-indeno[1,2-*a*]acenaphthylen-11-yl)diazenyl)pyridine (9):**

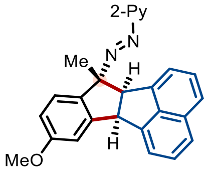


Prepared according to general procedure **GP7** on a 0.3 mmol scale, column chromatography (*n*-hexane/ethyl acetate = 3:1) afforded the title compound as a yellow sticky solid (80 mg, 0.2 mmol, 68%), with an enantiomeric ratio of 98.0:2.0.

**$^1$H-NMR** (400 MHz, $CDCl_3$) δ 8.73 – 8.67 (m, 1H), 7.89 – 7.80 (m, 1H), 7.71 – 7.64 (m, 2H), 7.63 – 7.59 (m, 1H), 7.59 – 7.44 (m, 4H), 7.41 – 7.35 (m, 1H), 7.24 (d, $J$ = 8.5 Hz, 1H), 7.19 – 7.14 (m, 1H), 6.83 – 6.77 (m, 1H), 5.38 (d, $J$ = 7.2 Hz, 1H), 5.31 (d, $J$ = 7.2 Hz, 1H), 3.82 (s, 3H), 1.68 (s, 3H) ppm.

**$^{13}$C-NMR** (101 MHz, $CDCl_3$) δ 162.8 ($C_q$), 160.7 ($C_q$), 149.4 (CH), 146.4 ($C_q$), 146.3 ($C_q$), 143.7 ($C_q$), 138.5 (CH), 136.7 ($C_q$), 131.9 ($C_q$), 128.0 (CH), 127.9 (CH), 126.2 (CH), 125.1 (CH), 123.5 (2xCH), 122.7 (CH), 119.1 (CH), 114.5 (CH), 114.0 (CH), 109.6 (CH), 84.8 ($C_q$), 57.0 (CH), 55.6 ($CH_3$), 54.7 (CH), 23.5 ($CH_3$) ppm. (one $C_q$ peak missing)

**IR** (ATR): $\tilde{\nu}$ = 2926, 1700, 1600, 1493, 1464, 1427, 1283, 1237, 786 $cm^{-1}$.

**HRMS** (ESI): *m/z* $[M+Na]^+$ calcd for $C_{26}H_{21}N_3ONa$: 414.1582; found: 414.1577.

$[\alpha]^D_{20}$ = +8.0 (c = 0.25, $CHCl_3$).

**$R_t$** (IA column, *n*-hexane/*i*-PrOH 90/10, 1.0 mL/min, 250.4 nm): tr(major) = 20.8 min, tr(minor) = 11.2 min, 98.0:2.0 e.r.

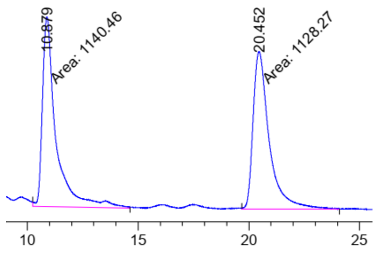


| Peak # | RetTime [min] | Type | Width [min] | Area [mAU*s] | Height [mAU] | Area % |
|---|---|---|---|---|---|---|
| 1 | 10.879 | MM | 0.7112 | 1140.46497 | 26.72468 | 50.2688 |
| 2 | 20.452 | MM | 0.8469 | 1128.27051 | 22.20381 | 49.7312 |

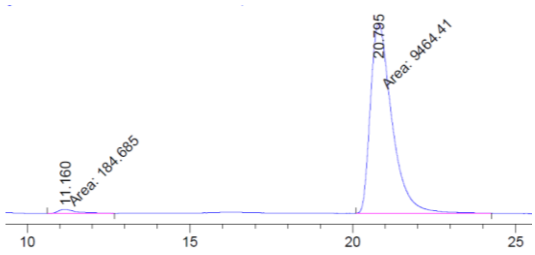


| Peak # | RetTime [min] | Type | Width [min] | Area [mAU*s] | Height [mAU] | Area % |
|---|---|---|---|---|---|---|
| 1 | 11.160 | MM | 0.6811 | 184.68530 | 4.51919 | 1.9140 |
| 2 | 20.795 | MM | 0.7706 | 9464.41211 | 204.69748 | 98.0860 |

**2-((*E*)-((6*bS*,11*R*,11*aR*)-11-methyl-8-(methylthio)-6b,11a-dihydro-11H-indeno[1,2-*a*]acenaphthylen-11-yl)diazenyl)pyridine (10):**

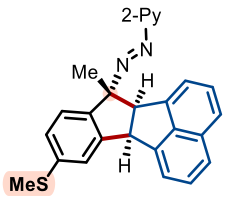


Prepared according to general procedure **GP7** on a 0.3 mmol scale, column chromatography (*n*-hexane/Acetone = 5:1) afforded the title compound as a sticky solid (92 mg, 0.225 mmol, 75%), with an enantiomeric ratio of 97.0:3.0.

**$^{1}$H-NMR** (400 MHz, $CDCl_3$) δ 8.68 (d, *J* = 2.8 Hz, 1H), 7.84 – 7.77 (m, 1H), 7.65 (dd, *J* = 8.0, 3.3 Hz, 2H), 7.59 (d, *J* = 6.9 Hz, 1H), 7.57 – 7.41 (m, 5H), 7.37 – 7.32 (m, 1H), 7.26 – 7.22 (m, 1H), 7.14 (d, *J* = 8.1 Hz, 1H), 5.37 (d, *J* = 7.3 Hz, 1H), 5.29 (d, *J* = 7.3 Hz, 1H), 2.48 (s, 3H), 1.68 (s, 3H) ppm;

**$^{13}$C-NMR** (101 MHz, $CDCl_3$) δ 162.6 ($C_q$), 149.3 (CH), 145.9 ($C_q$), 145.5 ($C_q$), 143.4 ($C_q$), 141.7 ($C_q$), 139.3 ($C_q$), 138.5 (CH), 138.4 ($C_q$), 131.7 ($C_q$), 128.0 (CH), 127.9 (CH), 126.3 (CH), 125.7 (CH), 125.1 (CH), 123.5 (CH), 123.5 (CH), 122.7 (CH), 122.6 (CH), 119.0 (CH), 114.5 (CH), 84.9 ($C_q$), 56.6 (CH), 54.5 ($CH_3$), 23.4 (CH), 16.2 ($CH_3$) ppm;

**IR** (ATR): $\tilde{\nu}$ = 3050, 2920, 1586, 1463, 1426, 1369, 784, 757 $cm^{-1}$;

**HRMS (ESI):** *m/z* $[M+H]^+$ calcd for $C_{26}H_{22}N_3S$: 408.1534; found: 408.1534;

**$[\alpha]^{D}_{20}$** = - 45.24 (c =1.5, $CHCl_3$);

**$R_t$** (IA column, *n*-hexane/*i*-PrOH 90/10, 1.0 mL/min, 250.4 nm): tr(major) = 18.4 min, tr(minor) = 10.2 min, 97.0:3.0 e.r.

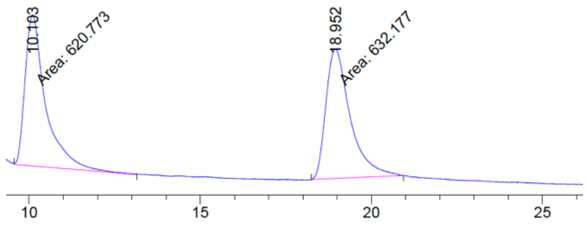


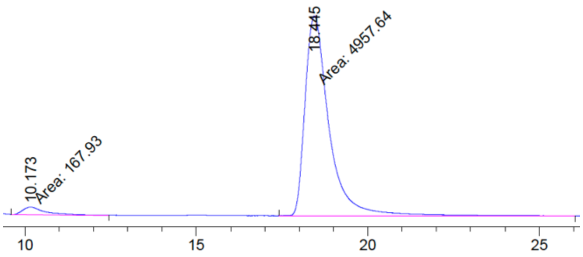


| Peak # | RetTime [min] | Type | Width [min] | Area [mAU*s] | Height [mAU] | Area % |
|---|---|---|---|---|---|---|
| 1 | 10.103 | MM | 0.6940 | 620.77283 | 14.90818 | 49.5449 |
| 2 | 18.952 | MM | 0.8248 | 632.17670 | 12.77454 | 50.4551 |

| Peak # | RetTime [min] | Type | Width [min] | Area [mAU*s] | Height [mAU] | Area % |
|---|---|---|---|---|---|---|
| 1 | 10.173 | MM | 0.7406 | 167.93031 | 3.77919 | 3.2763 |
| 2 | 18.445 | MM | 0.8252 | 4957.64209 | 100.12791 | 96.7237 |

**Methyl (6*bS*,11*R*,11*aR*)-11-methyl-11-((*E*)-pyridin-2-yldiazenyl)-6b,11a-dihydro-11H-indeno[1,2-*a*]acenaphthylene-8-carboxylate (11):**

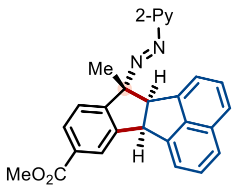


Prepared according to general procedure **GP7** on a 0.3 mmol scale, column chromatography (*n*-hexane/ethyl acetate = 3:1) afforded the title compound as a yellow sticky solid (73 mg, 0.17 mmol, 58%), with an enantiomeric ratio of 84.5:15.5.

**$^{1}$H-NMR** (400 MHz, $CDCl_3$) δ 8.72 – 8.68 (m, 1H), 8.33 (s, 1H), 7.96 – 7.91 (m, 1H), 7.89 – 7.83 (m, 1H), 7.70 – 7.64 (m, 3H), 7.60 – 7.46 (m, 4H), 7.44 – 7.37 (m, 2H), 5.43 (d, J = 7.4 Hz, 1H), 5.32 (d, J = 7.4 Hz, 1H), 3.92 (s, 3H), 1.70 (s, 3H).

**$^{13}$C-NMR** (101 MHz, $CDCl_3$) δ 167.1 ($C_q$), 162.5 ($C_q$), 149.8 ($C_q$), 149.5 (CH), 145.7 ($C_q$), 145.2 ($C_q$), 143.1 ($C_q$), 138.6 (CH), 138.4 ($C_q$), 131.8 ($C_q$), 131.0 ($C_q$), 129.3 (CH), 128.2 (CH), 128.0 (CH), 126.0 (CH), 125.5 (CH), 125.4 (CH), 123.7 (CH), 123.7 (CH), 122.8 (CH), 119.3 (CH), 115.0 (CH), 85.3 ($C_q$), 56.7 (CH), 54.5 (CH), 52.3 ($CH_3$), 23.7 ($CH_3$) ppm.

**IR** (ATR): $\tilde{\nu}$ = 2952, 1720, 1653, 1605, 1435, 1292, 1196, 988, 780 $cm^{-1}$.

**HRMS** (ESI): *m/z* $[M+H]^+$ calcd for $C_{27}H_{22}N_3O_2$: 420.1707; found: 420.1710.

**$[\alpha]^{D}_{20}$** = - 72.1 (c = 0.33, $CHCl_3$).

**$R_t$** (IA column, *n*-hexane/*i*-PrOH 60/40, 1.0 mL/min, 250.4 nm): tr(major) = 13.1 min, tr(minor) = 8.1 min, 84.5:15.5 e.r.

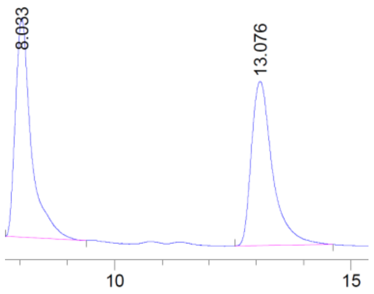


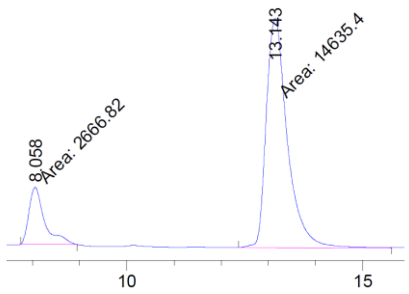


| Peak # | RetTime [min] | Type | Width [min] | Area [mAU*s] | Height [mAU] | Area % |
|---|---|---|---|---|---|---|
| 1 | 8.033 | BB | 0.3413 | 1298.48584 | 55.63282 | 50.0093 |
| 2 | 13.076 | BB | 0.4245 | 1298.00525 | 41.65818 | 49.9907 |

| Peak # | RetTime [min] | Type | Width [min] | Area [mAU*s] | Height [mAU] | Area % |
|---|---|---|---|---|---|---|
| 1 | 8.058 | MM | 0.3824 | 2666.81543 | 116.23814 | 15.4132 |
| 2 | 13.143 | MM | 0.5195 | 1.46354e4 | 469.53076 | 84.5868 |

***N*-((6*bS*,11*R*,11*aR*)-11-methyl-11-((*E*)-pyridin-2-yldiazenyl)-6b,11a-dihydro-11H-indeno[1,2-*a*]acenaphthylen-8-yl)acetamide (12):**

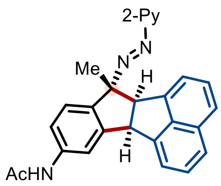


Prepared according to general procedure **GP7** on a 0.3 mmol scale, column chromatography (*n*-hexane/ethyl acetate = 1:1) afforded the title compound as a yellow sticky solid (78 mg, 0.19 mmol, 62%), with an enantiomeric ratio of 98.5:1.5.

**$^1$H-NMR** (400 MHz, $CDCl_3$) δ 8.66 (d, $J$ = 3.6 Hz, 1H), 7.95 (s, 1H), 7.90 – 7.80 (m, 1H), 7.76 (s, 1H), 7.66 – 7.60 (m, 2H), 7.59 – 7.53 (m, 2H), 7.51 – 7.42 (m, 3H), 7.40 – 7.35 (m, 1H), 7.18 (s, 2H), 5.33 (d, $J$ = 7.3 Hz, 1H), 5.26 (d, $J$ = 7.3 Hz, 1H), 2.14 (s, 3H), 1.68 (s, 3H) ppm.

**$^{13}$C-NMR** (101 MHz, $CDCl_3$) δ 168.5 ($C_q$), 162.6 ($C_q$), 149.3 (CH), 146.1 ($C_q$), 145.9 ($C_q$), 143.5 ($C_q$), 140.1 ($C_q$), 138.9 ($C_q$), 138.7 (CH), 138.5 ($C_q$), 131.8 ($C_q$), 128.1 (CH), 127.9 (CH), 125.8 (CH), 125.3 (CH), 123.6 (CH), 123.5 (CH), 122.7 (CH), 119.6 (CH), 119.2 (CH), 115.9 (CH), 114.9 (CH), 85.0 ($C_q$), 56.9 (CH), 54.7 (CH), 24.7 ($CH_3$), 23.3 ($CH_3$) ppm.

**IR** (ATR): $\tilde{\nu}$ = 2925, 1670, 1600, 1541, 1423, 1370, 1324, 1257, 785 $cm^{-1}$.

**HRMS** (ESI): *m/z* $[M+Na]^+$ calcd for $C_{27}H_{22}N_4ONa$: 441.1686; found: 441.1691.

**$[\alpha]^D_{20}$** = - 73.0 (c = 0.5, $CHCl_3$).

**$R_t$** (IA column, *n*-hexane/*i*-PrOH 60/40, 1.0 mL/min, 250.4 nm): tr(major) = 7.7 min, tr(minor) = 5.3 min, 98.5:1.5 e.r.

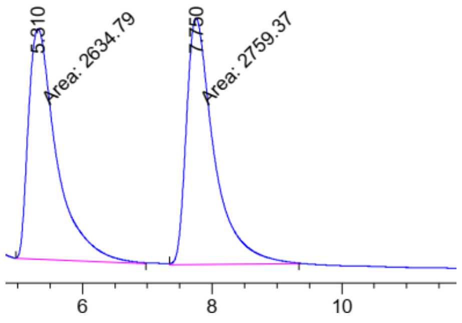


| Peak # | RetTime [min] | Type | Width [min] | Area [mAU*s] | Height [mAU] | Area % |
|---|---|---|---|---|---|---|
| 1 | 5.310 | MM | 0.5056 | 2634.78516 | 86.85712 | 48.8452 |
| 2 | 7.750 | MM | 0.4950 | 2759.37012 | 92.91553 | 51.1548 |

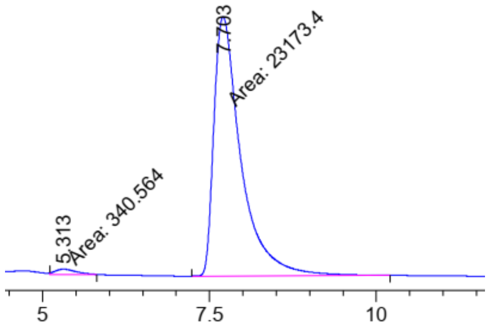


| Peak # | RetTime [min] | Type | Width [min] | Area [mAU*s] | Height [mAU] | Area % |
|---|---|---|---|---|---|---|
| 1 | 5.313 | MM | 0.3515 | 340.56366 | 16.14650 | 1.4483 |
| 2 | 7.703 | MM | 0.4717 | 2.31734e4 | 818.86444 | 98.5517 |

**2-((*E*)-((6*bS*,11*R*,11*aR*)-11-methyl-9-(trifluoromethyl)-6b,11a-dihydro-11H-indeno[1,2-*a*]acenaphthylen-11-yl)diazenyl)pyridine (13):**

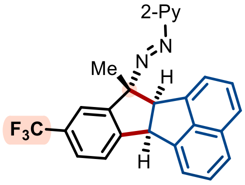


Prepared according to general procedure **GP7** on a 0.3 mmol scale, column chromatography (*n*-hexane/Acetone = 5:1) afforded the title compound as a sticky solid (59 mg, 0.14 mmol, 46%), with an enantiomeric ratio of 92.0:8.0.

**$^1$H-NMR** (400 MHz, $CDCl_3$) δ 8.73 – 8.70 (m, 1H), 7.92 – 7.85 (m, 1H), 7.79 – 7.76 (m, 1H), 7.71 – 7.66 (m, 2H), 7.63 – 7.58 (m, 4H), 7.55 – 7.48 (m, 3H), 7.44 – 7.38 (m, 1H), 5.43 (d, $J$ = 7.4 Hz, 1H), 5.33 (d, $J$ = 7.4 Hz, 1H), 1.71 (s, 3H) ppm.

**$^{13}$C-NMR** (101 MHz, $CDCl_3$) δ 162.4 ($C_q$), 149.6 (CH), 148.8 ($C_q$), 145.6 ($C_q$), 145.3 ($C_q$), 143.0 ($C_q$), 138.7 (CH), 138.4 ($C_q$), 131.9 ($C_q$), 128.1 (CH), 126.2 (q, $J_{CF}$ = 3.9 Hz, CH), 125.5 (CH), 125.1 (CH), 123.9 (CH), 123.8 (CH), 122.9 (CH), 122.6 (q, $J_{CF}$ = 4.0 Hz, CH), 119.2 (CH), 115.4 (CH), 85.1 (CH), 56.5 ($CH_3$), 54.6 (CH), 23.8 (CH) ppm; (three carbon missing)

**$^{19}$F-NMR** (282 MHz, $CDCl_3$) δ -61.99 ppm;

**IR** (ATR): $\tilde{\nu}$ = 1585, 1426, 1334, 1261, 1242, 1167, 1121, 786 $cm^{-1}$;

**HRMS (ESI):** *m/z* $[M+H]^+$ calcd for $C_{26}H_{19}F_3N_3$: 430.1531; found: 430.1525;

**$[\alpha]^D_{20}$** = +6.39 (c = 0.5, $CHCl_3$);

**$R_t$** (OD column, *n*-hexane/*i*-PrOH 95/5, 1.0 mL/min, 250.4 nm): tr(major) = 17.1 min, tr(minor) = 12.8 min, 92.0:8.0 e.r.

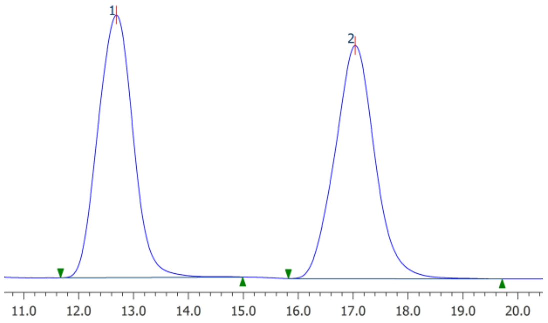


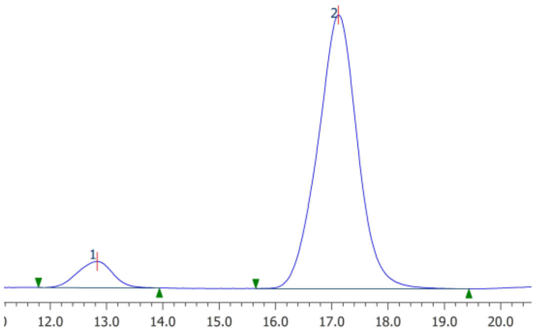


| # | Peak Name | CH | tR [min] | Area [µV·sec] | Height [µV] | Area% | Height% |
|---|---|---|---|---|---|---|---|
| 1 | Unknown | 10 | 12.687 | 4172470 | 93673 | 49.717 | 52.963 |
| 2 | Unknown | 10 | 17.033 | 4220003 | 83193 | 50.283 | 47.037 |

| # | Peak Name | CH | tR [min] | Area [µV·sec] | Height [µV] | Area% | Height% |
|---|---|---|---|---|---|---|---|
| 1 | Unknown | 10 | 12.833 | 285370 | 6568 | 7.823 | 8.778 |
| 2 | Unknown | 10 | 17.117 | 3362354 | 68259 | 92.177 | 91.222 |

**2-((*E*)-((6*bS*,11*R*,11*aR*)-11-methyl-6b,11a-dihydro-11H-indeno[1,2-*a*]acenaphthylen-11-yl)diazenyl)pyridine (14):**

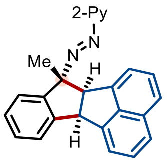


Prepared according to general procedure **GP7** on a 0.3 mmol scale, column chromatography (*n*-hexane/ethyl acetate = 4:1) afforded the title compound as a yellow sticky solid (81 mg, 0.225 mmol, 75%), with an enantiomeric ratio of 96.5:3.5.

**$^1$H-NMR** (400 MHz, $CDCl_3$) δ 8.71 (dd, *J* = 4.7, 1.8 Hz, 1H), 7.83 (td, *J* = 7.7, 1.8 Hz, 1H), 7.71 – 7.59 (m, 4H), 7.60 – 7.44 (m, 4H), 7.41 – 7.29 (m, 3H), 7.30 – 7.21 (m, 1H), 5.45 (d, *J* = 7.3 Hz, 1H), 5.32 (d, *J* = 7.3 Hz, 1H), 1.70 (s, 3H) ppm.

**$^{13}$C-NMR** (101 MHz, $CDCl_3$) δ 162.7 ($C_q$), 149.4 (CH), 146.4 ($C_q$), 144.8 ($C_q$), 144.6 ($C_q$), 143.6 ($C_q$), 138.5 (2C, 1CH, 1$C_q$), 131.8 ($C_q$), 129.0 (CH), 128.0 (CH), 127.9 (CH), 127.8 (CH), 125.4 (CH), 125.1 (CH), 124.6 (CH), 123.5 (CH), 123.5 (CH), 122.7 (CH), 119.1 (CH), 114.5 (CH), 85.4 ($C_q$), 56.5 (CH), 54.8 (CH), 23.6 ($CH_3$) ppm.

**IR** (ATR): $\tilde{\nu}$ = 3046, 2972, 1718, 1653, 1591, 1464, 1429, 1234, 755 $cm^{-1}$.

**HRMS** (ESI): *m/z* $[M+Na]^+$ calcd for $C_{25}H_{19}N_3Na$: 384.1469; found: 384.1471.

**$[\alpha]^D_{20}$** = +18.6 (c = 1.0, $CHCl_3$).

**$R_t$** (IA column, *n*-hexane/*i*-PrOH 90/10, 1.0 mL/min, 250.4 nm): tr(major) = 12.9 min, tr(minor) = 8.2 min, 96.5:3.5 e.r.

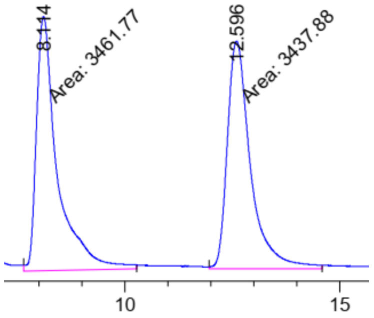


| Peak # | RetTime [min] | Type | Width [min] | Area [mAU*s] | Height [mAU] | Area % |
|---|---|---|---|---|---|---|
| 1 | 8.114 | MM | 0.5645 | 3461.77222 | 102.21111 | 50.1731 |
| 2 | 12.596 | MM | 0.6260 | 3437.88452 | 91.53598 | 49.8269 |

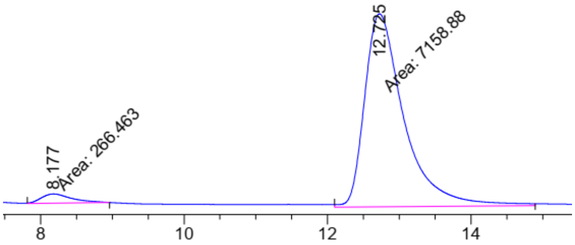


| Peak # | RetTime [min] | Type | Width [min] | Area [mAU*s] | Height [mAU] | Area % |
|---|---|---|---|---|---|---|
| 1 | 8.177 | MM | 0.4937 | 266.46292 | 8.99538 | 3.5886 |
| 2 | 12.725 | MM | 0.6390 | 7158.87598 | 186.70744 | 96.4114 |

**2-((*E*)-((6*bS*,11*R*,11*aR*)-8-(tert-butyl)-11-methyl-6b,11a-dihydro-11H-indeno[1,2-*a*]acenaphthylen-11-yl)diazenyl)pyridine (15):**

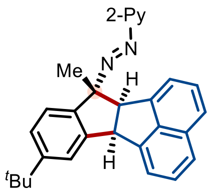


Prepared according to general procedure **GP7** on a 0.3 mmol scale, column chromatography (*n*-hexane/Acetone = 5:1) afforded the title compound as a sticky solid (101 mg, 0.24 mmol, 81%), with an enantiomeric ratio of 95.5:4.5.

**$^{1}$H-NMR** (400 MHz, $CDCl_3$) δ 8.75 – 8.66 (m, 1H), 7.86 – 7.81 (m, 1H), 7.71 – 7.62 (m, 4H), 7.60 – 7.57 (m, 1H), 7.55 – 7.49 (m, 2H), 7.47 – 7.44 (m, 1H), 7.39 – 7.36 (m, 1H), 7.32 – 7.26 (m, 2H), 5.44 (d, $J$ = 7.4 Hz, 1H), 5.33 (d, $J$ = 7.4 Hz, 1H), 1.64 (s, 3H), 1.35 (s, 9H) ppm;

**$^{13}$C-NMR** (101 MHz, $CDCl_3$) δ 162.8 ($C_q$), 152.2 ($C_q$), 149.4 (CH), 146.6 ($C_q$), 144.4 ($C_q$), 143.9 ($C_q$), 141.8 ($C_q$), 138.6 ($C_q$), 138.5 ($C_q$), 131.8 ($C_q$), 128.1 (CH), 127.9 (CH), 125.2 (CH), 125.1 (CH), 124.8 (CH), 123.5 (CH), 123.4 (CH), 122.6 (CH), 121.2 (CH), 119.0 (CH), 114.4 (CH), 85.2 ($C_q$), 56.7 (CH), 55.0 (CH), 34.9 ($C_q$), 31.6 ($CH_3$), 23.8 ($CH_3$) ppm;

**IR** (ATR): $\tilde{\nu}$ = 2963, 1583, 1994, 1463, 1499, 1463, 1426, 784 $cm^{-1}$;

**HRMS (ESI):** *m/z* $[M+H]^+$ calcd for $C_{29}H_{28}N_3$: 418.2283; found: 418.2283;

**$[\alpha]^{D}_{20}$** = +7.75 (c =1.0, $CHCl_3$);

**$R_t$** (OD column, *n*-hexane/*i*-PrOH 95/5, 1.0 mL/min, 250.4 nm): tr(major) = 11.3 min, tr(minor) = 7.8 min, 95.5:4.5 e.r.

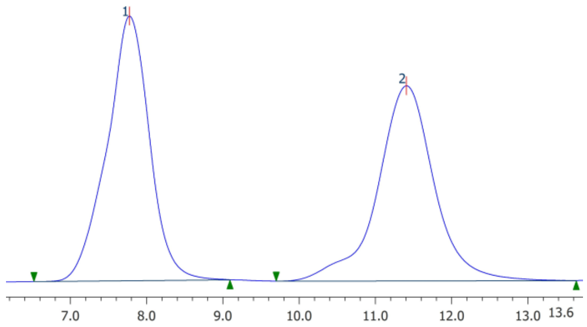


| # | Peak Name | CH | tR [min] | Area [µV·sec] | Height [µV] | Area% | Height% |
|---|---|---|---|---|---|---|---|
| 1 | Unknown | 10 | 7.773 | 3261107 | 83988 | 50.559 | 57.563 |
| 2 | Unknown | 10 | 11.403 | 3188940 | 61918 | 49.441 | 42.437 |

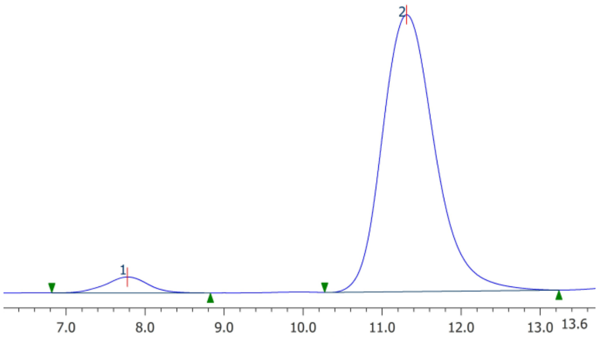


| # | Peak Name | CH | tR [min] | Area [µV·sec] | Height [µV] | Area% | Height% |
|---|---|---|---|---|---|---|---|
| 1 | Unknown | 10 | 7.773 | 532077 | 14111 | 4.480 | 5.430 |
| 2 | Unknown | 10 | 11.307 | 11344759 | 245775 | 95.520 | 94.570 |

**2-((*E*)-((6b*S*,11*R*,11a*R*)-11-methyl-8-phenyl-6b,11a-dihydro-11*H*-indeno[1,2-*a*]acenaphthylen-11-yl)diazenyl)pyridine (16):**

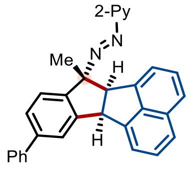


Prepared according to general procedure **GP7** on a 0.3 mmol scale, column chromatography (*n*-hexane/ethyl acetate = 3:1) afforded the title compound as a yellow sticky solid (102 mg, 0.23 mmol, 78%), with an enantiomeric ratio of 92.5:7.5.

**$^1$H-NMR** (400 MHz, $CDCl_3$) δ 8.74 – 8.69 (m, 1H), 7.88 – 7.83 (m, 2H), 7.69 – 7.66 (m, 2H), 7.62 – 7.57 (m, 3H), 7.55 – 7.50 (m, 3H), 7.48 – 7.43 (m, 4H), 7.41 – 7.34 (m, 3H), 5.49 (d, *J* = 7.4 Hz, 1H), 5.36 (d, *J* = 7.4 Hz, 1H), 1.74 (s, 3H) ppm.

**$^{13}$C-NMR** (101 MHz, $CDCl_3$) δ 162.7 ($C_q$), 149.4 (CH), 146.3 ($C_q$), 145.5 ($C_q$), 143.7 ($C_q$), 143.6 ($C_q$), 142.4 ($C_q$), 141.4 ($C_q$), 138.6 (2C, 1CH, 1$C_q$), 131.9 ($C_q$), 128.9 (CH), 128.1 (CH), 127.9 (CH), 127.5 (CH), 127.4 (CH), 127.2 (CH), 125.7 (CH), 125.2 (CH), 123.6 (CH), 123.5 (CH), 123.4 (CH), 122.7 (CH), 119.2 (CH), 114.6 (CH), 85.2 ($C_q$), 56.8 (CH), 54.8 (CH), 23.6 ($CH_3$) ppm.

**IR** (ATR): $\tilde{\nu}$ = 3054, 2931, 1711, 1676, 1601, 1480, 1216, 1075, 754 $cm^{-1}$.

**HRMS** (ESI): *m/z* $[M+Na]^+$ calcd for $C_{31}H_{23}N_3Na$: 460.1789; found: 460.1784.

**$[\alpha]^D_{20}$** = - 92.8 (c = 0.5, $CHCl_3$).

**$R_t$** (IA column, *n*-hexane/*i*-PrOH 95/5, 1.0 mL/min, 250.4 nm): tr(major) = 20.2 min, tr(minor) = 9.7 min, 92.5:7.5 e.r.

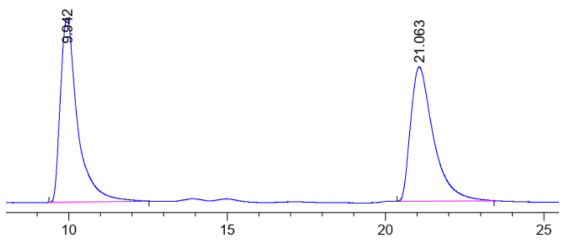


| Peak # | RetTime [min] | Type | Width [min] | Area [mAU*s] | Height [mAU] | Area % |
|---|---|---|---|---|---|---|
| 1 | 9.942 | BB | 0.5404 | 4534.46582 | 120.66723 | 50.8067 |
| 2 | 21.063 | BB | 0.6995 | 4390.46533 | 87.93112 | 49.1933 |

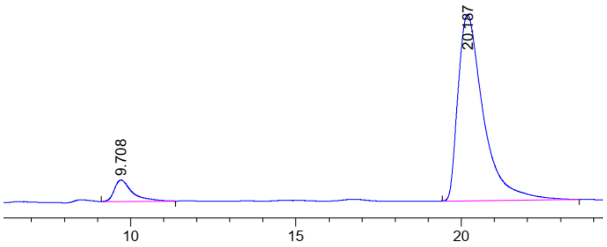


| Peak # | RetTime [min] | Type | Width [min] | Area [mAU*s] | Height [mAU] | Area % |
|---|---|---|---|---|---|---|
| 1 | 9.708 | BB | 0.4775 | 309.89963 | 8.22622 | 7.5172 |
| 2 | 20.187 | BB | 0.7868 | 3812.62085 | 71.64745 | 92.4828 |

**2-((*E*)-((6b*S*,11*R*,11a*R*)-10,11-dimethyl-6b,11a-dihydro-11*H*-indeno[1,2-*a*]acenaphthylen-11-yl)diazenyl)pyridine (17):**

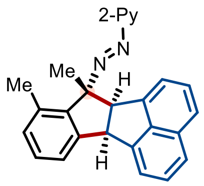


Prepared according to general procedure **GP7** on a 0.3 mmol scale, column chromatography (*n*-hexane/ethyl acetate = 4:1) yielded the title compound **15** as a sticky solid (71 mg, 0.19 mmol, 63%) with an enantiomeric ratio of 97.0:3.0.

**$^{1}$H-NMR** (400 MHz, $CDCl_3$) δ 8.74 (ddd, $J$ = 4.8 Hz, $J$ = 1.9 Hz, $J$ = 0.9 Hz, 1H), 7.90 – 7.85 (m, 1H), 7.69 – 7.63 (m, 2H), 7.63 – 7.61 (m, 1H), 7.61 – 7.59 (m, 1H), 7.59 – 7.56 (m, 1H), 7.53 – 7.51 (m, 1H), 7.50 – 7.48 (m, 1H), 7.44 – 7.40 (m, 2H), 7.32 – 7.27 (m, 1H), 7.06 (d, $J$ = 7.3 Hz, 1H), 5.40 (d, $J$ = 7.9 Hz, 1H), 4.99 (dt, $J$ = 7.9 Hz, $J$ = 1.0 Hz, 1H), 2.30 (s, 3H), 1.46 (s, 3H) ppm;

**$^{13}$C-NMR** (101 MHz, $CDCl_3$) δ 163.0 ($C_q$), 149.5 (CH), 146.0 ($C_q$), 144.5 ($C_q$), 143.8 ($C_q$), 143.3 ($C_q$), 138.8 ($C_q$), 138.6 (CH), 136.2 ($C_q$), 131.7 ($C_q$), 130.6 (CH), 129.1 (CH), 128.0 (CH), 127.9 (CH), 125.2 (CH), 123.7 (CH), 123.4 (CH), 122.8 (CH), 122.6 (CH), 119.2 (CH), 113.8 (CH), 86.7 ($C_q$), 56.5 (CH), 55.0 (CH), 22.5 ($CH_3$), 19.5 ($CH_3$) ppm;

**IR** (ATR): $\tilde{\nu}$ = 3056, 2985, 1651, 1586, 1494, 1464, 1427, 1368, 1260, 1217 $cm^{-1}$;

**HRMS (ESI):** *m/z* $[M+H]^+$ calcd for $C_{26}H_{22}N_3$: 376.1808; found: 376.1808.

**$[\alpha]^{D}_{20}$** = - 60.30 (c = 1.0, $CHCl_3$);

**$R_t$** (IF column, *n*-hexane/*i*-PrOH 95/5, 1.0 mL/min, 250.4 nm): tr(major) = 11.4 min, tr(minor) = 14.6 min, 97:3 e.r.

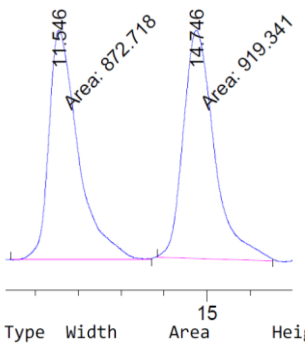


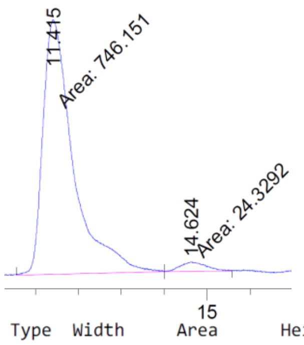


| Peak # | RetTime [min] | Type | Width [min] | Area [mAU*s] | Height [mAU] | Area % |
|---|---|---|---|---|---|---|
| 1 | 11.546 | MM | 0.7940 | 872.71838 | 18.31959 | 48.6992 |
| 2 | 14.746 | MM | 0.8398 | 919.34106 | 18.24631 | 51.3008 |

| Peak # | RetTime [min] | Type | Width [min] | Area [mAU*s] | Height [mAU] | Area % |
|---|---|---|---|---|---|---|
| 1 | 11.415 | MM | 0.8213 | 746.15063 | 15.14155 | 96.8423 |
| 2 | 14.624 | MM | 0.7163 | 24.32916 | 5.66068e-1 | 3.1577 |

**2-((*E*)-((6b*S*,13*R*,13a*R*)-13-methyl-6b,9,10,11,13,13a-hexahydro-8*H*-benzo[5,6]indeno[1,2-*a*]acenaphthylen-13-yl)diazenyl)pyridine (18):**

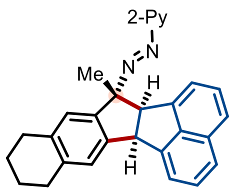


Prepared according to general procedure **GP7** on a 0.3 mmol scale, column chromatography (*n*-hexane/Acetone = 5:1) afforded the title compound as a sticky solid (87 mg, 0.209 mmol, 70%), with an enantiomeric ratio of 93.5:6.5.

**$^1$H-NMR** (400 MHz, $CDCl_3$) δ 8.82 – 8.53 (m, 1H), 7.92 – 7.77 (m, 1H), 7.68 – 7.57 (m, 4H), 7.55 – 7.42 (m, 3H), 7.41 – 7.34 (m, 2H), 7.05 (s, 1H), 5.39 (d, *J* = 7.2 Hz, 1H), 5.28 (d, *J* = 7.3 Hz, 1H), 2.88 – 2.66 (m, 4H), 1.80 – 1.71 (m, 4H), 1.69 (s, 3H) ppm.

**$^{13}$C-NMR** (101 MHz, $CDCl_3$) δ 162.8 ($C_q$), 149.3 (CH), 146.7 ($C_q$), 143.8 ($C_q$), 142.2 ($C_q$), 142.0 ($C_q$), 138.5 ($C_q$), 138.5 (CH), 138.3 ($C_q$), 136.9 ($C_q$), 131.8 ($C_q$), 128.0 (CH), 127.8 (CH), 125.6 (CH), 125.0 (CH), 124.9 (CH), 123.4 (CH), 123.3 (CH), 122.6 (CH), 119.0 (CH), 114.3 (CH), 85.2 ($C_q$), 56.9 (CH), 54.5 (CH), 29.8 ($CH_2$), 29.7 ($CH_2$), 23.5 ($CH_3$), 23.3 ($CH_2$), 23.3 ($CH_2$) ppm.

**IR** (ATR): $\tilde{\nu}$ = 2977, 2930, 2857, 1585, 1489, 1427, 1369, 1216, 827, 784, 666 $cm^{-1}$.

**HRMS** (ESI): *m/z* $[M+Na]^+$ calcd for $C_{29}H_{25}N_3Na$: 438.1941; found: 438.1945.

**$[\alpha]^D_{20}$** = - 24.7 (c = 1.0, $CHCl_3$).

**$R_t$** (IE column, *n*-hexane/*i*-PrOH 95/5, 1.0 mL/min, 250.4 nm): tr(major) = 22.8 min, tr(minor) = 26.8 min, 93.5:6.5 e.r.

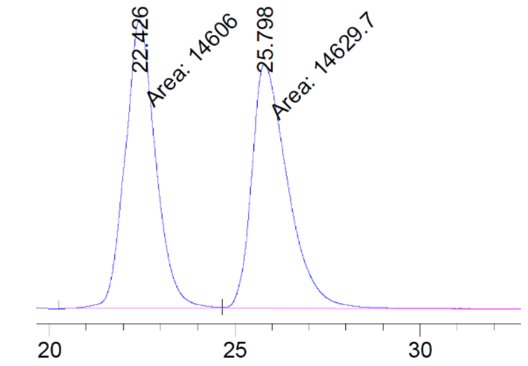


| Peak # | RetTime [min] | Type | Width [min] | Area [mAU*s] | Height [mAU] | Area % |
|---|---|---|---|---|---|---|
| 1 | 22.426 | MF | 0.9575 | 1.46060e4 | 254.23825 | 49.9595 |
| 2 | 25.798 | FM | 1.1307 | 1.46297e4 | 215.64615 | 50.0405 |

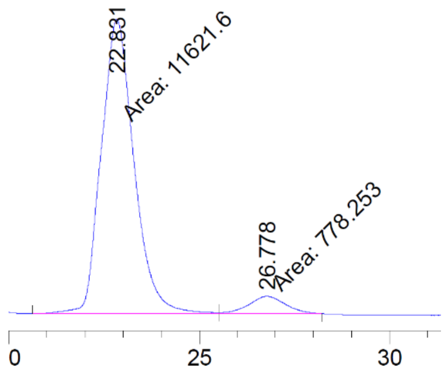


| Peak # | RetTime [min] | Type | Width [min] | Area [mAU*s] | Height [mAU] | Area % |
|---|---|---|---|---|---|---|
| 1 | 22.831 | MF | 0.9968 | 1.16216e4 | 194.32323 | 93.7237 |
| 2 | 26.778 | FM | 1.1294 | 778.25287 | 11.48429 | 6.2763 |

**2-((E)-((6b*S*,13*R*,13a*R*)-13-methyl-6b,13a-dihydro-13*H*-benzo[5,6]indeno[1,2-*a*]acenaphthylen-13-yl)diazenyl)pyridine (19):**

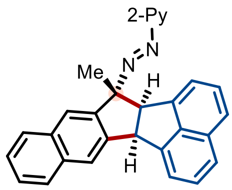


Prepared according to general procedure **GP7** on a 0.3 mmol scale, column chromatography (*n*-hexane/Acetone = 5:1) afforded the title compound as a sticky solid (92 mg, 0.224 mmol, 75%), with an enantiomeric ratio of 90.0:10.0.

**$^1$H-NMR** (400 MHz, $CDCl_3$) δ 8.73 – 8.69 (m, 1H), 8.10 (s, 1H), 7.88 – 7.34 (m, 14H), 5.59 (d, $J$ = 7.5 Hz, 1H), 5.38 (d, $J$ = 7.5 Hz, 1H), 1.85 (s, 3H) ppm.

**$^{13}$C-NMR** (101 MHz, $CDCl_3$) δ 162.7 ($C_q$), 149.4 (CH), 146.5 ($C_q$), 144.0 ($C_q$), 143.5 ($C_q$), 143.4 ($C_q$), 138.6 (CH), 134.3 ($C_q$), 133.5 ($C_q$), 131.8 ($C_q$), 128.3 (CH), 128.1 (CH), 127.9 (CH), 127.8 (CH), 126.0 (CH), 125.4 (CH), 125.2 (CH), 124.4 (CH), 123.6 (CH), 123.5 (CH), 123.1 (CH), 122.7 (CH), 119.2 (CH), 114.6 (CH), 85.0 ($C_q$), 57.1 (CH), 54.3 (CH), 23.7 ($CH_3$) ppm. One signal ($C_q$) is not observed.

**IR** (ATR): $\tilde{\nu}$ = 3054, 2978, 1600, 1586, 1500, 1463, 1427, 861, 785, 750 $cm^{-1}$.

**HRMS** (ESI): *m/z* $[M+Na]^+$ calcd for $C_{29}H_{21}N_3Na$: 434.1628; found: 434.1628.

**$[\alpha]^D_{20}$** = - 191.2 (c = 1.0, $CHCl_3$).

**$R_t$** (IF column, *n*-hexane/*i*-PrOH 80/20, 1.0 mL/min, 250.4 nm): tr(major) = 9.3 min, tr(minor) = 10.4 min, 90:10 e.r.

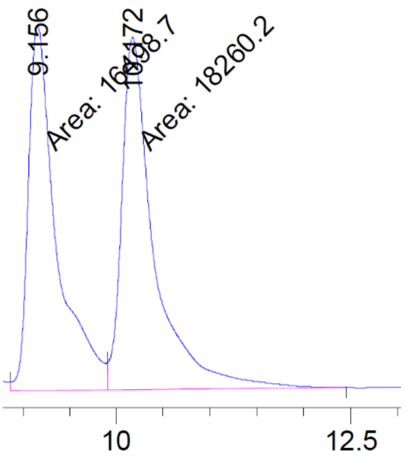


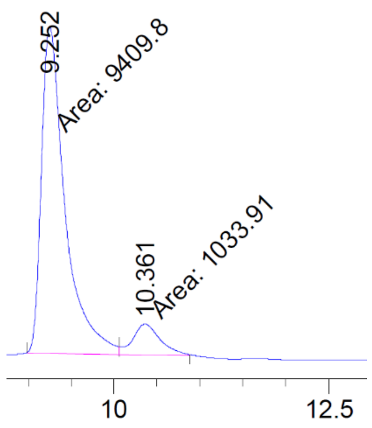


| Peak # | RetTime [min] | Type | Width [min] | Area [mAU*s] | Height [mAU] | Area % |
|---|---|---|---|---|---|---|
| 1 | 9.156 | MF | 0.3511 | 1.64987e4 | 783.14740 | 47.4660 |
| 2 | 10.172 | FM | 0.4032 | 1.82602e4 | 754.84491 | 52.5340 |

| Peak # | RetTime [min] | Type | Width [min] | Area [mAU*s] | Height [mAU] | Area % |
|---|---|---|---|---|---|---|
| 1 | 9.252 | MF | 0.3191 | 9409.79688 | 491.42789 | 90.1001 |
| 2 | 10.361 | FM | 0.3715 | 1033.91284 | 46.38000 | 9.8999 |

**2-((*E*)-((6b*S*,11*R*,11a*R*)-11-propyl-6b,11a-dihydro-11*H*-indeno[1,2-*a*]acenaphthylen-11-yl)diazenyl)pyridine (20):**

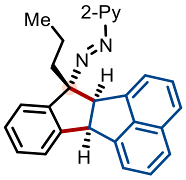


Prepared according to general procedure **GP7** on a 0.3 mmol scale, column chromatography (*n*-hexane/ethyl acetate = 3:1) afforded the title compound **19** as a yellow sticky solid (99 mg, 0.26 mmol, 85%), with an enantiomeric ratio of 93.0:7.0.

**$^{1}$H-NMR** (400 MHz, $CDCl_3$) δ 8.75 – 8.69 (m, 1H), 7.90 – 7.82 (m, 1H), 7.67 – 7.50 (m, 9H), 7.42 – 7.27 (m, 3H), 5.39 (d, $J$ = 7.5 Hz, 1H), 5.31 (d, $J$ = 7.5 Hz, 1H), 2.28 – 2.13 (m, 2H), 0.97 – 0.81 (m, 1H), 0.59 (t, $J$ = 7.2 Hz, 3H), 0.46 – 0.30 (m, 1H) ppm.

**$^{13}$C-NMR** (101 MHz, $CDCl_3$) δ 162.8 ($C_q$), 149.4 (CH), 146.1 ($C_q$), 144.8 ($C_q$), 143.8 ($C_q$), 143.6 ($C_q$), 138.9 ($C_q$), 138.5 (CH), 131.7 ($C_q$), 128.9 (2xCH), 128.0 (CH), 127.5 (CH), 126.2 (CH), 125.1 (CH), 124.7 (CH), 123.6 (CH), 123.4 (CH), 122.0 (CH), 119.1 (CH), 114.1 (CH), 88.9 ($C_q$), 55.2 (CH), 54.7 (CH), 38.4 ($CH_2$), 18.0 ($CH_2$), 14.3 ($CH_3$) ppm.

**IR** (ATR): $\tilde{\nu}$ = 2961, 1712, 1652, 1591, 1464, 1430, 1227, 1107, 756 $cm^{-1}$.

**HRMS** (ESI): *m/z* $[M+H]^+$ calcd for $C_{27}H_{24}N_3$: 390.1965; found: 390.1964.

**$[\alpha]^{D}_{20}$** = - 0.6 (c = 0.5, $CHCl_3$).

**$R_t$** (IA column, *n*-hexane/*i*-PrOH 90/10, 1.0 mL/min, 250.4 nm): tr(major) = 12.7 min, tr(minor) = 6.7 min, 93.0:7.0 e.r.

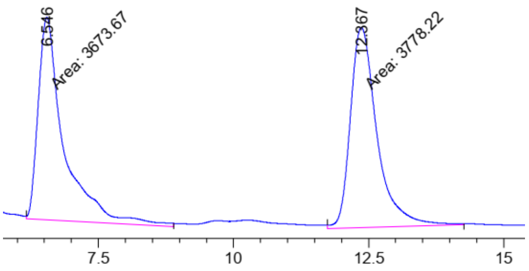


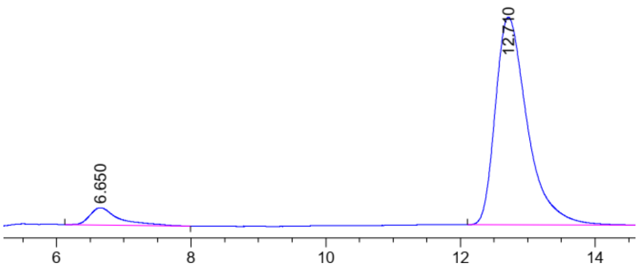


| Peak # | RetTime [min] | Type | Width [min] | Area [mAU*s] | Height [mAU] | Area % |
|---|---|---|---|---|---|---|
| 1 | 6.546 | MM | 0.5495 | 3673.66895 | 111.42335 | 49.2985 |
| 2 | 12.367 | MM | 0.5726 | 3778.22192 | 109.98173 | 50.7015 |

| Peak # | RetTime [min] | Type | Width [min] | Area [mAU*s] | Height [mAU] | Area % |
|---|---|---|---|---|---|---|
| 1 | 6.650 | BB | 0.4172 | 811.81915 | 27.27768 | 6.9810 |
| 2 | 12.710 | BB | 0.5027 | 1.08171e4 | 328.04758 | 93.0190 |

**2-((*E*)-((6b*S*,11*R*,11a*R*)-11-ethyl-8-methyl-6b,11a-dihydro-11*H*-indeno[1,2-*a*]acenaphthylen-11-yl)diazenyl)pyridine (21):**

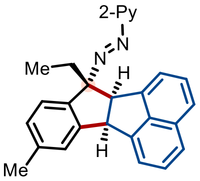


Prepared according to general procedure **GP7** on a 0.3 mmol scale, column chromatography (*n*-hexane/ethyl acetate = 5:1) afford ed the title compound as a sticky solid (71 mg, 0.183 mmol, 61%), with an enantiomeric ratio of 94.0:6.0.

**$^1$H-NMR** (400 MHz, $CDCl_3$) δ 8.70 (d, $J$ = 3.2 Hz, 1H), 7.86 – 7.81 (m, 1H), 7.68 – 7.64 (m, 2H), 7.61 (d, $J$ = 6.9 Hz, 1H), 7.57 – 7.49 (m, 4H), 7.46 (s, 1H), 7.41 (d, $J$ = 7.8 Hz, 1H), 7.37 (dd, $J$ = 7.5, 4.7 Hz, 1H), 7.11 (d, $J$ = 6.9 Hz, 1H), 5.38 (d, $J$ = 7.5 Hz, 1H), 5.28 (d, $J$ = 7.5 Hz, 1H), 2.39 (s, 3H), 2.35 – 2.28 (m, 1H), 2.23 – 2.14 (m, 1H), 0.38 (t, $J$ = 7.4 Hz, 3H) ppm;

**$^{13}$C-NMR** (101 MHz, $CDCl_3$) δ 162.8 ($C_q$), 149.3 (CH), 146.1 ($C_q$), 144.9 ($C_q$), 143.8 ($C_q$), 140.5 ($C_q$), 139.0 ($C_q$), 138.7 ($C_q$), 138.5 (CH), 131.7 ($C_q$), 128.5 (CH), 128.0 (2xCH), 126.0 (CH), 125.3 (CH), 125.0 (CH), 123.5 (CH), 123.4 (CH), 122.1 (CH), 119.0 (CH), 114.0 (CH), 88.8 ($C_q$), 55.3 (CH), 54.5 ($CH_3$), 29.1 ($CH_2$), 21.6 (CH), 9.3 ($CH_3$) ppm;

**IR** (ATR): $\tilde{\nu}$ = 2964, 2928, 1584, 1464, 1426, 1215, 1044, 783, 753 $cm^{-1}$;

**HRMS (ESI):** *m/z* $[M+H]^+$ calcd for $C_{27}H_{24}N_3$: 390.1970; found: 390.1965;

**$[\alpha]^D_{20}$** = - 20.49 (c = 1.0, $CHCl_3$);

**$R_t$** (IA column, *n*-hexane/*i*-PrOH 90/10, 1.0 mL/min, 250.4 nm): tr(major) = 9.1 min, tr(minor) = 6.5 min, 94.0:6.0 e.r.

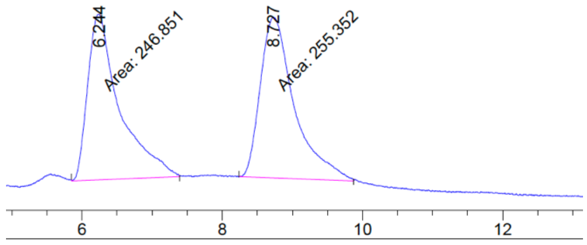


| Peak # | RetTime [min] | Type | Width [min] | Area [mAU*s] | Height [mAU] | Area % |
|---|---|---|---|---|---|---|
| 1 | 6.244 | MM | 0.5285 | 246.85143 | 7.78443 | 49.1537 |
| 2 | 8.727 | MM | 0.5633 | 255.35182 | 7.55530 | 50.8463 |

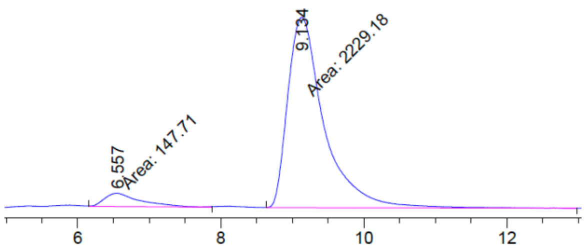


| Peak # | RetTime [min] | Type | Width [min] | Area [mAU*s] | Height [mAU] | Area % |
|---|---|---|---|---|---|---|
| 1 | 6.557 | MM | 0.5670 | 147.70979 | 4.34162 | 6.2144 |
| 2 | 9.134 | MM | 0.5892 | 2229.18262 | 63.05751 | 93.7856 |

**2-((E)-((6bS,11R,11aR)-11-ethyl-8-methoxy-6b,11a-dihydro-11H-indeno[1,2-a]acenaphthylen-11-yl)diazenyl)pyridine (22):**

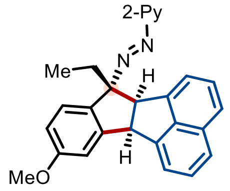


Prepared according to general procedure **GP7** on a 0.3 mmol scale, column chromatography (*n*-hexane/ethyl acetate = 4:1) afforded the title compound as a sticky solid (96 mg, 0.24 mmol, 79%), with an enantiomeric ratio of 98.0:2.0.

**$^{1}$H-NMR** (400 MHz, $CDCl_3$) δ 8.70 (dd, $J$ = 4.7, 1.0 Hz, 1H), 7.86 – 7.81 (m, 1H), 7.69 – 7.63 (m, 2H), 7.60 – 7.49 (m, 5H), 7.43 (d, $J$ = 8.5 Hz, 1H), 7.39 – 7.35 (m, 1H), 7.16 (d, $J$ = 2.6 Hz, 1H), 6.85 (dd, $J$ = 8.4, 2.6 Hz, 1H), 5.40 (d, $J$ = 7.5 Hz, 1H), 5.27 (d, $J$ = 7.5 Hz, 1H), 3.83 (s, 3H), 2.35 – 2.25 (m, 1H), 2.24 – 2.15 (m, 1H), 0.39 (t, $J$ = 7.4 Hz, 3H) ppm;

**$^{13}$C-NMR** (101 MHz, $CDCl_3$) δ 162.8 ($C_q$), 160.5 ($C_q$), 149.4 (CH), 146.4 ($C_q$), 145.9 ($C_q$), 143.7 ($C_q$), 138.9 ($C_q$), 138.5 (CH), 135.5 ($C_q$), 131.7 ($C_q$), 128.0 (2xCH), 127.0 (CH), 125.0 (CH), 123.5 (CH), 123.4 (CH), 122.1 (CH), 119.0 (CH), 114.0 (CH), 113.7 (CH), 109.7 (CH), 88.5 ($C_q$), 55.5 (CH), 55.5 (CH), 54.5 ($CH_3$), 29.0 ($CH_2$), 9.3 ($CH_3$) ppm; (two carbon merged)

**IR** (ATR): $\tilde{\nu}$ = 3030, 1644, 1635, 1511, 1432, 1371, 1214, 770 $cm^{-1}$;

**HRMS (ESI):** *m/z* $[M+H]^+$ calcd for $C_{27}H_{24}N_3O$: 406.1919; found: 406.1916;

**$[\alpha]^D_{20}$** = - 15.63 (c =0.2, $CHCl_3$);

**$R_t$** (IA column, *n*-hexane/*i*-PrOH 90/10, 1.0 mL/min, 250.4 nm): tr(major) = 23.2 min, tr(minor) = 8.8 min, 98.0:2.0 e.r.

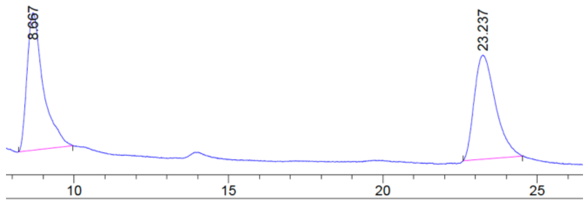


| Peak # | RetTime [min] | Type | Width [min] | Area [mAU*s] | Height [mAU] | Area % |
|---|---|---|---|---|---|---|
| 1 | 8.667 | BB | 0.4382 | 388.10101 | 10.64855 | 50.0870 |
| 2 | 23.237 | BB | 0.5652 | 386.75348 | 8.05458 | 49.9130 |

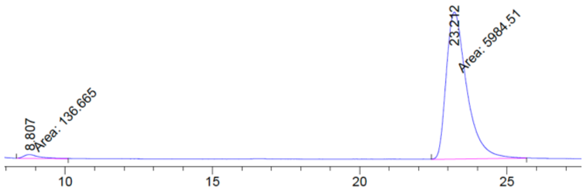


| Peak # | RetTime [min] | Type | Width [min] | Area [mAU*s] | Height [mAU] | Area % |
|---|---|---|---|---|---|---|
| 1 | 8.807 | MM | 0.6748 | 136.66451 | 3.37537 | 2.2327 |
| 2 | 23.212 | MM | 0.8073 | 5984.51318 | 123.55203 | 97.7673 |

**2-((*E*)-((6b*S*,11*R*,11a*R*)-11-pentyl-6b,11a-dihydro-11*H*-indeno[1,2-*a*]acenaphthylen-11-yl)diazenyl)pyridine (23):**

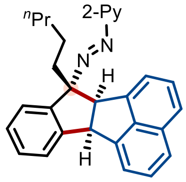


Prepared according to general procedure **GP7** on a 0.3 mmol scale, column chromatography (*n*-hexane/Acetone = 5:1) afforded the title compound as a sticky solid (88 mg, 0.21 mmol, 70%), with an enantiomeric ratio of 96.5:3.5.

**$^1$H-NMR** (400 MHz, $CDCl_3$) δ 8.71 (d, *J* = 2.3 Hz, 1H), 7.87 – 7.83 (m, 1H), 7.68 – 7.63 (m, 3H), 7.60 – 7.55 (m, 2H), 7.54 – 7.48 (m, 4H), 7.40 – 7.28 (m, 3H), 5.38 (d, *J* = 7.5 Hz, 1H), 5.32 (d, *J* = 7.6 Hz, 1H), 2.27 – 2.08 (m, 2H), 0.95 – 0.74 (m, 5H), 0.57 (t, *J* = 7.0 Hz, 3H), 0.30 – 0.15 (m, 1H) ppm;

**$^{13}$C-NMR** (101 MHz, $CDCl_3$) δ 162.9 ($C_q$), 149.4 (CH), 146.1 ($C_q$), 144.7 ($C_q$), 143.8 (2x$C_q$), 139.0 ($C_q$), 138.6 (CH), 131.7 ($C_q$), 128.9 (CH), 128.0 (2xCH), 127.5 (CH), 126.3 (CH), 125.1 (CH), 124.7 (CH), 123.6 (CH), 123.4 (CH), 122.1 (CH), 119.1 (CH), 114.2 (CH), 89.0 ($C_q$), 55.1 (CH), 54.7 (CH), 36.3 ($CH_2$), 32.2 ($CH_2$), 24.0 ($CH_2$), 22.2 ($CH_2$), 13.9 ($CH_3$) ppm;

**IR** (ATR): $\tilde{\nu}$ = 2963, 2928, 2159, 1466, 1426, 786 cm$^{-1}$;

**HRMS (ESI):** *m/z* $[M+H]^+$ calcd for $C_{29}H_{28}N_3$: 418.2283; found: 418.2279;

**$[\alpha]^D_{20}$** = - 15.63 (c =0.2, $CHCl_3$);

**$R_t$** (IA column, *n*-hexane/*i*-PrOH 90/10, 1.0 mL/min, 250.4 nm): tr(major) = 10.1 min, tr(minor) = 6.1 min, 96.5:3.5 e.r.

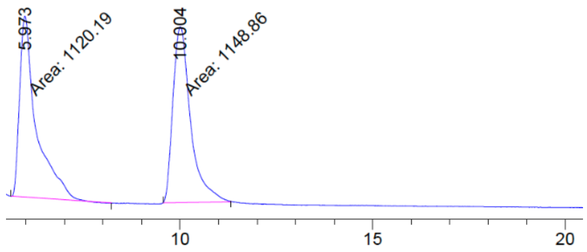


| Peak # | RetTime [min] | Type | Width [min] | Area [mAU*s] | Height [mAU] | Area % |
|---|---|---|---|---|---|---|
| 1 | 5.973 | MM | 0.5048 | 1120.18713 | 36.98470 | 49.3681 |
| 2 | 10.004 | MM | 0.5330 | 1148.86292 | 35.92739 | 50.6319 |

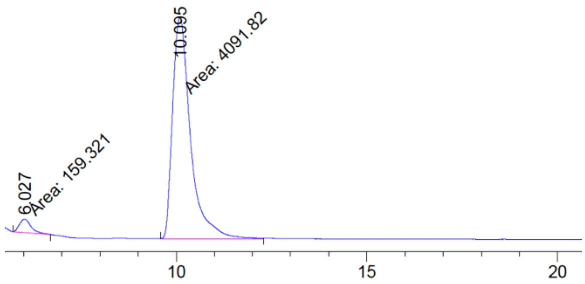


| Peak # | RetTime [min] | Type | Width [min] | Area [mAU*s] | Height [mAU] | Area % |
|---|---|---|---|---|---|---|
| 1 | 6.027 | MM | 0.3469 | 159.32066 | 7.65542 | 3.7477 |
| 2 | 10.095 | MM | 0.5439 | 4091.81616 | 125.38630 | 96.2523 |

**2-((*E*)-((6b*S*,11*R*,11a*R*)-11-(2-(l1-oxidaneyl)ethyl)-6b,11a-dihydro-11*H*-indeno[1,2-*a*]acenaphthylen-11-yl)diazenyl)pyridine (24):**

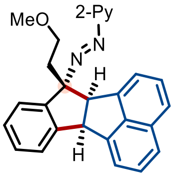


Prepared according to general procedure **GP7** on a 0.3 mmol scale, column chromatography (*n*-hexane/ethyl acetate = 3:2) afforded the title compound as a sticky solid (53 mg, 0.131 mmol, 42%), with an enantiomeric ratio of 92.5:7.5.

**$^{1}$H-NMR** (400 MHz, $CDCl_3$) δ 8.77 – 8.68 (m, 1H), 7.92 – 7.84 (m, 1H), 7.73 – 7.65 (m, 3H), 7.63 – 7.51 (m, 6H), 7.45 – 7.40 (m, 1H), 7.39 – 7.35 (m, 1H), 7.33 – 7.29 (m, 1H), 5.44 – 5.31 (m, 2H), 3.08 – 2.99 (m, 1H), 2.95 (s, 3H), 2.68 – 2.59 (m, 1H), 2.58 – 2.46 (m, 2H) ppm.

**$^{13}$C-NMR** (101 MHz, CDCl3) δ 162.7 ($C_q$), 149.4 (CH), 145.8 ($C_q$), 144.6 ($C_q$), 143.2 ($C_q$), 142.8 ($C_q$), 138.8 ($C_q$), 138.6 (CH), 131.8 ($C_q$), 129.1 (CH), 128.2 (CH), 128.0 (CH), 127.6 (CH), 126.2 (CH), 125.2 (CH), 124.8 (CH), 123.8 (CH), 123.5 (CH), 122.4 (CH), 119.1 (CH), 114.4 (CH), 87.2 ($C_q$), 68.8 ($CH_2$), 58.5 ($CH_3$), 55.4 (CH), 54.7 (CH), 35.9 ($CH_2$) ppm.

**IR** (ATR): $\tilde{\nu}$ = 3051, 2973, 2924, 2876, 1583, 1463, 1426, 1112, 785, 754 $cm^{-1}$.

**HRMS** (ESI): *m/z* $[M+Na]^+$ calcd for $C_{27}H_{23}N_3ONa$: 428.1733; found: 428.1736.

**$[\alpha]^{D}_{20}$** = - 13.1 (c = 1.0, $CHCl_3$).

**$R_t$** (IA column, *n*-hexane/*i*-PrOH 90/10, 1.0 mL/min, 250.4 nm): tr(major) = 15.9 min, tr(minor) = 11.0 min, 92.5:7.5 e.r.

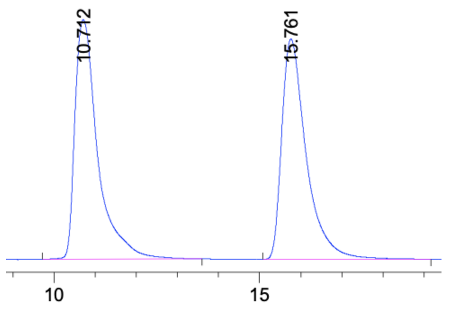


| Peak # | RetTime [min] | Type | Width [min] | Area [mAU*s] | Height [mAU] | Area % |
|---|---|---|---|---|---|---|
| 1 | 10.712 | BB | 0.5828 | 4329.55811 | 112.05909 | 50.3239 |
| 2 | 15.761 | BB | 0.6177 | 4273.83057 | 103.08632 | 49.6761 |

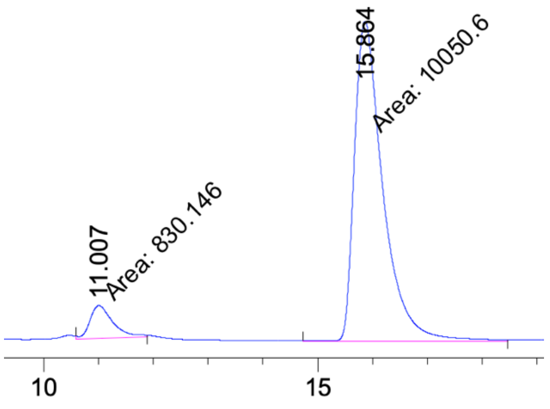


| Peak # | RetTime [min] | Type | Width [min] | Area [mAU*s] | Height [mAU] | Area % |
|---|---|---|---|---|---|---|
| 1 | 11.007 | MM | 0.4879 | 830.14636 | 28.35517 | 7.6295 |
| 2 | 15.864 | MM | 0.6038 | 1.00506e4 | 277.43561 | 92.3705 |

**2-((*E*)-((6b*S*,11*R*,11a*R*)-11-cyclopropyl-6b,11a-dihydro-11*H*-indeno[1,2-*a*]acenaphthylen-11-yl)diazenyl)pyridine (25):**

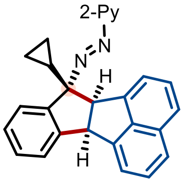


Prepared according to general procedure **GP7** on a 0.3 mmol scale, column chromatography (*n*-hexane/Acetone = 5:1) afforded the title compound as a sticky solid (59 mg, 0.15 mmol, 51%), with an enantiomeric ratio of 97:0:3.0. (10:1 diastereomeric ratio was observed)

**$^1$H-NMR** (400 MHz, $CDCl_3$) δ 8.70 – 8.66 (m, 1H), 7.85 – 7.78 (m, 1H), 7.72 (d, *J* = 6.9 Hz, 1H), 7.67 – 7.60 (m, 4H), 7.58 (d, *J* = 6.9 Hz, 1H), 7.53 – 7.47 (m, 2H), 7.45 – 7.41 (m, 1H), 7.39 – 7.34 (m, 1H), 7.33 – 7.28 (m, 1H), 7.24 (s, 1H), 5.30 – 5.24 (m, 2H), 1.26 – 1.19 (m, 1H), 0.71 – 0.60 (m, 3H), 0.41 – 0.33 (m, 1H) ppm.

**$^{13}$C-NMR** (101 MHz, $CDCl_3$) δ 162.9 ($C_q$), 149.3 (CH), 146.3 ($C_q$), 145.0 ($C_q$), 144.2 ($C_q$), 143.9 ($C_q$), 138.8 ($C_q$), 138.5 (CH), 131.7 ($C_q$), 129.0 (CH), 127.9 (CH), 127.8 (CH), 127.2 (CH), 126.5 (CH), 125.2 (CH), 124.7 (CH), 123.4 (2xCH), 123.0 (CH), 118.9 (CH), 113.0 (CH), 85.7 ($C_q$), 56.5 (CH), 54.5 (CH), 19.1 (CH), 1.2 ($CH_2$), 1.4 ($CH_2$) ppm.

**IR** (ATR): $\tilde{\nu}$ = 2870, 1590, 1481, 1459, 1432, 1373, 835, 791 $cm^{-1}$;

**HRMS (ESI):** *m/z* $[M+H]^+$ calcd for $C_{27}H_{22}N_3$: 388.1814; found: 388.1812;

**$[\alpha]^D_{20}$** = - 15.40 (c =1.0, $CHCl_3$);

**$R_t$** (IA column, *n*-hexane/*i*-PrOH 90/10, 1.0 mL/min, 250.4 nm): tr(major) = 12.9 min, tr(minor) = 7.3 min, 97.0:3.0 e.r.

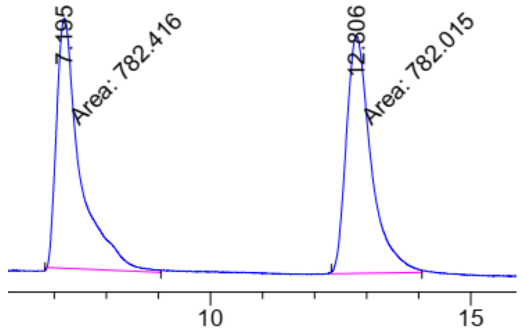


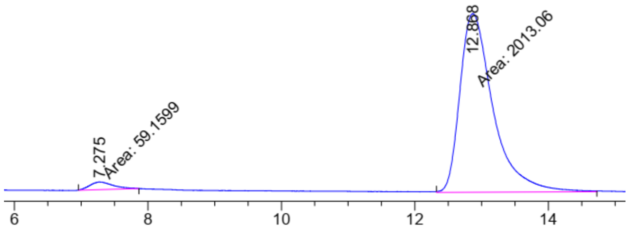


| Peak # | RetTime [min] | Type | Width [min] | Area [mAU*s] | Height [mAU] | Area % |
|---|---|---|---|---|---|---|
| 1 | 7.195 | MM | 0.5245 | 782.41553 | 24.86448 | 50.0128 |
| 2 | 12.806 | MM | 0.5522 | 782.01526 | 23.60255 | 49.9872 |

| Peak # | RetTime [min] | Type | Width [min] | Area [mAU*s] | Height [mAU] | Area % |
|---|---|---|---|---|---|---|
| 1 | 7.275 | MM | 0.3989 | 59.15994 | 2.47180 | 2.8549 |
| 2 | 12.868 | MM | 0.5761 | 2013.06116 | 58.23897 | 97.1451 |

**2-((*E*)-((6b*S*,11*R*,11a*R*)-3,4-dibromo-8,11-dimethyl-6b,11a-dihydro-11*H*-indeno[1,2-*a*]acenaphthylen-11-yl)diazenyl)pyridine (26):**

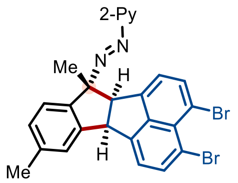


Prepared according to general procedure **GP7** on a 0.3 mmol scale, column chromatography (*n*-hexane/Acetone = 3:2) afforded the title compound as a amorphous solid (82 mg, 0.153 mmol, 51%), with an enantiomeric ratio of 95.0:5.0.

**$^{1}$H-NMR** (300 MHz, $CDCl_3$) δ 8.73 – 8.67 (m, 1H), 7.90 (d, $J$ = 2.6 Hz, 1H), 7.88 (d, $J$ = 2.7 Hz, 1H), 7.85 – 7.82 (m, 1H), 7.58 – 7.51 (m, 1H), 7.44 (dd, $J$ = 7.5, 1.1 Hz, 1H), 7.42 – 7.35 (m, 2H), 7.31 – 7.26 (m, 2H), 7.15 – 7.08 (m, 1H), 5.31 – 5.15 (m, 2H), 2.37 (s, 3H), 1.58 (s, 3H) ppm.

**$^{13}$C-NMR** (101 MHz, $CDCl_3$) δ 162.6 ($C_q$), 149.4 (CH), 147.5 ($C_q$), 145.0 ($C_q$), 143.7 ($C_q$), 141.8 ($C_q$), 141.2 ($C_q$), 139.2 ($C_q$), 138.6 (CH), 136.2 (CH), 136.0 (CH), 129.2 (CH), 128.0 ($C_q$), 125.3 (2xCH), 124.9 (CH), 124.3 (CH), 120.9 (CH), 116.1 ($C_q$), 115.9 ($C_q$), 114.4 (CH), 85.0 ($C_q$), 55.6 (CH), 53.9 (CH), 23.9 ($CH_3$), 21.6 ($CH_3$) ppm.

**IR** (ATR): $\tilde{\nu}$ = 2974, 2923, 1538, 1463, 1425, 1322, 1024, 834, 787, 753 $cm^{-1}$.

**HRMS** (ESI): *m/z* $[M+H]^+$ calcd for $C_{26}H_{20}Br_2N_3$: 534.0000; found: 534.0007.

**$[\alpha]^{D}_{20}$** = +38.4 (c = 1.0, $CHCl_3$).

**$R_t$** (IA column, *n*-hexane/*i*-PrOH 80/20, 1.0 mL/min, 250.4 nm): tr(major) = 9.6 min, tr(minor) = 7.2 min, 95.0:5.0 e.r.

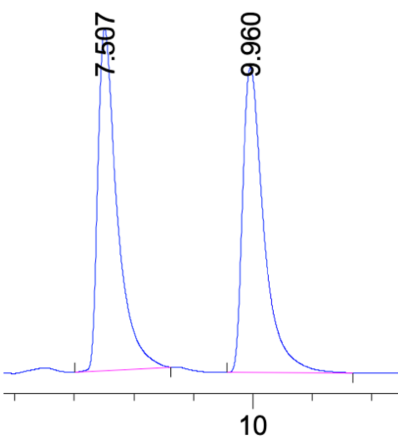


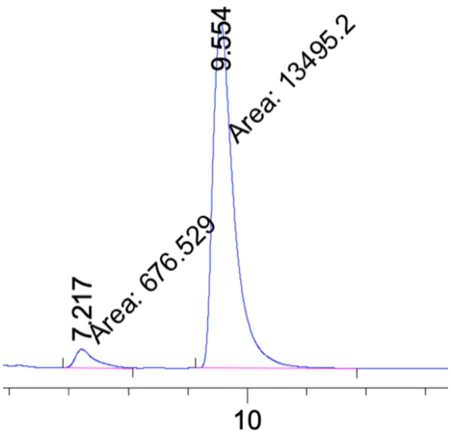


| Peak # | RetTime [min] | Type | Width [min] | Area [mAU*s] | Height [mAU] | Area % |
|---|---|---|---|---|---|---|
| 1 | 7.507 | BB | 0.3342 | 6643.98438 | 293.42618 | 50.3676 |
| 2 | 9.960 | BB | 0.3804 | 6546.99365 | 261.23599 | 49.6324 |

| Peak # | RetTime [min] | Type | Width [min] | Area [mAU*s] | Height [mAU] | Area % |
|---|---|---|---|---|---|---|
| 1 | 7.217 | MM | 0.3835 | 676.52930 | 29.40205 | 4.7738 |
| 2 | 9.554 | MM | 0.4153 | 1.34952e4 | 541.54407 | 95.2262 |

**2-((*E*)-((1*S*,2*S*)-1,5-dimethyl-3-methylene-2-phenyl-2,3-dihydro-1*H*-inden-1-yl)diazenyl)pyridine (27):**

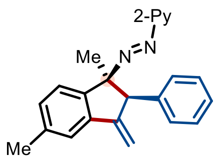


Prepared according to general procedure **GP7** on a 0.3 mmol scale, column chromatography (*n*-hexane/ethyl acetate = 5:1) afforded the title compound as a sticky solid (51 mg, 0.150 mmol, 50%, 5.0:1 d.r.), with an enantiomeric ratio of 94.0:6.0 (major diastereomer).

**$^1$H-NMR** (400 MHz, $CDCl_3$, major diastereomer) δ 8.57 – 8.49 (m, 1H), 7.65 – 7.58 (m, 1H), 7.51 (s, 1H), 7.33 (dd, $J$ = 8.3, 1.3 Hz, 2H), 7.25 – 7.08 (m, 6H), 6.91 (d, $J$ = 8.1 Hz, 1H), 5.78 (s, 1H), 5.01 (s, 1H), 4.43 – 4.37 (m, 1H), 2.41 (s, 3H), 1.78 (s, 3H) ppm.

**$^{13}$C-NMR** (101 MHz, $CDCl_3$, major diastereomer) δ 162.9 ($C_q$), 150.9 ($C_q$), 148.8 (CH), 141.9 (2x$C_q$), 138.9 ($C_q$), 138.2 (CH), 137.7 ($C_q$), 131.3 (CH), 130.6 (CH), 127.8 (CH), 127.0 (CH), 124.8 (CH), 124.7 (CH), 121.6 (CH), 111.7 (CH), 105.8 ($CH_2$), 82.1 ($C_q$), 64.6 (CH), 21.7 ($CH_3$), 21.6 ($CH_3$) ppm.

**IR** (ATR): $\tilde{\nu}$ = 3058, 3023, 2978, 2926, 1584, 1485, 1464, 1453, 1426, 701 $cm^{-1}$.

**HRMS** (ESI): *m/z* $[M+Na]^+$ calcd for $C_{23}H_{21}N_3Na$: 362.1628; found: 362.1620.

**$[\alpha]^D_{20}$** = +782.2 (c = 0.5, $CHCl_3$).

**$R_t$** (IC column, *n*-hexane/*i*-PrOH 90/10, 1.0 mL/min, 250.4 nm): tr(major) = 9.6 min, tr(minor) = 11.7 min, 94:6 e.r.

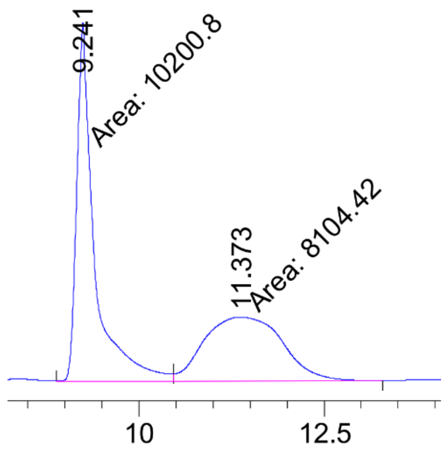


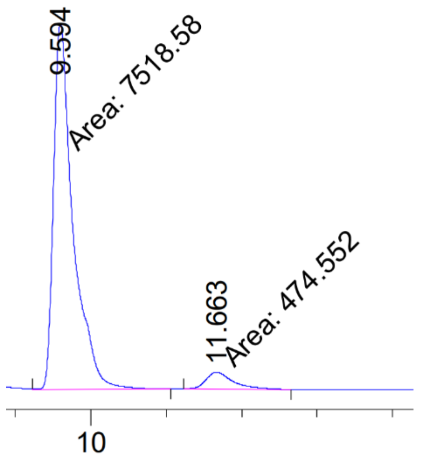


| Peak # | RetTime [min] | Type | Width [min] | Area [mAU*s] | Height [mAU] | Area % |
|---|---|---|---|---|---|---|
| 1 | 9.241 | MF | 0.2766 | 1.02008e4 | 614.66797 | 55.7261 |
| 2 | 11.373 | FM | 1.2308 | 8104.42041 | 109.74201 | 44.2739 |

| Peak # | RetTime [min] | Type | Width [min] | Area [mAU*s] | Height [mAU] | Area % |
|---|---|---|---|---|---|---|
| 1 | 9.594 | MM | 0.3006 | 7518.57666 | 416.84509 | 94.0630 |
| 2 | 11.663 | MM | 0.4157 | 474.55237 | 19.02572 | 5.9370 |

**2-((*E*)-((1*S*,2*S*)-5-methoxy-1-methyl-3-methylene-2-phenyl-2,3-dihydro-1*H*-inden-1-yl)diazenyl)pyridine (28):**

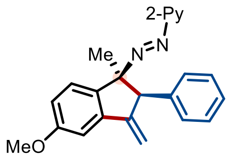


Prepared according to general procedure **GP7** on a 0.3 mmol scale, column chromatography (*n*-hexane/Acetone = 5:1) afforded the title compound as a sticky solid (43 mg, 0.121 mmol, 40%, 4.0:1 d.r.), with an enantiomeric ratio of 97.5:2.5 (major diastereomer).

**$^1$H-NMR** (400 MHz, $CDCl_3$, major diastereomer) δ 8.54 (ddd, *J* = 4.8, 1.9, 0.9 Hz, 1H), 7.62 (ddd, *J* = 8.1, 7.4, 1.9 Hz, 1H), 7.37 – 7.15 (m, 7H), 7.13 (d, *J* = 8.4 Hz, 1H), 6.96 – 6.83 (m, 2H), 5.77 (d, *J* = 3.0 Hz, 1H), 5.04 (d, *J* = 2.6 Hz, 1H), 4.50 – 4.38 (m, 1H), 3.86 (s, 3H), 1.78 (s, 3H) ppm.

**$^{13}$C-NMR** (101 MHz, $CDCl_3$, major diastereomer) δ 162.9 ($C_q$), 160.7 ($C_q$), 150.8 ($C_q$), 148.8 (CH), 143.2 ($C_q$), 138.2 (CH), 137.6 ($C_q$), 137.1 ($C_q$), 131.3 (CH), 127.8 (CH), 127.1 (CH), 125.9 (CH), 124.8 (CH), 116.7 (CH), 111.7 (CH), 106.1 ($CH_2$), 105.2 (CH), 81.8 ($C_q$), 64.8 (CH), 55.6 ($CH_3$), 21.6 ($CH_3$) ppm.

**IR** (ATR): $\tilde{\nu}$ = 2971, 2931, 1604, 1583, 1486, 1427, 1249, 1226, 1027, 755, 701 $cm^{-1}$.

**HRMS** (ESI): *m/z* $[M+Na]^+$ calcd for $C_{23}H_{21}N_3ONa$: 378.1577; found: 378.1576.

**$[\alpha]^D_{20}$** = +612.7 (c = 1.0, $CHCl_3$).

**$R_t$** (IC column, *n*-hexane/*i*-PrOH 95/5, 1.0 mL/min, 273.4 nm): tr(major) = 17.4 min, tr(minor) = 27.6 min, 97.5:2.5 e.r. (major diastereomer)

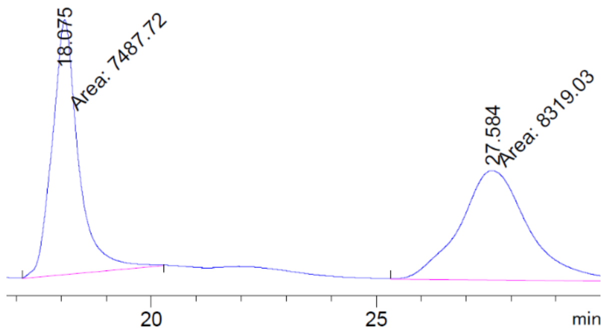


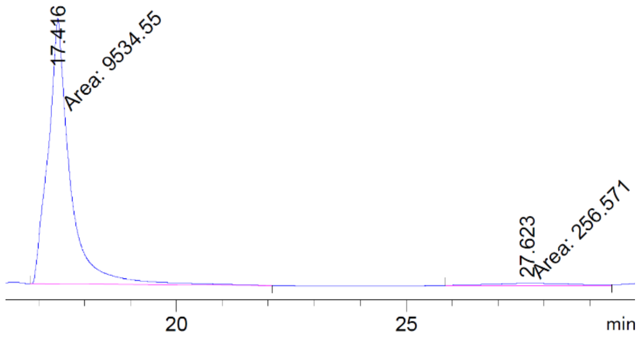


| Peak # | RetTime [min] | Type | Width [min] | Area [mAU*s] | Height [mAU] | Area % |
|---|---|---|---|---|---|---|
| 1 | 18.075 | MM | 0.6771 | 7487.72070 | 184.31721 | 47.3704 |
| 2 | 27.584 | MM | 1.7543 | 8319.02832 | 79.03427 | 52.6296 |

| Peak # | RetTime [min] | Type | Width [min] | Area [mAU*s] | Height [mAU] | Area % |
|---|---|---|---|---|---|---|
| 1 | 17.416 | MM | 0.5533 | 9534.54883 | 287.19348 | 97.3796 |
| 2 | 27.623 | MM | 1.8228 | 256.57053 | 2.34590 | 2.6204 |

**2-((*E*)-((1*S*,2*S*)-5-methyl-3-methylene-2-phenyl-1-propyl-2,3-dihydro-1*H*-inden-1-yl)diazenyl)pyridine (29):**

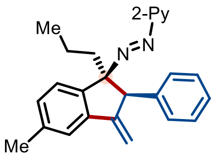


Prepared according to general procedure **GP7** on a 0.3 mmol scale, column chromatography (*n*-hexane/Acetone = 6:1) afforded the title compound as a sticky solid (55 mg, 0.156 mmol, 52%, 3.0:1 d.r.), with an enantiomeric ratio of 92.5:7.5 (major diastereomer).

**$^1$H-NMR** (400 MHz, $CDCl_3$) δ 8.62 – 8.51 (m, 1H), 7.68 – 7.64 (m, 1H), 7.58 (ddd, $J$ = 8.2, 7.4, 1.9 Hz, 1H), 7.40 – 7.13 (m, 9H), 6.70 (d, $J$ = 8.1 Hz, 1H), 5.77 (d, $J$ = 2.7 Hz, 1H), 5.04 (d, $J$ = 2.3 Hz, 1H), 4.68 – 4.60 (m, 1H), 2.37 (ddd, $J$ = 14.1, 12.5, 4.1 Hz, 1H), 2.07 (ddd, $J$ = 14.2, 12.4, 4.7 Hz, 1H), 1.60 – 1.44 (m, 1H), 1.31 – 1.22 (m, 1H), 0.97 (t, $J$ = 7.3 Hz, 3H) ppm.

**$^{13}$C-NMR** (101 MHz, $CDCl_3$) δ 163.0 ($C_q$), 151.4 ($C_q$), 148.8 (CH), 143.6 ($C_q$), 142.0 ($C_q$), 139.2 ($C_q$), 138.2 (CH), 130.9 (CH), 129.3 (CH), 128.7 (CH), 127.9 (CH), 126.7 (CH), 126.0 (CH), 124.7 (CH), 120.9 (CH), 111.6 (CH), 106.1 ($CH_2$), 85.5 ($C_q$), 59.8 (CH), 37.6 ($CH_2$), 18.2 ($CH_2$), 14.8 ($CH_3$) ppm.
**IR** (ATR): $\tilde{\nu}$ = 2960, 2932, 2871, 1583, 1494, 1464, 1426, 882, 786, 760, 701 $cm^{-1}$.

**HRMS** (ESI): *m/z* $[M+Na]^+$ calcd for $C_{24}H_{23}N_3Na$: 376.1784; found: 376.1785.

**$[\alpha]^D_{20}$** = +347.7 (c = 1.0, $CHCl_3$).

**$R_t$** (IC column, *n*-hexane/*i*-PrOH 95/5, 1.0 mL/min, 250.4 nm): tr(major) = 15.0 min, tr(minor) = 9.2 min, 92.5:7.5 e.r. (major diastereomer).

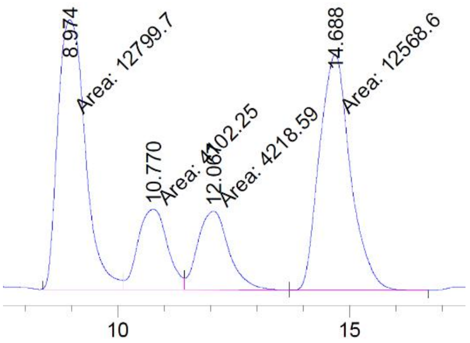


| Peak # | RetTime [min] | Type | Width [min] | Area [mAU*s] | Height [mAU] | Area % |
|---|---|---|---|---|---|---|
| 1 | 8.974 | MF | 0.6876 | 1.27997e4 | 310.23413 | 37.9935 |
| 2 | 10.770 | MF | 0.7399 | 4102.24609 | 92.40866 | 12.1768 |
| 3 | 12.067 | MF | 0.7780 | 4218.59326 | 90.36832 | 12.5221 |
| 4 | 14.688 | FM | 0.7728 | 1.25686e4 | 271.07745 | 37.3075 |

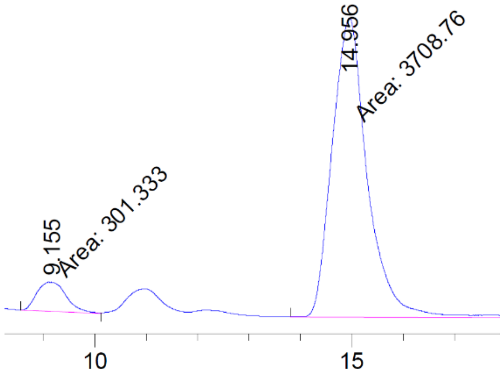


| Peak # | RetTime [min] | Type | Width [min] | Area [mAU*s] | Height [mAU] | Area % |
|---|---|---|---|---|---|---|
| 1 | 9.155 | MM | 0.6628 | 301.33267 | 7.57742 | 7.5144 |
| 2 | 14.956 | MM | 0.8005 | 3708.75830 | 77.22076 | 92.4856 |

**2-((*E*)-((2'*S*,3'*R*)-3',6'-dimethyl-2'-phenyl-2',3'-dihydrospiro[cyclopropane-1,1'-inden]-3'-yl)diazenyl)pyridine (30):**

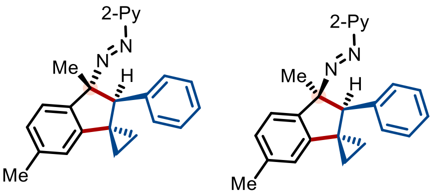


(major) (minor)

Prepared according to general procedure **GP7** on a 0.3 mmol scale, column chromatography (*n*-hexane/ethyl acetate = 3:1) afforded the title compound as a yellow sticky solid (64 mg, 0.18 mmol, 60%), with an enantiomeric ratio of 98.0:2.0 (major isomer) and 99.0:1.0 e.r. (minor isomer). (4:1 d.r. was obtained)

**$^1$H-NMR** (400 MHz, $CDCl_3$, major isomer) δ 8.68 – 8.63 (m, 1H), 7.81 – 7.73 (m, 1H), 7.45 – 7.41 (m, 1H), 7.36 – 7.30 (m, 1H), 7.24 – 7.18 (m, 4H), 7.14 – 7.04 (m, 5H), 6.63 (d, $J$ = 1.7 Hz, 1H), 3.94 (s, 1H), 2.37 (s, 3H), 1.36 (s, 3H), 1.22 – 1.14 (m, 2H), 0.87 – 0.81 (m, 1H), 0.78 – 0.71 (m, 1H) ppm.

**$^{13}$C-NMR** (101 MHz, $CDCl_3$, major isomer) δ 162.9 ($C_q$), 149.7 ($C_q$), 149.2 (CH), 141.8 ($C_q$), 141.2 ($C_q$), 138.9 ($C_q$), 138.4 (CH), 129.8 (CH), 128.0 (CH), 127.4 (CH), 126.5 (CH), 124.9 (CH), 124.7 (CH), 119.0 (CH), 113.9 (CH), 86.3 ($C_q$), 60.7 (CH), 30.9 ($C_q$), 21.7 ($CH_3$), 20.9 ($CH_3$), 20.7 ($CH_2$), 12.6 ($CH_2$) ppm.

**IR** (ATR): $\tilde{\nu}$ = 2996, 2925, 1680, 1582, 1492, 1458, 1426, 1033, 787, 703 $cm^{-1}$.

**HRMS** (ESI): *m/z* $[M+Na]^+$ calcd for $C_{24}H_{23}N_3Na$: 376.1784; found: 376.1784.

**$[\alpha]^D_{20}$** = +138.9 (c = 1.0, $CHCl_3$).

*major isomer:*

**$R_t$** (IC column, *n*-hexane/*i*-PrOH 98/2, 1.0 mL/min, 250.4 nm): tr(major) = 20.9 min, tr(minor) = 17.7 min, 98.0:2.0 e.r.

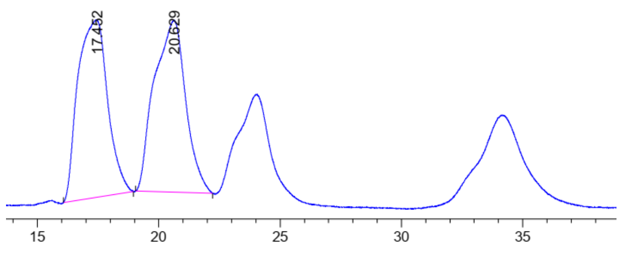


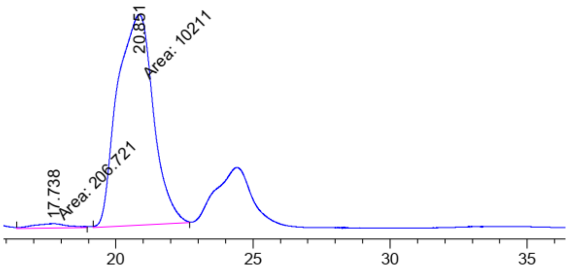


| Peak # | RetTime [min] | Type | Width [min] | Area [mAU*s] | Height [mAU] | Area % |
|---|---|---|---|---|---|---|
| 1 | 17.452 | BB | 1.0049 | 1360.72742 | 15.95703 | 49.5789 |
| 2 | 20.629 | BB | 1.0515 | 1383.84045 | 15.45765 | 50.4211 |

| Peak # | RetTime [min] | Type | Width [min] | Area [mAU*s] | Height [mAU] | Area % |
|---|---|---|---|---|---|---|
| 1 | 17.738 | MM | 1.4660 | 206.72133 | 2.35017 | 1.9843 |
| 2 | 20.851 | MM | 1.5166 | 1.02110e4 | 112.21325 | 98.0157 |

*minor isomer:*

**R$_t$** (IC column, *n*-hexane/*i*-PrOH 98/2, 1.0 mL/min, 250.4 nm): tr(major) = 24.4 min, tr(minor) = 34.5 min, 99.0:1.0 e.r.

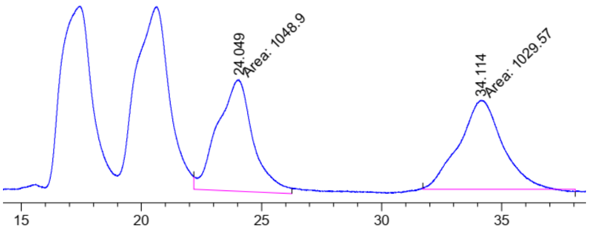


| Peak # | RetTime [min] | Type | Width [min] | Area [mAU*s] | Height [mAU] | Area % |
|---|---|---|---|---|---|---|
| 1 | 24.049 | MM | 1.7227 | 1048.89771 | 10.14798 | 50.4651 |
| 2 | 34.114 | MM | 2.1151 | 1029.56555 | 8.11287 | 49.5349 |

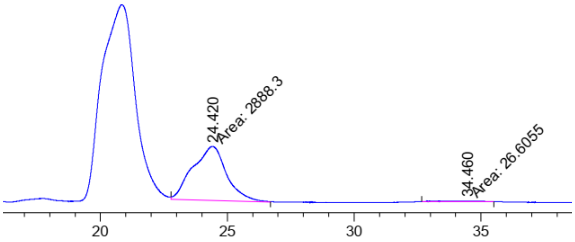


| Peak # | RetTime [min] | Type | Width [min] | Area [mAU*s] | Height [mAU] | Area % |
|---|---|---|---|---|---|---|
| 1 | 24.420 | MM | 1.5490 | 2888.30200 | 31.07755 | 99.0873 |
| 2 | 34.460 | MM | 1.2917 | 26.60551 | 3.43277e-1 | 0.9127 |

**2-((*E*)-((2'*S*,3'*R*)-2'-(4-bromophenyl)-3',6'-dimethyl-2',3'-dihydrospiro[cyclopropane-1,1'-inden]-3'-yl)diazenyl)pyridine (31):**

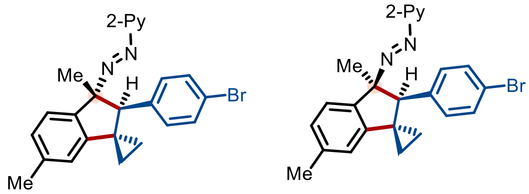


(major) (minor)

Prepared according to general procedure **GP7** on a 0.3 mmol scale, column chromatography (*n*-hexane/ethyl acetate = 3:1) afforded the title compound as a yellow sticky solid (84 mg, 0.2 mmol, 65%), with an enantiomeric ratio of 97.5:2.5 (major isomer) and 97.5:2.5 (minor isomer). (3:1 d.r. was obtained)

**$^{1}$H-NMR** (400 MHz, $CDCl_3$, major isomer) δ 8.67 – 8.64 (m, 1H), 7.81 – 7.74 (m, 1H), 7.45 – 7.40 (m, 1H), 7.36 – 7.31 (m, 2H), 7.28 – 7.20 (m, 2H), 7.08 – 7.02 (m, 1H), 7.00 – 6.94 (m, 2H), 6.63 – 6.58 (m, 1H), 3.91 (s, 1H), 2.36 (s, 3H), 1.34 (s, 3H), 1.20 – 1.12 (m, 2H), 0.86 – 0.80 (m, 1H), 0.73 – 0.66 (m, 1H) ppm.

**$^{13}$C-NMR** (101 MHz, $CDCl_3$, major isomer) δ 162.7 ($C_q$), 149.2 (2$C_q$), 140.9 ($C_q$), 139.1 ($C_q$), 138.4 (CH), 138.3 (CH), 131.4 (CH), 131.1 (CH), 127.5 (CH), 124.9 (CH), 124.7 (CH), 120.4 ($C_q$), 119.0 (CH), 114.0 (CH), 86.0 ($C_q$), 59.8 (CH), 30.8 ($C_q$), 21.7 ($CH_3$), 20.9 ($CH_3$), 20.7 ($CH_2$), 12.5 ($CH_2$) ppm.

**IR** (ATR): $\tilde{\nu}$ = 2972, 2928, 1612, 1584, 1487, 1426, 1011, 813, 756 $cm^{-1}$.

**HRMS** (ESI): *m/z* $[M+Na]^+$ calcd for $C_{24}H_{22}N_3BrNa$: 454.0889; found: 454.0889.

**$[\alpha]^D_{20}$** = +117.6 (c = 1.0, $CHCl_3$).

*major isomer:*

**$R_t$** (IC column, *n*-hexane/*i*-PrOH 98/2, 1.0 mL/min, 250.4 nm): tr(major) = 20.2 min, tr(minor) = 16.9 min, 97.5:2.5 e.r.

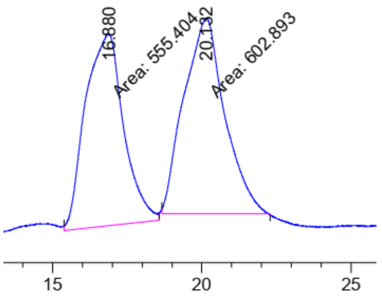


| Peak # | RetTime [min] | Type | Width [min] | Area [mAU*s] | Height [mAU] | Area % |
|---|---|---|---|---|---|---|
| 1 | 16.880 | MM | 1.4873 | 555.40424 | 6.22386 | 47.9501 |
| 2 | 20.132 | MM | 1.5814 | 602.89264 | 6.35395 | 52.0499 |

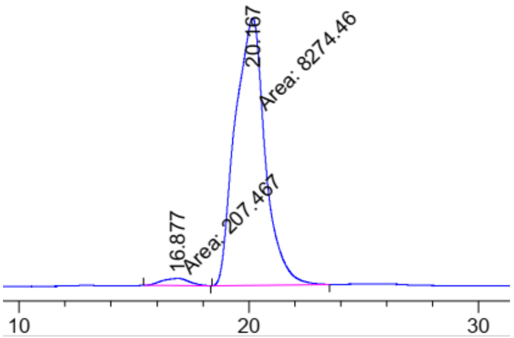


| Peak # | RetTime [min] | Type | Width [min] | Area [mAU*s] | Height [mAU] | Area % |
|---|---|---|---|---|---|---|
| 1 | 16.877 | MM | 1.3967 | 207.46709 | 2.47563 | 2.4460 |
| 2 | 20.167 | MM | 1.5094 | 8274.46484 | 91.36431 | 97.5540 |

*minor isomer:*

**$R_t$** (IC column, *n*-hexane/*i*-PrOH 98/2, 1.0 mL/min, 250.4 nm): tr(major) = 20.7 min, tr(minor) = 29.1 min, 97.5:2.5 e.r.

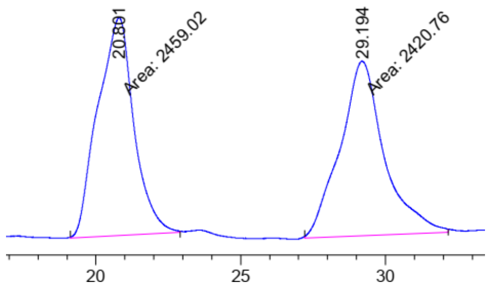


| Peak # | RetTime [min] | Type | Width [min] | Area [mAU*s] | Height [mAU] | Area % |
|---|---|---|---|---|---|---|
| 1 | 20.801 | MM | 1.4431 | 2459.01904 | 28.40033 | 50.3920 |
| 2 | 29.194 | MM | 1.7736 | 2420.76489 | 22.74829 | 49.6080 |

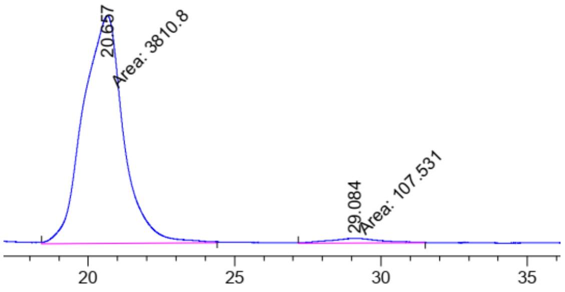


| Peak # | RetTime [min] | Type | Width [min] | Area [mAU*s] | Height [mAU] | Area % |
|---|---|---|---|---|---|---|
| 1 | 20.657 | MM | 1.5933 | 3810.80249 | 39.86391 | 97.2557 |
| 2 | 29.084 | MM | 2.1319 | 107.53119 | 8.40635e-1 | 2.7443 |

**2-((*E*)-((2'*S*,3'*R*)-2'-(4-bromophenyl)-3'-methyl-6'-(methylthio)-2',3'-dihydrospiro[cyclopropane-1,1'-inden]-3'-yl)diazenyl)pyridine (32):**

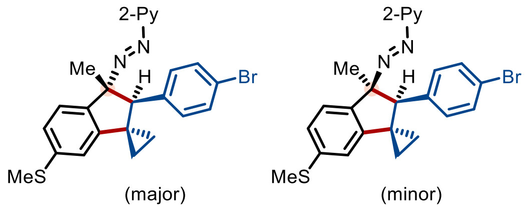


Prepared according to general procedure **GP7** on a 0.3 mmol scale, column chromatography (*n*-hexane/ethyl acetate = 3:1) afforded the title compound as a yellow sticky solid (82 mg, 0.18 mmol, 59%), with an enantiomeric ratio of 99.0:1.0 (major isomer) and 98.5:1.5 (minor isomer). (3:1 d.r. was obtained)

**$^{1}$H-NMR** (400 MHz, $CDCl_3$, major isomer) δ 8.61 – 8.56 (m, 1H), 7.76 – 7.68 (m, 1H), 7.38 – 7.33 (m, 1H), 7.29 – 7.25 (m, 2H), 7.20 – 7.15 (m, 2H), 7.08 – 7.00 (m, 1H), 6.90 – 6.86 (m, 2H), 6.64 – 6.57 (m, 1H), 3.83 (s, 1H), 2.41 (s, 3H), 1.26 (s, 3H), 1.14 – 1.06 (m, 2H), 0.79 – 0.72 (m, 1H), 0.67 – 0.59 (m, 1H) ppm.

**$^{13}$C-NMR** (101 MHz, $CDCl_3$, major isomer) δ 162.6 ($C_q$), 150.0 ($C_q$), 149.3 (CH), 140.9 ($C_q$), 140.7 ($C_q$), 139.7 ($C_q$), 138.5 (CH), 131.4 (CH), 131.3 (CH), 125.4 (CH), 125.1 (CH), 124.7 (CH), 120.5 ($C_q$), 116.4 (CH), 114.2 (CH), 86.0 ($C_q$), 59.8 (CH), 30.9 ($C_q$), 21.0 ($CH_2$), 20.9 ($CH_3$), 16.0 ($CH_3$), 12.6 ($CH_2$) ppm.

**IR** (ATR): $\tilde{\nu}$ = 2981, 2922, 1674, 1586, 1485, 1425, 1073, 1011, 784 $cm^{-1}$.

**HRMS** (ESI): *m/z* $[M+Na]^+$ calcd for $C_{24}H_{22}N_3BrSNa$: 486.0608; found: 486.0610.

**$[\alpha]^D_{20}$** = +111.8 (c = 0.5, $CHCl_3$).

*major isomer:*

**$R_t$** (IA column, *n*-hexane/*i*-PrOH 98/2, 1.0 mL/min, 250.4 nm): tr(major) = 18.7 min, tr(minor) = 13.7 min, 99.0:1.0 e.r.

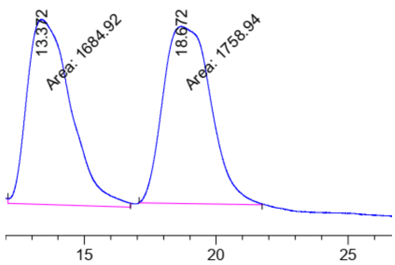


| Peak # | RetTime [min] | Type | Width [min] | Area [mAU*s] | Height [mAU] | Area % |
|---|---|---|---|---|---|---|
| 1 | 13.372 | MM | 1.9274 | 1684.92078 | 14.56966 | 48.9253 |
| 2 | 18.672 | MM | 2.1034 | 1758.94214 | 13.93712 | 51.0747 |

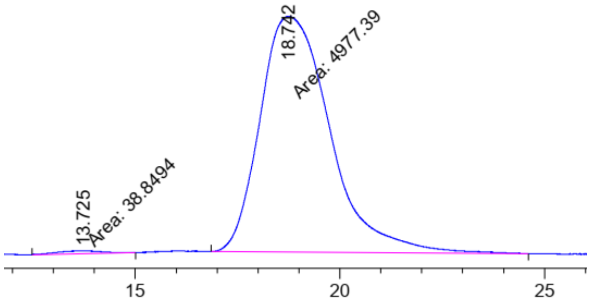


| Peak # | RetTime [min] | Type | Width [min] | Area [mAU*s] | Height [mAU] | Area % |
|---|---|---|---|---|---|---|
| 1 | 13.725 | MM | 1.3183 | 38.84937 | 4.91151e-1 | 0.7745 |
| 2 | 18.742 | MM | 1.9859 | 4977.38672 | 41.77307 | 99.2255 |

*minor isomer:*

**$R_t$** (IC column, *n*-hexane/*i*-PrOH 98/2, 1.0 mL/min, 250.4 nm): tr(major) = 29.0 min, tr(minor) = 52.2 min, 98.5:1.5 e.r.

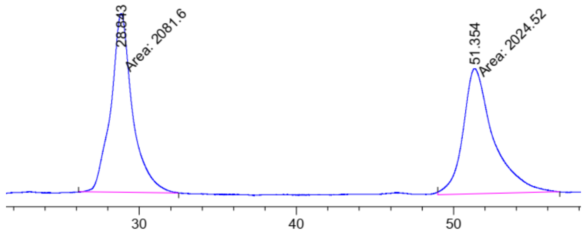


| Peak # | RetTime [min] | Type | Width [min] | Area [mAU*s] | Height [mAU] | Area % |
|---|---|---|---|---|---|---|
| 1 | 28.843 | MM | 1.6086 | 2081.60376 | 21.56788 | 50.6951 |
| 2 | 51.354 | MM | 2.2317 | 2024.52039 | 15.11969 | 49.3049 |

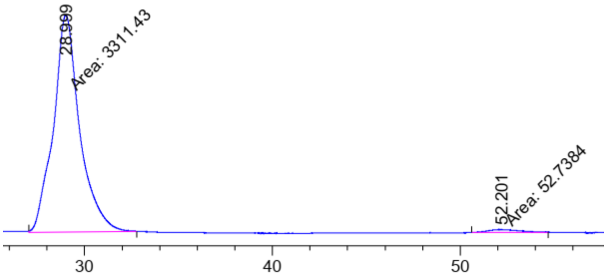


| Peak # | RetTime [min] | Type | Width [min] | Area [mAU*s] | Height [mAU] | Area % |
|---|---|---|---|---|---|---|
| 1 | 28.999 | MM | 1.6290 | 3311.43237 | 33.88099 | 98.4323 |
| 2 | 52.201 | MM | 1.8959 | 52.73844 | 4.63609e-1 | 1.5677 |

**2-((*E*)-((2'*S*,3'*R*)-2'-(4-bromophenyl)-6'-iodo-3'-methyl-2',3'-dihydrospiro[cyclopropane-1,1'-inden]-3'-yl)diazenyl)pyridine (33):**

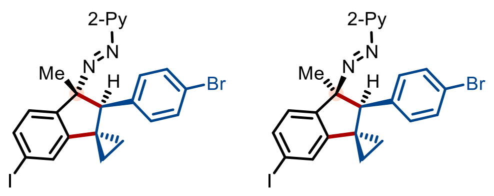

(major) (minor)

Prepared according to general procedure **GP7** on a 0.3 mmol scale, column chromatography (*n*-hexane/ethyl acetate = 3:1) afforded the title compound as a yellow sticky solid (67 mg, 0.14 mmol, 45%), with an enantiomeric ratio of 96.0:4.0 (major isomer) and 93.0:7.0 (minor isomer). (3:1 d.r. was obtained)

**$^{1}$H-NMR** (400 MHz, $CDCl_3$, major isomer) δ 8.67 – 8.63 (m, 1H), 7.84 – 7.77 (m, 1H), 7.59 – 7.55 (m, 1H), 7.45 – 7.40 (m, 1H), 7.35 (d, $J$ = 8.5 Hz, 2H), 7.28 – 7.24 (m, 1H), 7.14 – 7.11 (m, 1H), 7.09 – 7.05 (m, 1H), 6.97 – 6.90 (m, 2H), 3.87 (s, 1H), 1.33 (s, 3H), 1.21 – 1.16 (m, 2H), 0.87 – 0.79 (m, 1H), 0.76 – 0.68 (m, 1H) ppm.

**$^{13}$C-NMR** (101 MHz, $CDCl_3$, major isomer) δ 162.5 ($C_q$), 151.8 ($C_q$), 149.4 (CH), 143.6 ($C_q$), 140.3 ($C_q$), 138.5 (CH), 135.7 (CH), 131.4 (CH), 131.3 (CH), 127.8 (CH), 127.0 (CH), 125.2 (CH), 120.7 ($C_q$), 114.5 (CH), 95.3 ($C_q$), 86.0 ($C_q$), 59.7 (CH), 30.7 ($C_q$), 21.3 ($CH_2$), 20.8 ($CH_3$), 12.8 ($CH_2$) ppm.

**IR** (ATR): $\tilde{\nu}$ = 2972, 1713, 1652, 1588, 1486, 1409, 1277, 1010, 755 cm$^{-1}$.

**HRMS** (ESI): *m/z* $[M+Na]^+$ calcd for $C_{23}H_{19}N_3BrINa$: 565.9701; found: 565.9699.

**$[\alpha]^{D}_{20}$** = +35.6 (c = 1.0, $CHCl_3$).

*major isomer:*

**$R_t$** (IC column, *n*-hexane/*i*-PrOH 98/2, 1.0 mL/min, 250.4 nm): tr(major) = 17.7 min, tr(minor) = 15.5 min, 96.0:4.0 e.r.

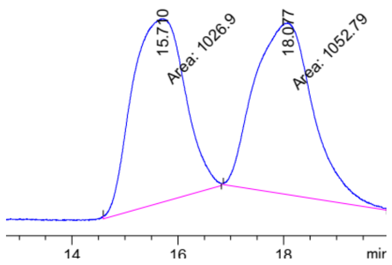


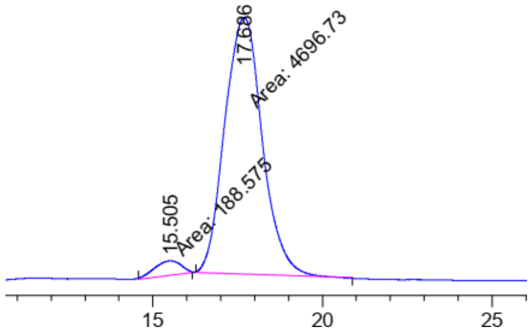


| Peak # | RetTime [min] | Type | Width [min] | Area [mAU*s] | Height [mAU] | Area % |
|---|---|---|---|---|---|---|
| 1 | 15.710 | MM | 1.1671 | 1026.89661 | 14.66505 | 49.3774 |
| 2 | 18.077 | MM | 1.2785 | 1052.79358 | 13.72408 | 50.6226 |

| Peak # | RetTime [min] | Type | Width [min] | Area [mAU*s] | Height [mAU] | Area % |
|---|---|---|---|---|---|---|
| 1 | 15.505 | MM | 0.9217 | 188.57539 | 3.40977 | 3.8600 |
| 2 | 17.686 | MM | 1.2822 | 4696.73486 | 61.04826 | 96.1400 |

*minor isomer:*

**R$_t$** (IC column, *n*-hexane/*i*-PrOH 98/2, 1.0 mL/min, 250.4 nm): tr(major) = 17.6 min, tr(minor) = 29.9 min, 93.0:7.0 e.r.

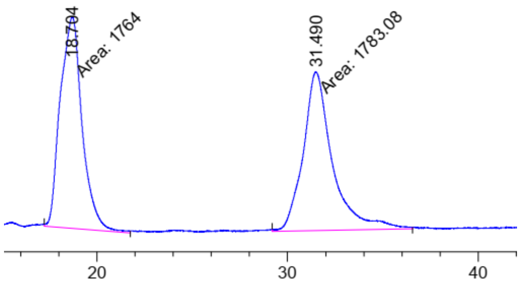


| Peak # | RetTime [min] | Type | Width [min] | Area [mAU*s] | Height [mAU] | Area % |
|---|---|---|---|---|---|---|
| 1 | 18.704 | MM | 1.3230 | 1764.00146 | 22.22241 | 49.7311 |
| 2 | 31.490 | MM | 1.7807 | 1783.07617 | 16.68846 | 50.2689 |

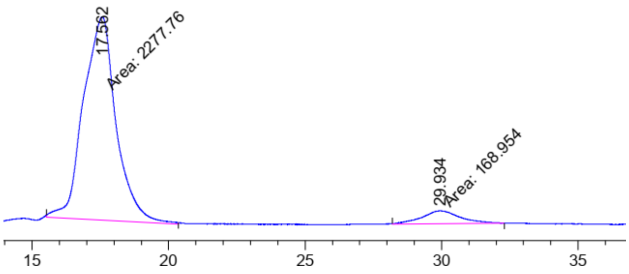


| Peak # | RetTime [min] | Type | Width [min] | Area [mAU*s] | Height [mAU] | Area % |
|---|---|---|---|---|---|---|
| 1 | 17.562 | MM | 1.3833 | 2277.75659 | 27.44418 | 93.0946 |
| 2 | 29.934 | MM | 1.6118 | 168.95396 | 1.74711 | 6.9054 |

**(6b*S*,11*R*,11a*R*)-9-methoxy-11-methyl-11-((*E*)-pyridin-2-yldiazenyl)-6b,11a-dihydro-11*H*-indeno[1,2-*a*]acenaphthylen-8-yl pivalate (34):**

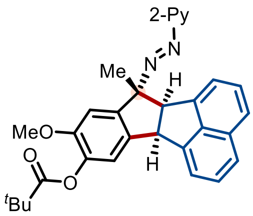


Prepared according to general procedure **GP7** on a 0.3 mmol scale, column chromatography (DCM/Acetone = 20:1) afforded the title compound as a sticky solid (85 mg, 0.173 mmol, 58%), with an enantiomeric ratio of 83.5:16.5.

**$^1$H-NMR** (400 MHz, $CDCl_3$) δ 8.75 – 8.64 (m, 1H), 7.92 – 7.81 (m, 1H), 7.69 – 7.63 (m, 2H), 7.60 – 7.54 (m, 2H), 7.54 – 7.48 (m, 2H), 7.45 (d, *J* = 7.0 Hz, 1H), 7.42 – 7.36 (m, 1H), 7.28 (s, 1H), 6.89 (s, 1H), 5.36 (d, *J* = 7.3 Hz, 1H), 5.31 (d, *J* = 7.3 Hz, 1H), 3.74 (s, 3H), 1.67 (s, 3H), 1.37 (s, 9H) ppm.

**$^{13}$C-NMR** (101 MHz, $CDCl_3$) δ 176.8 ($C_q$), 162.6 ($C_q$), 151.3 ($C_q$), 149.4 (CH), 146.1 ($C_q$), 143.5 ($C_q$), 142.5 ($C_q$), 141.4 ($C_q$), 138.6 (CH), 138.5 ($C_q$), 136.7 ($C_q$), 131.8 ($C_q$), 128.1 (CH), 127.9 (CH), 125.2 (CH), 123.6 (CH), 123.4 (CH), 122.6 (CH), 119.0 (CH), 118.4 (CH), 114.7 (CH), 108.8 (CH), 85.4 ($C_q$), 57.1 (CH), 56.3 ($CH_3$), 54.2 (CH), 39.2 ($C_q$), 27.4 ($CH_3$), 23.7 ($CH_3$) ppm.

**IR** (ATR): $\tilde{\nu}$ = 2976, 1752, 1496, 1320, 1277, 1114, 1050, 785, 753, 397 $cm^{-1}$.

**HRMS** (ESI): *m/z* $[M+Na]^+$ calcd for $C_{31}H_{29}N_3O_3Na$: 514.2101; found: 514.2106.

**$[\alpha]^D_{20}$** = - 5.1 (c = 1.0, $CHCl_3$).

**$R_t$** (IE column, *n*-hexane/*i*-PrOH 80/20, 1.0 mL/min, 250.4 nm): tr(major) = 16.1 min, tr(minor) = 14.3 min, 83.5:16.5 e.r.

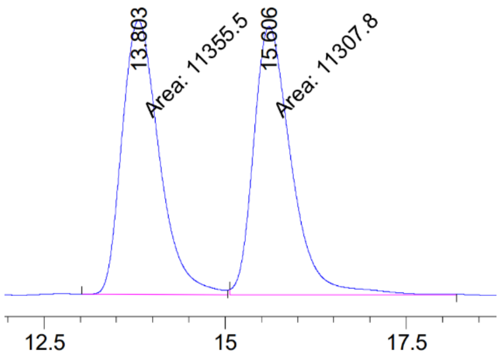


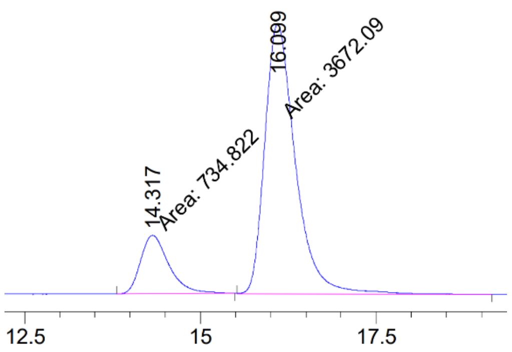


| Peak # | RetTime [min] | Type | Width [min] | Area [mAU*s] | Height [mAU] | Area % |
|---|---|---|---|---|---|---|
| 1 | 13.803 | MM | 0.5963 | 1.13555e4 | 317.38916 | 50.1052 |
| 2 | 15.606 | MM | 0.6063 | 1.13078e4 | 310.84970 | 49.8948 |

| Peak # | RetTime [min] | Type | Width [min] | Area [mAU*s] | Height [mAU] | Area % |
|---|---|---|---|---|---|---|
| 1 | 14.317 | MM | 0.4671 | 734.82233 | 26.21672 | 16.6743 |
| 2 | 16.099 | MM | 0.5061 | 3672.08691 | 120.93077 | 83.3257 |

**5-(2,5-dimethylphenoxy)-2,2-dimethyl-*N*-((6bS,11R,11aR)-11-methyl-11-((*E*)-pyridin-2-yldiazenyl)-6b,11a-dihydro-11H-indeno[1,2-*a*]acenaphthylen-8-yl)pentanamide (35):**

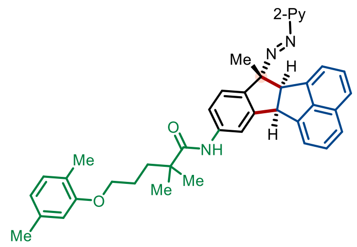


Prepared according to general procedure **GP7** on a 0.3 mmol scale, column chromatography (*n*-hexane/ethyl acetate = 2:1) afforded the title compound as a yellow sticky solid (86 mg, 0.14 mmol, 47%), with an enantiomeric ratio of 97.5:2.5.

**$^{1}$H-NMR** (300 MHz, $CDCl_3$) δ 8.70 (d, $J$ = 3.8 Hz, 1H), 8.04 (s, 1H), 7.90 – 7.80 (m, 1H), 7.70 – 7.60 (m, 3H), 7.59 – 7.34 (m, 7H), 7.24 (d, $J$ = 1.7 Hz, 1H), 6.99 (d, $J$ = 7.5 Hz, 1H), 6.69 – 6.59 (m, 2H), 5.38 (d, $J$ = 7.3 Hz, 1H), 5.30 (d, $J$ = 7.3 Hz, 1H), 3.94 (s, 2H), 2.28 (s, 3H), 2.17 (s, 3H), 1.83 (s, 4H), 1.69 (s, 3H), 1.34 (s, 6H) ppm.

**$^{13}$C-NMR** (75 MHz, $CDCl_3$) δ 175.8 ($C_q$), 162.7 ($C_q$), 157.0 ($C_q$), 149.4 (CH), 146.0 ($C_q$), 145.9 ($C_q$), 143.5 ($C_q$), 140.3 ($C_q$), 138.8 ($C_q$), 138.6 (CH), 138.5 ($C_q$), 136.7 ($C_q$), 131.8 ($C_q$), 130.5 (CH), 128.2 (CH), 127.9 (CH), 125.8 (CH), 125.2 (CH), 123.7 ($C_q$), 123.6 (CH), 123.5 (CH), 122.7 (CH), 121.0 (CH), 119.7 (CH), 119.3 (CH), 116.0 (CH), 114.5 (CH), 112.4 (CH), 84.5 ($C_q$), 68.0 ($CH_2$), 56.8 (CH), 54.7 (CH), 43.0 ($C_q$), 37.8 ($CH_2$), 25.9 ($CH_3$), 25.7 ($CH_3$), 25.3 ($CH_2$), 23.5 ($CH_3$), 21.5 ($CH_3$), 16.0 ($CH_3$) ppm.

**IR** (ATR): $\tilde{\nu}$ = 3342, 2959, 2924, 1660, 1598, 1493, 1368, 1265, 1129 $cm^{-1}$.

**HRMS** (ESI): *m/z* $[M+Na]^+$ calcd for $C_{40}H_{40}N_4O_2Na$: 631.3049; found: 631.3059.

**$[\alpha]^{D}_{20}$** = -59.82 (c = 1.12, $CHCl_3$).

**$R_t$** (IA column, *n*-hexane/*i*-PrOH 75/25, 1.0 mL/min, 250.4 nm): tr(major) = 14.1 min, tr(minor) = 8.2 min, 97.5:2.5 e.r.

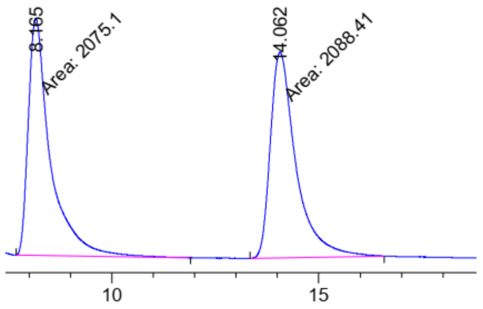


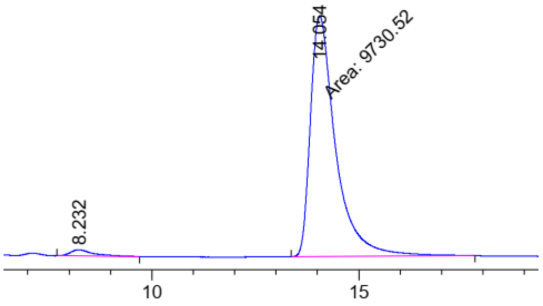


| Peak # | RetTime [min] | Type | Width [min] | Area [mAU*s] | Height [mAU] | Area % |
|---|---|---|---|---|---|---|
| 1 | 8.165 | MM | 0.6221 | 2075.10156 | 55.59595 | 49.8402 |
| 2 | 14.062 | MM | 0.7216 | 2088.40796 | 48.23845 | 50.1598 |

| Peak # | RetTime [min] | Type | Width [min] | Area [mAU*s] | Height [mAU] | Area % |
|---|---|---|---|---|---|---|
| 1 | 8.232 | BB | 0.4480 | 226.65367 | 5.98281 | 2.2763 |
| 2 | 14.054 | MM | 0.7100 | 9730.51953 | 228.42905 | 97.7237 |

**2-((*E*)-((6*bS*,11*R*,11*aR*)-8-(((*E*)-3,7-dimethylocta-2,6-dien-1-yl)oxy)-11-methyl-6b,11a-dihydro-11H-indeno[1,2-*a*]acenaphthylen-11-yl)diazenyl)pyridine (36):**

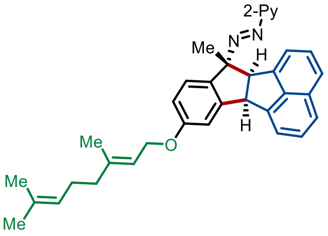


Prepared according to general procedure **GP7** on a 0.3 mmol scale, column chromatography (*n*-hexane/ethyl acetate = 4:1) afforded the title compound as a yellow sticky solid (94 mg, 0.18 mmol, 61%), with an enantiomeric ratio of 98.5:1.5.

**$^{1}$H-NMR** (400 MHz, $CDCl_3$) δ 8.72 – 8.67 (m, 1H), 7.88 – 7.80 (m, 1H), 7.71 – 7.64 (m, 2H), 7.62 – 7.54 (m, 2H), 7.53 – 7.44 (m, 3H), 7.41 – 7.36 (m, 1H), 7.22 (d, *J* = 8.5 Hz, 1H), 7.20 – 7.17 (m, 1H), 6.83 – 6.79 (m, 1H), 5.53 – 5.44 (m, 1H), 5.37 (d, *J* = 7.3 Hz, 1H), 5.30 (d, *J* = 7.3 Hz, 1H), 5.14 – 5.06 (m, 1H), 4.55 (d, *J* = 6.6 Hz, 2H), 2.17 – 2.03 (m, 4H), 1.75 (s, 3H), 1.71 – 1.64 (m, 6H), 1.60 (s, 3H) ppm.

**$^{13}$C-NMR** (101 MHz, $CDCl_3$) δ 162.7 ($C_q$), 159.9 ($C_q$), 149.3 ($C_q$), 146.3 ($C_q$), 146.2 ($C_q$), 143.7 ($C_q$), 141.3 ($C_q$), 138.4 (2xCH), 136.4 ($C_q$), 131.9 ($C_q$), 131.8 ($C_q$), 127.9 (CH), 127.8 (CH), 126.0 (CH), 125.0 (CH), 123.8 (CH), 123.4 (2xCH), 122.6 (CH), 119.5 (CH), 119.0 (CH), 114.5 (CH), 114.4 (CH), 110.3 (CH), 84.7 ($C_q$), 65.1 ($CH_2$), 56.9 (CH), 54.6 (CH), 39.6 (CH), 26.4 ($CH_2$), 25.7 ($CH_2$), 23.4 ($CH_3$), 17.7 ($CH_3$), 16.7 ($CH_3$) ppm.

**IR** (ATR): $\tilde{\nu}$ = 3049, 2970, 1751, 1595, 1490, 1460, 1373, 1280, 1144, 1106, 784 $cm^{-1}$.

**HRMS** (ESI): *m/z* $[M+Na]^+$ calcd for $C_{35}H_{35}N_3ONa$: 536.2672; found: 536.2682.

**$[\alpha]^D_{20}$** = -73.0 (c = 0.5, $CHCl_3$).

**$R_t$** (IA column, *n*-hexane/*i*-PrOH 60/40, 1.0 mL/min, 250.4 nm): tr(major) = 8.0 min, tr(minor) = 5.1 min, 98.5:1.5 e.r.

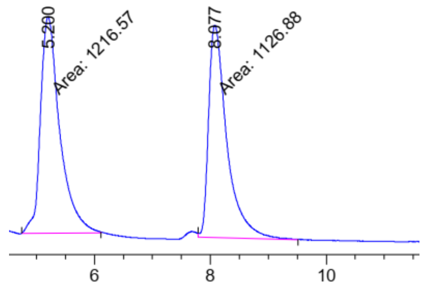


| Peak # | RetTime [min] | Type | Width [min] | Area [mAU*s] | Height [mAU] | Area % |
|---|---|---|---|---|---|---|
| 1 | 5.200 | MM | 0.3869 | 1216.57043 | 52.41175 | 51.9136 |
| 2 | 8.077 | MM | 0.3658 | 1126.88000 | 51.34631 | 48.0864 |

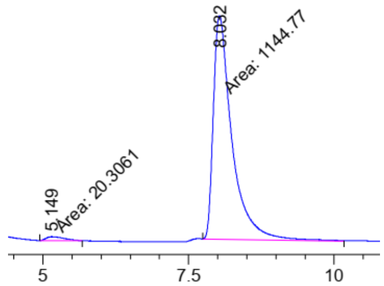


| Peak # | RetTime [min] | Type | Width [min] | Area [mAU*s] | Height [mAU] | Area % |
|---|---|---|---|---|---|---|
| 1 | 5.149 | MM | 0.3558 | 20.30614 | 9.51204e-1 | 1.7429 |
| 2 | 8.032 | MM | 0.3735 | 1144.76855 | 51.08543 | 98.2571 |

**(1*R*,2*S*,5*R*)-2-isopropyl-5-methylcyclohexyl (6*bS*,11*R*,11*aR*)-11-methyl-11-((*E*)-pyridin-2-yldiazenyl)-6b,11a-dihydro-11H-indeno[1,2-a]acenaphthylene-8-carboxylate (37):**

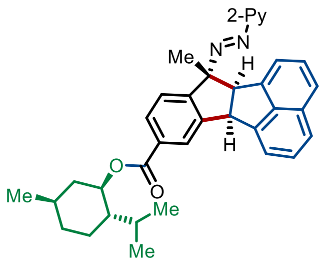


Prepared according to general procedure **GP7** on a 0.3 mmol scale, column chromatography (*n*-hexane/ethyl acetate = 2:1) afforded the title compound as a yellow sticky solid (81 mg, 0.15 mmol, 50%), with an enantiomeric ratio of 89.0:11.0.

**$^{1}$H-NMR** (400 MHz, $CDCl_3$) δ 8.74 – 8.68 (m, 1H), 8.34 – 8.31 (m, 1H), 7.96 – 7.91 (m, 1H), 7.89 – 7.82 (m, 1H), 7.71 – 7.65 (m, 3H), 7.58 – 7.47 (m, 4H), 7.43 – 7.38 (m, 2H), 5.45 (d, *J* = 7.3 Hz, 1H), 5.32 (d, *J* = 7.3 Hz, 1H), 4.99 – 4.89 (m, 1H), 2.15 – 2.06 (m, 1H), 1.98 – 1.87 (m, 1H), 1.79 – 1.69 (m, 5H), 1.62 – 1.51 (m, 3H), 1.19 – 1.08 (m, 2H), 0.95 – 0.89 (m, 6H), 0.77 (d, *J* = 6.9 Hz, 3H) ppm.

**$^{13}$C-NMR** (101 MHz, $CDCl_3$) δ 166.0 ($C_q$), 162.6 ($C_q$), 149.5 ($C_q$), 149.5 (CH), 145.7 ($C_q$), 145.1 ($C_q$), 143.2 ($C_q$), 138.6 (CH), 138.5 ($C_q$), 131.9 ($C_q$), 131.8 ($C_q$), 129.2 (CH), 128.2 (CH), 128.0 (CH), 126.0 (CH), 125.4 (CH), 123.7 (CH), 123.7 (CH), 122.8 (CH), 119.4 (CH), 114.8 (CH), 85.3 ($C_q$), 75.0 ($CH_3$), 56.8 (CH), 54.5 (CH), 47.4 ($CH_3$), 41.1 ($CH_2$), 34.5 ($CH_2$), 31.6 ($CH_3$), 26.6 ($CH_3$), 23.8 ($CH_2$), 23.6 ($CH_3$), 22.2 ($CH_3$), 20.9 ($CH_3$), 16.6 ($CH_3$) ppm..

**IR** (ATR): $\tilde{\nu}$ = 2995, 2928, 1710, 1584, 1459, 1425, 1251, 1099, 783 $cm^{-1}$.

**HRMS** (ESI): *m/z* $[M+H]^+$ calcd for $C_{36}H_{38}N_3O_2$: 544.2959; found: 544.2954.

**$[\alpha]^D_{20}$** = -73.0 (c = 0.5, $CHCl_3$).

**$R_t$** (IA column, *n*-hexane/*i*-PrOH 60/40, 1.0 mL/min, 250.4 nm): tr(major) = 20.0 min, tr(minor) = 7.1 min, 89.0:11.0 e.r.

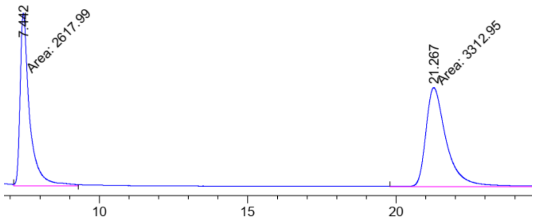


| Peak # | RetTime [min] | Type | Width [min] | Area [mAU*s] | Height [mAU] | Area % |
|---|---|---|---|---|---|---|
| 1 | 7.442 | MM | 0.3695 | 2617.99219 | 118.08688 | 44.1412 |
| 2 | 21.267 | MM | 0.8152 | 3312.95166 | 67.73058 | 55.8588 |

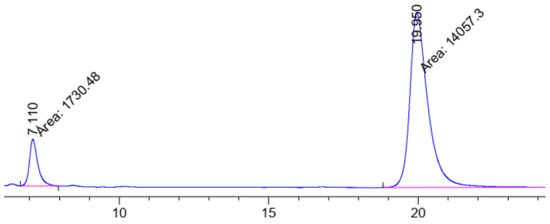


| Peak # | RetTime [min] | Type | Width [min] | Area [mAU*s] | Height [mAU] | Area % |
|---|---|---|---|---|---|---|
| 1 | 7.110 | MM | 0.3265 | 1730.47754 | 88.34578 | 10.9609 |
| 2 | 19.950 | MM | 0.7075 | 1.40573e4 | 331.13791 | 89.0391 |

## Derivatization of the Products

### General Procedure for the Reduction of the Azo Compound 5 to Amine 38 (GP10)

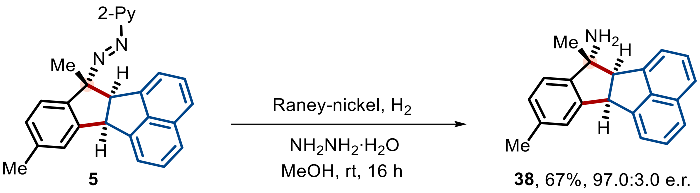


**5** **38**, 67%, 97.0:3.0 e.r.

The chiral azo compound **5** was derivatized to amine **38** following the following procedure. First, in a 100 mL round bottom flask charged with magnetic stir bar the compound **5** (1.0 mmol, 1.0 equiv) was added and the flask was evacuated and backfilled with $N_2$ gas. Next MeOH (25 mL) was added into round bottom flask followed by the addition of aqueous Raney-Ni (~10% w/w) under nitrogen. Then flask was kept under an ice bath and the resulting solution was bubbled with Hydrogen gas for 15 minutes to saturate the flask with hydrogen gas. Following this, the bubbling was stopped and the reaction flask was maintained under a $H_2$ atmosphere using a hydrogen balloon. Further, 6 mL of hydrazine hydrate was added to the reaction mixture and the mixture was stirred at room temperature for 16 h. The reaction was then quenched with basic NaOH solution (2 M) and the product was extracted through a workup with ethyl acetate. The extracted solvent was dried over $MgSO_4$ and concentrated through rotary evaporator. The resultant crude was purified through column chromatography with Acetone/*n*-hexane (10:1) to obtain the desired amine **38** (67% yield, 191 mg).

**$^1$H-NMR** (400 MHz, $CDCl_3$) δ 7.67 – 7.46 (m, 6H), 7.43 – 7.39 (m, 1H), 7.15 (d, *J* = 7.8 Hz, 1H), 7.09 – 7.05 (m, 1H), 5.30 (d, *J* = 7.3 Hz, 1H), 4.41 (d, *J* = 7.3 Hz, 1H), 2.38 (s, 3H), 1.32 (s, 3H) ppm.

**$^{13}$C-NMR** (101 MHz, $CDCl_3$) δ 148.1 ($C_q$), 146.2 ($C_q$), 144.2 ($C_q$), 142.5 ($C_q$), 138.2 ($C_q$), 138.0 ($C_q$), 131.8 ($C_q$), 128.9 (CH), 128.0 (CH), 127.8 (CH), 125.1 (CH), 123.5 (CH), 123.3 (CH), 122.7 (CH), 122.2 (CH), 119.0 (CH), 63.7 (CH), 63.5 ($C_q$), 53.9 (CH), 28.1 ($CH_3$), 21.5 ($CH_3$) ppm.

**IR** (ATR): $\tilde{\nu}$ = 3041, 2921, 2034, 1598, 1491, 1449, 1370, 823, 785 cm$^{-1}$.

**HRMS** (ESI): *m/z* $[M+H]^+$ calcd for $C_{21}H_{20}N$: 286.1589; found: 286.1590.

**$[\alpha]^D_{20}$** = - 219.9 (c = 2.0, $CHCl_3$).

**$R_t$** (ID column, *n*-hexane/*i*-PrOH 90/10, 1.0 mL/min, 250.4 nm): tr(major) = 8.8 min, tr(minor) = 11.5 min, 97.0:3.0 e.r.

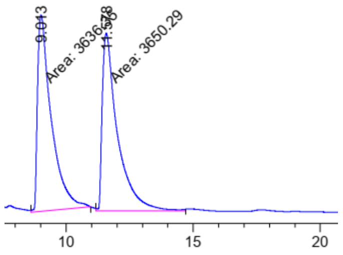


| Peak # | RetTime [min] | Type | Width [min] | Area [mAU*s] | Height [mAU] | Area % |
|---|---|---|---|---|---|---|
| 1 | 9.013 | MM | 0.6290 | 3636.34570 | 96.34789 | 49.9043 |
| 2 | 11.578 | MM | 0.6982 | 3650.28979 | 87.13218 | 50.0957 |

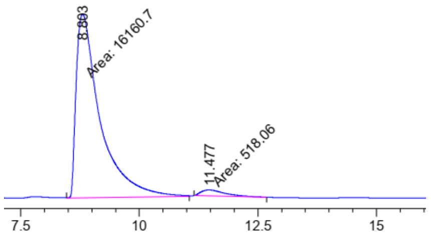


| Peak # | RetTime [min] | Type | Width [min] | Area [mAU*s] | Height [mAU] | Area % |
|---|---|---|---|---|---|---|
| 1 | 8.803 | MM | 0.5853 | 1.61607e4 | 460.20831 | 96.8939 |
| 2 | 11.477 | MM | 0.5959 | 518.05969 | 14.49068 | 3.1061 |

## General Procedure for the Synthesis of Urea 39 (GP11)

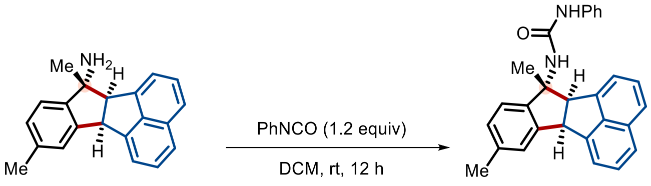


**38**, 67%, 97.0:3.0 e.r. **39**, 65%, 97.5:2.5 e.r.

The chiral amine **38** was derivatized to urea **39** following the following procedure. First, in a 25 mL round bottom flask charged with magnetic stir bar, the compound **38** (0.1 mmol, 1.0 equiv) was added followed by the addition of dichloromethane (2.5 mL). Next, PhNCO (1.2 equiv) was added to the flask, stirring the reaction mixture overnight at room temperature. Then the solvent was evaporated to dryness and the crude mixture was purified through column chromatography with gradient eluent hexane/ethyl acetate (5:1). The desired urea **39** was obtained in 65% yield (26 mg).

**$^1$H-NMR** (300 MHz, Acetone-d$_6$) δ 7.98 – 7.88 (m, 2H), 7.74 – 7.63 (m, 3H), 7.61 – 7.47 (m, 5H), 7.31 – 7.18 (m, 3H), 7.13 – 7.05 (m, 1H), 7.01 – 6.88 (m, 1H), 6.28 (s, 1H), 5.36 (d, *J* = 7.7 Hz, 1H), 5.07 (d, *J* = 7.7 Hz, 1H), 2.37 (s, 3H), 1.44 (s, 3H) ppm.

**$^{13}$C-NMR** (101 MHz, Acetone-d$_6$) δ 154.7 (C$_q$), 145.8 (C$_q$), 144.5 (C$_q$), 143.9 (C$_q$), 143.8 (C$_q$), 139.2 (C$_q$), 138.8 (C$_q$), 138.3 (C$_q$), 131.7 (C$_q$), 129.3 (CH), 129.2 (CH), 128.0 (CH), 127.9 (CH), 125.3 (CH), 123.7 (CH), 123.5 (CH), 123.4 (CH), 123.4 (CH), 123.1 (CH), 120.6 (CH), 118.9 (CH), 65.4 (C$_q$), 60.3 (CH), 54.7 (CH), 27.2 (CH$_3$), 21.6 (CH$_3$) ppm.

**IR** (ATR): $\tilde{\nu}$ = 3340, 3042, 1646, 1598, 1546, 1496, 1240, 753 cm$^{-1}$.

**HRMS** (ESI): *m/z* [M+Na]$^+$ calcd for $C_{28}H_{24}N_2ONa$: 427.1778; found: 427.1781.

**Melting point**: 158–160 °C

**$[\alpha]^D_{20}$** = -199.8 (c = 1.0, Acetone).

**R$_t$** (ID column, *n*-hexane/*i*-PrOH 98/2, 1.5 mL/min, 250.4 nm): tr(major) = 21.4 min, tr(minor) = 27.0 min, 97.0:3.0 e.r.

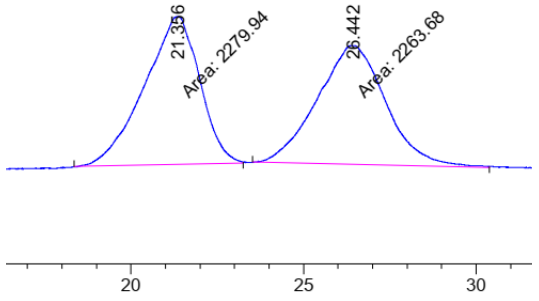


| Peak # | RetTime [min] | Type | Width [min] | Area [mAU*s] | Height [mAU] | Area % |
|---|---|---|---|---|---|---|
| 1 | 21.356 | MM | 1.8603 | 2279.93823 | 20.42672 | 50.1789 |
| 2 | 26.442 | MM | 2.2994 | 2263.68140 | 16.40750 | 49.8211 |

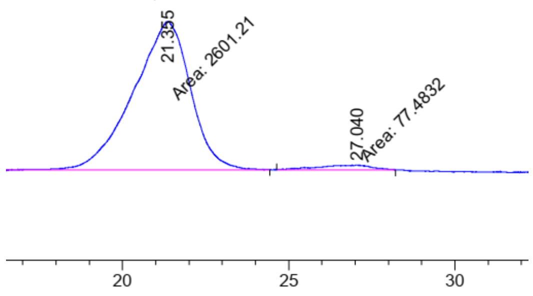


| Peak # | RetTime [min] | Type | Width [min] | Area [mAU*s] | Height [mAU] | Area % |
|---|---|---|---|---|---|---|
| 1 | 21.355 | MM | 1.9982 | 2601.20801 | 21.69648 | 97.1074 |
| 2 | 27.040 | MM | 1.8977 | 77.48315 | 6.80494e-1 | 2.8926 |

## Miscellaneous Studies

### Qualitative Analysis of Electrochemical Oxygen Reduction during the Catalysis

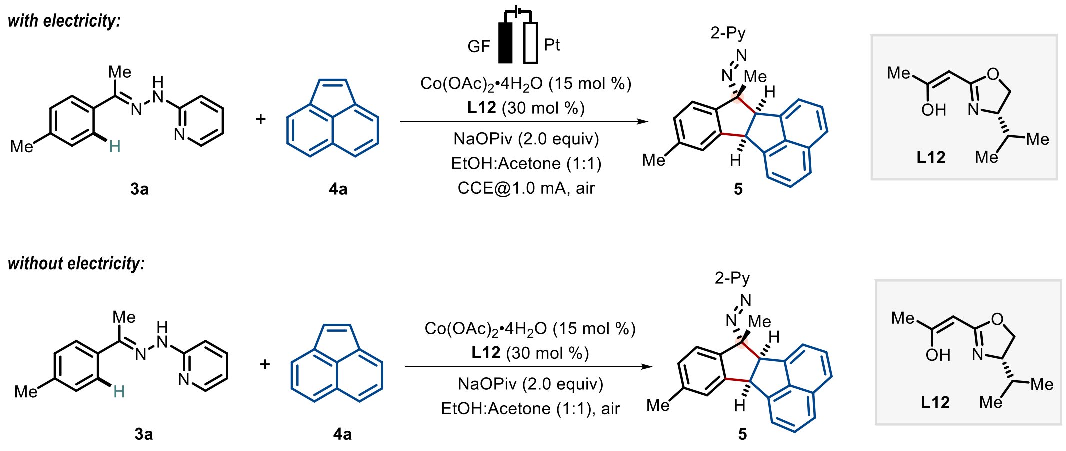


To analyze the possible influence of electrochemical oxygen reduction during the catalysis, six separate reactions were carried out under the standard reaction conditions – with and without electricity. In all the cases, a septum was used instead of the Teflon cap to ensure the proper analysis. Individual reactions were studied separately after 2 h, 4 h, and 6 h by taking 1 mL gas volume from the headspace of the reaction flask with a gas syringe and analyzed by headspace-GC. Comparative analysis suggested a reasonable oxygen reduction under electrochemical conditions.

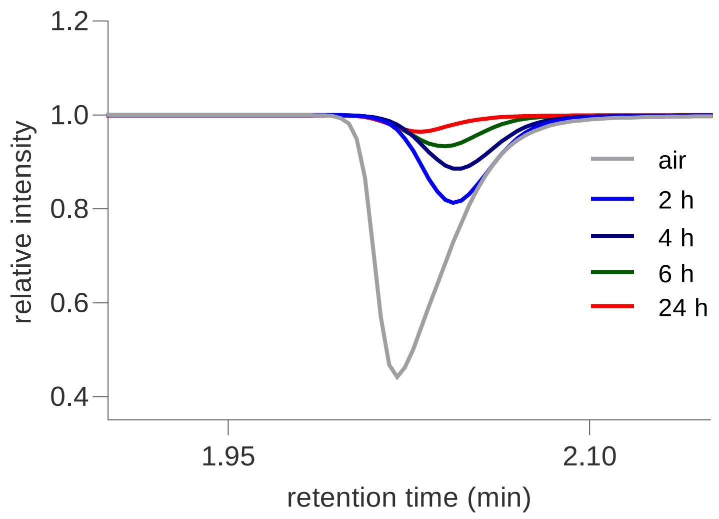


**Figure S4.** Headspace GC analysis under electrochemical conditions.

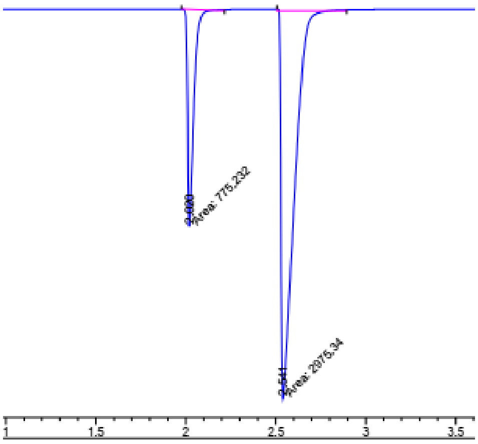


| Peak # | RetTime [min] | Type | Width [min] | Area [25 µV*s] | Height [25 µV] | Area % |
|---|---|---|---|---|---|---|
| 1 | 2.020 | MM N | 0.0359 | 775.23242 | 359.89777 | 20.66972 |
| 2 | 2.541 | MM N | 0.0773 | 2975.33740 | 641.54010 | 79.33028 |

Standard headspace GC analysis of air

Headspace GC analysis of oxygen consumption after 2 h, 4 h, and 6 h – **aerial oxygen** was consumed

| Time (h) | under air without electricity | under air with electricity |
|---|---|---|
| 2 h | see below | see below |

**2 h — under air without electricity**

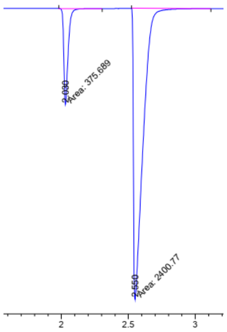


| Peak # | RetTime [min] | Type | Width [min] | Area [25 µV*s] | Height [25 µV] | Area % |
|---|---|---|---|---|---|---|
| 1 | 2.030 | MM N | 0.0349 | 375.68939 | 179.20934 | 13.53124 |
| 2 | 2.550 | MM N | 0.0738 | 2400.76953 | 541.89142 | 86.46876 |

**2 h — under air with electricity**

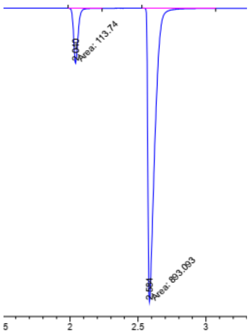


| Peak # | RetTime [min] | Type | Width [min] | Area [25 µV*s] | Height [25 µV] | Area % |
|---|---|---|---|---|---|---|
| 1 | 2.040 | MM N | 0.0371 | 113.74018 | 51.05330 | 11.29682 |
| 2 | 2.584 | MM N | 0.0546 | 893.09344 | 272.76385 | 88.70318 |

**4 h — under air without electricity**

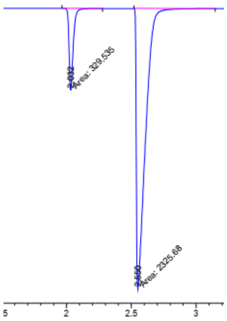


| Peak # | RetTime [min] | Type | Width [min] | Area [25 µV*s] | Height [25 µV] | Area % |
|---|---|---|---|---|---|---|
| 1 | 2.032 | MM N | 0.0354 | 329.53482 | 155.30399 | 12.41087 |
| 2 | 2.550 | MM N | 0.0730 | 2325.67554 | 531.02002 | 87.58913 |

**4 h — under air with electricity**

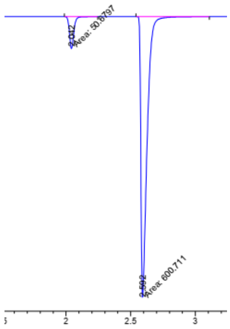


| Peak # | RetTime [min] | Type | Width [min] | Area [25 µV*s] | Height [25 µV] | Area % |
|---|---|---|---|---|---|---|
| 1 | 2.042 | MM N | 0.0376 | 50.67975 | 22.47148 | 7.78024 |
| 2 | 2.592 | MM N | 0.0505 | 600.71063 | 198.31223 | 92.21976 |

6 h

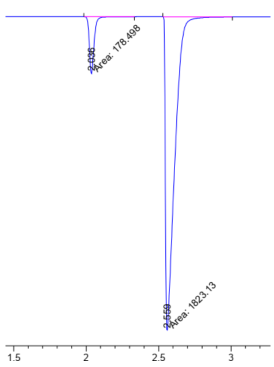

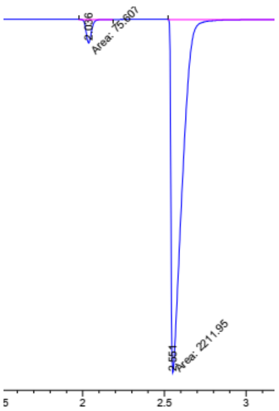

| Peak # | RetTime [min] | Type | Width [min] | Area [25 µV*s] | Height [25 µV] | Area % |
|---|---|---|---|---|---|---|
| 1 | 2.036 | MM N | 0.0360 | 178.49794 | 82.59914 | 8.91764 |
| 2 | 2.559 | MM N | 0.0670 | 1823.13074 | 453.81204 | 91.08236 |

| Peak # | RetTime [min] | Type | Width [min] | Area [25 µV*s] | Height [25 µV] | Area % |
|---|---|---|---|---|---|---|
| 1 | 2.036 | MM N | 0.0367 | 75.60698 | 34.32688 | 3.30515 |
| 2 | 2.551 | MM N | 0.0713 | 2211.94678 | 517.36127 | 96.69485 |

After 20 h - aerial oxygen was almost fully consumed and hydrogen gas was detected

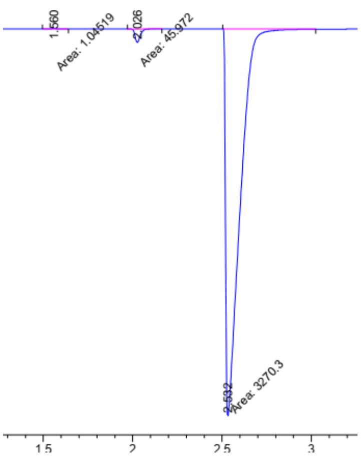

| Peak # | RetTime [min] | Type | Width [min] | Area [25 µV*s] | Height [25 µV] | Area % |
|---|---|---|---|---|---|---|
| 1 | 1.560 | MM | 0.0390 | 1.04519 | 4.46907e-1 | 0.03151 |
| 2 | 2.026 | MM N | 0.0332 | 45.97197 | 23.06493 | 1.38582 |
| 3 | 2.532 | MM N | 0.0826 | 3270.30029 | 659.76971 | 98.58268 |

**Figure S5.** Headspace GC analysis of the reaction under electrochemical conditions.

To further support the role of electricity, the reaction was performed under an oxygen atmosphere and in this case, a lower yield of the product was found supporting the crucial role of electricity in this transformation.

*without electricity:*

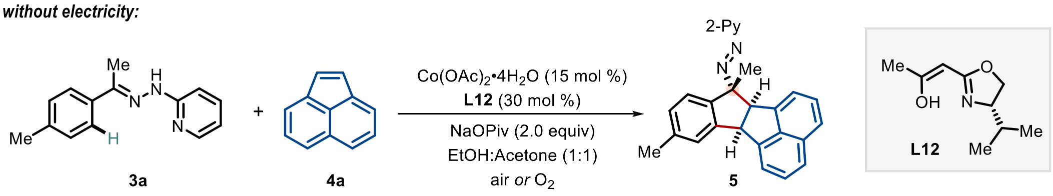


**Table S3.** Reaction under oxygen.

| deviation from the conditions | results |
|---|---|
| same, oxygen balloon | 38%, 97.0:3.0 e.r. |
| EtOH as the solvent, $O_2$ atmosphere | 38%, 96.0:4.0 e.r. |
| same, only air | 35%, 97.0:3.0 e.r. |
| same, 50 °C, air | 36%, 97.0:3.0 e.r. |

## Qualitative Analysis of Electrochemical Oxygen Reduction during the Catalysis

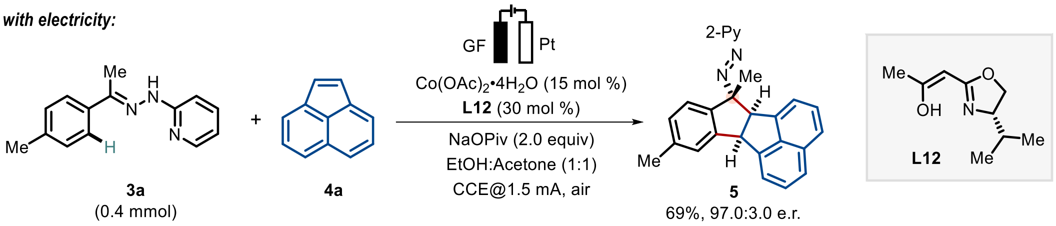


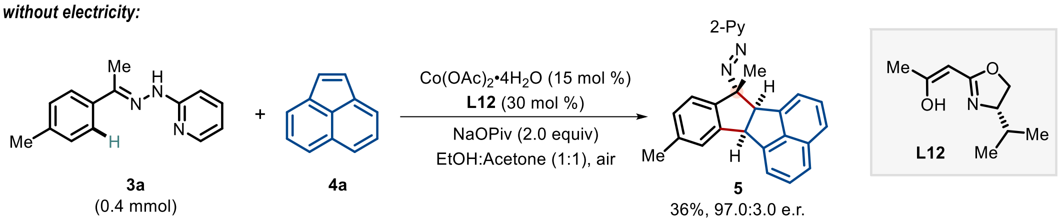


To quantify the amount of oxygen gas consumed during the catalysis, two reactions were carried out under standard reaction conditions - with and without electricity. During the progress of the reactions the side-arms of the Schlenk tubes were connected to the GasMess Instrument. A septum was used instead of the teflon cap to ensure a tightly closed reaction system. For 21 h reaction time, the volume of the consumed gas was analyzed for both the reactions using the GasMess instrument.

### Reaction under air- Oxygen Gas Consumption Studies

Under air, with current (**9.3 mL**)

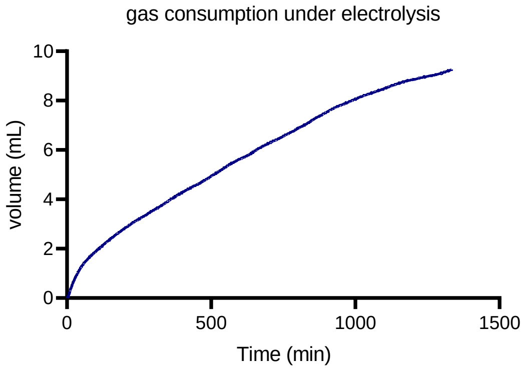


**Figure S6.** Quantitative analysis of oxygen gas consumption under electricity.

Under air, without current (**4.4 mL**)

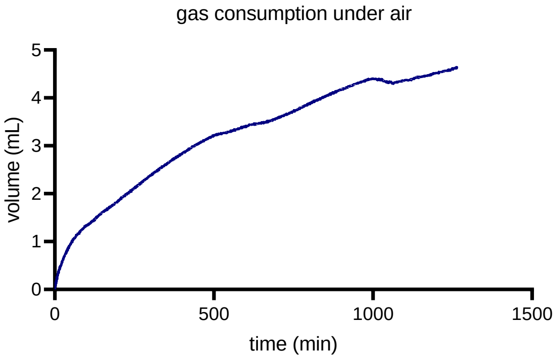


**Figure S7.** Quantitative analysis of oxygen gas consumption without electricity.

## Qualitative Detection of Molecular Dihydrogen as the Byproduct of Catalysis

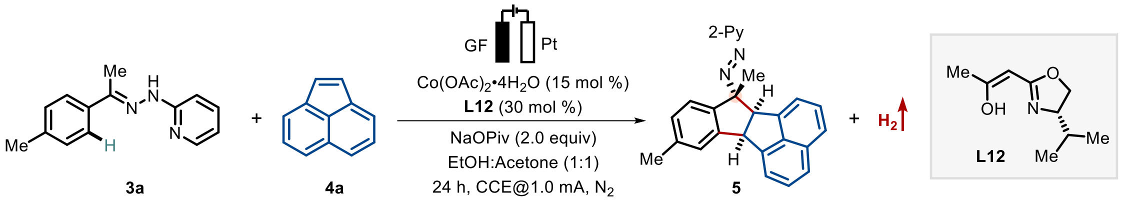


To detect the hydrogen gas during the catalysis, the reaction was carried out under the standard reaction conditions under a nitrogen atmosphere. A septum was used instead of a Teflon cap to ensure the hydrogen formed did not escape. After 7 h reaction time, 1 mL gas volume was taken from the headspace of the reaction flask with a gas syringe and analyzed by headspace-GC.

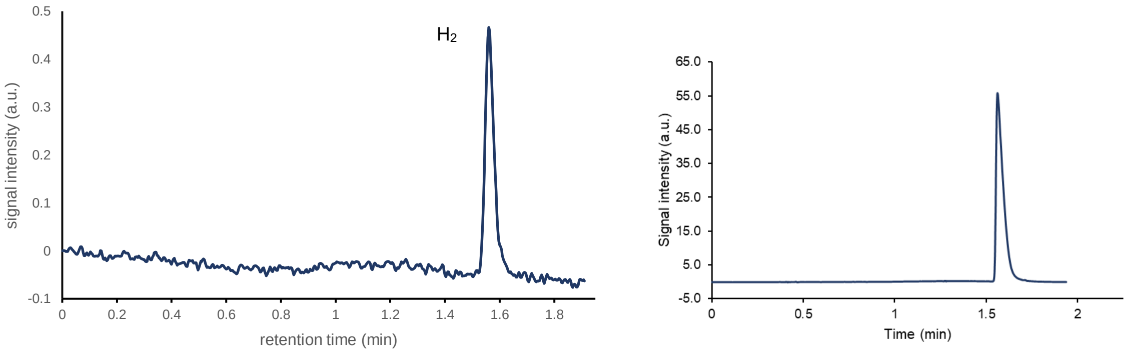


**Figure S8.** Chromatogram detected *via* headspace GC analysis for enantioselective cobalta-electrocatalyzed C–H activation (left) and chromatogram of a pure $H_2$ sample as reference (right).

**Quantitative Detection of Molecular Dihydrogen as the Byproduct of Catalysis**

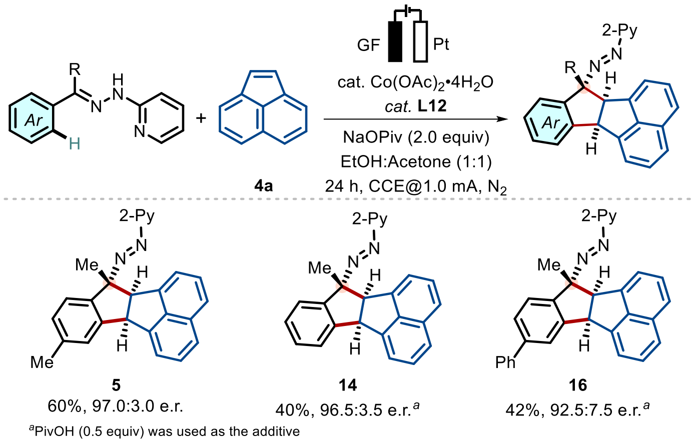


In order to quantify the hydrogen formed during the catalysis, the reaction was carried out under the general conditions under an inert atmosphere. A septum was used instead of the teflon cap to ensure the hydrogen formed did not escape. For 24 h reaction time, the volume of the gas was analyzed using the GasMess instrument for the synthesis of **5**. Analysis of the reaction mixture provided 60% yield with 97.0:3.0 e.r. of the product.

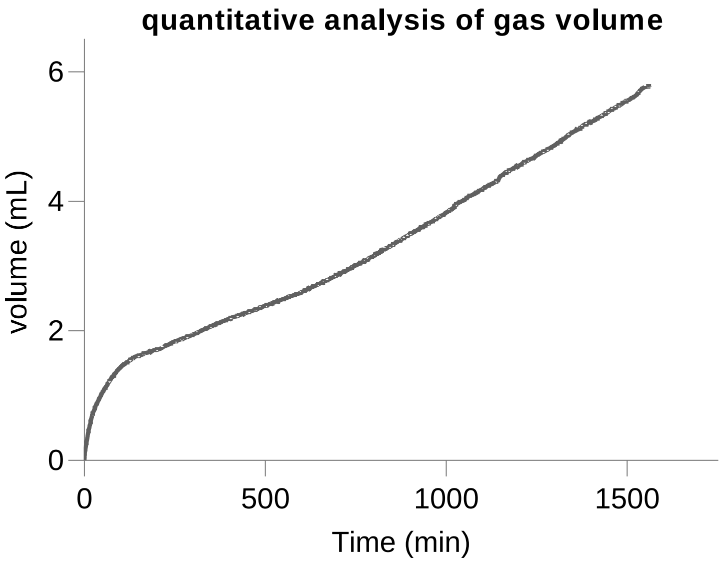


**Figure S9.** Quantification of evolved gas through GasMess instrument.

The $H_2$ to product ratio $n(H_2)/n$(product **5**) was calculated from the determined volume change as follows:

$$\frac{n(\mathrm{H_2})}{n(\text{product } \mathbf{5})} = \frac{p \cdot V(\mathrm{H_2})}{R \cdot T \cdot n(\text{product } \mathbf{5})} = \frac{98520\ Pa \cdot 5.365 \cdot 10^{-6}\ m^3}{8.3145\ J{\cdot}mol^{-1} \cdot K^{-1} \cdot 298.15\ \mathrm{K} \cdot 0.3 \cdot 10^{-3}\ mol \cdot 0.6}$$

$$= 0.43$$

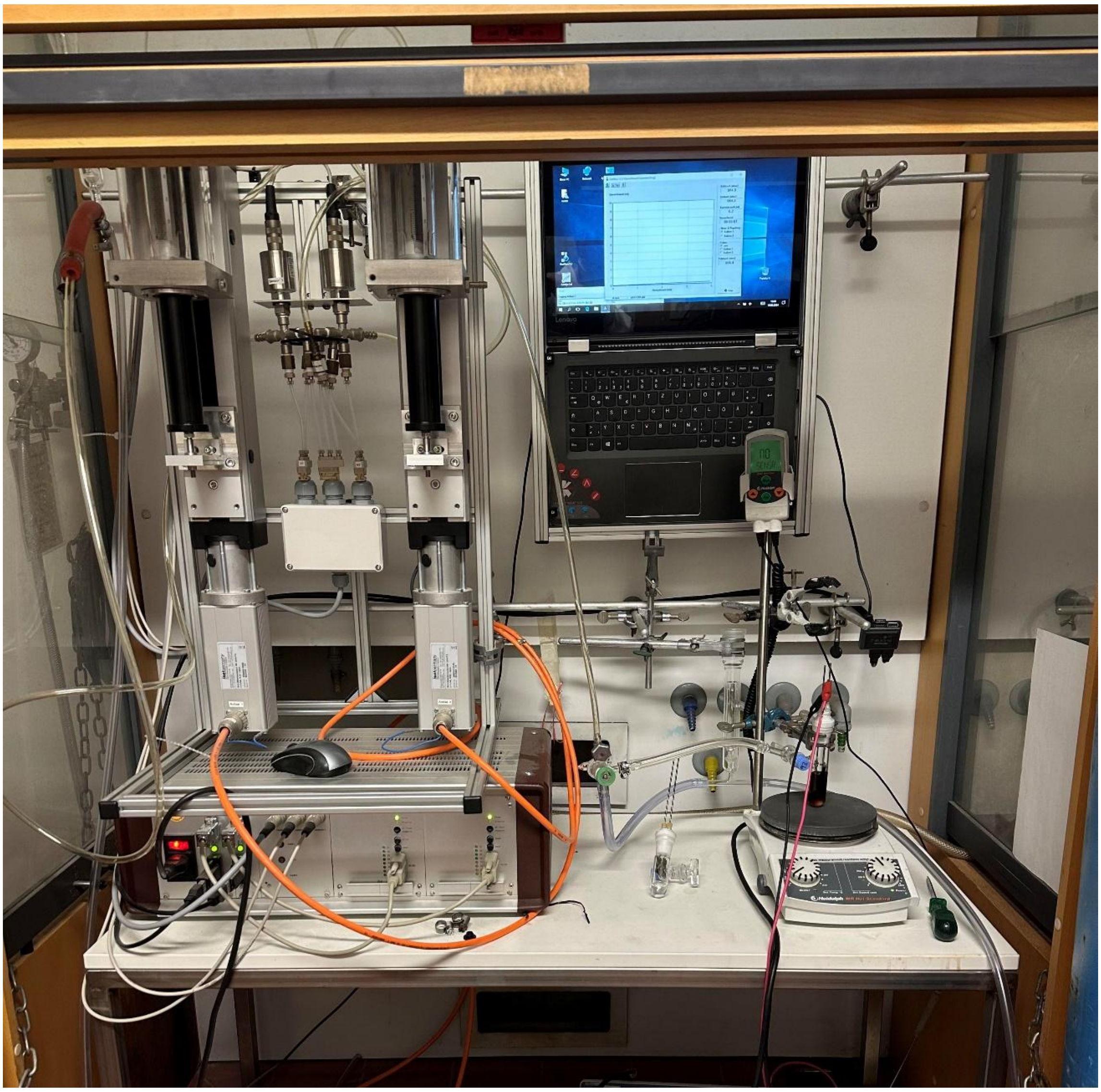

**Figure S10.** Instrumental set up for the quantification of evolved gas through GasMess instrument.

## Monitoring by Mass Spectrometry

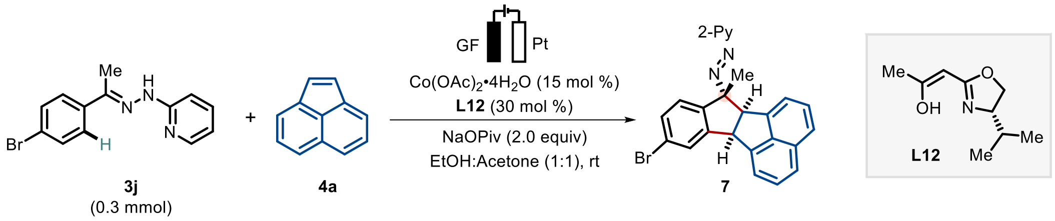


Electrocatalysis was performed according to the general procedure using hydrazone **3j**. An aliquot (50 µL) was taken 240 minutes reaction time. The aliquot was diluted with EtOH (500 µL), filtered through a HPLC filter, and directly analyzed via HR ESI MS.

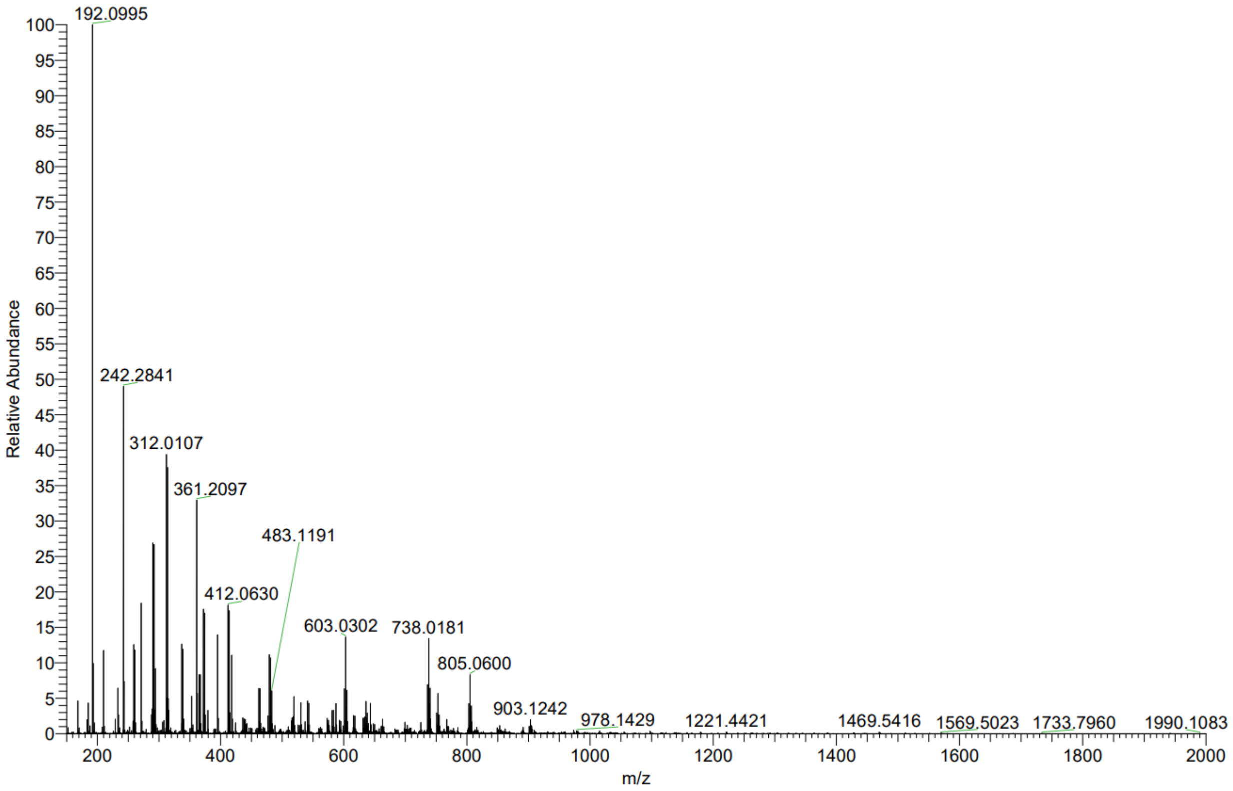


**Figure S11.** HR-ESI-mass spectrometric monitoring of the enantioselective electrochemical cobalt-catalyzed C–H activation with total m/z range.

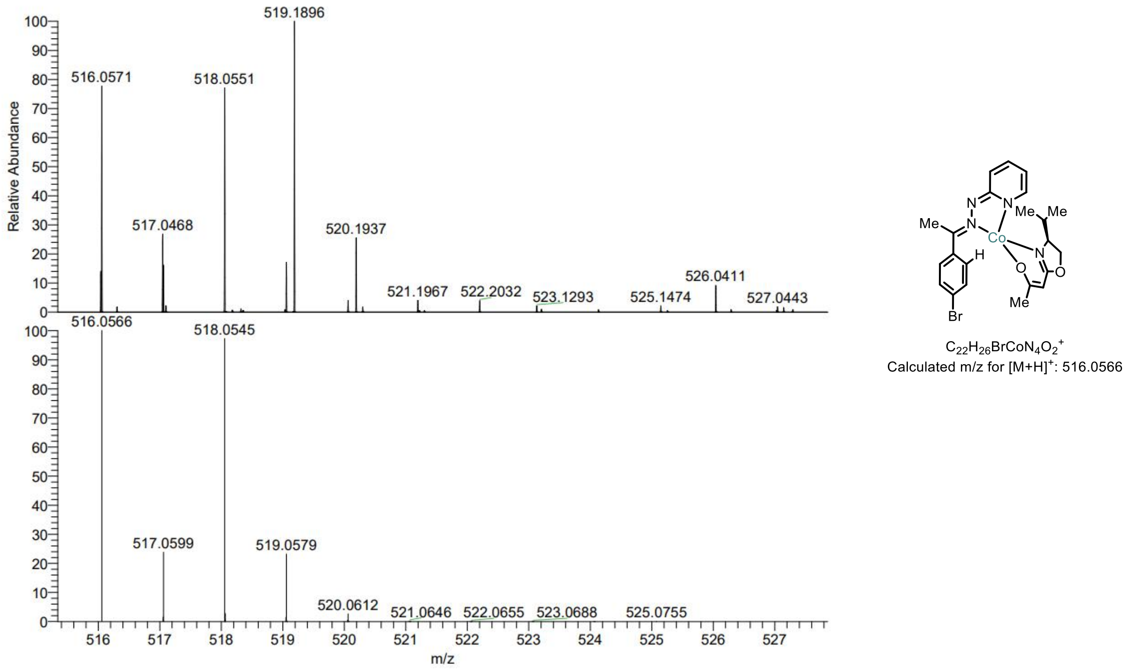


**Figure S12.** Comparison of measured and simulated isotope pattern. m/z calculated for $[C_{22}H_{26}Br_1Co_1N_4O_2]^+$ = 516.0566.

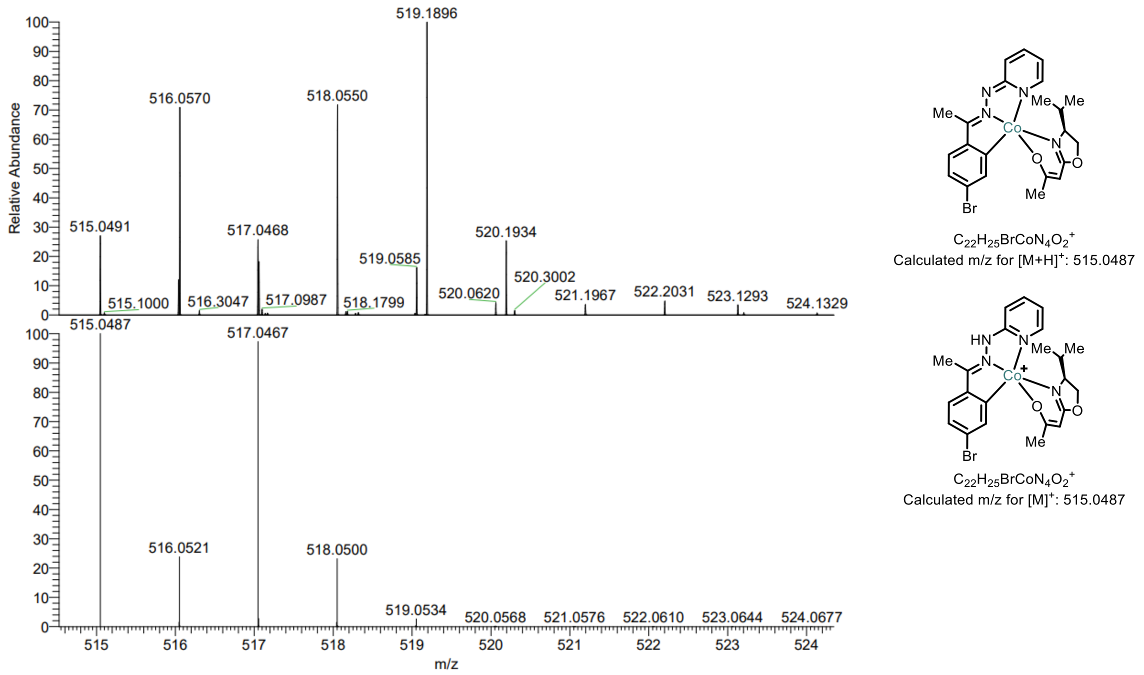


**Figure S13.** Comparison of measured and simulated isotope pattern. m/z calculated for $[C_{22}H_{25}Br_1Co_1N_4O_2]^+$ = 515.0487.

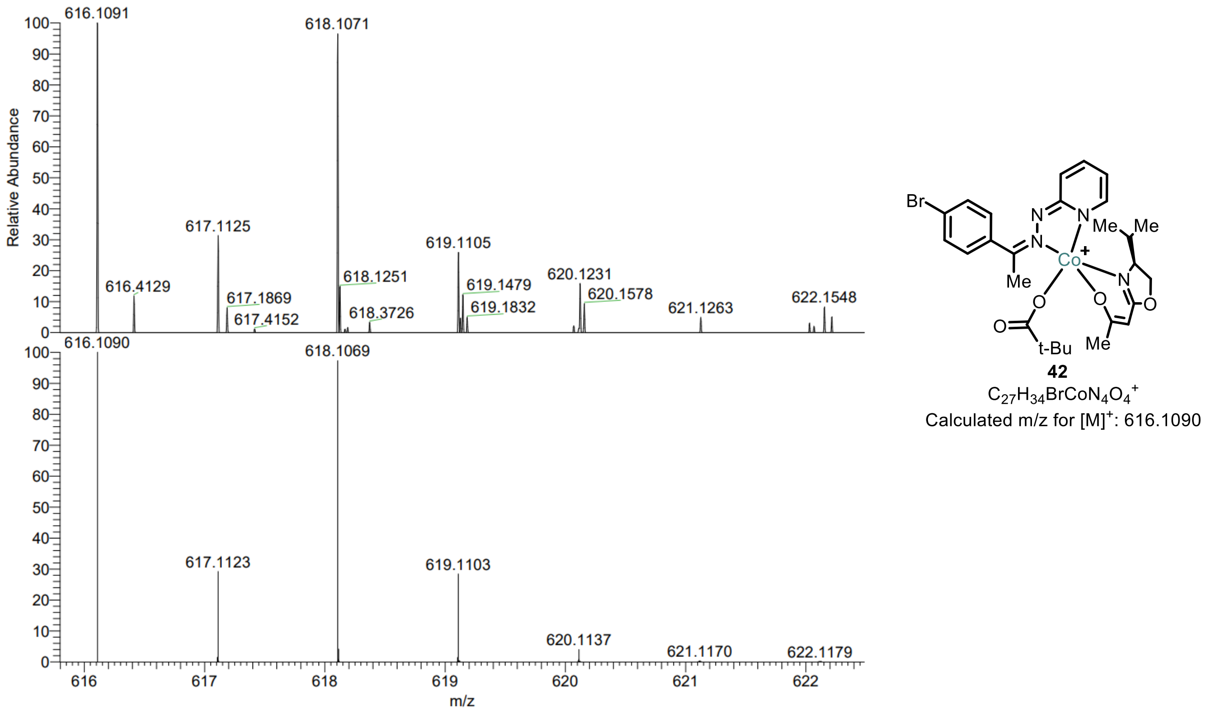


**Figure S14.** Comparison of measured and simulated isotope patterns. m/z calculated for $[C_{27}H_{34}Br_1Co_1N_4O_4]^+ = 616.1090$.

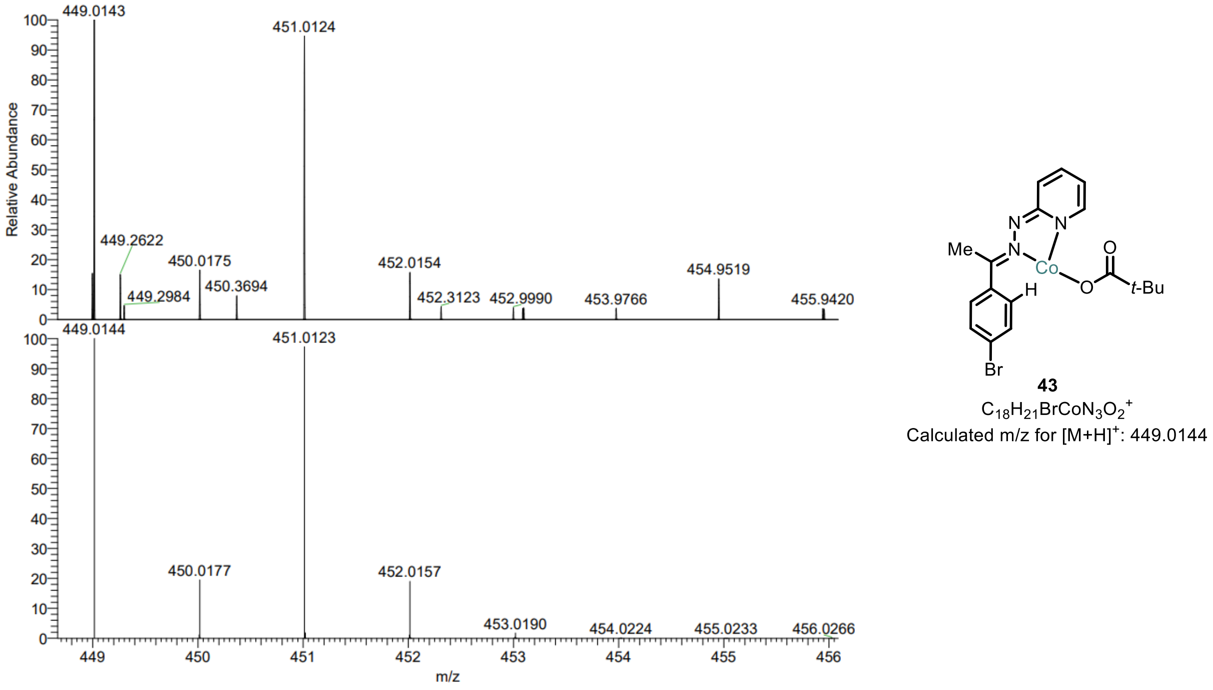


**Figure S15.** Comparison of measured and simulated isotope pattern. m/z calculated for $[C_{18}H_{21}Br_1Co_1N_3O_2]^+ = 449.0144$.

**Crystallographic data of 38**

**Crystallization Method:** The crystallization of the sample was performed in Acetone:Hexane (1:5) solution through diffusion method. First compound **38** (10 mg) was dissolved in the solution and hexane was used as the anti-solvent. Then the sample was stored in -20 ºC for 3 days. Then the sample was kept at ambient temperature until the crystals were obtained.

**General Data Acquisition and Processing**

The diffraction data were collected using Mo Kα radiation and a Bruker Photon III C7 Detector. The data were integrated with SAINT.[6] A multi-scan absorption correction was applied using SADABS.[7] The structure was solved by SHELXT[8] and refined on $F^2$ using SHELXL[9] in the graphical user interface ShelXle.[10]

**Table S4.** Crystal data and structure refinement for compound **38**

| | |
|---|---|
| Compound | **38** |
| CCDC | 2369195 |
| Empirical Formula | $C_{28}H_{24}N_2O$ |
| Formula weight | 404.49 |
| $T$ [K] | 100(2) |
| $\lambda$ [Å] | 0.71073 |
| Crystal system | Orthorhombic |
| Space group | $P2_12_12_1$ |
| $a$ [Å] | 12.292(2) |
| $b$ [Å] | 16.887(2) |
| $c$ [Å] | 20.779(3) |
| $V$ [Å$^3$] | 4313.2(11) |
| $Z$ | 8 |
| $\mu$ [mm$^{-1}$] | 0.076 |
| $F$(000) | 1712 |
| Crystal size [mm] | 0.56 x 0.224 x 0.086 |
| $\Theta$ max [°] | 1.960 to 30.533 |
| Reflections collected | 453308 |
| Independent reflections | 13185 |
| $R_{int}$ | 0.0447 |
| Data/restraints/parameters | 13185 / 297 / 628 |
| GooF | 1.061 |
| $R1$ [$I > 2\sigma(I)$] | 0.0333 |
| $wR2$ [all data] | 0.0904 |
| Absolute structure parameter[6] | -0.13(14) |
| $\rho_{max}/\rho_{min}$ [e A$^{-3}$] | 0.269/-0.218 |
| Shape and color | colorless blocks |

$^aR1 = \Sigma||F_o| - |F_c||/\Sigma|F_o|$. $^bwR_2 = [\Sigma w(F_o^2 - F_c^2)^2/\Sigma(F_o^2)^2]^{1/2}$

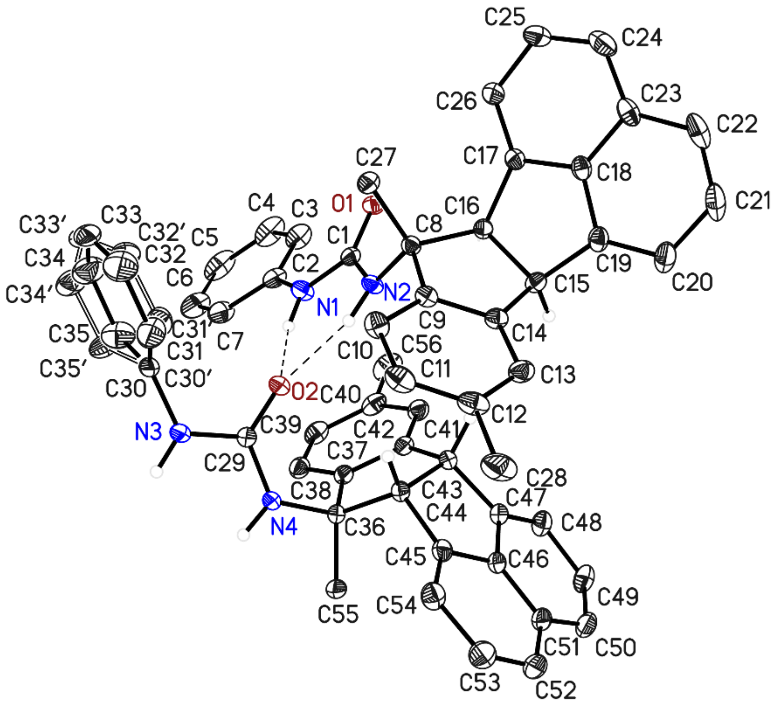


**Figure S16.** Asymmetric unit of **38**.

All hydrogen atoms bonded to carbon atoms were placed according to geometrical criteria and refined with a riding model. Hydrogen atoms bond to nitrogen atoms were refined freely.
One phenyl ring was disordered over two positions. It was refined with distance restraints and restraints for the anisotropic displacement parameters. The occupancy of the minor position was refined to 0.49(3).
The structure crystallizes in space group $P2_12_12_1$ with two molecules in the asymmetric unit. Both molecules show the same absolute configuration (see Figure S17 and S18). The absolute structure was confirmed by the Flack parameter of -0.13(14).[11] Both molecules differ in their conformation (see Figure S19). The molecules are connected by hydrogen bonds (see Figure S20 and Table S5). Bond lengths and angles are listed in Table S6.

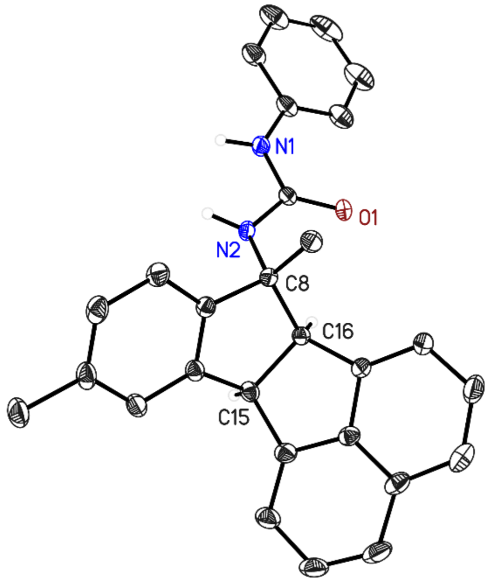


**Figure S17.** Structure of molecule 1

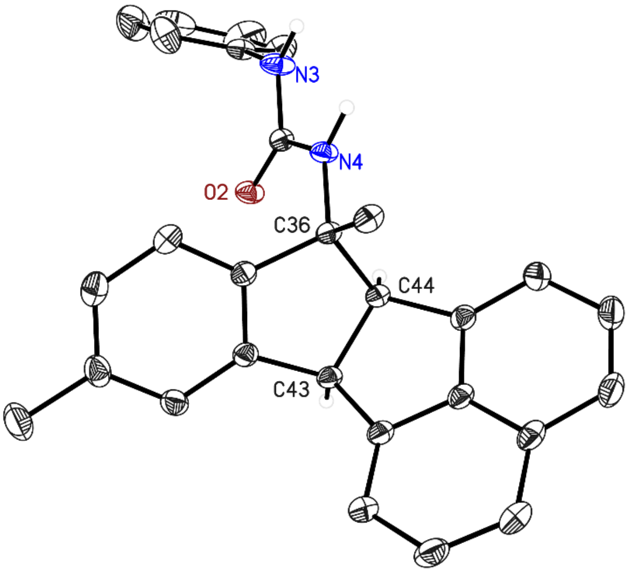


**Figure S18.** Structure of molecule 2.

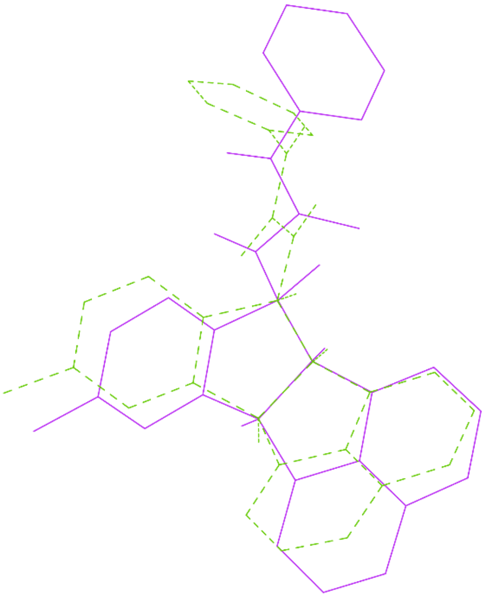

**Figure S19.** Overlay of both molecules.

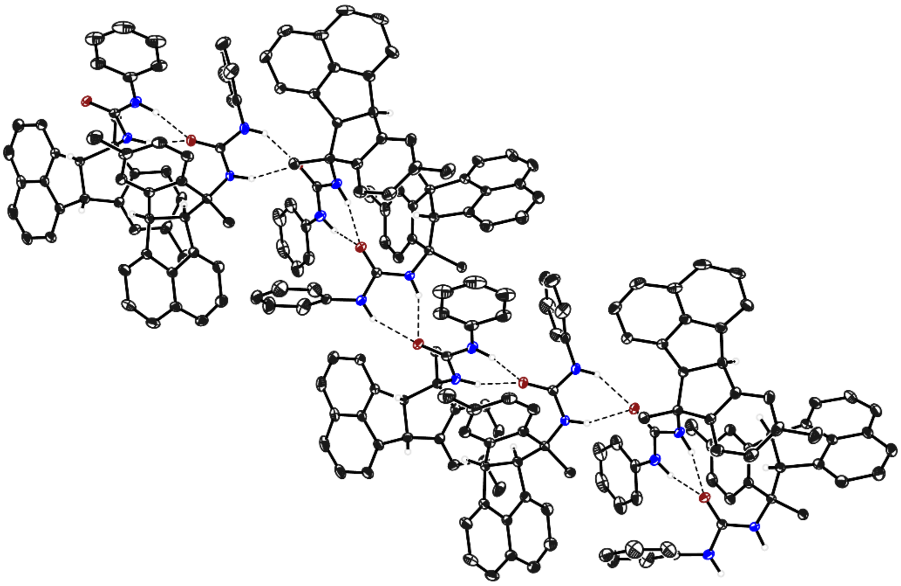

**Figure S20.** Hydrogen bonding in **38**.

**Table S5.** Hydrogen bonds for **38** [Å and °].

| D-H...A | d(D-H) | d(H...A) | d(D...A) | <(DHA) |
|---|---|---|---|---|
| N(1)-H(1)...O(2) | 0.899(17) | 2.029(18) | 2.8592(16) | 152.9(19) |
| N(2)-H(2)...O(2) | 0.899(16) | 1.875(17) | 2.7391(15) | 160.5(19) |
| N(3)-H(3)...O(1)#1 | 0.873(18) | 2.083(19) | 2.8731(15) | 150(2) |
| N(4)-H(4)...O(1)#1 | 0.887(16) | 1.993(17) | 2.8249(15) | 155.6(18) |

Symmetry transformations used to generate equivalent atoms:

#1 -x+1,y+1/2,-z+3/2

**Table S6.** Bond lengths [Å] and angles [°] for **38**.

.

| | | | |
|---|---|---|---|
| C(2)-C(3) | 1.395(2) | C(33')-C(34') | 1.366(10) |
| C(2)-C(7) | 1.400(2) | C(34')-C(35') | 1.403(9) |
| C(2)-N(1) | 1.4066(18) | C(36)-C(37) | 1.5211(18) |
| C(1)-O(1) | 1.2425(15) | C(36)-C(55) | 1.5360(18) |
| C(1)-N(2) | 1.3578(16) | C(36)-C(44) | 1.5856(18) |
| C(1)-N(1) | 1.3684(17) | C(37)-C(42) | 1.3881(18) |
| N(2)-C(8) | 1.4757(16) | C(37)-C(38) | 1.3934(19) |
| N(3)-C(29) | 1.3665(17) | C(38)-C(39) | 1.392(2) |
| N(3)-C(30) | 1.420(11) | C(39)-C(40) | 1.396(2) |
| N(3)-C(30') | 1.445(11) | C(40)-C(41) | 1.397(2) |
| N(4)-C(29) | 1.3515(16) | C(40)-C(56) | 1.504(2) |
| N(4)-C(36) | 1.4641(16) | C(41)-C(42) | 1.3910(19) |
| O(2)-C(29) | 1.2354(16) | C(42)-C(43) | 1.5003(19) |
| C(3)-C(4) | 1.397(2) | C(43)-C(47) | 1.5220(18) |
| C(4)-C(5) | 1.387(3) | C(43)-C(44) | 1.5686(18) |
| C(5)-C(6) | 1.381(3) | C(44)-C(45) | 1.5144(18) |
| C(6)-C(7) | 1.391(2) | C(45)-C(54) | 1.3747(19) |
| C(8)-C(9) | 1.5206(18) | C(45)-C(46) | 1.4127(19) |
| C(8)-C(27) | 1.5319(18) | C(46)-C(51) | 1.4064(19) |
| C(8)-C(16) | 1.5636(18) | C(46)-C(47) | 1.4100(19) |
| C(9)-C(14) | 1.3854(19) | C(47)-C(48) | 1.3748(19) |
| C(9)-C(10) | 1.3970(18) | C(48)-C(49) | 1.424(2) |
| C(10)-C(11) | 1.393(2) | C(49)-C(50) | 1.373(2) |
| C(11)-C(12) | 1.394(2) | C(50)-C(51) | 1.423(2) |
| C(12)-C(13) | 1.398(2) | C(51)-C(52) | 1.424(2) |
| C(12)-C(28) | 1.514(2) | C(52)-C(53) | 1.378(2) |
| C(13)-C(14) | 1.3980(18) | C(53)-C(54) | 1.427(2) |
| C(14)-C(15) | 1.5163(18) | | |
| C(15)-C(19) | 1.5146(19) | C(3)-C(2)-C(7) | 119.24(14) |
| C(15)-C(16) | 1.5789(18) | C(3)-C(2)-N(1) | 124.19(14) |
| C(16)-C(17) | 1.5199(18) | C(7)-C(2)-N(1) | 116.55(14) |
| C(17)-C(26) | 1.3783(19) | O(1)-C(1)-N(2) | 122.77(12) |
| C(17)-C(18) | 1.4072(18) | O(1)-C(1)-N(1) | 123.40(12) |
| C(18)-C(23) | 1.4077(19) | N(2)-C(1)-N(1) | 113.83(11) |
| C(18)-C(19) | 1.4105(19) | C(1)-N(1)-C(2) | 128.74(12) |
| C(19)-C(20) | 1.371(2) | C(1)-N(2)-C(8) | 122.45(10) |
| C(20)-C(21) | 1.415(2) | C(29)-N(3)-C(30) | 120.2(19) |
| C(21)-C(22) | 1.373(3) | C(29)-N(3)-C(30') | 119.4(18) |
| C(22)-C(23) | 1.423(2) | C(29)-N(4)-C(36) | 123.47(11) |
| C(23)-C(24) | 1.416(2) | C(2)-C(3)-C(4) | 119.21(17) |
| C(24)-C(25) | 1.376(3) | C(5)-C(4)-C(3) | 121.44(18) |
| C(25)-C(26) | 1.426(2) | C(6)-C(5)-C(4) | 119.14(16) |
| C(30)-C(35) | 1.364(13) | C(5)-C(6)-C(7) | 120.42(18) |
| C(30)-C(31) | 1.376(12) | C(6)-C(7)-C(2) | 120.52(17) |
| C(31)-C(32) | 1.364(11) | N(2)-C(8)-C(9) | 105.83(10) |
| C(32)-C(33) | 1.364(11) | N(2)-C(8)-C(27) | 110.11(11) |
| C(33)-C(34) | 1.372(11) | C(9)-C(8)-C(27) | 111.27(11) |
| C(34)-C(35) | 1.396(11) | N(2)-C(8)-C(16) | 109.57(10) |
| C(30')-C(35') | 1.381(12) | C(9)-C(8)-C(16) | 103.51(10) |
| C(30')-C(31') | 1.385(13) | C(27)-C(8)-C(16) | 115.94(10) |
| C(31')-C(32') | 1.413(10) | C(14)-C(9)-C(10) | 120.68(12) |
| C(32')-C(33') | 1.368(11) | C(14)-C(9)-C(8) | 112.60(11) |

| | | | |
|---|---|---|---|
| C(10)-C(9)-C(8) | 126.63(12) | C(37)-C(36)-C(55) | 109.49(11) |
| C(11)-C(10)-C(9) | 118.58(14) | N(4)-C(36)-C(44) | 112.71(11) |
| C(10)-C(11)-C(12) | 121.50(14) | C(37)-C(36)-C(44) | 103.15(10) |
| C(11)-C(12)-C(13) | 119.17(13) | C(55)-C(36)-C(44) | 113.13(10) |
| C(11)-C(12)-C(28) | 119.84(14) | C(42)-C(37)-C(38) | 119.75(13) |
| C(13)-C(12)-C(28) | 120.98(15) | C(42)-C(37)-C(36) | 112.09(11) |
| C(12)-C(13)-C(14) | 119.74(14) | C(38)-C(37)-C(36) | 128.15(12) |
| C(9)-C(14)-C(13) | 120.31(13) | C(39)-C(38)-C(37) | 119.07(13) |
| C(9)-C(14)-C(15) | 111.41(11) | C(38)-C(39)-C(40) | 121.64(14) |
| C(13)-C(14)-C(15) | 128.27(12) | C(39)-C(40)-C(41) | 118.69(14) |
| C(19)-C(15)-C(14) | 114.73(11) | C(39)-C(40)-C(56) | 120.77(15) |
| C(19)-C(15)-C(16) | 104.78(10) | C(41)-C(40)-C(56) | 120.50(16) |
| C(14)-C(15)-C(16) | 104.08(10) | C(42)-C(41)-C(40) | 119.81(14) |
| C(17)-C(16)-C(8) | 117.59(10) | C(37)-C(42)-C(41) | 121.02(13) |
| C(17)-C(16)-C(15) | 104.23(10) | C(37)-C(42)-C(43) | 111.07(11) |
| C(8)-C(16)-C(15) | 106.86(10) | C(41)-C(42)-C(43) | 127.91(12) |
| C(26)-C(17)-C(18) | 118.52(13) | C(42)-C(43)-C(47) | 115.00(11) |
| C(26)-C(17)-C(16) | 132.36(13) | C(42)-C(43)-C(44) | 105.03(10) |
| C(18)-C(17)-C(16) | 109.01(11) | C(47)-C(43)-C(44) | 103.75(10) |
| C(17)-C(18)-C(23) | 124.04(13) | C(45)-C(44)-C(43) | 104.00(10) |
| C(17)-C(18)-C(19) | 112.71(12) | C(45)-C(44)-C(36) | 114.56(10) |
| C(23)-C(18)-C(19) | 123.24(13) | C(43)-C(44)-C(36) | 104.82(10) |
| C(20)-C(19)-C(18) | 119.16(14) | C(54)-C(45)-C(46) | 118.46(13) |
| C(20)-C(19)-C(15) | 132.07(14) | C(54)-C(45)-C(44) | 133.00(13) |
| C(18)-C(19)-C(15) | 108.77(11) | C(46)-C(45)-C(44) | 108.32(12) |
| C(19)-C(20)-C(21) | 118.64(15) | C(51)-C(46)-C(47) | 123.38(13) |
| C(22)-C(21)-C(20) | 122.51(15) | C(51)-C(46)-C(45) | 124.28(13) |
| C(21)-C(22)-C(23) | 120.22(15) | C(47)-C(46)-C(45) | 112.29(12) |
| C(18)-C(23)-C(24) | 116.13(13) | C(48)-C(47)-C(46) | 119.16(13) |
| C(18)-C(23)-C(22) | 116.22(14) | C(48)-C(47)-C(43) | 132.66(13) |
| C(24)-C(23)-C(22) | 127.65(14) | C(46)-C(47)-C(43) | 108.18(11) |
| C(25)-C(24)-C(23) | 120.34(14) | C(47)-C(48)-C(49) | 118.41(14) |
| C(24)-C(25)-C(26) | 122.30(15) | C(50)-C(49)-C(48) | 122.34(14) |
| C(17)-C(26)-C(25) | 118.58(14) | C(49)-C(50)-C(51) | 120.38(13) |
| O(2)-C(29)-N(4) | 123.18(12) | C(46)-C(51)-C(50) | 116.20(13) |
| O(2)-C(29)-N(3) | 121.04(12) | C(46)-C(51)-C(52) | 116.02(13) |
| N(4)-C(29)-N(3) | 115.78(11) | C(50)-C(51)-C(52) | 127.77(13) |
| C(35)-C(30)-C(31) | 118.3(10) | C(53)-C(52)-C(51) | 119.71(14) |
| C(35)-C(30)-N(3) | 120.3(11) | C(52)-C(53)-C(54) | 123.01(14) |
| C(31)-C(30)-N(3) | 121.4(10) | C(45)-C(54)-C(53) | 118.33(14) |
| C(32)-C(31)-C(30) | 119.8(10) | | |
| C(31)-C(32)-C(33) | 122.9(10) | | |
| C(32)-C(33)-C(34) | 117.8(10) | | |
| C(33)-C(34)-C(35) | 119.7(10) | | |
| C(30)-C(35)-C(34) | 121.5(9) | | |
| C(35')-C(30')-C(31') | 120.4(9) | | |
| C(35')-C(30')-N(3) | 120.7(10) | | |
| C(31')-C(30')-N(3) | 118.9(10) | | |
| C(30')-C(31')-C(32') | 119.8(10) | | |
| C(33')-C(32')-C(31') | 118.9(10) | | |
| C(34')-C(33')-C(32') | 121.4(10) | | |
| C(33')-C(34')-C(35') | 120.2(8) | | |
| C(30')-C(35')-C(34') | 119.1(8) | | |
| N(4)-C(36)-C(37) | 112.35(11) | | |
| N(4)-C(36)-C(55) | 106.12(10) | | |

## Ligand Parameterization

### Ligand Screening Data Used for Feature Analysis

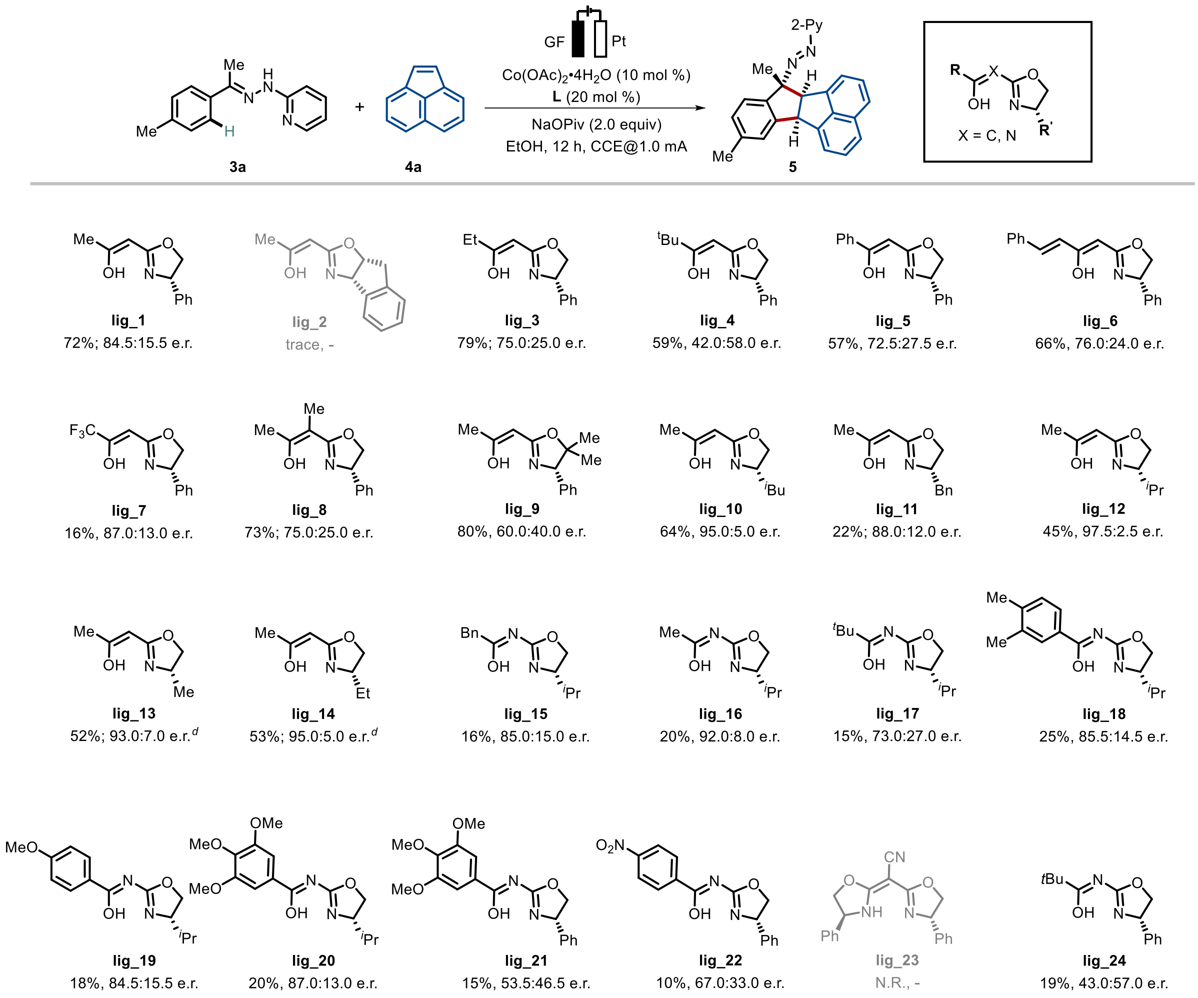


**Figure S21.** Ligand screening data used for feature analysis and MVLR studies with the indicated product's yield (%) and enantiomeric ratio (e.r.). Unsuccessful ligands (faded) were not included in statistical model development.

### Features (descriptor) Calculations and Analysis

The above-mentioned free ligands' (FL) 3D structures were generated with the help of ETKDG[12] method integrated into the RDKit library.[13] Conformational search was then performed using Grimme's Conformer-Rotamer Ensemble Sampling Tool (CREST)[14] at the GFN2-xTB[15] level of theory. The most stable conformer was then optimized using Gaussian 16, Revision A.03 package[16] at M06-2X/def2-TZVPP level of theory. To better understand the ligand behavior in a metal complexation setting, a hydrazone substrate was considered for a cobalt-cyclometalated complex (CMC) with the ligand corresponding to the intermediate before the migratory insertion step. These CMC structures were optimized at TPSS/def2-SVP level of theory with singlet spin multiplicity to minimize the computational cost. All the geometry optimizations were performed in the gas phase. The xyz coordinates of optimized geometries for all the considered FL and CMC structures can be found in the folders named "optimized_FL_strs" and "optimized_CMC_strs", respectively.[17]

Several features describing electronic, geometric, and steric properties of the ligand and its surroundings (in CMC) were used and have been discussed below (**Figure S22**) and in further sub-sections. Natural bond orbital (NBO) atomic charges of indicated atoms and the main dihedral angle (ϕ) of the ligand backbone were used from both the FL and CMC systems. The NBO charges were collected from the single point calculations for this were performed in gas phase at M06-2X/def2-TZVPP (for FL) and PW6B95D3/def2-TZVPP (for CMC) level of theories. Sterimol parameters ($B_{min}$, $B_{max}$, and L) were measured from the C2 and C6 substituents of the FL structures using Paton's DFT-based Steric Parameters (DBSTEP) python package.[18] Moreover, the crucial bond lengths (ℓ) and bite angle (θ) from the ligand coordinating atoms (O1 and N5) to the metal (Co) were measured from the CMC geometries. All the features used were summarized in **Table S8**.

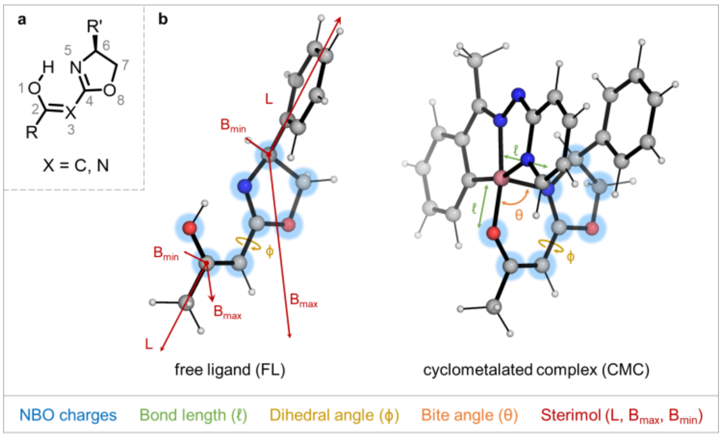


**Figure S22.** Ligand featurization: a) general atom indexing, b) NBO charges, bond lengths, dihedral angle, bite angle, and sterimol parameters from the FL and the CMC structure. Here lig_1 ligand has been taken for illustration.

**Percentage Buried Volume (%Vbur)**

In addition to the sterimol parameters measured from the FL structures, percentage of buried volumes (%Vbur) at 3.5 Å sphere radius were calculated from the CMC model geometries. Firstly, %Vburs of the whole sphere at Co center were calculated. Then %Vburs of each divided octants were calculated at same radii excluding the contributions of the considered substrate (**Figure S23**). Thus, due to zero variance in Vbur_oct0, and Vbur_oct1 were eliminated before feature analysis.

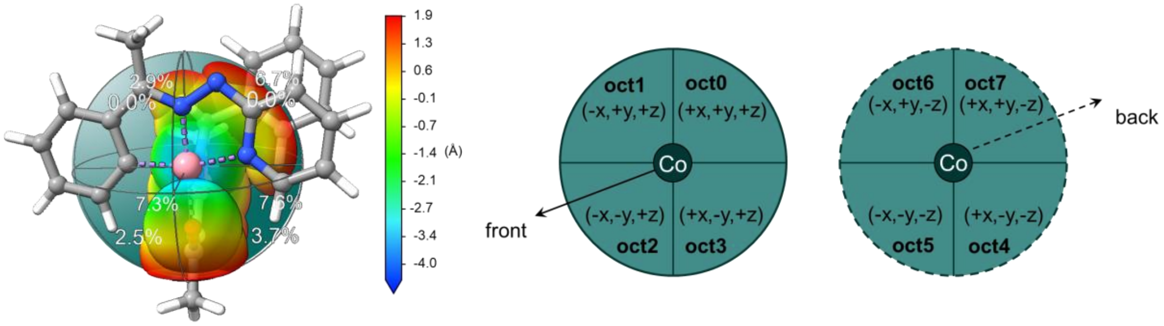


**Figure S23.** %Vbur at Co center of coordinated ligand in divided octants of complex sphere. Here lig_1 ligand has been taken for illustration.

## Interaction Energy (IE) Calculation

To better describe the interaction between the oxazoline substituents and the hydrazone substrate, interaction energy (IE) was considered as a descriptor. The IE (in Hartree) for each ligand was measured using **Equation S1**. To measure this, the pre-optimized CMC geometries with each ligand were used (**Figure S22**). The metal (cobalt) with some parts of the complex were excluded to avoid their interaction contributions and the connecting points were terminated by substituting them with hydrogen atoms. Thereafter, the energies were computed at PW6B95D3/def2-TZVPP level of theory for the remaining fragment as all together ($F_T$), followed by the energies of the substrate ($F_S$) and ligand ($F_L$) fragments separately without altering the geometries (**Figure S24**).

$$IE = EE_T - (EE_S + EE_L) \qquad \textbf{(Equation S1)}$$

Where, $EE_T$, $EE_S$, and $EE_L$ are the electronic energies of $F_T$, $F_S$, and $F_L$, respectively.

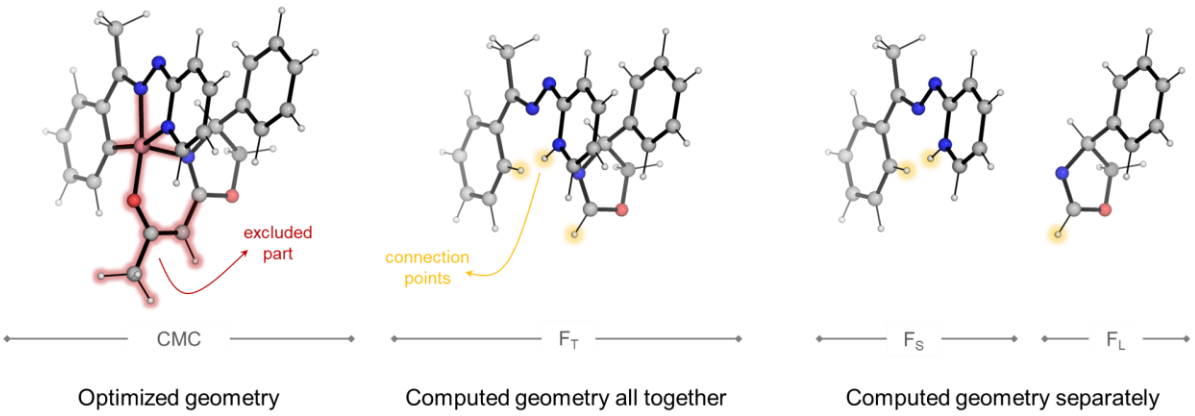


**Figure S24.** Measurement of the IE from all ligands' CMCs. Here lig_1 ligand has been taken for illustration.

**Tabel S7.** Calculated electronic energies at the PW6B95D3/def2-TZVPP level of theory (all in Hartree, Ha) for different ligands from their corresponding optimized CMC geometries.

| Ligand | Electronic Energy (Ha) | | | |
|---|---|---|---|---|
| | $F_T$ | $F_S$ | $F_L$ | IE |
| lig_1 | -1147.651030 | -668.533360 | -479.116605 | -0.001065 |
| lig_3 | -1147.652630 | -668.534991 | -479.116647 | -0.000993 |
| lig_4 | -1147.654204 | -668.534982 | -479.117993 | -0.001230 |
| lig_5 | -1147.652810 | -668.534892 | -479.117224 | -0.000694 |
| lig_6 | -1147.652515 | -668.535015 | -479.116783 | -0.000718 |
| lig_7 | -1147.654337 | -668.534580 | -479.119109 | -0.000649 |
| lig_8 | -1147.651128 | -668.534960 | -479.115106 | -0.001063 |
| lig_9 | -1226.424657 | -668.534935 | -557.886205 | -0.003518 |
| lig_10 | -1073.691845 | -668.508858 | -405.183848 | 0.000860 |
| lig_11 | -1187.032524 | -668.534042 | -518.498179 | -0.000303 |
| lig_12 | -1034.321891 | -668.512989 | -365.811844 | 0.002942 |
| lig_13 | -955.583529 | -668.534281 | -287.051661 | 0.002413 |
| lig_14 | -994.941193 | -668.513626 | -326.430787 | 0.003219 |
| lig_15 | -1034.321946 | -668.512796 | -365.811493 | 0.002342 |

| | | | | |
|---|---|---|---|---|
| lig_16 | -1034.321504 | -668.512808 | -365.811544 | 0.002847 |
| lig_17 | -1034.321345 | -668.512667 | -365.811401 | 0.002723 |
| lig_18 | -1034.322192 | -668.513993 | -365.811087 | 0.002889 |
| lig_19 | -1034.321880 | -668.513861 | -365.810926 | 0.002907 |
| lig_20 | -1034.319979 | -668.513984 | -365.808923 | 0.002928 |
| lig_21 | -1147.651875 | -668.534724 | -479.116099 | -0.001052 |
| lig_22 | -1147.651818 | -668.533406 | -479.117138 | -0.001273 |
| lig_24 | -1147.653947 | -668.534902 | -479.117435 | -0.001610 |

**Tabel S8.** List of all the features considered for the analysis and model development.

| Feature name | Unit | Description |
|---|---|---|
| nbo_O1_cmc, nbo_C2_cmc, nbo_X3_cmc, nbo_C4_cmc, nbo_N5_cmc, nbo_C6_cmc, nbo_C7_cmc, nbo_O8_cmc, nbo_Co_cmc | e | NBO charges of indexed and Co atom from the cyclometalated complex |
| da_C2_X3_C4_N5_cmc | ° | Dihedral angle between the indexed atoms from the cyclometalated complex |
| bl_OCo_cmc, bl_NCo_cmc | Å | Bond length of the ligand's metal coordinating atoms from the cyclometalated complex |
| ba_OCoN_cmc | ° | Bite angle of the ligand from its metal coordinating atoms from the cyclometalated complex |
| vbur_Co_cmc | % | Percentage buried volume at the metal center from the cyclometalated complex |
| vbur_oct0_cmc, vbur_oct1_cmc, vbur_oct2_cmc, vbur_oct3_cmc, vbur_oct4_cmc, vbur_oct5_cmc, vbur_oct6_cmc, vbur_oct7_cmc | % | Percentage buried volume of each octant from the cyclometalated complex |
| IE | Ha | Interaction energy between the substrate and the ligand fragments |
| nbo_O1_fl, nbo_C2_fl, nbo_X3_fl, nbo_C4_fl, nbo_N5_fl, nbo_C6_fl, nbo_C7_fl, nbo_O8_fl | e | NBO charges of indexed and Co atom from the free ligand |
| da_C2_X3_C4_N5_fl | ° | Dihedral angle between the indexed atoms from the free ligand |
| sC2_Bmin_fl, sC2_Bmax_fl, sC2_L_fl | Å | Sterimol parameters of the ligand substituent at C2 from the free ligand |
| sC6_Bmin_fl, sC6_Bmax_fl, sC6_L_fl | Å | Sterimol parameters of the ligand substituent at C6 from the free ligand |

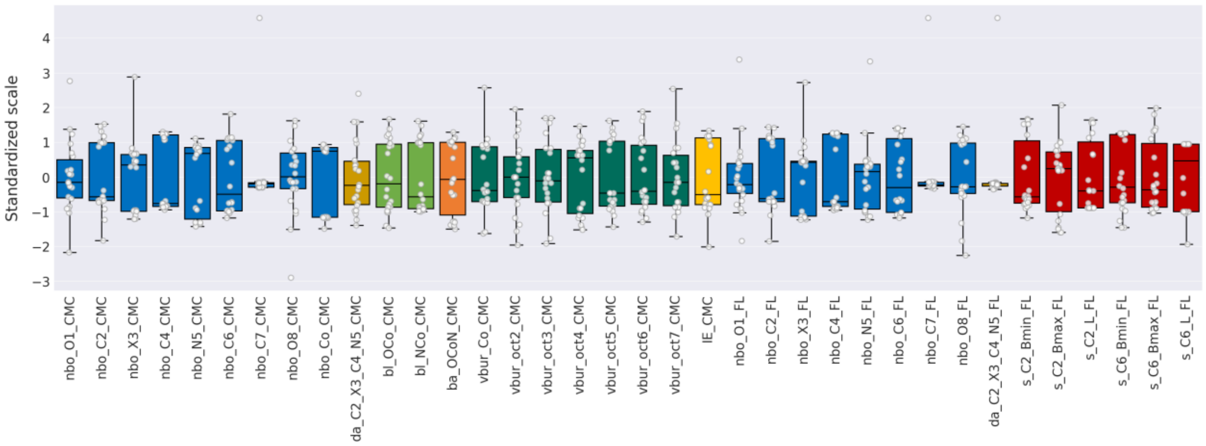


**Figure S25.** Box-plot for all the calculated features on a standardized scale.

## Statistical Modeling

Multivariate linear regression (MVLR) analyses were performed using python based in-house scripts (https://github.com/neeraj-compchem/Ligand-parameterization)[17] and scikit-learn libraries.[19] The ligand screening data was used with the measured $\Delta\Delta G^{\ddagger}$ (kcal mol$^{-1}$) values as reaction targets for the modelling which were calculated using **Equation S2**. All of the obtained features were normalized with scikit-learn's StandardScaler function to get the coefficient values indicating their relative importance on the same scale. Afterwards, highly correlated features (with a Pearson correlation coefficient > 0.9) were eliminated prior to the modelling to reduce computational cost for the following variable search. Given the reduced feature dimension, best subset selection method was used to select feature subsets in the model development with the train:test (80:20) split along using the available random function from the scikit-learn. Moreover, leave-one-out (LOO) cross-validations were also performed on the training sample. Mean absolute errors (MAE) and coefficient of determination (R2 score) values were used as performance metrics for each model on their train and test samples. Here independently best produced models (M1, M2, M3, M4, M5, and M6) were selected from each 1 to 6 variable (feature) sizes for overall performance evaluation (**Figure S26** and **S27**). The criteria of best possible correlation between measured and predicted $\Delta\Delta G^{\ddagger}$ values (largest R2 scores) were chosen for best overall model selection. It can be observed from the final evaluation that the model M5 had lowest errors ($MAE_{train}$ = 0.13 kcal mol$^{-1}$, $MAE_{test}$ = 0.26 kcal mol$^{-1}$), however the average $MAE_{LOO}$ for M5 ($MAE_{LOO}$ = 0.51 kcal mol$^{-1}$) was higher compared to M4 ($MAE_{LOO}$ = 0.20 kcal mol$^{-1}$) with very similar errors ($MAE_{train}$ = 0.16 kcal mol$^{-1}$, $MAE_{test}$ = 0.26 kcal mol$^{-1}$). The increase in $MAE_{LOO}$ and drop in R2 test scores from M5 onwards shows that the models started including noise from the data. Thus, the M4 model was selected as the best overall model as it shows better generalization ability.

$$\Delta\Delta G^{\ddagger} = - RT\ln(e.r.) \qquad \textbf{(Equation S2)}$$

Where, R is the gas constant (1.987 cal K$^{-1}$ mol$^{-1}$), T is temperature (298.15 K), and e.r. is the enantiomeric ratios for each ligand.

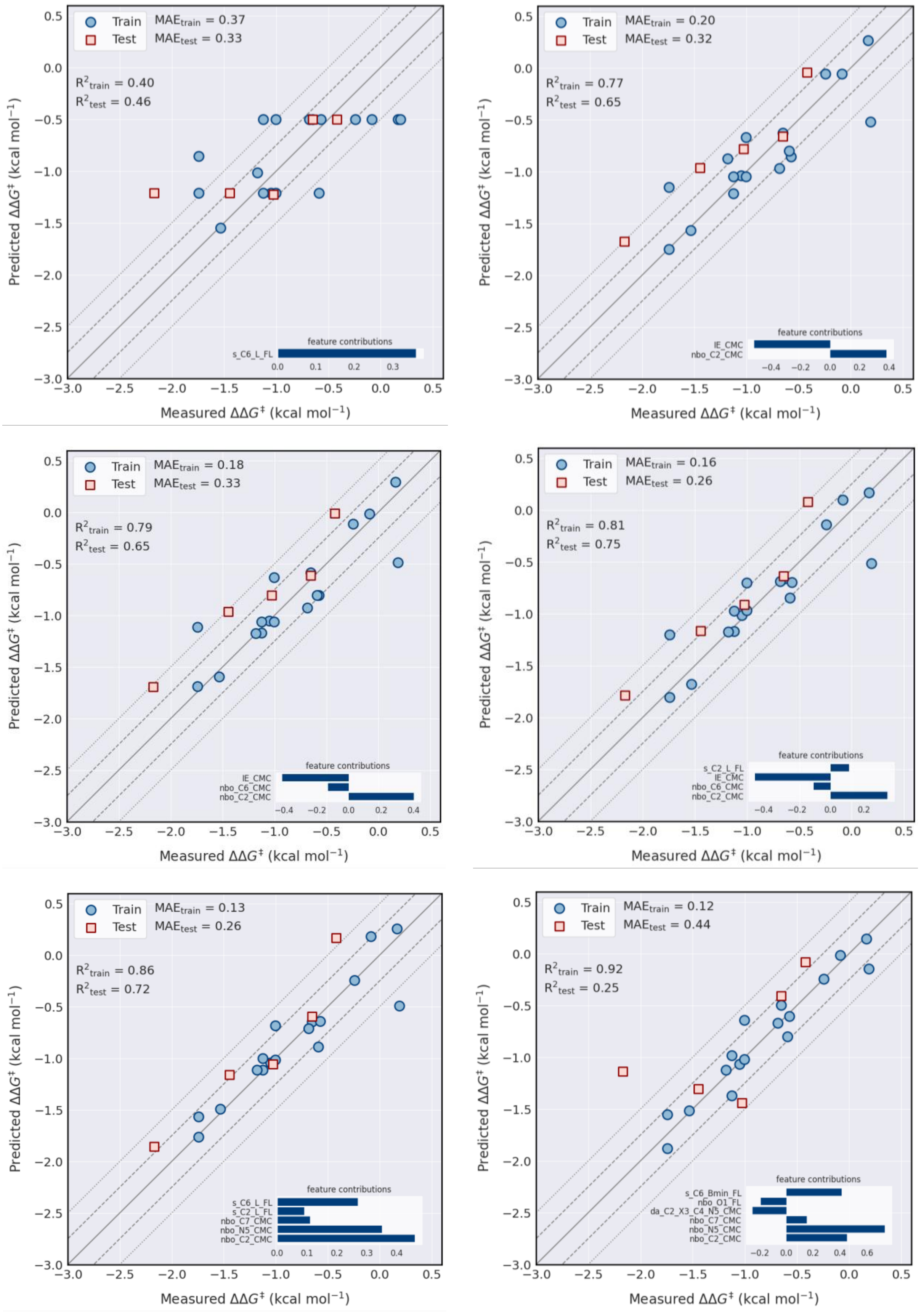


**Figure S26.** Measured vs. predicted $\Delta\Delta G^{\ddagger}$ value plots for all the final six models with their feature contributions.

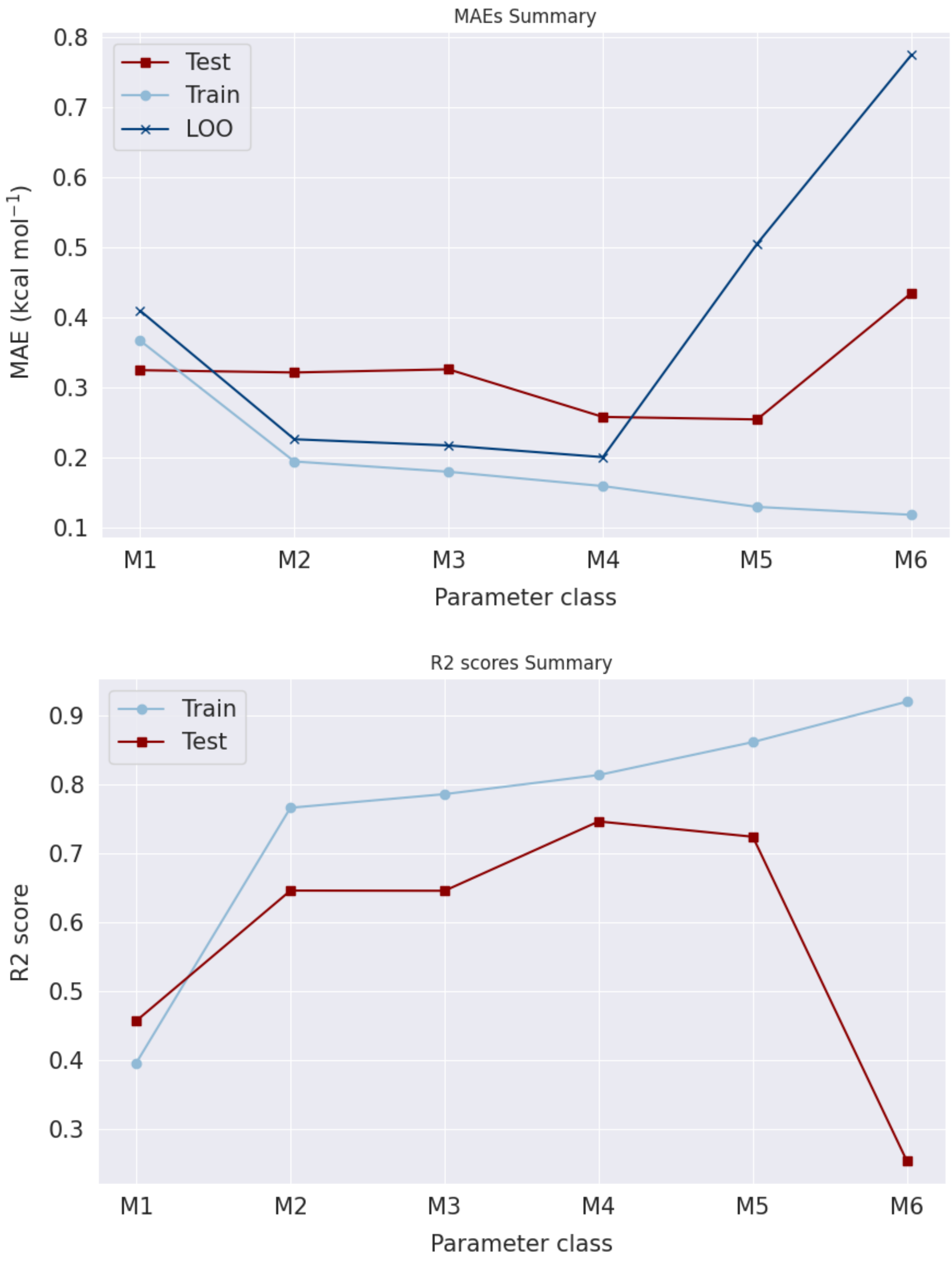


**Figure S27.** Performance metrics comparison of all the final six models.

**Tabel S9.** Measured, predicted and predicted$_{LOO}$ $\Delta\Delta G^{\ddagger}$ values in each sample class for the M4 model.

| Ligand | $\Delta\Delta G^{\ddagger}$ (kcal mol$^{-1}$) | | | Sample class |
|---|---|---|---|---|
| | Measured | Predicted | Predicted$_{LOO}$ | |
| lig_1 | -1.00 | -0.70 | -0.65 | train |
| lig_3 | -0.65 | -0.64 | NA | test |
| lig_4 | 0.19 | -0.51 | -0.60 | train |
| lig_5 | -0.57 | -0.70 | -0.73 | train |
| lig_6 | -0.68 | -0.69 | -0.69 | train |
| lig_7 | -1.13 | -1.17 | -1.18 | train |
| lig_8 | -0.65 | -0.66 | -0.67 | train |
| lig_9 | -0.24 | -0.14 | -0.09 | train |
| lig_10 | -1.74 | -1.20 | -1.10 | train |
| lig_11 | -1.18 | -1.17 | -1.12 | train |
| lig_12 | -2.17 | -1.78 | NA | test |
| lig_13 | -1.53 | -1.68 | -1.72 | train |
| lig_14 | -1.74 | -1.80 | -1.84 | train |
| lig_15 | -1.03 | -0.91 | NA | test |
| lig_16 | -1.45 | -1.16 | NA | test |
| lig_17 | -0.59 | -0.85 | -0.93 | train |
| lig_18 | -1.05 | -1.01 | -1.00 | train |
| lig_19 | -1.00 | -0.97 | -0.96 | train |
| lig_20 | -1.13 | -0.97 | -0.93 | train |
| lig_21 | -0.08 | 0.10 | 0.18 | train |
| lig_22 | -0.42 | 0.08 | NA | test |
| lig_24 | 0.17 | 0.17 | 0.17 | train |

## Ligand Reactivity Analysis

The analysis was performed using a modified Python script.**Error! Bookmark not defined.**[17] of the o riginal code from Newman-Stonebraker et al.'s reported work.[20] Here, a single node decision tree classifier was applied to classify the experimental yields for the selected feature values at a cutoff of 35% as the median yield of the dataset and class weight as "balanced". (**Table S10**).

**Tabel S10.** Performance metrics for the feature-wise univariate reactivity classification.

| Feature name | Unit | Feature threshold | Accuracy | F1 score |
|---|---|---|---|---|
| nbo_C2_CMC | e | 0.57 | 0.91 | 0.92 |
| nbo_C6_CMC | e | -0.07 | 0.82 | 0.85 |
| s_C2_L_FL | Å | 6.42 | 0.73 | 0.77 |
| IE_CMC | kcal mol$^{-1}$ | -0.421 | 0.68 | 0.67 |

## Computational Studies

### Method Details

All DFT calculations were performed using Gaussian 16, Revision A.03 package.[16] Geometry optimizations were performed at the TPSS[21] level of theory in combination with Grimme's D3 dispersion corrections with a Becke-Johnson damping scheme (D3BJ)[22-23] in the gas phase. All atoms were described with a def2-SVP basis set.[24-27] Analytical frequencies were carried out at the same level of theory to identify each stationary point as either intermediates (zero imaginary frequencies) or transition states (one imaginary frequency). These also provided thermal and non-thermal corrections to the Gibbs free energy at room temperature (298.15 K) and 1 atm pressure. Transition states were verified by intrinsic reaction coordinate (IRC) calculations.[28] The electronic energy was then refined through single-point calculations with the PW6B95[29] functional on the optimized geometries with a def2-TZVPP basis set[24-27] in combination with a standalone version of Grimme's dispersion correction D4.[30-31] Considering the 1:1 solvent mixture of Acetone: EtOH, solvent effects were included implicitly through the use of the individual SMD models[32] with a dielectric constant of $\varepsilon = 20.493$, and $\varepsilon = 24.852$, which corresponds to Acetone and Ethanol, respectively. The reported energies are based on gas-phase Gibbs free energies with a def2-SVP basis set for which the electronic energies were corrected to PW6B95-D4 with a def2-TZVPP basis set including solvent effects.

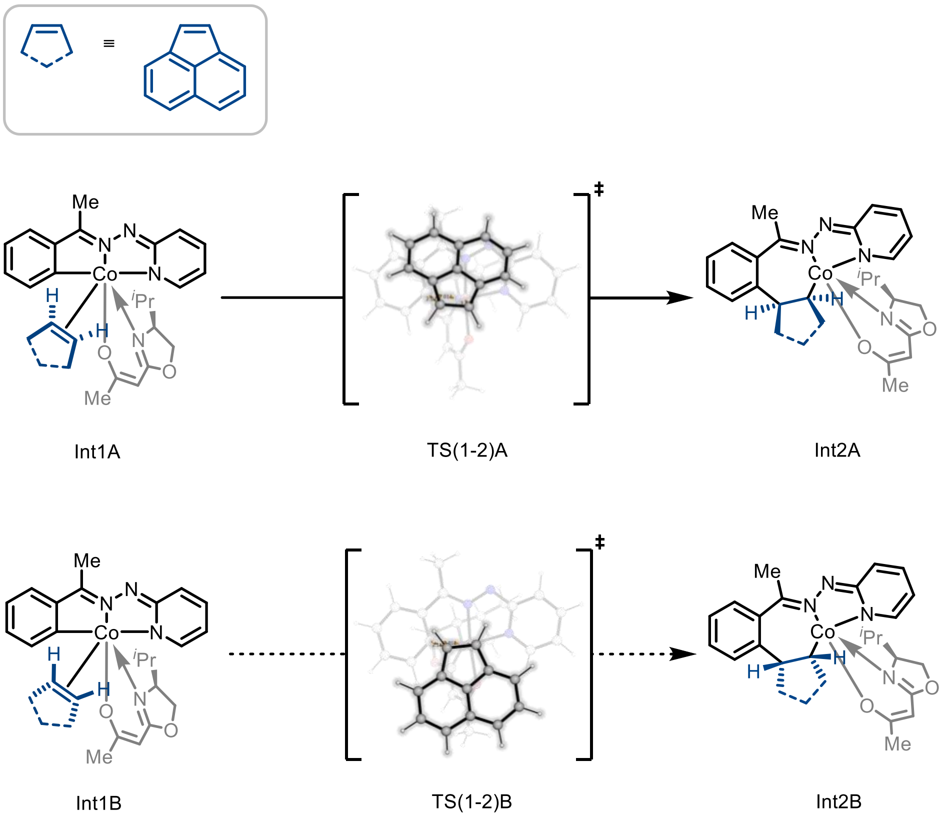


**Figure S28.** Stereoisomers generation *via* migratory insertion step.

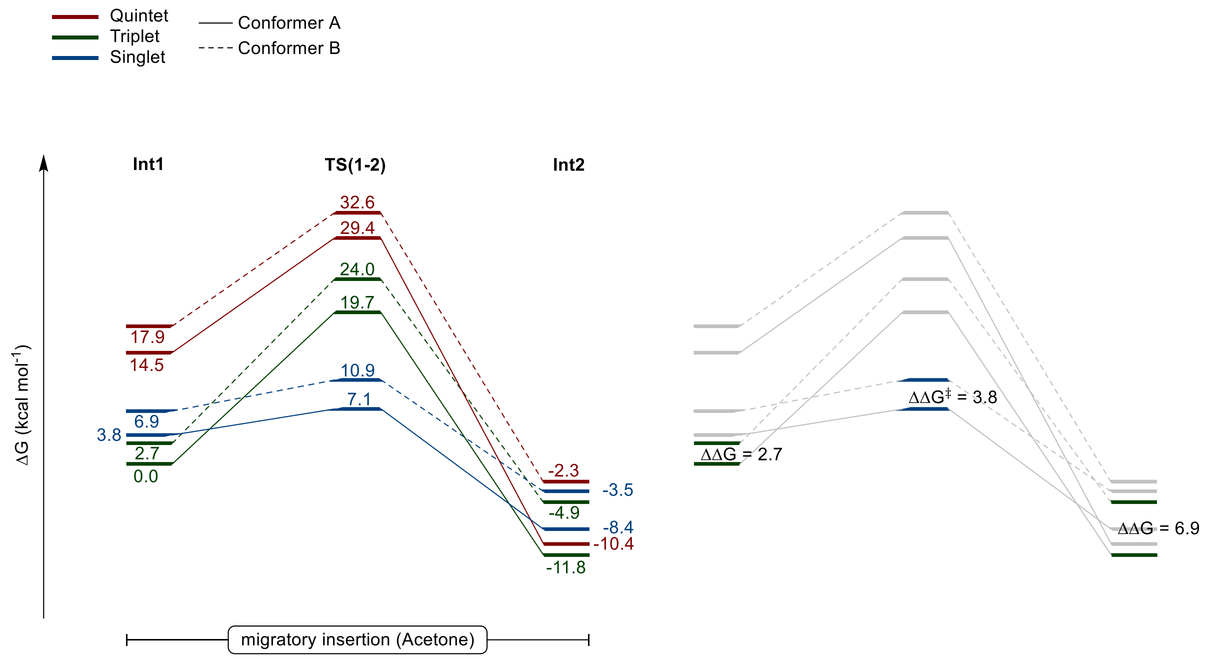


**Figure S29.** Computed relative Gibbs free energies ($\Delta G_{298.15}$) in kcal mol$^{-1}$ for the migratory insertion step with two stereoisomeric conformation pathways at the PW6B95-D4/def2-TZVPP+SMD(Acetone)//TPSS-D3(BJ)/def2-SVP level of theory.

**Table S11.** Calculated electronic and Gibbs free energies at the PW6B95-D4/def2-TZVPP+SMD(Acetone)//TPSS-D3(BJ)/def2-SVP level of theory with dispersion corrections (all in Hartree) for conformer A and B in the migratory insertion step. #Superscripted 1, 3, and 5 represents the singlet, triplet, and quintet multiplicity states, respectively.

| Conformer | Reaction Coordinate# | Electronic Energy | Total Gibbs Free Energy |
|---|---|---|---|
| A | **Int1$^1$** | -3071.412564 | -3070.919797 |
| | **Int1$^3$** | -3071.412774 | -3070.925806 |
| | **Int1$^5$** | -3071.386864 | -3070.902738 |
| | **TS(1-2)$^1$** | -3071.406310 | -3070.914428 |
| | **TS(1-2)$^3$** | -3071.381527 | -3070.894357 |
| | **TS(1-2)$^5$** | -3071.363923 | -3070.879013 |
| | **Int2$^1$** | -3071.432795 | -3070.939256 |
| | **Int2$^3$** | -3071.437696 | -3070.944665 |
| | **Int2$^5$** | -3071.431668 | -3070.942367 |
| B | **Int1$^1$** | -3071.408343 | -3070.914863 |
| | **Int1$^3$** | -3071.407310 | -3070.921495 |
| | **Int1$^5$** | -3071.380676 | -3070.897234 |
| | **TS(1-2)$^1$** | -3071.401077 | -3070.908375 |
| | **TS(1-2)$^3$** | -3071.375228 | -3070.887533 |
| | **TS(1-2)$^5$** | -3071.358711 | -3070.873841 |
| | **Int2$^1$** | -3071.424625 | -3070.931393 |
| | **Int2$^3$** | -3071.425463 | -3070.933651 |
| | **Int2$^5$** | -3071.415451 | -3070.929459 |

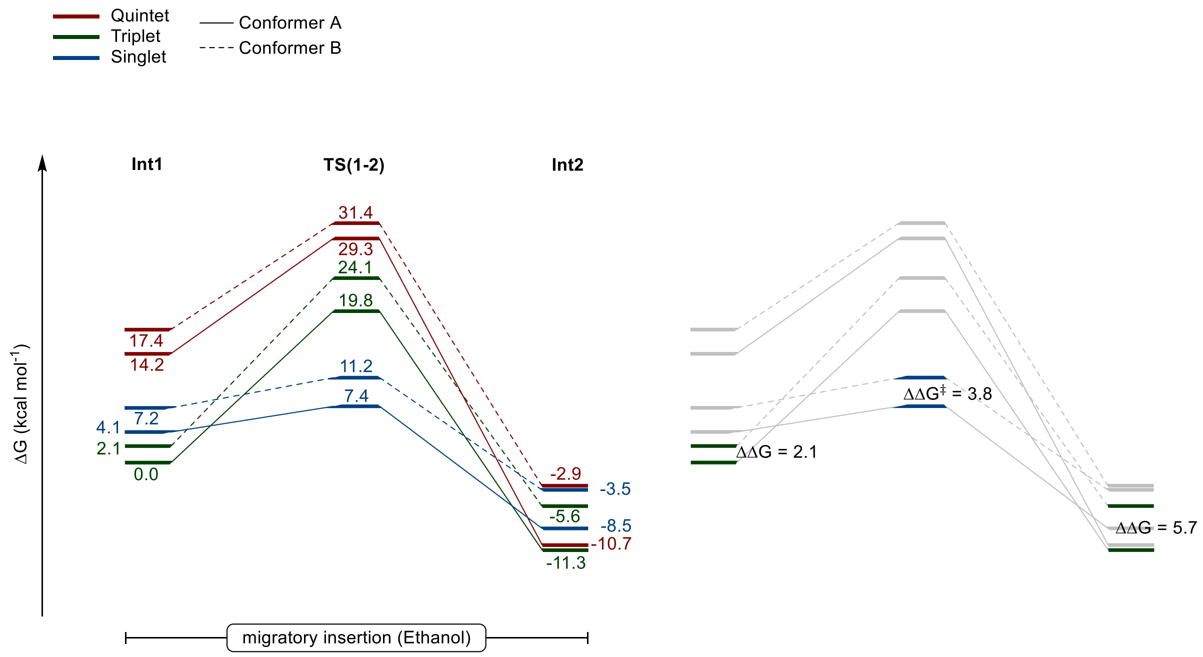


**Figure S30.** Computed relative Gibbs free energies ($\Delta G_{298.15}$) in kcal mol$^{-1}$ for the migratory insertion step with two stereoisomeric conformation pathways at the PW6B95-D4/def2-TZVPP+SMD(Ethanol)//TPSS-D3(BJ)/def2-SVP level of theory.

**Table S12.** Calculated electronic and Gibbs free energies at the PW6B95-D4/def2-TZVPP+SMD(Ethanol)//TPSS-D3(BJ)/def2-SVP level of theory with dispersion corrections (all in Hartree) for conformer A and B in the migratory insertion step. #Superscripted 1, 3, and 5 represents the singlet, triplet, and quintet multiplicity states, respectively.

| Conformer | Reaction Coordinate# | Electronic Energy | Total Gibbs Free Energy |
|---|---|---|---|
| **A** | **Int1$^1$** | -3071.409018 | -3070.916251 |
| | **Int1$^3$** | -3071.409713 | -3070.922745 |
| | **Int1$^5$** | -3071.384231 | -3070.900104 |
| | **TS(1-2)$^1$** | -3071.402905 | -3070.911023 |
| | **TS(1-2)$^3$** | -3071.378364 | -3070.891194 |
| | **TS(1-2)$^5$** | -3071.361021 | -3070.876110 |
| | **Int2$^1$** | -3071.429848 | -3070.936310 |
| | **Int2$^3$** | -3071.433779 | -3070.940747 |
| | **Int2$^5$** | -3071.429156 | -3070.939855 |
| **B** | **Int1$^1$** | -3071.404808 | -3070.911327 |
| | **Int1$^3$** | -3071.405161 | -3070.919346 |
| | **Int1$^5$** | -3071.378532 | -3070.895090 |
| | **TS(1-2)$^1$** | -3071.397651 | -3070.904949 |
| | **TS(1-2)$^3$** | -3071.372035 | -3070.884340 |
| | **TS(1-2)$^5$** | -3071.357794 | -3070.872924 |
| | **Int2$^1$** | -3071.421559 | -3070.928326 |
| | **Int2$^3$** | -3071.423482 | -3070.931670 |
| | **Int2$^5$** | -3071.413436 | -3070.927444 |

## Cartesian Coordinates of the Optimized Structures from Computational Studies

### Int1A

Lowest frequency = 26.3469 cm$^{-1}$

Charge = 0, Multiplicity = 1

74

| | | | |
|---|---|---|---|
| C | 0.346955 | -1.872007 | 0.251940 |
| C | -0.304791 | -2.120782 | -0.992456 |
| C | 0.921932 | -2.938756 | 0.946230 |
| C | -0.348287 | -3.427697 | -1.522148 |
| C | 0.875935 | -4.243575 | 0.411480 |
| H | 1.416500 | -2.770867 | 1.909070 |
| C | 0.245717 | -4.485939 | -0.818974 |
| H | -0.845563 | -3.613503 | -2.480634 |
| H | 1.333299 | -5.071350 | 0.965339 |
| H | 0.212599 | -5.501234 | -1.228575 |
| C | -0.885576 | -0.951097 | -1.626301 |
| N | -0.594585 | 0.174331 | -0.984624 |
| C | -0.434334 | 2.369361 | -0.694074 |
| C | -0.658832 | 3.742546 | -1.001140 |
| N | 0.360498 | 2.041698 | 0.386124 |
| C | -0.036974 | 4.720116 | -0.238900 |
| H | -1.308292 | 3.982734 | -1.846096 |
| C | 0.954981 | 2.997856 | 1.119134 |
| C | 0.801930 | 4.355132 | 0.841677 |
| H | -0.195216 | 5.777903 | -0.476082 |
| H | 1.559544 | 2.632725 | 1.957087 |
| H | 1.308923 | 5.105287 | 1.453797 |
| N | 2.128952 | -0.111878 | -0.313162 |
| C | 3.122679 | -0.721866 | 0.323056 |
| C | 2.398693 | -0.245961 | -1.770409 |

| | | | |
|---|---|---|---|
| H | 1.460544 | -0.466545 | -2.300697 |
| C | 3.341518 | -1.454587 | -1.785845 |
| H | 4.123929 | -1.412018 | -2.558330 |
| H | 2.800092 | -2.416343 | -1.837706 |
| O | 3.991338 | -1.379841 | -0.486402 |
| C | 3.386291 | -0.756889 | 1.712745 |
| H | 4.327966 | -1.203462 | 2.034431 |
| C | 2.466644 | -0.275641 | 2.637351 |
| O | 1.270928 | 0.116447 | 2.340440 |
| C | 2.806854 | -0.204238 | 4.109834 |
| H | 2.060032 | -0.771330 | 4.693022 |
| H | 3.814064 | -0.595094 | 4.320692 |
| H | 2.750853 | 0.846391 | 4.447347 |
| C | 3.066268 | 1.006037 | -2.411638 |
| H | 3.316275 | 0.670266 | -3.439569 |
| C | 2.129283 | 2.210975 | -2.534845 |
| H | 1.962779 | 2.674474 | -1.549983 |
| H | 2.580512 | 2.976470 | -3.190676 |
| H | 1.146428 | 1.930358 | -2.947752 |
| C | 4.371850 | 1.417383 | -1.710690 |
| H | 4.827867 | 2.276227 | -2.233615 |
| H | 4.164558 | 1.728158 | -0.671284 |
| H | 5.114786 | 0.603114 | -1.679375 |
| N | -0.953968 | 1.380579 | -1.448481 |
| C | -1.700111 | -0.958011 | -2.881646 |
| H | -1.062336 | -1.115546 | -3.773398 |
| H | -2.442428 | -1.773906 | -2.851218 |
| H | -2.213083 | 0.008765 | -2.989290 |
| Co | 0.385316 | 0.048778 | 0.616934 |
| C | -2.529583 | 1.064488 | 1.157588 |
| C | -3.159388 | -0.113131 | 0.669724 |
| C | -3.120883 | 2.292637 | 0.886843 |

| | | | |
|---|---|---|---|
| C | -2.437813 | -1.271693 | 1.056195 |
| C | -4.332754 | -0.125638 | -0.112432 |
| C | -4.316992 | 2.312471 | 0.110264 |
| H | -2.680285 | 3.232248 | 1.234182 |
| C | -2.913330 | -2.514912 | 0.659108 |
| C | -4.801498 | -1.416927 | -0.506829 |
| C | -4.909614 | 1.153273 | -0.388947 |
| H | -4.781126 | 3.280252 | -0.108154 |
| C | -4.106980 | -2.563819 | -0.120630 |
| H | -2.380544 | -3.438417 | 0.906197 |
| H | -5.712347 | -1.506918 | -1.109317 |
| H | -5.826766 | 1.221363 | -0.984518 |
| H | -4.485377 | -3.542824 | -0.434104 |
| C | -1.250950 | -0.796260 | 1.804442 |
| H | -0.698739 | -1.405369 | 2.519044 |
| C | -1.315997 | 0.623028 | 1.867204 |
| H | -0.774440 | 1.219755 | 2.600166 |

**Int1A**

Lowest frequency = 13.5126 $cm^{-1}$

Charge = 0, Multiplicity = 3

74

| | | | |
|---|---|---|---|
| C | 0.493634 | -1.821323 | 0.269939 |
| C | -0.075259 | -2.193313 | -0.994095 |
| C | 1.013630 | -2.823368 | 1.100309 |
| C | -0.076607 | -3.544897 | -1.406590 |
| C | 1.003628 | -4.167339 | 0.684871 |
| H | 1.449049 | -2.562566 | 2.070794 |
| C | 0.463866 | -4.523869 | -0.565936 |
| H | -0.509322 | -3.827508 | -2.372602 |

| | | | |
|---|---|---|---|
| H | 1.427148 | -4.941525 | 1.335005 |
| H | 0.463245 | -5.573261 | -0.880181 |
| C | -0.669794 | -1.092778 | -1.715925 |
| N | -0.492639 | 0.064637 | -1.073543 |
| C | -0.743394 | 2.224441 | -0.710025 |
| C | -1.205277 | 3.546851 | -0.971053 |
| N | -0.003365 | 1.968311 | 0.432707 |
| C | -0.922423 | 4.554764 | -0.065134 |
| H | -1.784368 | 3.719346 | -1.880898 |
| C | 0.246704 | 2.959054 | 1.314509 |
| C | -0.184438 | 4.265167 | 1.111365 |
| H | -1.272495 | 5.575189 | -0.254731 |
| H | 0.825884 | 2.657827 | 2.194285 |
| H | 0.058093 | 5.044383 | 1.838667 |
| N | 2.414899 | 0.119807 | -0.366597 |
| C | 3.420313 | -0.360743 | 0.346946 |
| C | 2.777133 | -0.011568 | -1.792814 |
| H | 1.915520 | -0.412069 | -2.352974 |
| C | 3.927878 | -1.032104 | -1.739889 |
| H | 4.755150 | -0.820490 | -2.434825 |
| H | 3.578031 | -2.071070 | -1.877661 |
| O | 4.432896 | -0.907250 | -0.385332 |
| C | 3.560931 | -0.387003 | 1.759839 |
| H | 4.524584 | -0.716396 | 2.152126 |
| C | 2.517449 | -0.087255 | 2.634244 |
| O | 1.300327 | 0.171058 | 2.275350 |
| C | 2.748751 | -0.085248 | 4.130216 |
| H | 2.056412 | -0.797531 | 4.613390 |
| H | 3.785928 | -0.345603 | 4.391846 |
| H | 2.515921 | 0.915775 | 4.535939 |
| C | 3.199462 | 1.345117 | -2.422181 |
| H | 3.501391 | 1.096582 | -3.460710 |

| | | | |
|---|---|---|---|
| C | 2.031530 | 2.335160 | -2.485391 |
| H | 1.756002 | 2.662545 | -1.469662 |
| H | 2.314239 | 3.231378 | -3.065365 |
| H | 1.134785 | 1.889292 | -2.947335 |
| C | 4.402336 | 1.978003 | -1.703825 |
| H | 4.702054 | 2.911788 | -2.210951 |
| H | 4.134923 | 2.230148 | -0.662315 |
| H | 5.280321 | 1.310405 | -1.676090 |
| N | -1.013195 | 1.205178 | -1.549432 |
| C | -1.426281 | -1.159111 | -3.003756 |
| H | -0.784020 | -0.858357 | -3.854195 |
| H | -1.801220 | -2.176618 | -3.191222 |
| H | -2.269364 | -0.451030 | -2.969181 |
| Co | 0.572868 | 0.077440 | 0.504036 |
| C | -2.897565 | 0.840829 | 1.336378 |
| C | -3.285940 | -0.337497 | 0.639008 |
| C | -3.482671 | 2.043662 | 0.958711 |
| C | -2.573509 | -1.460431 | 1.141759 |
| C | -4.203423 | -0.377250 | -0.424011 |
| C | -4.426232 | 2.038879 | -0.114043 |
| H | -3.221584 | 2.988885 | 1.446077 |
| C | -2.809000 | -2.699033 | 0.557676 |
| C | -4.428584 | -1.665627 | -1.005576 |
| C | -4.782354 | 0.877841 | -0.796784 |
| H | -4.876924 | 2.990801 | -0.414952 |
| C | -3.747902 | -2.779499 | -0.516094 |
| H | -2.274541 | -3.598545 | 0.880083 |
| H | -5.131281 | -1.775570 | -1.839192 |
| H | -5.503794 | 0.925949 | -1.620050 |
| H | -3.927075 | -3.756493 | -0.978078 |
| C | -1.680267 | -0.939997 | 2.191447 |
| H | -0.992381 | -1.537271 | 2.790114 |

| | | | |
|---|---|---|---|
| C | -1.877448 | 0.420829 | 2.310016 |
| H | -1.356702 | 1.076667 | 3.009077 |

**Int1A**

Lowest frequency = 9.2660 cm$^{-1}$

Charge = 0, Multiplicity = 5

74

| | | | |
|---|---|---|---|
| C | 0.368684 | -1.891461 | 0.197085 |
| C | -0.347600 | -2.227891 | -0.995938 |
| C | 0.993776 | -2.935418 | 0.902498 |
| C | -0.454570 | -3.573846 | -1.428888 |
| C | 0.889966 | -4.268425 | 0.473179 |
| H | 1.567151 | -2.713155 | 1.812581 |
| C | 0.160552 | -4.587001 | -0.692722 |
| H | -1.019316 | -3.820796 | -2.334943 |
| H | 1.370539 | -5.070076 | 1.047153 |
| H | 0.080288 | -5.629444 | -1.019658 |
| C | -0.974917 | -1.121854 | -1.692714 |
| N | -0.631172 | 0.065647 | -1.194561 |
| C | -0.743100 | 2.253674 | -0.967182 |
| C | -1.146834 | 3.566331 | -1.343231 |
| N | 0.092210 | 2.061624 | 0.111251 |
| C | -0.682503 | 4.651714 | -0.619508 |
| H | -1.817544 | 3.669103 | -2.199683 |
| C | 0.535889 | 3.130657 | 0.806301 |
| C | 0.180688 | 4.437402 | 0.484218 |
| H | -0.980550 | 5.668460 | -0.896420 |
| H | 1.188784 | 2.895281 | 1.654045 |
| H | 0.562384 | 5.271782 | 1.078962 |
| N | 2.507351 | -0.076110 | -0.258092 |
| C | 3.520814 | -0.423403 | 0.517945 |

| | | | |
|---|---|---|---|
| C | 2.855550 | -0.440490 | -1.646920 |
| H | 1.991140 | -0.947218 | -2.109427 |
| C | 4.025765 | -1.419773 | -1.437725 |
| H | 4.848116 | -1.304675 | -2.160831 |
| H | 3.693714 | -2.472990 | -1.411280 |
| O | 4.531762 | -1.078292 | -0.121493 |
| C | 3.669107 | -0.213517 | 1.914975 |
| H | 4.631904 | -0.481712 | 2.353815 |
| C | 2.628022 | 0.241853 | 2.724041 |
| O | 1.423527 | 0.471151 | 2.310038 |
| C | 2.857191 | 0.469736 | 4.203876 |
| H | 2.143511 | -0.141112 | 4.784927 |
| H | 3.885461 | 0.226920 | 4.514426 |
| H | 2.647035 | 1.527002 | 4.447462 |
| C | 3.242940 | 0.788620 | -2.513259 |
| H | 3.569768 | 0.361124 | -3.483625 |
| C | 2.046485 | 1.709214 | -2.776472 |
| H | 1.734722 | 2.203721 | -1.843070 |
| H | 2.314800 | 2.495958 | -3.503523 |
| H | 1.177808 | 1.155863 | -3.170638 |
| C | 4.413942 | 1.582658 | -1.912069 |
| H | 4.701509 | 2.410587 | -2.583435 |
| H | 4.118803 | 2.020029 | -0.941797 |
| H | 5.307023 | 0.956970 | -1.742986 |
| N | -1.180071 | 1.180050 | -1.671201 |
| C | -1.931099 | -1.272532 | -2.835701 |
| H | -1.437171 | -1.764438 | -3.694054 |
| H | -2.785415 | -1.904606 | -2.535944 |
| H | -2.297587 | -0.282431 | -3.141803 |
| Co | 0.641836 | 0.108596 | 0.511679 |
| C | -2.735681 | 1.043950 | 1.329867 |
| C | -3.255854 | -0.186918 | 0.837165 |

| | | | |
|---|---|---|---|
| C | -3.346418 | 2.221773 | 0.912924 |
| C | -2.501265 | -1.277715 | 1.348456 |
| C | -4.339888 | -0.304145 | -0.050097 |
| C | -4.450066 | 2.138721 | 0.010041 |
| H | -2.990701 | 3.203158 | 1.244372 |
| C | -2.864455 | -2.562546 | 0.961576 |
| C | -4.698355 | -1.638196 | -0.423036 |
| C | -4.942098 | 0.925958 | -0.467930 |
| H | -4.921865 | 3.069905 | -0.322217 |
| C | -3.975104 | -2.720759 | 0.078464 |
| H | -2.307022 | -3.443467 | 1.296235 |
| H | -5.538596 | -1.809436 | -1.105369 |
| H | -5.789292 | 0.913499 | -1.162559 |
| H | -4.259382 | -3.734653 | -0.223580 |
| C | -1.440175 | -0.684623 | 2.183558 |
| H | -0.713212 | -1.241668 | 2.774858 |
| C | -1.591887 | 0.692997 | 2.183154 |
| H | -0.971941 | 1.390612 | 2.746959 |

**TS(1-2)A**

Lowest frequency = -282.7828 $cm^{-1}$

Charge = 0, Multiplicity = 1

74

| | | | |
|---|---|---|---|
| C | -0.037854 | -1.781873 | 0.359403 |
| C | -0.505461 | -1.924541 | -0.994709 |
| C | 0.521117 | -2.905012 | 1.000109 |
| C | -0.453056 | -3.184082 | -1.626189 |
| C | 0.602171 | -4.142413 | 0.346733 |
| H | 0.875937 | -2.816236 | 2.032143 |
| C | 0.098117 | -4.285929 | -0.959580 |

| | | | |
|---|---|---|---|
| H | -0.815265 | -3.290019 | -2.654460 |
| H | 1.042324 | -5.002018 | 0.863281 |
| H | 0.142147 | -5.259319 | -1.459696 |
| C | -0.947328 | -0.710089 | -1.642643 |
| N | -0.521909 | 0.381778 | -1.011098 |
| C | -0.160669 | 2.535059 | -0.696794 |
| C | -0.262282 | 3.928732 | -0.958167 |
| N | 0.567669 | 2.103180 | 0.395882 |
| C | 0.386926 | 4.827221 | -0.122926 |
| H | -0.853737 | 4.249664 | -1.818662 |
| C | 1.179072 | 2.983150 | 1.209848 |
| C | 1.130472 | 4.357389 | 0.984779 |
| H | 0.319681 | 5.902265 | -0.321822 |
| H | 1.711087 | 2.536741 | 2.056239 |
| H | 1.655878 | 5.042637 | 1.654754 |
| N | 2.140732 | -0.307913 | -0.298944 |
| C | 3.008448 | -1.051842 | 0.364514 |
| C | 2.443146 | -0.463639 | -1.743447 |
| H | 1.503987 | -0.564398 | -2.309479 |
| C | 3.229648 | -1.782465 | -1.744708 |
| H | 4.049927 | -1.822212 | -2.477486 |
| H | 2.573752 | -2.665050 | -1.855561 |
| O | 3.812531 | -1.825784 | -0.414667 |
| C | 3.205116 | -1.126025 | 1.767496 |
| H | 4.049340 | -1.717280 | 2.124716 |
| C | 2.364481 | -0.472286 | 2.659521 |
| O | 1.269704 | 0.139999 | 2.333938 |
| C | 2.674620 | -0.451504 | 4.140920 |
| H | 1.815906 | -0.852466 | 4.708145 |
| H | 3.579120 | -1.030638 | 4.382725 |
| H | 2.818839 | 0.593373 | 4.470410 |
| C | 3.271821 | 0.709205 | -2.341961 |

| | | | |
|---|---|---|---|
| H | 3.508845 | 0.372676 | -3.372721 |
| C | 2.480641 | 2.016652 | -2.449330 |
| H | 2.328563 | 2.458411 | -1.451818 |
| H | 3.037005 | 2.750811 | -3.058595 |
| H | 1.488545 | 1.863157 | -2.905285 |
| C | 4.595401 | 0.945202 | -1.595767 |
| H | 5.170684 | 1.749884 | -2.086340 |
| H | 4.395295 | 1.259268 | -0.555981 |
| H | 5.230696 | 0.044314 | -1.560590 |
| N | -0.728250 | 1.608302 | -1.501444 |
| C | -1.741572 | -0.609958 | -2.907089 |
| H | -1.093213 | -0.391253 | -3.777570 |
| H | -2.292520 | -1.543423 | -3.100249 |
| H | -2.453516 | 0.227293 | -2.820439 |
| Co | 0.416865 | 0.178494 | 0.592895 |
| C | -2.466244 | 1.068373 | 1.025574 |
| C | -3.211554 | -0.062663 | 0.586500 |
| C | -2.980669 | 2.333256 | 0.771565 |
| C | -2.575032 | -1.274379 | 0.946924 |
| C | -4.442697 | 0.010734 | -0.108636 |
| C | -4.219975 | 2.439024 | 0.070049 |
| H | -2.453122 | 3.239726 | 1.083812 |
| C | -3.184356 | -2.478825 | 0.618627 |
| C | -5.047860 | -1.238979 | -0.432656 |
| C | -4.936204 | 1.328567 | -0.368311 |
| H | -4.618952 | 3.439084 | -0.132543 |
| C | -4.431037 | -2.437637 | -0.066165 |
| H | -2.711999 | -3.440003 | 0.845990 |
| H | -6.006157 | -1.259165 | -0.963794 |
| H | -5.885861 | 1.461019 | -0.898229 |
| H | -4.920280 | -3.384162 | -0.320909 |
| C | -1.270040 | -0.913526 | 1.594141 |

| | | | |
|---|---|---|---|
| H | -0.917214 | -1.478592 | 2.461040 |
| C | -1.248180 | 0.555852 | 1.681333 |
| H | -0.811395 | 1.050913 | 2.552100 |

**TS(1-2)A**

Lowest frequency = -333.5821 $cm^{-1}$

Charge = 0, Multiplicity = 3

74

| | | | |
|---|---|---|---|
| C | 0.186191 | -1.790396 | 0.341751 |
| C | -0.320964 | -2.051292 | -0.981235 |
| C | 0.911897 | -2.804186 | 1.003004 |
| C | -0.157755 | -3.333639 | -1.549612 |
| C | 1.122330 | -4.047065 | 0.393243 |
| H | 1.311008 | -2.620756 | 2.006325 |
| C | 0.566389 | -4.321705 | -0.872863 |
| H | -0.567976 | -3.537098 | -2.544715 |
| H | 1.705413 | -4.813562 | 0.915237 |
| H | 0.709430 | -5.305738 | -1.331764 |
| C | -0.936702 | -0.933467 | -1.663293 |
| N | -0.596214 | 0.226800 | -1.111403 |
| C | -0.587203 | 2.412410 | -0.811136 |
| C | -0.895621 | 3.768091 | -1.129946 |
| N | 0.161330 | 2.137943 | 0.318784 |
| C | -0.456466 | 4.782520 | -0.297334 |
| H | -1.486078 | 3.958842 | -2.029253 |
| C | 0.566984 | 3.134415 | 1.132681 |
| C | 0.292191 | 4.471410 | 0.867093 |
| H | -0.689609 | 5.825886 | -0.536015 |
| H | 1.138368 | 2.811377 | 2.010260 |
| H | 0.656530 | 5.252827 | 1.539029 |

| | | | |
|---|---|---|---|
| N | 2.249829 | -0.059978 | -0.297258 |
| C | 3.228303 | -0.679575 | 0.361204 |
| C | 2.516540 | -0.243069 | -1.744060 |
| H | 1.571530 | -0.468965 | -2.260784 |
| C | 3.450089 | -1.461224 | -1.736473 |
| H | 4.245795 | -1.431079 | -2.496363 |
| H | 2.897135 | -2.415944 | -1.799072 |
| O | 4.076409 | -1.393973 | -0.427724 |
| C | 3.481935 | -0.686370 | 1.751276 |
| H | 4.396506 | -1.179009 | 2.085991 |
| C | 2.584466 | -0.139853 | 2.675807 |
| O | 1.424829 | 0.320030 | 2.365123 |
| C | 2.938060 | -0.097809 | 4.148786 |
| H | 2.962411 | 0.953928 | 4.487020 |
| H | 2.150501 | -0.605762 | 4.733113 |
| H | 3.913048 | -0.563726 | 4.361179 |
| C | 3.165473 | 1.006948 | -2.402370 |
| H | 3.385402 | 0.688967 | -3.442605 |
| C | 2.201855 | 2.196767 | -2.465349 |
| H | 2.014827 | 2.593600 | -1.454463 |
| H | 2.634270 | 3.010514 | -3.073969 |
| H | 1.229395 | 1.913861 | -2.901262 |
| C | 4.487508 | 1.416419 | -1.732993 |
| H | 4.935407 | 2.272531 | -2.267311 |
| H | 4.307601 | 1.727958 | -0.688705 |
| H | 5.227793 | 0.598683 | -1.719518 |
| N | -1.028862 | 1.398905 | -1.588390 |
| C | -1.823274 | -1.034067 | -2.866564 |
| H | -1.276821 | -1.446502 | -3.736014 |
| H | -2.678074 | -1.702342 | -2.659569 |
| H | -2.194991 | -0.031183 | -3.122818 |
| Co | 0.478687 | 0.185296 | 0.573090 |

| | | | |
|---|---|---|---|
| C | -2.467441 | 0.819168 | 1.168834 |
| C | -3.162772 | -0.320819 | 0.677567 |
| C | -3.032173 | 2.073768 | 0.968411 |
| C | -2.483496 | -1.520628 | 1.011218 |
| C | -4.378628 | -0.266551 | -0.041361 |
| C | -4.263857 | 2.159813 | 0.254622 |
| H | -2.546546 | 2.986120 | 1.328583 |
| C | -3.033755 | -2.733926 | 0.605838 |
| C | -4.922313 | -1.524418 | -0.440223 |
| C | -4.921425 | 1.040371 | -0.250611 |
| H | -4.702223 | 3.150235 | 0.090971 |
| C | -4.260395 | -2.711368 | -0.114563 |
| H | -2.534247 | -3.686359 | 0.809856 |
| H | -5.865933 | -1.560060 | -0.995966 |
| H | -5.863872 | 1.158197 | -0.796656 |
| H | -4.699724 | -3.664602 | -0.428618 |
| C | -1.232811 | -1.127119 | 1.700532 |
| H | -0.787388 | -1.732016 | 2.491304 |
| C | -1.229133 | 0.327695 | 1.799555 |
| H | -0.740073 | 0.836538 | 2.633209 |

**TS(1-2)A**

Lowest frequency = -279.0365 cm$^{-1}$

Charge = 0, Multiplicity = 5

74

| | | | |
|---|---|---|---|
| C | 0.326036 | -1.768704 | 0.476169 |
| C | -0.031193 | -2.073818 | -0.888356 |
| C | 1.126615 | -2.697023 | 1.179325 |
| C | 0.368747 | -3.301515 | -1.470602 |
| C | 1.588453 | -3.862121 | 0.560586 |

| | | | |
|---|---|---|---|
| H | 1.394175 | -2.503354 | 2.224704 |
| C | 1.187777 | -4.178257 | -0.758104 |
| H | 0.062956 | -3.543852 | -2.494516 |
| H | 2.244791 | -4.547144 | 1.109112 |
| H | 1.522559 | -5.112991 | -1.220335 |
| C | -0.741093 | -1.036689 | -1.592019 |
| N | -0.591195 | 0.164481 | -1.014451 |
| C | -0.977836 | 2.316278 | -0.729290 |
| C | -1.557520 | 3.573524 | -1.049986 |
| N | -0.160655 | 2.193370 | 0.370953 |
| C | -1.298810 | 4.668400 | -0.237383 |
| H | -2.203431 | 3.632521 | -1.929283 |
| C | 0.075750 | 3.261491 | 1.155525 |
| C | -0.467554 | 4.519603 | 0.897132 |
| H | -1.741550 | 5.642500 | -0.471297 |
| H | 0.729495 | 3.075353 | 2.015982 |
| H | -0.248637 | 5.361258 | 1.559804 |
| N | 2.385662 | 0.140295 | -0.369094 |
| C | 3.404314 | -0.392503 | 0.291927 |
| C | 2.668025 | 0.015639 | -1.812682 |
| H | 1.762765 | -0.355923 | -2.322069 |
| C | 3.783422 | -1.046664 | -1.830797 |
| H | 4.591836 | -0.842597 | -2.549859 |
| H | 3.388649 | -2.066635 | -1.985815 |
| O | 4.344526 | -0.985975 | -0.493919 |
| C | 3.623324 | -0.417993 | 1.694373 |
| H | 4.570477 | -0.841995 | 2.032517 |
| C | 2.685438 | 0.032595 | 2.625702 |
| O | 1.508012 | 0.471914 | 2.315644 |
| C | 3.002582 | 0.009561 | 4.105144 |
| H | 2.253186 | -0.607691 | 4.632567 |
| H | 4.010444 | -0.381980 | 4.312992 |

| | | | |
|---|---|---|---|
| H | 2.920323 | 1.032898 | 4.513058 |
| C | 3.081667 | 1.367489 | -2.452393 |
| H | 3.342155 | 1.122392 | -3.502691 |
| C | 1.914333 | 2.361666 | -2.465925 |
| H | 1.657689 | 2.662382 | -1.437075 |
| H | 2.184062 | 3.271922 | -3.029756 |
| H | 1.009437 | 1.925526 | -2.921855 |
| C | 4.316116 | 1.981758 | -1.773727 |
| H | 4.617693 | 2.909200 | -2.291016 |
| H | 4.086729 | 2.238366 | -0.724299 |
| H | 5.181742 | 1.297073 | -1.773194 |
| N | -1.245056 | 1.218037 | -1.488516 |
| C | -1.571091 | -1.195963 | -2.825428 |
| H | -1.181395 | -0.563308 | -3.643436 |
| H | -1.620204 | -2.244226 | -3.153734 |
| H | -2.593357 | -0.835043 | -2.613828 |
| Co | 0.621159 | 0.290939 | 0.587994 |
| C | -2.744546 | 0.519176 | 1.313016 |
| C | -3.220705 | -0.655324 | 0.638129 |
| C | -3.451107 | 1.715454 | 1.112721 |
| C | -2.417431 | -1.775821 | 0.959662 |
| C | -4.337386 | -0.690887 | -0.222163 |
| C | -4.572412 | 1.704511 | 0.240956 |
| H | -3.143716 | 2.648638 | 1.595611 |
| C | -2.749048 | -3.012808 | 0.407995 |
| C | -4.653729 | -1.967986 | -0.774158 |
| C | -5.016488 | 0.555877 | -0.420989 |
| H | -5.110591 | 2.644993 | 0.077071 |
| C | -3.878044 | -3.086501 | -0.452726 |
| H | -2.148596 | -3.907319 | 0.603008 |
| H | -5.510249 | -2.074395 | -1.449500 |
| H | -5.890302 | 0.604950 | -1.079675 |

| | | | |
|---|---|---|---|
| H | -4.144389 | -4.057004 | -0.885870 |
| C | -1.304027 | -1.268594 | 1.803099 |
| H | -0.798586 | -1.887195 | 2.547524 |
| C | -1.577404 | 0.126881 | 2.053751 |
| H | -1.020104 | 0.742166 | 2.762375 |

**Int2A**

Lowest frequency = 31.6095 cm$^{-1}$

Charge = 0, Multiplicity = 1

74

| | | | |
|---|---|---|---|
| C | 0.102322 | -2.095954 | 0.691108 |
| C | 0.146721 | -2.177526 | -0.745750 |
| C | 1.071997 | -2.808724 | 1.430131 |
| C | 1.018647 | -3.100593 | -1.356861 |
| C | 2.003029 | -3.640572 | 0.800301 |
| H | 1.056970 | -2.738780 | 2.523384 |
| C | 1.940919 | -3.824317 | -0.591984 |
| H | 1.016354 | -3.195054 | -2.448317 |
| H | 2.750011 | -4.176015 | 1.394271 |
| H | 2.634308 | -4.514836 | -1.084097 |
| C | -0.617626 | -1.208042 | -1.531338 |
| N | -0.550564 | 0.016868 | -1.028171 |
| C | -0.956680 | 2.167331 | -0.881497 |
| C | -1.518818 | 3.428243 | -1.218226 |
| N | -0.144393 | 2.072498 | 0.242785 |
| C | -1.253476 | 4.532331 | -0.422961 |
| H | -2.162769 | 3.479198 | -2.098802 |
| C | 0.090414 | 3.148396 | 1.027138 |
| C | -0.434543 | 4.400014 | 0.724702 |
| H | -1.685384 | 5.505587 | -0.678737 |

| | | | |
|---|---|---|---|
| H | 0.716884 | 2.952596 | 1.901856 |
| H | -0.206946 | 5.254044 | 1.367582 |
| N | 2.224622 | 0.041226 | -0.334224 |
| C | 3.248277 | -0.393704 | 0.382362 |
| C | 2.623662 | -0.021635 | -1.757466 |
| H | 1.793242 | -0.435204 | -2.354681 |
| C | 3.816849 | -0.995808 | -1.713869 |
| H | 4.655228 | -0.699702 | -2.363602 |
| H | 3.527564 | -2.038389 | -1.927903 |
| O | 4.270170 | -0.930752 | -0.339529 |
| C | 3.414480 | -0.331643 | 1.788563 |
| H | 4.351747 | -0.707530 | 2.200818 |
| C | 2.465590 | 0.271159 | 2.607352 |
| O | 1.301446 | 0.681669 | 2.214905 |
| C | 2.743634 | 0.489980 | 4.078907 |
| H | 1.950121 | 0.014236 | 4.682212 |
| H | 3.724084 | 0.089508 | 4.379665 |
| H | 2.713513 | 1.571861 | 4.301257 |
| C | 3.031244 | 1.358383 | -2.347474 |
| H | 3.438889 | 1.117397 | -3.351244 |
| C | 1.843323 | 2.306212 | -2.538963 |
| H | 1.485728 | 2.677092 | -1.566069 |
| H | 2.144568 | 3.182046 | -3.140386 |
| H | 0.995854 | 1.812052 | -3.042673 |
| C | 4.136906 | 2.039349 | -1.524541 |
| H | 4.449677 | 2.981367 | -2.007681 |
| H | 3.762081 | 2.283910 | -0.514976 |
| H | 5.031408 | 1.403802 | -1.407886 |
| N | -1.173649 | 1.053982 | -1.609458 |
| C | -1.373610 | -1.489783 | -2.792385 |
| H | -1.068889 | -0.801487 | -3.600559 |
| H | -1.234230 | -2.534183 | -3.110752 |

| | | | |
|---|---|---|---|
| H | -2.452047 | -1.318582 | -2.616960 |
| Co | 0.445957 | 0.336165 | 0.522337 |
| C | -2.523991 | 0.434712 | 1.149219 |
| C | -3.194238 | -0.669009 | 0.550111 |
| C | -3.179280 | 1.663276 | 1.173929 |
| C | -2.444178 | -1.861313 | 0.654050 |
| C | -4.464143 | -0.588099 | -0.079306 |
| C | -4.459775 | 1.770088 | 0.559852 |
| H | -2.721273 | 2.546304 | 1.631166 |
| C | -2.949903 | -3.028727 | 0.098807 |
| C | -4.962862 | -1.801219 | -0.641511 |
| C | -5.090778 | 0.695232 | -0.062929 |
| H | -4.962346 | 2.743881 | 0.571150 |
| C | -4.218060 | -2.976355 | -0.549453 |
| H | -2.393227 | -3.971193 | 0.138706 |
| H | -5.936070 | -1.807673 | -1.145078 |
| H | -6.072426 | 0.828812 | -0.530729 |
| H | -4.621526 | -3.896187 | -0.987744 |
| C | -1.155689 | -1.554661 | 1.414052 |
| H | -1.174310 | -2.053812 | 2.401434 |
| C | -1.193385 | -0.017462 | 1.608770 |
| H | -0.854525 | 0.351764 | 2.585700 |

**Int2A**

Lowest frequency = 25.3006 $cm^{-1}$

Charge = 0, Multiplicity = 3

74

| | | | |
|---|---|---|---|
| C | -0.774275 | 2.603101 | 0.825643 |
| C | -0.992552 | 2.437332 | -0.568614 |
| C | -1.726519 | 3.301128 | 1.587302 |

| | | | |
|---|---|---|---|
| C | -2.114082 | 3.069945 | -1.157108 |
| C | -2.867739 | 3.865011 | 1.002216 |
| H | -1.550168 | 3.424902 | 2.662166 |
| C | -3.050153 | 3.764959 | -0.385166 |
| H | -2.262398 | 2.986529 | -2.239396 |
| H | -3.592751 | 4.404609 | 1.620255 |
| H | -3.918457 | 4.228810 | -0.865300 |
| C | -0.055010 | 1.697148 | -1.426335 |
| N | 0.408753 | 0.508002 | -1.062922 |
| C | 1.690674 | -1.272437 | -1.552776 |
| C | 2.743806 | -1.959376 | -2.227828 |
| N | 0.968476 | -1.928189 | -0.578446 |
| C | 3.021109 | -3.272973 | -1.892426 |
| H | 3.304719 | -1.415764 | -2.991556 |
| C | 1.254536 | -3.208121 | -0.255816 |
| C | 2.264215 | -3.924676 | -0.885619 |
| H | 3.829956 | -3.807731 | -2.402214 |
| H | 0.642977 | -3.629478 | 0.548728 |
| H | 2.464043 | -4.960953 | -0.600780 |
| N | -2.108333 | -0.623756 | -0.069309 |
| C | -3.007389 | -0.863112 | 0.879585 |
| C | -2.843994 | -0.257861 | -1.297901 |
| H | -2.346934 | 0.595023 | -1.782360 |
| C | -4.211397 | 0.158547 | -0.730788 |
| H | -5.070784 | -0.176347 | -1.332148 |
| H | -4.275544 | 1.245275 | -0.548019 |
| O | -4.278056 | -0.515399 | 0.554712 |
| C | -2.803041 | -1.471530 | 2.143473 |
| H | -3.674318 | -1.579043 | 2.791157 |
| C | -1.579197 | -2.035092 | 2.500239 |
| O | -0.493708 | -1.925860 | 1.803110 |
| C | -1.442914 | -2.852244 | 3.766053 |

| | | | |
|---|---|---|---|
| H | -0.667030 | -2.406901 | 4.413846 |
| H | -2.390742 | -2.918354 | 4.321917 |
| H | -1.103503 | -3.871271 | 3.507808 |
| C | -2.913518 | -1.424949 | -2.318178 |
| H | -3.605637 | -1.071156 | -3.110241 |
| C | -1.544305 | -1.685063 | -2.957163 |
| H | -0.831758 | -2.047620 | -2.199287 |
| H | -1.620976 | -2.450032 | -3.749671 |
| H | -1.121081 | -0.767860 | -3.401658 |
| C | -3.490856 | -2.709845 | -1.704379 |
| H | -3.596834 | -3.491965 | -2.476093 |
| H | -2.815240 | -3.096005 | -0.920935 |
| H | -4.482060 | -2.546989 | -1.246669 |
| N | 1.387467 | 0.000857 | -1.852202 |
| C | 0.430128 | 2.295179 | -2.719023 |
| H | 0.258433 | 1.604478 | -3.563969 |
| H | -0.049944 | 3.266026 | -2.913376 |
| H | 1.525170 | 2.433161 | -2.658581 |
| Co | -0.190595 | -0.656123 | 0.384679 |
| C | 1.885779 | 0.126920 | 1.637329 |
| C | 2.595503 | 1.151823 | 0.936005 |
| C | 2.560763 | -1.066177 | 1.929304 |
| C | 1.811029 | 2.313534 | 0.766576 |
| C | 3.917619 | 1.007729 | 0.446663 |
| C | 3.889315 | -1.224821 | 1.457421 |
| H | 2.055244 | -1.879942 | 2.455644 |
| C | 2.343715 | 3.385673 | 0.063746 |
| C | 4.441883 | 2.121822 | -0.272795 |
| C | 4.557018 | -0.239276 | 0.725139 |
| H | 4.403931 | -2.170451 | 1.659888 |
| C | 3.665798 | 3.265788 | -0.455358 |
| H | 1.769629 | 4.303273 | -0.104025 |

| | | | |
|---|---|---|---|
| H | 5.455464 | 2.075336 | -0.686074 |
| H | 5.575447 | -0.421165 | 0.365166 |
| H | 4.086953 | 4.108619 | -1.014889 |
| C | 0.485522 | 2.095787 | 1.494533 |
| H | 0.559763 | 2.624711 | 2.468305 |
| C | 0.499999 | 0.586053 | 1.811467 |
| H | -0.078764 | 0.275720 | 2.691087 |

**Int2A**

Lowest frequency = 27.7391 $cm^{-1}$

Charge = 0, Multiplicity = 5

74

| | | | |
|---|---|---|---|
| C | -0.776620 | 2.500075 | 0.914334 |
| C | -0.937443 | 2.480990 | -0.501520 |
| C | -1.807241 | 3.045510 | 1.701407 |
| C | -2.071974 | 3.116927 | -1.063156 |
| C | -2.959424 | 3.602434 | 1.133766 |
| H | -1.682680 | 3.058454 | 2.790372 |
| C | -3.079693 | 3.661553 | -0.263112 |
| H | -2.173382 | 3.144714 | -2.153585 |
| H | -3.741149 | 4.019912 | 1.776713 |
| H | -3.955114 | 4.129129 | -0.726245 |
| C | 0.032559 | 1.845065 | -1.405068 |
| N | 0.494673 | 0.641177 | -1.091005 |
| C | 1.809630 | -1.114847 | -1.564219 |
| C | 2.926126 | -1.734288 | -2.199686 |
| N | 1.073310 | -1.825463 | -0.644232 |
| C | 3.247867 | -3.045461 | -1.889905 |
| H | 3.500001 | -1.139983 | -2.914821 |
| C | 1.404458 | -3.098595 | -0.342407 |

| | | | |
|---|---|---|---|
| C | 2.473656 | -3.756309 | -0.941275 |
| H | 4.105044 | -3.527617 | -2.372466 |
| H | 0.775119 | -3.573595 | 0.418316 |
| H | 2.702805 | -4.791598 | -0.675190 |
| N | -2.165582 | -0.639821 | -0.158267 |
| C | -3.096358 | -0.875459 | 0.759882 |
| C | -2.845222 | -0.232990 | -1.403636 |
| H | -2.325880 | 0.640162 | -1.828707 |
| C | -4.242414 | 0.160810 | -0.888842 |
| H | -5.070744 | -0.188475 | -1.524991 |
| H | -4.332537 | 1.246532 | -0.712476 |
| O | -4.352154 | -0.510428 | 0.393306 |
| C | -2.946736 | -1.496171 | 2.027238 |
| H | -3.845232 | -1.575619 | 2.641723 |
| C | -1.754096 | -2.101373 | 2.435634 |
| O | -0.653127 | -2.051399 | 1.762562 |
| C | -1.699391 | -2.884232 | 3.730575 |
| H | -0.927027 | -2.449861 | 4.390328 |
| H | -2.665656 | -2.895975 | 4.258501 |
| H | -1.391010 | -3.922714 | 3.514369 |
| C | -2.853537 | -1.362074 | -2.467103 |
| H | -3.507543 | -0.987661 | -3.281663 |
| C | -1.449883 | -1.589031 | -3.042352 |
| H | -0.770455 | -1.966235 | -2.260566 |
| H | -1.476472 | -2.331086 | -3.859497 |
| H | -1.014960 | -0.654839 | -3.437603 |
| C | -3.448937 | -2.669782 | -1.922923 |
| H | -3.511379 | -3.428199 | -2.722452 |
| H | -2.809104 | -3.074672 | -1.119184 |
| H | -4.462890 | -2.528028 | -1.509582 |
| N | 1.477227 | 0.159051 | -1.875015 |
| C | 0.504340 | 2.520869 | -2.661328 |

| | | | |
|---|---|---|---|
| H | 0.260032 | 1.911467 | -3.550917 |
| H | 0.078716 | 3.530284 | -2.765072 |
| H | 1.607147 | 2.580195 | -2.632790 |
| Co | -0.253338 | -0.684250 | 0.352020 |
| C | 1.875456 | 0.067035 | 1.776662 |
| C | 2.589324 | 1.078815 | 1.046159 |
| C | 2.548652 | -1.146100 | 2.049028 |
| C | 1.817875 | 2.246612 | 0.889882 |
| C | 3.890761 | 0.907885 | 0.514212 |
| C | 3.850313 | -1.324692 | 1.532950 |
| H | 2.049579 | -1.950756 | 2.595852 |
| C | 2.346702 | 3.312232 | 0.166831 |
| C | 4.407584 | 2.008386 | -0.229769 |
| C | 4.515971 | -0.350754 | 0.772266 |
| H | 4.357089 | -2.277986 | 1.719406 |
| C | 3.647281 | 3.168669 | -0.390407 |
| H | 1.781107 | 4.237117 | 0.010995 |
| H | 5.404387 | 1.941220 | -0.679486 |
| H | 5.516813 | -0.555103 | 0.377654 |
| H | 4.066216 | 4.001132 | -0.966841 |
| C | 0.494765 | 2.038045 | 1.619270 |
| H | 0.544859 | 2.623288 | 2.562788 |
| C | 0.541472 | 0.551572 | 2.012784 |
| H | -0.095670 | 0.193196 | 2.828229 |

**Int1B**

Lowest frequency = 31.6083 $cm^{-1}$

Charge = 0, Multiplicity = 1

74

| | | | |
|---|---|---|---|
| C | 0.506081 | -1.860696 | -0.569402 |

| | | | |
|---|---|---|---|
| C | 1.484847 | -2.041666 | -1.594312 |
| C | 0.044356 | -2.970943 | 0.138742 |
| C | 1.986291 | -3.331517 | -1.874883 |
| C | 0.554631 | -4.255501 | -0.142974 |
| H | -0.709908 | -2.851178 | 0.922539 |
| C | 1.521617 | -4.434716 | -1.144400 |
| H | 2.742172 | -3.468475 | -2.656441 |
| H | 0.190212 | -5.118393 | 0.426284 |
| H | 1.911845 | -5.435331 | -1.358901 |
| C | 1.913981 | -0.822453 | -2.261445 |
| N | 1.377453 | 0.269025 | -1.726470 |
| C | 1.143936 | 2.444639 | -1.321212 |
| C | 1.347254 | 3.838545 | -1.529577 |
| N | 0.389796 | 2.031212 | -0.241341 |
| C | 0.827977 | 4.744720 | -0.615149 |
| H | 1.925813 | 4.154528 | -2.400858 |
| C | -0.115756 | 2.915221 | 0.632387 |
| C | 0.089518 | 4.288542 | 0.502098 |
| H | 0.993853 | 5.817770 | -0.760750 |
| H | -0.710534 | 2.479040 | 1.442080 |
| H | -0.322197 | 4.981434 | 1.240296 |
| N | 1.604447 | -0.212547 | 1.057750 |
| C | 1.321445 | -0.909541 | 2.154571 |
| C | 3.071195 | -0.324291 | 0.838962 |
| H | 3.276051 | -0.465558 | -0.232717 |
| C | 3.393542 | -1.594408 | 1.634945 |
| H | 4.375514 | -1.583622 | 2.131405 |
| H | 3.275512 | -2.514273 | 1.034103 |
| O | 2.369992 | -1.602979 | 2.667751 |
| C | 0.087935 | -1.001632 | 2.837076 |
| H | 0.071794 | -1.533312 | 3.789216 |
| C | -1.071656 | -0.462672 | 2.288390 |

| | | | |
|---|---|---|---|
| O | -1.140552 | 0.040396 | 1.101951 |
| C | -2.379029 | -0.469269 | 3.046249 |
| H | -3.099515 | -1.124012 | 2.524547 |
| H | -2.252631 | -0.812806 | 4.084390 |
| H | -2.811079 | 0.546308 | 3.040374 |
| C | 3.887548 | 0.896527 | 1.356118 |
| H | 4.942769 | 0.570464 | 1.246277 |
| C | 3.706644 | 2.162462 | 0.513392 |
| H | 2.720158 | 2.614846 | 0.699365 |
| H | 4.471869 | 2.911156 | 0.784619 |
| H | 3.787273 | 1.955403 | -0.566511 |
| C | 3.632285 | 1.204326 | 2.840981 |
| H | 4.270602 | 2.043244 | 3.169341 |
| H | 2.579752 | 1.502788 | 2.992322 |
| H | 3.837594 | 0.341876 | 3.496756 |
| N | 1.685621 | 1.513099 | -2.137972 |
| C | 2.904656 | -0.753531 | -3.383081 |
| H | 3.921222 | -1.011005 | -3.027088 |
| H | 2.645886 | -1.472685 | -4.180341 |
| H | 2.927025 | 0.266492 | -3.793297 |
| Co | 0.173397 | 0.043792 | -0.293664 |
| C | -2.598795 | -1.167057 | -1.003311 |
| C | -3.296599 | -0.003063 | -0.584750 |
| C | -3.143434 | -2.409348 | -0.704395 |
| C | -2.601791 | 1.174007 | -0.974413 |
| C | -4.510812 | -0.012899 | 0.131702 |
| C | -4.380846 | -2.453809 | 0.006427 |
| H | -2.645288 | -3.339176 | -0.994987 |
| C | -3.141205 | 2.407135 | -0.630967 |
| C | -5.045439 | 1.269367 | 0.470320 |
| C | -5.052703 | -1.303668 | 0.420542 |
| H | -4.816835 | -3.432563 | 0.234473 |

| | | | |
|---|---|---|---|
| C | -4.371574 | 2.430316 | 0.093157 |
| H | -2.645566 | 3.346210 | -0.896111 |
| H | -5.990813 | 1.339699 | 1.019774 |
| H | -6.000156 | -1.389982 | 0.964218 |
| H | -4.802436 | 3.401931 | 0.358722 |
| C | -1.388772 | 0.724307 | -1.678393 |
| H | -0.828492 | 1.342056 | -2.382151 |
| C | -1.377934 | -0.694476 | -1.694346 |
| H | -0.846305 | -1.287869 | -2.437696 |

**Int1B**

Lowest frequency = 10.3722 cm$^{-1}$

Charge = 0, Multiplicity = 3

74

| | | | |
|---|---|---|---|
| C | 0.621475 | -1.770987 | -0.718158 |
| C | 1.498131 | -1.880541 | -1.847892 |
| C | 0.142270 | -2.939193 | -0.111203 |
| C | 1.885480 | -3.151991 | -2.330803 |
| C | 0.536108 | -4.199883 | -0.594801 |
| H | -0.529246 | -2.875850 | 0.750652 |
| C | 1.403889 | -4.303897 | -1.699128 |
| H | 2.553820 | -3.235717 | -3.195258 |
| H | 0.170118 | -5.109309 | -0.104185 |
| H | 1.700818 | -5.291833 | -2.067566 |
| C | 1.871317 | -0.612328 | -2.433368 |
| N | 1.355066 | 0.413770 | -1.750710 |
| C | 0.837586 | 2.521319 | -1.335696 |
| C | 0.804350 | 3.927948 | -1.561590 |
| N | 0.172219 | 1.990306 | -0.242010 |
| C | 0.117258 | 4.741114 | -0.674382 |

| | | | |
|---|---|---|---|
| H | 1.327902 | 4.324165 | -2.434828 |
| C | -0.507835 | 2.793560 | 0.601458 |
| C | -0.554311 | 4.173709 | 0.437461 |
| H | 0.092924 | 5.824411 | -0.834741 |
| H | -1.017321 | 2.277635 | 1.421312 |
| H | -1.097002 | 4.794502 | 1.155034 |
| N | 1.978992 | -0.176559 | 1.149540 |
| C | 1.763508 | -0.888581 | 2.245923 |
| C | 3.425551 | -0.226032 | 0.858237 |
| H | 3.573190 | -0.427791 | -0.216412 |
| C | 3.886618 | -1.418408 | 1.715795 |
| H | 4.859082 | -1.272373 | 2.210876 |
| H | 3.892427 | -2.368039 | 1.151061 |
| O | 2.867180 | -1.516569 | 2.743361 |
| C | 0.541020 | -1.084563 | 2.938971 |
| H | 0.593852 | -1.613235 | 3.892226 |
| C | -0.697435 | -0.707661 | 2.416625 |
| O | -0.872398 | -0.203968 | 1.238703 |
| C | -1.966519 | -0.914056 | 3.211462 |
| H | -2.640724 | -1.589442 | 2.656491 |
| H | -1.766798 | -1.325338 | 4.212914 |
| H | -2.496830 | 0.049539 | 3.309296 |
| C | 4.147305 | 1.100298 | 1.224212 |
| H | 5.220628 | 0.908707 | 1.017775 |
| C | 3.687735 | 2.264339 | 0.339784 |
| H | 2.645521 | 2.532737 | 0.577015 |
| H | 4.315292 | 3.155773 | 0.515651 |
| H | 3.732800 | 2.013707 | -0.733285 |
| C | 3.993602 | 1.461715 | 2.710623 |
| H | 4.561138 | 2.380168 | 2.941322 |
| H | 2.931996 | 1.651757 | 2.948763 |
| H | 4.353771 | 0.663301 | 3.381652 |

| | | | |
|---|---|---|---|
| N | 1.489644 | 1.684198 | -2.168330 |
| C | 2.691999 | -0.426116 | -3.671305 |
| H | 3.727892 | -0.781336 | -3.512538 |
| H | 2.272915 | -1.011424 | -4.509865 |
| H | 2.715004 | 0.639150 | -3.942528 |
| Co | 0.423379 | 0.038038 | -0.127773 |
| C | -2.899049 | -1.273130 | -1.017185 |
| C | -3.516192 | -0.119730 | -0.459623 |
| C | -3.308398 | -2.519052 | -0.557001 |
| C | -2.968364 | 1.057299 | -1.041039 |
| C | -4.510424 | -0.139591 | 0.531541 |
| C | -4.326069 | -2.577619 | 0.445141 |
| H | -2.866835 | -3.445925 | -0.937800 |
| C | -3.453585 | 2.285177 | -0.605756 |
| C | -4.985608 | 1.139037 | 0.967638 |
| C | -4.918192 | -1.435477 | 0.982711 |
| H | -4.650128 | -3.561166 | 0.802835 |
| C | -4.464274 | 2.302633 | 0.405540 |
| H | -3.076498 | 3.230020 | -1.010060 |
| H | -5.761321 | 1.202393 | 1.739007 |
| H | -5.693061 | -1.530170 | 1.751699 |
| H | -4.841964 | 3.272538 | 0.747616 |
| C | -1.954322 | 0.602606 | -2.006178 |
| H | -1.351614 | 1.251032 | -2.645394 |
| C | -1.913186 | -0.777211 | -1.992035 |
| H | -1.281337 | -1.405550 | -2.620678 |

**Int1B**

Lowest frequency = 14.0560 cm$^{-1}$

Charge = 0, Multiplicity = 5

74

| | | | |
|---|---|---|---|
| C | 0.932689 | -1.739828 | -0.866247 |
| C | 1.830670 | -1.646707 | -1.978337 |
| C | 0.725002 | -3.005176 | -0.288859 |
| C | 2.456243 | -2.806492 | -2.505034 |
| C | 1.349351 | -4.150632 | -0.807442 |
| H | 0.057409 | -3.106018 | 0.576953 |
| C | 2.212865 | -4.049493 | -1.919430 |
| H | 3.133767 | -2.730197 | -3.363263 |
| H | 1.164296 | -5.131669 | -0.353137 |
| H | 2.694465 | -4.947752 | -2.320883 |
| C | 2.035083 | -0.316084 | -2.525036 |
| N | 1.466404 | 0.637862 | -1.791951 |
| C | 0.767938 | 2.677258 | -1.324530 |
| C | 0.675468 | 4.085128 | -1.521530 |
| N | 0.133165 | 2.083015 | -0.253603 |
| C | -0.044089 | 4.850777 | -0.618579 |
| H | 1.186004 | 4.516885 | -2.386052 |
| C | -0.566570 | 2.842921 | 0.615520 |
| C | -0.682149 | 4.223253 | 0.479929 |
| H | -0.119769 | 5.934913 | -0.753887 |
| H | -1.055717 | 2.291563 | 1.424631 |
| H | -1.262638 | 4.798102 | 1.206593 |
| N | 1.740243 | -0.218177 | 1.388539 |
| C | 1.431920 | -0.861920 | 2.504287 |
| C | 3.206536 | -0.276999 | 1.220106 |
| H | 3.434163 | -0.554670 | 0.176333 |
| C | 3.595519 | -1.407438 | 2.191701 |
| H | 4.517091 | -1.216050 | 2.763054 |
| H | 3.662283 | -2.389377 | 1.690137 |
| O | 2.487860 | -1.458155 | 3.127067 |
| C | 0.155534 | -1.011810 | 3.104300 |

| | | | |
|---|---|---|---|
| H | 0.122903 | -1.497053 | 4.081509 |
| C | -1.027147 | -0.632004 | 2.464559 |
| O | -1.082824 | -0.153804 | 1.265577 |
| C | -2.359205 | -0.809093 | 3.158481 |
| H | -2.960912 | -1.549854 | 2.603246 |
| H | -2.247079 | -1.134853 | 4.204448 |
| H | -2.918946 | 0.141684 | 3.122551 |
| C | 3.905082 | 1.070339 | 1.548338 |
| H | 4.991101 | 0.851398 | 1.486915 |
| C | 3.576944 | 2.154104 | 0.516254 |
| H | 2.513794 | 2.432989 | 0.584333 |
| H | 4.178494 | 3.061610 | 0.700803 |
| H | 3.770672 | 1.814797 | -0.515029 |
| C | 3.586371 | 1.561511 | 2.969724 |
| H | 4.149489 | 2.484477 | 3.192905 |
| H | 2.509778 | 1.790595 | 3.060492 |
| H | 3.839073 | 0.814464 | 3.741654 |
| N | 1.472908 | 1.910009 | -2.194005 |
| C | 2.783148 | -0.025429 | -3.792457 |
| H | 3.846156 | -0.312962 | -3.689617 |
| H | 2.370119 | -0.606498 | -4.636564 |
| H | 2.721016 | 1.049108 | -4.017780 |
| Co | 0.362573 | 0.043756 | -0.061455 |
| C | -2.624577 | -1.401418 | -1.163449 |
| C | -3.432454 | -0.361406 | -0.628120 |
| C | -2.908597 | -2.709236 | -0.789730 |
| C | -2.977706 | 0.903377 | -1.095660 |
| C | -4.504061 | -0.552652 | 0.258635 |
| C | -4.003863 | -2.942649 | 0.098423 |
| H | -2.312314 | -3.553058 | -1.152240 |
| C | -3.634051 | 2.041445 | -0.639342 |
| C | -5.165488 | 0.635103 | 0.707298 |

| | | | |
|---|---|---|---|
| C | -4.787504 | -1.910490 | 0.612780 |
| H | -4.231999 | -3.975019 | 0.385526 |
| C | -4.729403 | 1.882999 | 0.265017 |
| H | -3.328502 | 3.046303 | -0.949669 |
| H | -6.012763 | 0.563804 | 1.398507 |
| H | -5.615952 | -2.138477 | 1.292896 |
| H | -5.246191 | 2.781451 | 0.620296 |
| C | -1.839916 | 0.625704 | -1.983687 |
| H | -1.286837 | 1.370236 | -2.558859 |
| C | -1.621027 | -0.740238 | -2.017678 |
| H | -0.892600 | -1.255938 | -2.642762 |

**TS(1-2)B**

Lowest frequency = -297.2222 $cm^{-1}$

Charge = 0, Multiplicity = 1

74

| | | | |
|---|---|---|---|
| C | 0.127888 | -1.747411 | -0.816668 |
| C | 1.210811 | -1.935089 | -1.748617 |
| C | -0.297039 | -2.844658 | -0.048823 |
| C | 1.766431 | -3.219606 | -1.929207 |
| C | 0.296288 | -4.106027 | -0.205513 |
| H | -1.108182 | -2.716933 | 0.672018 |
| C | 1.309519 | -4.299366 | -1.161679 |
| H | 2.587717 | -3.360874 | -2.640349 |
| H | -0.043208 | -4.944630 | 0.411876 |
| H | 1.752104 | -5.291666 | -1.299286 |
| C | 1.736050 | -0.733188 | -2.363272 |
| N | 1.352552 | 0.371107 | -1.724774 |
| C | 1.307843 | 2.520060 | -1.209426 |
| C | 1.572538 | 3.907338 | -1.364414 |

| | | | |
|---|---|---|---|
| N | 0.554730 | 2.095262 | -0.130794 |
| C | 1.088339 | 4.802809 | -0.418971 |
| H | 2.156907 | 4.229479 | -2.229399 |
| C | 0.070870 | 2.969649 | 0.767932 |
| C | 0.325602 | 4.337559 | 0.675955 |
| H | 1.296767 | 5.872677 | -0.527038 |
| H | -0.536421 | 2.525852 | 1.563051 |
| H | -0.061655 | 5.020093 | 1.436670 |
| N | 1.644076 | -0.404319 | 0.998313 |
| C | 1.316723 | -1.169828 | 2.026270 |
| C | 3.089523 | -0.613868 | 0.738798 |
| H | 3.265450 | -0.686252 | -0.346080 |
| C | 3.325847 | -1.967798 | 1.424606 |
| H | 4.307071 | -2.063543 | 1.914149 |
| H | 3.152559 | -2.821885 | 0.744988 |
| O | 2.303838 | -2.004129 | 2.455143 |
| C | 0.084482 | -1.213810 | 2.724218 |
| H | 0.025142 | -1.851326 | 3.607290 |
| C | -1.012292 | -0.474179 | 2.297342 |
| O | -1.055213 | 0.207318 | 1.198109 |
| C | -2.299651 | -0.451186 | 3.089882 |
| H | -3.093493 | -0.953507 | 2.507969 |
| H | -2.193476 | -0.943999 | 4.068622 |
| H | -2.624676 | 0.593795 | 3.232714 |
| C | 4.004152 | 0.500372 | 1.324880 |
| H | 5.033169 | 0.118849 | 1.158881 |
| C | 3.880456 | 1.837738 | 0.587828 |
| H | 2.920699 | 2.322817 | 0.825947 |
| H | 4.687743 | 2.522945 | 0.901728 |
| H | 3.934072 | 1.712599 | -0.506454 |
| C | 3.802370 | 0.701230 | 2.835814 |
| H | 4.505883 | 1.462417 | 3.216377 |

| | | | |
|---|---|---|---|
| H | 2.776849 | 1.057408 | 3.038491 |
| H | 3.957917 | -0.226033 | 3.412316 |
| N | 1.789877 | 1.590656 | -2.068624 |
| C | 2.701990 | -0.678150 | -3.507600 |
| H | 3.749142 | -0.714567 | -3.147225 |
| H | 2.549356 | -1.524029 | -4.197460 |
| H | 2.580312 | 0.274341 | -4.046840 |
| Co | 0.199181 | 0.200077 | -0.256779 |
| C | -2.656880 | -1.123312 | -0.937083 |
| C | -3.279720 | 0.076715 | -0.511386 |
| C | -3.316952 | -2.326856 | -0.719881 |
| C | -2.506741 | 1.217462 | -0.861693 |
| C | -4.528485 | 0.135227 | 0.150790 |
| C | -4.580732 | -2.301267 | -0.061290 |
| H | -2.888157 | -3.282956 | -1.035485 |
| C | -2.996543 | 2.475539 | -0.535429 |
| C | -5.001190 | 1.442671 | 0.486793 |
| C | -5.175025 | -1.116828 | 0.374337 |
| H | -5.102064 | -3.250046 | 0.106384 |
| C | -4.249072 | 2.563691 | 0.144312 |
| H | -2.444711 | 3.387601 | -0.783345 |
| H | -5.963605 | 1.560459 | 0.996746 |
| H | -6.148084 | -1.146599 | 0.877439 |
| H | -4.634517 | 3.557087 | 0.400027 |
| C | -1.290824 | 0.725699 | -1.528963 |
| H | -0.832398 | 1.298203 | -2.341678 |
| C | -1.361163 | -0.744061 | -1.595633 |
| H | -1.112224 | -1.217918 | -2.550101 |

**TS(1-2)B**

Lowest frequency = -315.3900 $cm^{-1}$

Charge = 0, Multiplicity = 3

74

| | | | |
|---|---|---|---|
| C | 0.110117 | -1.697062 | -0.861056 |
| C | 1.180117 | -1.952373 | -1.792561 |
| C | -0.431740 | -2.767960 | -0.129711 |
| C | 1.607311 | -3.281748 | -2.008327 |
| C | 0.051400 | -4.071825 | -0.312446 |
| H | -1.241083 | -2.587069 | 0.583204 |
| C | 1.052225 | -4.333169 | -1.267937 |
| H | 2.408889 | -3.480699 | -2.728421 |
| H | -0.367496 | -4.890704 | 0.282723 |
| H | 1.405682 | -5.357705 | -1.425301 |
| C | 1.799802 | -0.789872 | -2.399328 |
| N | 1.503789 | 0.328389 | -1.745459 |
| C | 1.510437 | 2.488192 | -1.271795 |
| C | 1.886917 | 3.853127 | -1.432318 |
| N | 0.673364 | 2.142290 | -0.229060 |
| C | 1.428129 | 4.799041 | -0.529097 |
| H | 2.538786 | 4.110829 | -2.270701 |
| C | 0.222070 | 3.070418 | 0.636661 |
| C | 0.581287 | 4.411519 | 0.538638 |
| H | 1.723400 | 5.847931 | -0.641156 |
| H | -0.440431 | 2.688276 | 1.420920 |
| H | 0.217369 | 5.134841 | 1.272995 |
| N | 1.577855 | -0.471136 | 1.119328 |
| C | 1.181539 | -1.251326 | 2.127276 |
| C | 3.006125 | -0.778005 | 0.868782 |
| H | 3.180290 | -0.829387 | -0.216818 |
| C | 3.145915 | -2.162615 | 1.517483 |
| H | 4.109875 | -2.334812 | 2.020176 |
| H | 2.932051 | -2.982753 | 0.808477 |

| | | | |
|---|---|---|---|
| O | 2.105215 | -2.165645 | 2.529689 |
| C | -0.058338 | -1.241586 | 2.799730 |
| H | -0.171532 | -1.914524 | 3.651488 |
| C | -1.124682 | -0.442137 | 2.364393 |
| O | -1.083098 | 0.271843 | 1.299455 |
| C | -2.438320 | -0.430187 | 3.115769 |
| H | -3.206831 | -0.940188 | 2.506458 |
| H | -2.366221 | -0.923578 | 4.097768 |
| H | -2.776826 | 0.612041 | 3.243602 |
| C | 3.972903 | 0.273211 | 1.483306 |
| H | 4.984309 | -0.149785 | 1.311195 |
| C | 3.899794 | 1.624839 | 0.764687 |
| H | 2.941635 | 2.124413 | 0.979279 |
| H | 4.711776 | 2.288614 | 1.110475 |
| H | 3.984280 | 1.511905 | -0.329090 |
| C | 3.774754 | 0.451148 | 2.997163 |
| H | 4.518417 | 1.161847 | 3.398216 |
| H | 2.770299 | 0.860125 | 3.205841 |
| H | 3.875853 | -0.496996 | 3.551937 |
| N | 1.957435 | 1.531924 | -2.122667 |
| C | 2.736237 | -0.819727 | -3.570801 |
| H | 2.868123 | 0.203005 | -3.955179 |
| H | 3.731307 | -1.203776 | -3.271780 |
| H | 2.356114 | -1.477356 | -4.371480 |
| Co | 0.247351 | 0.195626 | -0.192597 |
| C | -2.743306 | -0.978959 | -1.105309 |
| C | -3.279218 | 0.217127 | -0.557572 |
| C | -3.460056 | -2.162434 | -0.942147 |
| C | -2.443636 | 1.331844 | -0.840946 |
| C | -4.498267 | 0.293878 | 0.151220 |
| C | -4.690602 | -2.117338 | -0.225677 |
| H | -3.095744 | -3.113628 | -1.342499 |

| | | | |
|---|---|---|---|
| C | -2.853650 | 2.590966 | -0.415523 |
| C | -4.884457 | 1.597444 | 0.593046 |
| C | -5.204755 | -0.937247 | 0.313971 |
| H | -5.255404 | -3.047488 | -0.098804 |
| C | -4.080610 | 2.699085 | 0.303208 |
| H | -2.259725 | 3.487451 | -0.617151 |
| H | -5.822133 | 1.731975 | 1.143545 |
| H | -6.159889 | -0.952254 | 0.850734 |
| H | -4.403925 | 3.691520 | 0.636535 |
| C | -1.270298 | 0.810377 | -1.558156 |
| H | -0.715453 | 1.414706 | -2.280844 |
| C | -1.465573 | -0.619116 | -1.760214 |
| H | -1.135710 | -1.103700 | -2.680827 |

**TS(1-2)B**

Lowest frequency = -280.6342 $cm^{-1}$

Charge = 0, Multiplicity = 5

74

| | | | |
|---|---|---|---|
| C | -0.317399 | -1.554511 | 1.135334 |
| C | -1.395360 | -1.408492 | 2.090067 |
| C | -0.101640 | -2.830954 | 0.568830 |
| C | -2.138691 | -2.544385 | 2.503138 |
| C | -0.905185 | -3.918841 | 0.916828 |
| H | 0.699556 | -2.968623 | -0.164663 |
| C | -1.903877 | -3.784247 | 1.910528 |
| H | -2.928430 | -2.437481 | 3.255565 |
| H | -0.750562 | -4.888777 | 0.430168 |
| H | -2.500068 | -4.654128 | 2.206310 |
| C | -1.694980 | -0.056771 | 2.489447 |
| N | -1.176305 | 0.849844 | 1.651182 |

| | | | |
|---|---|---|---|
| C | -0.601340 | 2.868609 | 0.962919 |
| C | -0.550424 | 4.287260 | 1.024214 |
| N | -0.001147 | 2.203598 | -0.080731 |
| C | 0.098571 | 4.986827 | 0.014968 |
| H | -1.032321 | 4.786729 | 1.868511 |
| C | 0.631313 | 2.892655 | -1.050407 |
| C | 0.704308 | 4.284435 | -1.052077 |
| H | 0.140098 | 6.080920 | 0.047266 |
| H | 1.092370 | 2.280362 | -1.833123 |
| H | 1.225415 | 4.807609 | -1.858237 |
| N | -1.899020 | -0.387435 | -1.040552 |
| C | -1.774468 | -1.362016 | -1.933589 |
| C | -3.325985 | -0.281674 | -0.679166 |
| H | -3.411978 | -0.202859 | 0.417871 |
| C | -3.881006 | -1.629162 | -1.177461 |
| H | -4.870222 | -1.562815 | -1.656365 |
| H | -3.897755 | -2.394682 | -0.381086 |
| O | -2.922695 | -2.051605 | -2.181122 |
| C | -0.612208 | -1.760003 | -2.641519 |
| H | -0.736494 | -2.551407 | -3.382697 |
| C | 0.650161 | -1.206715 | -2.406352 |
| O | 0.886365 | -0.321923 | -1.494375 |
| C | 1.849669 | -1.643130 | -3.215298 |
| H | 2.608132 | -2.070092 | -2.535651 |
| H | 1.587279 | -2.379210 | -3.991054 |
| H | 2.309936 | -0.758591 | -3.690018 |
| C | -4.008734 | 0.954116 | -1.322955 |
| H | -5.079203 | 0.872625 | -1.042671 |
| C | -3.457485 | 2.262643 | -0.743888 |
| H | -2.404402 | 2.396135 | -1.040494 |
| H | -4.030042 | 3.128047 | -1.121300 |
| H | -3.497962 | 2.273557 | 0.358515 |

| | | | |
|---|---|---|---|
| C | -3.902968 | 0.948136 | -2.856175 |
| H | -4.448283 | 1.808123 | -3.282500 |
| H | -2.846043 | 1.029723 | -3.165962 |
| H | -4.316762 | 0.028254 | -3.304045 |
| N | -1.222844 | 2.148826 | 1.942428 |
| C | -2.469663 | 0.371097 | 3.697950 |
| H | -3.328494 | 1.001662 | 3.405050 |
| H | -2.826304 | -0.488617 | 4.284277 |
| H | -1.831700 | 1.007128 | 4.338336 |
| Co | -0.266657 | 0.173981 | -0.019721 |
| C | 2.654022 | -1.406608 | 1.107266 |
| C | 3.288003 | -0.284246 | 0.517148 |
| C | 3.108972 | -2.677168 | 0.759388 |
| C | 2.748858 | 0.932232 | 1.048472 |
| C | 4.323253 | -0.354782 | -0.434250 |
| C | 4.164162 | -2.785112 | -0.192810 |
| H | 2.680000 | -3.583047 | 1.199332 |
| C | 3.281660 | 2.141460 | 0.576136 |
| C | 4.831732 | 0.898898 | -0.904413 |
| C | 4.755665 | -1.669570 | -0.788435 |
| H | 4.522962 | -3.784043 | -0.464549 |
| C | 4.311701 | 2.095900 | -0.400805 |
| H | 2.916577 | 3.107110 | 0.939863 |
| H | 5.636518 | 0.920023 | -1.647390 |
| H | 5.562410 | -1.802406 | -1.518059 |
| H | 4.720096 | 3.043382 | -0.770282 |
| C | 1.739454 | 0.558200 | 2.005155 |
| H | 1.186850 | 1.243375 | 2.649059 |
| C | 1.600957 | -0.870150 | 2.006802 |
| H | 1.228118 | -1.424647 | 2.870229 |

**Int2B**

Lowest frequency = 21.8556 cm$^{-1}$

Charge = 0, Multiplicity = 1

74

| | | | |
|---|---|---|---|
| C | -0.320998 | -1.736735 | -1.159206 |
| C | 0.924915 | -1.798000 | -1.894809 |
| C | -0.599373 | -2.799686 | -0.268006 |
| C | 1.684242 | -2.987173 | -1.874240 |
| C | 0.226077 | -3.924323 | -0.176967 |
| H | -1.508932 | -2.755045 | 0.334768 |
| C | 1.336494 | -4.046177 | -1.028971 |
| H | 2.602241 | -3.041182 | -2.469611 |
| H | -0.016188 | -4.720519 | 0.533563 |
| H | 1.953429 | -4.951014 | -1.004818 |
| C | 1.480528 | -0.586415 | -2.493492 |
| N | 1.295211 | 0.486053 | -1.734177 |
| C | 1.357894 | 2.603803 | -1.158561 |
| C | 1.650812 | 3.989781 | -1.262432 |
| N | 0.644606 | 2.150595 | -0.056357 |
| C | 1.237343 | 4.855318 | -0.259020 |
| H | 2.198175 | 4.332797 | -2.143414 |
| C | 0.225857 | 3.000579 | 0.906251 |
| C | 0.515198 | 4.360442 | 0.852083 |
| H | 1.468020 | 5.923330 | -0.331703 |
| H | -0.350758 | 2.537798 | 1.711627 |
| H | 0.184996 | 5.018301 | 1.659933 |
| N | 1.732146 | -0.638962 | 0.883583 |
| C | 1.395845 | -1.458928 | 1.865759 |
| C | 3.164458 | -0.860871 | 0.589267 |
| H | 3.312975 | -0.897890 | -0.503620 |
| C | 3.410631 | -2.240806 | 1.228503 |

| | | | |
|---|---|---|---|
| H | 4.376633 | -2.324313 | 1.750410 |
| H | 3.299089 | -3.069225 | 0.508152 |
| O | 2.355733 | -2.361173 | 2.213932 |
| C | 0.176870 | -1.500267 | 2.586973 |
| H | 0.089966 | -2.235480 | 3.388065 |
| C | -0.854402 | -0.601642 | 2.326443 |
| O | -0.856961 | 0.252220 | 1.354620 |
| C | -2.110766 | -0.585239 | 3.167294 |
| H | -2.973872 | -0.856561 | 2.532965 |
| H | -2.048650 | -1.276140 | 4.022207 |
| H | -2.295590 | 0.439718 | 3.533149 |
| C | 4.099776 | 0.227094 | 1.189145 |
| H | 5.123734 | -0.160654 | 1.007667 |
| C | 3.979485 | 1.580823 | 0.482269 |
| H | 3.026819 | 2.067669 | 0.742214 |
| H | 4.795665 | 2.253508 | 0.799914 |
| H | 4.018206 | 1.480236 | -0.615329 |
| C | 3.903991 | 0.391471 | 2.705038 |
| H | 4.625858 | 1.123090 | 3.108004 |
| H | 2.887013 | 0.765553 | 2.917775 |
| H | 4.036974 | -0.555845 | 3.254968 |
| N | 1.750381 | 1.699541 | -2.083373 |
| C | 2.314212 | -0.519569 | -3.738487 |
| H | 3.381054 | -0.347376 | -3.498002 |
| H | 2.222993 | -1.445115 | -4.327557 |
| H | 1.994384 | 0.340649 | -4.351729 |
| Co | 0.323644 | 0.326021 | -0.144243 |
| C | -2.837654 | -1.075010 | -0.981826 |
| C | -3.334373 | 0.125671 | -0.423535 |
| C | -3.622254 | -2.219515 | -0.902747 |
| C | -2.449837 | 1.218724 | -0.636621 |
| C | -4.592095 | 0.237119 | 0.227178 |

| | | | |
|---|---|---|---|
| C | -4.882682 | -2.143250 | -0.240883 |
| H | -3.295750 | -3.171694 | -1.334100 |
| C | -2.846135 | 2.481567 | -0.208405 |
| C | -4.950359 | 1.540464 | 0.688688 |
| C | -5.362184 | -0.960249 | 0.316809 |
| H | -5.496332 | -3.048873 | -0.177618 |
| C | -4.097279 | 2.617701 | 0.459933 |
| H | -2.218063 | 3.362656 | -0.370704 |
| H | -5.906346 | 1.693012 | 1.201682 |
| H | -6.340311 | -0.943211 | 0.810588 |
| H | -4.400327 | 3.613803 | 0.802590 |
| C | -1.242129 | 0.711414 | -1.316260 |
| H | -0.889160 | 1.319800 | -2.160825 |
| C | -1.481621 | -0.787842 | -1.622991 |
| H | -1.545835 | -0.944982 | -2.717846 |

**Int2B**

Lowest frequency = 15.7873 cm$^{-1}$

Charge = 0, Multiplicity = 3

74

| | | | |
|---|---|---|---|
| C | -0.737156 | -1.852877 | -1.229284 |
| C | 0.566593 | -2.007333 | -1.797108 |
| C | -1.241186 | -2.903782 | -0.444647 |
| C | 1.195515 | -3.270267 | -1.734962 |
| C | -0.559733 | -4.122998 | -0.310930 |
| H | -2.206441 | -2.776984 | 0.051086 |
| C | 0.636342 | -4.328416 | -1.008645 |
| H | 2.169232 | -3.399883 | -2.220240 |
| H | -0.984940 | -4.917610 | 0.310420 |
| H | 1.149575 | -5.295033 | -0.963058 |

| | | | |
|---|---|---|---|
| C | 1.324987 | -0.880015 | -2.366518 |
| N | 1.356802 | 0.241169 | -1.664867 |
| C | 1.995766 | 2.361152 | -1.329396 |
| C | 2.625840 | 3.592491 | -1.679580 |
| N | 1.335045 | 2.260616 | -0.123945 |
| C | 2.552691 | 4.664429 | -0.805462 |
| H | 3.149555 | 3.651803 | -2.636903 |
| C | 1.266926 | 3.315067 | 0.717656 |
| C | 1.857589 | 4.538393 | 0.424611 |
| H | 3.033351 | 5.613593 | -1.066603 |
| H | 0.712860 | 3.133431 | 1.645783 |
| H | 1.787198 | 5.370924 | 1.129459 |
| N | 1.486726 | -0.905587 | 0.965354 |
| C | 0.875395 | -1.624741 | 1.901054 |
| C | 2.821063 | -1.495220 | 0.731580 |
| H | 2.980283 | -1.606336 | -0.354293 |
| C | 2.695491 | -2.873768 | 1.414823 |
| H | 3.559583 | -3.129356 | 2.048264 |
| H | 2.497899 | -3.690209 | 0.702191 |
| O | 1.536391 | -2.747372 | 2.276027 |
| C | -0.347193 | -1.338933 | 2.558678 |
| H | -0.720387 | -2.082561 | 3.264121 |
| C | -0.980923 | -0.103531 | 2.423662 |
| O | -0.577350 | 0.821795 | 1.611306 |
| C | -2.195079 | 0.254260 | 3.245453 |
| H | -3.048441 | 0.432367 | 2.567197 |
| H | -2.455534 | -0.533409 | 3.968839 |
| H | -2.007342 | 1.200889 | 3.781435 |
| C | 3.978344 | -0.645826 | 1.323382 |
| H | 4.872843 | -1.297722 | 1.243084 |
| C | 4.245804 | 0.626236 | 0.512156 |
| H | 3.422038 | 1.344142 | 0.641650 |

| | | | |
|---|---|---|---|
| H | 5.176700 | 1.113476 | 0.851653 |
| H | 4.338442 | 0.413475 | -0.566635 |
| C | 3.745119 | -0.315324 | 2.805838 |
| H | 4.615523 | 0.218138 | 3.225373 |
| H | 2.860044 | 0.335997 | 2.913645 |
| H | 3.575656 | -1.219717 | 3.416356 |
| N | 2.042328 | 1.294600 | -2.156527 |
| C | 2.148428 | -0.997526 | -3.619363 |
| H | 3.227898 | -0.900222 | -3.397174 |
| H | 1.961217 | -1.953880 | -4.130673 |
| H | 1.905866 | -0.159856 | -4.297263 |
| Co | 0.440971 | 0.482617 | 0.045657 |
| C | -3.051930 | -0.694905 | -1.063397 |
| C | -3.326538 | 0.540604 | -0.432504 |
| C | -4.062474 | -1.647142 | -1.125920 |
| C | -2.232360 | 1.444653 | -0.526796 |
| C | -4.577375 | 0.869690 | 0.159240 |
| C | -5.320661 | -1.355001 | -0.521194 |
| H | -3.916597 | -2.607571 | -1.630935 |
| C | -2.390000 | 2.731171 | -0.016118 |
| C | -4.692646 | 2.185691 | 0.699537 |
| C | -5.581756 | -0.142562 | 0.110903 |
| H | -6.111360 | -2.112197 | -0.567504 |
| C | -3.624276 | 3.077104 | 0.598491 |
| H | -1.579401 | 3.464271 | -0.066778 |
| H | -5.628258 | 2.498830 | 1.175784 |
| H | -6.566186 | 0.045947 | 0.553600 |
| H | -3.739668 | 4.087647 | 1.006703 |
| C | -1.113856 | 0.764359 | -1.213085 |
| H | -0.677401 | 1.337131 | -2.043127 |
| C | -1.629513 | -0.656321 | -1.619014 |
| H | -1.682083 | -0.694024 | -2.725817 |

**Int2B**

Lowest frequency = 13.1026 $cm^{-1}$

Charge = 0, Multiplicity = 5

74

| | | | |
|---|---|---|---|
| C | -0.931510 | -1.582935 | -1.247680 |
| C | 0.307428 | -1.908660 | -1.878406 |
| C | -1.499705 | -2.530962 | -0.377188 |
| C | 0.801987 | -3.228610 | -1.783192 |
| C | -0.944891 | -3.807406 | -0.215331 |
| H | -2.420315 | -2.278048 | 0.154667 |
| C | 0.179226 | -4.178315 | -0.966395 |
| H | 1.722094 | -3.488670 | -2.318379 |
| H | -1.415309 | -4.521345 | 0.468107 |
| H | 0.583595 | -5.193999 | -0.897951 |
| C | 1.145047 | -0.884043 | -2.527191 |
| N | 1.353765 | 0.212393 | -1.817119 |
| C | 2.279349 | 2.224788 | -1.497390 |
| C | 3.019532 | 3.377131 | -1.901269 |
| N | 1.746110 | 2.181748 | -0.222336 |
| C | 3.179616 | 4.432633 | -1.020797 |
| H | 3.437117 | 3.386768 | -2.911233 |
| C | 1.903263 | 3.226730 | 0.624367 |
| C | 2.608157 | 4.370608 | 0.276590 |
| H | 3.746206 | 5.318560 | -1.327740 |
| H | 1.430796 | 3.102854 | 1.605770 |
| H | 2.715737 | 5.192491 | 0.989169 |
| N | 1.457604 | -1.071856 | 1.039035 |
| C | 0.775621 | -1.668865 | 2.008305 |
| C | 2.663668 | -1.871063 | 0.753143 |

| | | | |
|---|---|---|---|
| H | 2.728174 | -2.044080 | -0.335015 |
| C | 2.372276 | -3.192463 | 1.500865 |
| H | 3.208727 | -3.530314 | 2.133955 |
| H | 2.066184 | -4.006221 | 0.823824 |
| O | 1.253990 | -2.888177 | 2.368643 |
| C | -0.344958 | -1.169879 | 2.717516 |
| H | -0.814450 | -1.844622 | 3.435346 |
| C | -0.762412 | 0.163504 | 2.622624 |
| O | -0.220283 | 1.029612 | 1.831770 |
| C | -1.904993 | 0.665962 | 3.475972 |
| H | -2.770757 | 0.887595 | 2.826078 |
| H | -2.206213 | -0.062650 | 4.244719 |
| H | -1.613512 | 1.615578 | 3.956410 |
| C | 3.970378 | -1.178315 | 1.221820 |
| H | 4.756367 | -1.957368 | 1.140688 |
| C | 4.353819 | -0.015098 | 0.299884 |
| H | 3.609391 | 0.792990 | 0.373587 |
| H | 5.336808 | 0.401659 | 0.581042 |
| H | 4.400940 | -0.330884 | -0.756847 |
| C | 3.878326 | -0.722525 | 2.686013 |
| H | 4.843229 | -0.308552 | 3.026138 |
| H | 3.110032 | 0.063435 | 2.790519 |
| H | 3.607235 | -1.550995 | 3.364432 |
| N | 2.108951 | 1.192950 | -2.357772 |
| C | 1.819611 | -1.081984 | -3.854390 |
| H | 2.916676 | -0.995114 | -3.749581 |
| H | 1.556202 | -2.051850 | -4.302845 |
| H | 1.522530 | -0.267596 | -4.540415 |
| Co | 0.689698 | 0.523778 | 0.147742 |
| C | -3.192571 | -0.392714 | -1.251455 |
| C | -3.505670 | 0.690396 | -0.405615 |
| C | -4.192457 | -1.308941 | -1.564201 |

| | | | |
|---|---|---|---|
| C | -2.379826 | 1.559126 | -0.222765 |
| C | -4.791217 | 0.923256 | 0.147468 |
| C | -5.490581 | -1.111597 | -1.014885 |
| H | -3.998911 | -2.170407 | -2.212018 |
| C | -2.566225 | 2.726362 | 0.540874 |
| C | -4.930177 | 2.105042 | 0.941305 |
| C | -5.794402 | -0.035224 | -0.180313 |
| H | -6.280057 | -1.829753 | -1.263152 |
| C | -3.840302 | 2.969460 | 1.108381 |
| H | -1.742298 | 3.424483 | 0.713066 |
| H | -5.894561 | 2.342112 | 1.403467 |
| H | -6.809466 | 0.081369 | 0.215520 |
| H | -3.978595 | 3.874827 | 1.710467 |
| C | -1.266109 | 1.002764 | -0.934226 |
| H | -0.553210 | 1.664330 | -1.443642 |
| C | -1.713032 | -0.303054 | -1.625935 |
| H | -1.594499 | -0.187386 | -2.722161 |

## References


(1) Asiri, A.; Bahajaj, A.; Ismail, I.; Fatani, N. Novel Dyes Derived from Hydrazones: Part 3. Synthesis and Characterizations of 2-[4-(1-Phenylethylidene)Hydrazino]Phenylethylene-1,1,2-Tricarbonitrile. *Dyes Pigm.* **2006**, *71*, 103–108.

(2) Dugal-Tessier, J.; Dake, G. R.; Gates, D. P. Chiral Phosphaalkene−Oxazoline Ligands for the Palladium-Catalyzed Asymmetric Allylic Alkylation. *Org. Lett.* **2010**, *12*, 4667–4669.

(3) May, K. L.; Resanović, S.; Chojnacka, M. W.; Herasymchuk, K.; Vaughan, D. G.; Zhu, J.; Wilson Quail, J.; Lough, A. J.; Gossage, R. A. Divalent Cobalt and Copper Coordination Complexes of K2-N, O-Derivatives of (Z)-1-R-2-(2′-Oxazolin-2′-Yl)-Eth-1-En-1-Ates: Structure and Reactivity Patterns. *Inorganica Chim.* **2021**, *514*, 119959.

(4) Jones, R. C.; Herasymchuk, K.; Mahdi, T.; Petrov, A.; Resanović, S.; Vaughan, D. G.; Lough, A. J.; Quail, J. W.; Koivisto, B. D.; Wylie, R. S.; Gossage, R. A. Tautomerism and Metal Complexation of 2-Acylmethyl-2-Oxazolines: A Combined Synthetic, Spectroscopic, Crystallographic and Theoretical Treatment. *Org. Biomol. Chem.* **2013**, *11*, 3484.

(5) Rasappan, R.; Hager, M.; Gissibl, A.; Reiser, O. Highly Enantioselective Michael Additions of Indole to Benzylidene Malonate Using Simple Bis(Oxazoline) Ligands: Importance of Metal/Ligand Ratio. *Org. Lett.* **2006**, *8*, 6099–6102.

(6) Bruker AXS Inc., SAINT *v8.40B*, Madison, **2016**.

(7) Krause, L.; Herbst-Irmer, R.; Sheldrick, G. M.; Stalke, D. Comparison of Silver and Molybdenum Microfocus X-Ray Sources for Single-Crystal Structure Determination. *J. Appl. Crystallogr.* **2015**, *48*, 3–10.

(8) Sheldrick, G. M. *SHELXT* – Integrated Space-Group and Crystal-Structure Determination. *Acta Crystallogr.* **2015**, *71*, 3–8.

(9) Sheldrick, G. M. Crystal Structure Refinement with *SHELXL*. *Acta Crystallogr.* **2015**, *71*, 3–8.

(10) Hübschle, C. B.; Sheldrick, G. M.; Dittrich, B. *ShelXle* : A Qt Graphical User Interface for *SHELXL*. *J. Appl. Crystallogr.* **2011**, *44*, 1281–1284.

(11) Parsons, S.; Flack, H. D.; Wagner, T. Use of Intensity Quotients and Differences in Absolute Structure Refinement. *Acta Crystallogr.* **2013**, *69*, 249–259.

(12) Riniker, S.; Landrum, G. A. Better Informed Distance Geometry: Using What We Know To Improve Conformation Generation. *J. Chem. Inf. Model.* **2015**, *55*, 2562–2574.

(13) RDKit: Open-source cheminformatics. https://www.rdkit.org.

(14) Pracht, P.; Bohle, F.; Grimme, S. Automated Exploration of the Low-Energy Chemical Space with Fast Quantum Chemical Methods. *Phys. Chem. Chem. Phys.* **2020**, *22*, 7169–7192.

(15) Bannwarth, C.; Ehlert, S.; Grimme, S. GFN2-xTB—An Accurate and Broadly Parametrized Self-Consistent Tight-Binding Quantum Chemical Method with Multipole Electrostatics and Density-Dependent Dispersion Contributions. *J. Chem. Theory Comput.* **2019**, *15*, 1652–1671.

(16) Gaussian 16, Revision A.03, M. J. Frisch, G. W. Trucks, H. B. Schlegel, G. E. Scuseria, M. A. Robb, J. R. Cheeseman, G. Scalmani, V. Barone, G. A. Petersson, H. Nakatsuji, X. Li, M. Caricato, A. V. Marenich, J. Bloino, B. G. Janesko, R. Gomperts, B. Mennucci, H. P. Hratchian, J. V. Ortiz, A. F. Izmaylov, J. L. Sonnenberg, D. Williams-Young, F. Ding, F. Lipparini, F. Egidi, J. Goings, B. Peng, A. Petrone, T. Henderson, D. Ranasinghe, V. G. akrzewski, J. Gao, N. Rega, G. Zheng, W. Liang, M. Hada, M. Ehara, K. Toyota, R. Fukuda, J. Hasegawa, M. Ishida, T. Nakajima, Y. Honda, O. Kitao, H. Nakai, T. Vreven, K. Throssell, J. A. Montgomery, Jr., J. E. Peralta, F. Ogliaro, M. J. Bearpark, J. J. Heyd, E. N. Brothers, K. N. Kudin, V. N. Staroverov, T. A. Keith, R. Kobayashi, J. Normand, K. Raghavachari, A. P. Rendell, J. C. Burant, S. S. Iyengar, J. Tomasi, M. Cossi, J. M. Millam, M. Klene, C. Adamo, R. Cammi, J. W. Ochterski, R. L. Martin, K. Morokuma, O. Farkas, J. B. Foresman, D. J. Fox, Gaussian, Inc., Wallingford CT, 2016.

(17) GitHub repository: https://github.com/neeraj-compchem/Ligand-parameterization

(18) Luchini, G.; Patterson, T.; Paton, R. S. DBSTEP: DFT Based Steric Parameters. 2022, DOI:10.5281/zenodo.4702097.

(19) Pedregosa, F.; Varoquaux, G.; Gramfort, A.; Michel, V.; Thirion, B.; Grisel, O.; Blondel, M.; Prettenhofer, P.; Weiss, R.; Dubourg, V.; Vanderplas, J.; Passos, A.; Cournapeau, D.; Brucher, M.; Perrot, M.; Duchesnay, É. Scikit-learn: Machine Learning in Python. *J. Mach. Learn. Res.* **2011**, *12*, 2825–2830.

(20) Newman-Stonebraker, S. H.; Smith, S. R.; Borowski, J. E.; Peters, E.; Gensch, T.; Johnson, H. C.; Sigman, M. S.; Doyle, A. G. Univariate Classification of Phosphine Ligation State and Reactivity in Cross-Coupling Catalysis. *Science* **2021**, *374*, 301–308.

(21) Tao, J.; Perdew, J. P.; Staroverov, V. N.; Scuseria, G. E. Climbing the Density Functional Ladder: Nonempirical Meta–Generalized Gradient Approximation Designed for Molecules and Solids. *Phys. Rev. Lett.* **2003**, *91*, 146401.

(22) Grimme, S.; Ehrlich, S.; Goerigk, L. Effect of the Damping Function in Dispersion Corrected Density Functional Theory. *J Comput. Chem.* **2011**, *32*, 1456–1465.

(23) Grimme, S.; Antony, J.; Ehrlich, S.; Krieg, H. A Consistent and Accurate *Ab Initio* Parametrization of Density Functional Dispersion Correction (DFT-D) for the 94 Elements H-Pu. *J. Chem. Phys.* **2010**, *132*, 154104.

(24) Weigend, F. Accurate Coulomb-Fitting Basis Sets for H to Rn. *Phys. Chem. Chem. Phys.* **2006**, *8*, 1057.

(25) Weigend, F.; Ahlrichs, R. Balanced Basis Sets of Split Valence, Triple Zeta Valence and Quadruple Zeta Valence Quality for H to Rn: Design and Assessment of Accuracy. *Phys. Chem. Chem. Phys.* **2005**, *7*, 3297–3305.

(26) Schäfer, A.; Huber, C.; Ahlrichs, R. Fully Optimized Contracted Gaussian Basis Sets of Triple Zeta Valence Quality for Atoms Li to Kr. *J. Chem. Phys.* **1994**, *100*, 5829–5835.

(27) Schäfer, A.; Horn, H.; Ahlrichs, R. Fully Optimized Contracted Gaussian Basis Sets for Atoms Li to Kr. *J. Chem. Phys.* **1992**, *97*, 2571–2577.

(28) Fukui, K. The Path of Chemical Reactions - the IRC Approach. *Acc. Chem. Res.* **1981**, *14*, 363–368.

(29) Zhao, Y.; Truhlar, D. G. Design of Density Functionals That Are Broadly Accurate for Thermochemistry, Thermochemical Kinetics, and Nonbonded Interactions. *J. Phys. Chem. A* **2005**, *109*, 5656–5667.

(30) Caldeweyher, E.; Bannwarth, C.; Grimme, S. Extension of the D3 Dispersion Coefficient Model. *J. Chem. Phys.* **2017**, *147*, 034112.

(31) Caldeweyher, E.; Ehlert, S.; Hansen, A.; Neugebauer, H.; Spicher, S.; Bannwarth, C.; Grimme, S. A Generally Applicable Atomic-Charge Dependent London Dispersion Correction. *J. Chem. Phys.* **2019**, *150*, 154122.

(32) Marenich, A. V.; Cramer, C. J.; Truhlar, D. G. Universal Solvation Model Based on Solute Electron Density and on a Continuum Model of the Solvent Defined by the Bulk Dielectric Constant and Atomic Surface Tensions. *J. Phys. Chem. B* **2009**, *113*, 6378–6396.

# NMR Characterization Data of the Synthesized Hydrazone Substrates

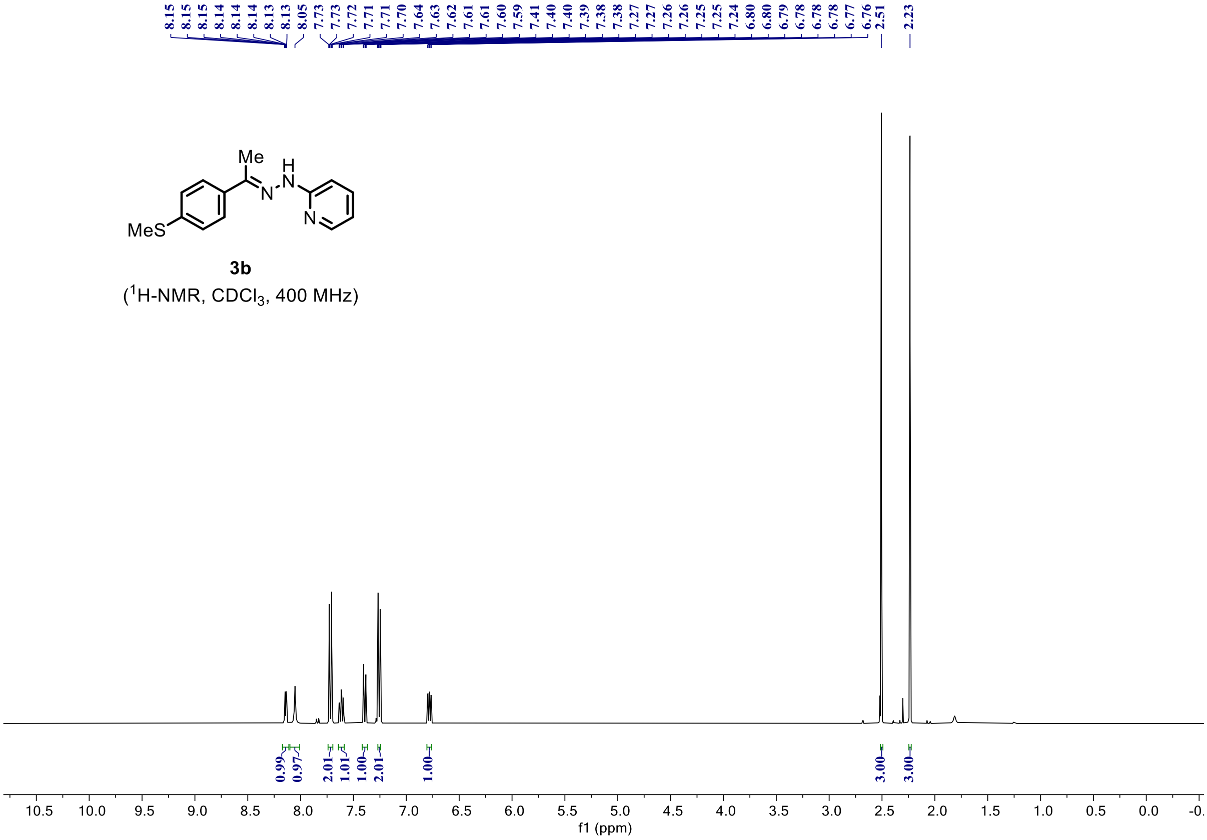


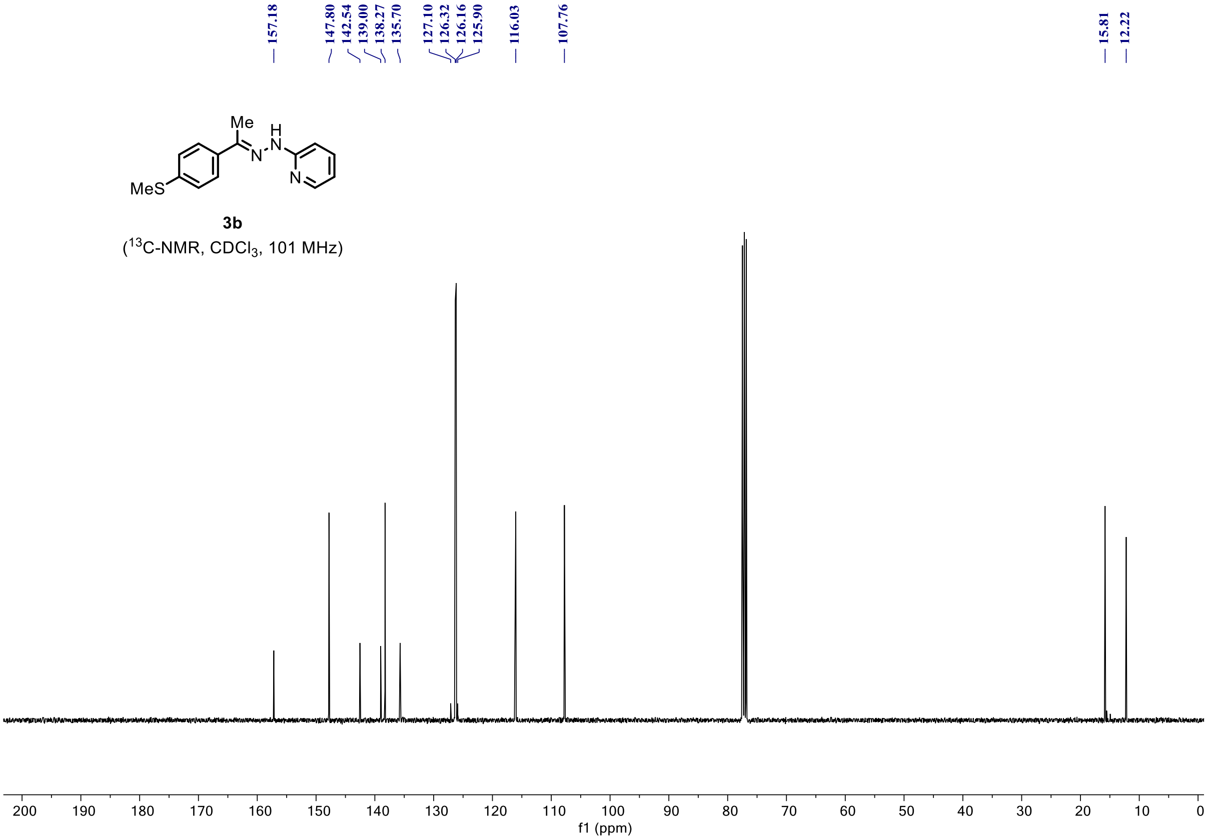

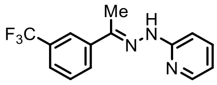

**3c**

($^1$H-NMR, $CDCl_3$, 300 MHz)

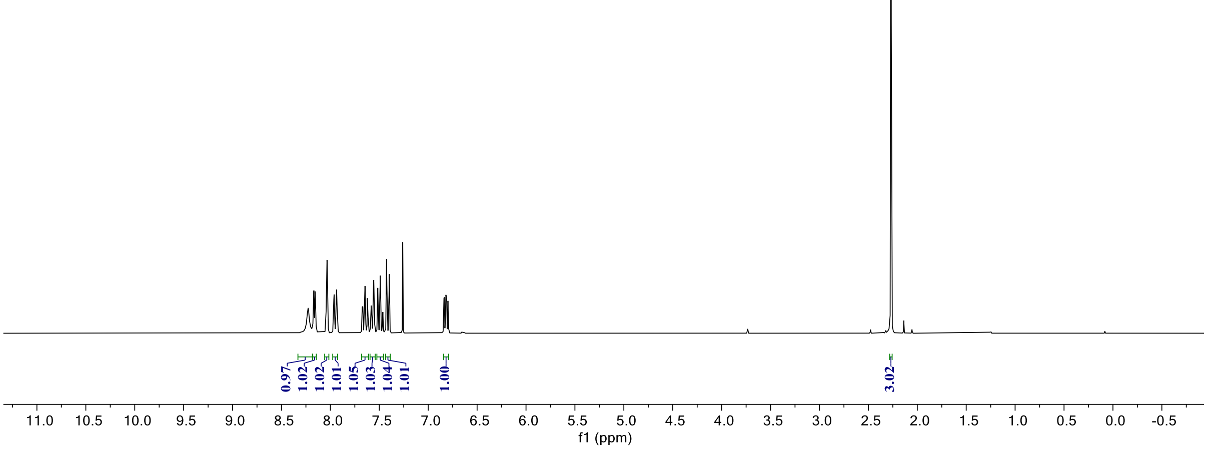


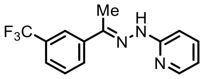

**3c**

($^{13}$C-NMR, $CDCl_3$, 75 MHz)

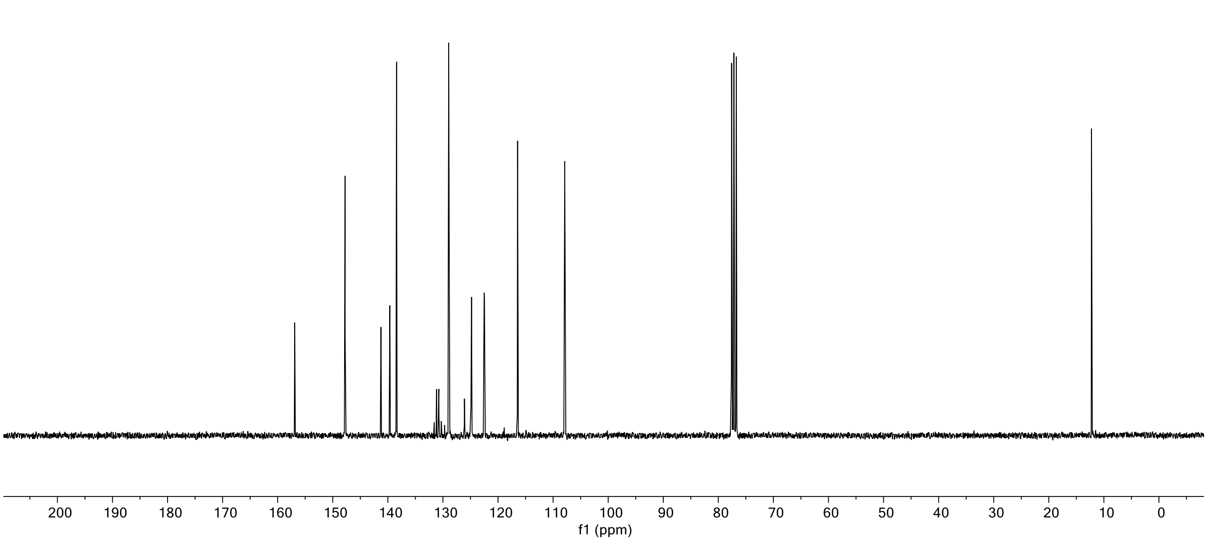

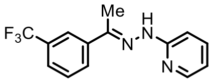


**3c**

($^{19}$F-NMR, $CDCl_3$, 282 MHz)

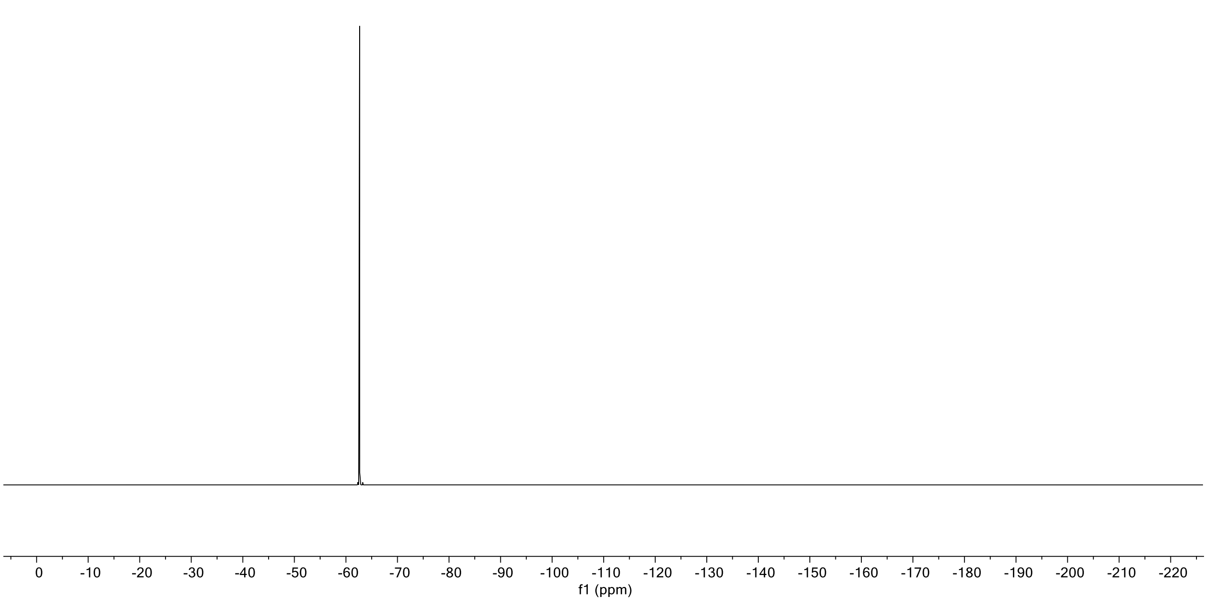

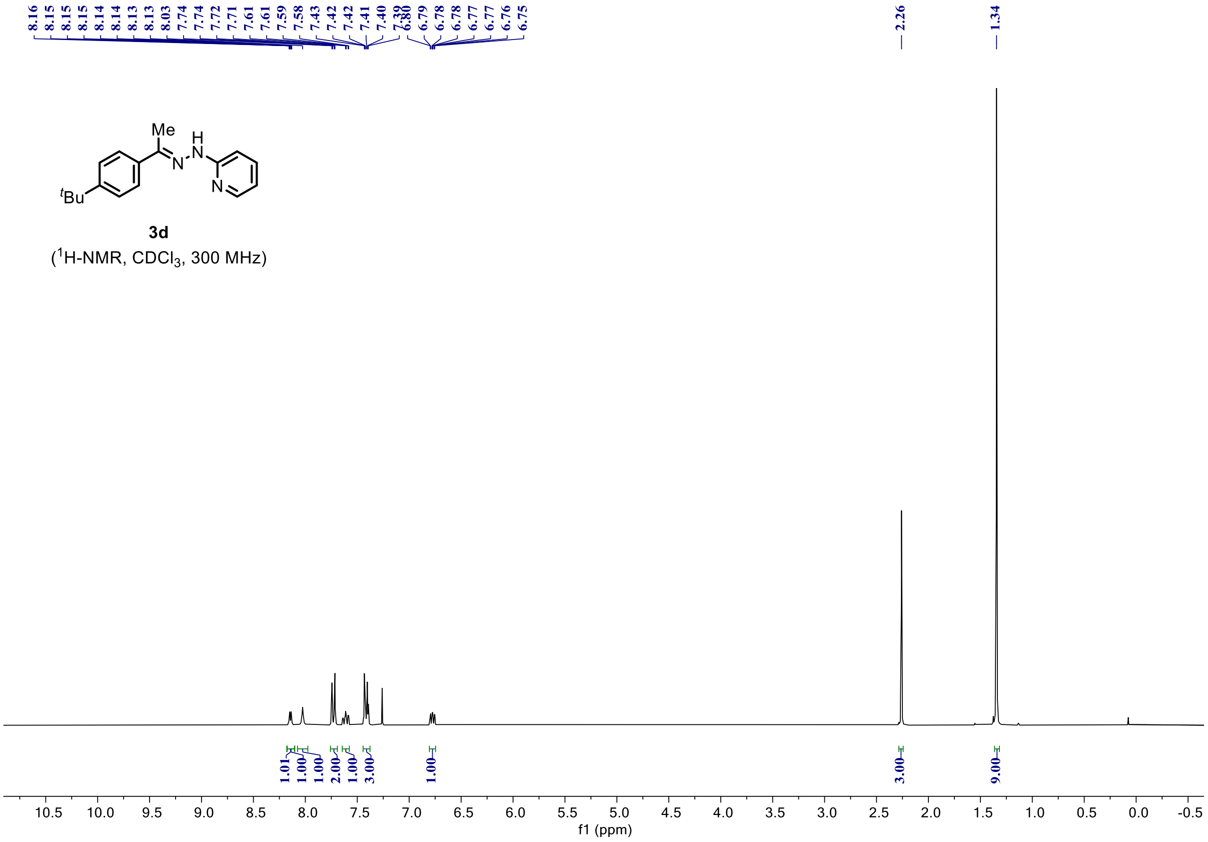
8.16 8.15 8.15 8.14 8.14 8.13 8.13 8.03 7.74 7.74 7.72 7.71 7.61 7.61 7.59 7.58 7.43 7.42 7.42 7.41 7.40 7.39 6.80 6.79 6.78 6.78 6.77 6.77 6.76 6.75
2.26
1.34
Me
H
N
N
N
tBu
3d
(1H-NMR, CDCl3, 300 MHz)
1.01 1.00 1.00 2.00 1.00 3.00
1.00
3.00
9.00
10.5 10.0 9.5 9.0 8.5 8.0 7.5 7.0 6.5 6.0 5.5 5.0 4.5 4.0 3.5 3.0 2.5 2.0 1.5 1.0 0.5 0.0 -0.5
f1 (ppm)

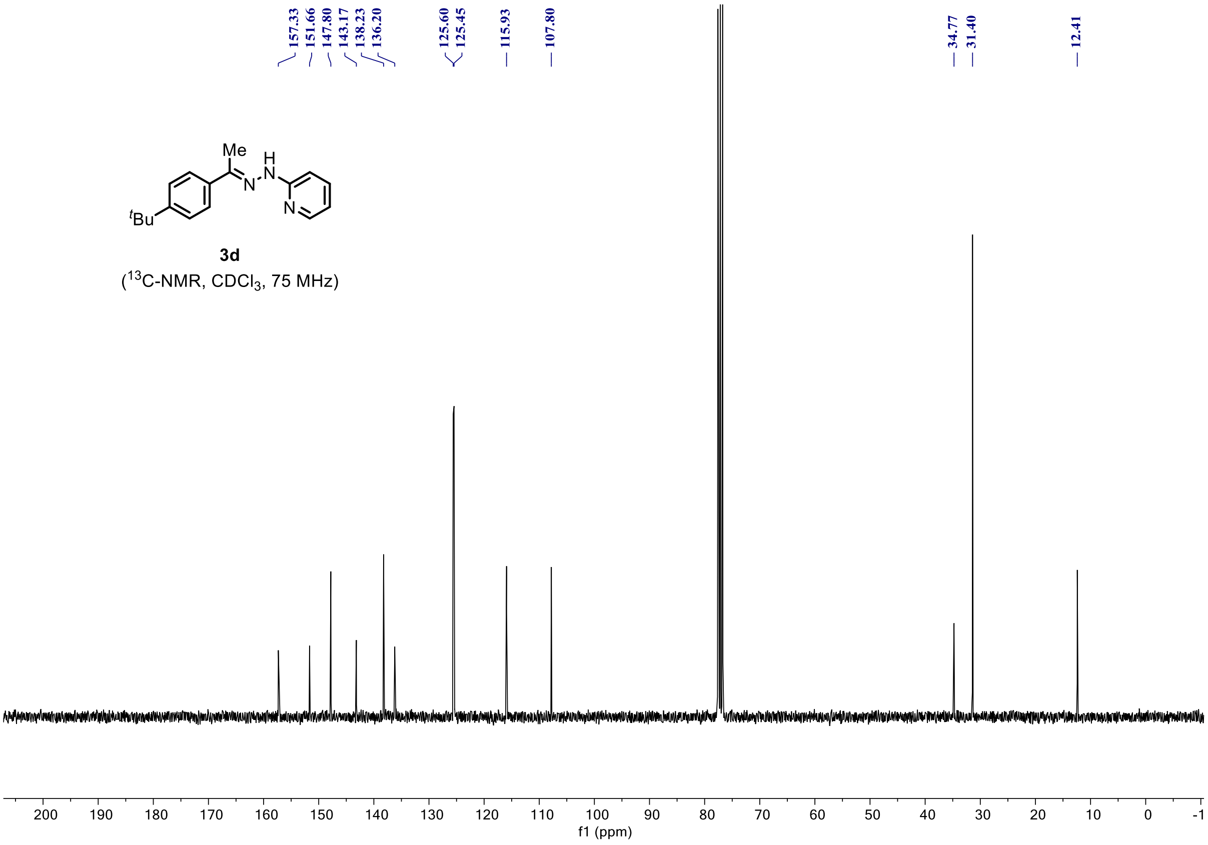
157.33 151.66 147.80 143.17 138.23 136.20
125.60 125.45
115.93
107.80
34.77 31.40
12.41
Me
H
N
N
N
tBu
3d
(13C-NMR, CDCl3, 75 MHz)
200 190 180 170 160 150 140 130 120 110 100 90 80 70 60 50 40 30 20 10 0 -1
f1 (ppm)

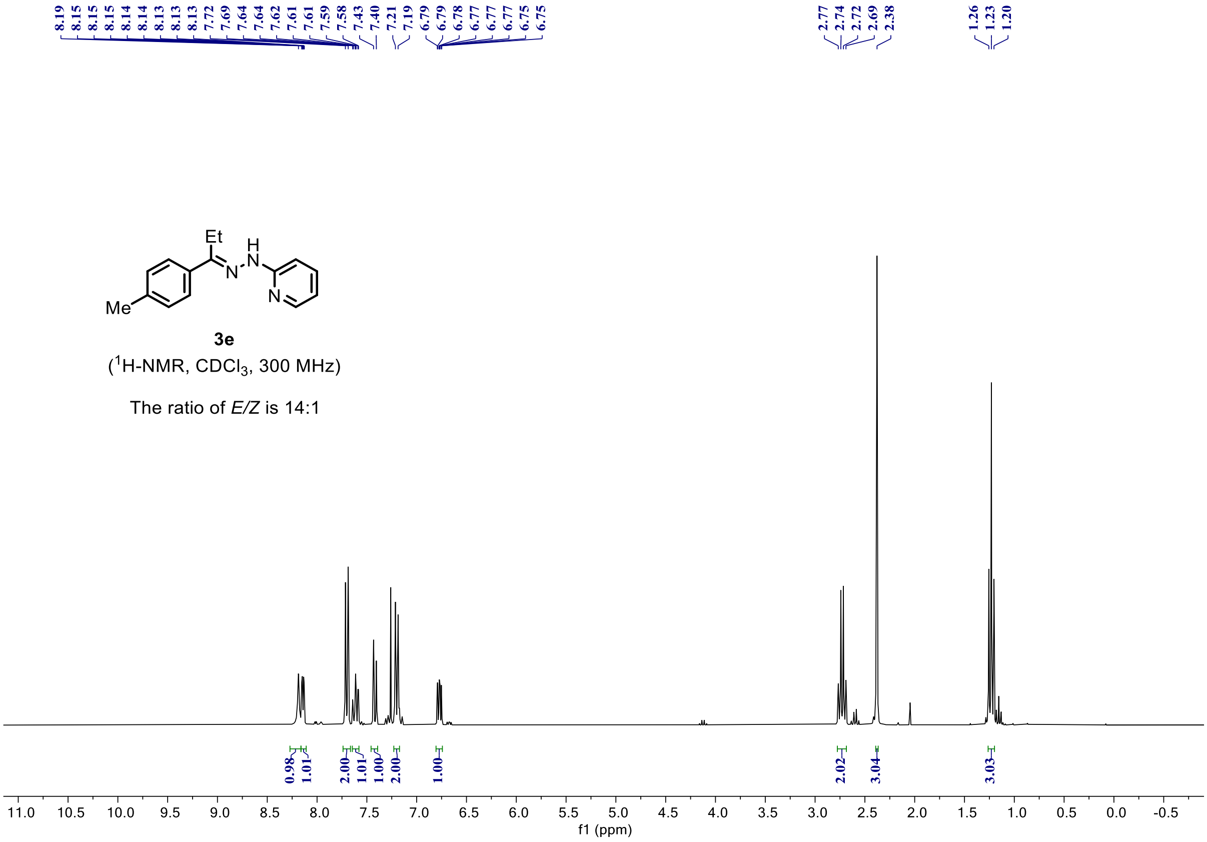


**3e**

($^{1}$H-NMR, $CDCl_3$, 300 MHz)

The ratio of *E/Z* is 14:1

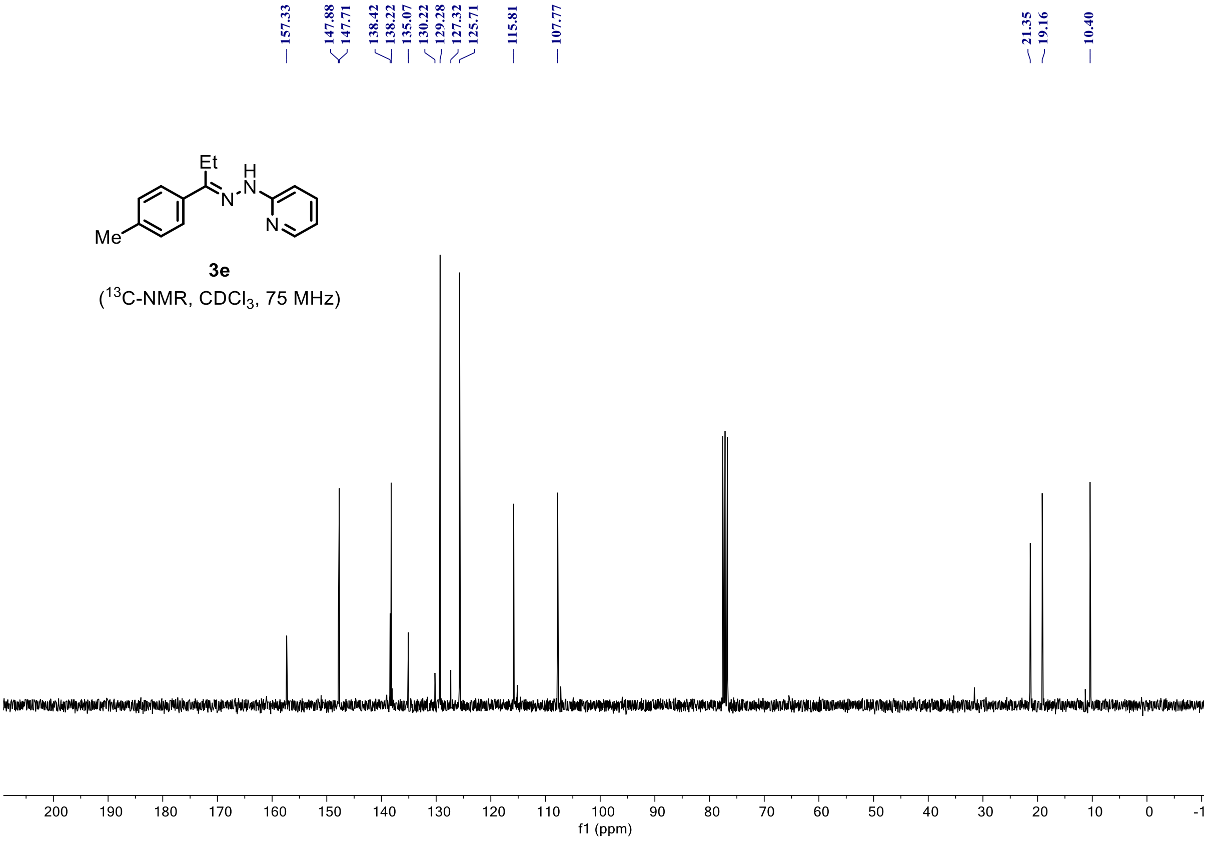


**3e**

($^{13}$C-NMR, $CDCl_3$, 75 MHz)

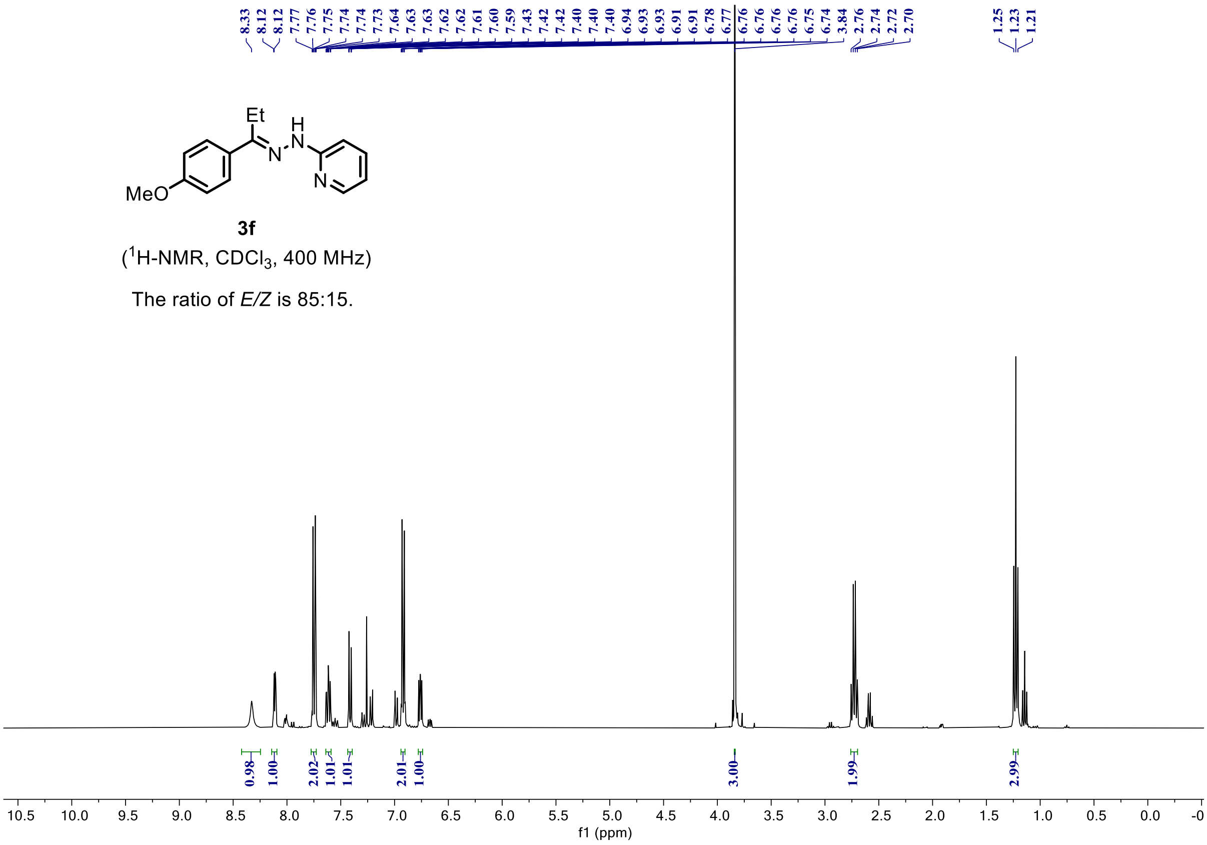


**3f**

($^{1}$H-NMR, $CDCl_3$, 400 MHz)

The ratio of *E/Z* is 85:15.

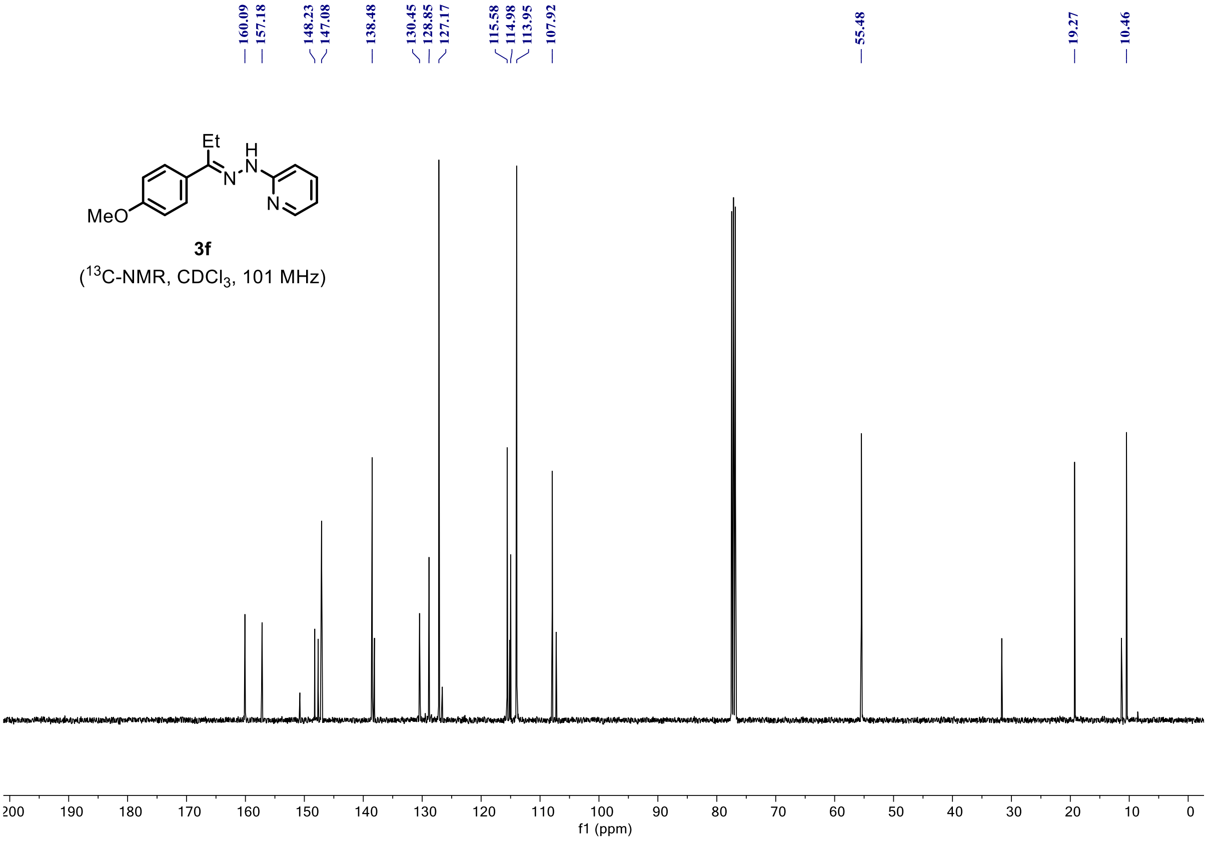


**3f**

($^{13}$C-NMR, $CDCl_3$, 101 MHz)

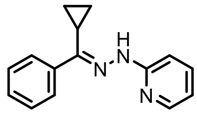

**3g**
($^1$H-NMR, $CDCl_3$, 400 MHz)

The ratio of *E/Z* is 3:1

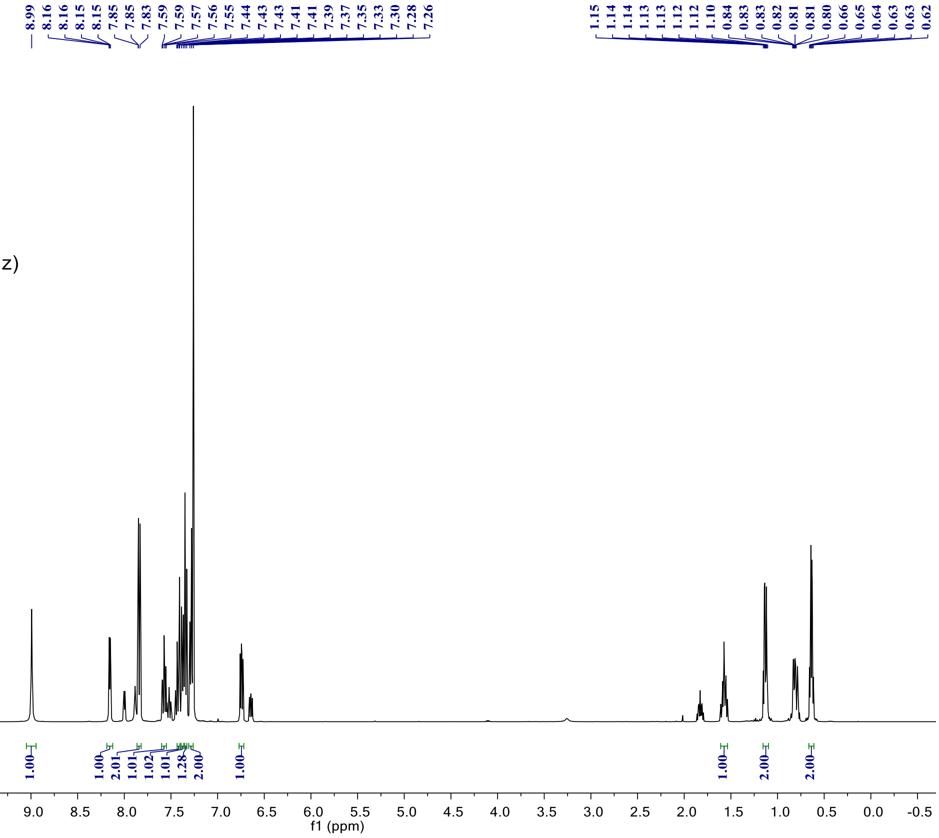


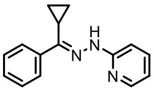

**3g**
($^{13}$C-NMR, $CDCl_3$, 101 MHz)

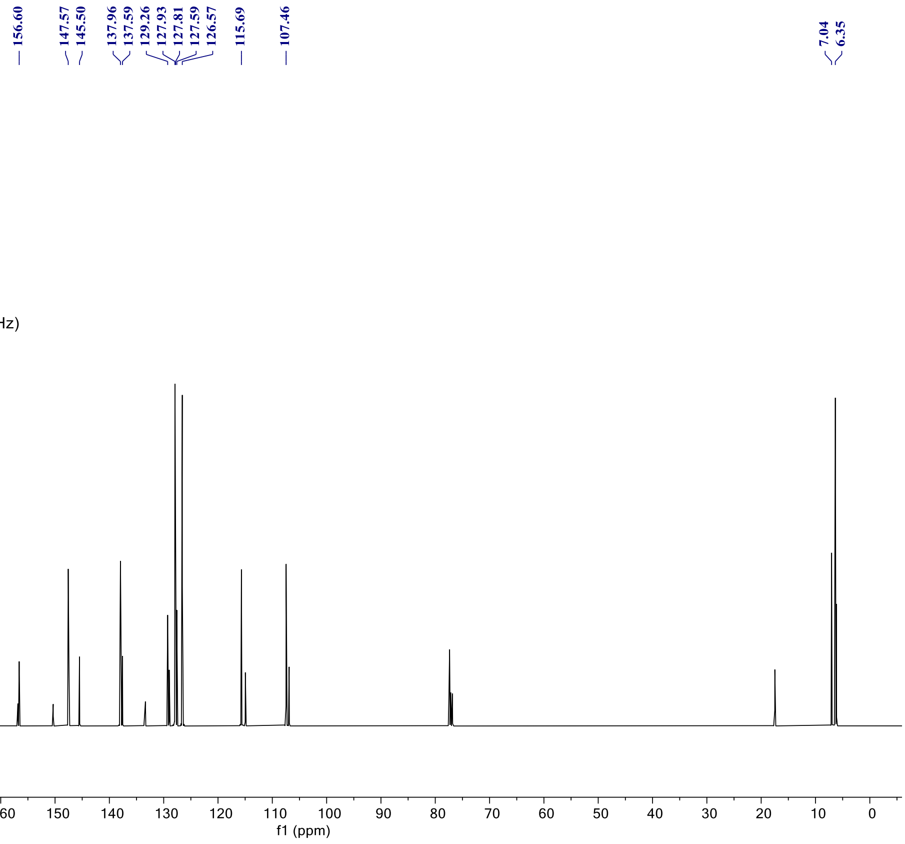

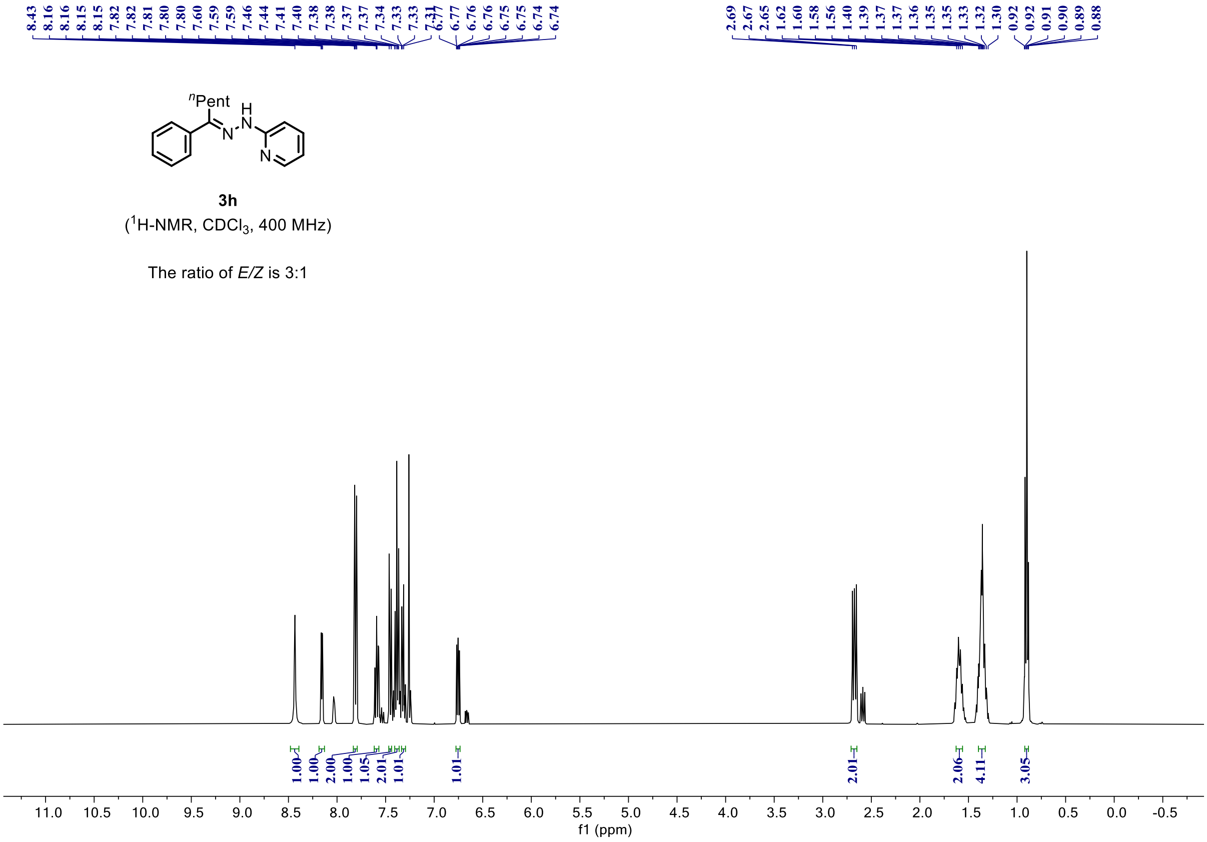


**3h**
($^{1}$H-NMR, $CDCl_3$, 400 MHz)

The ratio of *E/Z* is 3:1

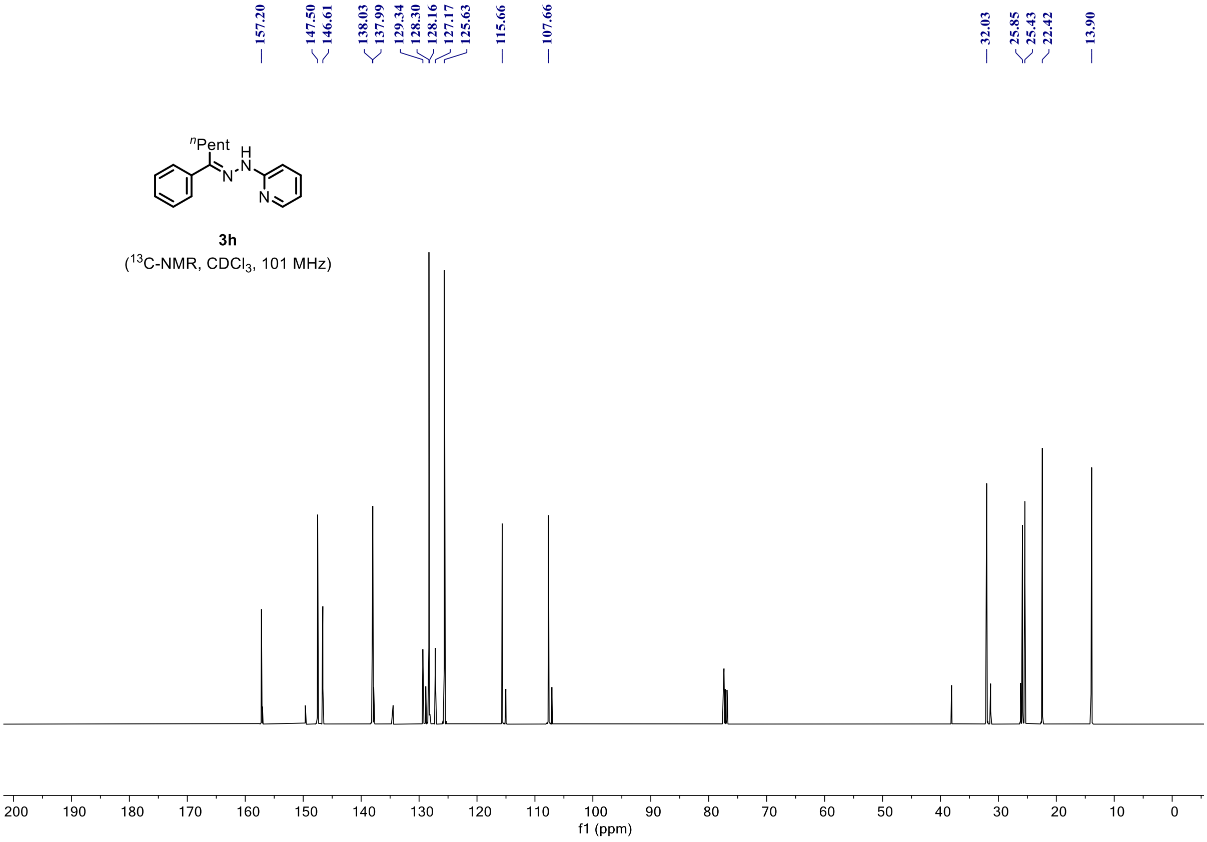


**3h**
($^{13}$C-NMR, $CDCl_3$, 101 MHz)

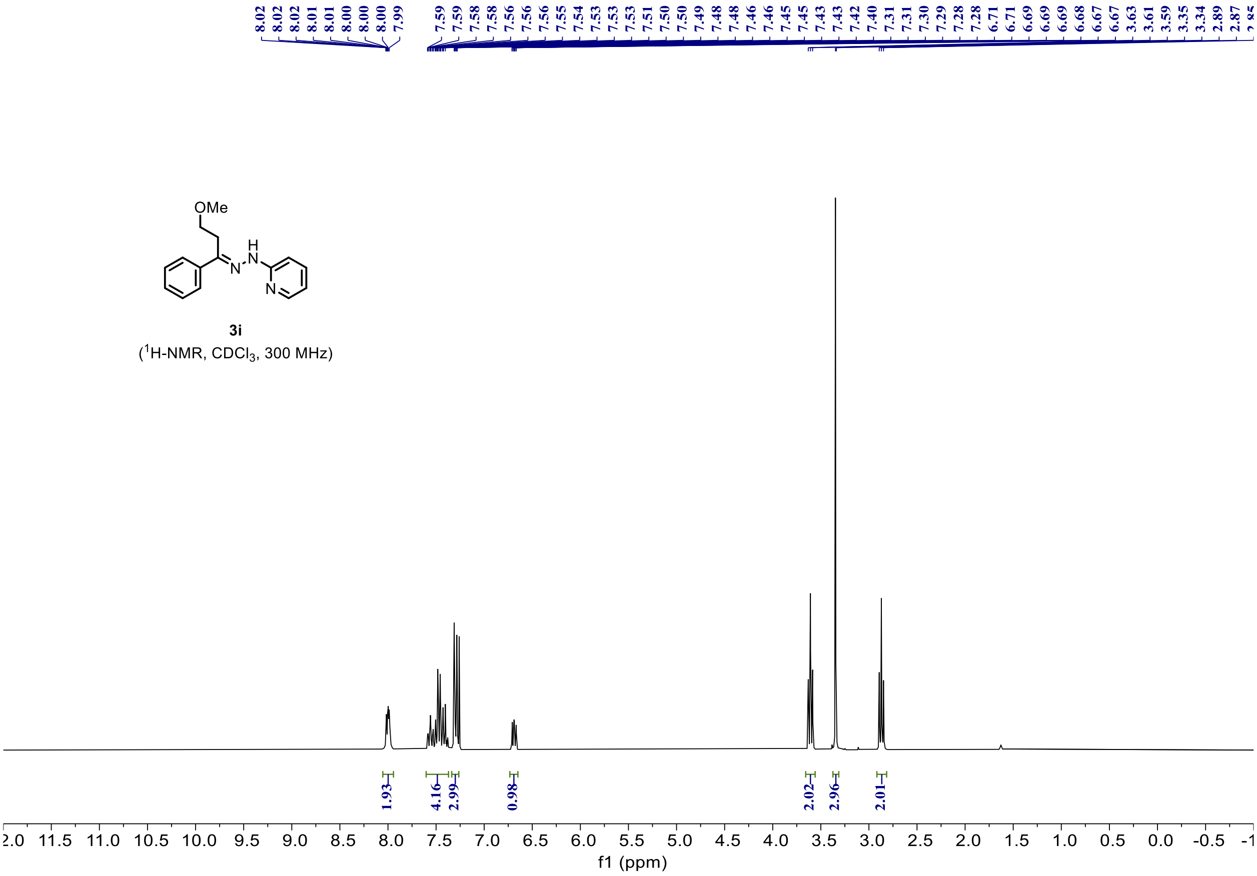

3i
($^1$H-NMR, $CDCl_3$, 300 MHz)

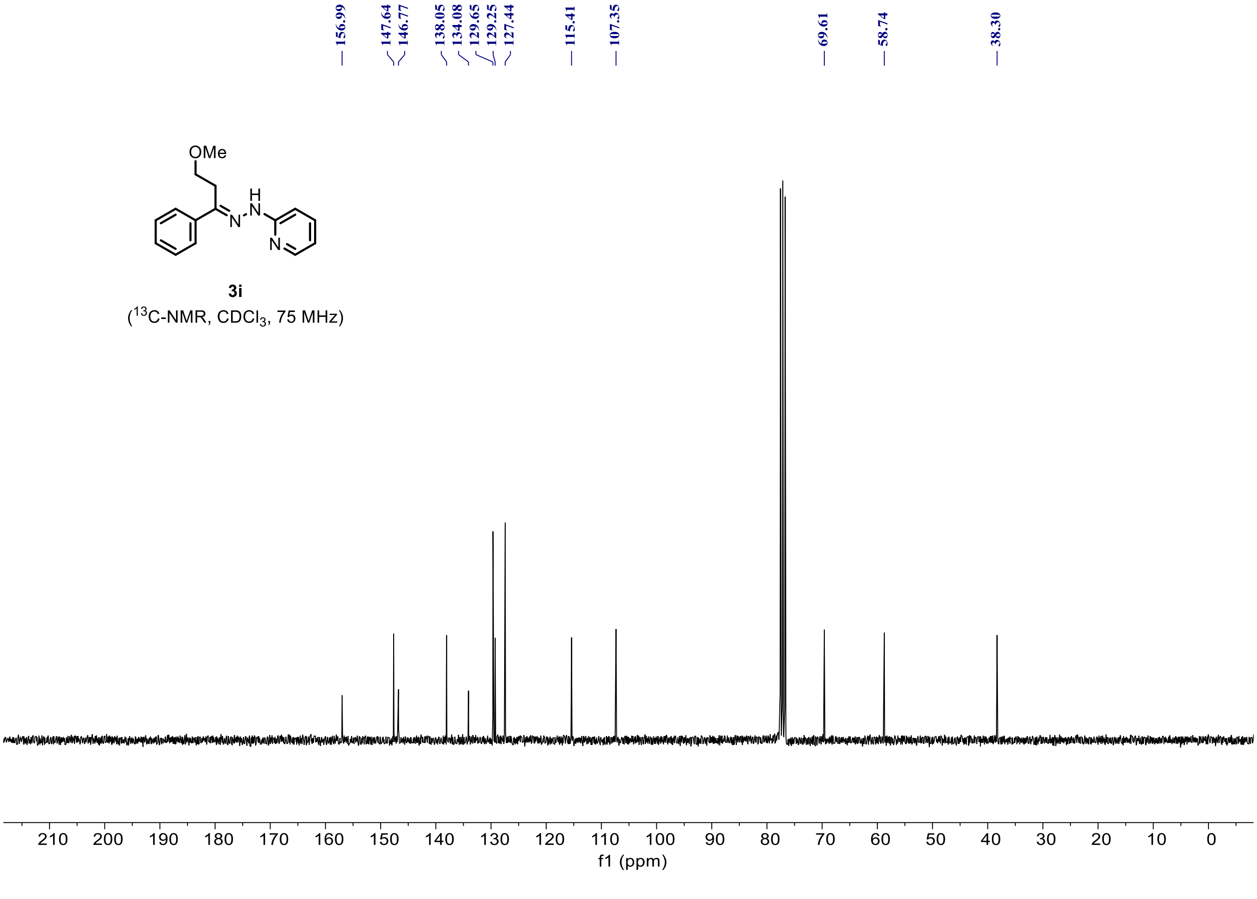

3i
($^{13}$C-NMR, $CDCl_3$, 75 MHz)

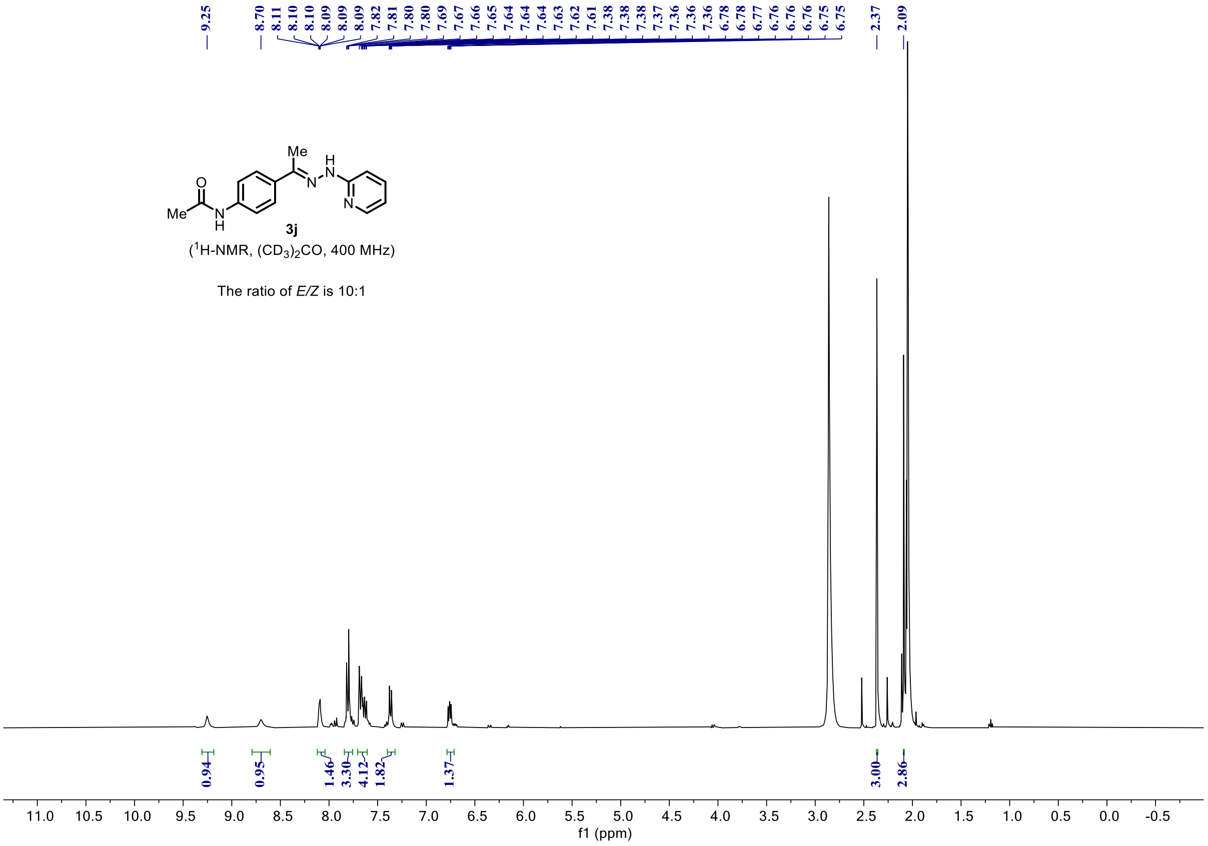

3j
(1H-NMR, (CD3)2CO, 400 MHz)
The ratio of E/Z is 10:1
f1 (ppm)


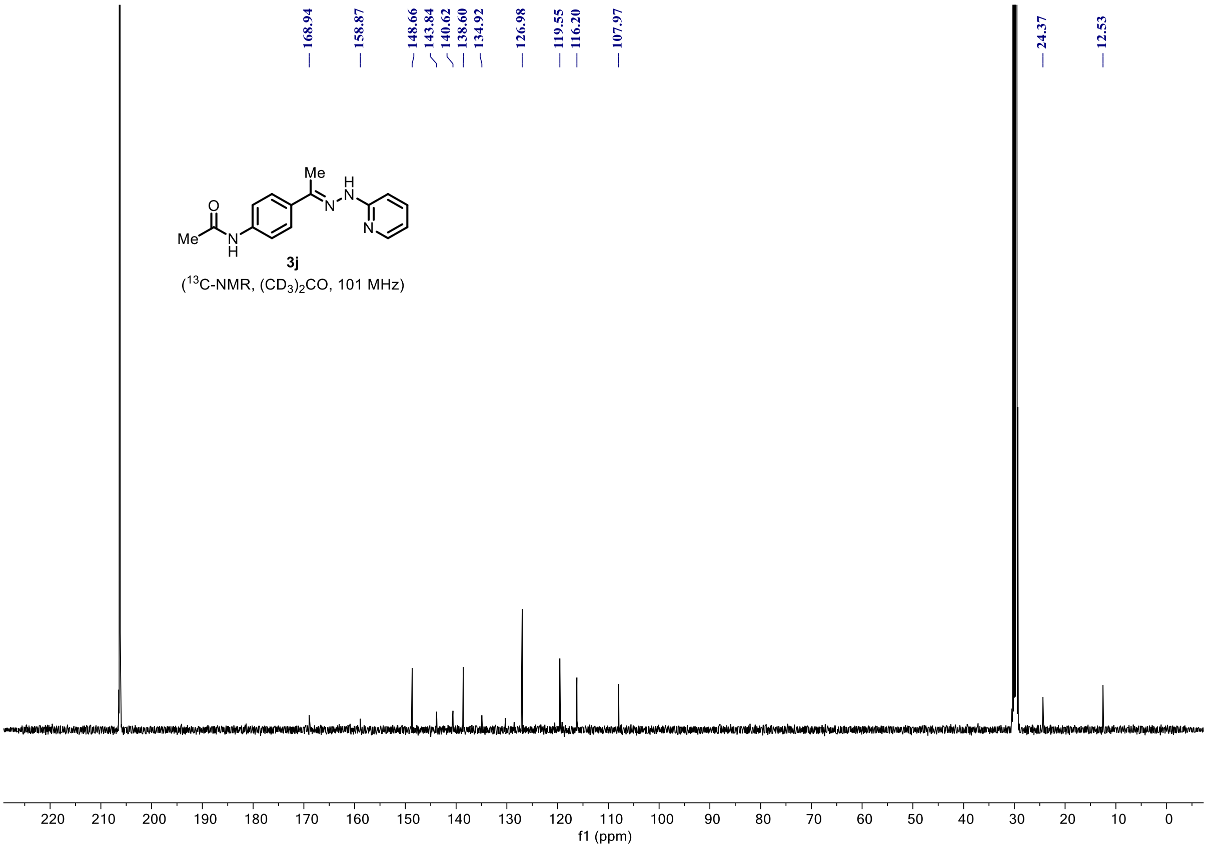

3j
(13C-NMR, (CD3)2CO, 101 MHz)
f1 (ppm)

# NMR Characterization Data of the Synthesized Ligands

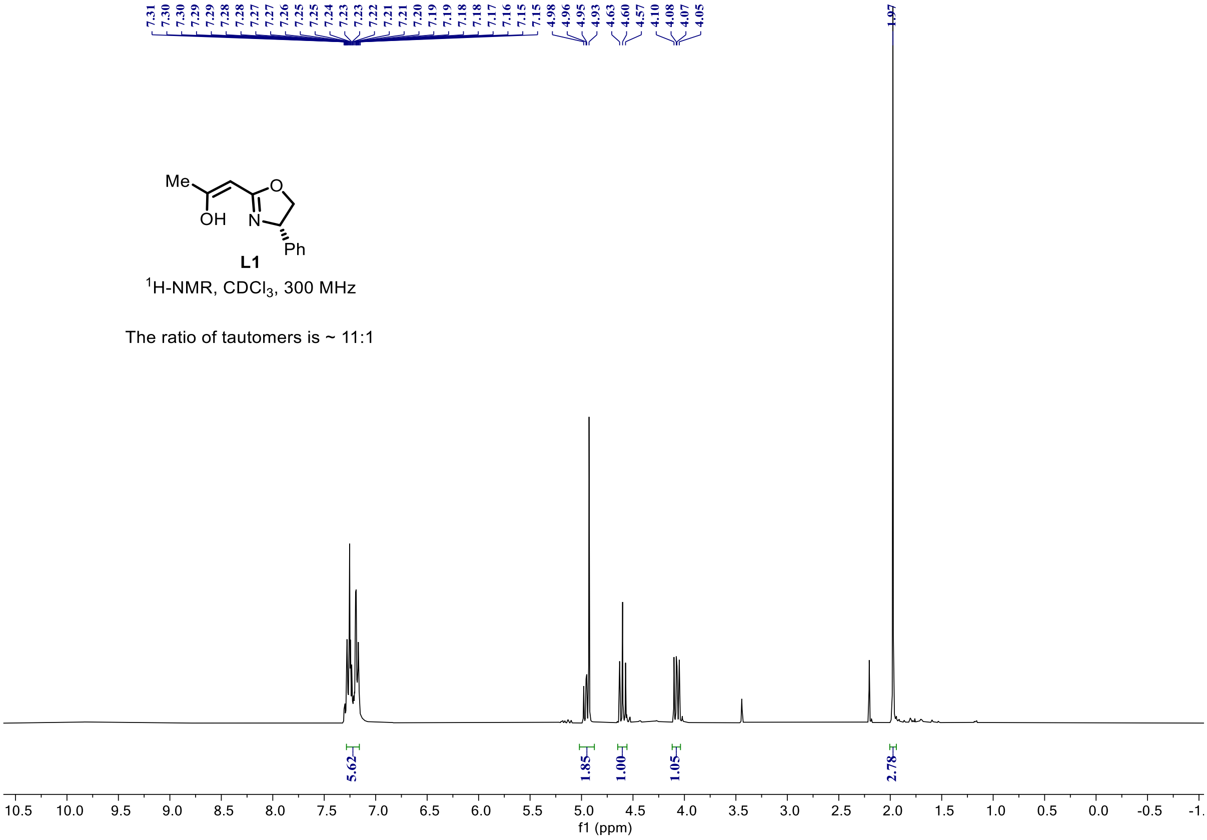


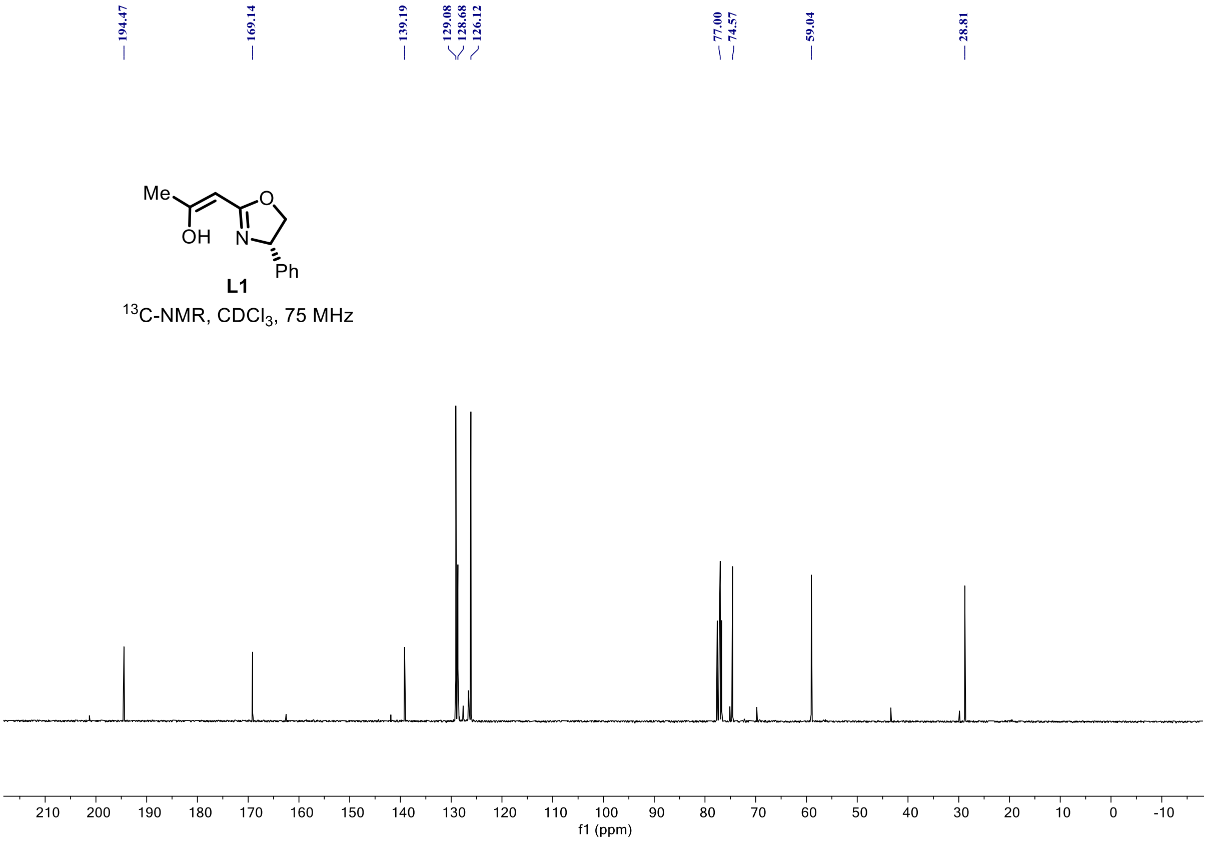

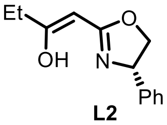

**L2**

$^1$H-NMR, $CDCl_3$, 400 MHz

The ratio of tautomers is ~ 7:1

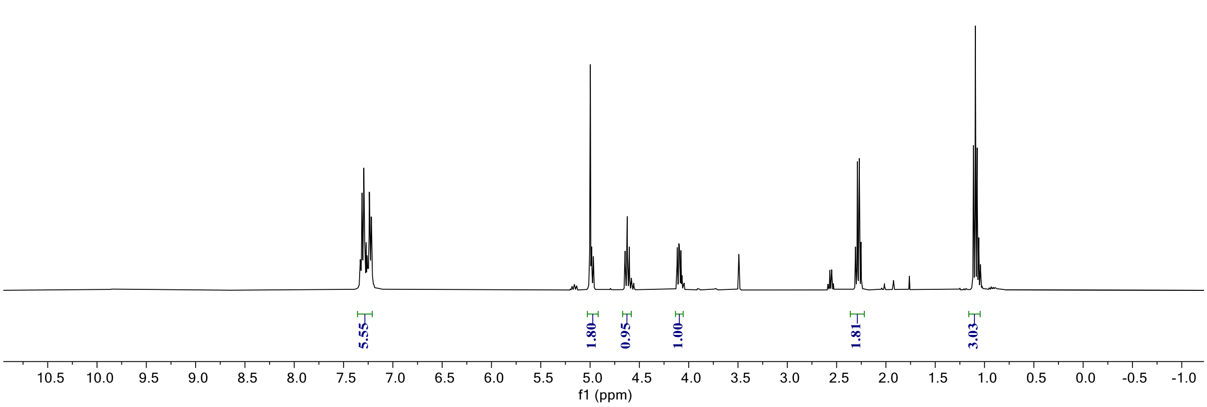


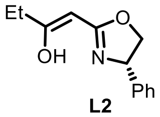

**L2**

$^{13}$C-NMR, $CDCl_3$, 101 MHz

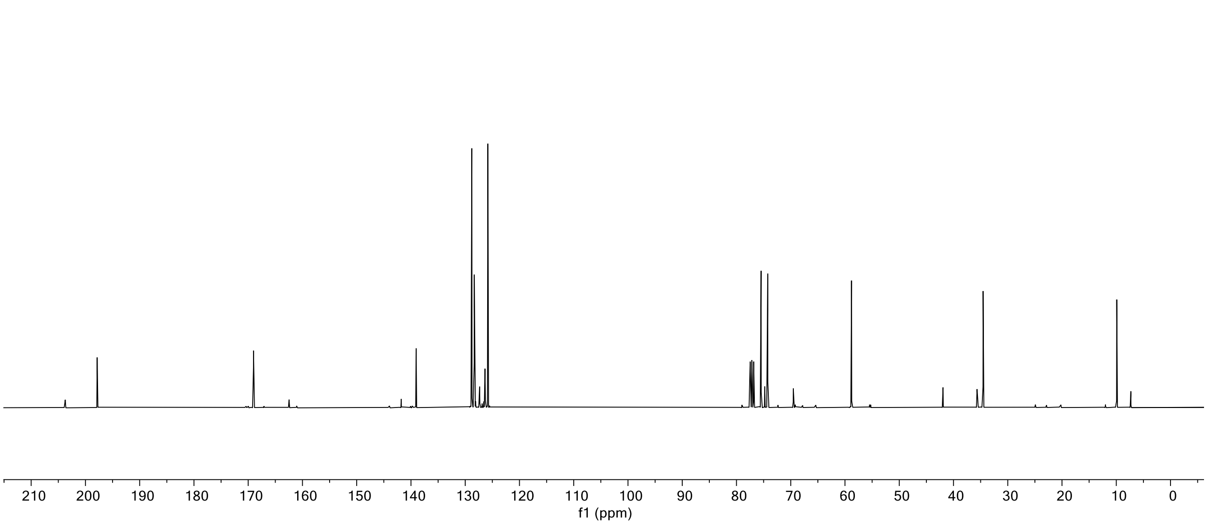

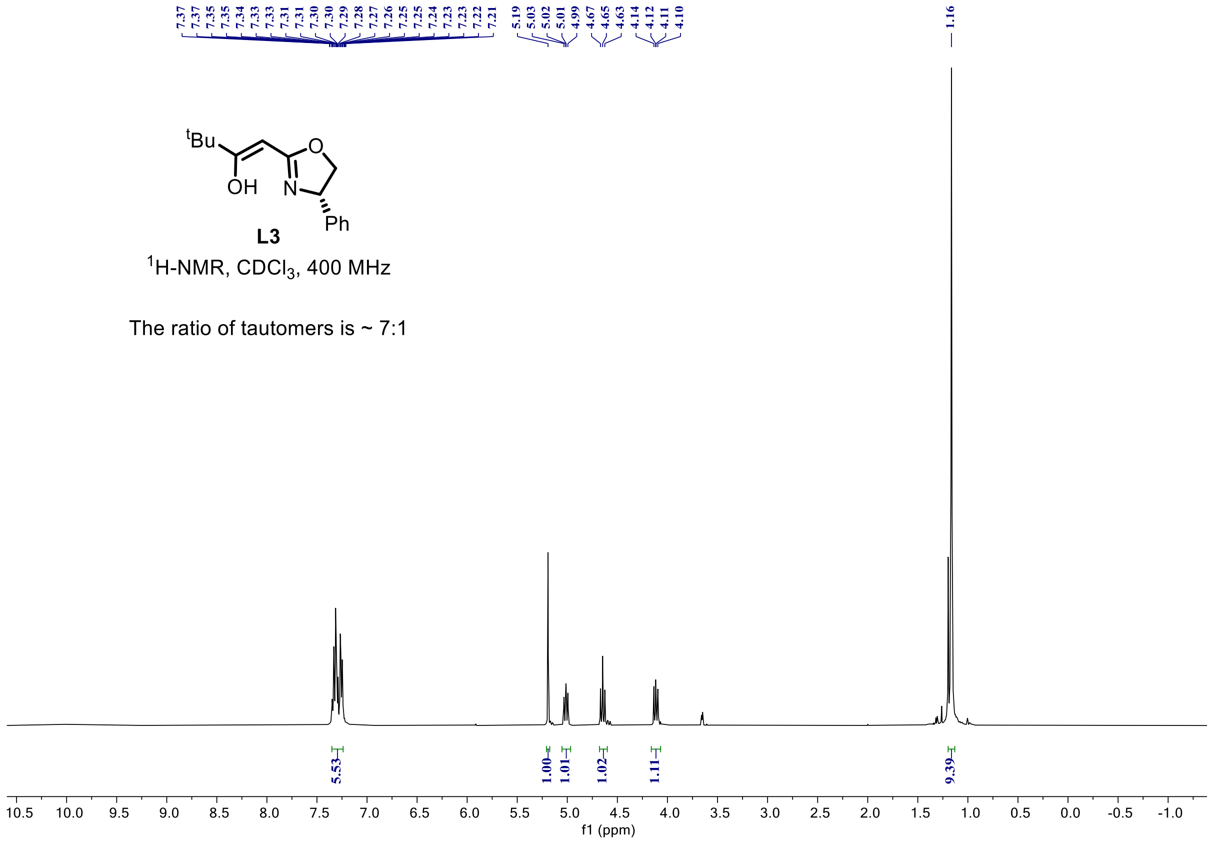
7.37 7.37 7.35 7.35 7.34 7.33 7.33 7.31 7.31 7.30 7.30 7.29 7.28 7.27 7.26 7.25 7.25 7.24 7.23 7.23 7.22 7.21
5.19 5.03 5.02 5.01 4.99 4.67 4.65 4.63 4.14 4.12 4.11 4.10
1.16
tBu
OH
N
O
Ph
L3
1H-NMR, CDCl3, 400 MHz
The ratio of tautomers is ~ 7:1
5.53
1.00
1.01
1.02
1.11
9.39
10.5 10.0 9.5 9.0 8.5 8.0 7.5 7.0 6.5 6.0 5.5 5.0 4.5 4.0 3.5 3.0 2.5 2.0 1.5 1.0 0.5 0.0 -0.5 -1.0
f1 (ppm)

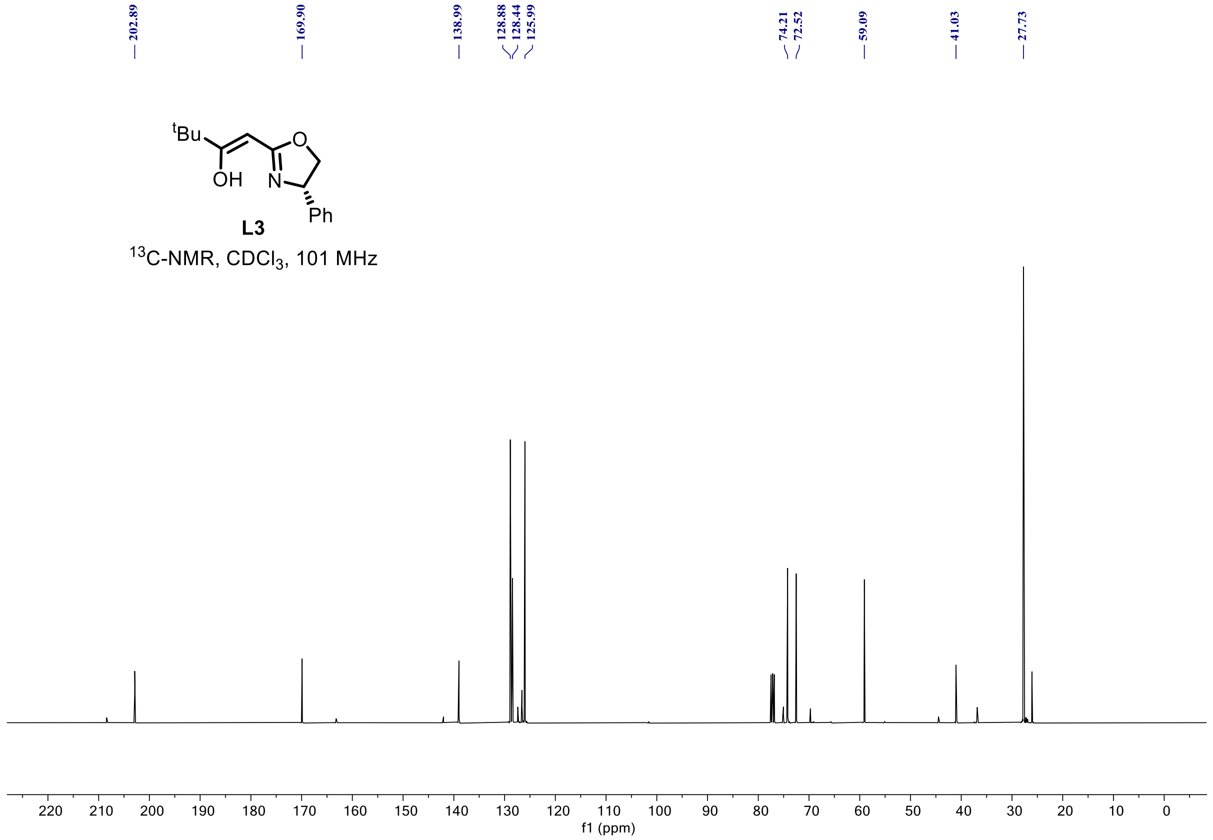
202.89
169.90
138.99
128.88 128.44 125.99
74.21 72.52
59.09
41.03
27.73
tBu
OH
N
O
Ph
L3
13C-NMR, CDCl3, 101 MHz
220 210 200 190 180 170 160 150 140 130 120 110 100 90 80 70 60 50 40 30 20 10 0
f1 (ppm)

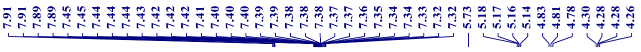


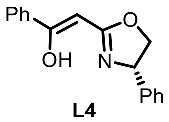


**L4**

$^1$H-NMR, $CDCl_3$, 400 MHz

The ratio of tautomers is ~ 11:1

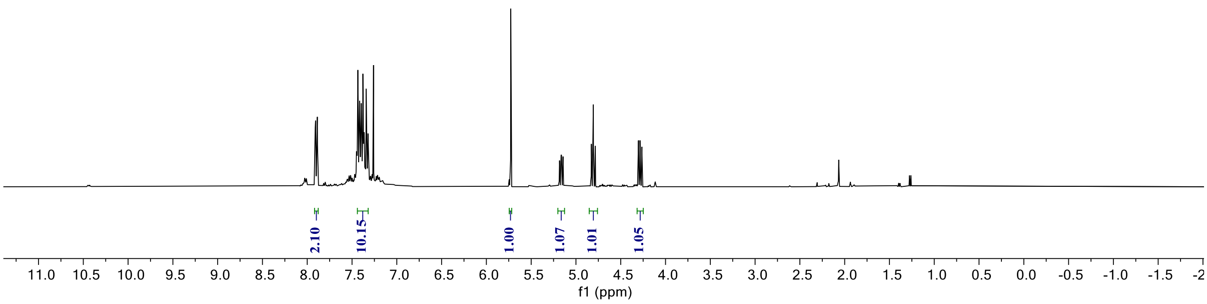


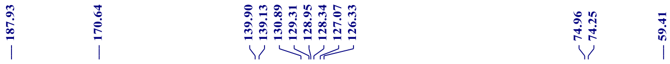


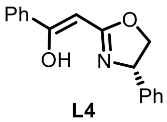


**L4**

$^{13}$C-NMR, $CDCl_3$, 101 MHz

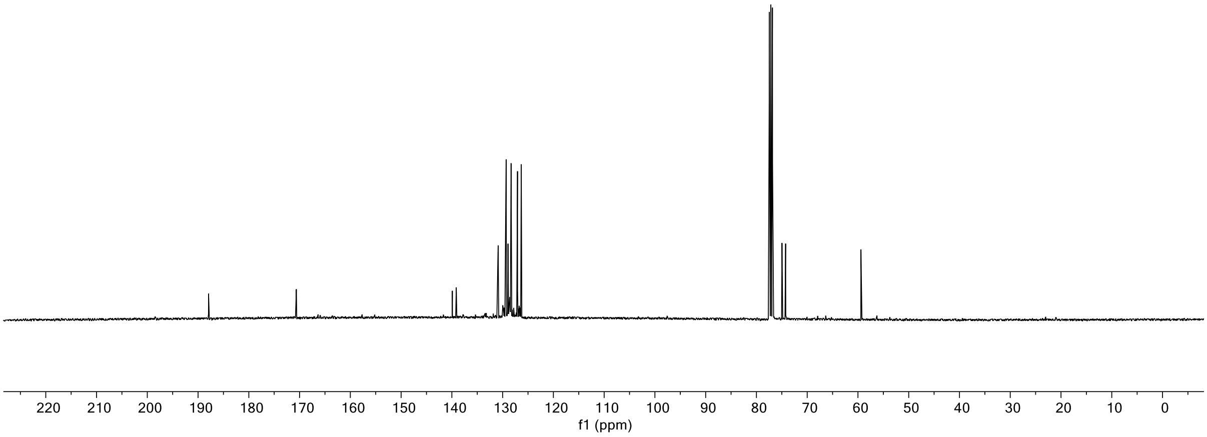

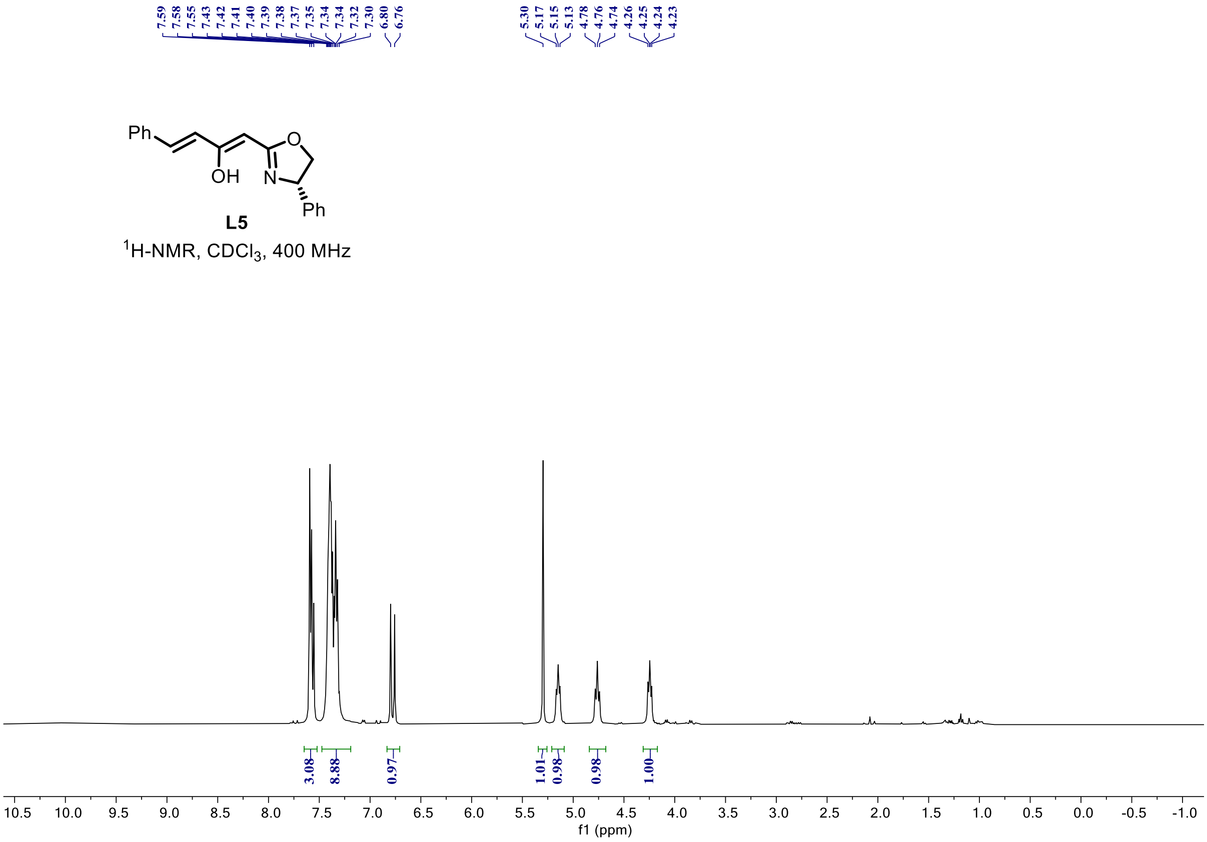


**L5**

$^{1}$H-NMR, $CDCl_3$, 400 MHz

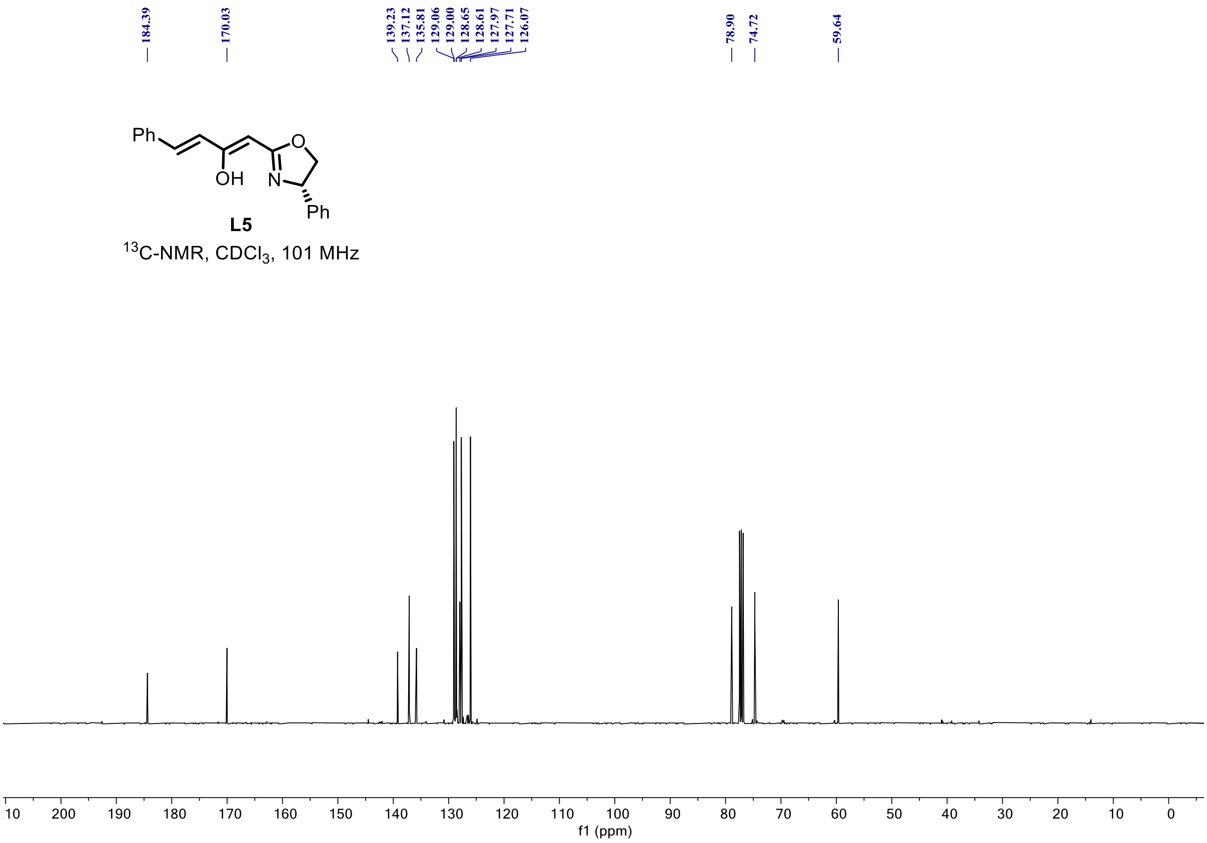


**L5**

$^{13}$C-NMR, $CDCl_3$, 101 MHz

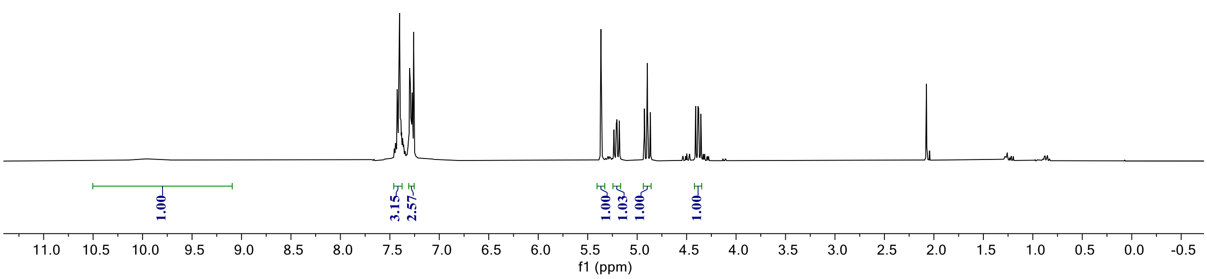


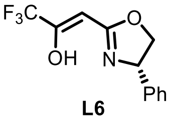


**L6**

$^{1}$H-NMR, $CDCl_3$, 300 MHz

The ratio of tautomers is ~ 7:1

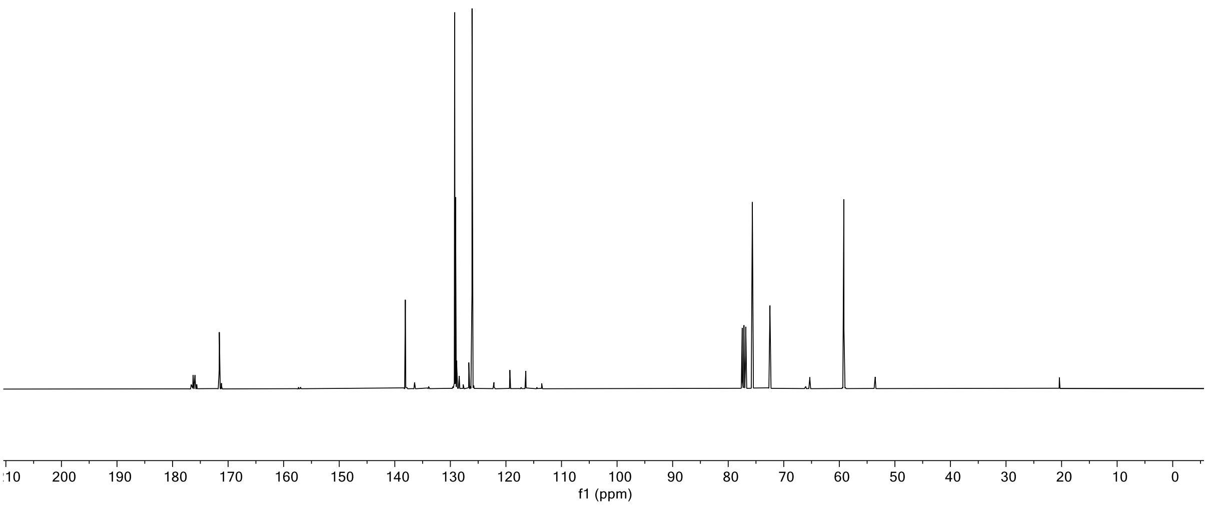


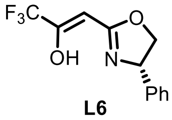


**L6**

$^{13}$C-NMR, $CDCl_3$, 101 MHz

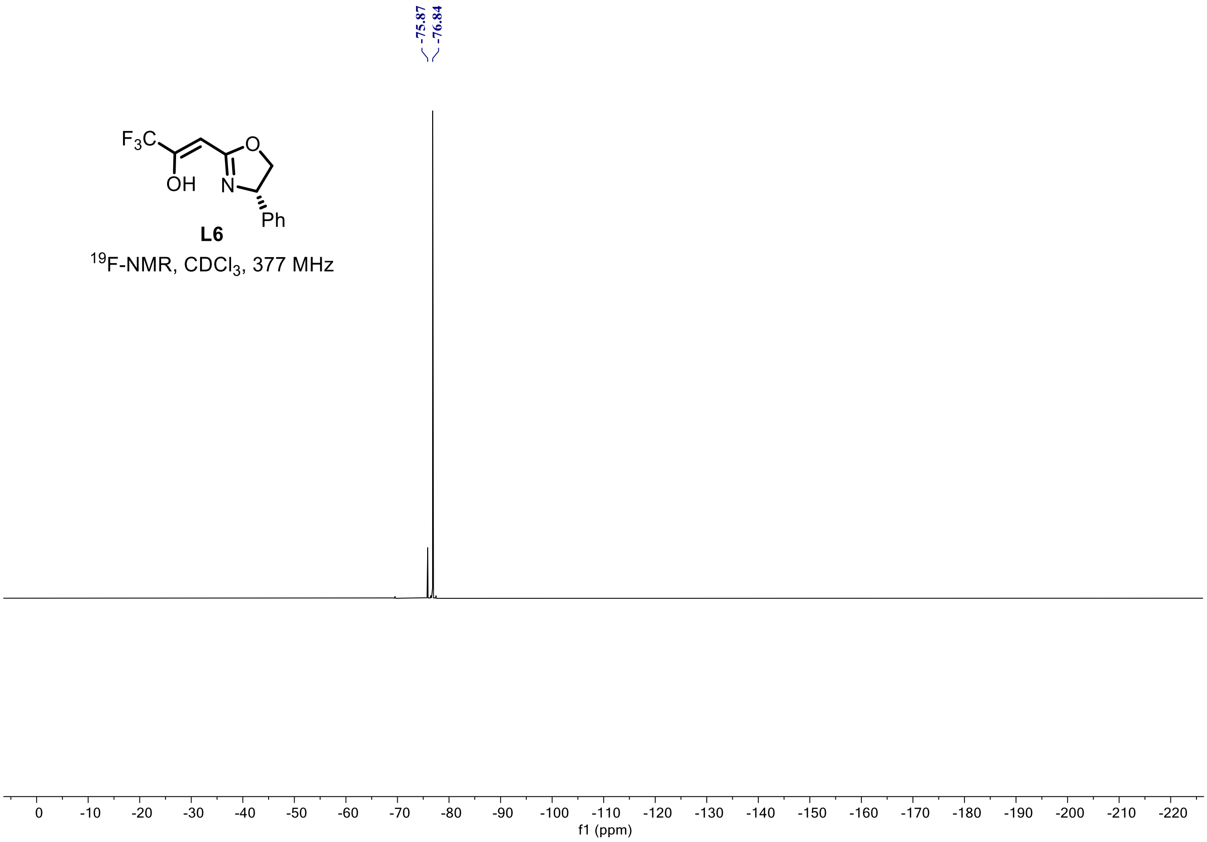

-75.87
-76.84
F3C
OH
N
O
Ph
L6
19F-NMR, CDCl3, 377 MHz
0
-10
-20
-30
-40
-50
-60
-70
-80
-90
-100
-110
-120
-130
-140
-150
-160
-170
-180
-190
-200
-210
-220
f1 (ppm)

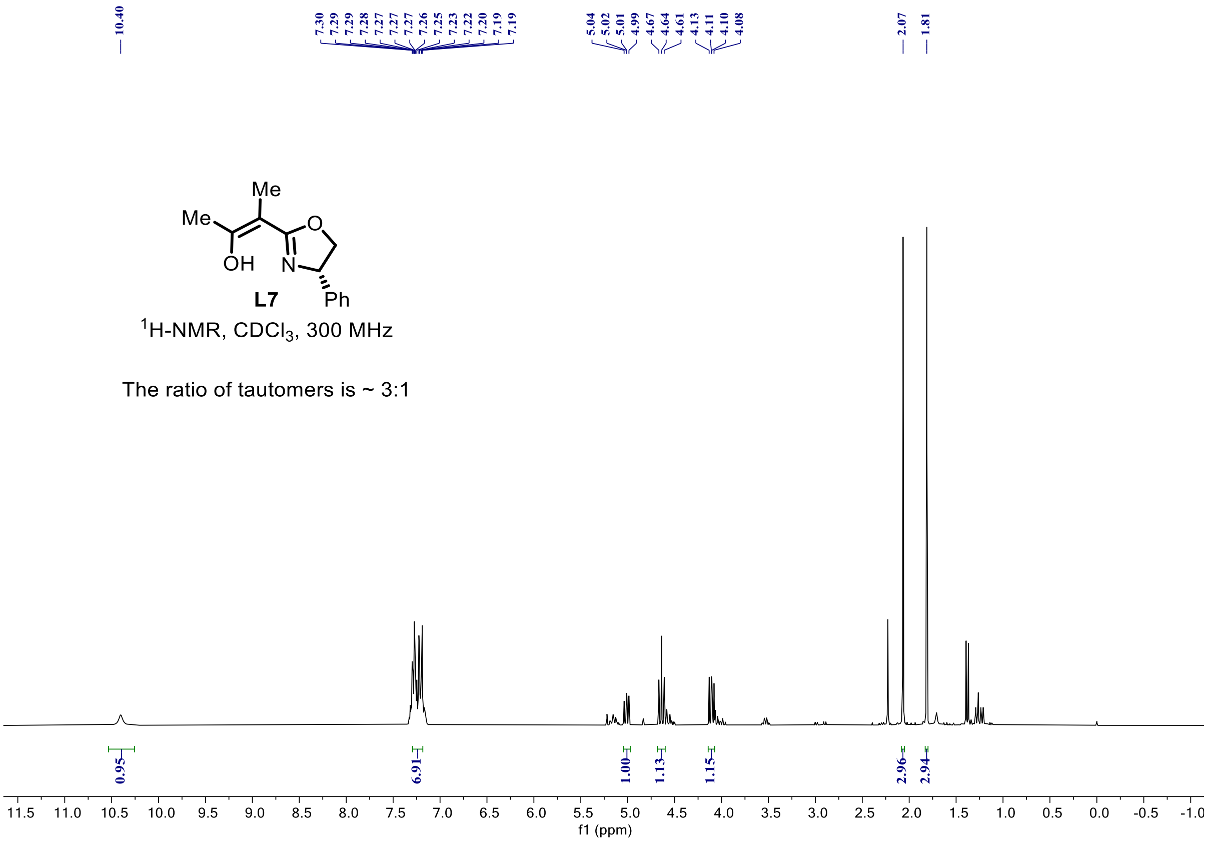
10.40
7.30 7.29 7.29 7.28 7.27 7.27 7.27 7.26 7.25 7.23 7.22 7.20 7.19 7.19
5.04 5.02 5.01 4.99 4.67 4.64 4.61 4.13 4.11 4.10 4.08
2.07 1.81
Me
Me
O
OH
N
L7
Ph
$^{1}$H-NMR, CDCl$_3$, 300 MHz
The ratio of tautomers is ~ 3:1
0.95 6.91 1.00 1.13 1.15 2.96 2.94
f1 (ppm)

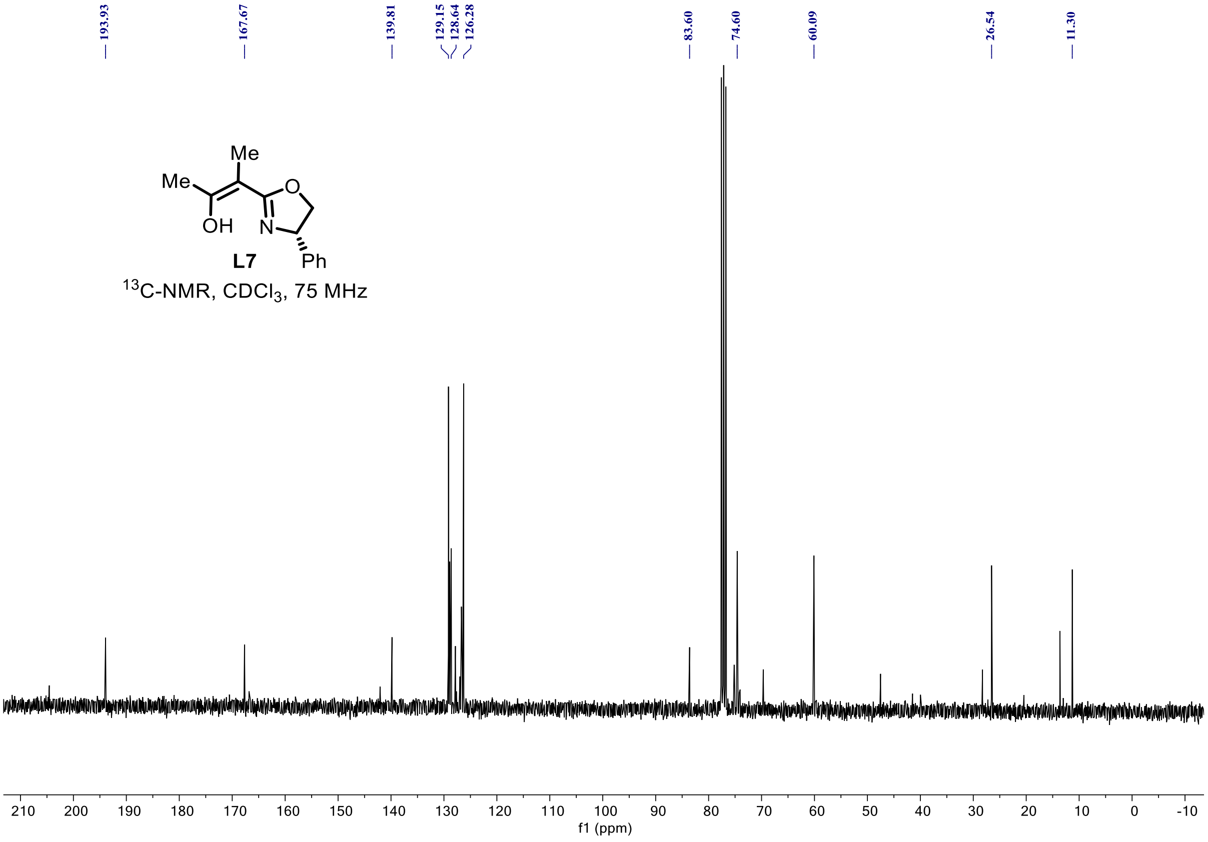
193.93
167.67
139.81
129.15 128.64 126.28
83.60
74.60
60.09
26.54
11.30
Me
Me
O
OH
N
L7
Ph
$^{13}$C-NMR, CDCl$_3$, 75 MHz
f1 (ppm)

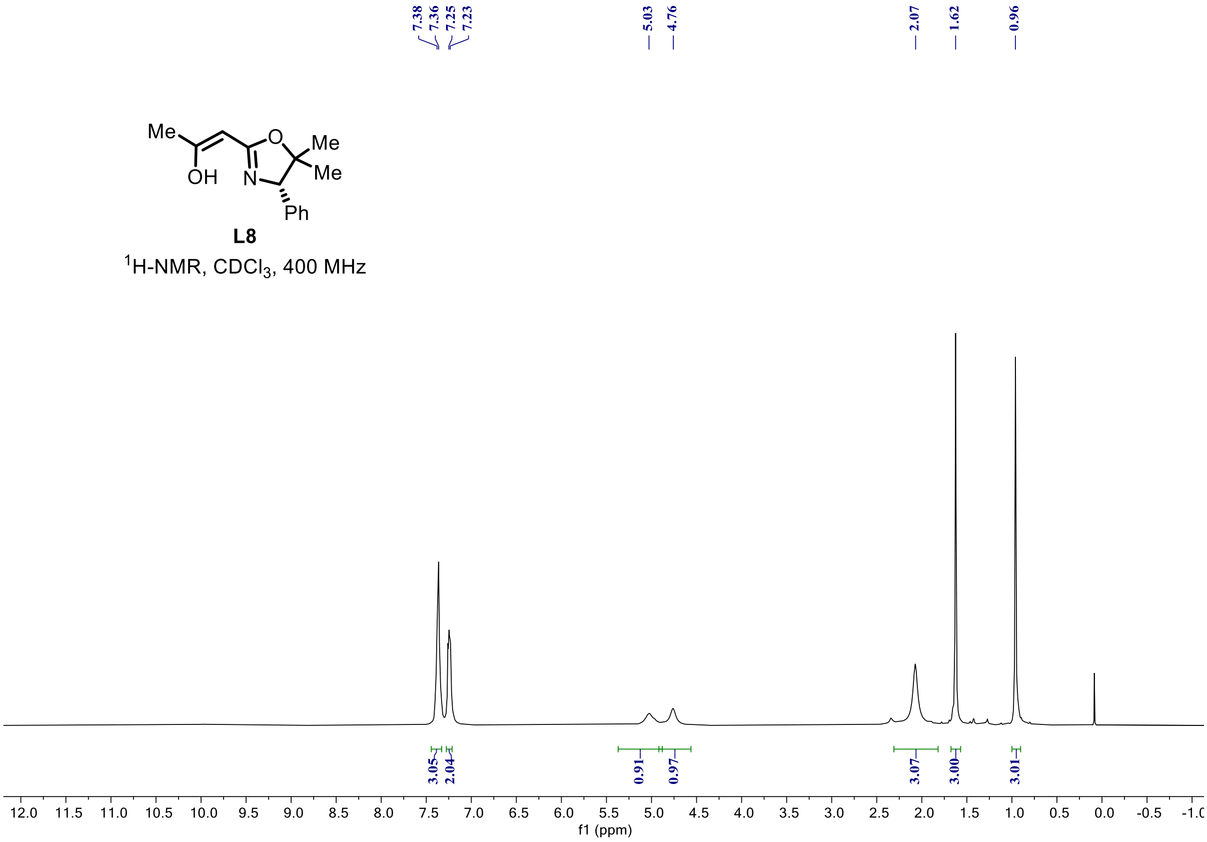

Me
OH
N
O
Me
Me
Ph
L8
$^{1}$H-NMR, CDCl$_3$, 400 MHz


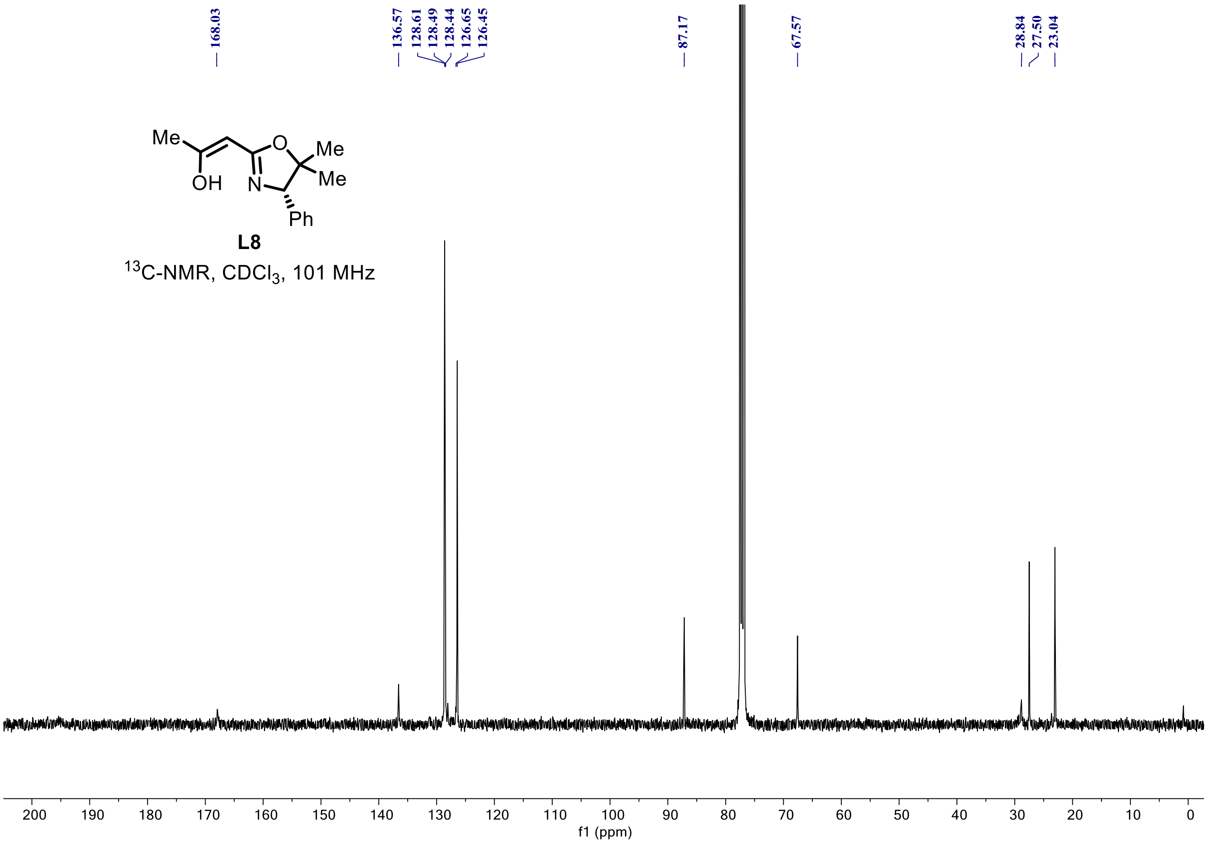

Me
OH
N
O
Me
Me
Ph
L8
$^{13}$C-NMR, CDCl$_3$, 101 MHz

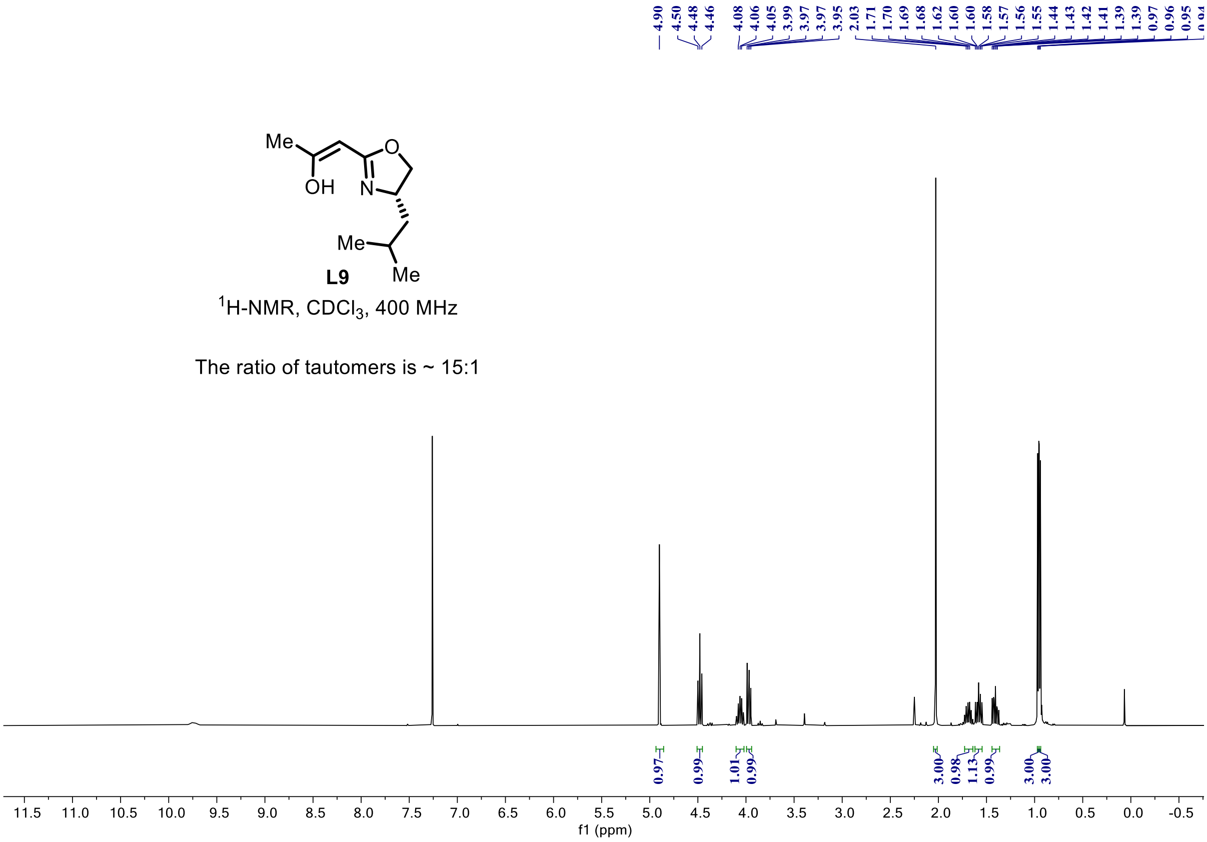
4.90 4.50 4.48 4.46 4.08 4.06 4.05 3.99 3.97 3.97 3.95 2.03 1.71 1.70 1.69 1.68 1.62 1.60 1.60 1.58 1.57 1.56 1.55 1.44 1.43 1.42 1.41 1.39 1.39 0.97 0.96 0.95
Me
OH
O
N
Me
Me
L9
1H-NMR, CDCl3, 400 MHz
The ratio of tautomers is ~ 15:1
0.97 0.99 1.01 0.99 3.00 0.98 1.13 0.99 3.00 3.00
11.5 11.0 10.5 10.0 9.5 9.0 8.5 8.0 7.5 7.0 6.5 6.0 5.5 5.0 4.5 4.0 3.5 3.0 2.5 2.0 1.5 1.0 0.5 0.0 -0.5
f1 (ppm)

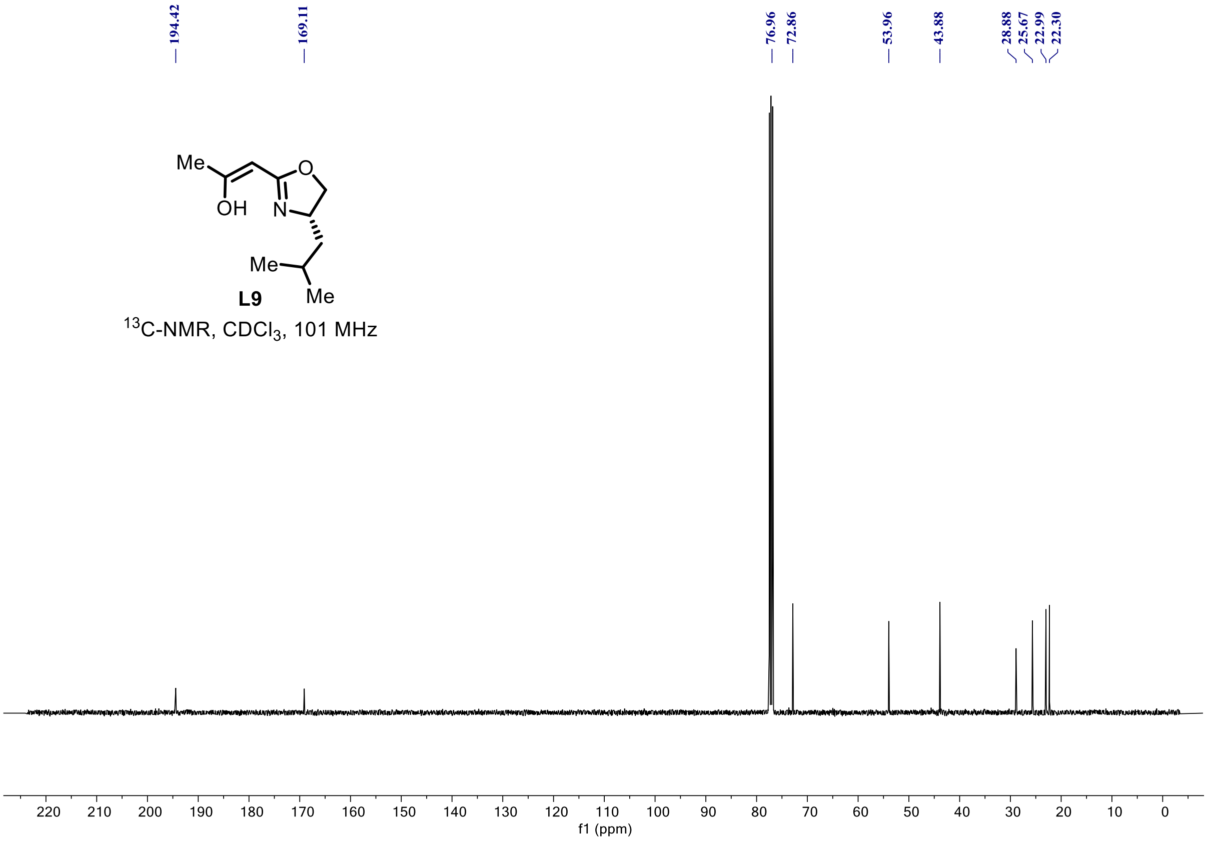
194.42 169.11 76.96 72.86 53.96 43.88 28.88 25.67 22.99 22.30
Me
OH
O
N
Me
Me
L9
13C-NMR, CDCl3, 101 MHz
220 210 200 190 180 170 160 150 140 130 120 110 100 90 80 70 60 50 40 30 20 10 0
f1 (ppm)

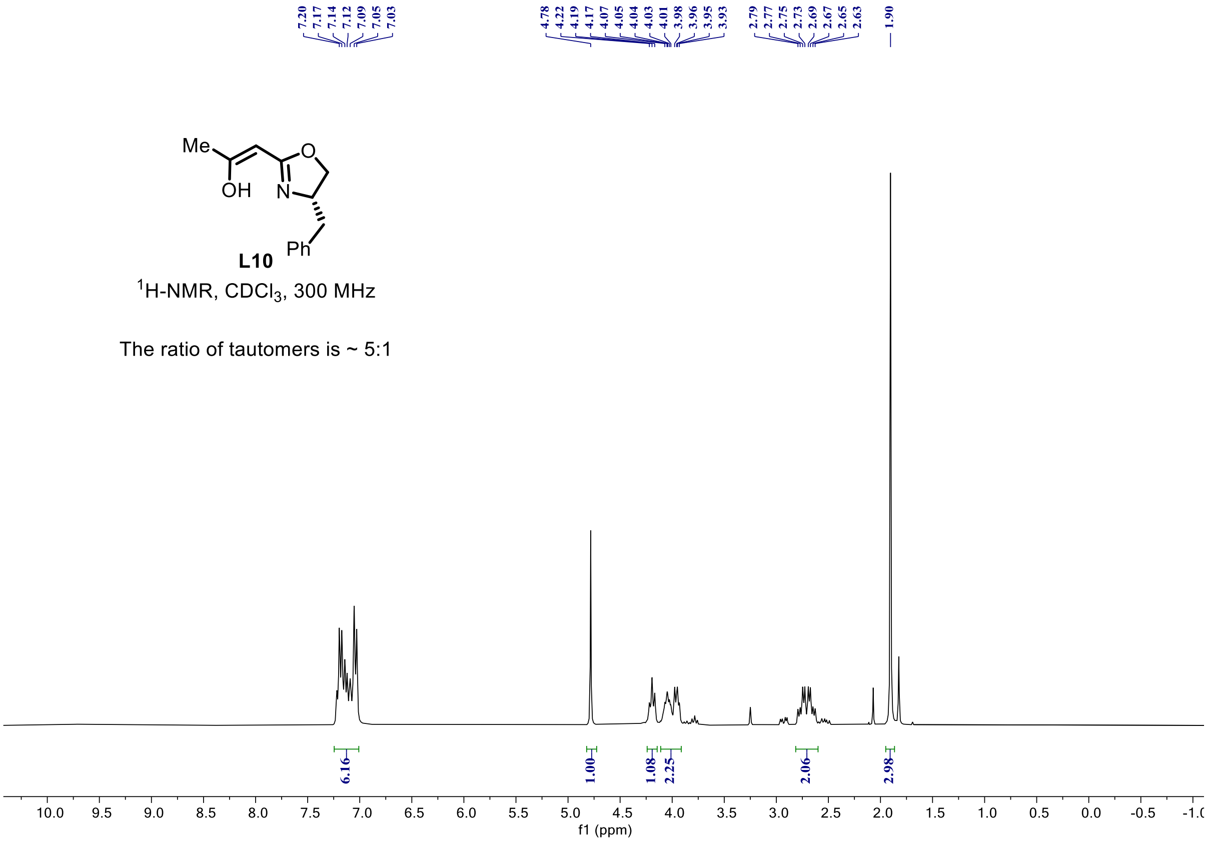
7.20
7.17
7.14
7.12
7.09
7.05
7.03
4.78
4.22
4.19
4.17
4.07
4.05
4.04
4.03
4.01
3.98
3.96
3.95
3.93
2.79
2.77
2.75
2.73
2.69
2.67
2.65
2.63
1.90
Me
OH
N
O
Ph
L10
¹H-NMR, CDCl₃, 300 MHz
The ratio of tautomers is ~ 5:1
6.16
1.00
1.08
2.25
2.06
2.98
f1 (ppm)

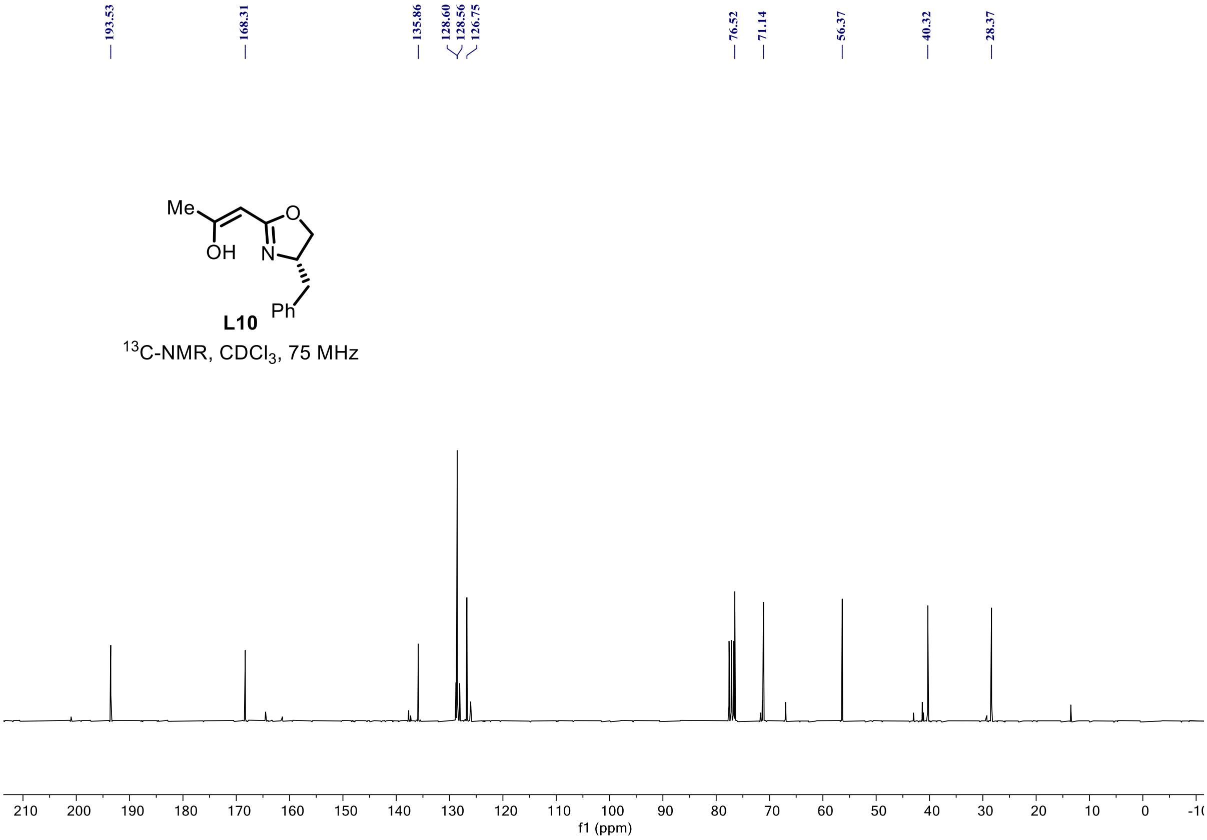
193.53
168.31
135.86
128.60
128.56
126.75
76.52
71.14
56.37
40.32
28.37
Me
OH
N
O
Ph
L10
¹³C-NMR, CDCl₃, 75 MHz
f1 (ppm)

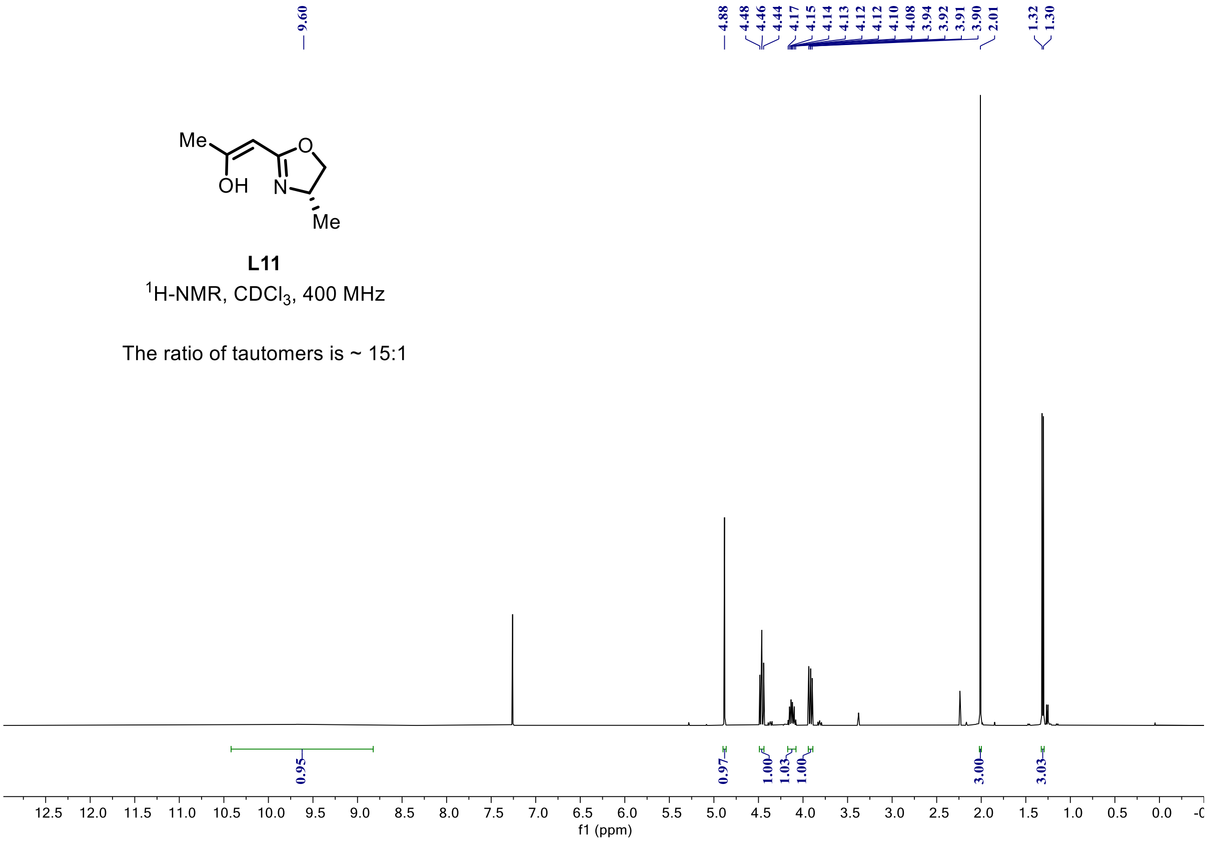
9.60
4.88 4.48 4.46 4.44 4.17 4.15 4.14 4.13 4.12 4.12 4.10 4.08 3.94 3.92 3.91 3.90 2.01
1.32 1.30
Me
OH
N
O
Me
L11
$^{1}$H-NMR, $CDCl_3$, 400 MHz
The ratio of tautomers is ~ 15:1
0.95
0.97
1.00
1.03
1.00
3.00
3.03
f1 (ppm)

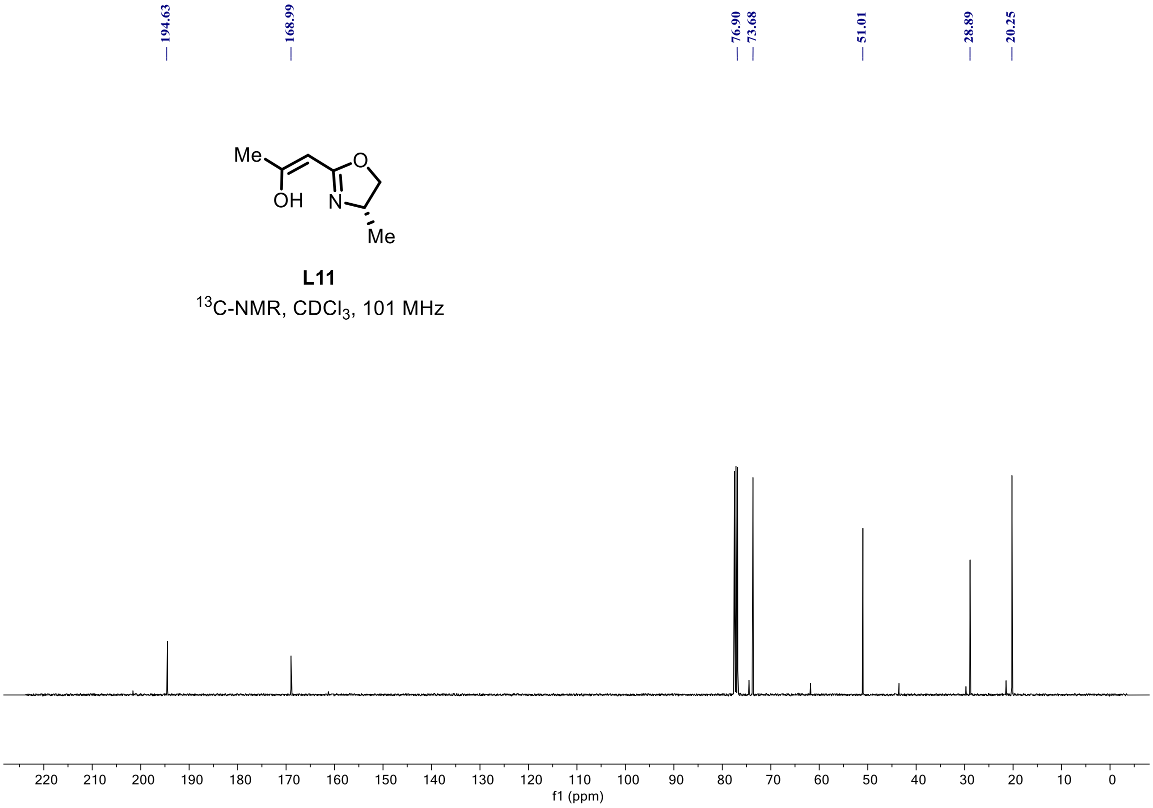
194.63
168.99
76.90
73.68
51.01
28.89
20.25
Me
OH
N
O
Me
L11
$^{13}$C-NMR, $CDCl_3$, 101 MHz
f1 (ppm)

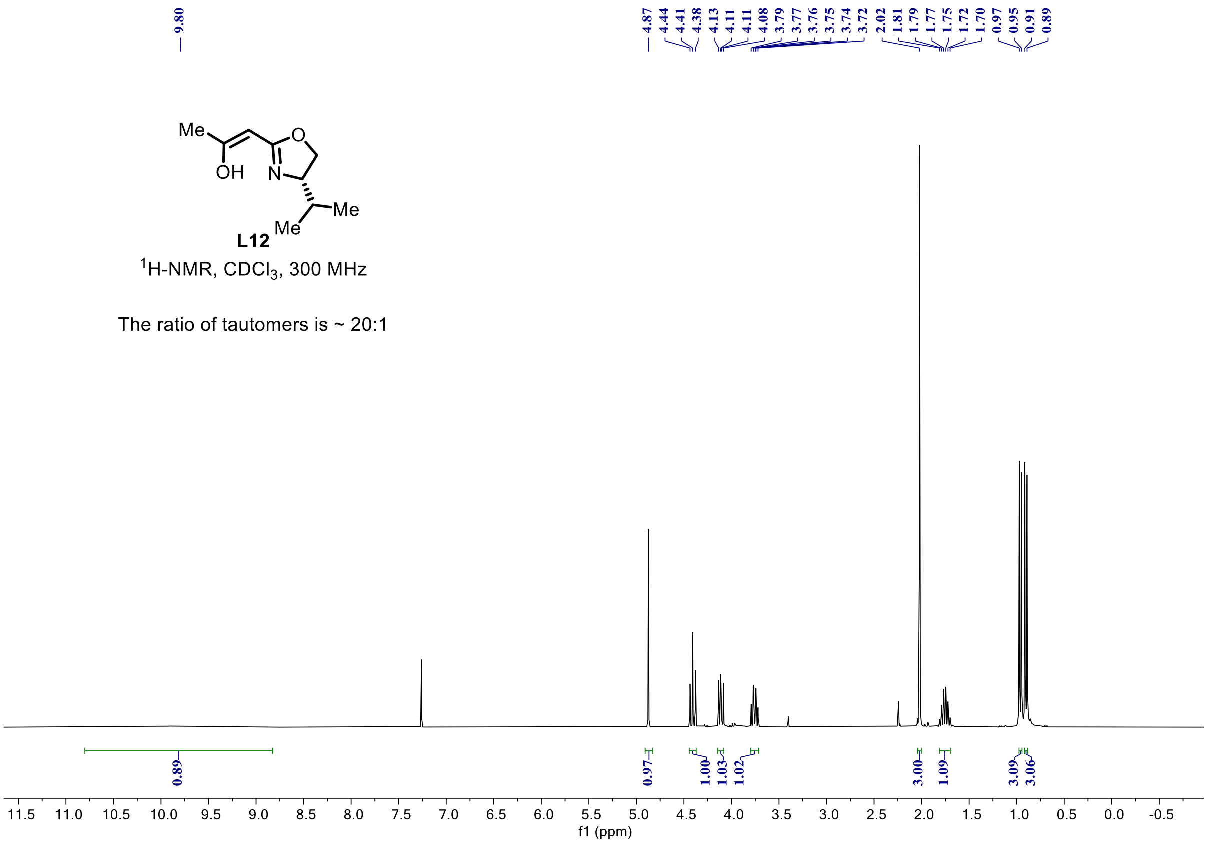
Me
OH
N
O
Me
Me
L12
$^{1}$H-NMR, CDCl$_3$, 300 MHz
The ratio of tautomers is ~ 20:1
9.80
4.87 4.44 4.41 4.38 4.13 4.11 4.11 4.08 3.79 3.77 3.76 3.75 3.74 3.72 2.02 1.81 1.79 1.77 1.75 1.72 1.70 0.97 0.95 0.91 0.89
0.89
0.97 1.00 1.03 1.02 3.00 1.09 3.09 3.06
f1 (ppm)

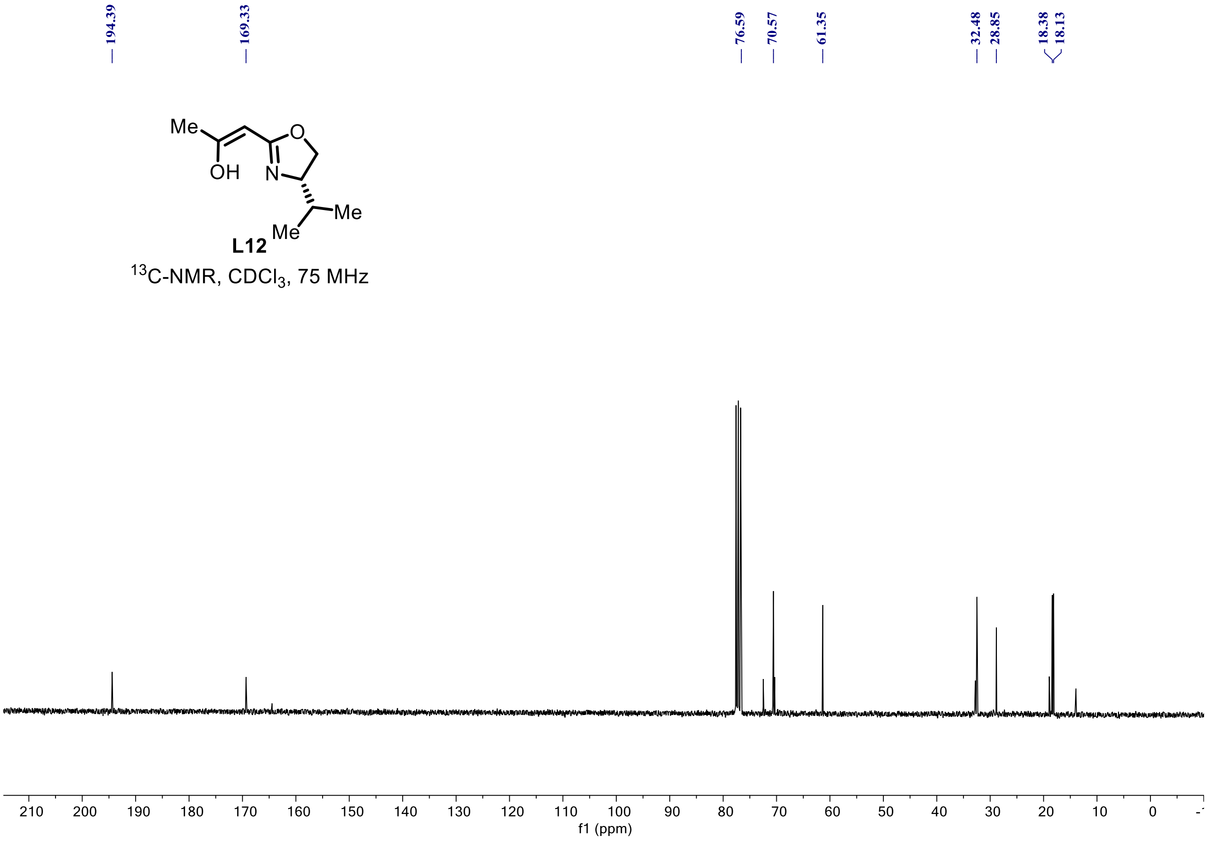
194.39
169.33
76.59
70.57
61.35
32.48
28.85
18.38
18.13
Me
OH
N
O
Me
Me
L12
$^{13}$C-NMR, CDCl$_3$, 75 MHz
f1 (ppm)

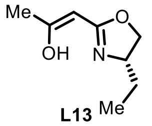


**L13**

$^1$H-NMR, $CDCl_3$, 400 MHz

The ratio of tautomers is ~ 15:

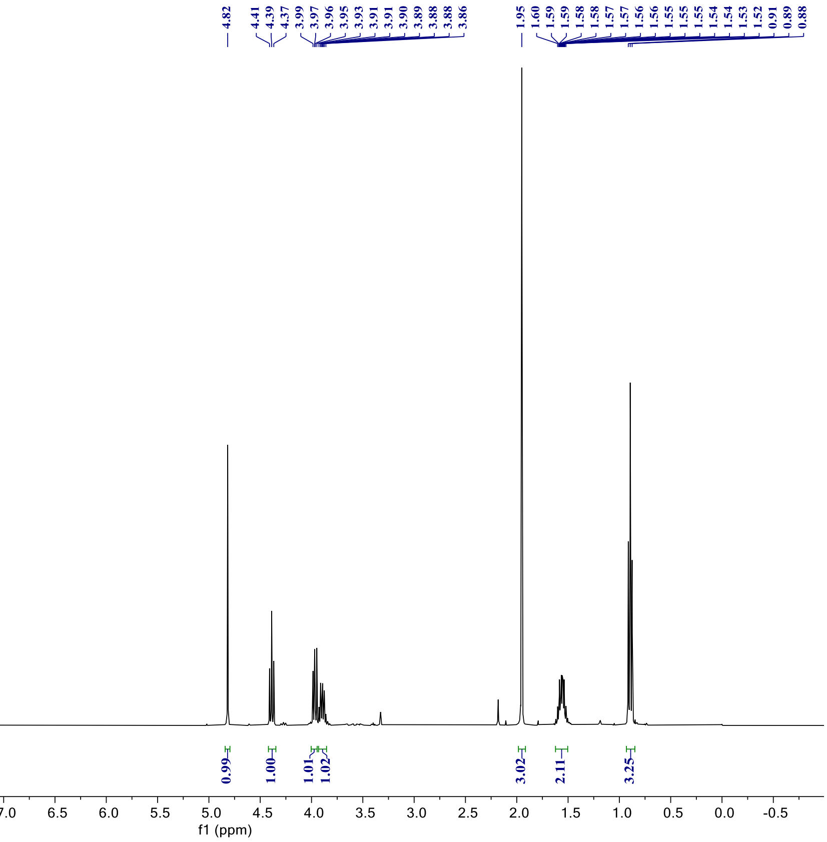


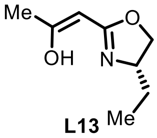


**L13**

$^{13}$C-NMR, $CDCl_3$, 101 MHz

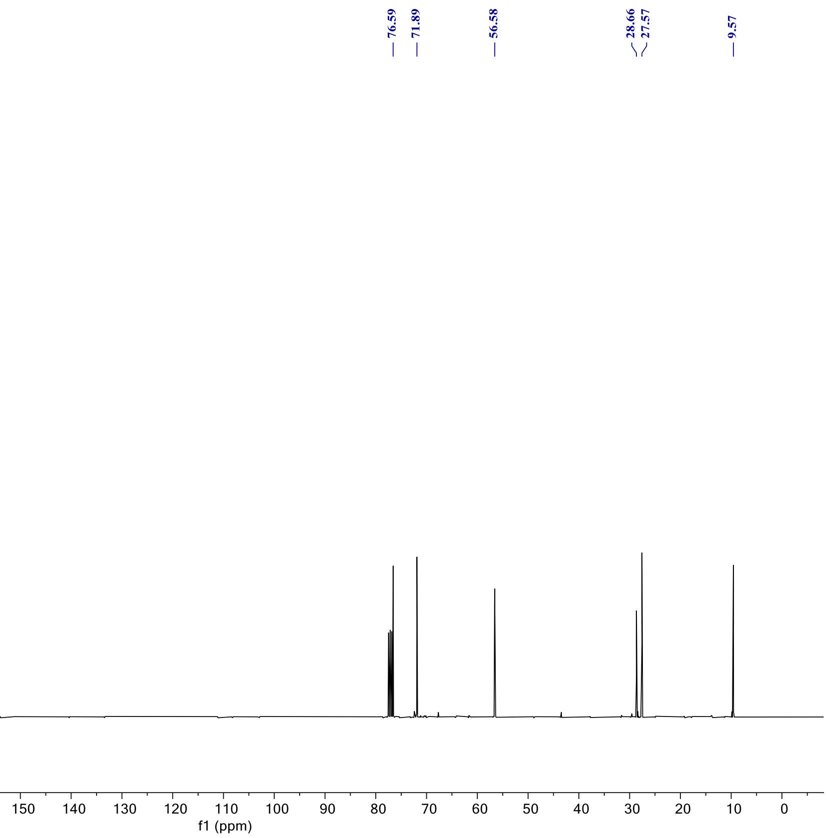

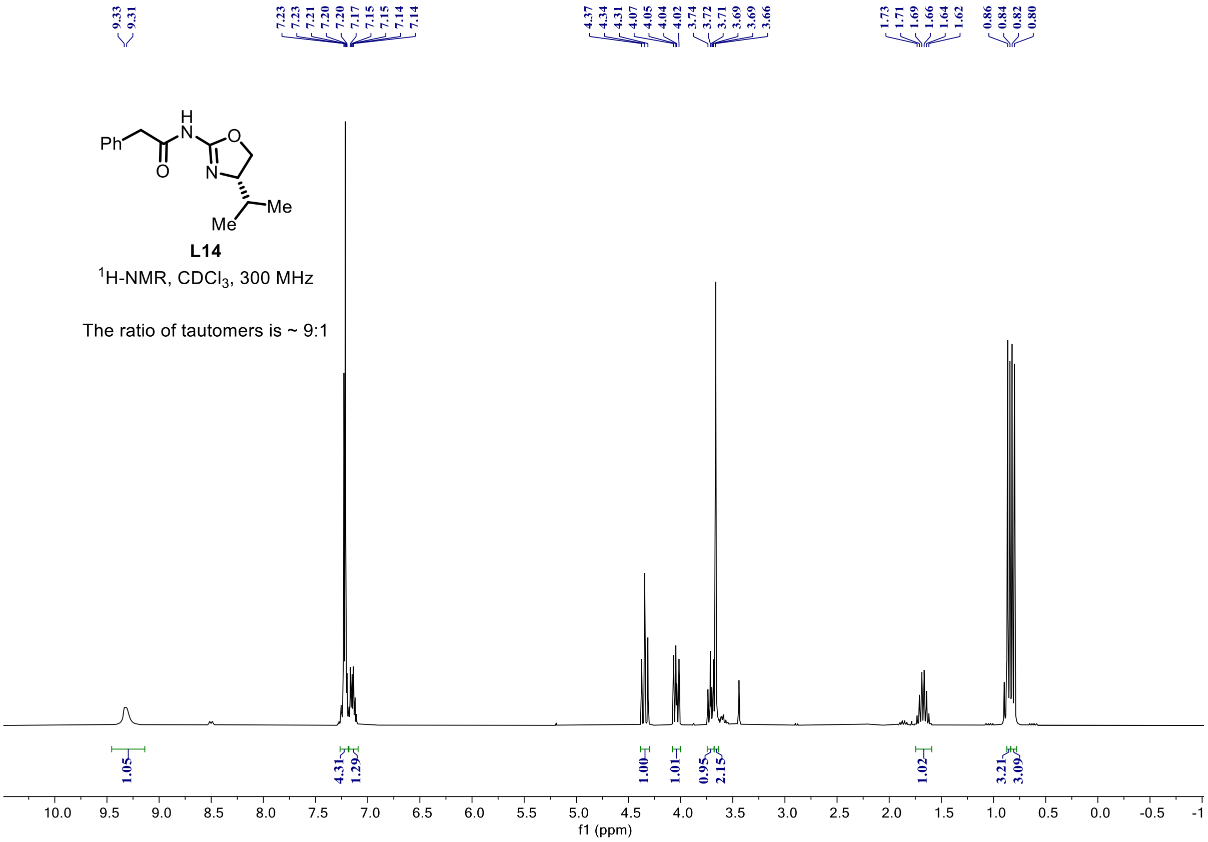
L14
$^{1}$H-NMR, CDCl$_3$, 300 MHz
The ratio of tautomers is ~ 9:1

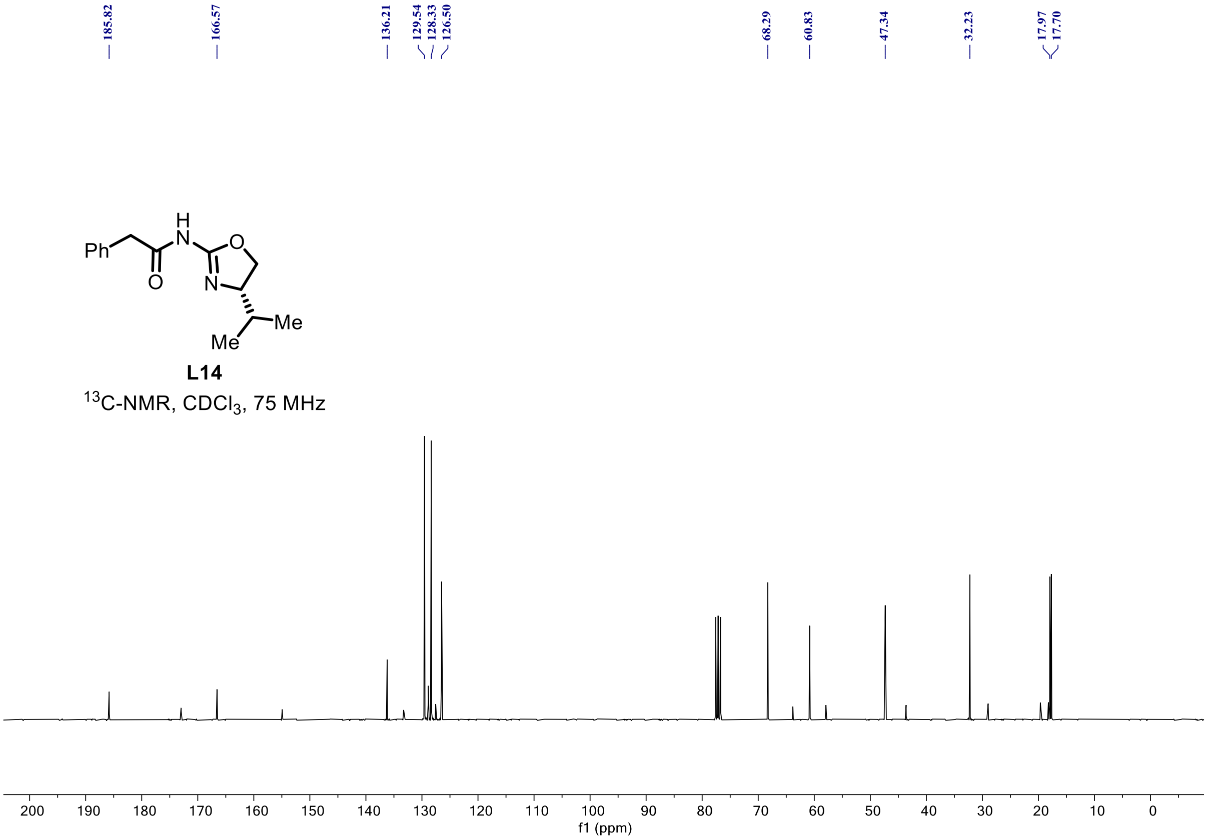
L14
$^{13}$C-NMR, CDCl$_3$, 75 MHz

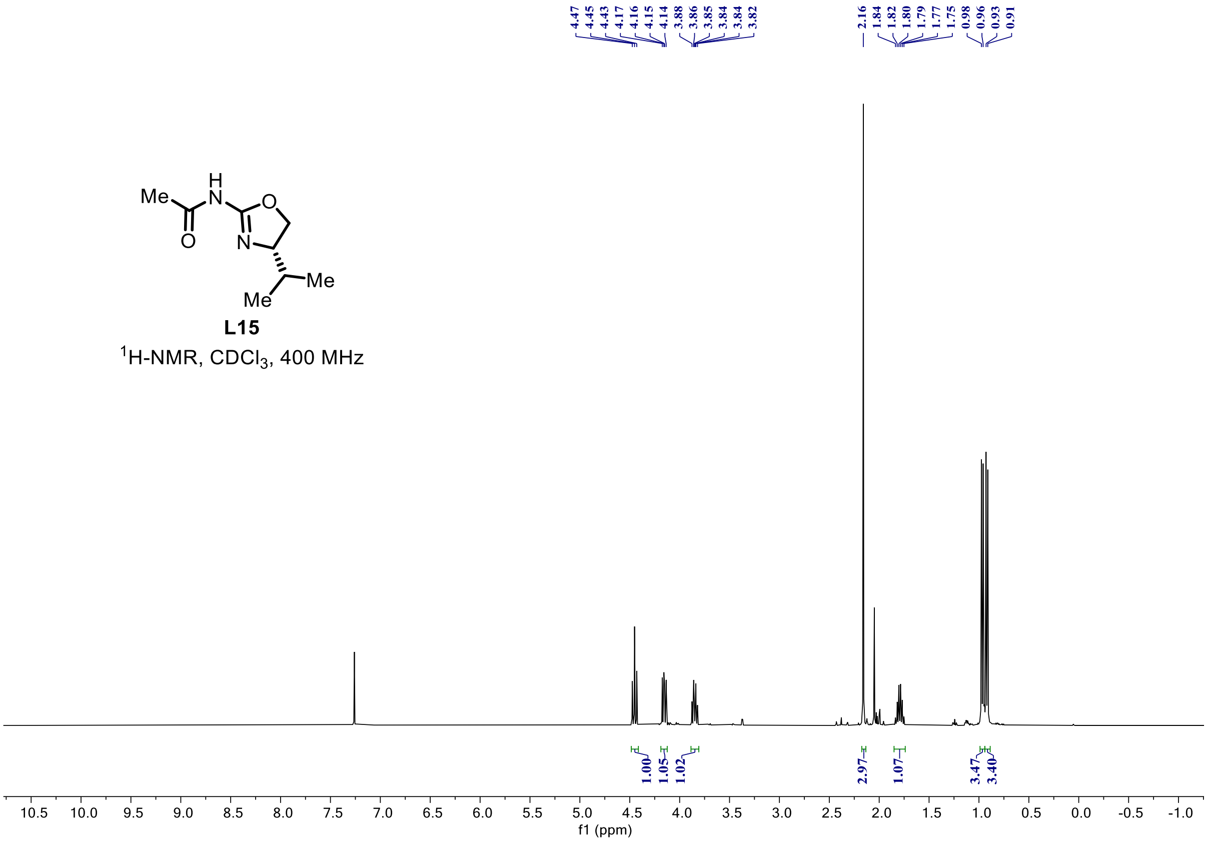
Me
H
N
O
N
Me
Me
L15
1H-NMR, CDCl3, 400 MHz
4.47 4.45 4.43 4.17 4.16 4.15 4.14 3.88 3.86 3.85 3.84 3.84 3.82
2.16 1.84 1.82 1.80 1.79 1.77 1.75 0.98 0.96 0.93 0.91
1.00 1.05 1.02 2.97 1.07 3.47 3.40
10.5 10.0 9.5 9.0 8.5 8.0 7.5 7.0 6.5 6.0 5.5 5.0 4.5 4.0 3.5 3.0 2.5 2.0 1.5 1.0 0.5 0.0 -0.5 -1.0
f1 (ppm)

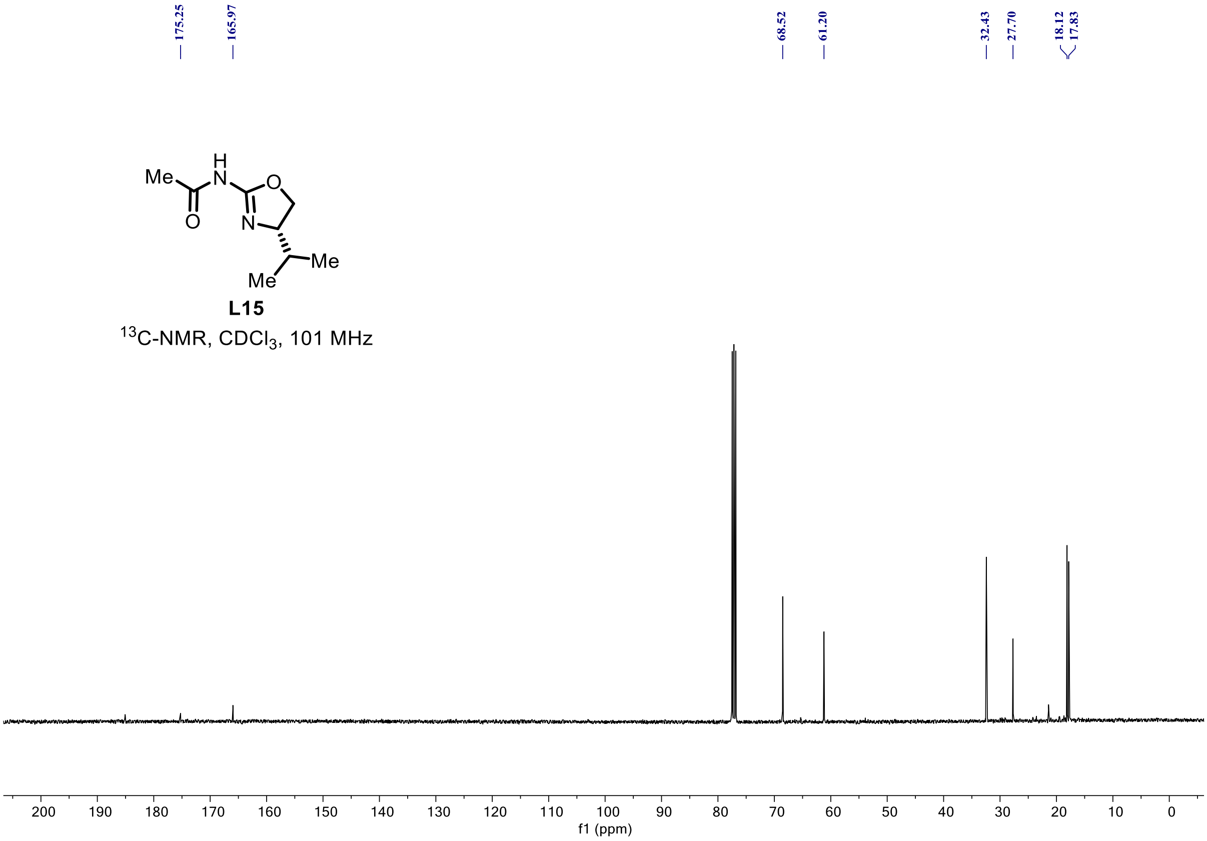
Me
H
N
O
N
Me
Me
L15
13C-NMR, CDCl3, 101 MHz
175.25
165.97
68.52
61.20
32.43
27.70
18.12 17.83
200 190 180 170 160 150 140 130 120 110 100 90 80 70 60 50 40 30 20 10 0
f1 (ppm)

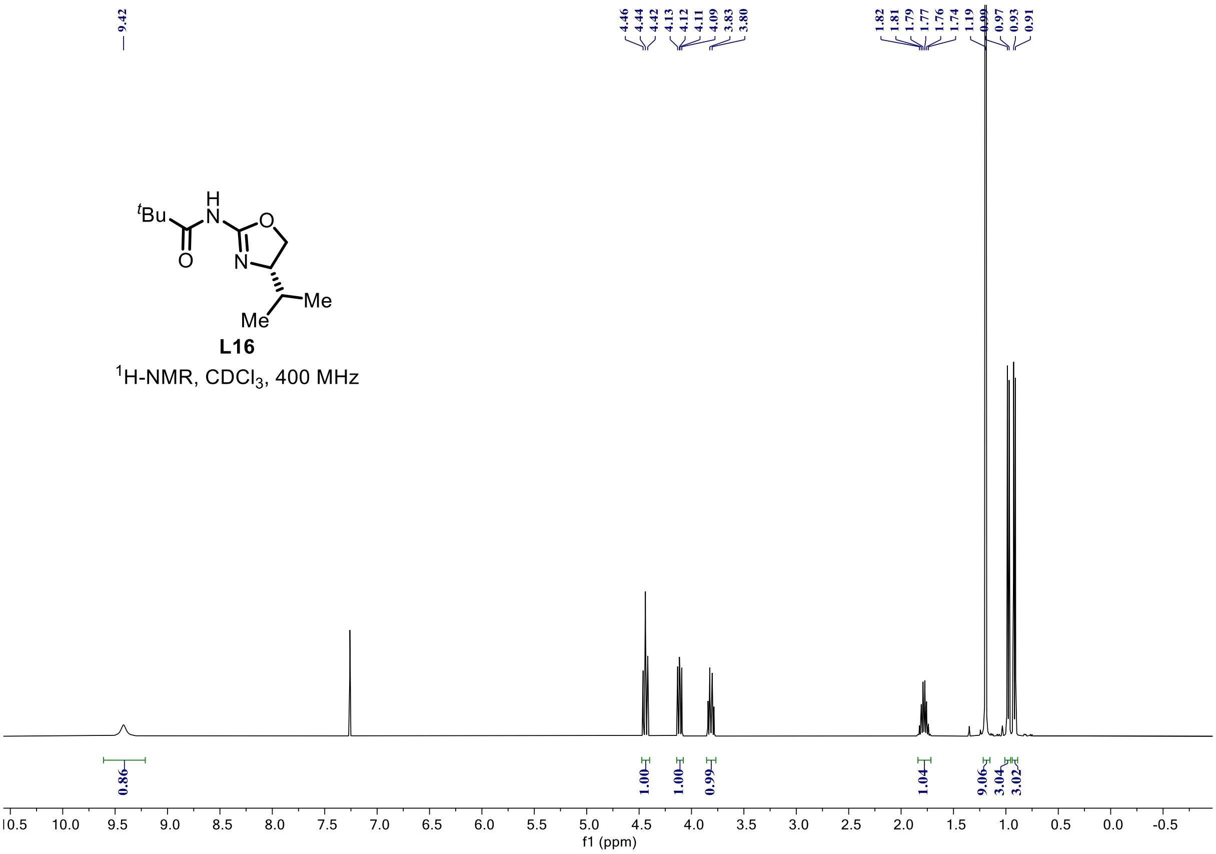
9.42
4.46 4.44 4.42 4.13 4.12 4.11 4.09 3.83 3.80
1.82 1.81 1.79 1.77 1.76 1.74 1.19 0.99 0.97 0.93 0.91
tBu
H
N
O
Me
Me
L16
$^1$H-NMR, $CDCl_3$, 400 MHz
0.86
1.00
1.00
0.99
1.04
9.06
3.04
3.02
f1 (ppm)

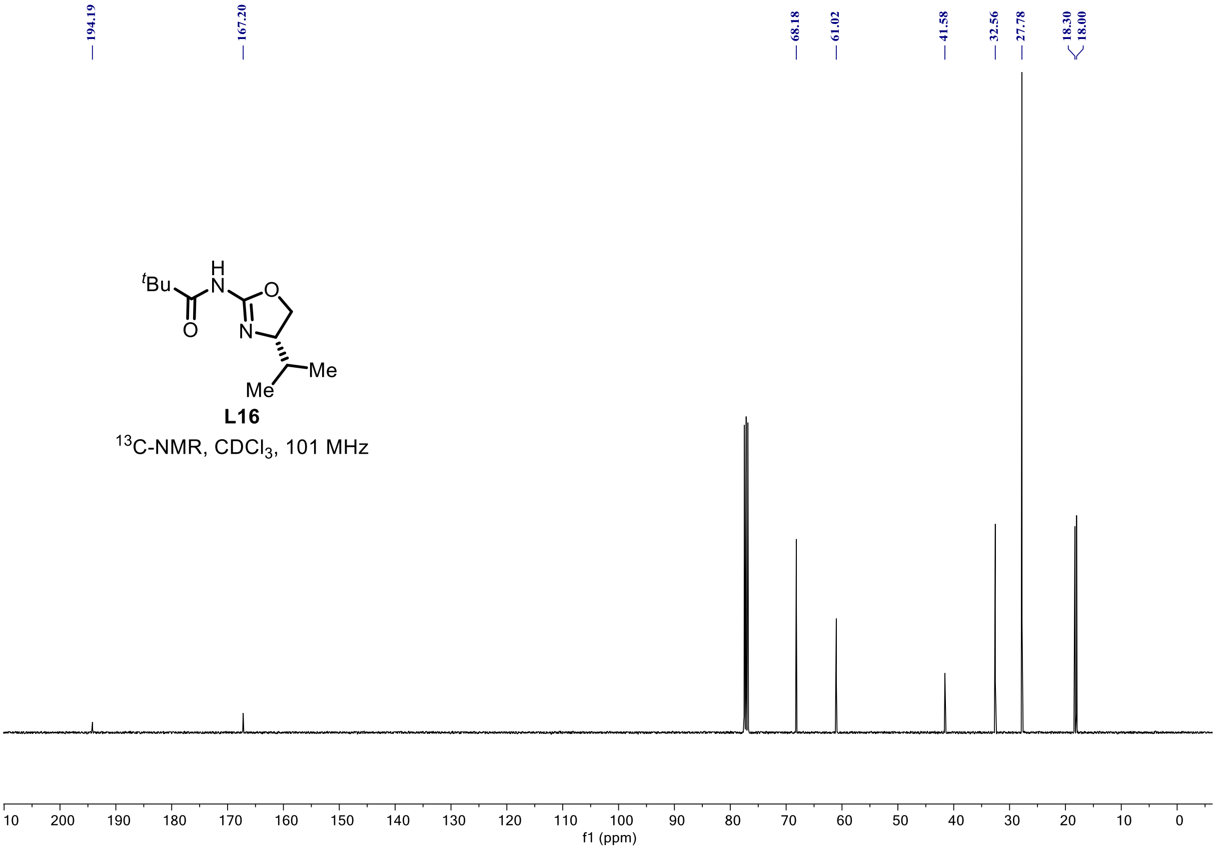
194.19
167.20
68.18
61.02
41.58
32.56
27.78
18.30
18.00
tBu
H
N
O
Me
Me
L16
$^{13}$C-NMR, $CDCl_3$, 101 MHz
f1 (ppm)

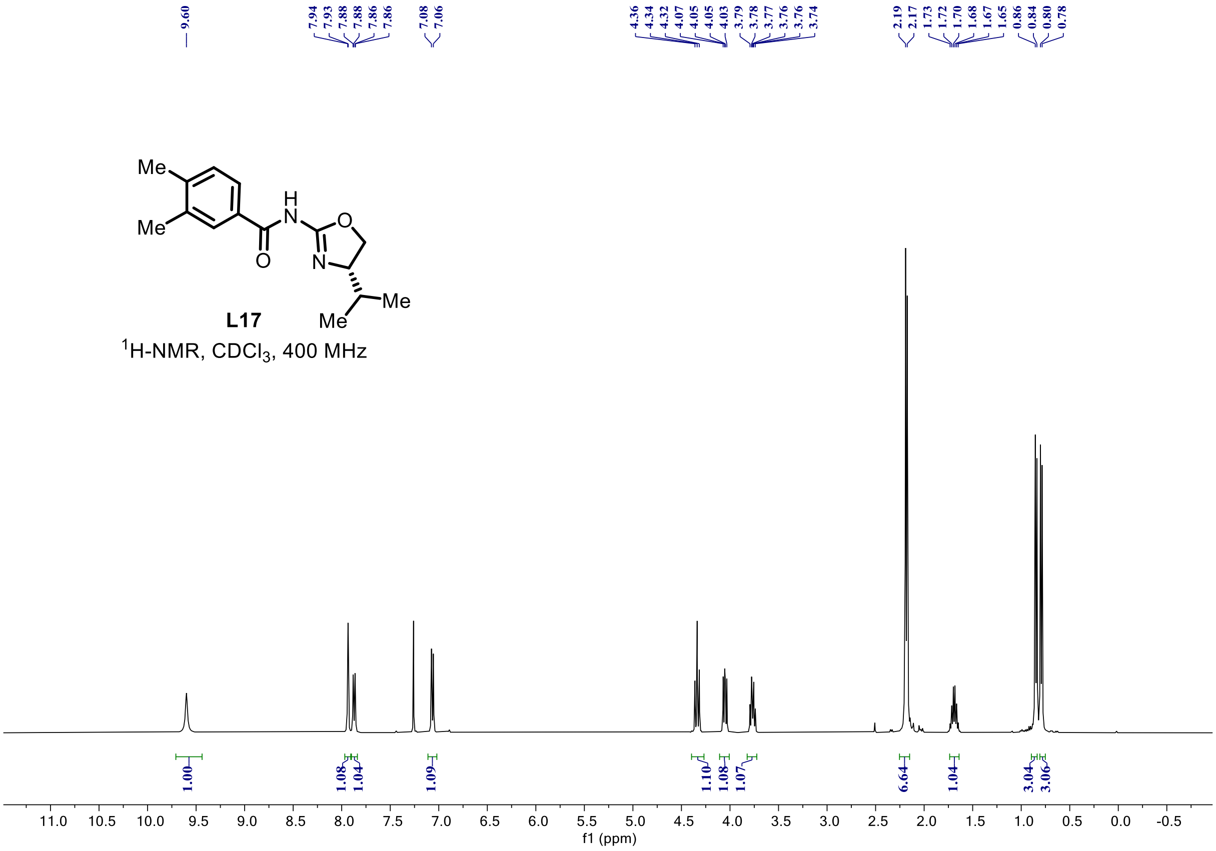
9.60
7.94 7.93 7.88 7.88 7.86 7.86 7.08 7.06
4.36 4.34 4.32 4.07 4.05 4.05 4.03 3.79 3.78 3.77 3.76 3.76 3.74
2.19 2.17 1.73 1.72 1.70 1.68 1.67 1.65 0.86 0.84 0.80 0.78
Me
Me
H
N
O
O
N
Me
Me
L17
1H-NMR, CDCl3, 400 MHz
1.00
1.08
1.04
1.09
1.10
1.08
1.07
6.64
1.04
3.04
3.06
f1 (ppm)

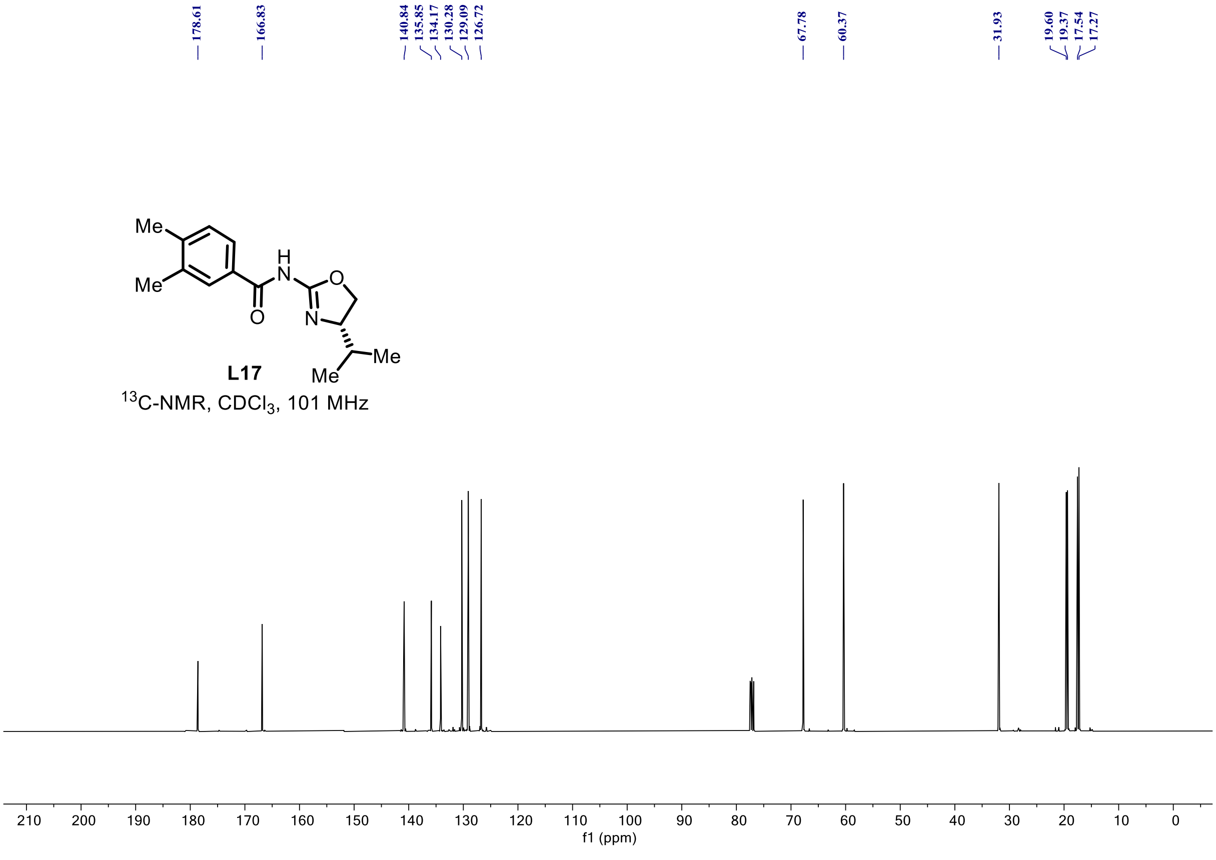
178.61
166.83
140.84 135.85 134.17 130.28 129.09 126.72
67.78
60.37
31.93
19.60 19.37 17.54 17.27
Me
Me
H
N
O
O
N
Me
Me
L17
13C-NMR, CDCl3, 101 MHz
f1 (ppm)

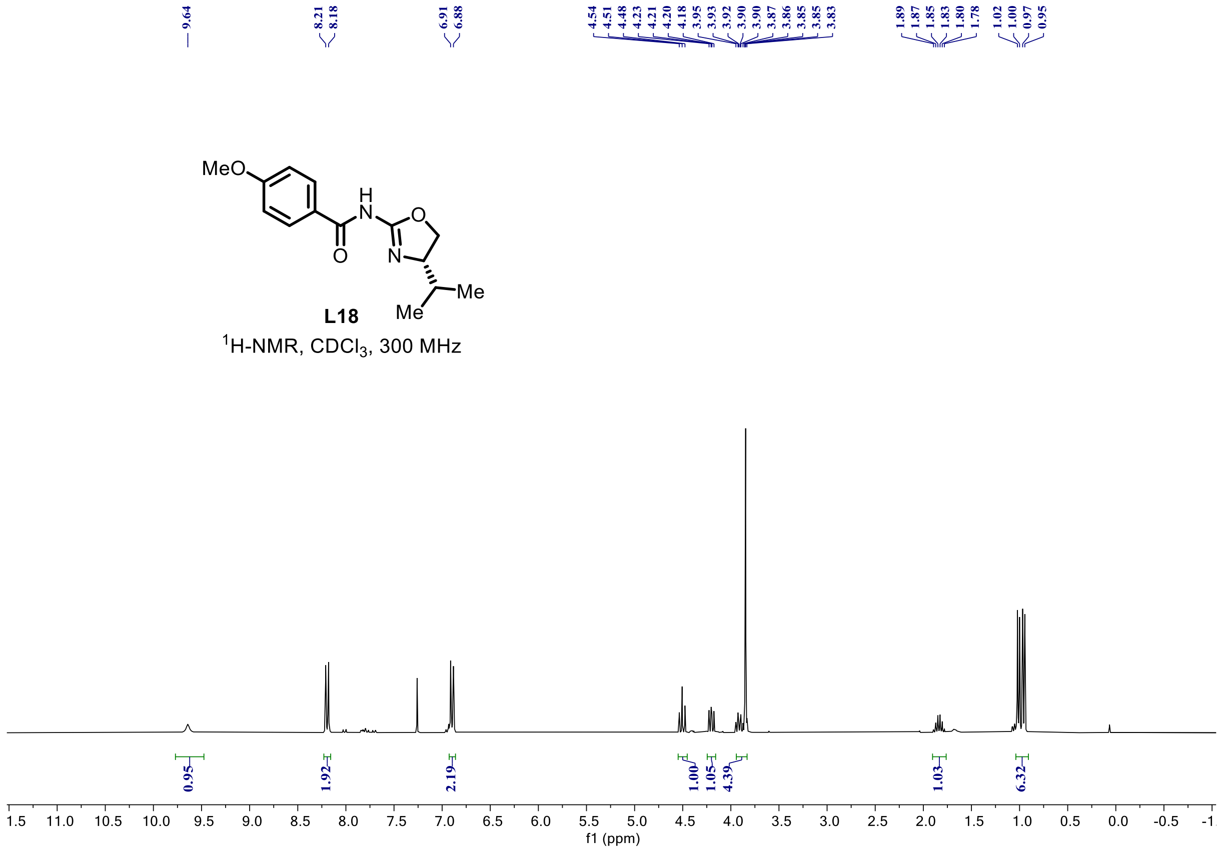
9.64
8.21
8.18
6.91
6.88
4.54
4.51
4.48
4.23
4.21
4.20
4.18
3.95
3.93
3.92
3.90
3.90
3.87
3.86
3.85
3.85
3.83
1.89
1.87
1.85
1.83
1.80
1.78
1.02
1.00
0.97
0.95
MeO
H
N
O
N
O
Me
Me
L18
$^{1}$H-NMR, $CDCl_3$, 300 MHz
0.95
1.92
2.19
1.00
1.05
4.39
1.03
6.32
f1 (ppm)

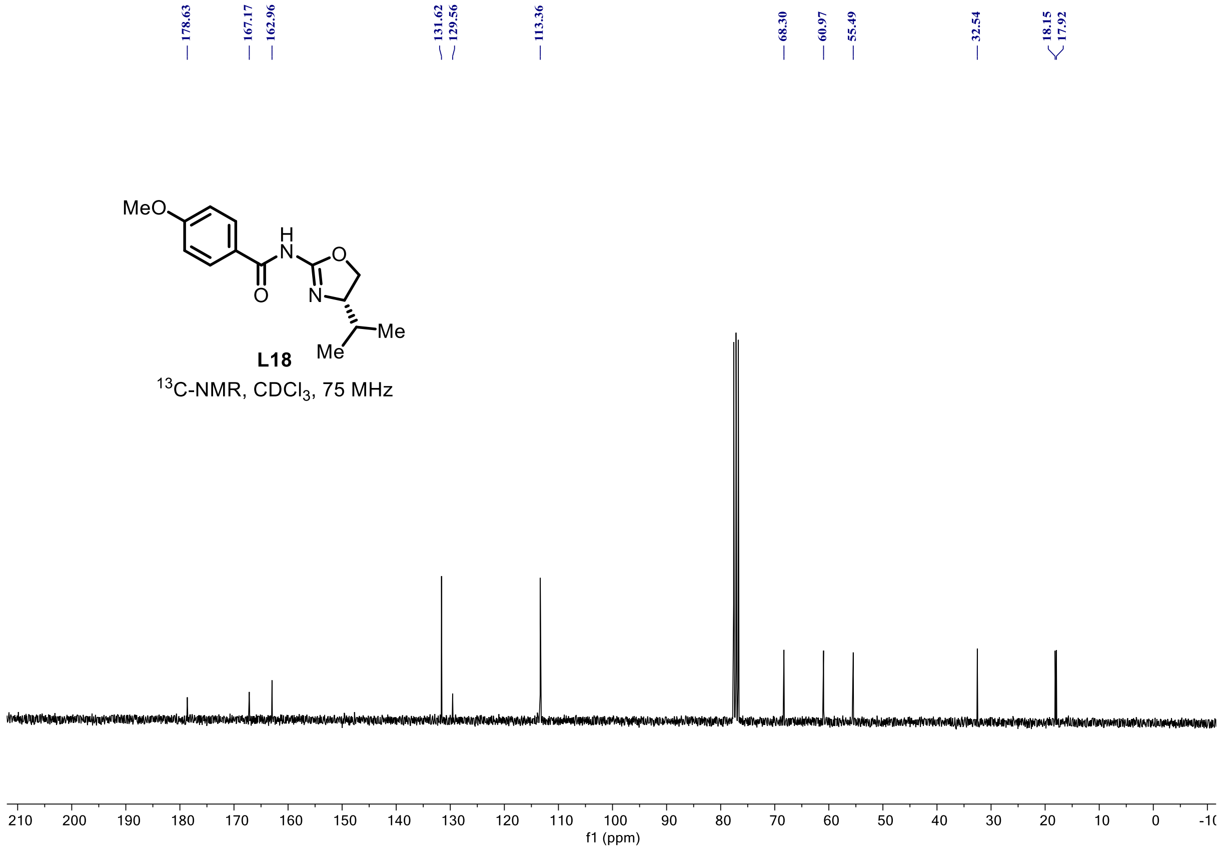
178.63
167.17
162.96
131.62
129.56
113.36
68.30
60.97
55.49
32.54
18.15
17.92
MeO
H
N
O
N
O
Me
Me
L18
$^{13}$C-NMR, $CDCl_3$, 75 MHz
f1 (ppm)

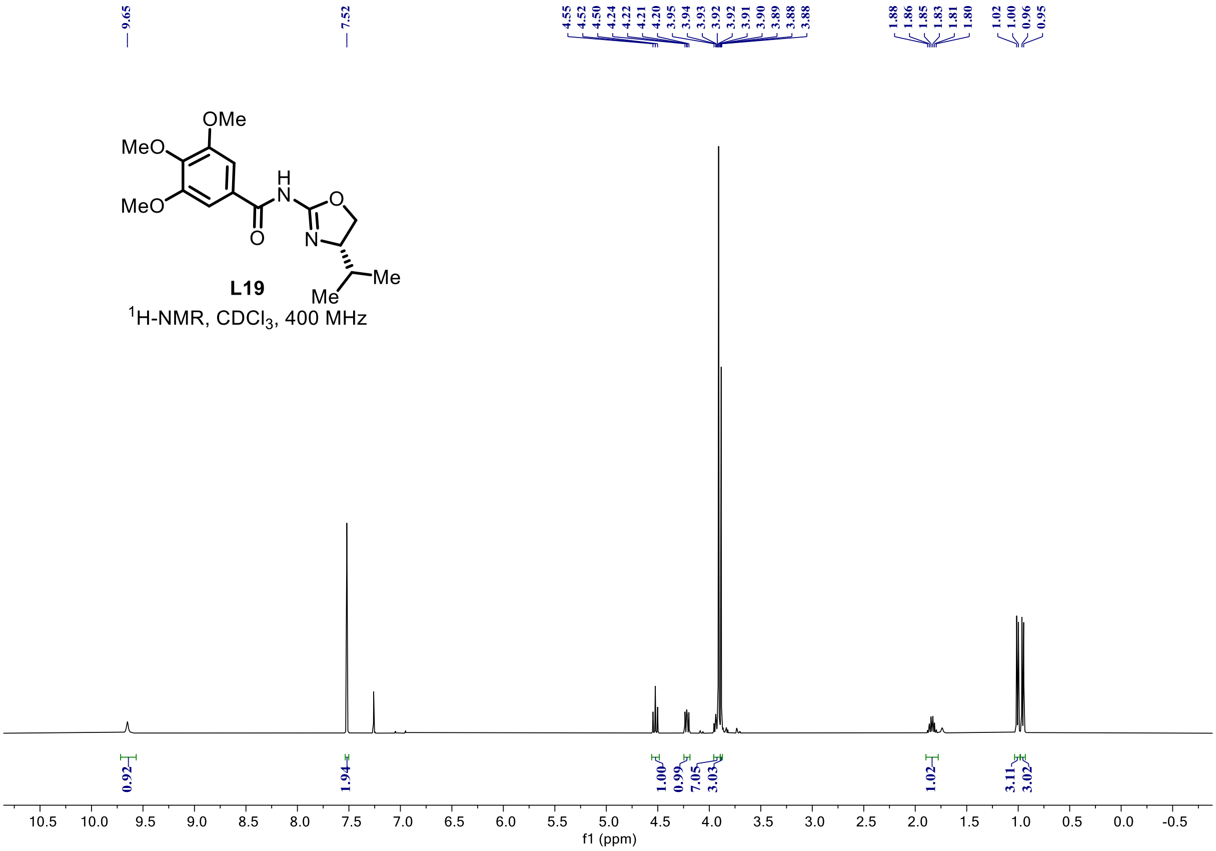
9.65
7.52
4.55 4.52 4.50 4.24 4.22 4.21 4.20 3.95 3.94 3.93 3.92 3.92 3.91 3.90 3.89 3.88 3.88
1.88 1.86 1.85 1.83 1.81 1.80
1.02 1.00 0.96 0.95
OMe
MeO
MeO
H
N
O
O
N
Me
Me
L19
1H-NMR, CDCl3, 400 MHz
0.92
1.94
1.00
0.99
7.05
3.03
1.02
3.11
3.02
f1 (ppm)

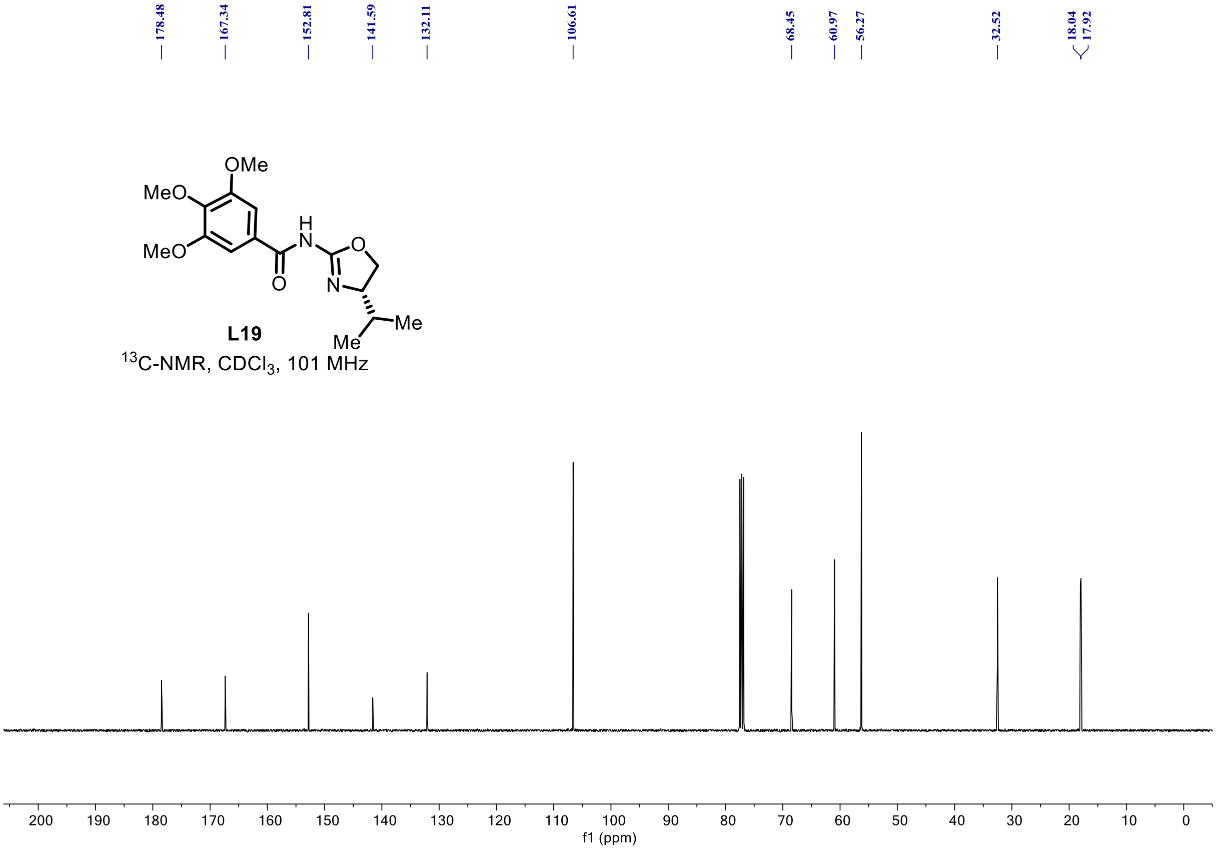
178.48
167.34
152.81
141.59
132.11
106.61
68.45
60.97
56.27
32.52
18.04
17.92
OMe
MeO
MeO
H
N
O
O
N
Me
Me
L19
13C-NMR, CDCl3, 101 MHz
f1 (ppm)

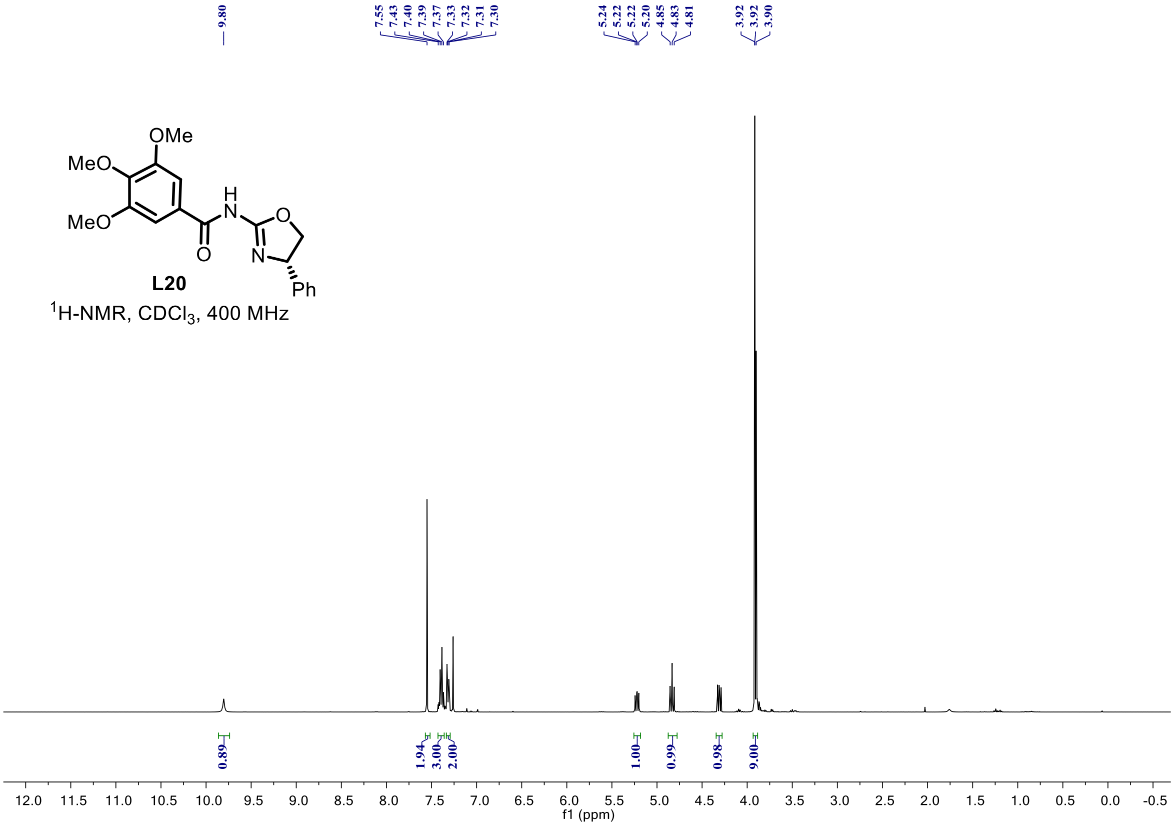
9.80
7.55 7.43 7.40 7.39 7.37 7.33 7.32 7.31 7.30
5.24 5.22 5.22 5.20 4.85 4.83 4.81
3.92 3.92 3.90
OMe
MeO
MeO
H
N
O
O
N
Ph
L20
1H-NMR, CDCl3, 400 MHz
0.89
1.94
3.00
2.00
1.00
0.99
0.98
9.00
12.0 11.5 11.0 10.5 10.0 9.5 9.0 8.5 8.0 7.5 7.0 6.5 6.0 5.5 5.0 4.5 4.0 3.5 3.0 2.5 2.0 1.5 1.0 0.5 0.0 -0.5
f1 (ppm)

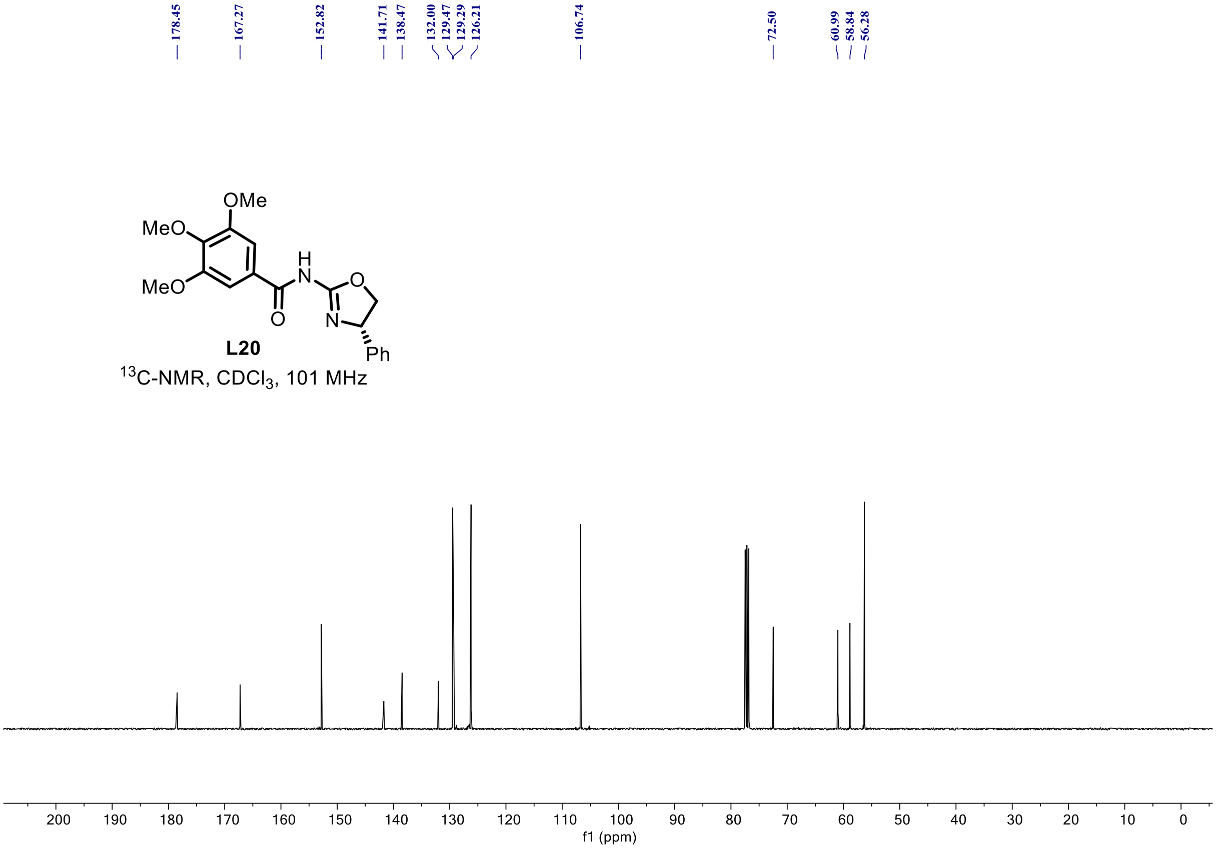
178.45
167.27
152.82
141.71
138.47
132.00
129.47
129.29
126.21
106.74
72.50
60.99
58.84
56.28
OMe
MeO
MeO
H
N
O
O
N
Ph
L20
13C-NMR, CDCl3, 101 MHz
200 190 180 170 160 150 140 130 120 110 100 90 80 70 60 50 40 30 20 10 0
f1 (ppm)

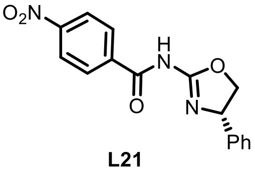

**L21**

$^{1}$H-NMR, $CDCl_3$, 400 MHz

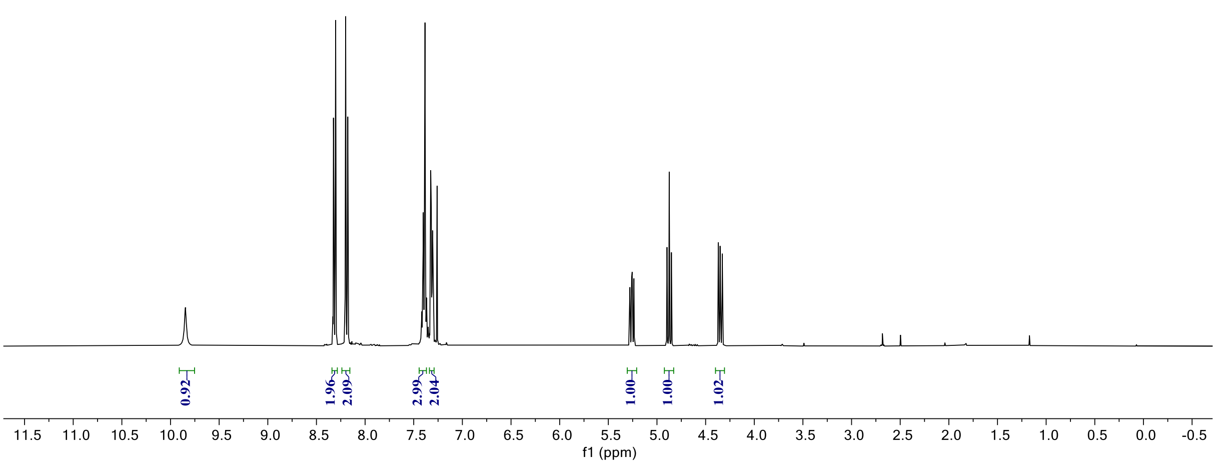


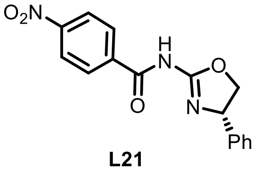

**L21**

$^{13}$C-NMR, $CDCl_3$, 101 MHz

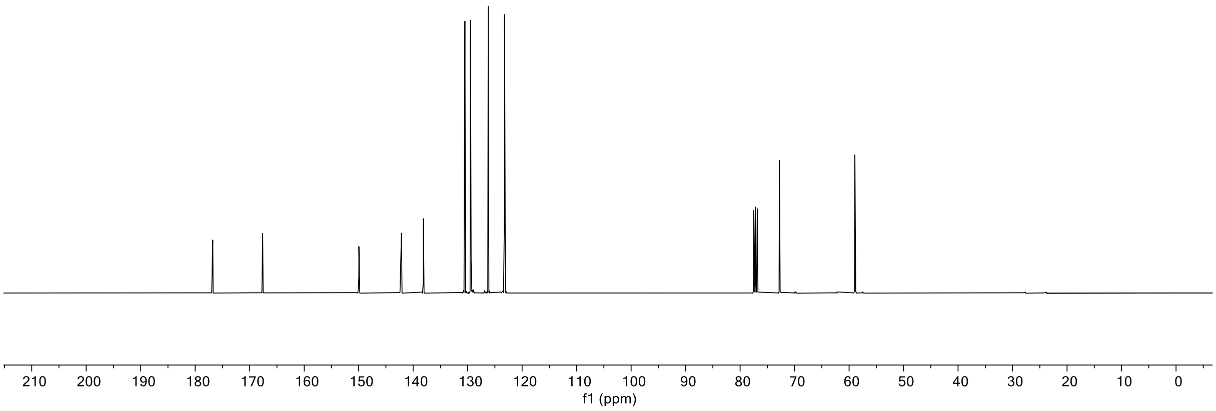

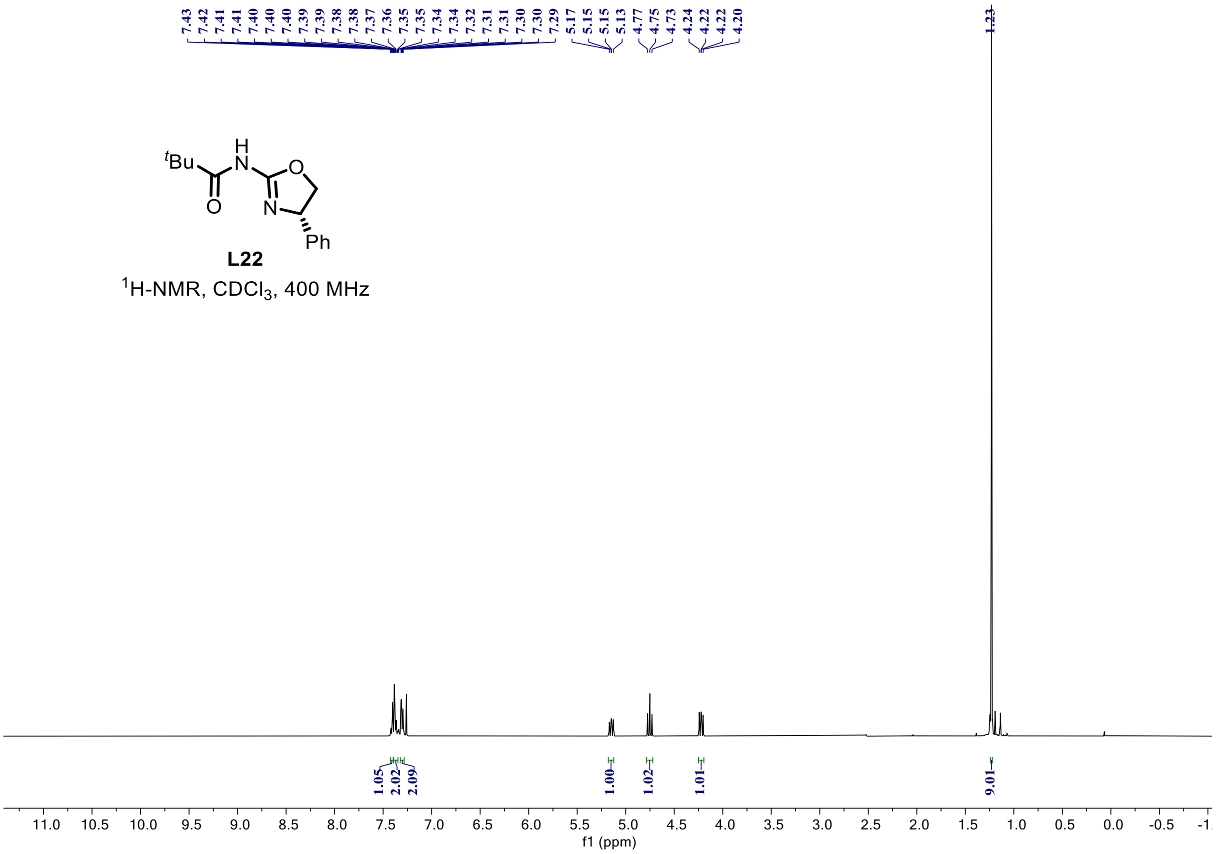
L22
$^1$H-NMR, CDCl$_3$, 400 MHz
f1 (ppm)

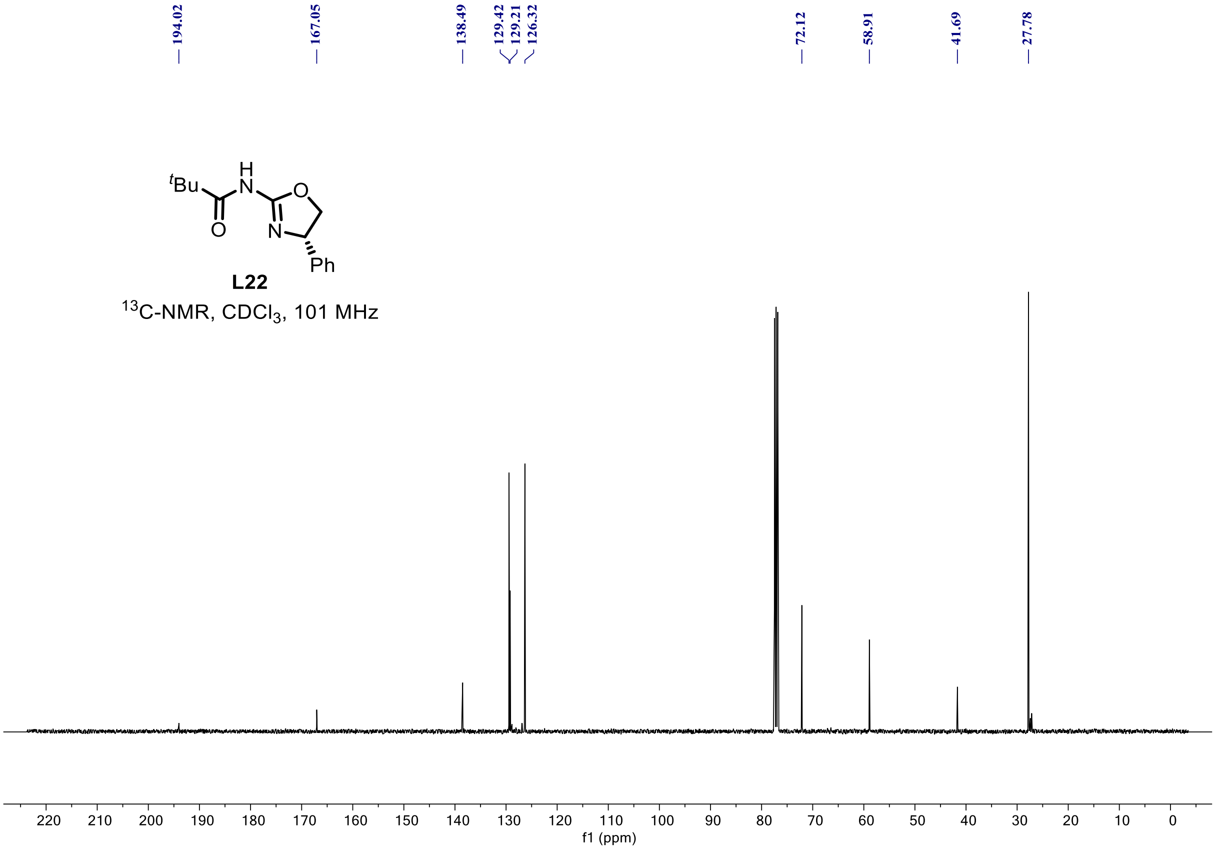
L22
$^{13}$C-NMR, CDCl$_3$, 101 MHz
f1 (ppm)

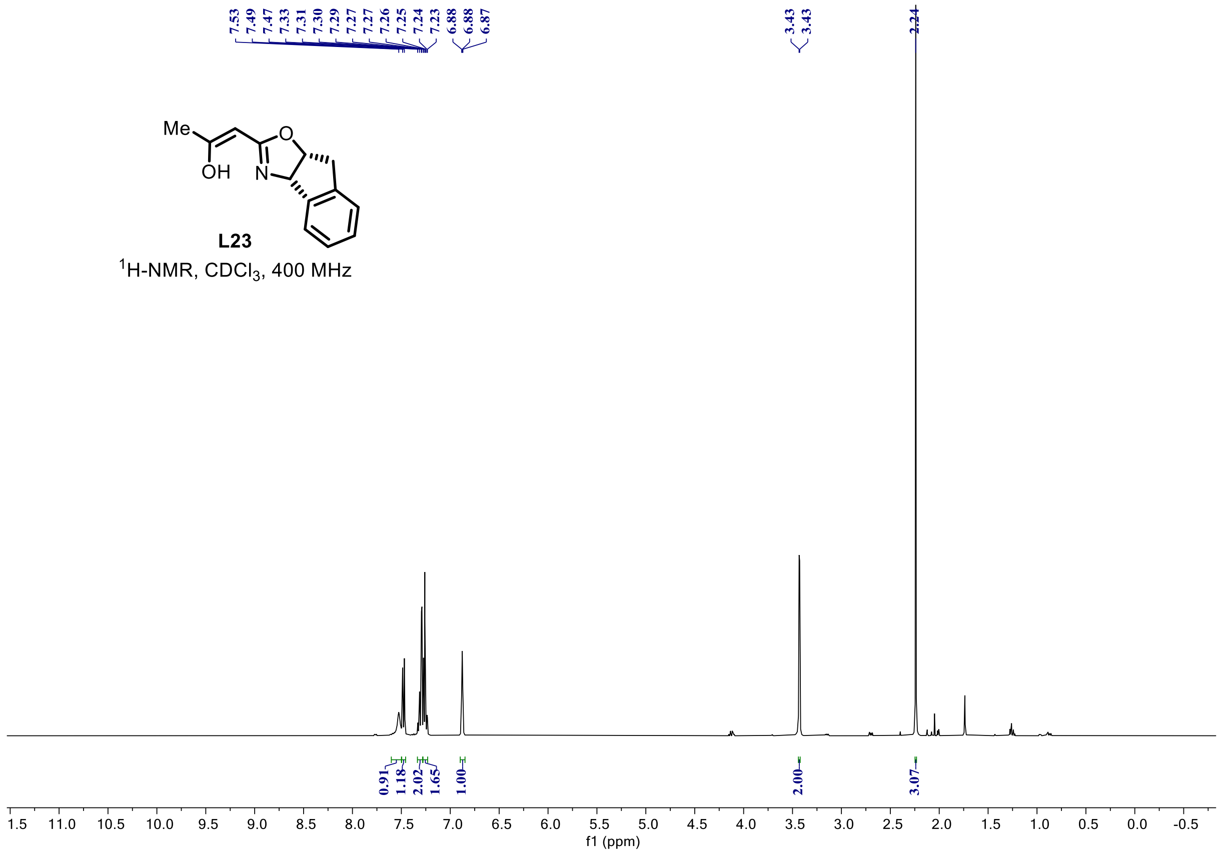
7.53 7.49 7.47 7.33 7.31 7.30 7.29 7.27 7.27 7.26 7.25 7.24 7.23 6.88 6.88 6.87
3.43 3.43
2.24
Me
OH
N
O
L23
$^{1}$H-NMR, $CDCl_3$, 400 MHz
0.91 1.18 2.02 1.65 1.00
2.00
3.07
f1 (ppm)

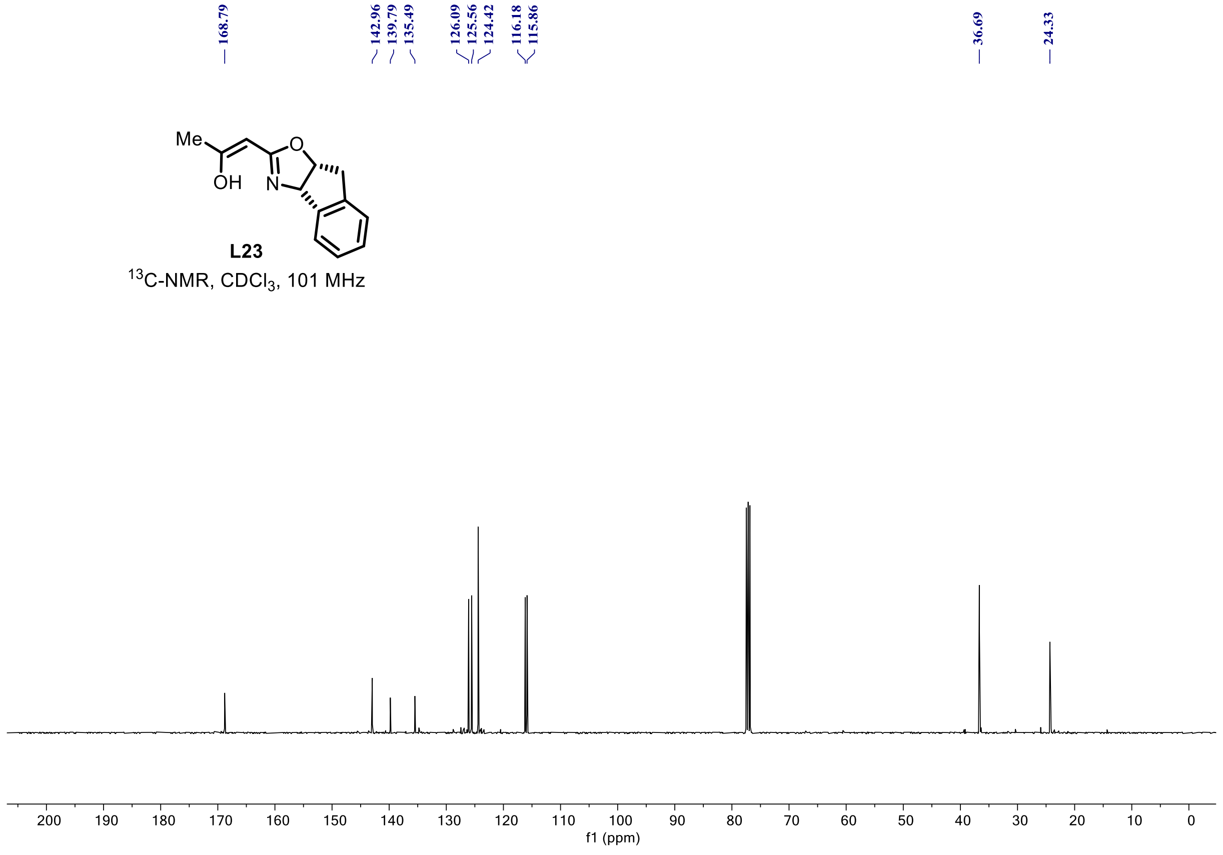
168.79
142.96 139.79 135.49
126.09 125.56 124.42
116.18 115.86
36.69
24.33
Me
OH
N
O
L23
$^{13}$C-NMR, $CDCl_3$, 101 MHz
f1 (ppm)

# NMR Characterization Data of the Synthesized Compounds

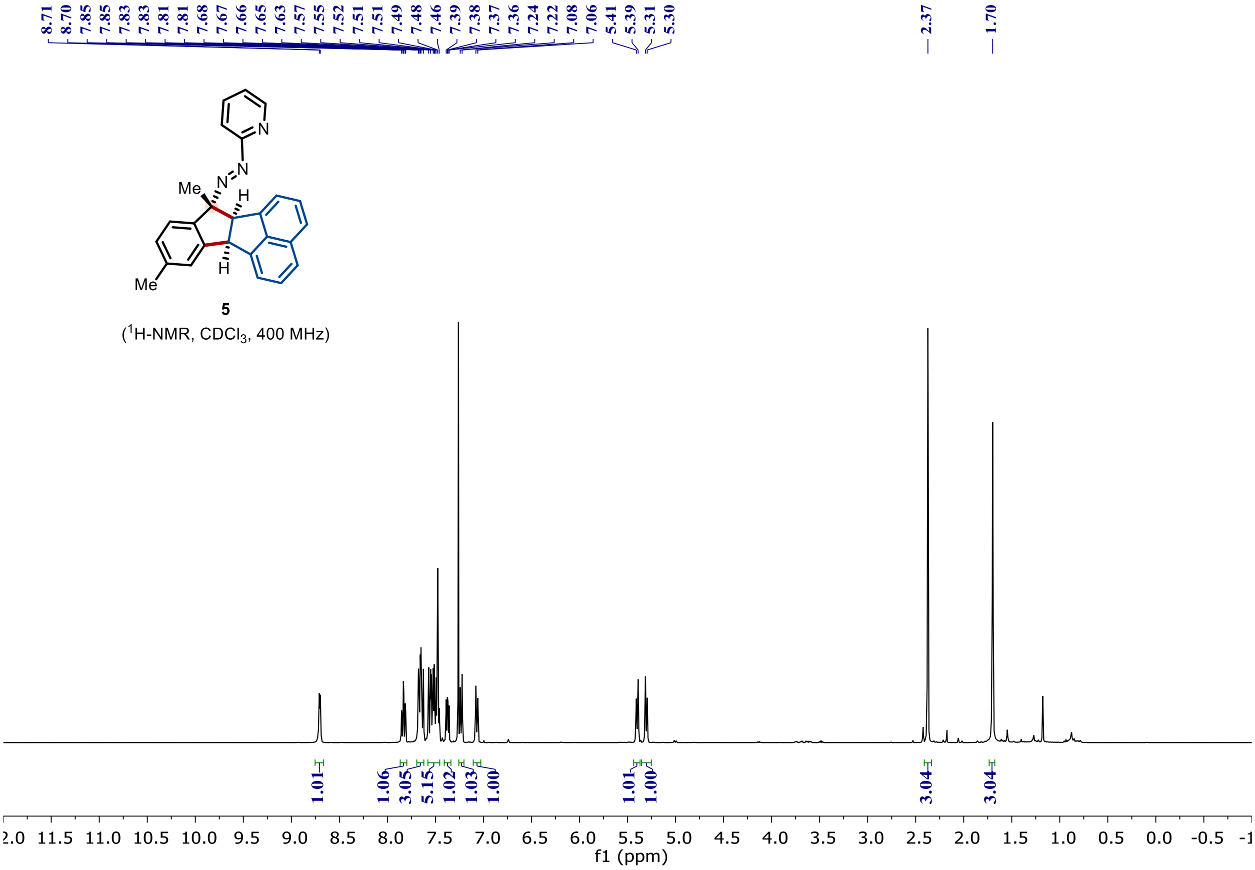


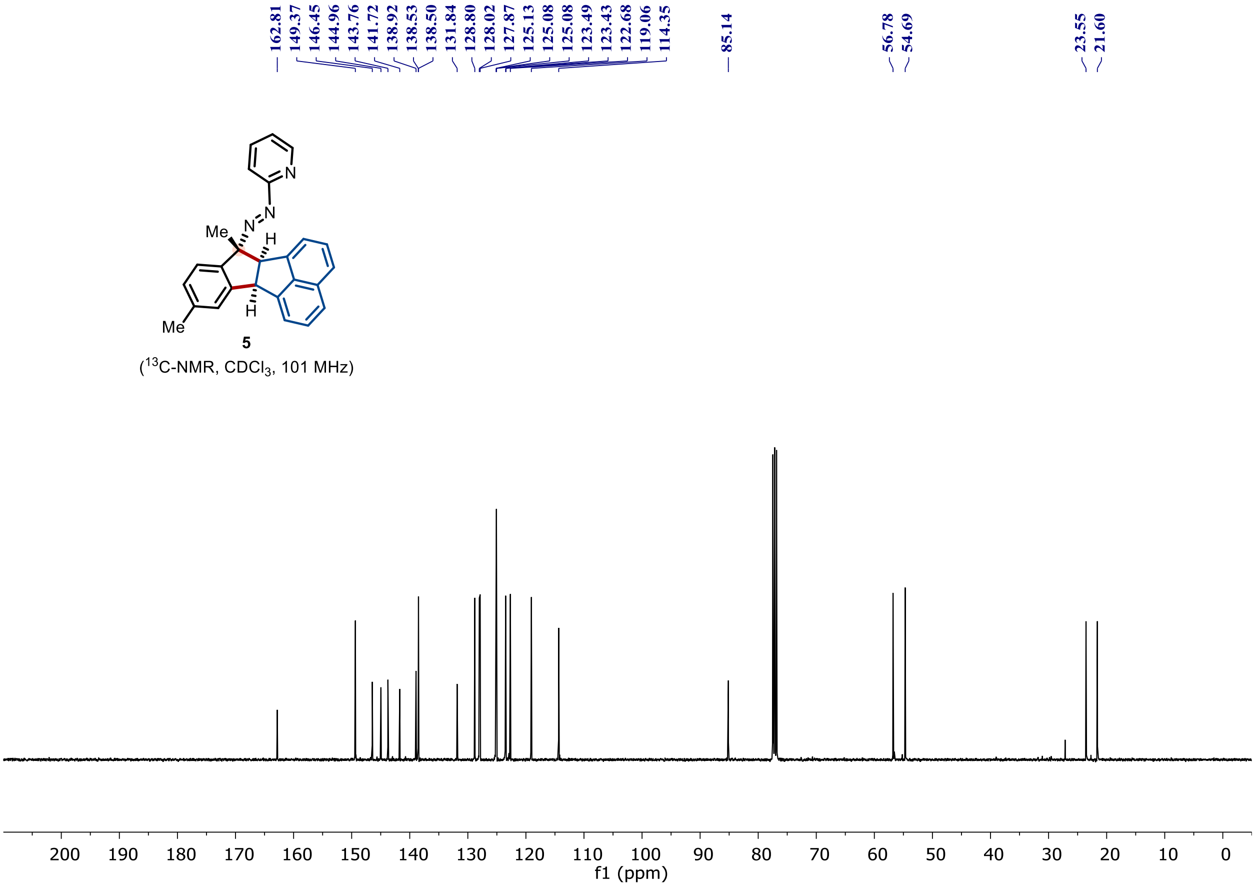

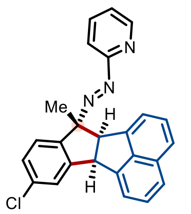


**6**
($^{1}$H-NMR, $CDCl_3$, 400 MHz)

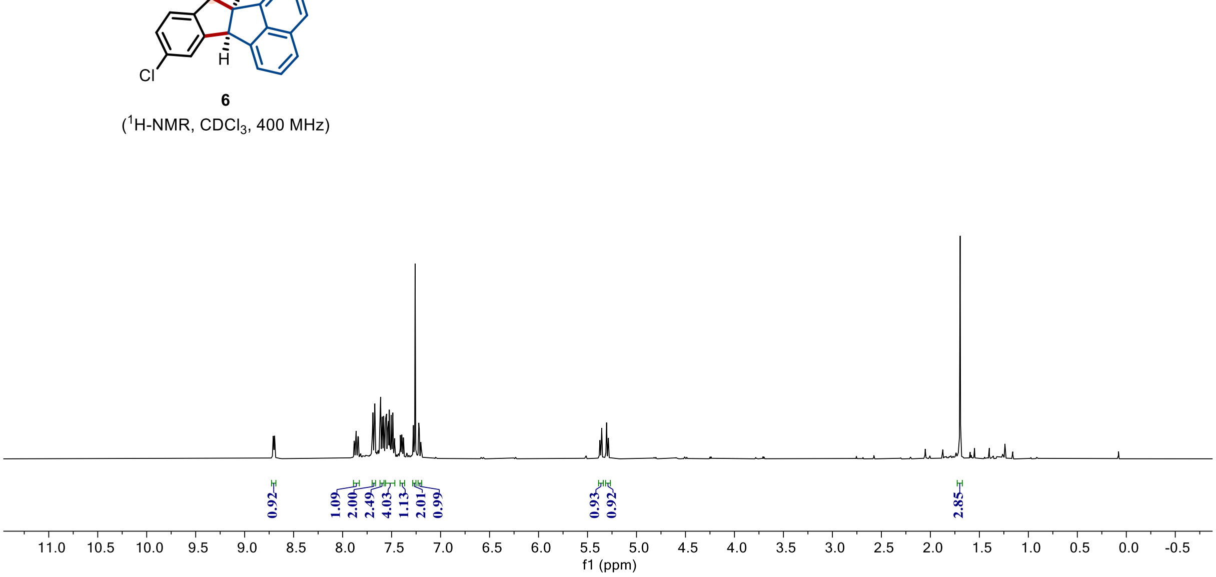


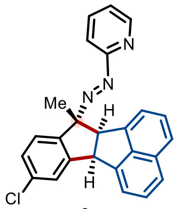


**6**
($^{13}$C-NMR, $CDCl_3$, 101 MHz)

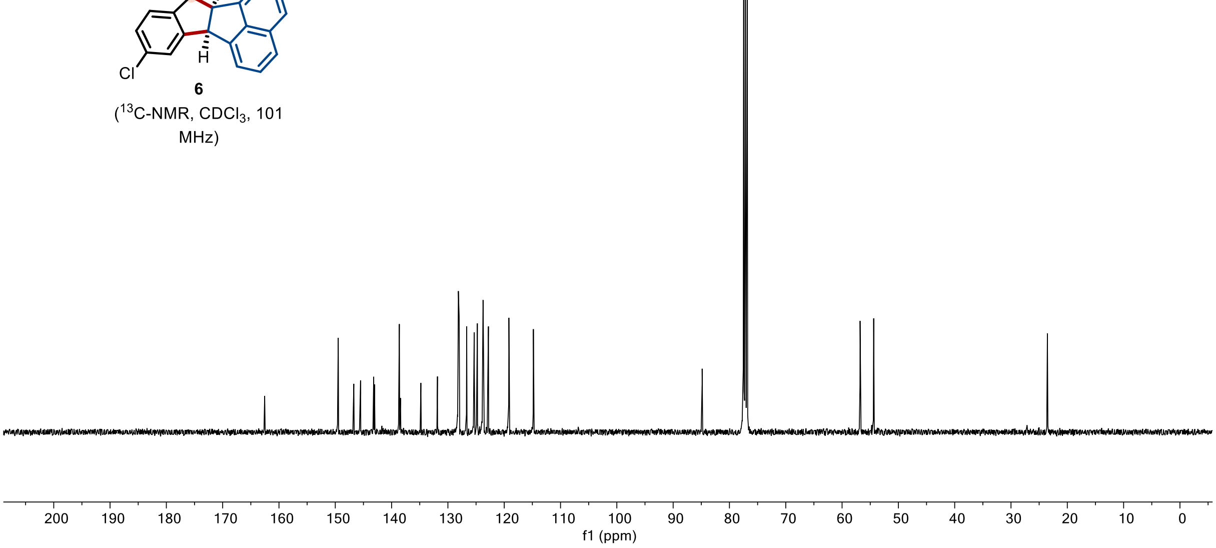

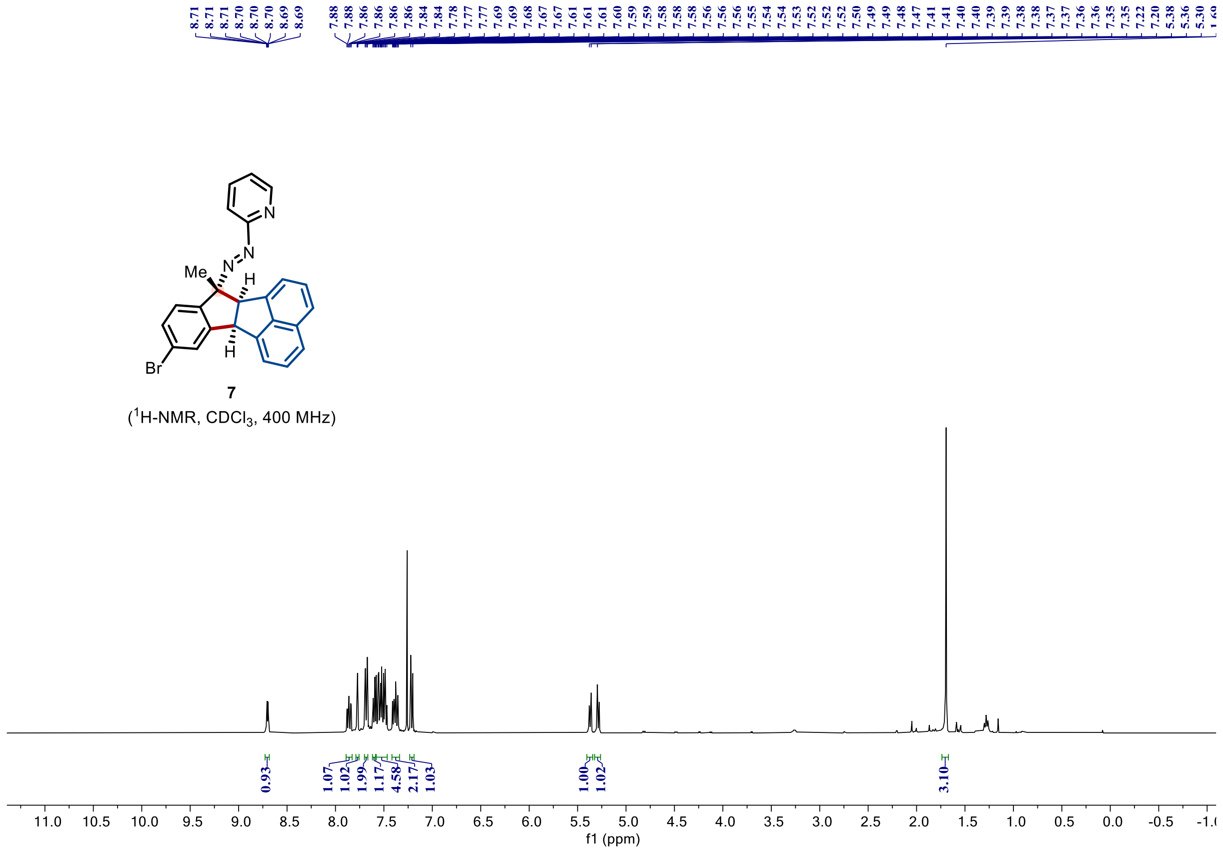


7

($^{1}$H-NMR, $CDCl_3$, 400 MHz)

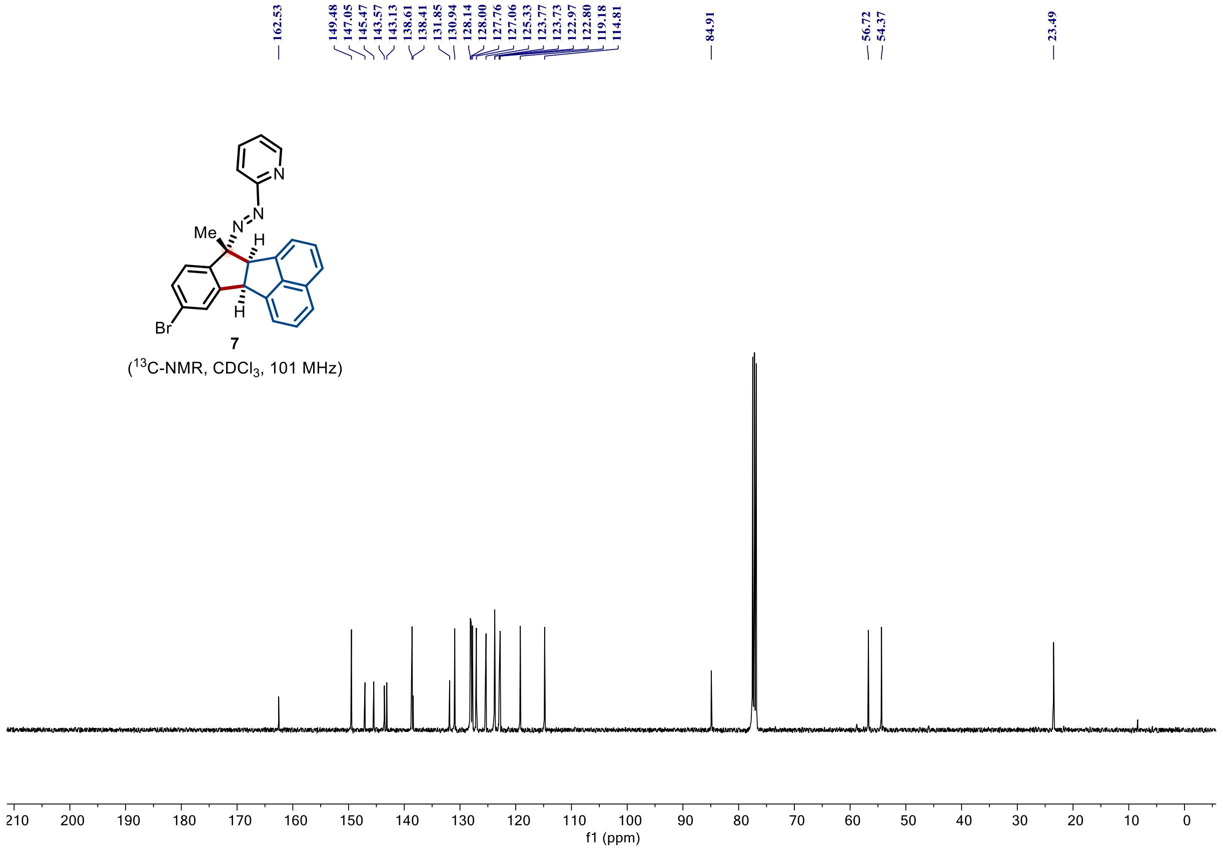


7

($^{13}$C-NMR, $CDCl_3$, 101 MHz)

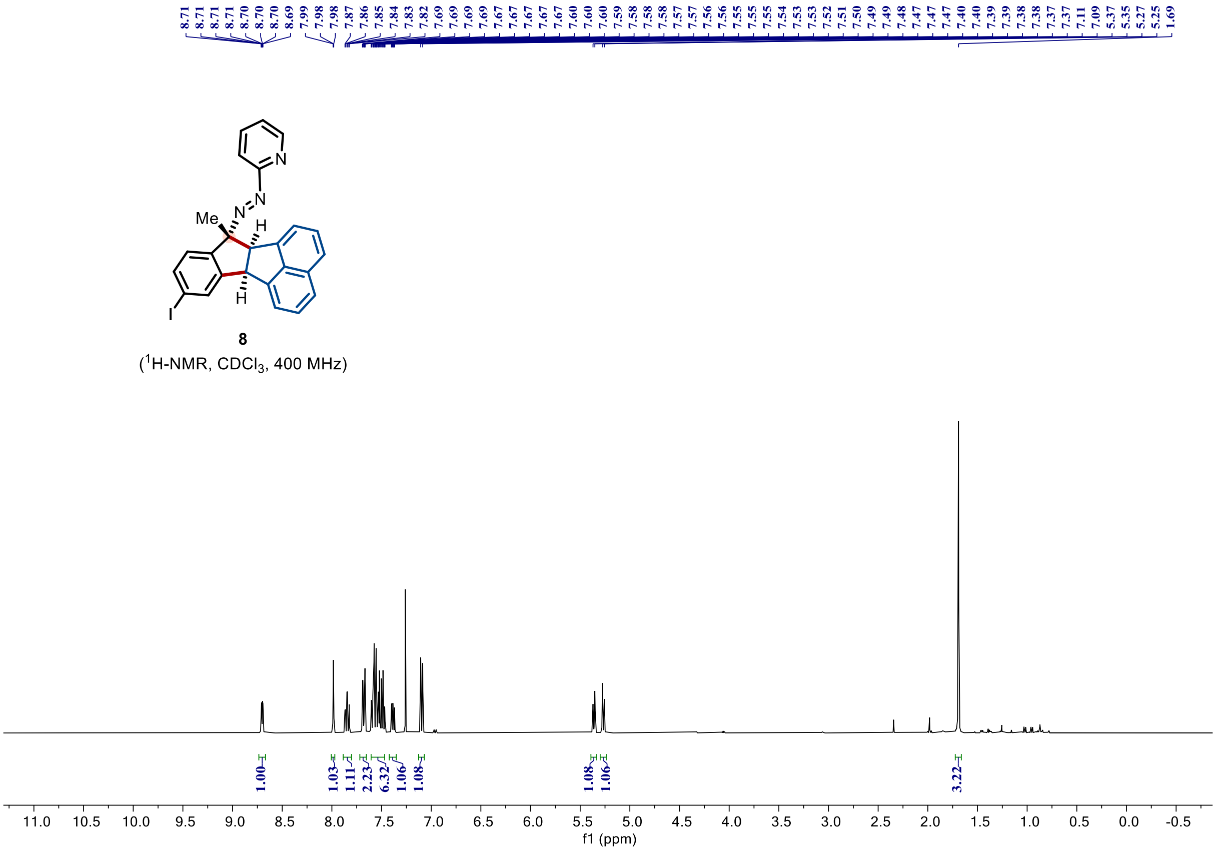


**8**
($^{1}$H-NMR, $CDCl_3$, 400 MHz)

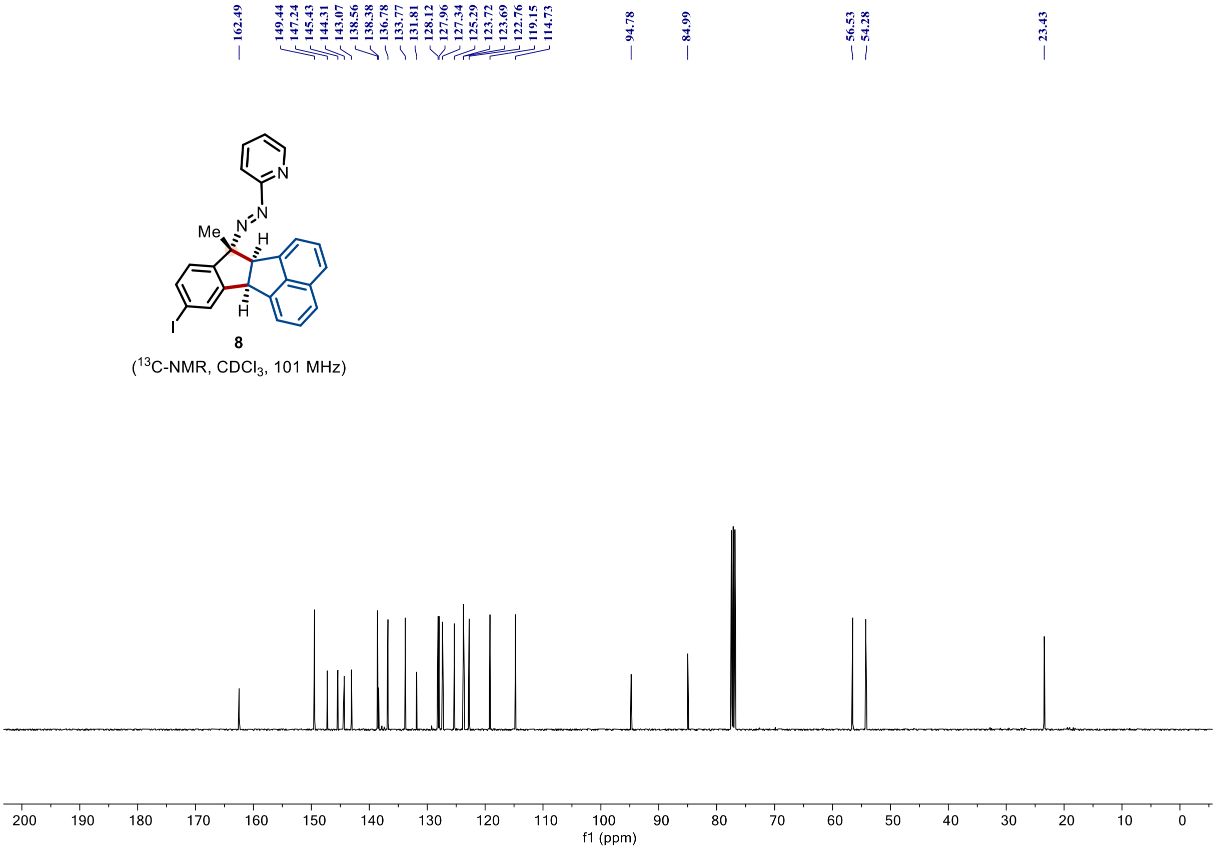


**8**
($^{13}$C-NMR, $CDCl_3$, 101 MHz)

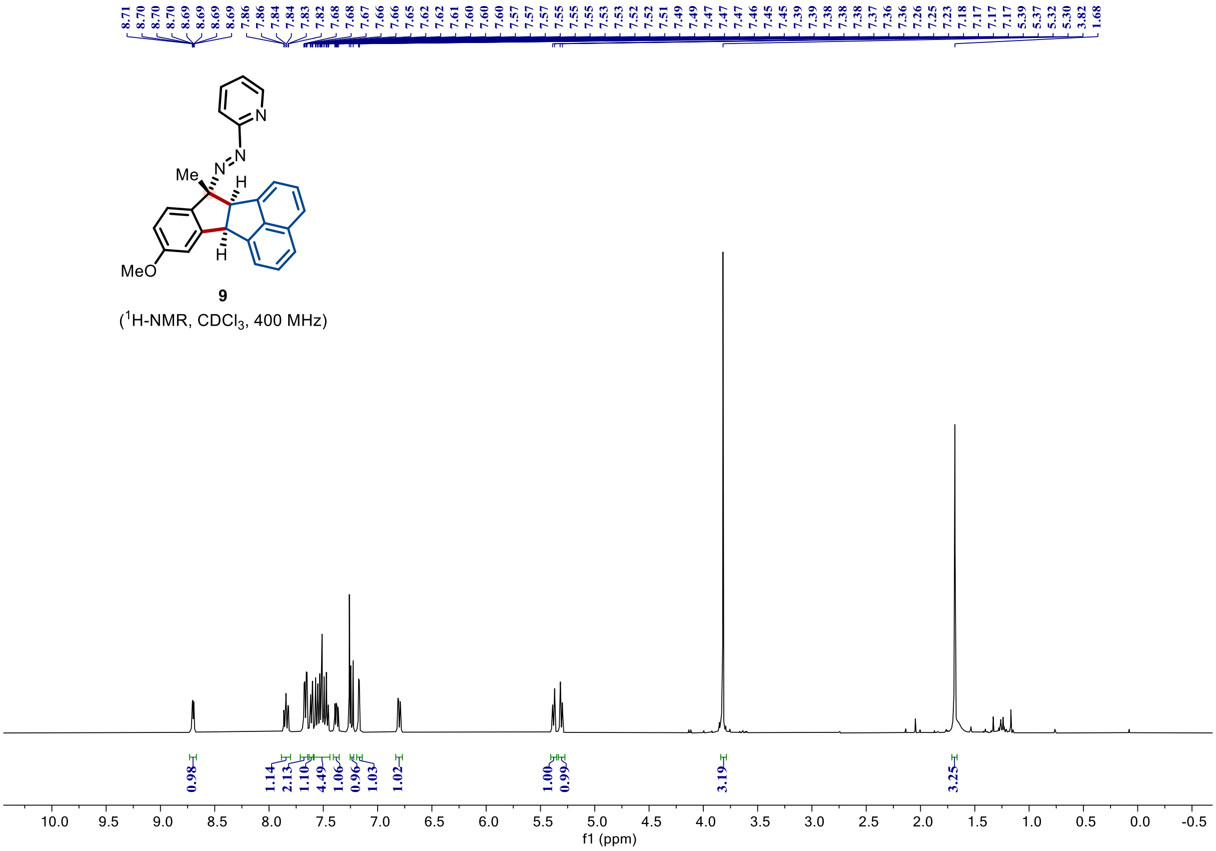

8.71 8.70 8.70 8.70 8.69 8.69 8.69 7.86 7.84 7.84 7.83 7.82 7.68 7.67 7.66 7.65 7.62 7.62 7.61 7.60 7.60 7.60 7.57 7.57 7.57 7.55 7.55 7.55 7.53 7.53 7.52 7.52 7.51 7.49 7.49 7.47 7.47 7.46 7.45 7.45 7.39 7.39 7.38 7.38 7.37 7.36 7.26 7.25 7.23 7.18 7.17 7.17 7.17 5.39 5.37 5.32 5.30 3.82 1.68
N
N
N
Me
H
H
MeO
9
($^1$H-NMR, $CDCl_3$, 400 MHz)
0.98 1.14 2.13 1.10 4.49 1.06 0.96 1.03 1.02 1.00 0.99 3.19 3.25
10.0 9.5 9.0 8.5 8.0 7.5 7.0 6.5 6.0 5.5 5.0 4.5 4.0 3.5 3.0 2.5 2.0 1.5 1.0 0.5 0.0 -0.5
f1 (ppm)


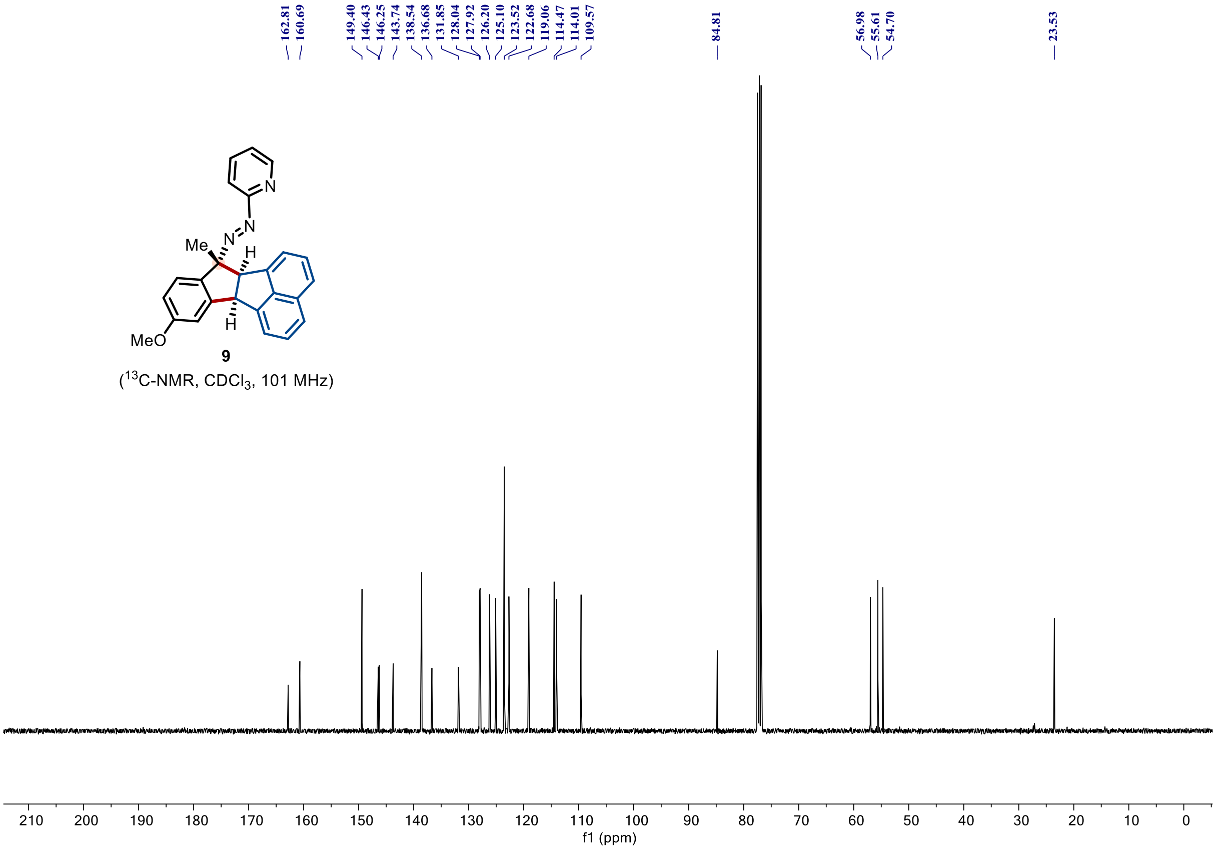

162.81 160.69 149.40 146.43 146.25 143.74 138.54 136.68 131.85 128.04 127.92 126.20 125.10 123.52 122.68 119.06 114.47 114.01 109.57 84.81 56.98 55.61 54.70 23.53
N
N
N
Me
H
H
MeO
9
($^{13}$C-NMR, $CDCl_3$, 101 MHz)
210 200 190 180 170 160 150 140 130 120 110 100 90 80 70 60 50 40 30 20 10 0
f1 (ppm)

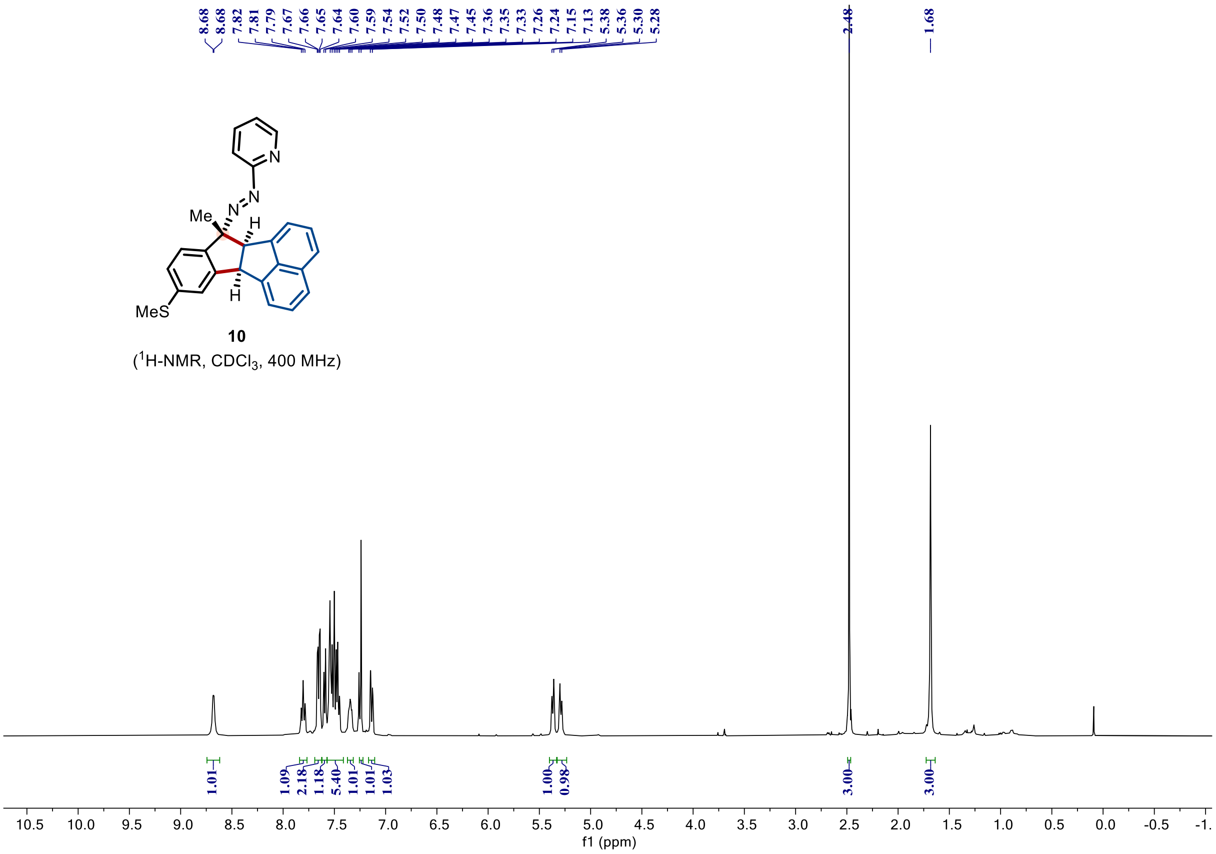
8.68 8.68 7.82 7.81 7.79 7.67 7.66 7.65 7.64 7.60 7.59 7.54 7.52 7.50 7.48 7.47 7.45 7.36 7.35 7.33 7.26 7.24 7.15 7.13 5.38 5.36 5.30 5.28
2.48
1.68
N
N
N
Me
H
H
MeS
10
($^1$H-NMR, $CDCl_3$, 400 MHz)
1.01
1.09 2.18 1.18 5.40 1.01 1.01 1.03
1.00 0.98
3.00
3.00
10.5 10.0 9.5 9.0 8.5 8.0 7.5 7.0 6.5 6.0 5.5 5.0 4.5 4.0 3.5 3.0 2.5 2.0 1.5 1.0 0.5 0.0 -0.5 -1.
f1 (ppm)

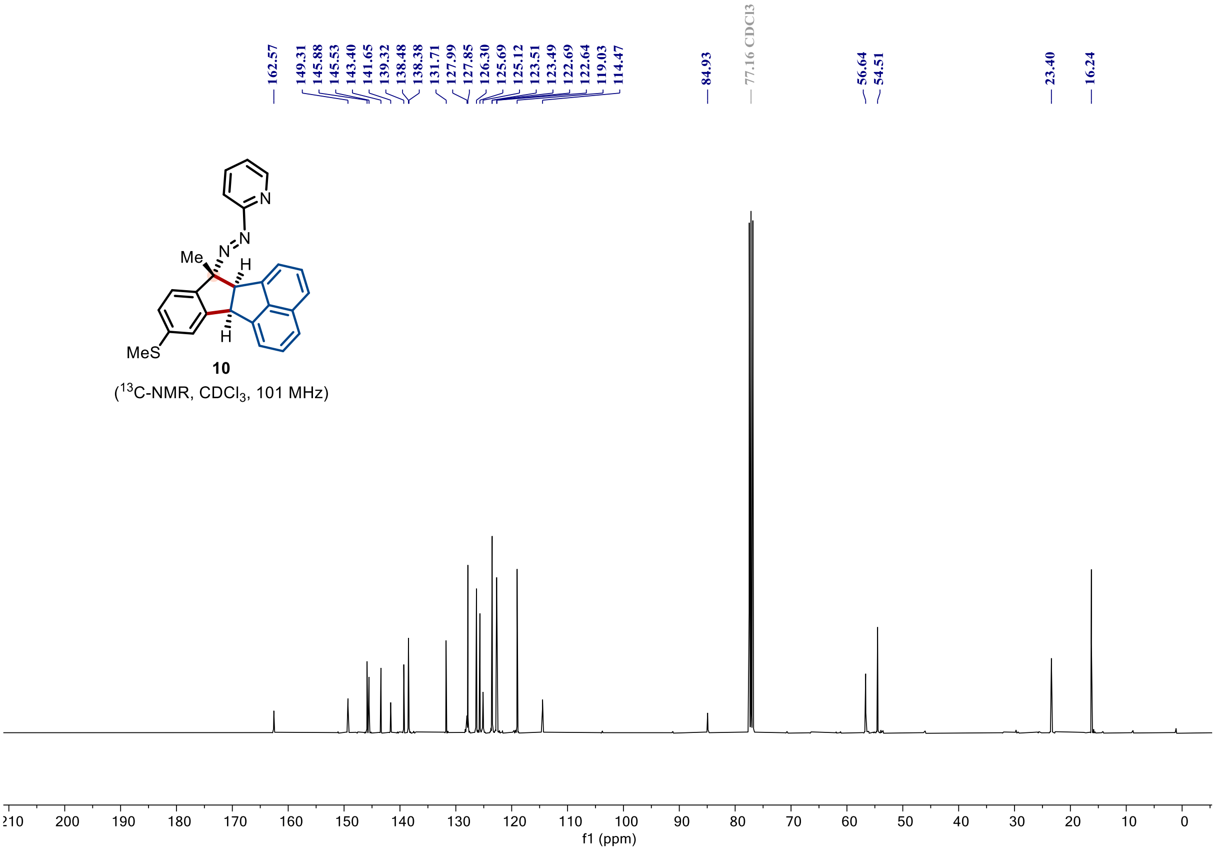
162.57
149.31 145.88 145.53 143.40 141.65 139.32 138.48 138.38 131.71 127.99 127.85 126.30 125.69 125.12 123.51 123.49 122.69 122.64 119.03 114.47
84.93
77.16 CDCl3
56.64 54.51
23.40
16.24
N
N
N
Me
H
H
MeS
10
($^{13}$C-NMR, $CDCl_3$, 101 MHz)
210 200 190 180 170 160 150 140 130 120 110 100 90 80 70 60 50 40 30 20 10 0
f1 (ppm)

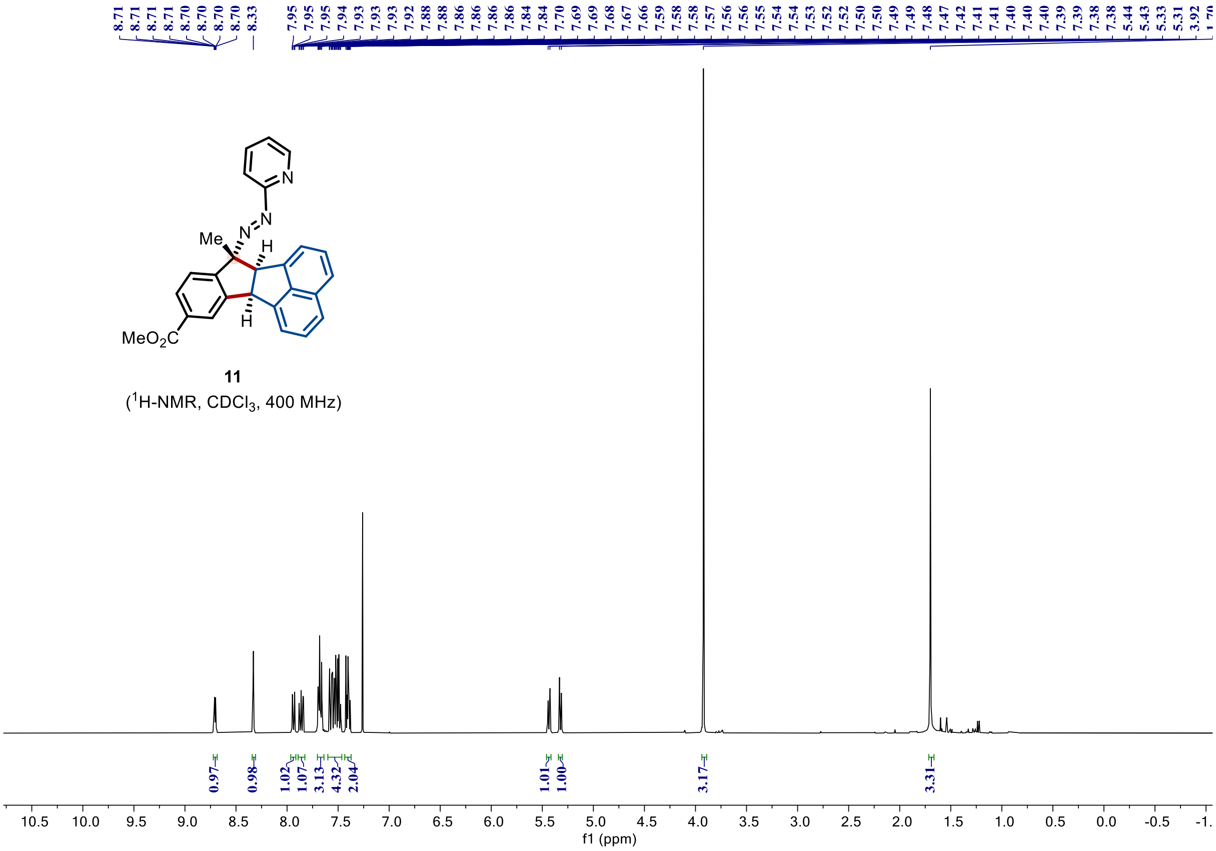

11
(1H-NMR, CDCl3, 400 MHz)
8.71
8.71
8.71
8.71
8.70
8.70
8.70
8.70
8.33
7.95
7.95
7.95
7.94
7.93
7.93
7.93
7.92
7.88
7.88
7.86
7.86
7.86
7.86
7.84
7.84
7.70
7.69
7.69
7.68
7.67
7.66
7.59
7.58
7.58
7.57
7.56
7.56
7.55
7.54
7.54
7.53
7.52
7.52
7.50
7.50
7.49
7.49
7.48
7.47
7.42
7.41
7.41
7.40
7.40
7.40
7.39
7.39
7.38
7.38
5.44
5.43
5.33
5.31
3.92
0.97
0.98
1.02
1.07
3.13
4.32
2.04
1.01
1.00
3.17
3.31
f1 (ppm)

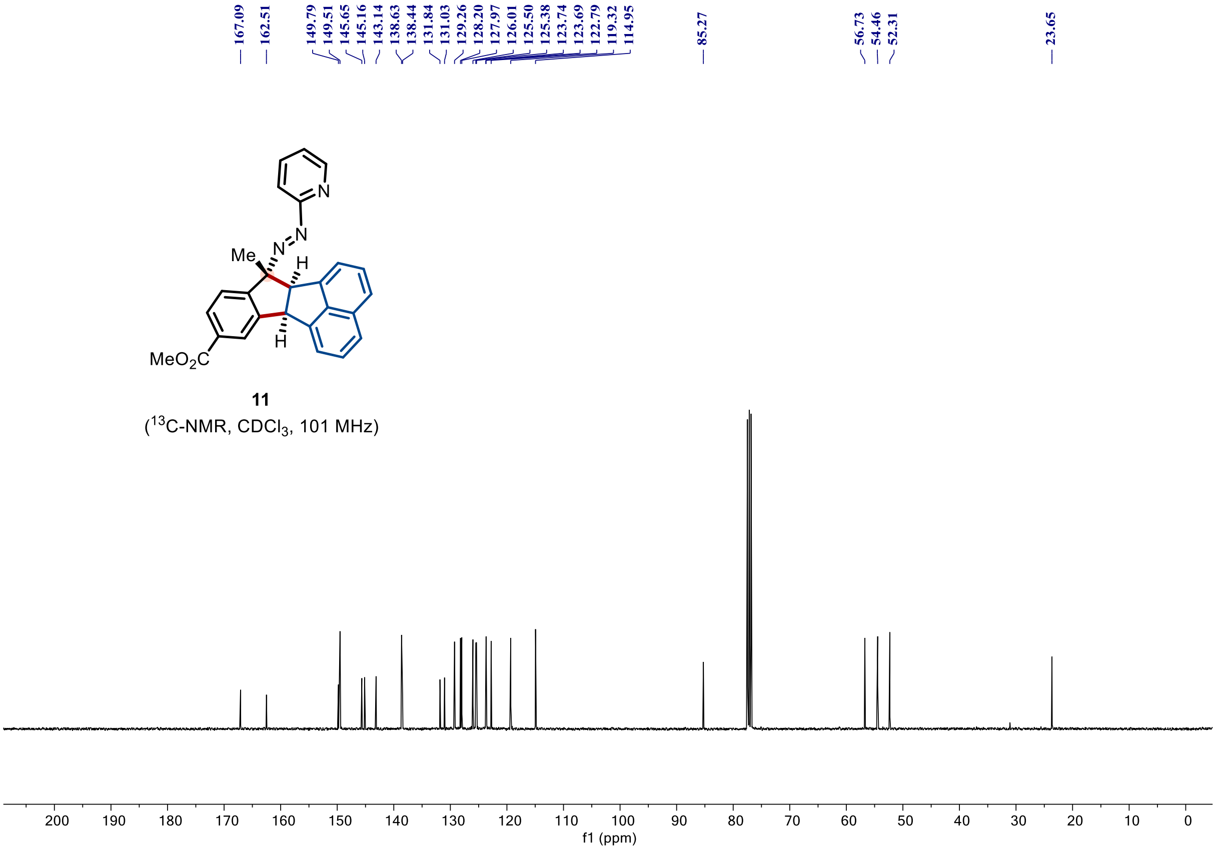

11
(13C-NMR, CDCl3, 101 MHz)
167.09
162.51
149.79
149.51
145.65
145.16
143.14
138.63
138.44
131.84
131.03
129.26
128.20
127.97
126.01
125.50
125.38
123.74
123.69
122.79
119.32
114.95
85.27
56.73
54.46
52.31
23.65
f1 (ppm)

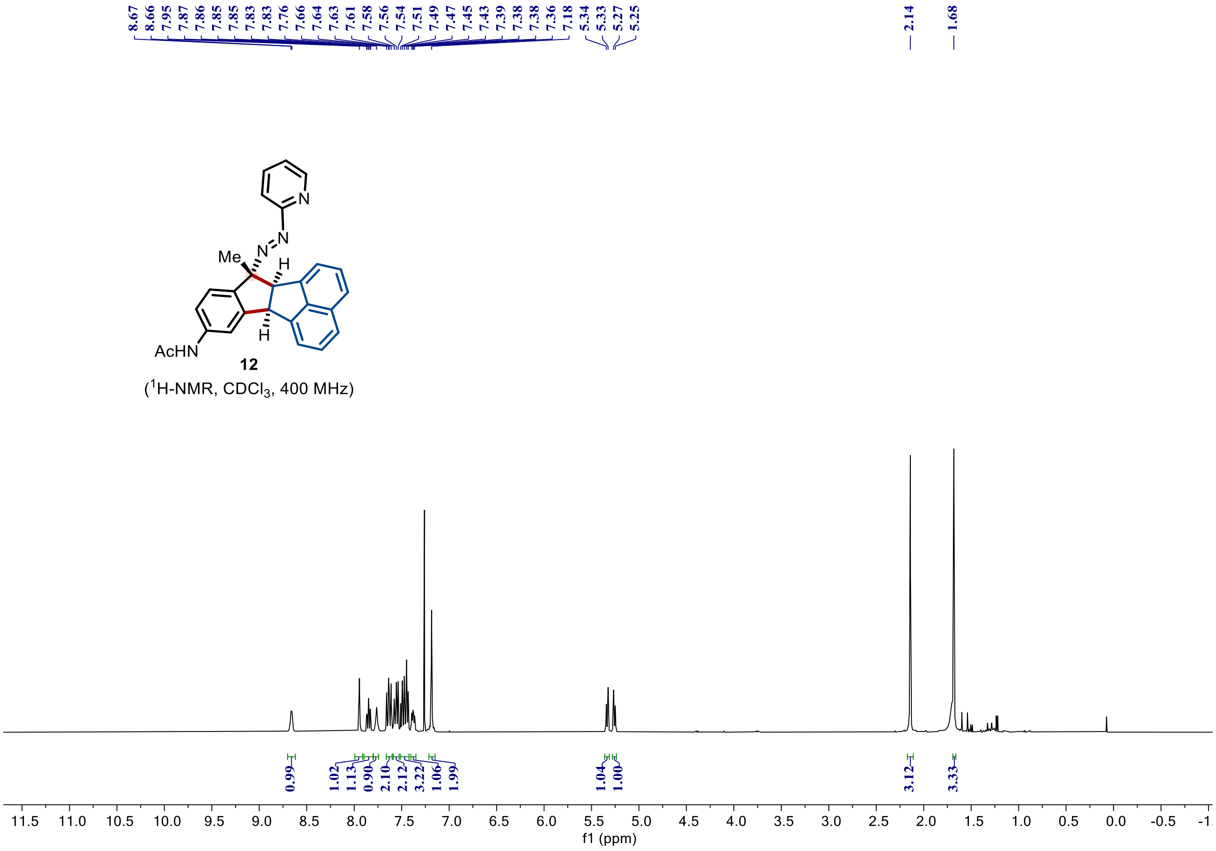

8.67
8.66
7.95
7.87
7.86
7.85
7.85
7.83
7.83
7.76
7.66
7.64
7.63
7.61
7.58
7.56
7.54
7.51
7.49
7.47
7.45
7.43
7.39
7.38
7.38
7.36
7.18
5.34
5.33
5.27
5.25
2.14
1.68
Me
N
N
N
H
H
AcHN
12
(1H-NMR, CDCl3, 400 MHz)
0.99
1.02
1.13
0.90
2.10
2.12
3.22
1.06
1.99
1.04
1.00
3.12
3.33
f1 (ppm)


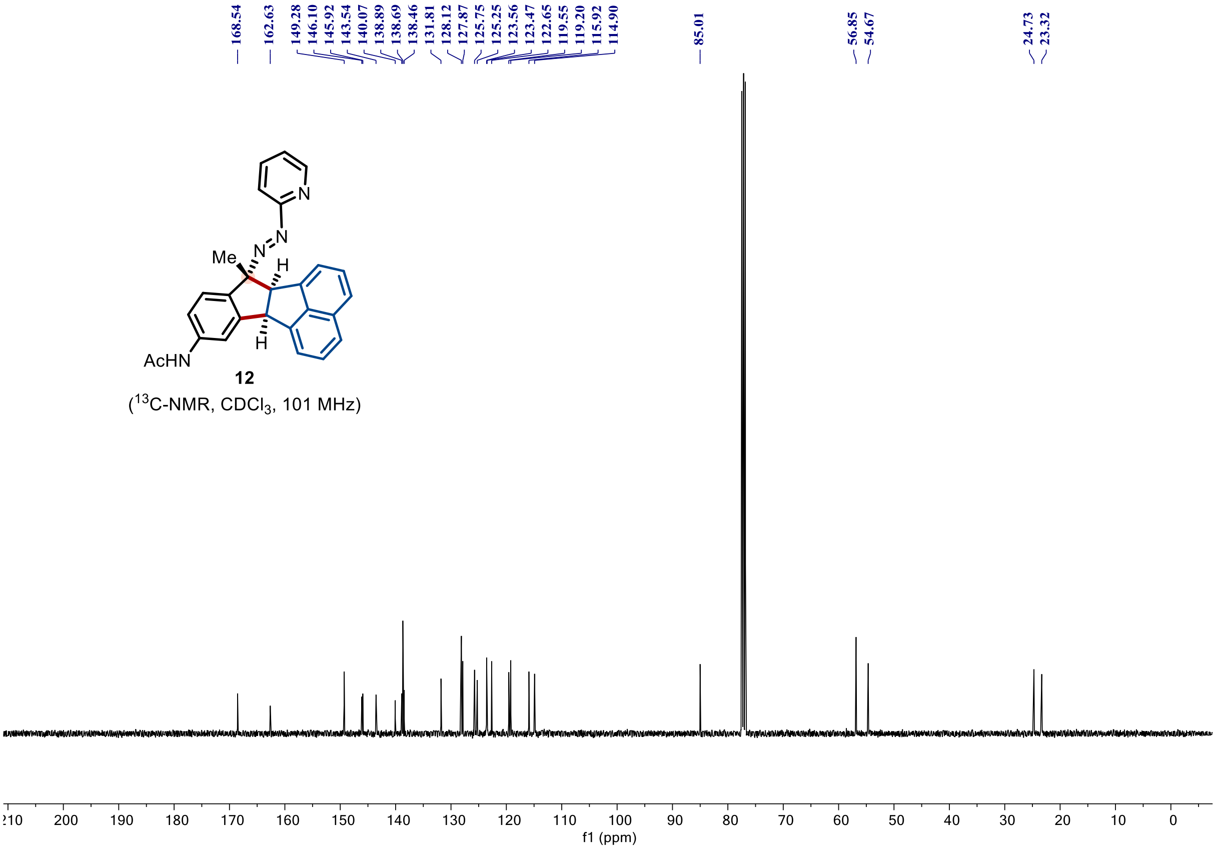

168.54
162.63
149.28
146.10
145.92
143.54
140.07
138.89
138.69
138.46
131.81
128.12
127.87
125.75
125.25
123.56
123.47
122.65
119.55
119.20
115.92
114.90
85.01
56.85
54.67
24.73
23.32
Me
N
N
N
H
H
AcHN
12
(13C-NMR, CDCl3, 101 MHz)
f1 (ppm)

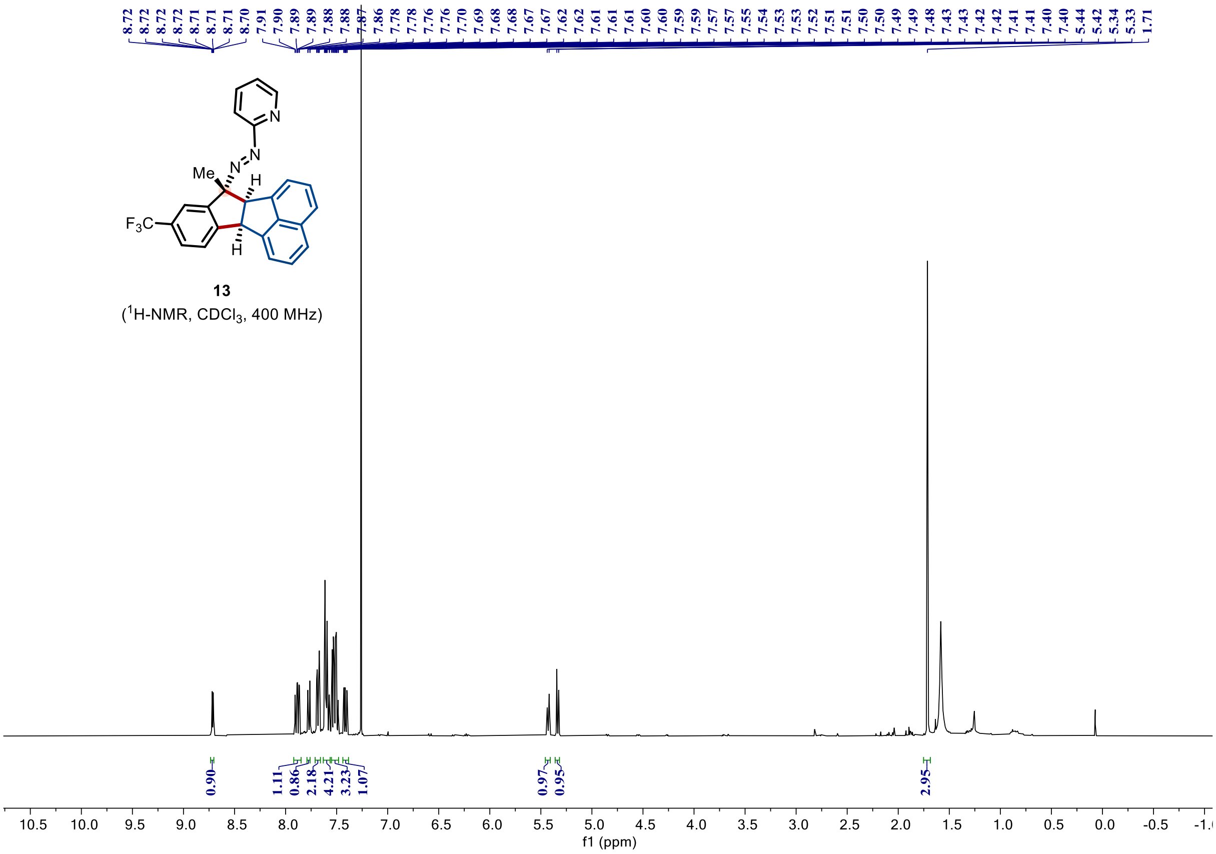
8.72 8.72 8.72 8.72 8.71 8.71 8.71 8.70 7.91 7.90 7.89 7.89 7.88 7.88 7.87 7.86 7.78 7.78 7.76 7.76 7.70 7.69 7.68 7.68 7.67 7.67 7.62 7.62 7.61 7.61 7.61 7.60 7.59 7.59 7.57 7.57 7.55 7.54 7.53 7.53 7.52 7.51 7.51 7.50 7.50 7.49 7.49 7.48 7.43 7.43 7.42 7.42 7.41 7.41 7.40 5.44 5.42 5.34 5.33 1.71
N
N
N
Me
H
F3C
H
13
(1H-NMR, CDCl3, 400 MHz)
0.90
1.11
0.86
2.18
4.21
3.23
1.07
0.97
0.95
2.95
10.5 10.0 9.5 9.0 8.5 8.0 7.5 7.0 6.5 6.0 5.5 5.0 4.5 4.0 3.5 3.0 2.5 2.0 1.5 1.0 0.5 0.0 -0.5 -1.
f1 (ppm)

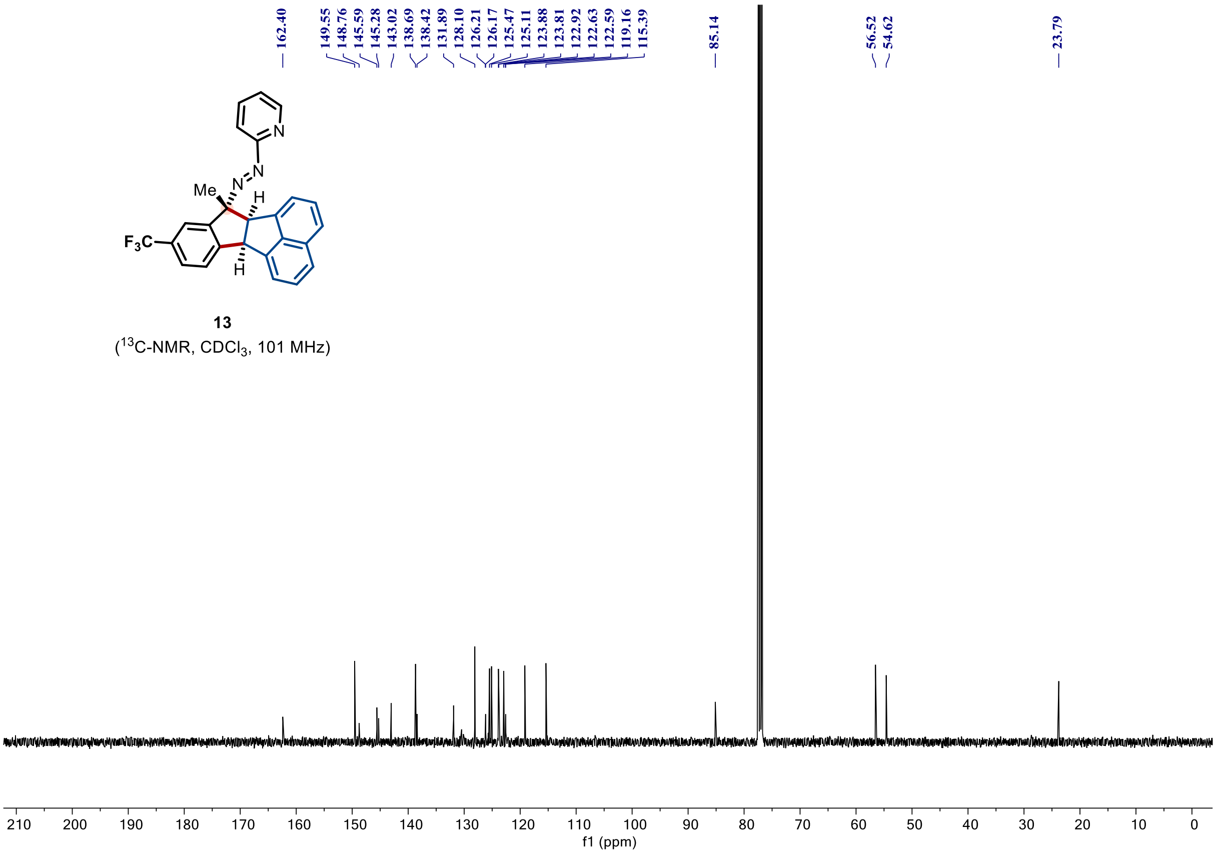
162.40
149.55 148.76 145.59 145.28 143.02 138.69 138.42 131.89 128.10 126.21 126.17 125.47 125.11 123.88 123.81 122.92 122.63 122.59 119.16 115.39
85.14
56.52 54.62
23.79
N
N
N
Me
H
F3C
H
13
(13C-NMR, CDCl3, 101 MHz)
210 200 190 180 170 160 150 140 130 120 110 100 90 80 70 60 50 40 30 20 10 0
f1 (ppm)

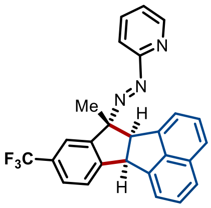

**13**

($^{19}$F-NMR, $CDCl_3$, 377 MHz)

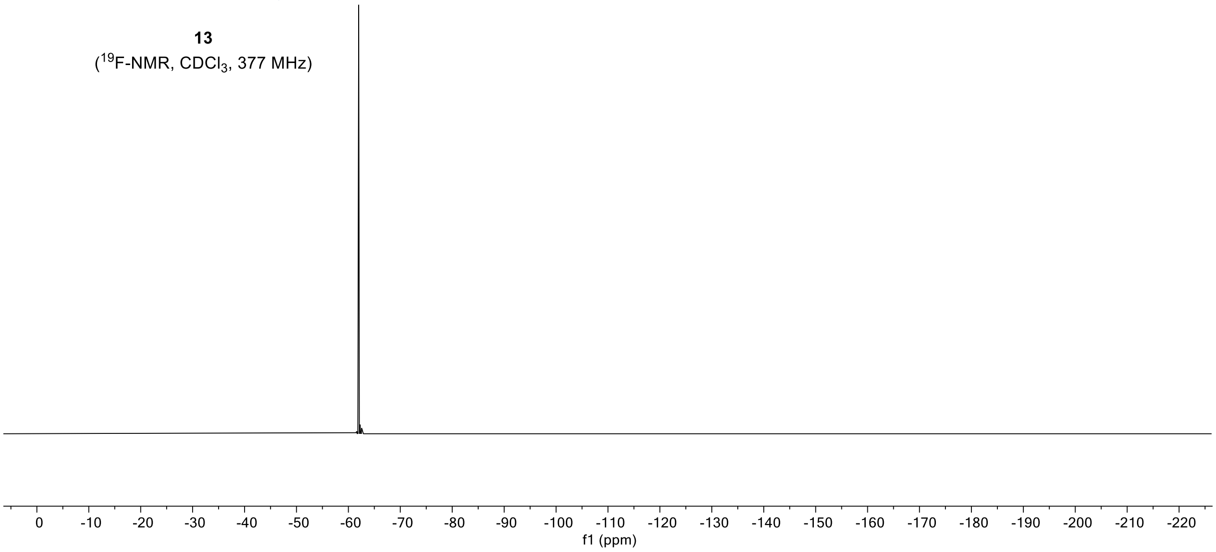

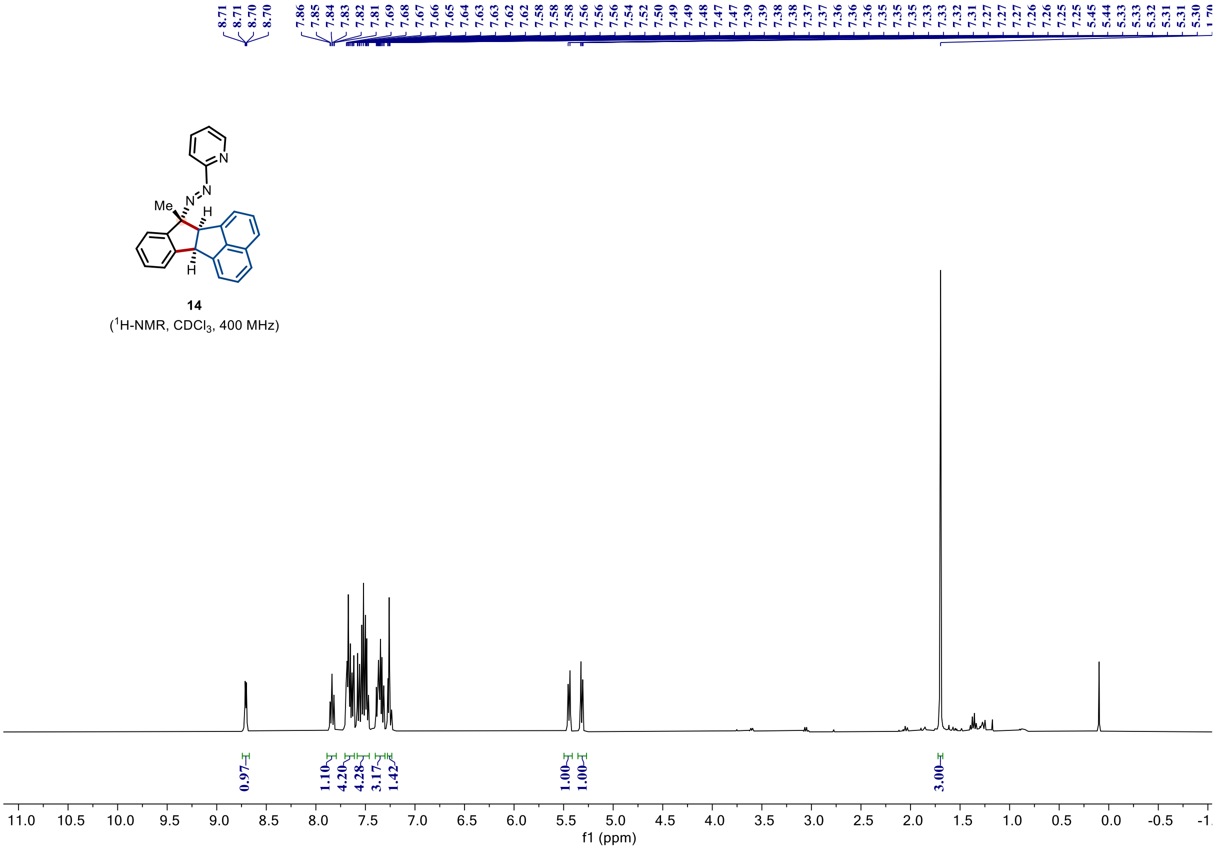


**14**
($^1$H-NMR, $CDCl_3$, 400 MHz)

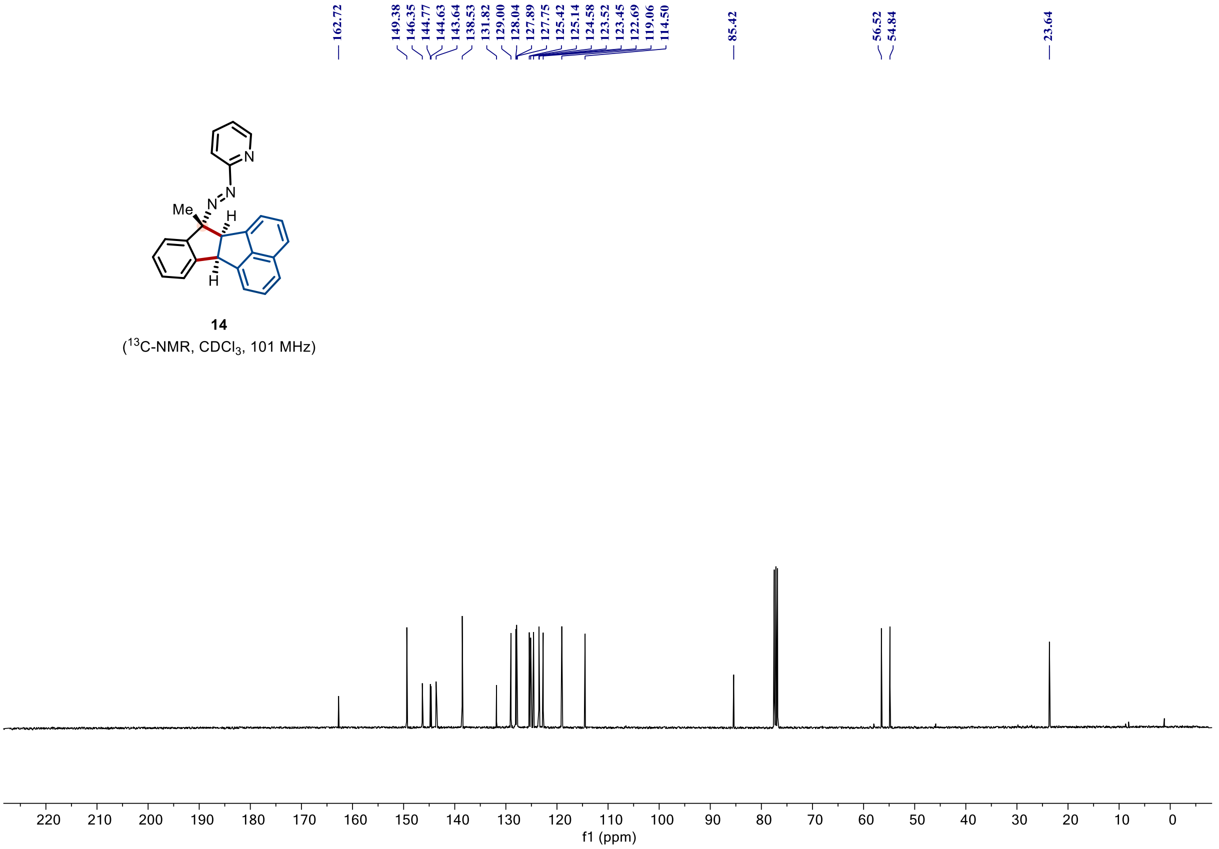


**14**
($^{13}$C-NMR, $CDCl_3$, 101 MHz)

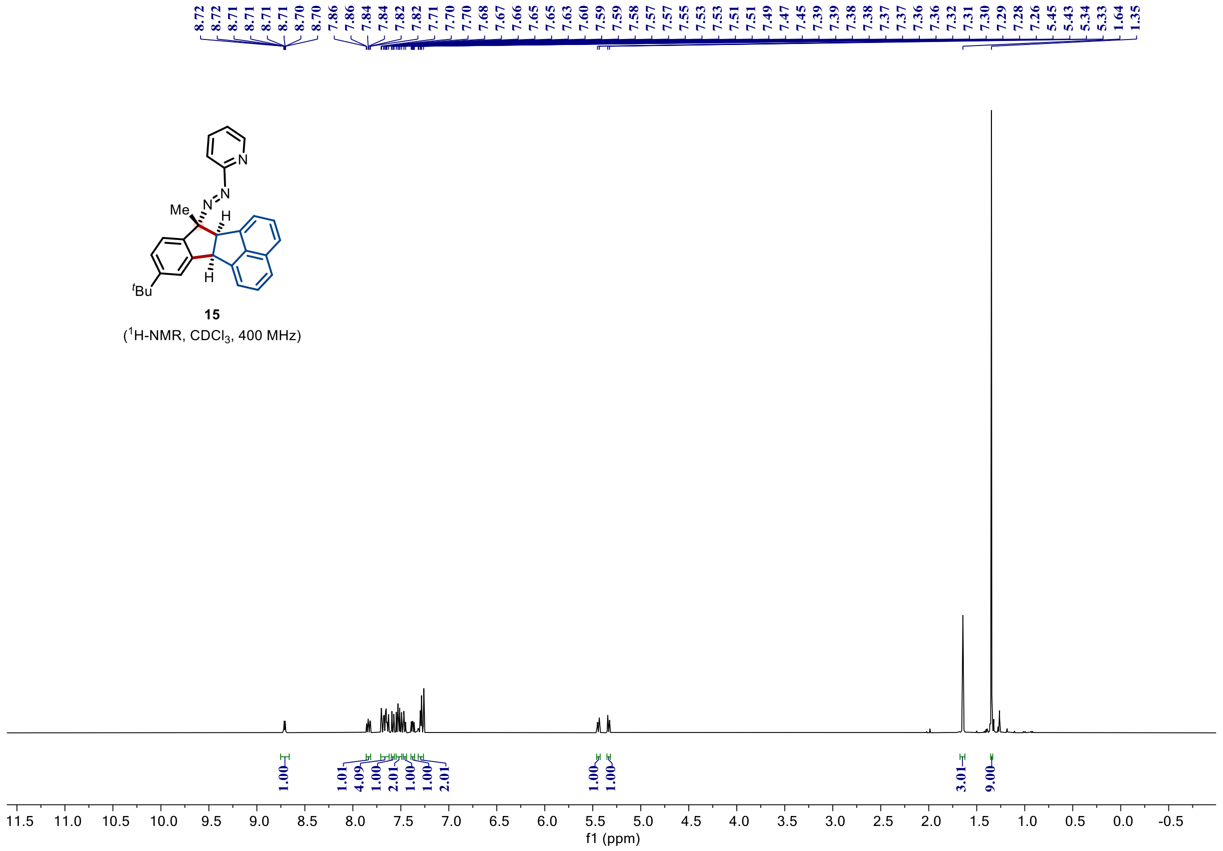


**15**
($^1$H-NMR, $CDCl_3$, 400 MHz)

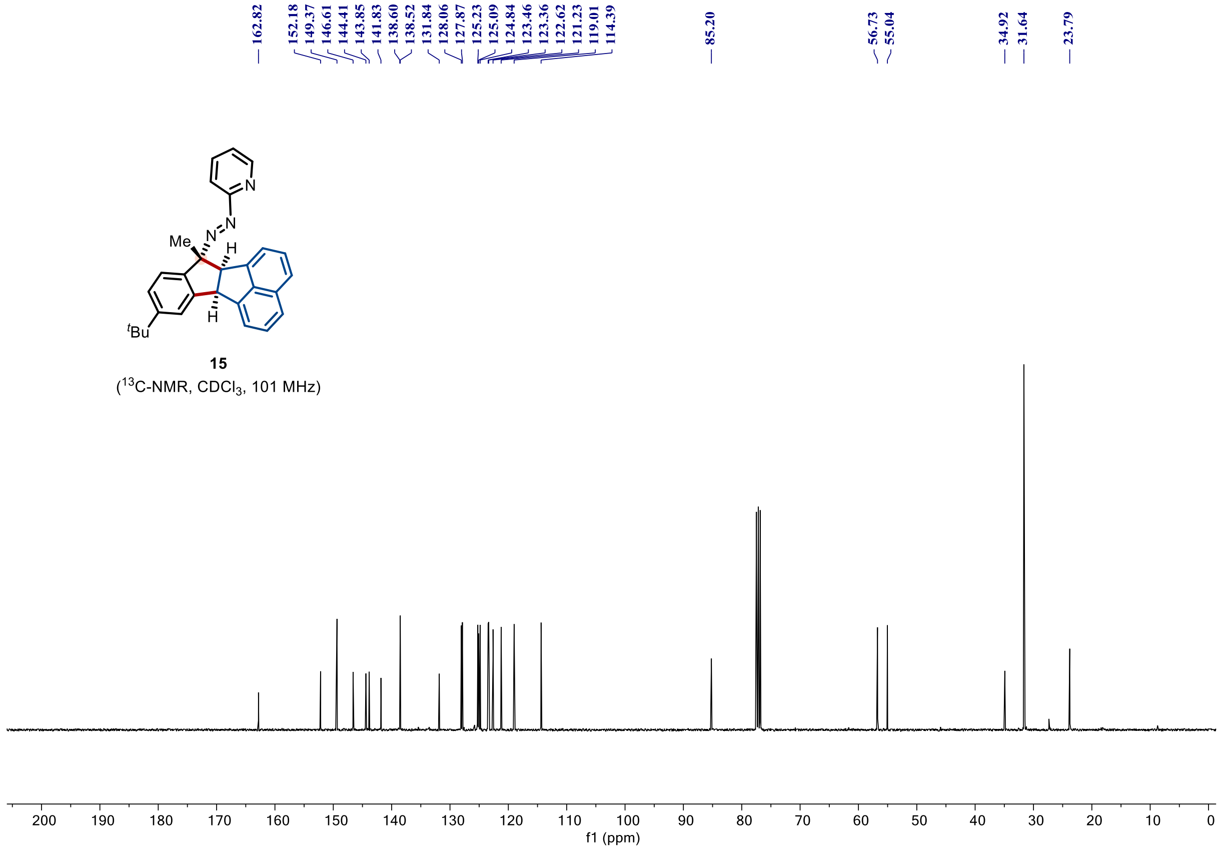


**15**
($^{13}$C-NMR, $CDCl_3$, 101 MHz)

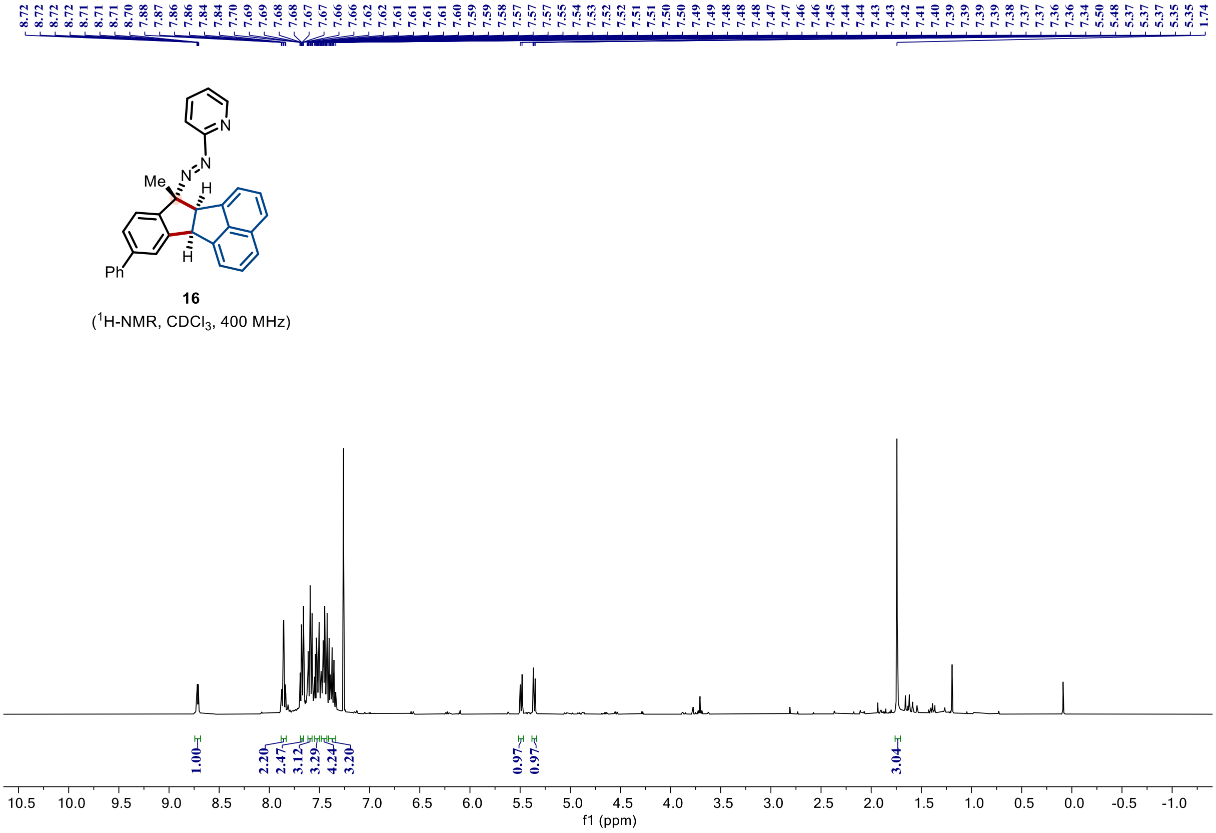


**16**

($^{1}$H-NMR, $CDCl_3$, 400 MHz)

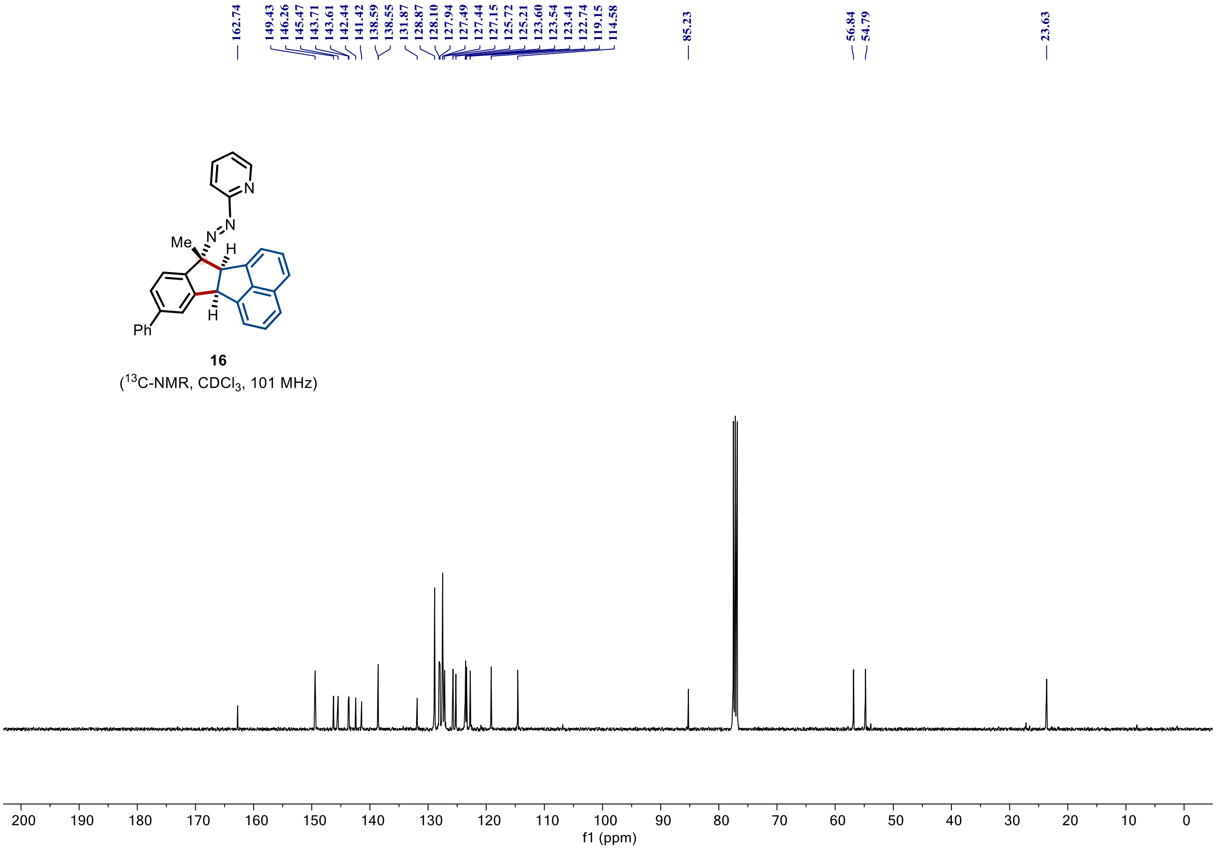


**16**

($^{13}$C-NMR, $CDCl_3$, 101 MHz)

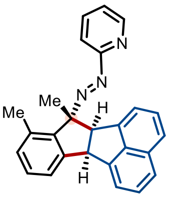

**17**
($^{1}$H-NMR, $CDCl_3$, 400 MHz)

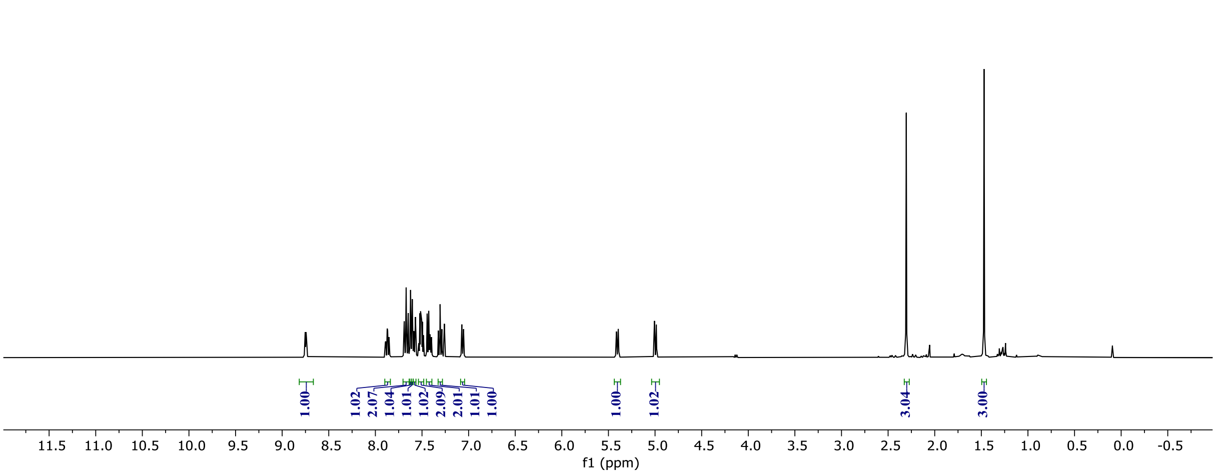


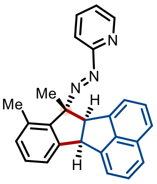

**17**
($^{13}$C-NMR, $CDCl_3$, 101 MHz)

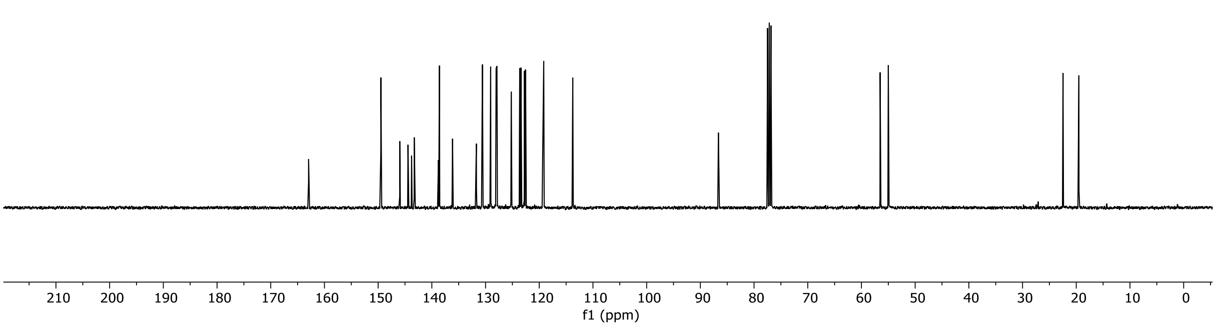

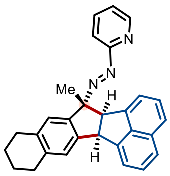

**18**
($^1$H-NMR, $CDCl_3$, 400 MHz)

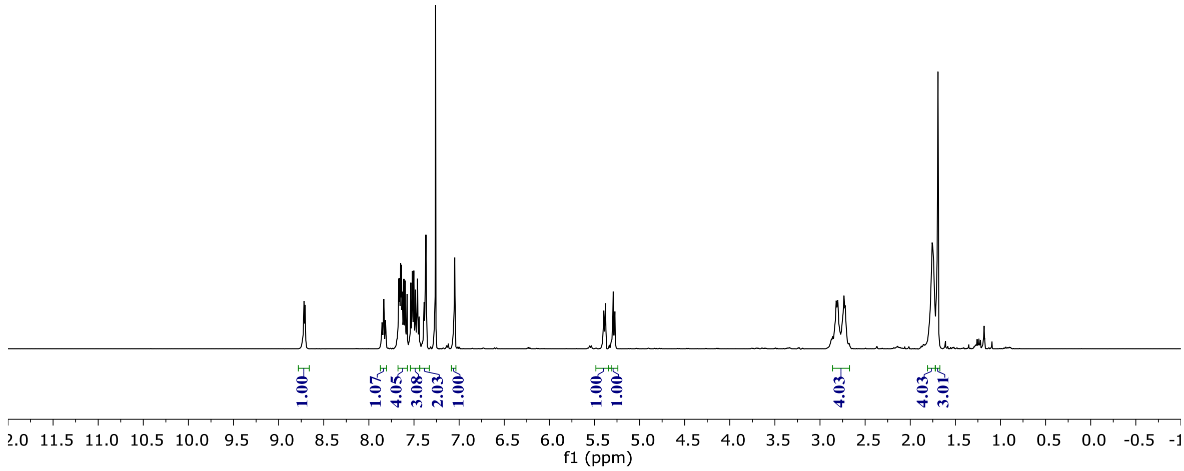


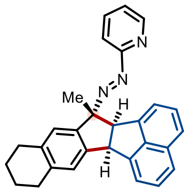

**18**
($^{13}$C-NMR, $CDCl_3$, 101 MHz)

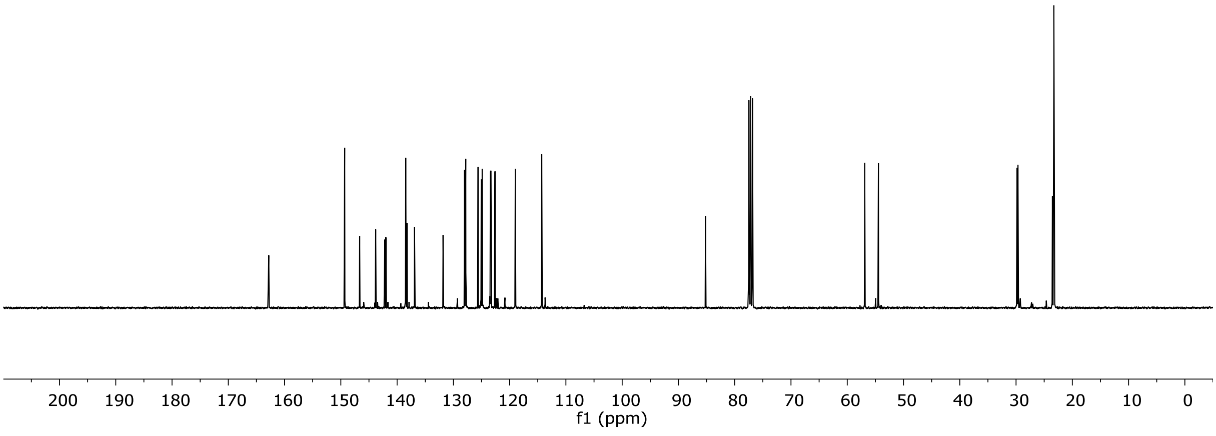

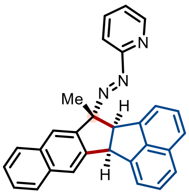

**19**

($^{1}$H-NMR, $CDCl_3$, 400 MHz)

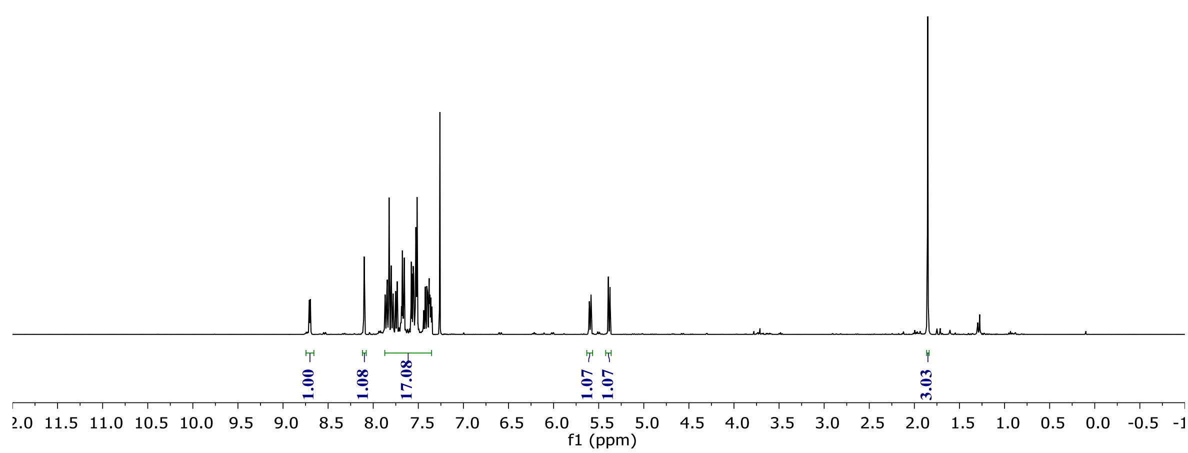


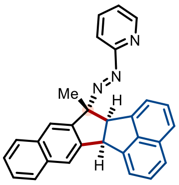

**19**

($^{13}$C-NMR, $CDCl_3$, 101 MHz)

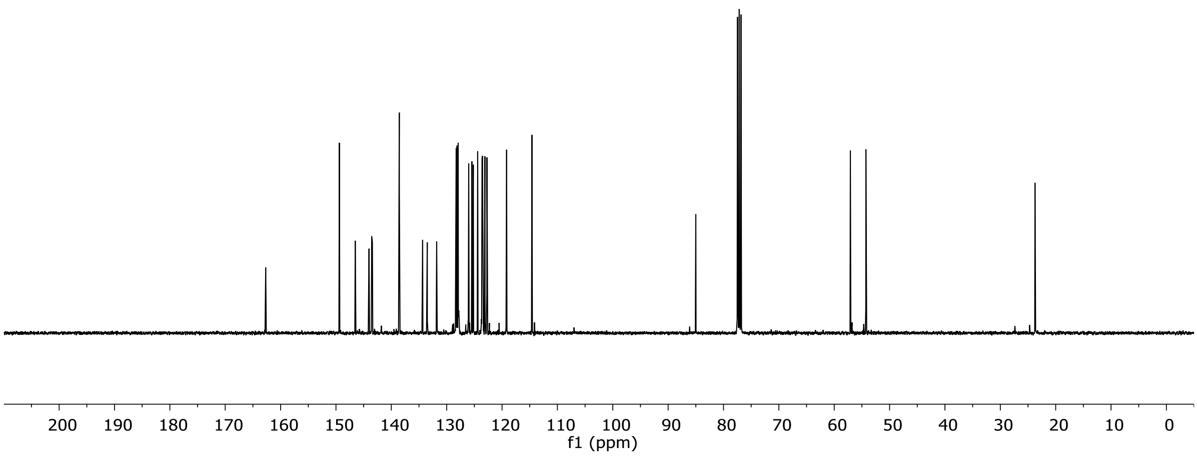

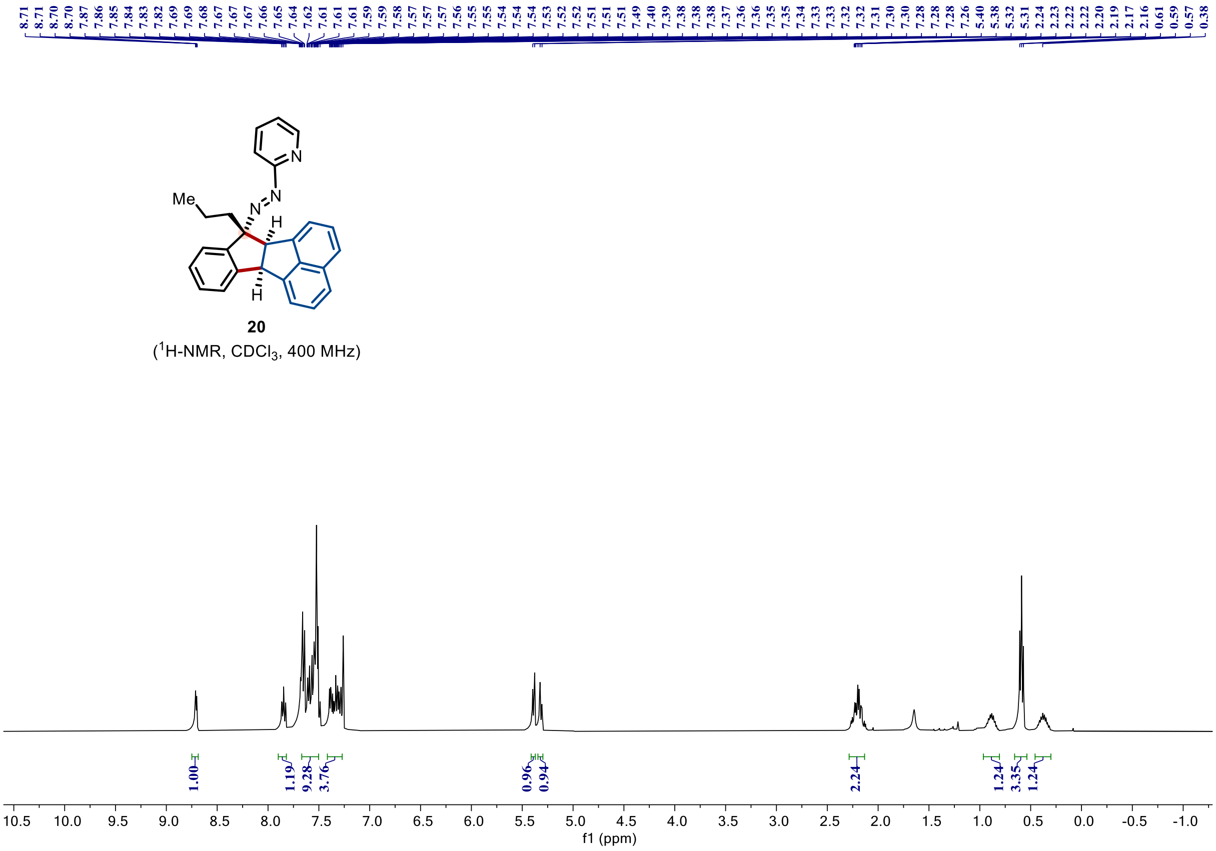

**20**
($^{1}$H-NMR, $CDCl_3$, 400 MHz)

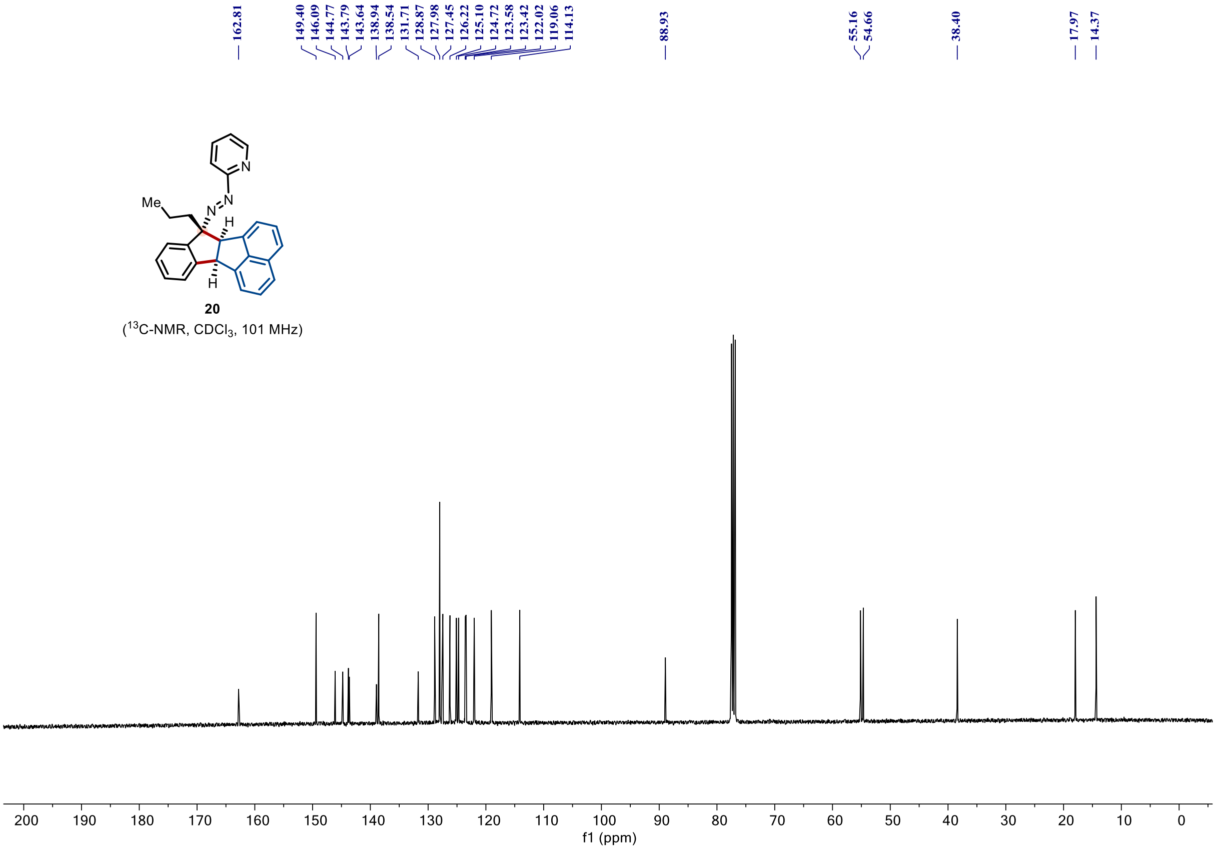

**20**
($^{13}$C-NMR, $CDCl_3$, 101 MHz)

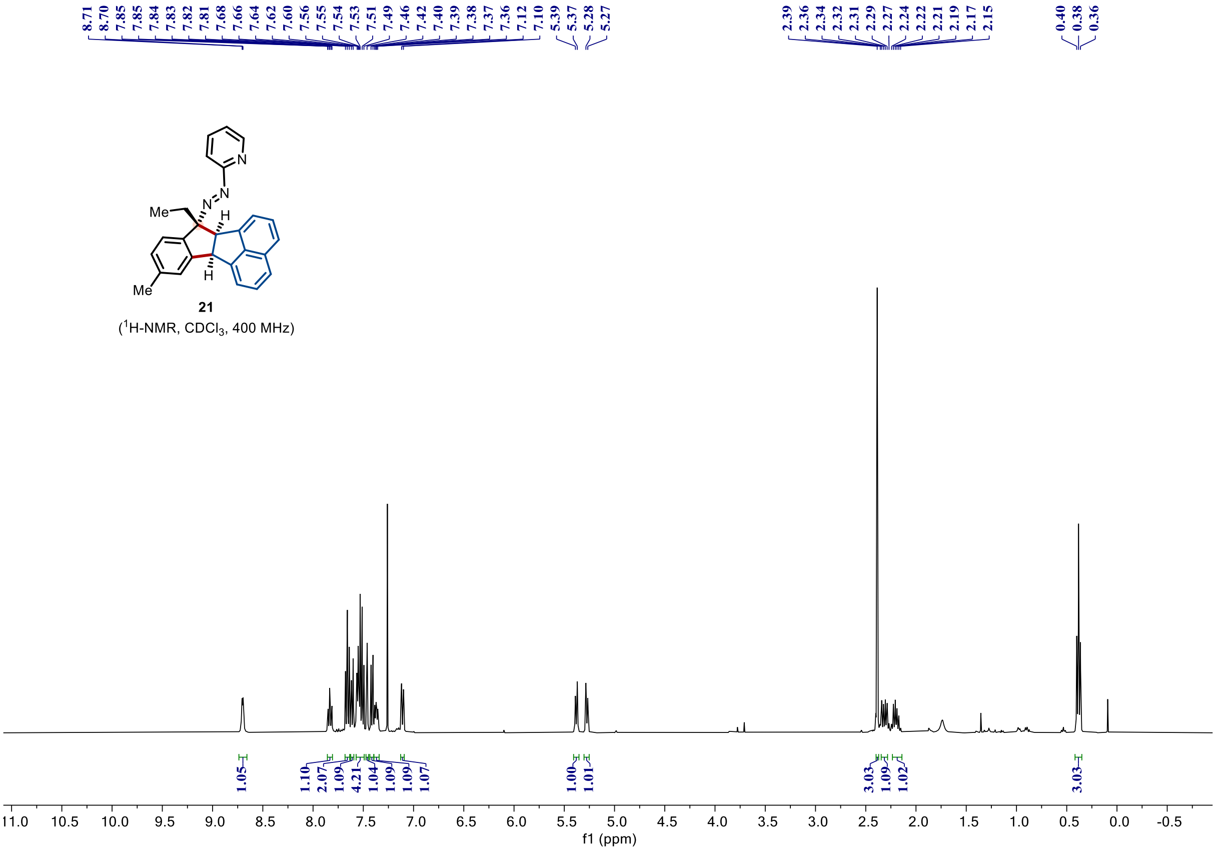
8.71
8.70
7.85
7.85
7.84
7.83
7.82
7.81
7.68
7.66
7.64
7.62
7.60
7.56
7.55
7.54
7.53
7.51
7.49
7.46
7.42
7.40
7.39
7.38
7.37
7.36
7.12
7.10
5.39
5.37
5.28
5.27
2.39
2.36
2.34
2.32
2.31
2.29
2.27
2.24
2.22
2.21
2.19
2.17
2.15
0.40
0.38
0.36
Me
N
N
N
H
H
Me
21
($^1$H-NMR, CDCl$_3$, 400 MHz)
1.05
1.10
2.07
1.09
4.21
1.04
1.09
1.09
1.07
1.00
1.01
3.03
1.09
1.02
3.03
f1 (ppm)

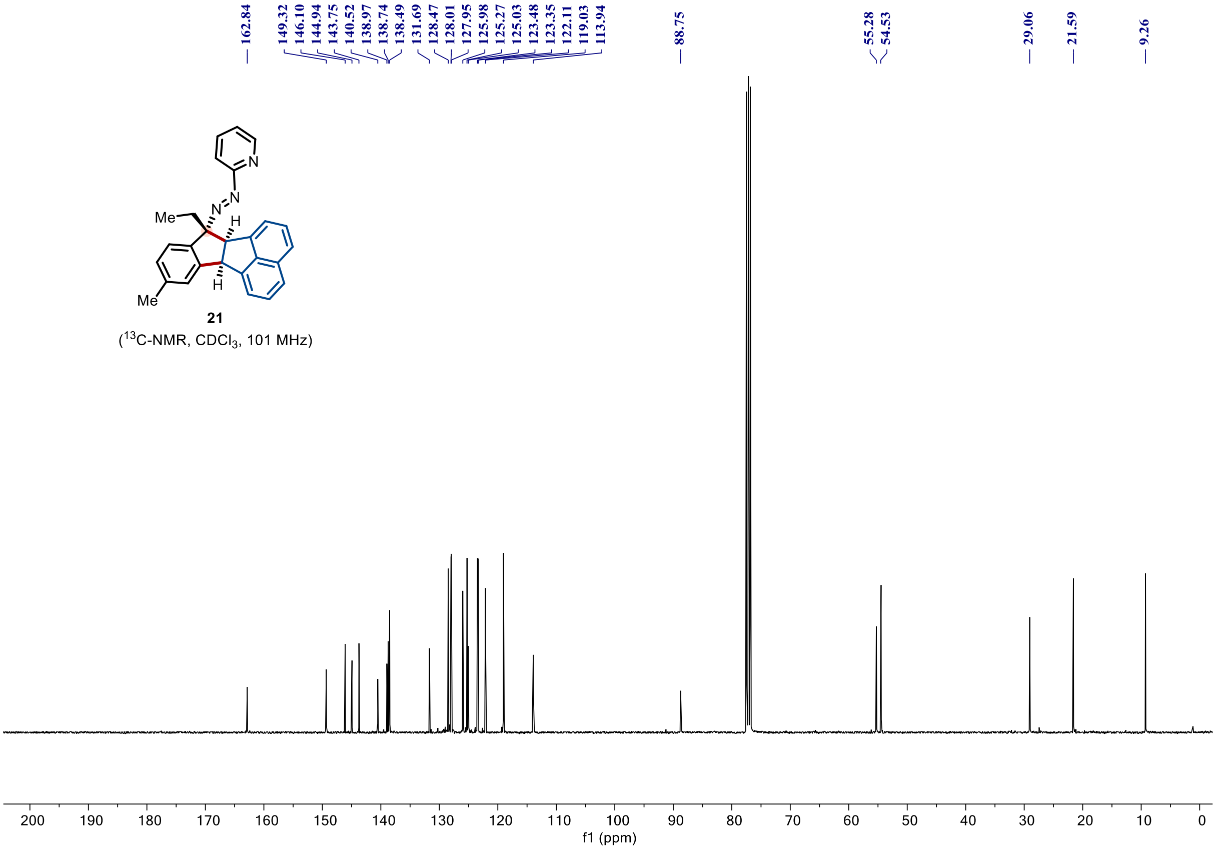
162.84
149.32
146.10
144.94
143.75
140.52
138.97
138.74
138.49
131.69
128.47
128.01
127.95
125.98
125.27
125.03
123.48
123.35
122.11
119.03
113.94
88.75
55.28
54.53
29.06
21.59
9.26
Me
N
N
N
H
H
Me
21
($^{13}$C-NMR, CDCl$_3$, 101 MHz)
f1 (ppm)

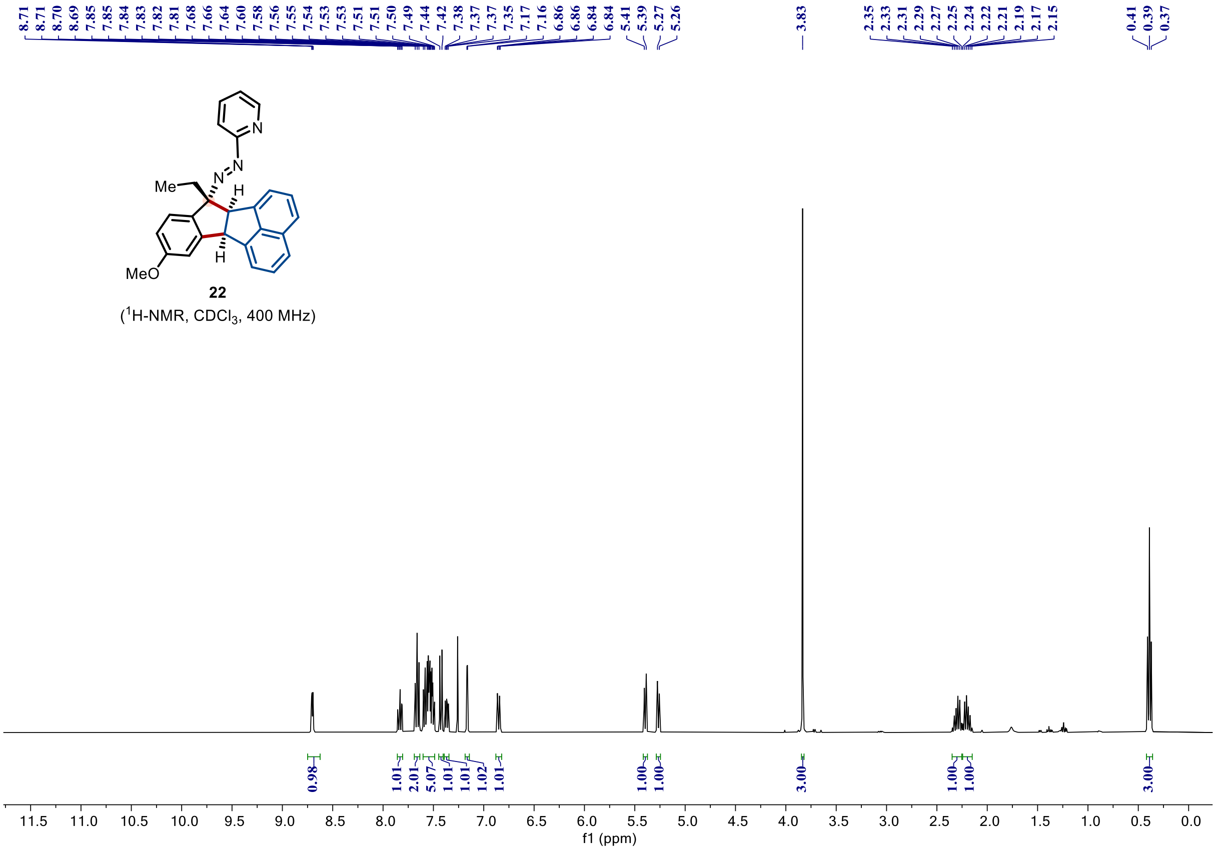

22

($^{1}$H-NMR, $CDCl_3$, 400 MHz)

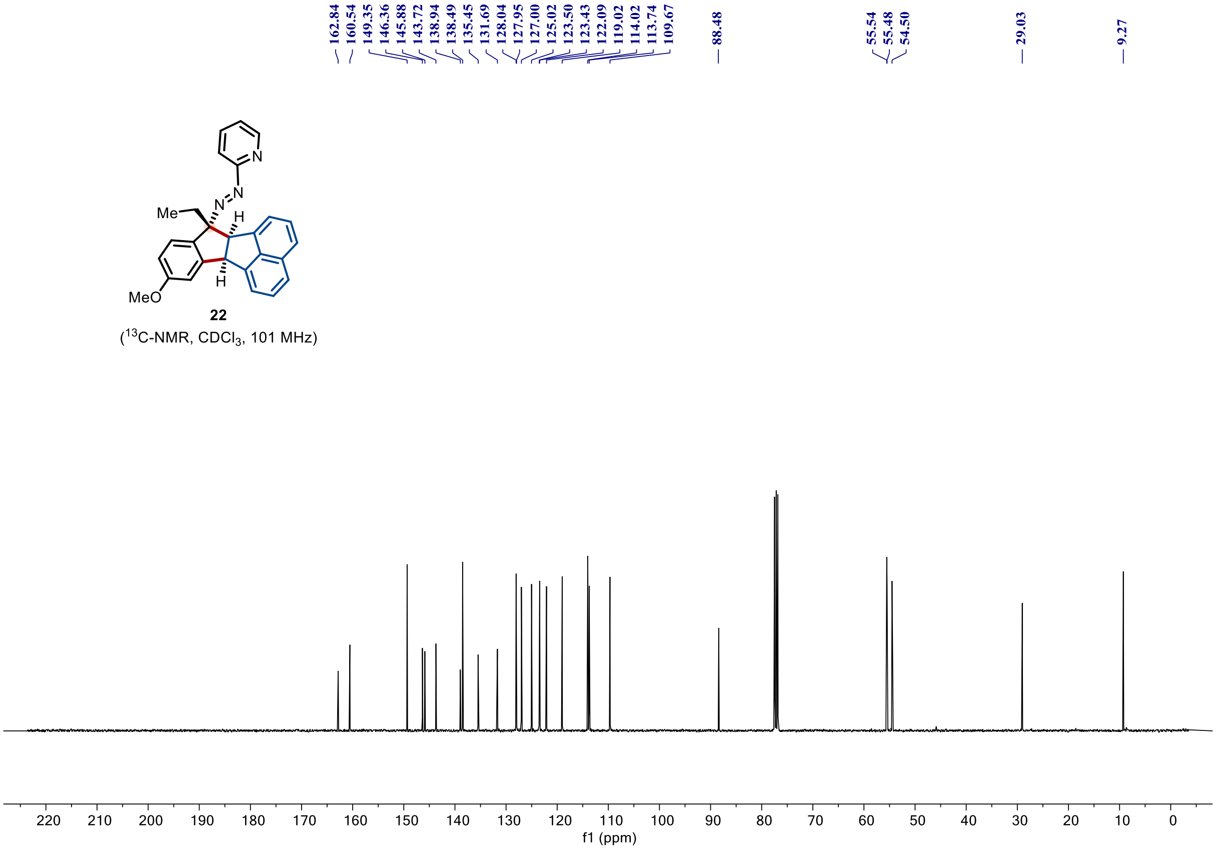

22

($^{13}$C-NMR, $CDCl_3$, 101 MHz)

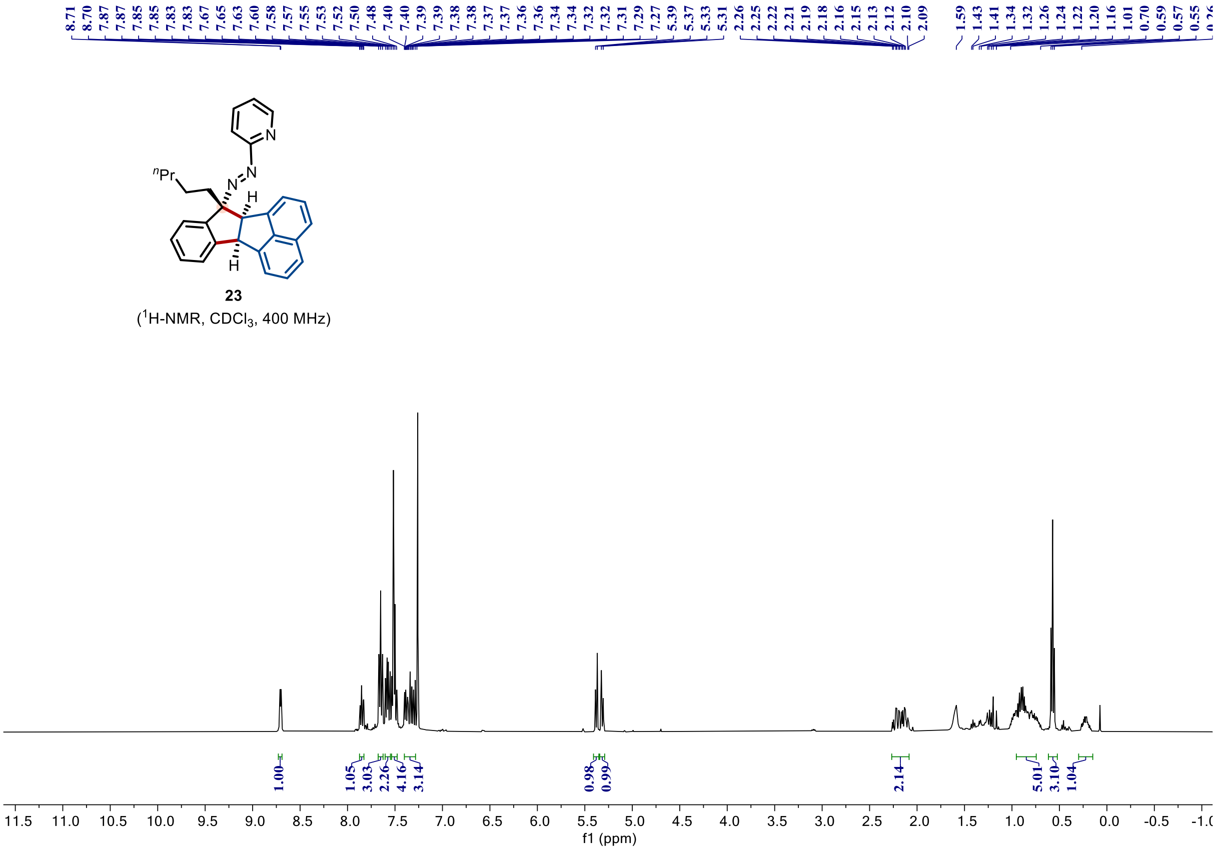
8.71 8.70 7.87 7.85 7.85 7.83 7.83 7.67 7.65 7.63 7.60 7.58 7.57 7.55 7.53 7.52 7.50 7.48 7.40 7.39 7.39 7.38 7.38 7.37 7.37 7.36 7.34 7.34 7.32 7.32 7.31 7.29 7.27 5.39 5.37 5.33 5.31 2.26 2.25 2.22 2.21 2.19 2.18 2.16 2.15 2.13 2.12 2.10 2.09 1.59 1.43 1.41 1.34 1.32 1.26 1.24 1.22 1.20 1.16 1.01 0.70 0.59 0.57 0.55
$^n$Pr
N
N=N
H
H
23
($^1$H-NMR, $CDCl_3$, 400 MHz)
1.00 1.05 3.03 2.26 4.16 3.14 0.98 0.99 2.14 5.01 3.10 1.04
11.5 11.0 10.5 10.0 9.5 9.0 8.5 8.0 7.5 7.0 6.5 6.0 5.5 5.0 4.5 4.0 3.5 3.0 2.5 2.0 1.5 1.0 0.5 0.0 -0.5
f1 (ppm)

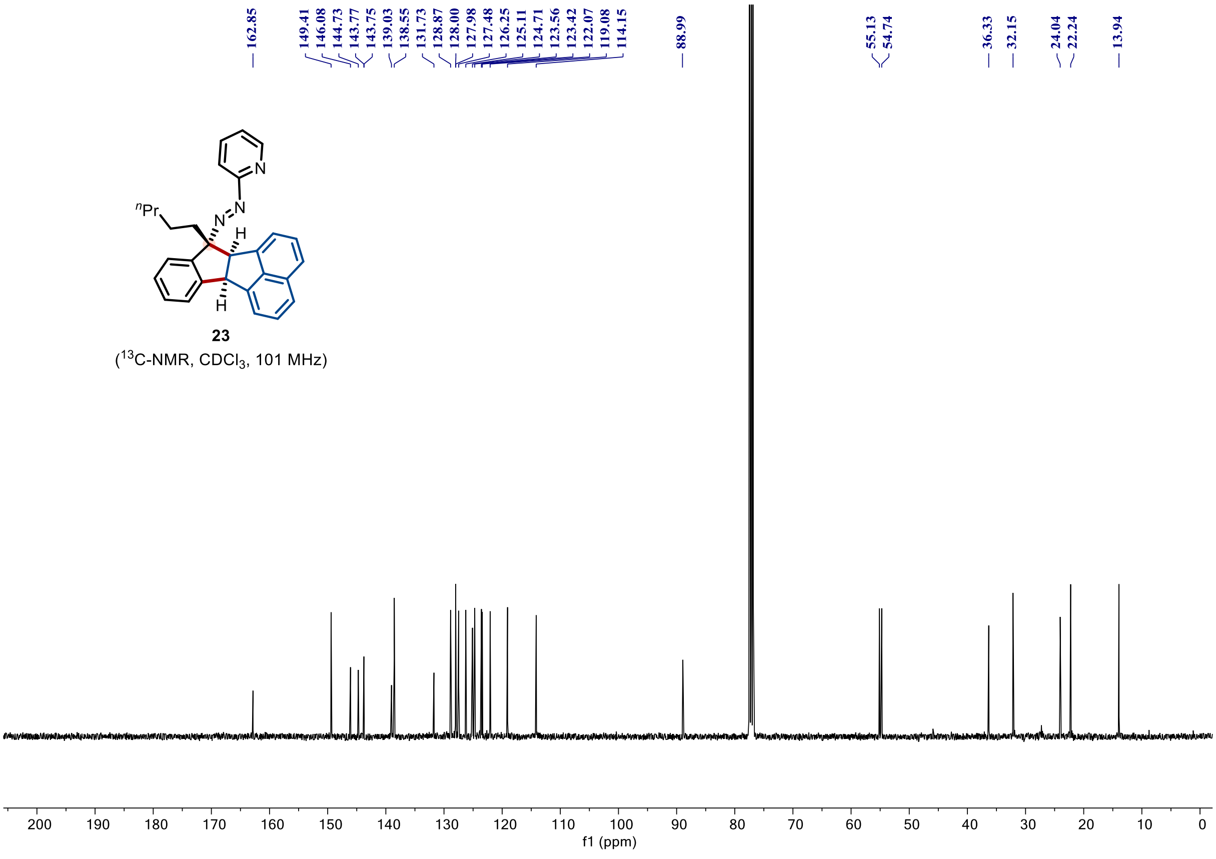
162.85 149.41 146.08 144.73 143.77 143.75 139.03 138.55 131.73 128.87 128.00 127.98 127.48 126.25 125.11 124.71 123.56 123.42 122.07 119.08 114.15 88.99 55.13 54.74 36.33 32.15 24.04 22.24 13.94
$^n$Pr
N
N=N
H
H
23
($^{13}$C-NMR, $CDCl_3$, 101 MHz)
200 190 180 170 160 150 140 130 120 110 100 90 80 70 60 50 40 30 20 10 0
f1 (ppm)

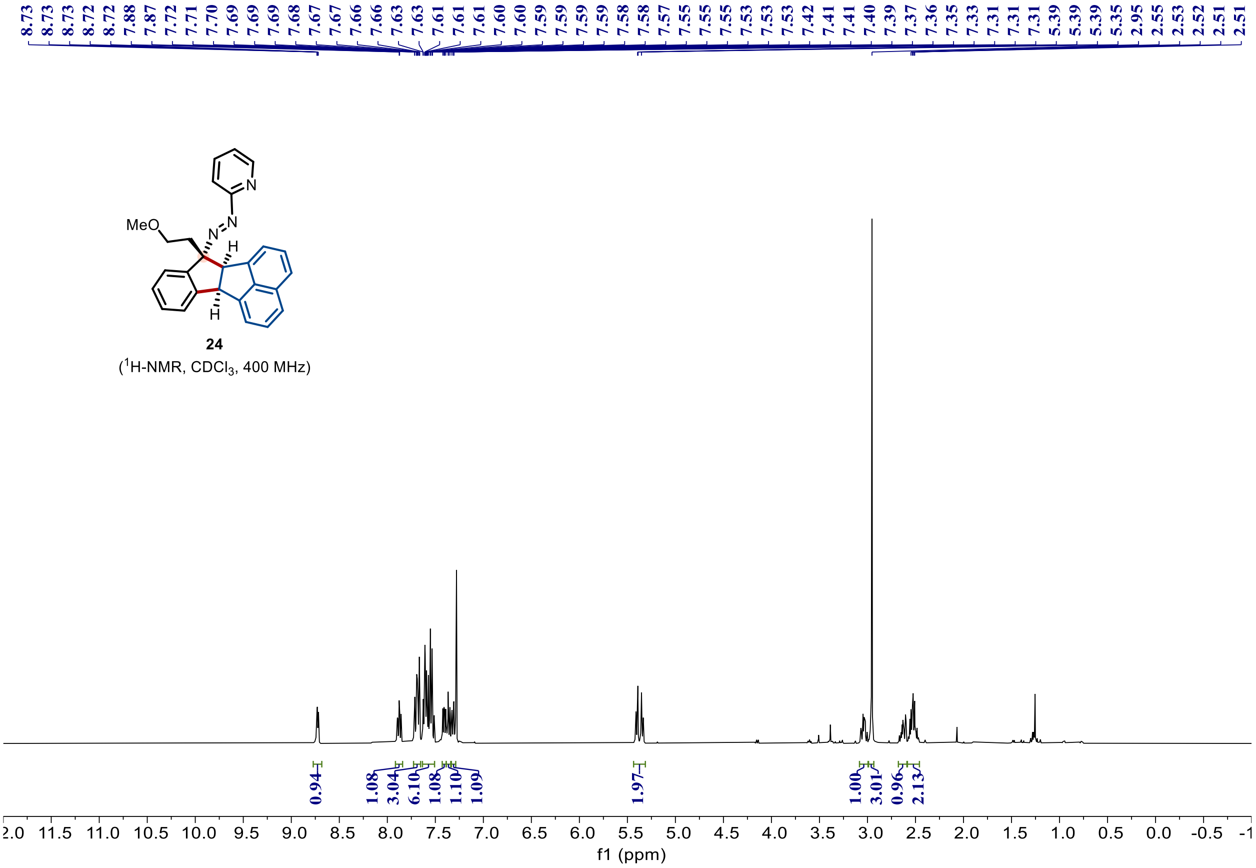


**24**
($^1$H-NMR, $CDCl_3$, 400 MHz)

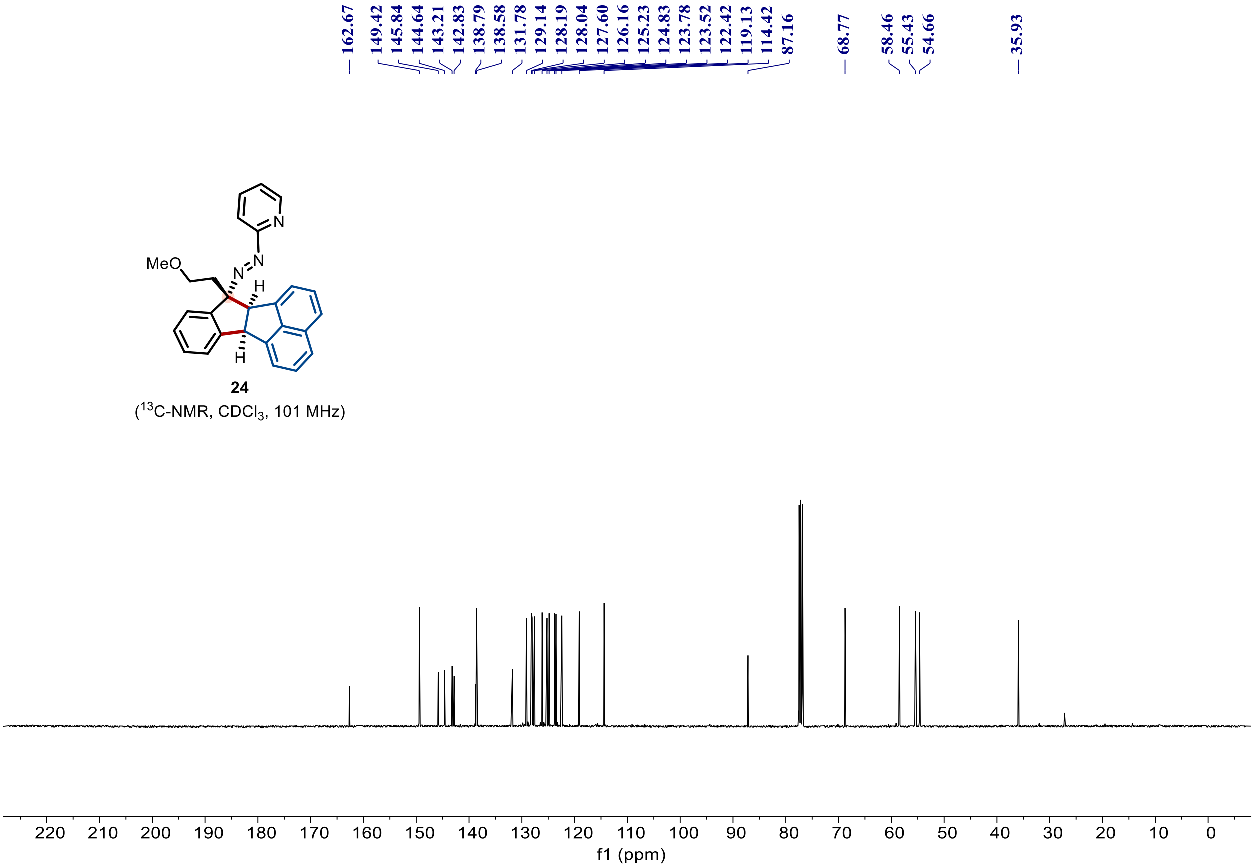


**24**
($^{13}$C-NMR, $CDCl_3$, 101 MHz)

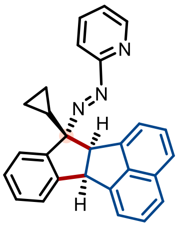

**25**

($^{1}$H-NMR, $CDCl_3$, 400 MHz)

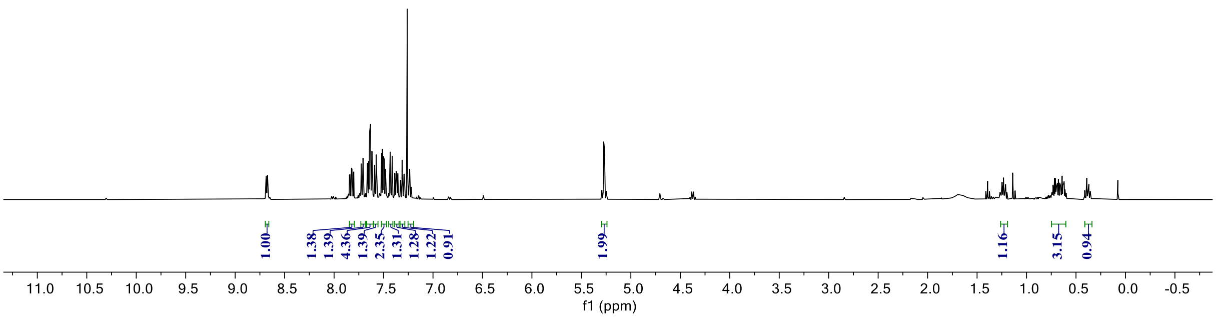


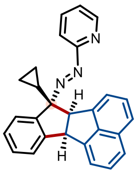

**25**

($^{13}$C-NMR, $CDCl_3$, 101 MHz)

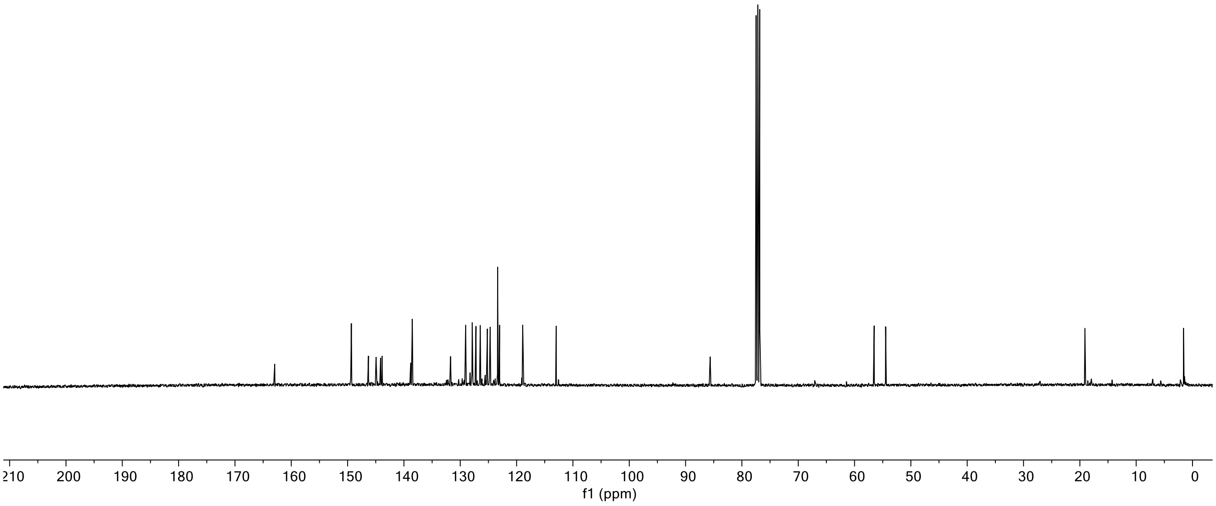

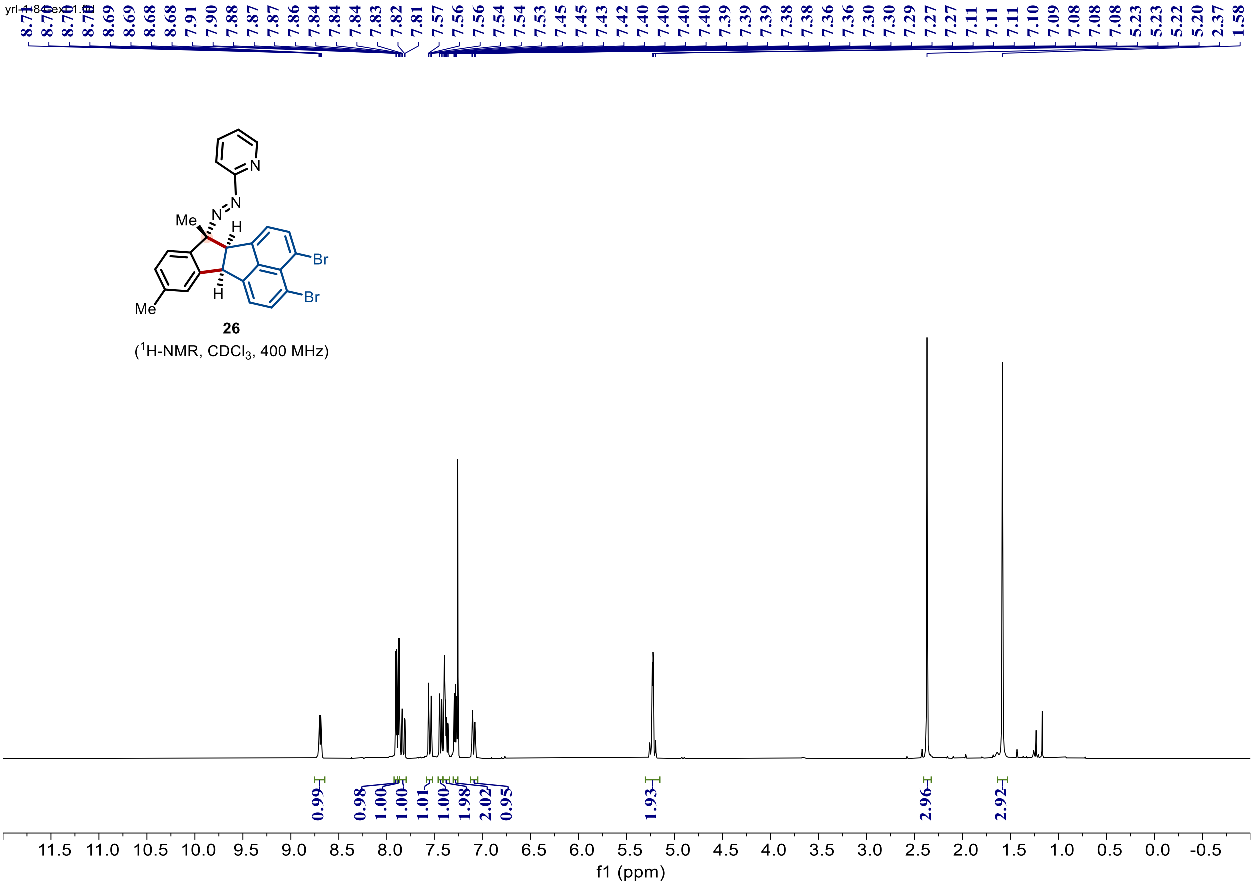


**26**

($^1$H-NMR, $CDCl_3$, 400 MHz)

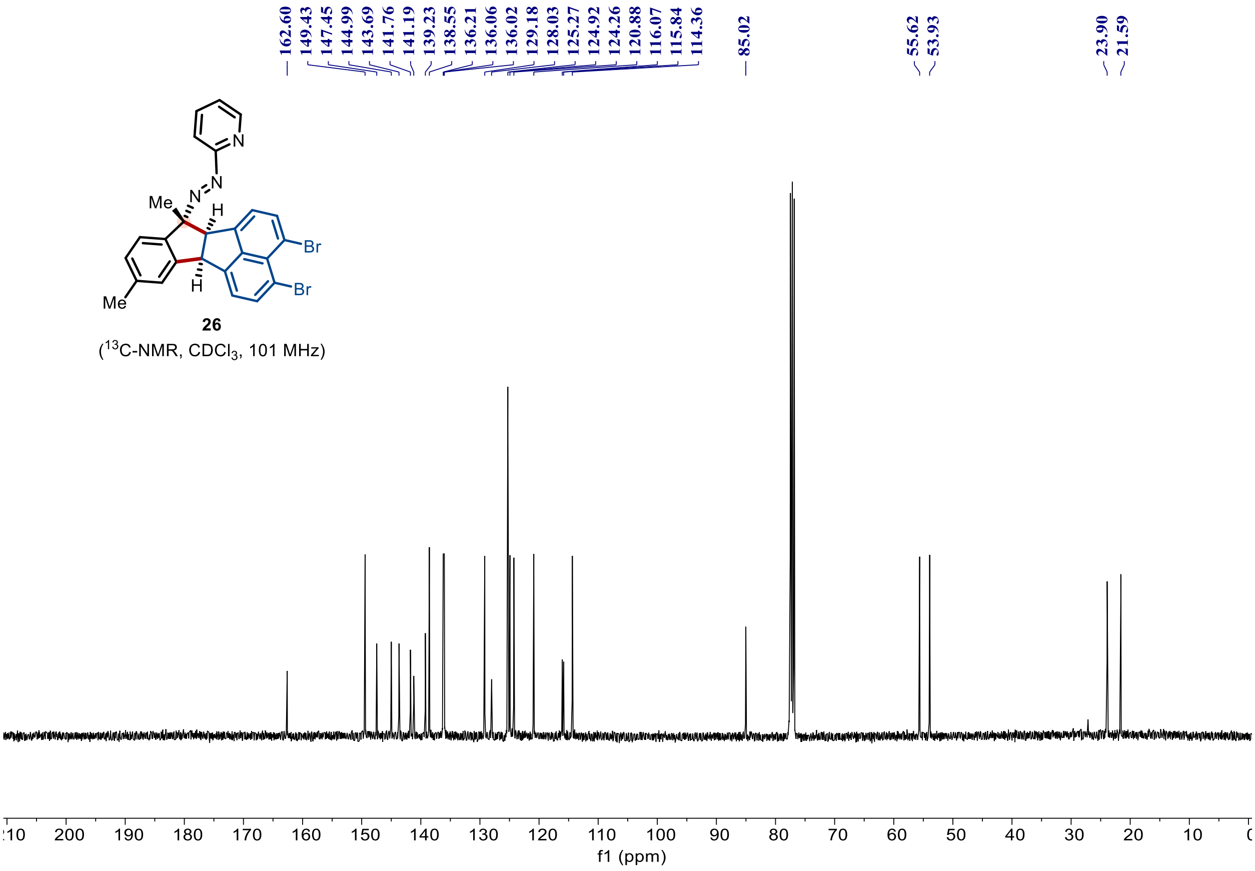


**26**

($^{13}$C-NMR, $CDCl_3$, 101 MHz)

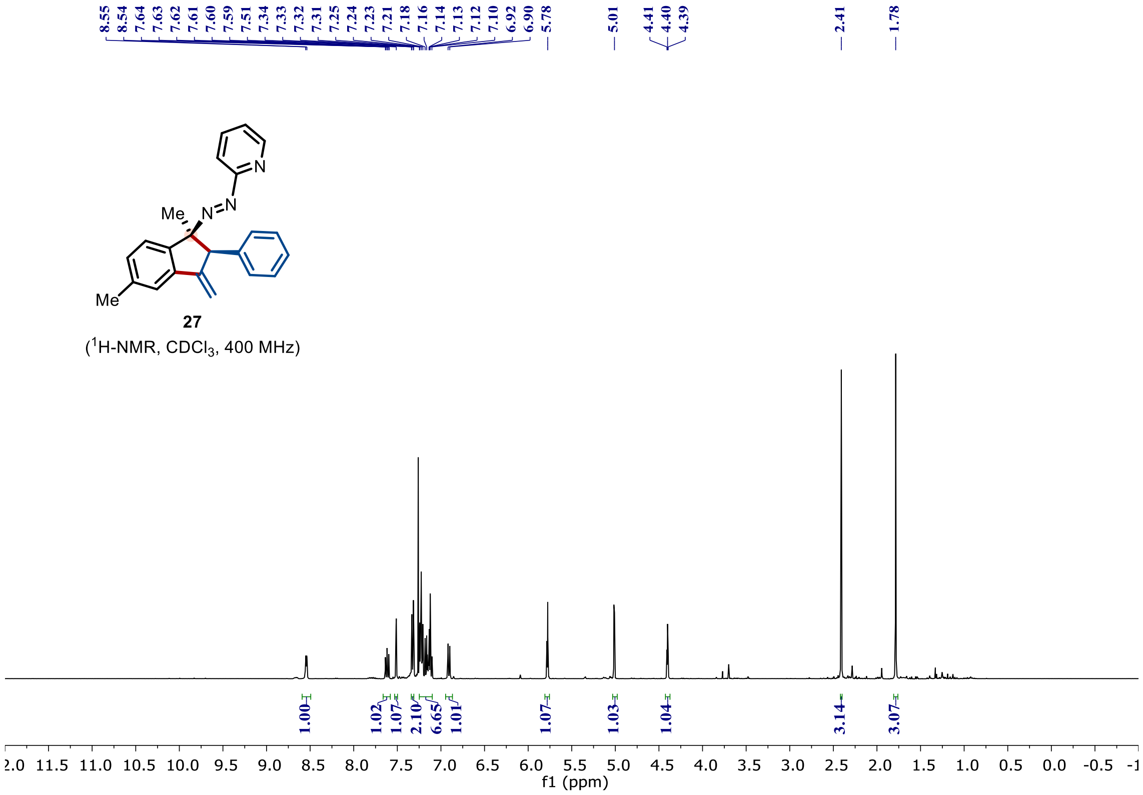
8.55 8.54 7.64 7.63 7.62 7.61 7.60 7.59 7.51 7.34 7.33 7.32 7.31 7.25 7.24 7.23 7.21 7.18 7.16 7.14 7.13 7.12 7.10 6.92 6.90 5.78 5.01 4.41 4.40 4.39 2.41 1.78
Me
N
N=N
Me
27
($^1$H-NMR, $CDCl_3$, 400 MHz)
1.00 1.02 1.07 2.10 6.65 1.01 1.07 1.03 1.04 3.14 3.07
12.0 11.5 11.0 10.5 10.0 9.5 9.0 8.5 8.0 7.5 7.0 6.5 6.0 5.5 5.0 4.5 4.0 3.5 3.0 2.5 2.0 1.5 1.0 0.5 0.0 -0.5 -1
f1 (ppm)

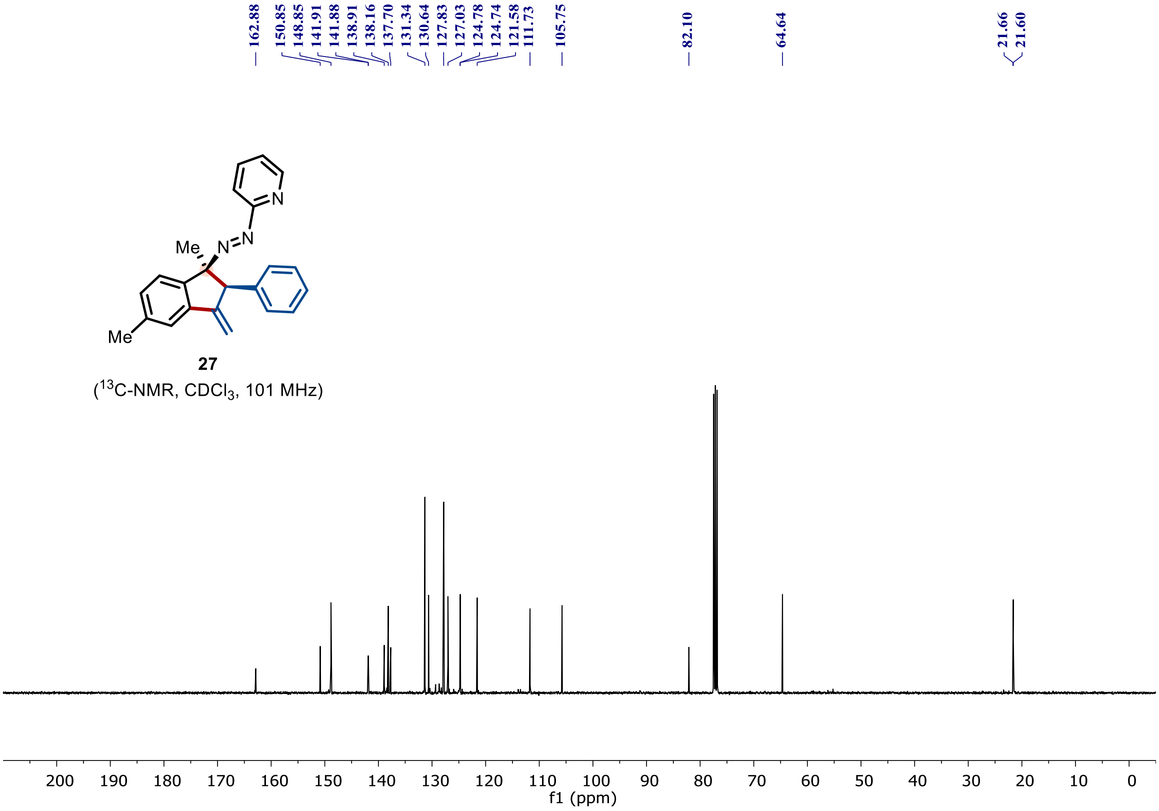
162.88 150.85 148.85 141.91 141.88 138.91 138.16 137.70 131.34 130.64 127.83 127.03 124.78 124.74 121.58 111.73 105.75 82.10 64.64 21.66 21.60
Me
N
N=N
Me
27
($^{13}$C-NMR, $CDCl_3$, 101 MHz)
200 190 180 170 160 150 140 130 120 110 100 90 80 70 60 50 40 30 20 10 0
f1 (ppm)

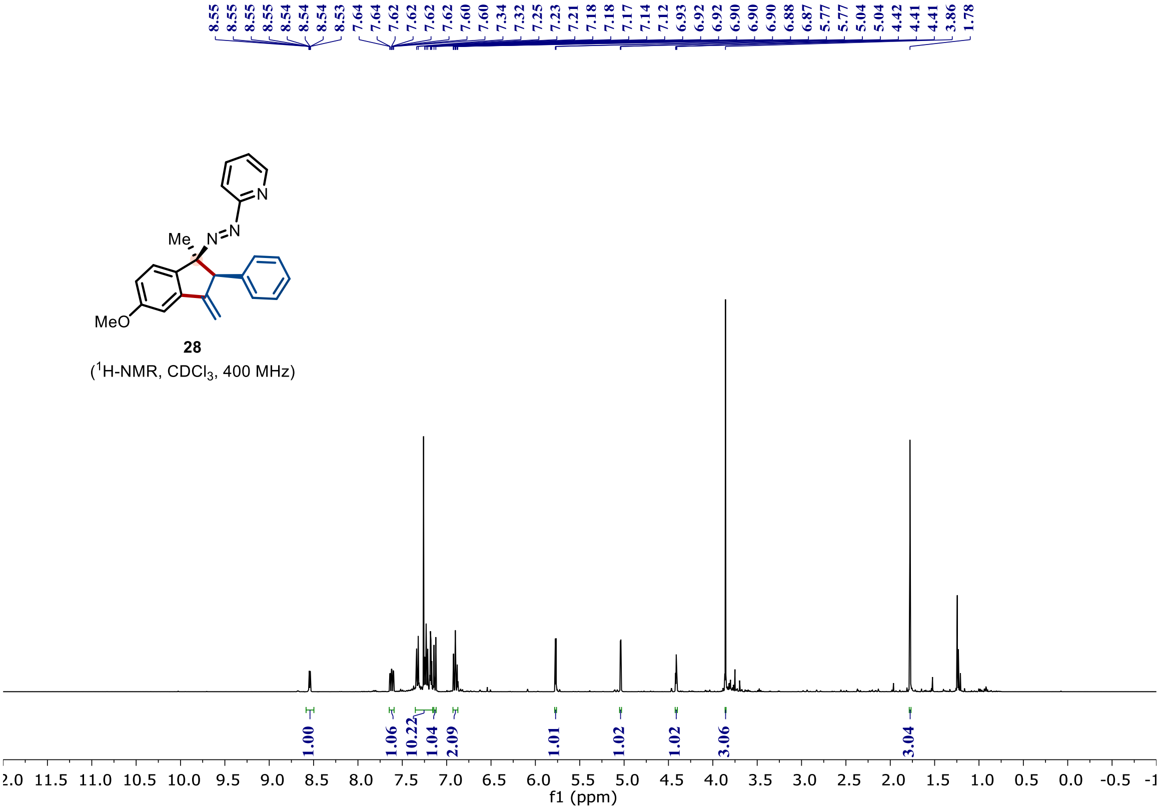
8.55 8.55 8.55 8.55 8.54 8.54 8.54 8.53 7.64 7.64 7.62 7.62 7.62 7.62 7.60 7.60 7.34 7.32 7.25 7.23 7.21 7.18 7.18 7.17 7.14 7.12 6.93 6.92 6.92 6.90 6.90 6.90 6.88 6.87 5.77 5.77 5.04 5.04 4.42 4.41 4.41 3.86 1.78
Me
N
N=N
MeO
28
($^{1}$H-NMR, $CDCl_3$, 400 MHz)
1.00 1.06 10.22 1.04 2.09 1.01 1.02 1.02 3.06 3.04
f1 (ppm)

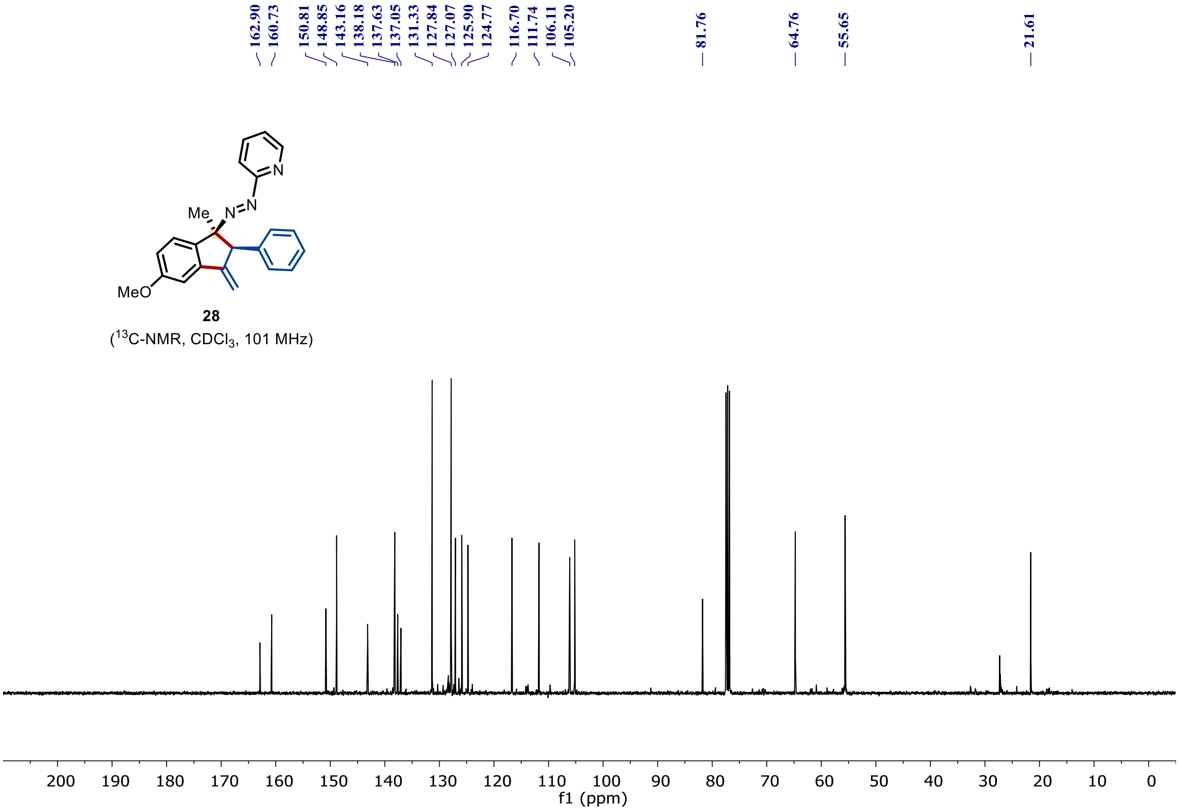
162.90 160.73 150.81 148.85 143.16 138.18 137.63 137.05 131.33 127.84 127.07 125.90 124.77 116.70 111.74 106.11 105.20 81.76 64.76 55.65 21.61
Me
N
N=N
MeO
28
($^{13}$C-NMR, $CDCl_3$, 101 MHz)
f1 (ppm)

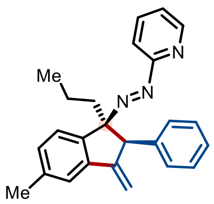


**29**

($^1$H-NMR, $CDCl_3$, 400 MHz)

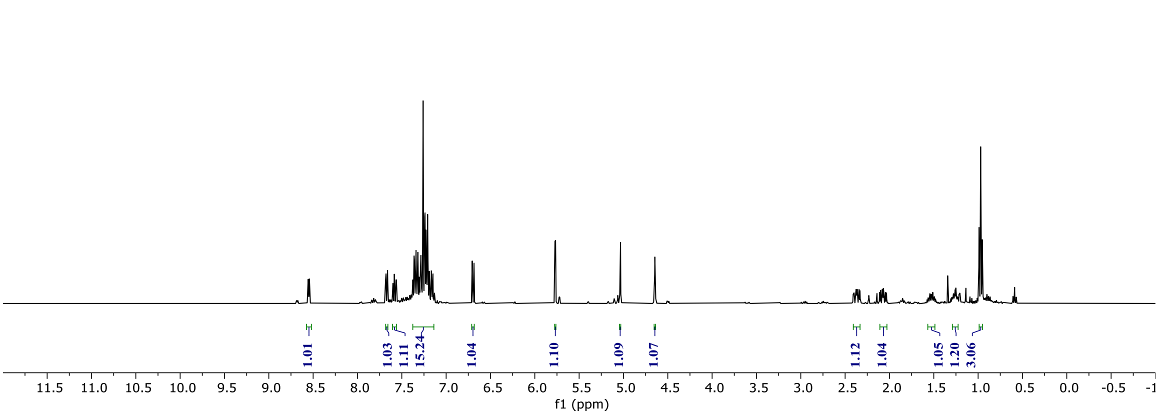


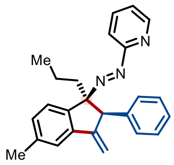


**29**

($^{13}$C-NMR, $CDCl_3$, 101 MHz)

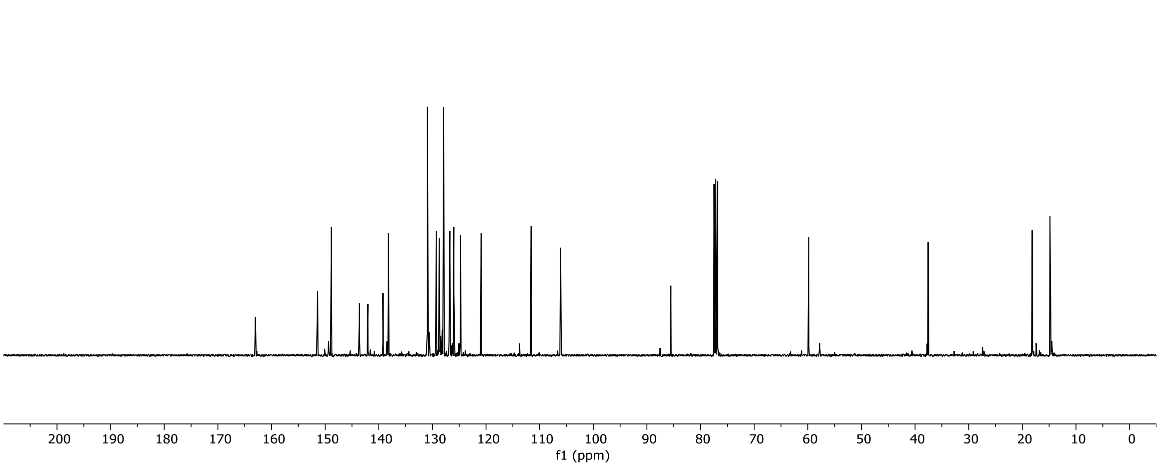

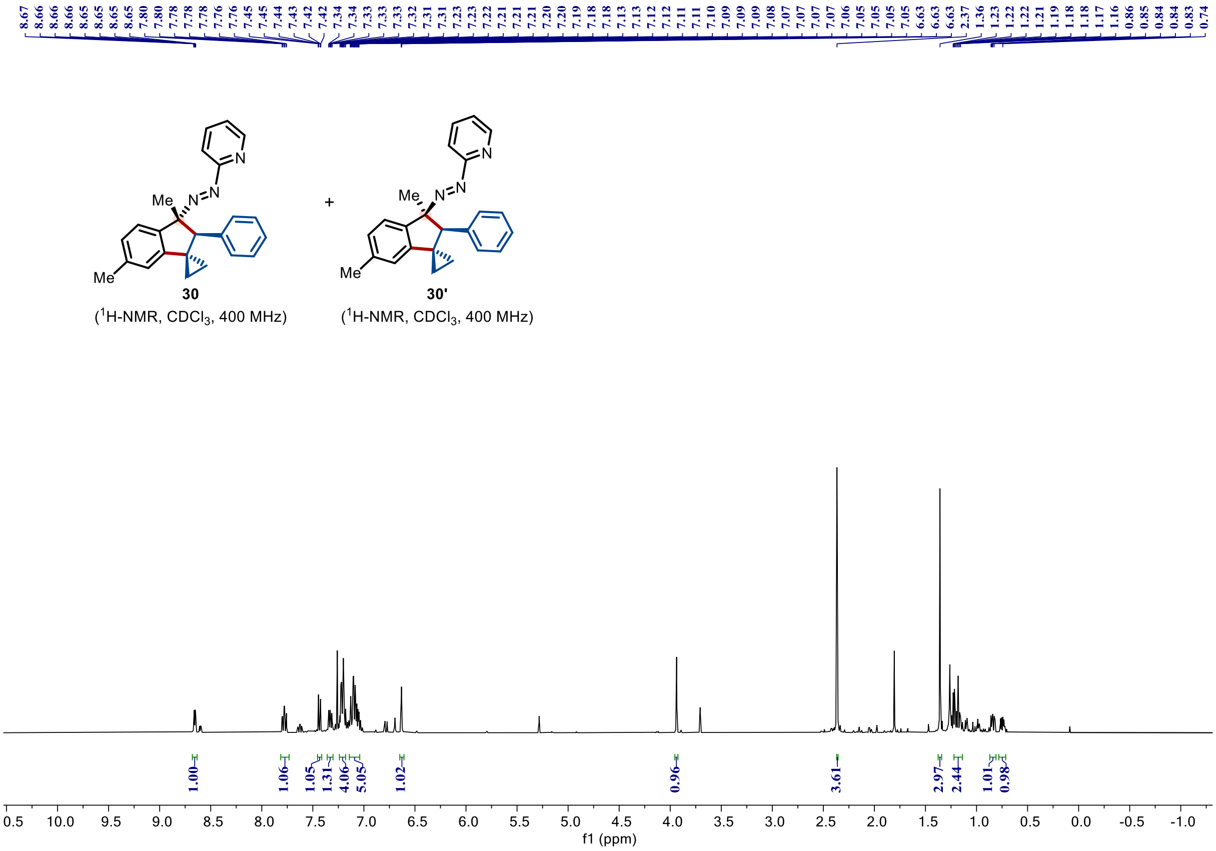
8.67 8.66 8.66 8.66 8.65 8.65 8.65 8.65 7.80 7.80 7.78 7.78 7.78 7.76 7.76 7.45 7.45 7.44 7.43 7.42 7.42 7.34 7.34 7.33 7.33 7.33 7.32 7.31 7.31 7.23 7.23 7.22 7.21 7.21 7.21 7.20 7.20 7.19 7.18 7.18 7.13 7.13 7.12 7.12 7.11 7.11 7.10 7.09 7.09 7.09 7.08 7.07 7.07 7.07 7.07 7.06 7.05 7.05 7.05 7.05 6.63 6.63 6.63 2.37 1.36 1.23 1.22 1.22 1.21 1.19 1.18 1.18 1.17 1.16 0.86 0.85 0.84 0.84 0.83 0.74
Me
N=N
N
Me
+
30
30'
($^{1}$H-NMR, $CDCl_3$, 400 MHz)
($^{1}$H-NMR, $CDCl_3$, 400 MHz)
1.00
1.06
1.05
1.31
4.06
5.05
1.02
0.96
3.61
2.97
2.44
1.01
0.98
0.5 10.0 9.5 9.0 8.5 8.0 7.5 7.0 6.5 6.0 5.5 5.0 4.5 4.0 3.5 3.0 2.5 2.0 1.5 1.0 0.5 0.0 -0.5 -1.0
f1 (ppm)

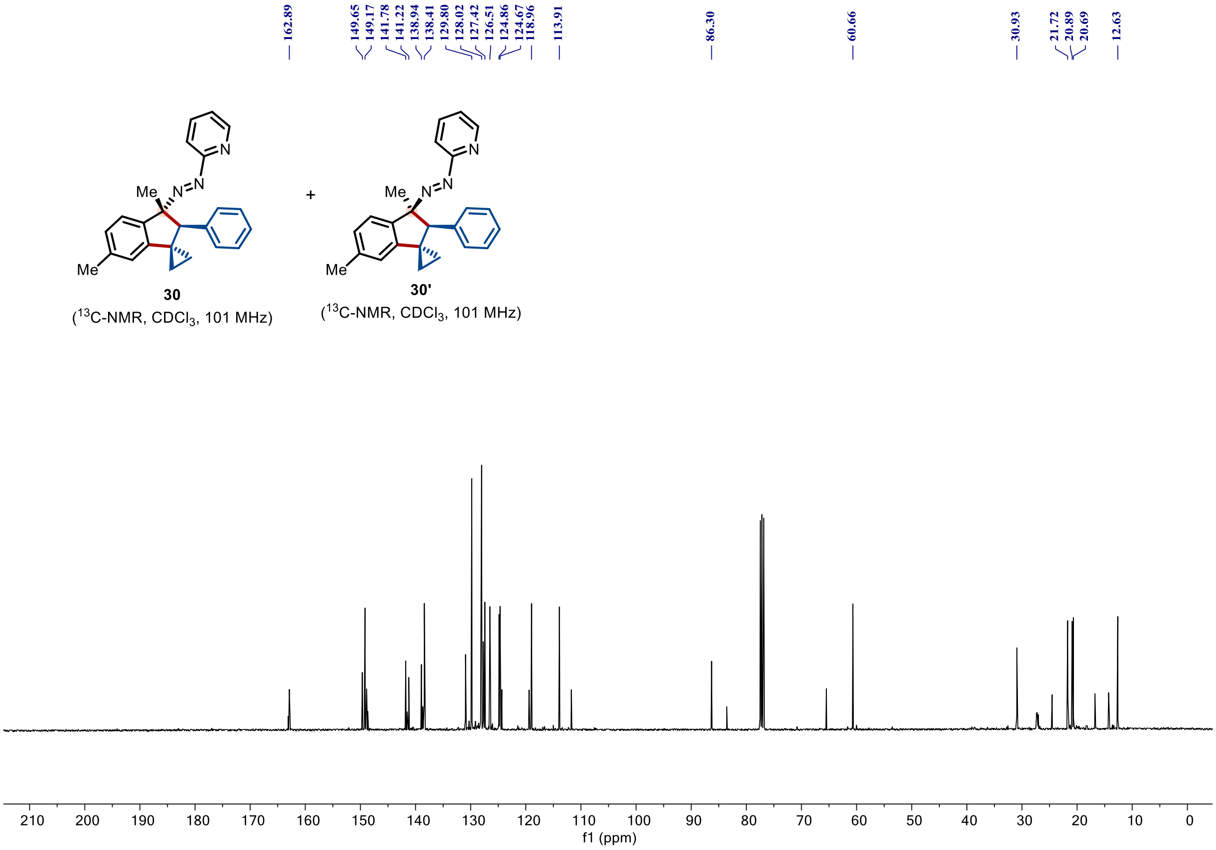
162.89
149.65 149.17 141.78 141.22 138.94 138.41 129.80 128.02 127.42 126.51 124.86 124.67 118.96
113.91
86.30
60.66
30.93
21.72 20.89 20.69
12.63
Me
N=N
N
Me
+
30
30'
($^{13}$C-NMR, $CDCl_3$, 101 MHz)
($^{13}$C-NMR, $CDCl_3$, 101 MHz)
210 200 190 180 170 160 150 140 130 120 110 100 90 80 70 60 50 40 30 20 10 0
f1 (ppm)

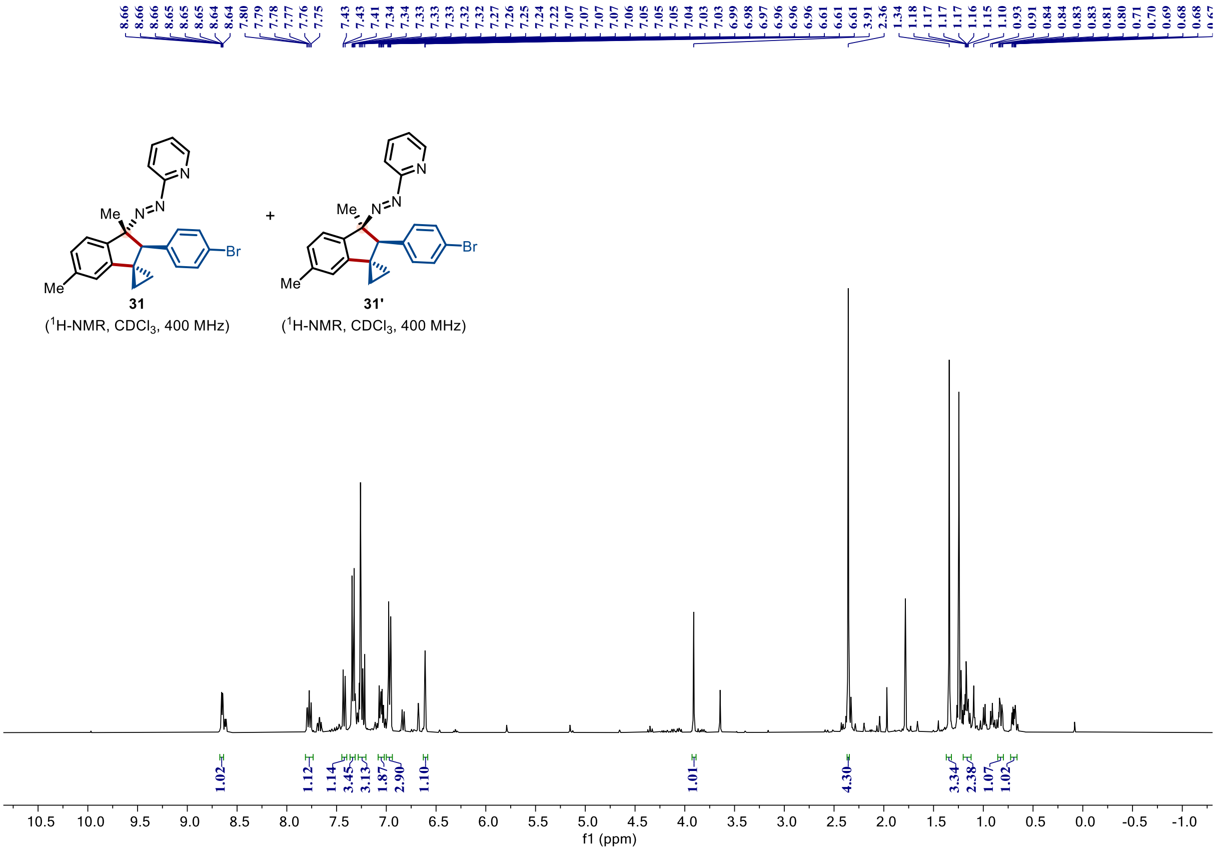
8.66 8.66 8.66 8.65 8.65 8.65 8.64 7.80 7.79 7.78 7.77 7.76 7.75 7.43 7.43 7.41 7.34 7.34 7.33 7.33 7.32 7.27 7.26 7.25 7.24 7.22 7.07 7.07 7.07 7.06 7.05 7.05 7.05 7.04 7.03 7.03 6.99 6.98 6.97 6.96 6.96 6.61 6.61 6.61 3.91 2.36 1.34 1.18 1.17 1.17 1.16 1.15 1.10 0.93 0.91 0.84 0.84 0.83 0.83 0.81 0.80 0.71 0.70 0.69 0.68 0.68
Me
N=N
N
Br
31
($^1$H-NMR, $CDCl_3$, 400 MHz)
+
31'
($^1$H-NMR, $CDCl_3$, 400 MHz)
1.02 1.12 1.14 3.45 3.13 1.87 2.90 1.10 1.01 4.30 3.34 2.38 1.07 1.02
10.5 10.0 9.5 9.0 8.5 8.0 7.5 7.0 6.5 6.0 5.5 5.0 4.5 4.0 3.5 3.0 2.5 2.0 1.5 1.0 0.5 0.0 -0.5 -1.0
f1 (ppm)

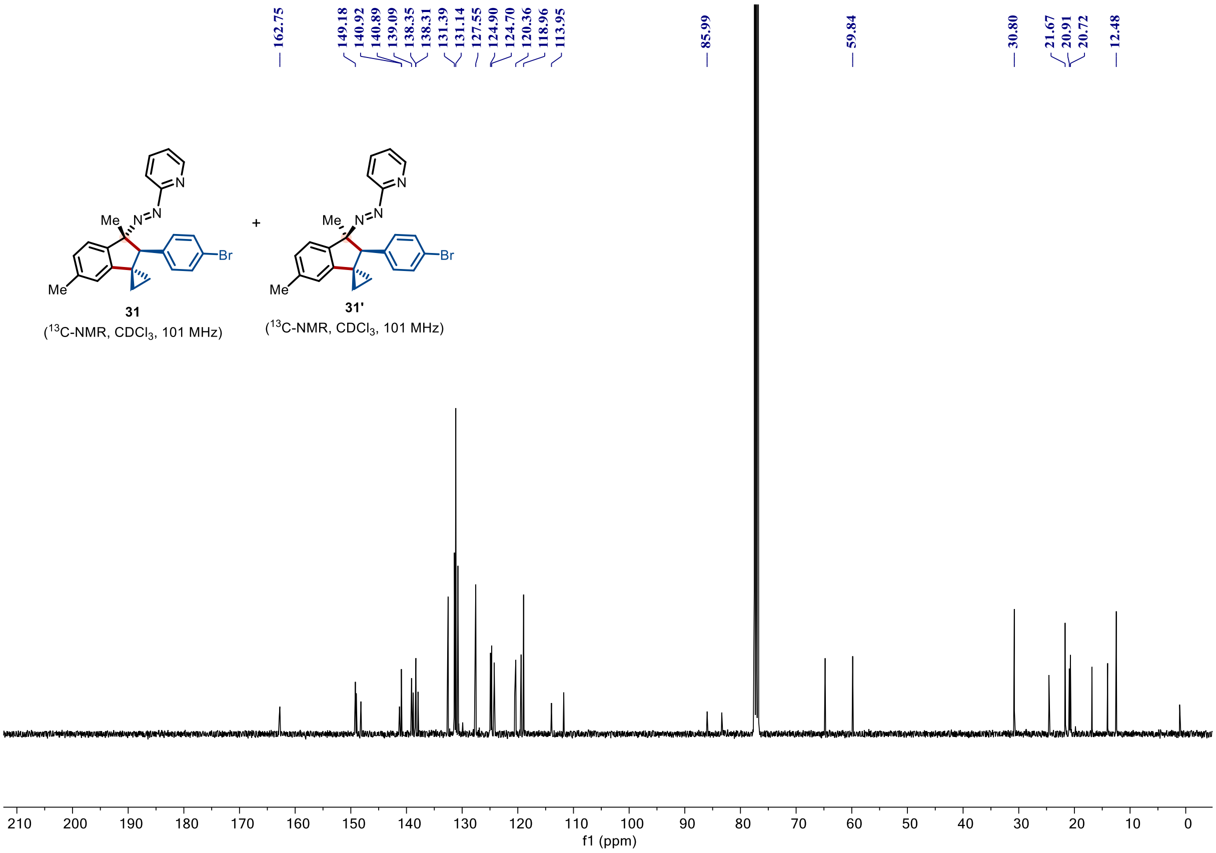
162.75
149.18 140.92 140.89 139.09 138.35 138.31 131.39 131.14 127.55 124.90 124.70 120.36 118.96 113.95
85.99
59.84
30.80
21.67 20.91 20.72
12.48
Me
N=N
N
Br
31
($^{13}$C-NMR, $CDCl_3$, 101 MHz)
+
31'
($^{13}$C-NMR, $CDCl_3$, 101 MHz)
210 200 190 180 170 160 150 140 130 120 110 100 90 80 70 60 50 40 30 20 10 0
f1 (ppm)

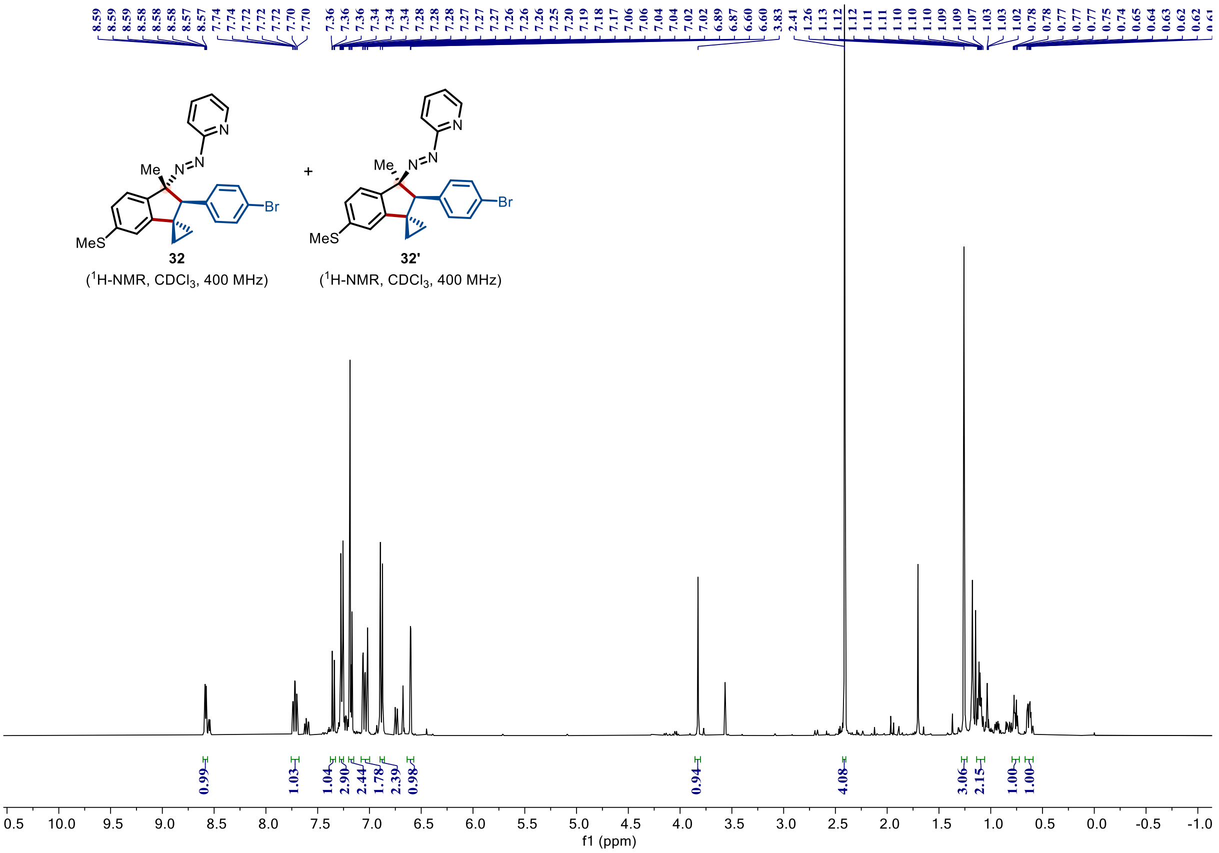
32
(1H-NMR, CDCl3, 400 MHz)
32'
(1H-NMR, CDCl3, 400 MHz)
f1 (ppm)

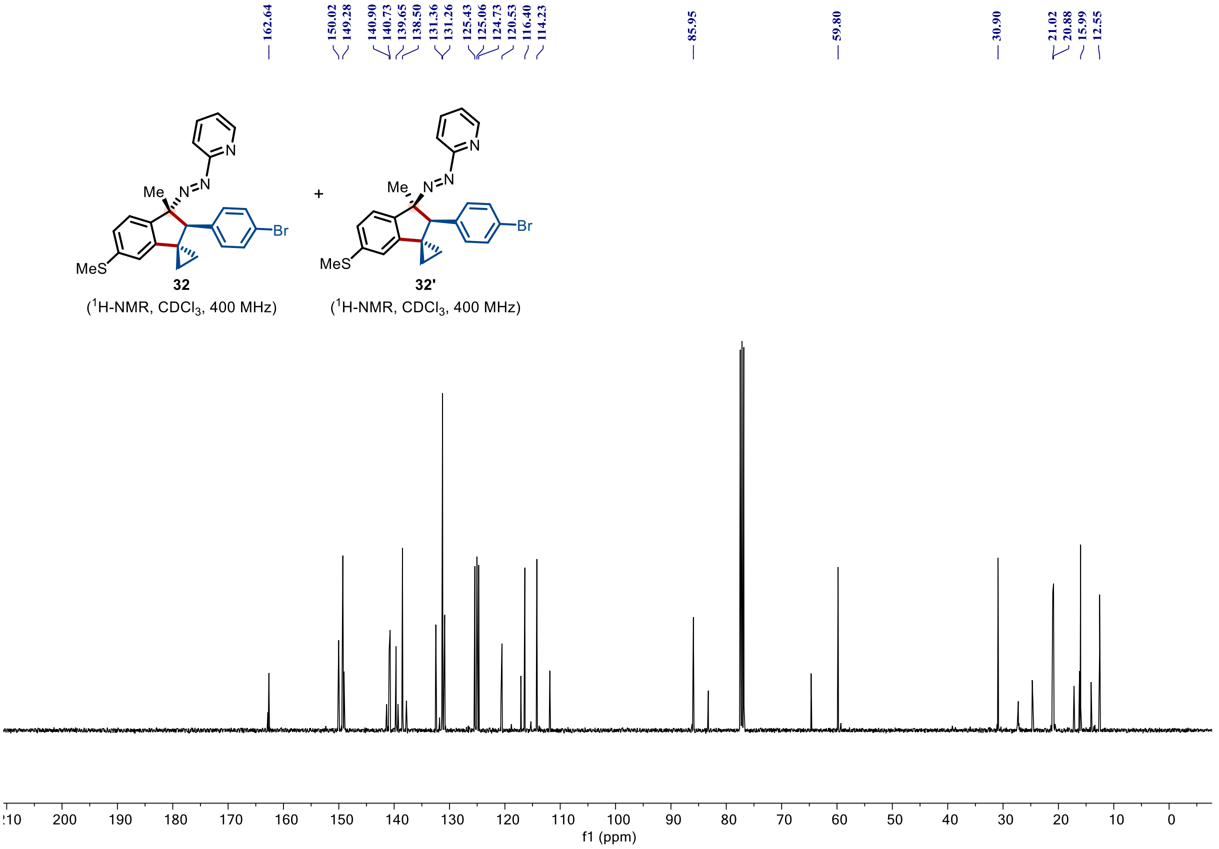
32
(1H-NMR, CDCl3, 400 MHz)
32'
(1H-NMR, CDCl3, 400 MHz)
f1 (ppm)

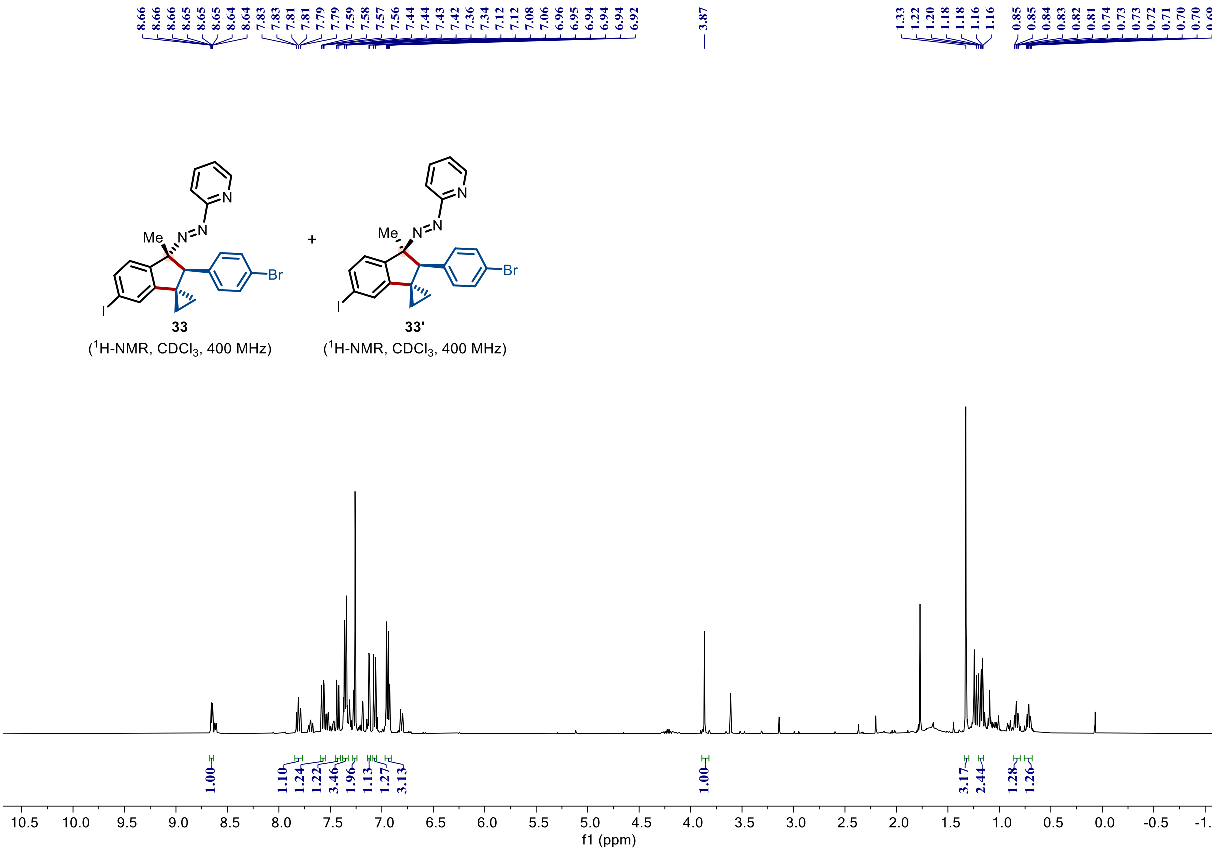
8.66 8.66 8.66 8.65 8.65 8.65 8.64 8.64 7.83 7.83 7.81 7.81 7.79 7.79 7.59 7.58 7.57 7.56 7.44 7.44 7.43 7.42 7.36 7.34 7.12 7.12 7.08 7.06 6.96 6.95 6.94 6.94 6.94 6.92
3.87
1.33 1.22 1.20 1.18 1.18 1.16 1.16 0.85 0.85 0.84 0.83 0.82 0.81 0.74 0.73 0.72 0.71 0.70 0.70
Me
N=N
N
Br
I
+
33
(1H-NMR, CDCl3, 400 MHz)
33'
(1H-NMR, CDCl3, 400 MHz)
1.00 1.10 1.24 1.22 3.46 1.96 1.13 1.27 3.13 1.00 3.17 2.44 1.28 1.26
f1 (ppm)

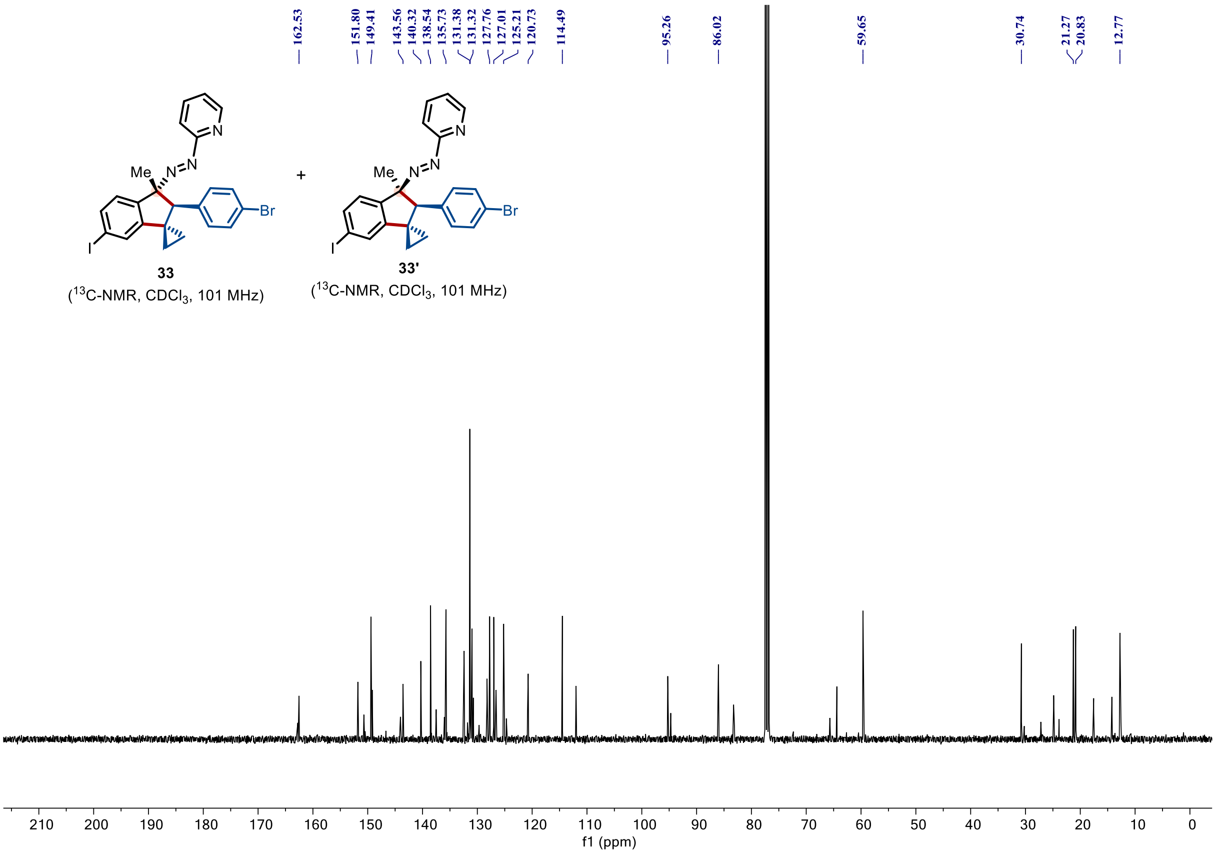
162.53 151.80 149.41 143.56 140.32 138.54 135.73 131.38 131.32 127.76 127.01 125.21 120.73 114.49 95.26 86.02 59.65 30.74 21.27 20.83 12.77
Me
N=N
N
Br
I
+
33
(13C-NMR, CDCl3, 101 MHz)
33'
(13C-NMR, CDCl3, 101 MHz)
f1 (ppm)

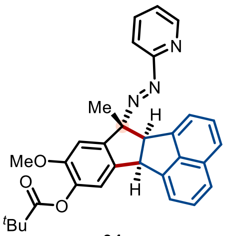

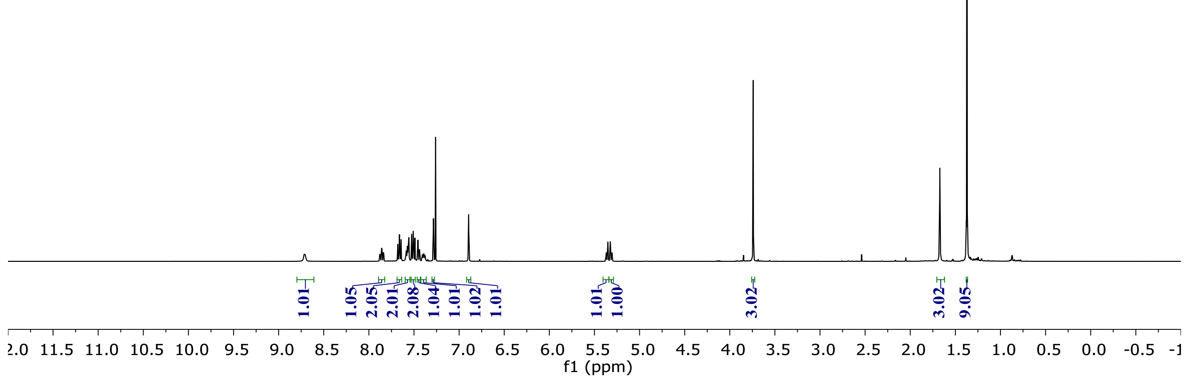


**34**
($^{1}$H-NMR, $CDCl_3$, 400 MHz)

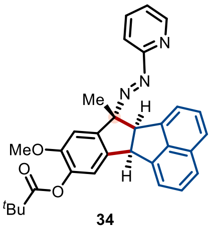

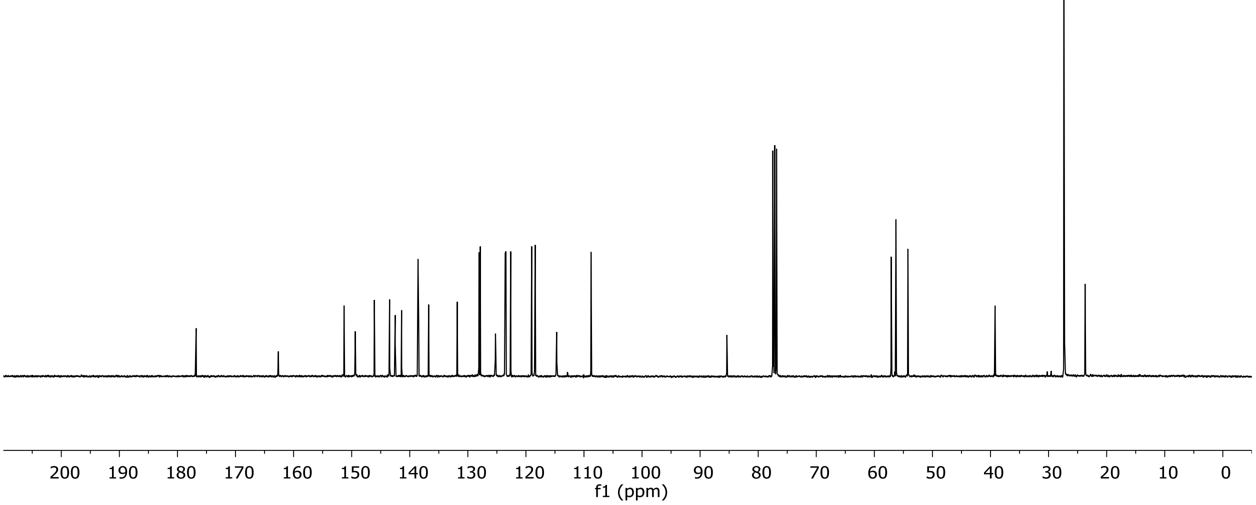


**34**
($^{13}$C-NMR, $CDCl_3$, 101 MHz)

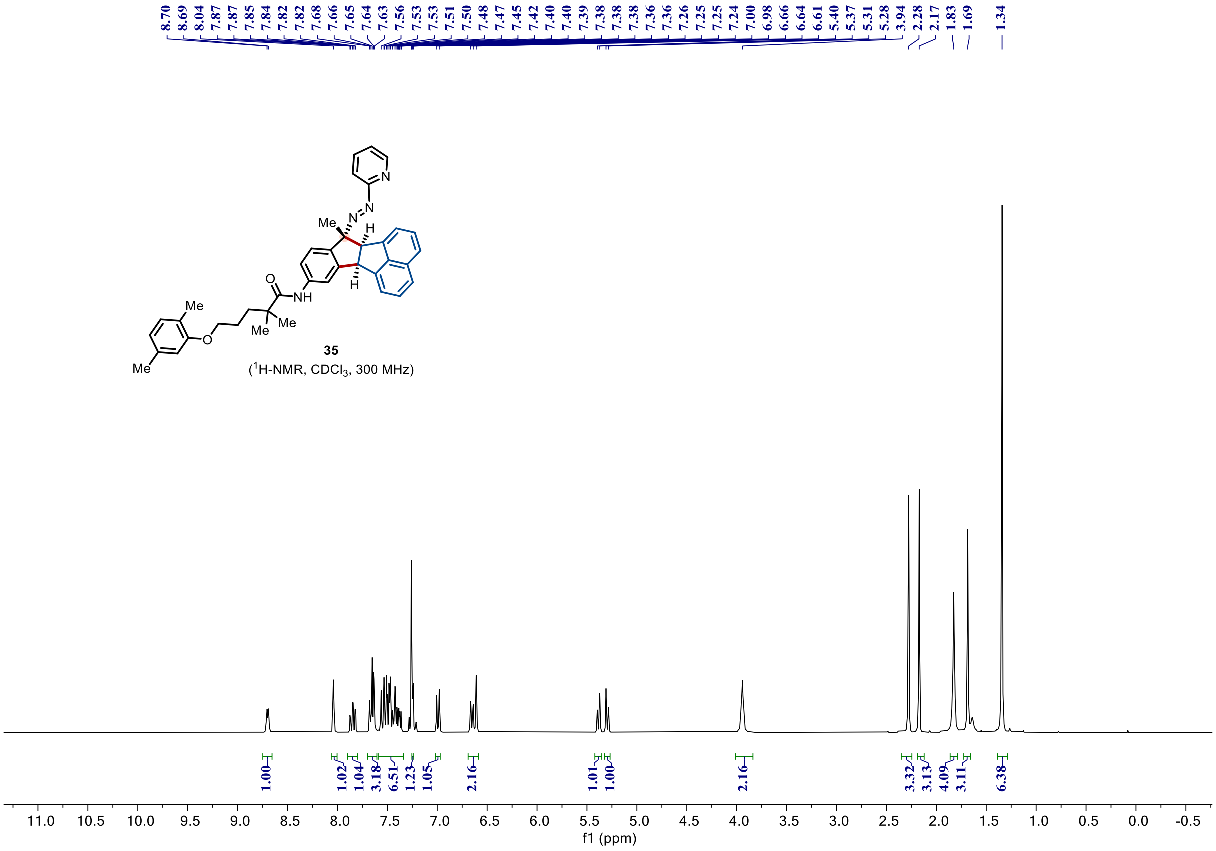
8.70 8.69 8.04 7.87 7.85 7.84 7.82 7.82 7.68 7.66 7.65 7.64 7.63 7.56 7.53 7.53 7.51 7.50 7.48 7.47 7.45 7.42 7.40 7.40 7.39 7.38 7.38 7.38 7.36 7.36 7.26 7.25 7.25 7.24 7.00 6.98 6.66 6.64 6.61 5.40 5.37 5.31 5.28 3.94 2.28 2.17 1.83 1.69 1.34
35
(1H-NMR, CDCl3, 300 MHz)
1.00 1.02 1.04 3.18 6.51 1.23 1.05 2.16 1.01 1.00 2.16 3.32 3.13 4.09 3.11 6.38
f1 (ppm)

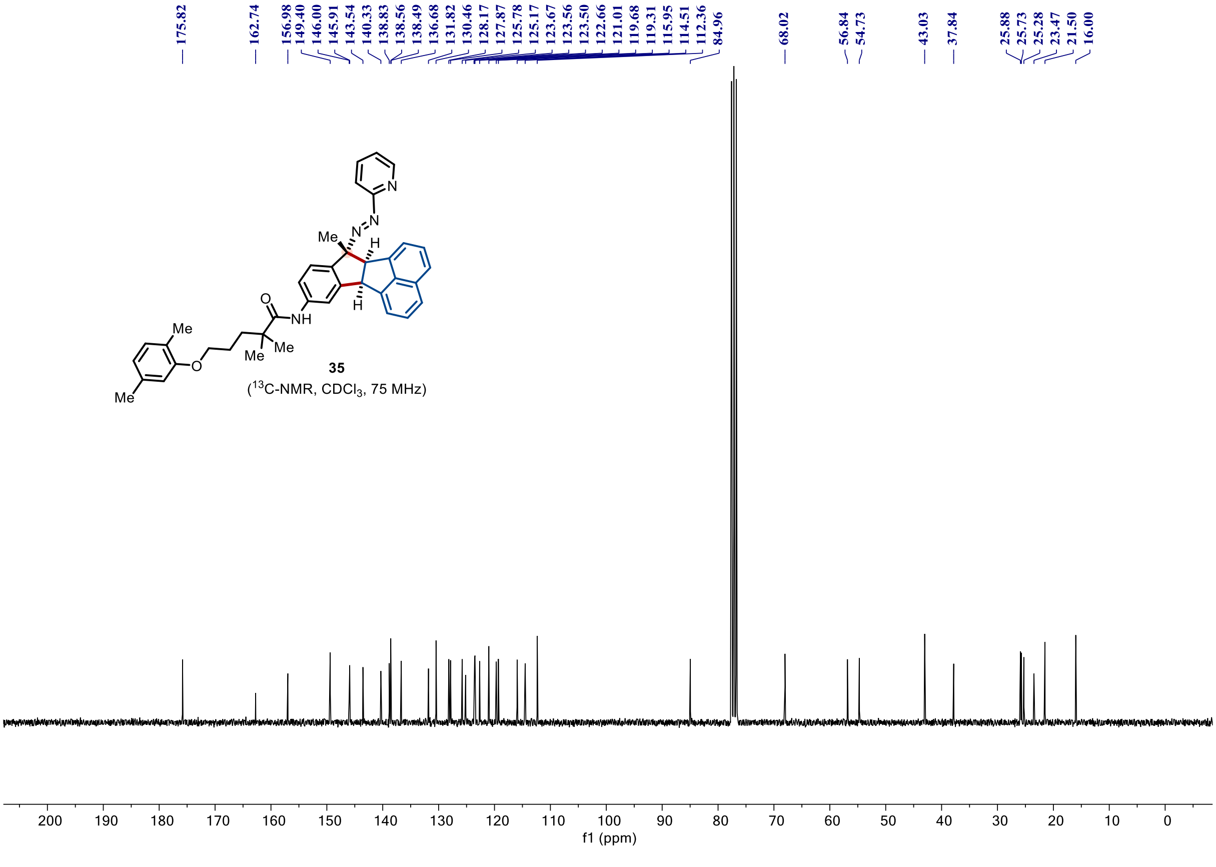
175.82 162.74 156.98 149.40 146.00 145.91 143.54 140.33 138.83 138.56 138.49 136.68 131.82 130.46 128.17 127.87 125.78 125.17 123.67 123.56 123.50 122.66 121.01 119.68 119.31 115.95 114.51 112.36 84.96 68.02 56.84 54.73 43.03 37.84 25.88 25.73 25.28 23.47 21.50 16.00
35
(13C-NMR, CDCl3, 75 MHz)
f1 (ppm)

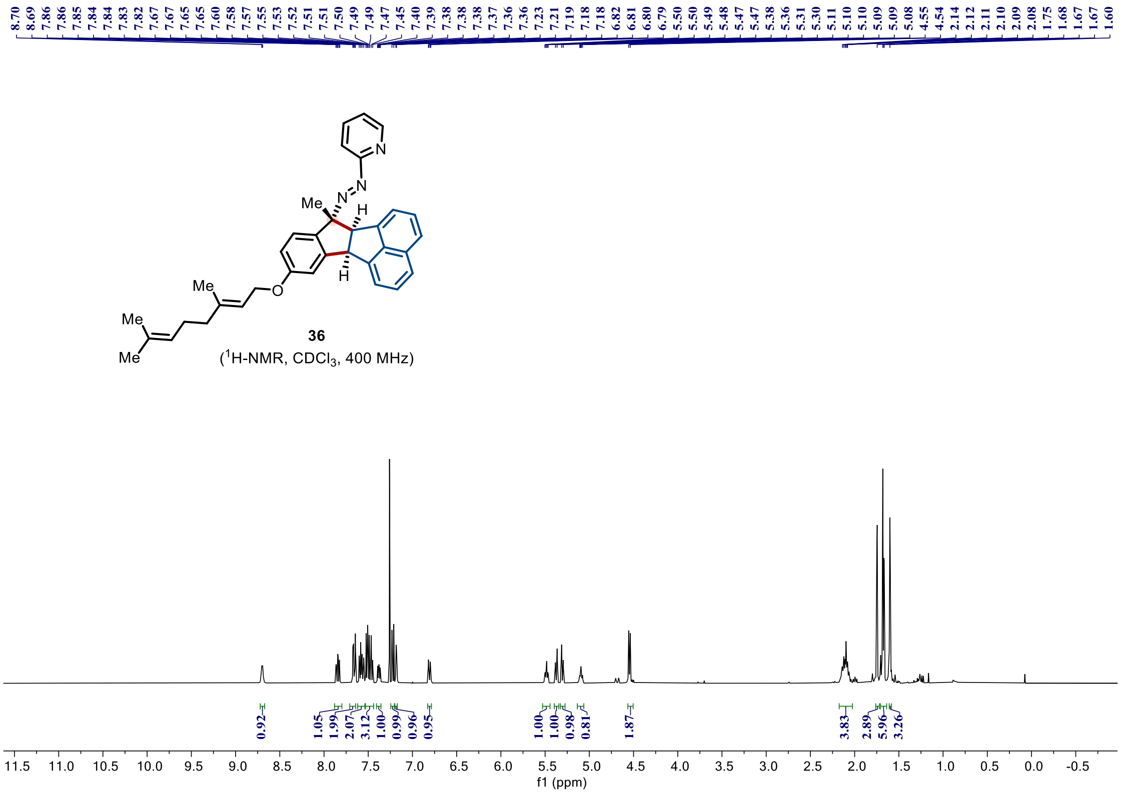
8.70 8.69 7.86 7.85 7.84 7.84 7.83 7.82 7.67 7.67 7.65 7.65 7.60 7.58 7.57 7.55 7.53 7.52 7.51 7.51 7.50 7.49 7.49 7.47 7.45 7.40 7.39 7.38 7.38 7.38 7.37 7.36 7.36 7.23 7.21 7.19 7.18 7.18 6.82 6.81 6.80 6.79 5.50 5.50 5.49 5.48 5.47 5.47 5.38 5.36 5.31 5.30 5.11 5.10 5.10 5.09 5.09 5.08 4.55 4.54 2.14 2.12 2.11 2.10 2.09 2.08 1.75 1.68 1.67 1.60
N
N
N
Me
H
H
O
Me
Me
Me
36
(1H-NMR, CDCl3, 400 MHz)
0.92 1.05 1.99 2.07 3.12 1.00 0.96 0.99 0.95 1.00 1.00 0.98 0.81 1.87 3.83 2.89 5.96 3.26
11.5 11.0 10.5 10.0 9.5 9.0 8.5 8.0 7.5 7.0 6.5 6.0 5.5 5.0 4.5 4.0 3.5 3.0 2.5 2.0 1.5 1.0 0.5 0.0 -0.5
f1 (ppm)

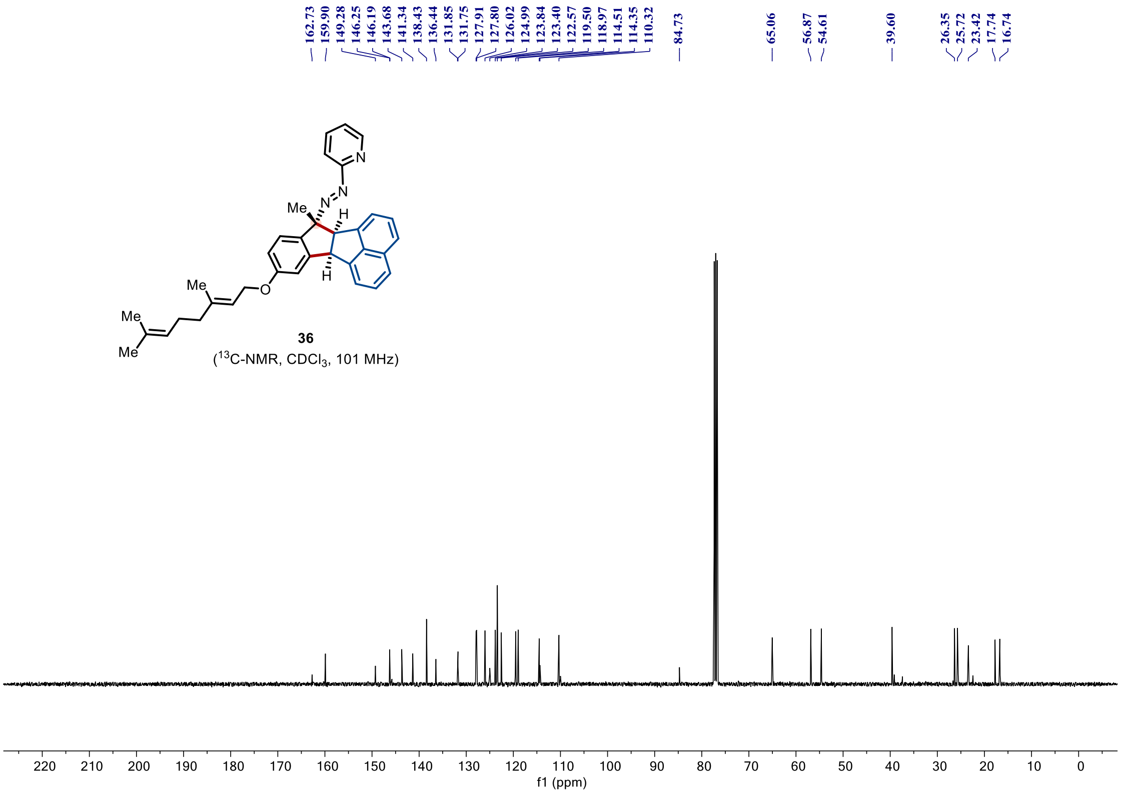
162.73 159.90 149.28 146.25 146.19 143.68 141.34 138.43 136.44 131.85 131.75 127.91 127.80 126.02 124.99 123.84 123.40 122.57 119.50 118.97 114.51 114.35 110.32 84.73 65.06 56.87 54.61 39.60 26.35 25.72 23.42 17.74 16.74
N
N
N
Me
H
H
O
Me
Me
Me
36
(13C-NMR, CDCl3, 101 MHz)
220 210 200 190 180 170 160 150 140 130 120 110 100 90 80 70 60 50 40 30 20 10 0
f1 (ppm)

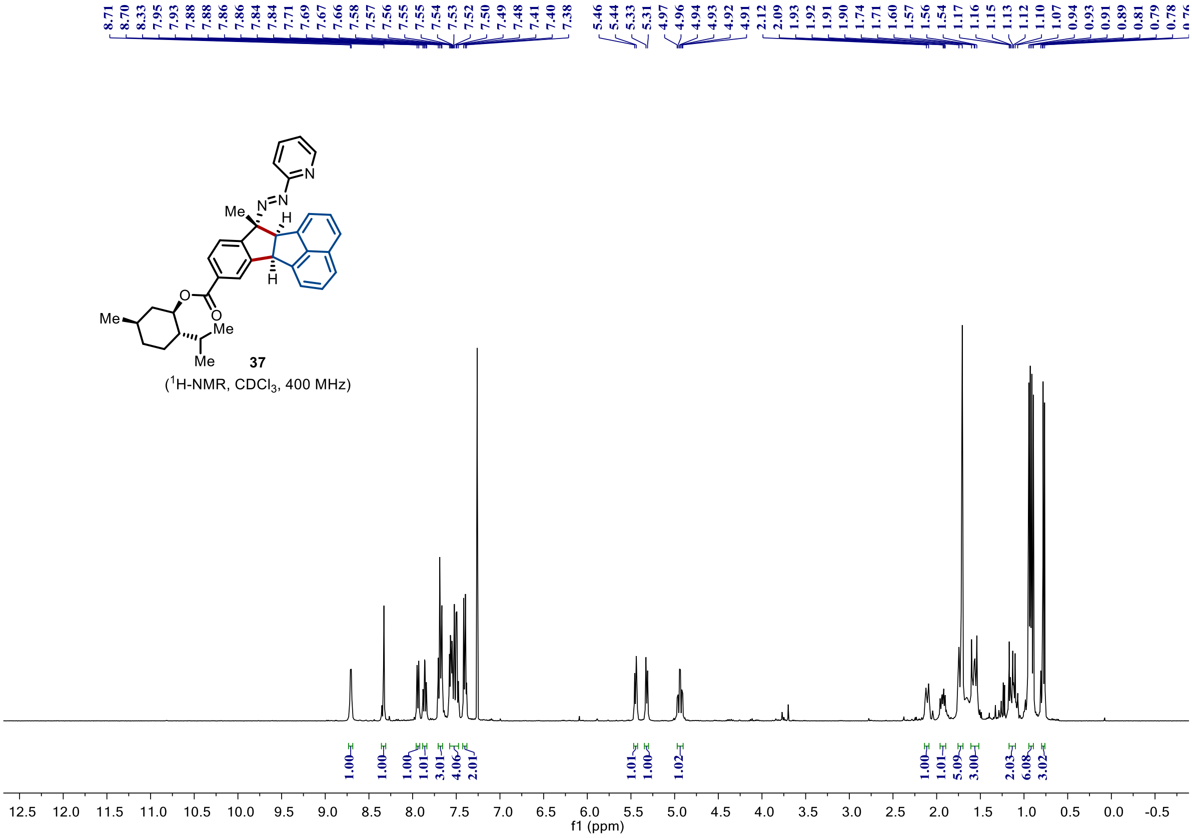

37
($^{1}$H-NMR, $CDCl_3$, 400 MHz)

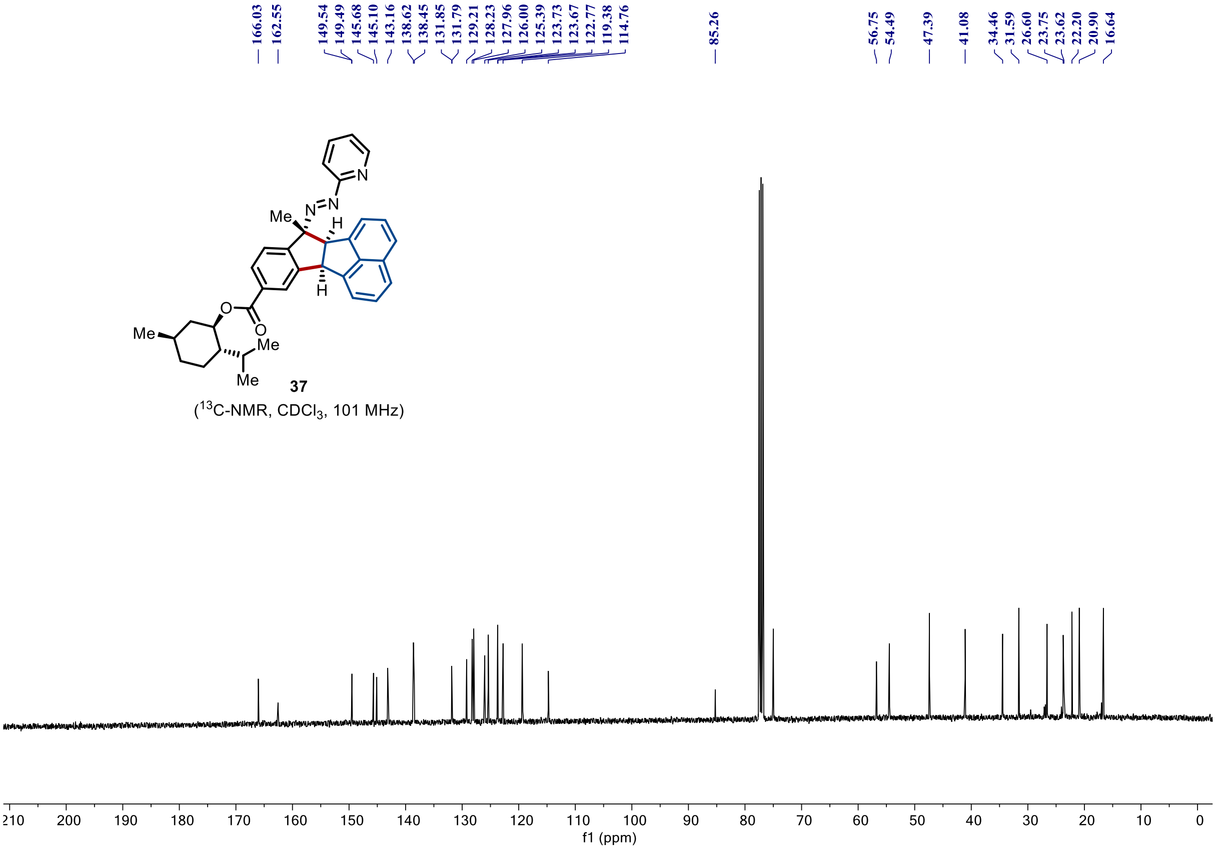

37
($^{13}$C-NMR, $CDCl_3$, 101 MHz)

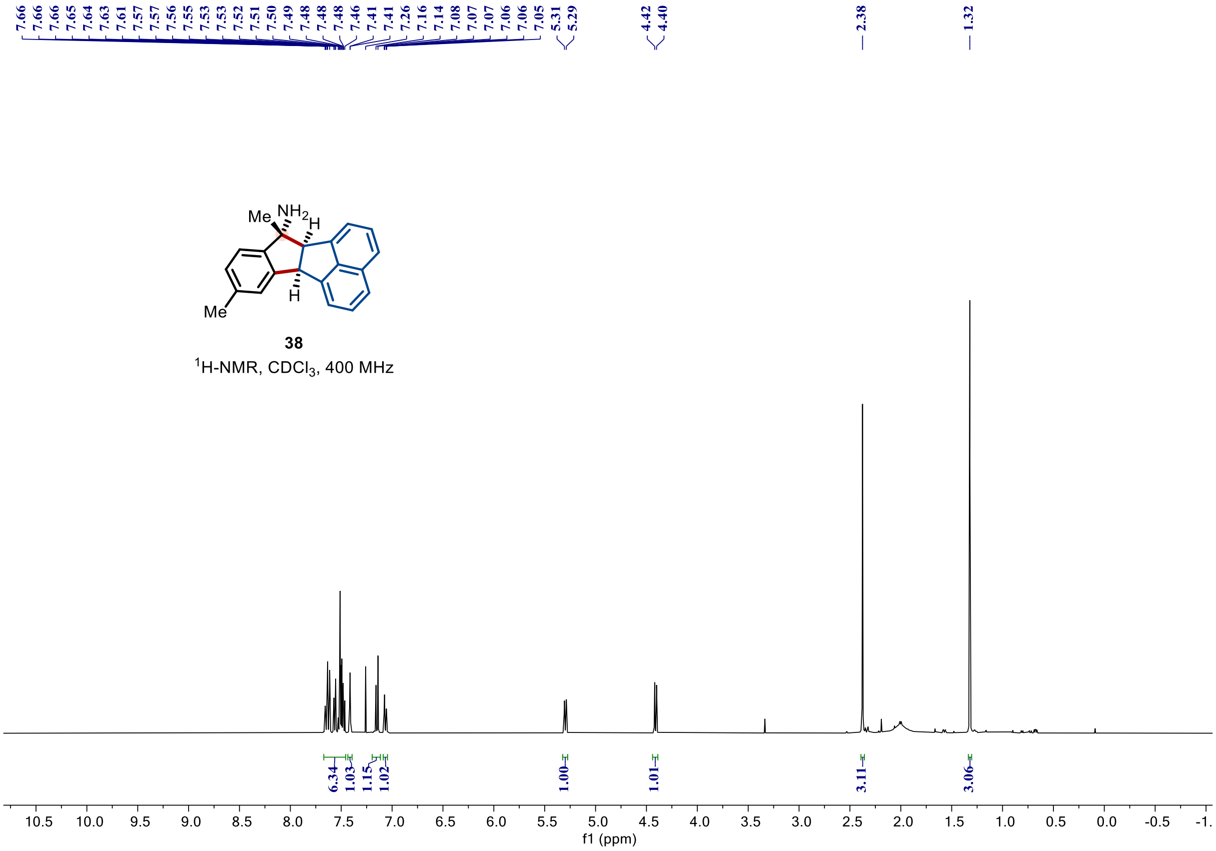
7.66 7.66 7.65 7.64 7.63 7.61 7.57 7.57 7.56 7.55 7.53 7.53 7.52 7.51 7.50 7.49 7.48 7.48 7.48 7.46 7.41 7.41 7.26 7.16 7.14 7.08 7.07 7.07 7.06 7.06 7.05 5.31 5.29 4.42 4.40 2.38 1.32
Me
NH2
H
H
Me
38
1H-NMR, CDCl3, 400 MHz
6.34 1.03 1.15 1.02 1.00 1.01 3.11 3.06
f1 (ppm)

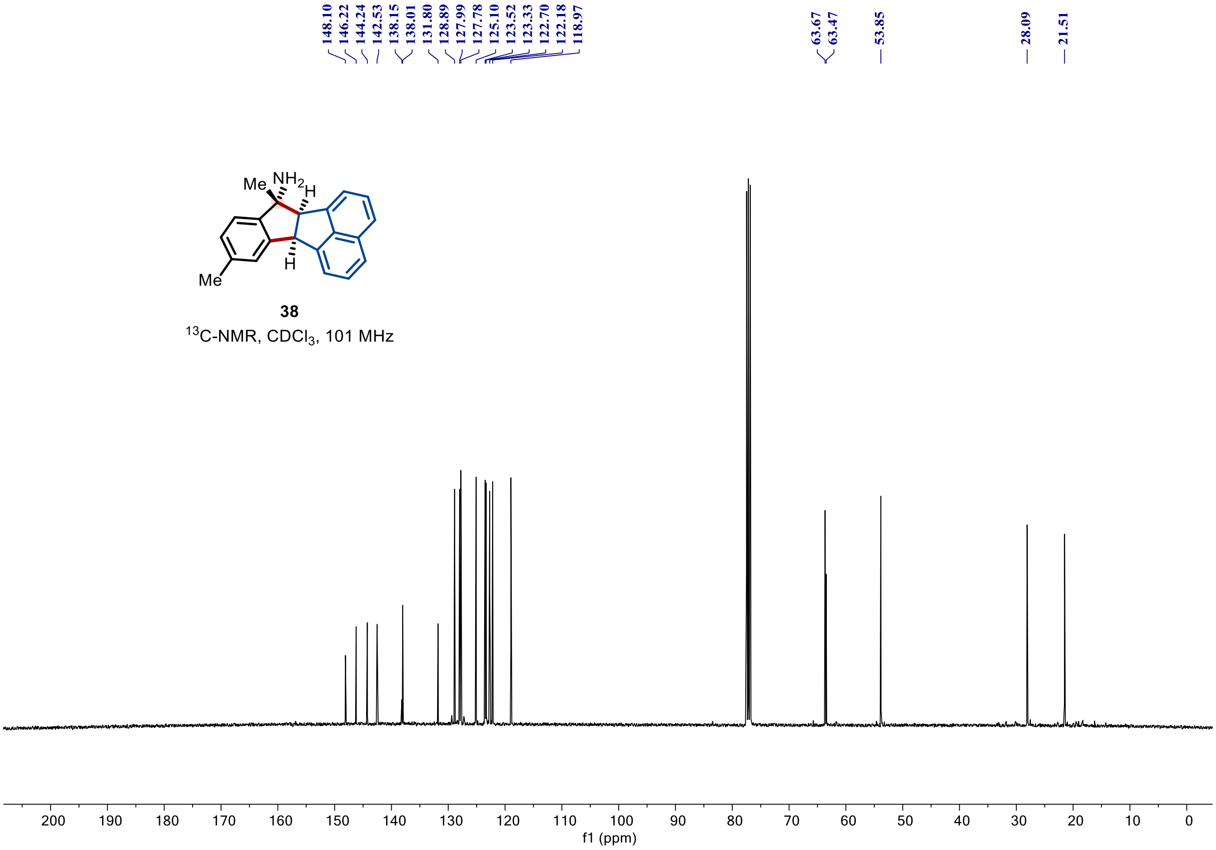
148.10 146.22 144.24 142.53 138.15 138.01 131.80 128.89 127.99 127.78 125.10 123.52 123.33 122.70 122.18 118.97 63.67 63.47 53.85 28.09 21.51
Me
NH2
H
H
Me
38
13C-NMR, CDCl3, 101 MHz
f1 (ppm)

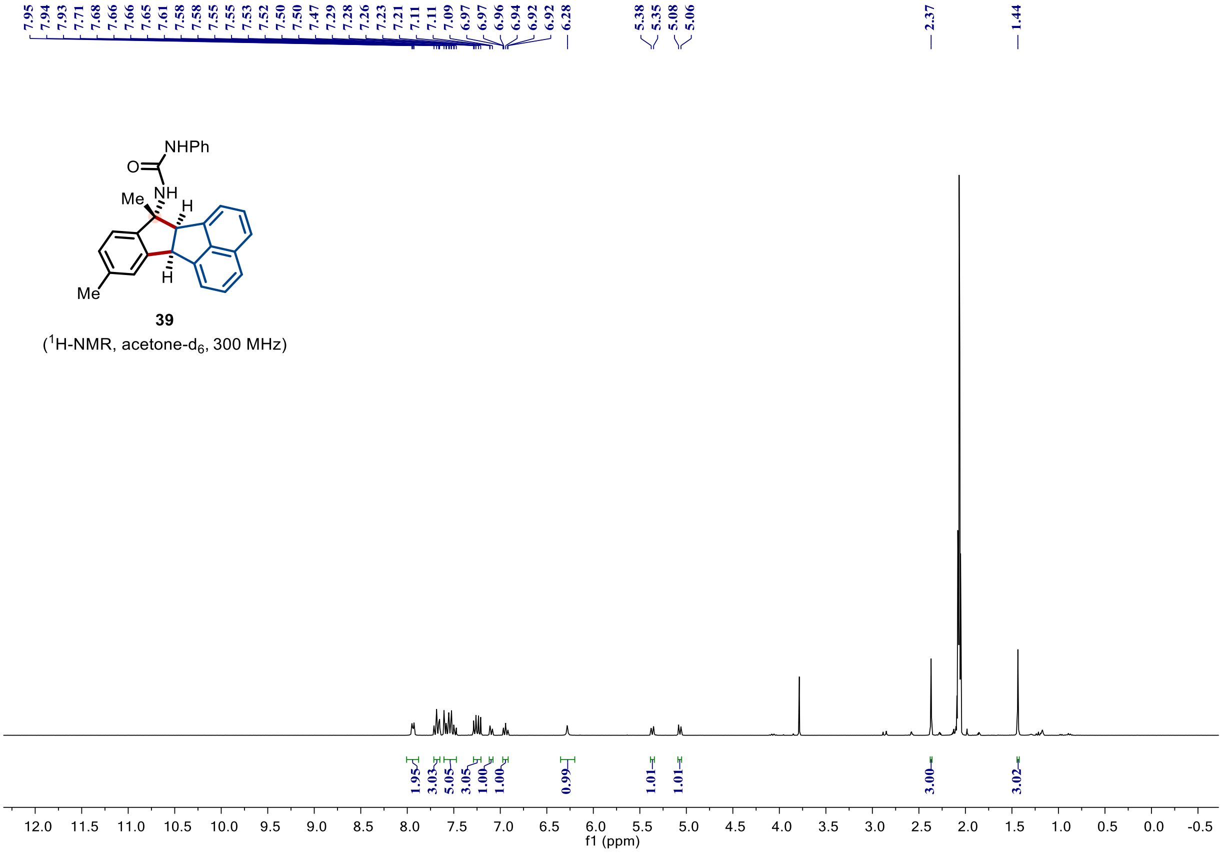
7.95 7.94 7.93 7.71 7.68 7.66 7.66 7.65 7.61 7.58 7.58 7.55 7.55 7.53 7.52 7.50 7.50 7.47 7.29 7.28 7.26 7.23 7.21 7.11 7.11 7.09 6.97 6.97 6.96 6.94 6.92 6.92 6.28
5.38 5.35 5.08 5.06
2.37
1.44
NHPh
O
NH
Me
H
H
Me
39
($^{1}$H-NMR, acetone-$d_6$, 300 MHz)
1.95 3.03 5.05 3.05 1.00 1.00 0.99 1.01 1.01 3.00 3.02
12.0 11.5 11.0 10.5 10.0 9.5 9.0 8.5 8.0 7.5 7.0 6.5 6.0 5.5 5.0 4.5 4.0 3.5 3.0 2.5 2.0 1.5 1.0 0.5 0.0 -0.5
f1 (ppm)

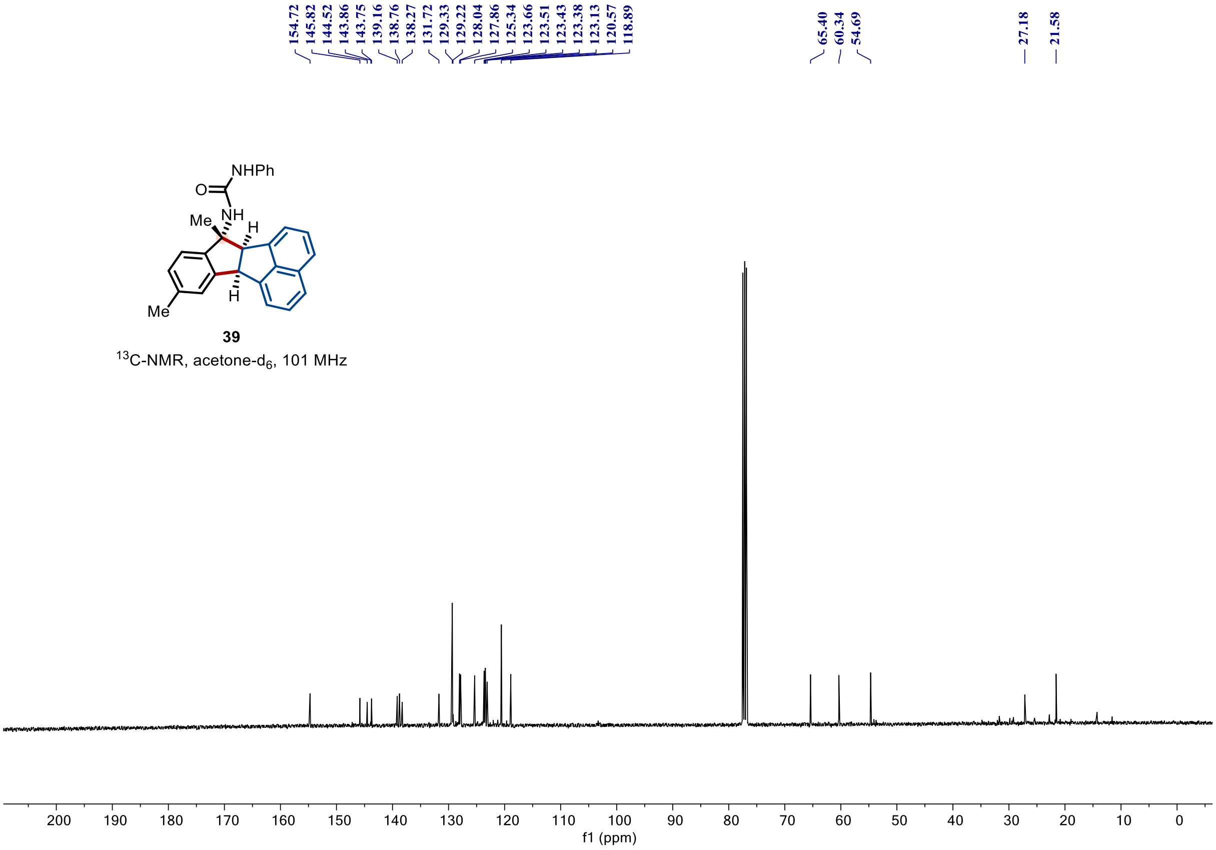
154.72 145.82 144.52 143.86 143.75 139.16 138.76 138.27 131.72 129.33 129.22 128.04 127.86 125.34 123.66 123.51 123.43 123.38 123.13 120.57 118.89
65.40 60.34 54.69
27.18 21.58
NHPh
O
NH
Me
H
H
Me
39
$^{13}$C-NMR, acetone-$d_6$, 101 MHz
200 190 180 170 160 150 140 130 120 110 100 90 80 70 60 50 40 30 20 10 0
f1 (ppm)

# Representative 2D NMR Spectra of the Synthesized Compounds

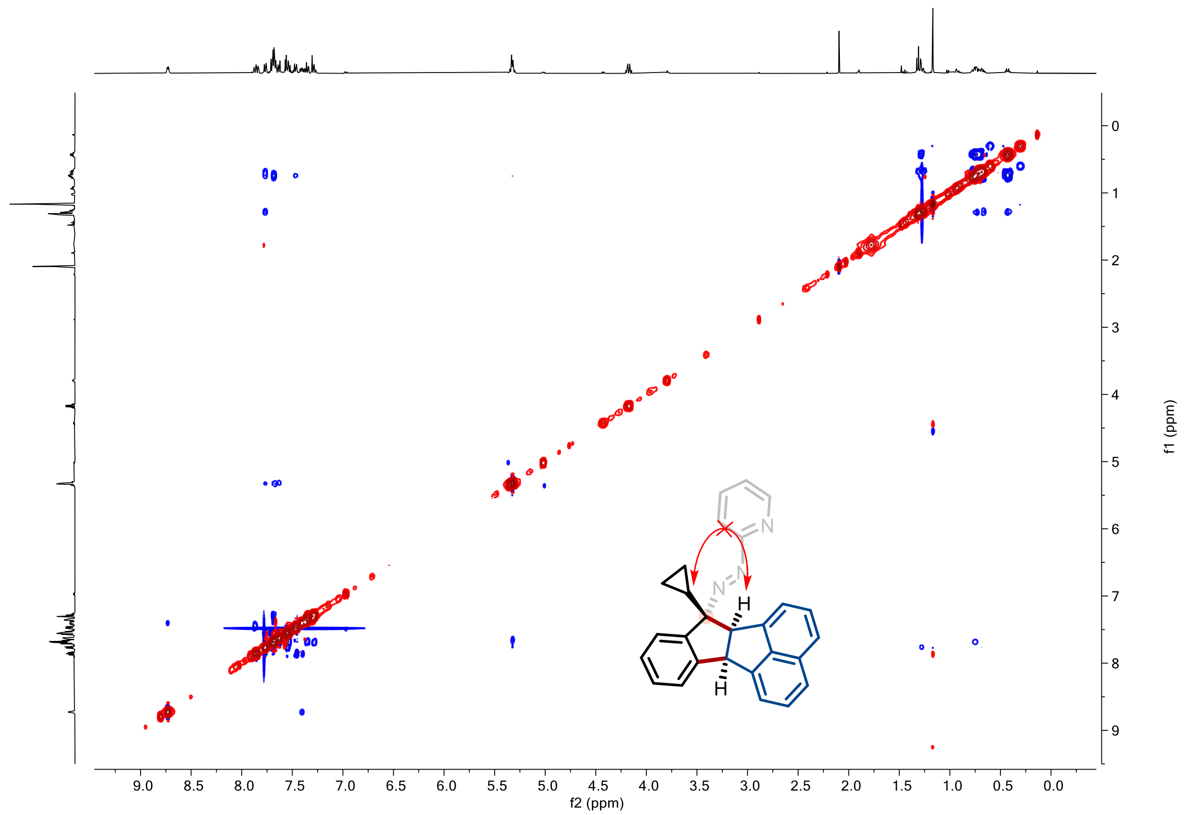


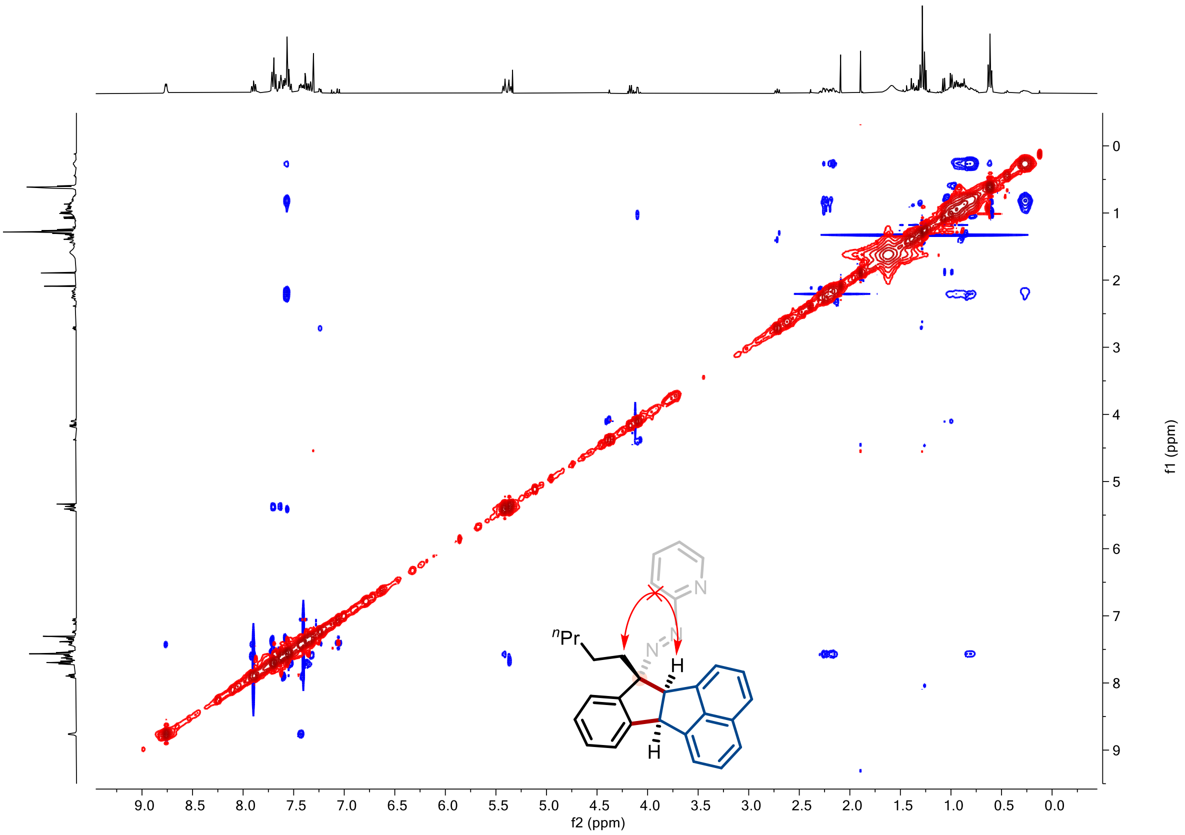

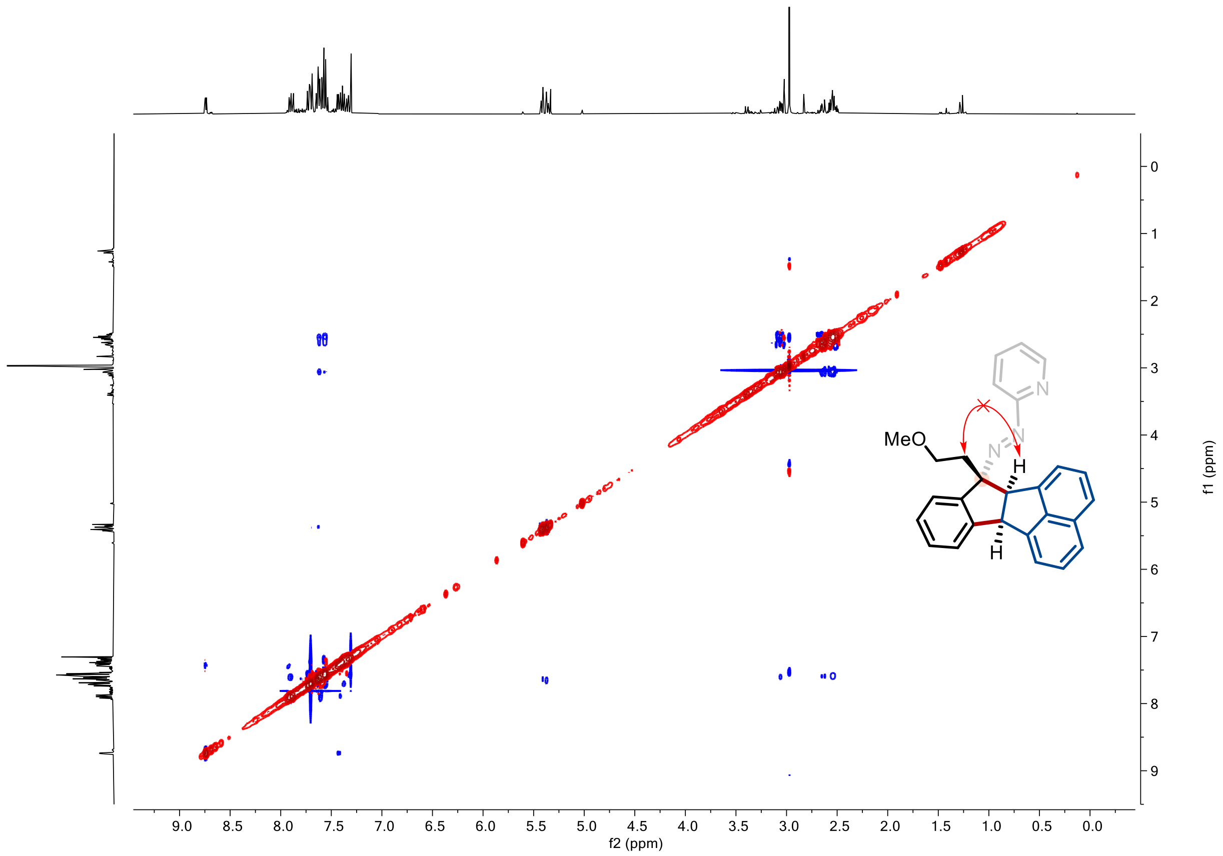
MeO
N
N
N
H
H
f2 (ppm)
f1 (ppm)

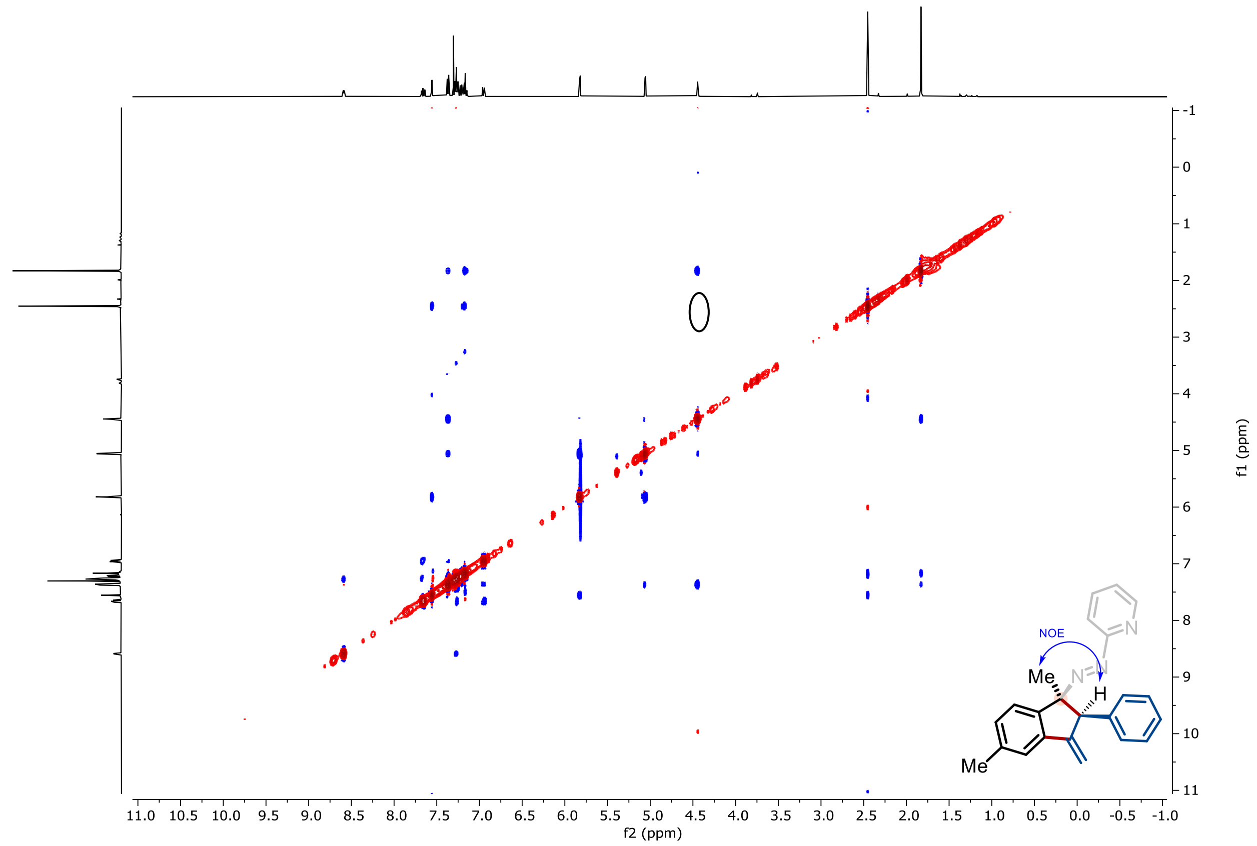
NOE
Me
N
N
H
Me
f2 (ppm)
f1 (ppm)

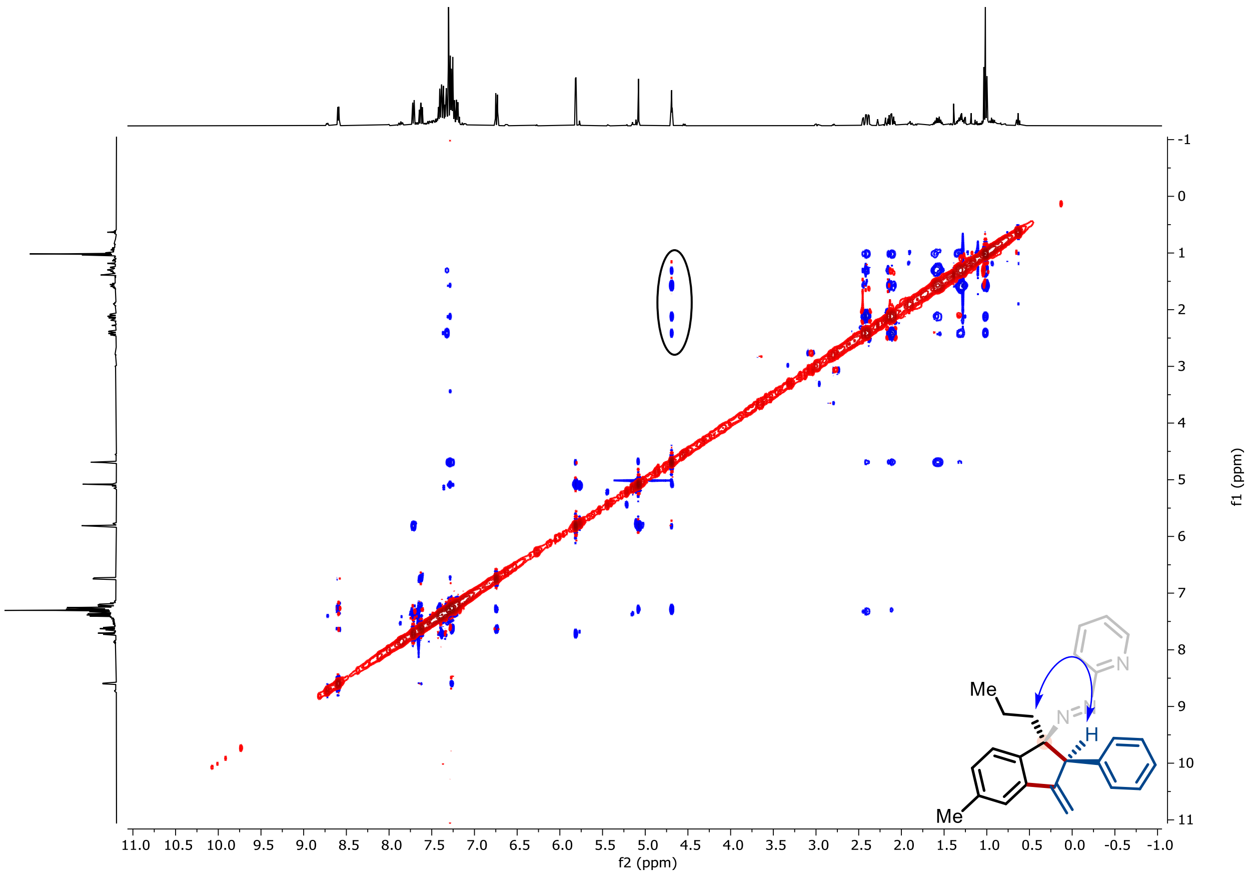
f2 (ppm)
f1 (ppm)
Me
Me
N
N
N
H

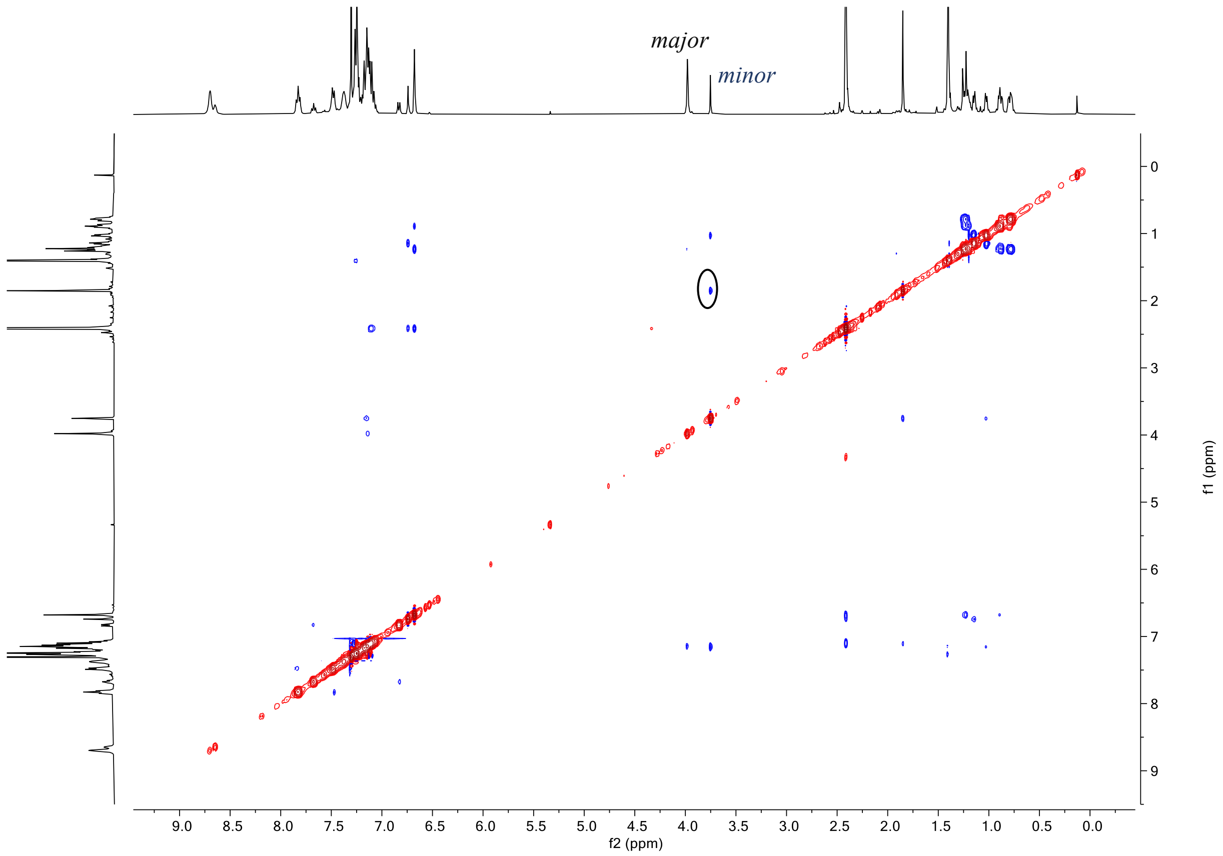
major
minor
f1 (ppm)
f2 (ppm)

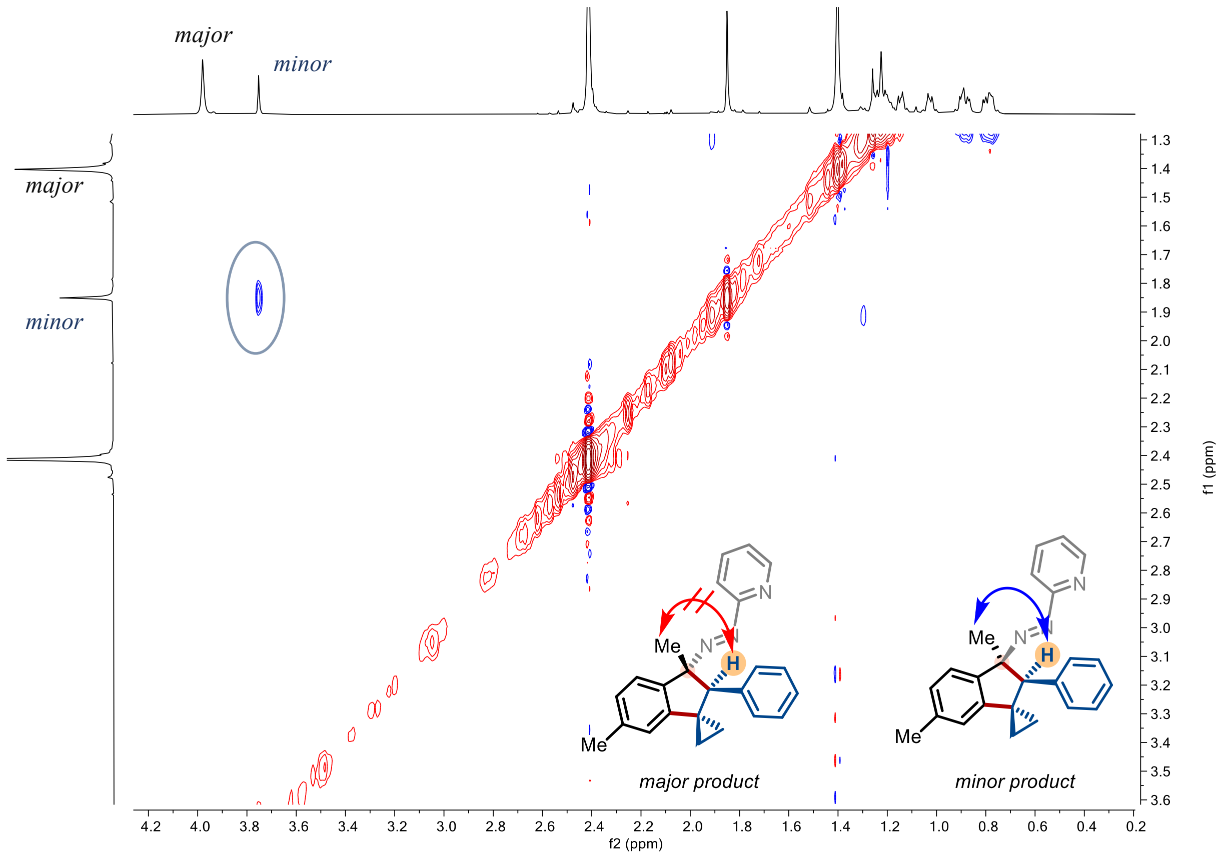
major
minor
major
minor
f1 (ppm)
f2 (ppm)
Me
N
N
N
H
Me
major product
Me
N
N
N
H
Me
minor product
